
JOURNAL INFORMATION
==============================
NLM Title Abbreviation: Trials
Journal ID: Trials
NLM Journal ID: 101263253

Trials

EISSN: 1745-6215
Publisher: BMC

ARTICLE INFORMATION
==============================
PMCID: PMC5425908
DOI: 10.1186/s13063-017-1902-y
Article ID: 1902
Article version: 1
Subjects: Meeting Abstracts

Meeting abstracts from the 4th International Clinical Trials Methodology Conference (ICTMC) and the 38th Annual Meeting of the Society for Clinical Trials

Liverpool, UK. 07–10 May 2017

Electronic publication date: 2017 May 8
Publication date: 2017
Volume: 18
Issue: Suppl 1
Issue sponsor: The publication charges for this supplement was not funded by any sponsorship.
Electronic Location ID: 200
Copyright: © The Author(s). 2017
License: Open AccessThis article is distributed under the terms of the Creative Commons Attribution 4.0 International License (http://creativecommons.org/licenses/by/4.0/), which permits unrestricted use, distribution, and reproduction in any medium, provided you give appropriate credit to the original author(s) and the source, provide a link to the Creative Commons license, and indicate if changes were made. The Creative Commons Public Domain Dedication waiver (http://creativecommons.org/publicdomain/zero/1.0/) applies to the data made available in this article, unless otherwise stated.
License URL: https://creativecommons.org/licenses/by/4.0/

Erratum in: Correction to: Meeting abstracts from the 4th International Clinical Trials Methodology Conference (ICTMC) and the 38th Annual Meeting of the Society for Clinical Trials 19 9 2 2018 99 Trials PMC5807775
Conference name: 4th International Clinical Trials Methodology Conference (ICTMC) and the 38th Annual Meeting of the Society for Clinical Trials
Conference Acronym: ICTMC-SCT 2017
Conference location: Liverpool, UK
Conference date: 07-10 May 2017
issue-copyright-statement: © The Author(s) 2017

==============================

### Poster Presentations

#### P1

**Ideal framework and recommendations: a literature review of its utilization by surgical innovators since 2009**

Joshua Feinberg[1], Claudia Ashton[1], Allison Hirst[1], Christopher Pennell[2], Peter McCulloch[1]
[1]University of Oxford; [2]Maimonides Medical Center
**Correspondence:** Joshua Feinberg
*Trials* 2017, **18(Suppl 1):**P1

**Background**

The evaluation of innovation in surgery is a complex process challenged by evolution of technique, operator learning curves, inconsistent procedural quality, and strong treatment preferences among patients and clinicians [1]. Given these challenges, the development of early-stage novel surgical techniques has been criticized for poor-quality study methodology and data reporting [2, 3]. To address this, the IDEAL framework (Idea, Development, Exploration, Assessment, Long-term follow-up) proposes a five stage stepwise evaluation of innovative procedures to allow a more transparent and ethical introduction of new techniques [4]. The IDEAL framework was proposed in 2009 and there has been no systematic assessment of its use. We examine the uptake and utilization of IDEAL by surgical innovators by reviewing the published literature.

**Methods**

We searched Web of Science to identify all articles published between 1st January 2009 and 30th September 2016 that cited any of the 11 key papers published by the IDEAL Collaboration. All abstracts were assessed by two independent researchers to identify papers explicitly describing using IDEAL recommendations to conduct their primary research. Included papers were reviewed and categorized by characteristics including clinical specialty area, type of journal, country of origin, publication date, and the IDEAL stage. Each paper was further critiqued on how well it met the specified IDEAL stage recommendations [1].

**Results**

We identified 311 papers citing one or more of the 11 key IDEAL papers. Of these, 30 described having followed the stage-appropriate IDEAL recommendations to conduct their innovation study. Interim analysis indicates considerable variation in uptake between clinical specialties and geographical regions. We are currently undertaking more in-depth analysis on the studies of these early users of IDEAL to examine how the framework and recommendations have been used. We also plan to conduct qualitative research with the Pis of these studies to learn more about how useful they found IDEAL as a tool for their research plan.

**Discussion**

Since its inception in 2009, surgical researchers worldwide are beginning to recognize and utilize the IDEAL recommendations. Early adopters have been concentrated within a few surgical specialties and focused on the pre-RCT developmental stages of IDEAL, where research guidance has previously been lacking. This review of the literature will help the IDEAL Collaboration to learn from the early adopters' experiences and identify how to work with future surgical innovators to develop IDEAL as a practical framework in order to conduct the highest quality surgical research.

#### P2

**Development of a complex exercise intervention for prevention of shoulder dysfunction in high-risk women following treatment for breast cancer: prevention of shoulder problems trial (PROSPER)**

Helen Richmond[1], Cynthia Srikesavan[2], Esther Williamson[2], Jane Moser[3], Meredith Newman[3], Sophie Rees[1], Sarah E Lamb[4], Julie Bruce[1]
[1]University of Warwick; [2]University of Oxford; [3]Oxford University Hospitals NHS Foundation Trust; [4]University of Oxford/University of Warwick
**Correspondence:** Helen Richmond
*Trials* 2017, **18(Suppl 1):**P2

**Background**

Shoulder dysfunction and pain following breast cancer treatment is common, impacting upon postoperative quality of life. Exercise may improve shoulder function and reduce the risk of postoperative

BioMed Central complications. However, there is uncertainty around the optimal timing (commencement) and exercise dosage (frequency, intensity, length of time and type of exercise) required for optimal results. We considered Medical Research Council (MRC) guidance for the development of a complex intervention, which highlights the need for a planned, phased approach based on available evidence, appropriate theoretical principles and thorough piloting. We developed a complex intervention for the prevention of shoulder dysfunction following breast cancer treatment for evaluation within the framework of a large pragmatic multicentre randomised controlled trial in the UK NHS setting.

**Methods**

Patient and public involvement (PPI) was central to the development of the PROSPER intervention from its inception. We engaged PPI members from the initial application phase and have had ongoing input throughout the project. In conjunction with PPI, development work began with a comprehensive literature review to identify systematic reviews and RCTs of shoulder-specific exercises and general physical activity during and after breast cancer treatment. This provided the broad theoretical basis for the content of a structured exercise programme which was further developed and refined in a workshop with clinical experts, researchers, and patient representatives. Individual face-to-face interviews were then conducted with seven women previously treated for breast cancer, providing feedback on intervention content and patient-facing materials. The PROSPER intervention was pilot tested with 18 women newly diagnosed with breast cancer, at three hospital sites, allowing further refinement to ensure feasibility for delivery within the UK NHS.

**Results**

The literature review identified several systematic reviews and new clinical trials suggesting that early structured exercise, started within a few days of surgery, versus delayed exercise may improve shoulder range of movement (ROM) in the short and long term. Evidence suggested that shoulder flexion and abduction be restricted to 90 degrees for the first postoperative week to reduce risk of increased wound drainage. There was also evidence to suggest that postoperative strength training was safe and that general physical activity can enhance physical and psychological recovery. The final PROSPER exercise intervention, underpinned by evidence, comprised of three main components: specific exercises targeting shoulder range of motion and upper arm muscle strength, general physical activity, and behavioural strategies to improve adherence. The exercise programme is structured, individualised, supported by trained physiotherapists, and delivered over a 12-month period with a focus upon self-management at home. Women randomised to the exercise programme receive three face-to-face sessions with a physiotherapist, with the option of a further three appointments that can be delivered either face-to-face or by telephone.

**Conclusions**

We followed the MRC theoretical framework to develop a multicomponent exercise programme for the prevention of shoulder problems following breast cancer treatment. This complex intervention is currently being evaluated within a large UK pragmatic RCT [ISRCTN35358984]. To date, 105 women with newly diagnosed breast cancer have been recruited from 12 centres across England.

### P3

#### Intervention development and treatment success in randomised controlled trials of rehabilitation

Jacqueline Hill, Claire Pentecost, Katie Finning, Angelique Hilli, David A. Richards, Victoria A. Goodwin
University of Exeter
**Correspondence:** Jacqueline Hill
*Trials* 2017, **18(Suppl 1):**P3

**Background**

Randomised controlled trials (RCTs) of physical rehabilitation interventions evaluate multi-faceted interventions that are delivered in complex healthcare systems. In a synthesis of the outcomes of trials of rehabilitation interventions funded by the UK National Institute for Health Research – Health Technology Programme (NIHR-HTA), we found that very few new interventions achieved superior outcomes relative to controls. To date, no research has examined why so few rehabilitation interventions that undergo testing in RCTs result in effective new treatments.

**Aim**

(1) To establish work that has been undertaken to develop physical rehabilitation interventions prior to testing in an NIHR-HTA funded RCT. (2) To examine the relationship between intervention development and the primary outcome of experimental testing.

**Methods**

We included 15 superiority RCTs funded by NIHR-HTA from 1997 to July 2016, that evaluated a physical rehabilitation programme and reported their main findings in a peer-reviewed journal or NIHR-HTA monograph. We extracted data on intervention development in respect of five areas described by the credeci 2 reporting criteria for “development” and “feasibility & piloting”: (i) description of the intervention’s underlying theoretical basis; (ii) description of all of the intervention components; (iii) illustration of any intended interactions between different components; (iv) description of the pilot test and its impact on the definitive intervention; (v) consideration of the context’s characteristics in intervention modelling. We coded the extracted data thematically. We classified primary outcome data into one of six categories developed by Djulbegovic et al. (2008) to differentiate studies where outcomes favour the intervention, the control, demonstrate no difference between trials arms and are conclusive or inconclusive. To examine the relationship between intervention development and primary outcome data, we are applying novel mixed methods analytical techniques. We are combining the narrative data on intervention development with the numeric data on treatment outcomes in a joint category/themes display: for each category defined by Djulbegovic et al. we will present a summary of the thematic data on intervention development in each trial for whom the category applies. In this way, we will compare the intervention development work that has been undertaken for trials that result in different outcomes.

**Results**

We found that four trials were significantly in favour of the new treatment, one was significantly in favour of the control, eight had a true negative outcome and two were inconclusive. Our preliminary data extraction reveals that the amount of (reported) intervention development work undertaken prior to experimental testing differs considerably. We are now applying our mixed methods analytical procedures to investigate the relationship between outcomes and intervention development.

**Conclusions**

We have applied techniques for mixed methods analysis in innovative ways to explore the relationship between intervention development and treatment effects. This work may help us better understand the role of intervention development in explaining why so few interventions in rehabilitation that undergo experimental testing result in effective new treatments. Other factors, including the design and conduct of fully-powered trials, may also help explain the relatively few number of treatment successes and require further research.

### P4

#### Randomised controlled trials and realist evaluation: in what contexts and how?

Rebecca Randell[1], Jon Hindmarsh[2], Joanne Greenhalgh[1], Natasha Alvarado[3], Peter Gardner[1], Dawn Dowding[4], Alexandra Cope[1], Julie Croft[1], Andrew Long[1], Alan Pearman[1]
[1]University of Leeds; [2]King’s College London; [3]Newcastle University; [4]Columbia University
**Correspondence:** Rebecca Randell
*Trials* 2017, **18(Suppl 1):**P4

**Background**

It is widely agreed that, if the aim is to inform policy and practice, randomised controlled trials (RCTs) of complex interventions should be coupled with process evaluations. Realist evaluation provides a strong theoretical foundation to explore complex interventions, using a process of eliciting, testing, and refining stakeholders’ theories of how an intervention works, for whom, and in what contexts. There is debate about the relationship between realist evaluation and RCTs. One concern is that RCTs take place in closely controlled contexts and so do not allow for exploration of how different contexts shape the outcomes of an intervention. In this presentation, we will draw on our experience of undertaking a three-phase realist process evaluation alongside an RCT comparing robotic and laparoscopic surgery to address two methodological questions: (1) To what types of trials can realist evaluation make a meaningful contribution?; and (2) How is that contribution best achieved?

**Methods**

In Phase 1, a literature review identified stakeholders' theories concerning how robotic surgery becomes embedded into practice and its impacts on teamwork. These were refined through interviews with theatre teams across nine hospitals. In Phase 2, the theories were tested through a multi-site case study across four hospitals. Case sites were selected to ensure variation in the theatre teams' experience of robotic surgery, an important contextual factor within the theories. Data were collected using multiple methods: structured and ethnographic observation; video analysis; qualitative interviews; and questionnaires. In Phase 3, interviews were conducted at case sites with staff representing other surgical disciplines, to assess generalisability of the findings.

**Results**

While the RCT delivered important results on outcomes, the findings from the realist process evaluation further enhanced our understanding of the introduction of robotic surgery. The combination of methods deployed enabled us to identify and interrogate a range of perspectives on the differences between robotic and laparoscopic surgery and the ways in which robotic surgery is implemented in different sites. Most strikingly, we were able to capture unanticipated consequences of robotic surgery in terms of impacts on teamwork, along with strategies used to counteract such unanticipated consequences. These issues relate to the introduction of robotic surgery as a surgeon-led process but which is dependent on support at different levels of the organisation. The process evaluation directed our attention to the importance of whole team training, experienced and dedicated teams, and suitably sized operating theatres.

**Conclusions**

Realist evaluation provided a robust framework to identify and test stakeholders' theories on deployment of robotic surgery. The results of this study move beyond the RCT to deliver clear guidance on how to deploy robotic surgery and how to ensure effective teamwork when undertaking robotic surgery. So, realist evaluation can play a valuable role alongside pragmatic rcts of complex interventions that seek to explore effectiveness in a range of contexts, eliciting theories about how contexts shape outcomes and then collecting empirical data to test and refine them. Theory elicitation should happen before the RCT to ensure it secures relevant data to support testing of identified theories.

### P5

#### Evaluation of a state-wide chronic disease management program on health service utilisation using a propensity-matched control group

Laurent Billot[1], Kate Corcoran[1], Alina Mcdonald[1], Gawaine Powell-Davies[2], Anne-Marie Feyer[1]
[1]The George Institute for Global Health; [2]Centre for Primary Health Care and Equity, University of New South Wales, University of Sydney)
**Correspondence:** Laurent Billot
*Trials* 2017, **18(Suppl 1):**P5

This abstract is not included here as it has already been published.

### P6

#### Analysis of the head position in stroke trial (headpost): an international cluster randomised cross-over trial

Laurent Billot, headpost Steering Committee
The George Institute for Global Health
**Correspondence:** Laurent Billot
*Trials* 2017, **18(Suppl 1):**P6

Cluster randomised controlled trials (CRCT) are used extensively in evaluations of healthcare interventions. However, cluster cross-over randomised trials are novel: a recent systematic review identified only 91 such studies [1]. While providing efficiency gains compared to crcts, the cross-over design adds complexity to the design and analyses. To date, the literature has been limited to the analysis of binary outcomes [2,3].

The headpost [4] study is an international multicentre randomised cross-over clinical trial comparing the effectiveness of the lying flat (0°) head position with the sitting up (> = 30°) head position, applied within the first 24 hours of admission to hospital for patients with acute stroke, on functional outcome according to the modified Rankin scale (MRS) by blind assessors at 90 days. A total of 114 sites were allocated either to (a) lying flat head position or (b) sitting up head position as the first intervention, to be applied to up to 70 consecutive stroke patients before crossing over to the other head position. All eligible stroke patients presenting to the hospital from the start date were to be prospectively and consecutively enrolled. The primary outcome was the modified Rankin score, a 7-level ordinal scale between 0 (completely independent) and 6 (dead), which is commonly used in stroke trials.

This presentation outlines the statistical analysis planning and conduct for the headpost study, taking account of its cluster cross-over nature. The primary efficacy analysis was conducted using a hierarchical cumulative logistic regression to allow direct modelling across all levels of clustering by including both random cluster and random cluster-period effects. The implications for the analysis of secondary and safety outcomes as well as strategies for sensitivity analyses and handling of missing data, are to be discussed. The focus will be on practicalities of analysis rather than mathematical aspects of cluster cross-over trials.

### P7

#### Implementing mobile electronic patient reported outcomes (EPRO) in a long-term trial with an aging and diverse population

Ashley Hogan, Nicole Butler, Ashley N. Hogan, Alla Sapozhnikova, Ella Temprosa
George Washington University
**Correspondence:** Ashley Hogan
*Trials* 2017, **18(Suppl 1):**P7

The clinical research enterprise cannot escape the shift from paper case report forms (CRF) to direct data entry of case report forms. This daunting shift has the potential to reduce the workload of clinical sites and provide an environmentally friendly source of data collection while also improving the quality and integrity of the data by removing the chance of transcription errors, minimizing missing data, allowing for real time logic and range checks, and leading to faster database locks. For our long-term multi-center clinical trial, the use of mobile electronic patient reported outcomes (EPRO) for self-administered questionnaires was a clear first step towards this shift implemented within our custom built web-based data collection and management system called MIDAS (Multi-modal Integrated Data Acquisition System). Mobile EPRO for self-administered questionnaires increases flexibility of visit flow, and allows for the collection of sensitive information such as questions about sexual health.

Concern for EPRO implementation and anxiety flourished when making considerations for the aging and diverse population of the trial for whom the use of mobile devices may be less ubiquitous. The aging study population is majority female, and ethnically diverse. During the implementation of EPRO for self-administered questionnaires, we kept in mind the needs of elderly participants with cognitive decline, dexterity problems, and visual impairments as well as the needs of participants who speak English as a second language or those with disabilities in reading and writing.
Data collection via mobile data entry for six self-administered questionnaires began in August 2016 and was accompanied by a survey to assess user acceptability. Of the 300 visits completed to date, 90% used the mobile EPRO version with nearly 100% survey response rate. This presentation will present the results of the survey as well as feedback from participants and staff including utilization rates, overall experience, font size, button size, view preference, ease of use, and whether paper or mobile entry is preferred. We will share results of the survey overall and by demographic subgroups using the ~1,000 visits expected by the time of the presentation.

### P8

#### Optimising primary outcome data collection in a neonatal trial

Alison J. Deary[1], Karen Willoughby[2], Ana Mora[1], Anna Curley[3]
[1]NHSBT Clinical Trials Unit, Long Road, Cambridge, CB2 0PT, UK; [2]Department of Obstetrics and Gynaecology, Box 223, Level 2 Rosie Hospital Robinson Way, Cambridge CB2 OSW, UK; [3]Neonatal Unit, National Maternity Hospital, Holles Street, Dublin 2, Eire
**Correspondence:** Alison J. Deary
*Trials* 2017, **18(Suppl 1):**P8

**Background**
planet-2 (ISRCTN87736839) is an international multicentre trial of platelet count thresholds for prophylactic platelet transfusions in preterm neonates. The trial commenced recruitment in June 2011 and to date 573/660 neonates have been randomised to one of 2 arms. Depending on their allocated threshold, the baby receives a platelet transfusion when platelet counts drops to either below 50x10^9 or 25x10^9. The primary outcome measure for planet-2 is the proportion of patients who either die or experience a major bleed up to and including Study Day 28 (SD28). A cranial ultrasound scan (CUSS) at SD28 is the prime marker for major intracranial bleeds at this point.
**Monitoring the Primary Outcome Data**
In order to monitor the completeness of the primary outcome data, a monthly reporting system was developed by the trial statistician to allow close analysis of data completeness. The reports revealed 17.9% of missing primary outcome data from babies known to be alive at SD28. A large proportion of these did not have a reported SD28 CUSS. This was due to a variety of causes, including transfer out of neonates prior to SD28 from the recruiting site to smaller non-participating units.
**Measures to optimise the data**
The monthly reports allow the TMG to take measures to maximise the completeness of the data obtained from the study. A transfer pack was developed to inform new sites of required information and an accompanying letter provided, to enable PIs to request primary outcome data from colleagues within the new units. The SD28 window was extended from +/−3 days to −5/+10 days of SD28. This was considered by the neonatologists on the TMG, and the independent members of the TSC, to possess the same clinical validity. If no scan had been obtained within the new extended timeframe, our medical experts, or, in some cases, independent expert, were given approval to impute the primary outcome given sufficient supporting clinical evidence.
**Result and Conclusions**
This monthly report system provides the information to allow the trial manager to contact the site teams a few days before each randomised baby reaches SD28 to remind them of the need to perform a CUSS and this has proved very effective in optimising the data completeness.
This piece of work shows the value of the statisticians and data team on the trial management team working together to improve the scientific integrity of the study. Our missing primary outcome data currently stands at approximately 1.4%. Liaising closely with research site teams and maintaining good relationships is crucial to trial success.

### P9

#### Data monitoring committee overseeing multiple international randomised controlled trials

Virginia Chiocchia[1], Susan Dutton[2], Rutger Ploeg[3]
[1]Centre for Statistics in Medicine (CSM) and Surgical Intervention Trials Unit (SITU), University of Oxford; [2]Oxford Clinical Trials Research Unit (OCTRU) and Centre for Statistics in Medicine (CSM), University of Oxford; [3]Nuffield Department of Surgical Sciences, University of Oxford
**Correspondence:** Virginia Chiocchia
*Trials* 2017, **18(Suppl 1):**P9

The COPE (Consortium for Organ Preservation in Europe) include three clinical trials to improve preservation and reconditioning strategies for kidneys and livers procured for transplantation aiming to increase the number and quality of grafts used.
Despite the trials being led at different centres, they are centrally managed from one main centre where the Principal Investigator, the project's governance and management are based. This is one of the reason why it was decided to set up a single 'combined' Data Monitoring Committee to oversee the three trials with a single six-monthly meeting to review all three trials.
This seems to be the most convenient approach in similar situations/settings as it reduces the number of meetings to organise as well as expenses. However, it does not come without difficulties particularly when the same person is preparing the reports for all the studies and multiple sites and/or countries are involved.
The different benefits and challenges experienced will be illustrated in order to provide a helpful reference to anyone that may consider this option in similar situations.

### P10

#### Surveillance of clinical trial performance using centralized statistical monitoring

Eileen Stock[1], Zhibao Mi[2], Kousick Biswas[2], Ilana Belitskaya-Levy[2]
[1]Department of Veterans Affairs; [2]Cooperative Studies Program Coordinating Center, Department of Veterans Affairs
**Correspondence:** Eileen Stock
*Trials* 2017, **18(Suppl 1):**P10

In recent years, a growing trend toward global research has led to randomized controlled trials (RCTs) becoming larger and increasingly more complex. More patients are being enrolled entailing greater use of multisite trials, case report forms (CRFs) are more complicated, and larger budgets are necessary to accommodate for the greater volume of participants and sites involved in a RCT. Centralized statistical monitoring (CSM) is commonly used for guaranteeing data quality by detecting data issues early, such as errors, sloppiness, tampering, and fraud, before significant problems occur. Through off-site central monitoring, onsite monitoring can be more efficiently targeted. Equally important to ensuring data quality is assessing the adequacy of the trial design and performance. Design errors, if not discovered and addressed early, can largely bias study findings and make a trial difficult or impossible to interpret. Poor adherence to design and a lack of oversight can result in an unsuccessful trial with drastic ramifications, including revoking one's clinical and research privileges, funding, and leaving a tarnished reputation. Consequently, the purpose of this research was to apply CSM to the monitoring of various aspects related to the design and performance of a clinical trial.
Study design quality metrics assessed for anomalies included adherence to inclusion and exclusion criteria, recruitment, administration of treatment, blinding, visit scheduling, patient follow-up, data submission, and the reporting of safety measures. Each metric can be evaluated across sites as in a multisite RCT, or across clinicians to assist in identifying potential threats to a trial's performance. A program was developed to apply CSM for monitoring the performance of a clinical trial. CSM was applied monthly, in conjunction with regularly scheduled risk-based monitoring. For continuous measures of trial performance, modified boxplots described distributions, differences in the proportion of outliers were assessed using chi-square analyses, differences by site were examined with analysis of variance (or the non-parametric equivalent) and further assessed using pairwise tests, and homogeneity of variance and sites with outlying or inlying variance were also determined. Confidence bands were used to provide additional monitoring of trial performance. For categorical measures, chi-square analyses and logistic regression were employed.
CSM applied to study design elements can be used to assess trial performance over time throughout the duration of a study. Monitoring trial performance helps to ensure the validity of a study and its design, consistency in reporting across sites and clinicians, and that a study's hypotheses are not being compromised. Continual monitoring of study design quality metrics through CSM enables corrective action to be taken early enough to address any potential threats to the design of a RCT, while also simultaneously improving data quality and the credibility of a study and its findings.

### P11

#### Operationalizing the use of latent variables in the process of determining an ARIC participant's neurocognitive status at visit 6

Sheila Burgard[1], James Bartow[1], Sonia Davis[1], Alden Gross[2], Tom Mosley[3], Richey Sharrett[2]
[1]University of North Carolina; [2]Johns Hopkins University; [3]University of Mississippi Medical Center
**Correspondence:** Sheila Burgard
*Trials* 2017, **18(Suppl 1):**P11

**Background**
Dementia and mild cognitive impairment (MCI) pose a large and increasing health and societal burden on the aging US population. In 1987–1989 the NHLBI-supported prospective epidemiologic Atherosclerosis Risk in Communities (ARIC) study enrolled 15,792 participants from 4 distinct US geographical regions in order to investigate the causes of atherosclerosis and its clinical outcomes, including cognitive function. Since visit 1 in 1987–1989, there have been 4 follow-up visits for the cohort. ARIC is uniquely suited to contribute critical information on the vascular, and potentially preventable, contributions to MCI and dementia of different origins.
**Methods**
The Collaborative Studies Coordinating Center (CSCC) in the Department of Biostatistics at the University of North Carolina serves as the coordinating center for ARIC and provides the infrastructure for the data collection using the CSCC-developed, web-based data management system, Carolina Data Acquisition and Reporting Tool (CDART).
Neurocognitive test data collection in ARIC began at Visit 2 (1990–92) and was repeated in Visits 3 (1993–95) and 4 (1996–98) using 3 neurocognitive tests. An ancillary study was conducted in 2004–06 on a subset of the ARIC cohort where the test battery was expanded. At Visit 5 (2011–13), the expanded neurocognitive test battery was collected on 6538 participants, enabling the investigators to examine cognitive function changes over 24 years, particularly in the areas of memory, language and executive function. A challenge to these longitudinal analyses has been that the neurocognitive measures change over time due to scientific improvements in the instruments. A group of ARIC investigators employed factor analysis to level differing cognitive test batteries over visits to common, comparable measurements in the area of general cognition and the 3 cognitive domains of interest (Gross et.al., Epidemiology vol 26, no 6, 11/2015).
**Objective**
Visit 6 is underway with continued neurocognitive emphasis that will allow quantification of cognitive decline, estimation of the incidence of mild cognitive impairment (MCI) and dementia, and tracking of progression from MCI at V5 to dementia. These measures will be immediately available for comparison to Visit 5 factor scores through an application called from CDART. The behind the scenes programming calculates the Visit 6 factor scores for the cognitive areas of interest allowing for immediate determination of cognitive domain failure and generalized cognitive decline compared to Visit 5 performance, despite the fact that not all participants completed the exact same battery at each visit.
The participants who show failure in at least 1 cognitive domain and significant global cognitive decline from Visit 5 will have additional data collected from a proxy or informant and will undergo a complete data review by members of the classification committee in order to determine neurocognitive status as dementia, MCI, normal, or unclassifiable.

### P12

#### An online tool for exploring recruitment achievability for a feasibility and pilot studies in the UK

Andrew Brand, Nicola Totton, Paul Brocklehurst
Bangor University
**Correspondence:** Andrew Brand
*Trials* 2017, **18(Suppl 1):**P12

The aim of our online tool, is to use openly available data to help inform researchers, in the UK, whether a given target size, is broadly achievable for a feasibility or pilot study investigating a specific health condition. Information obtained from the online tool may also further help determine a suitable recruitment period for a feasibility or pilot study investigating a given health condition.
Ideally, data on the actual sample sizes obtain in the feasibility and pilot studies would have been more informative than the target size data. Unfortunately, we were unable to find an openly available source for this data. However we feel that the target sample size data, along with the recruitment period can provide a rough guide to the achievability of recruitment targets for feasibility and pilot studies. For instance, if a pilot study had a target sample size of 60 and a recruitment period of 18 months for a study investigating a health condition you are interested in, you might want to reconsider running a similar pilot study with a target size of 100 for 6 months. We therefore believe that this data has value in making informed decisions with regard to recruitment for a feasibility and pilot studies.
We identified UK Clinical Trial Gateway (UKCTG) as providing the best source of UK based data to harvest. The UKCTG was set up by the National Institute for Health Research (NIHR) to essentially help recruit people to clinical trials in the UK. Because there were no facilities for downloading data from the UKCTG website, a web scraping methodological was adopted and implemented using R.
Four searches were run on the entire trial record. The following search terms were used: "feasibility trial", "feasibility study", "pilot trial" and "pilot study" and 3039 unique trial records were identified. The unique trial records ids were extracted from the search results and then the trial records were downloaded. Data such as the trial title, target sample size, recruitment start date, recruitment end date, the longitude and latitude of the recruiting centre were then extracted from the records, using regular expressions, and collated into an Excel where open-ended text fields (e.g., target sample size) were manually cleaned.
Shiny, a web application framework for R, was then used to create an online tool to interrogate the data. For various health conditions, specified by the researcher, the tool can be used to obtain descriptive summaries and graphical displays of pilot and feasibility studies for the following factors: period of recruitment, target sample size, target recruitment rate per month, location of trial centres.

### P13

#### Is it possible to randomise patients to potentially not receive a dressing after surgery? Preliminary findings of the NIHR HTA Bluebelle pilot randomised controlled trial

Leila Rooshenas[1], The Bluebelle Study Group[2]
[1]University of Bristol; [2]University of Bristol and University of Birmingham
**Correspondence:** Leila Rooshenas
*Trials* 2017, **18(Suppl 1):**P13

**Background**
Recruiting to randomised controlled trials (RCTs) can be difficult, especially when habitual clinical practices are compared with lesser-known or novel approaches. Surgical RCTs can be particularly challenging, due to ingrained clinician preferences and doctrine. Post-surgical wound care is an aspect of surgery in need of high quality evidence. It is common to apply dressings over closed wounds after most adult operations, despite there being limited evidence to support or refute this practice. The NIHR-funded Bluebelle study aimed to determine the feasibility of an RCT that randomises patients to different wound dressing strategies, including 'no dressing' (where the wound is exposed to air). The funder and health care professionals were sceptical about whether 'no dressing' would be acceptable to patients and clinical professionals, and questioned whether an RCT could successfully recruit participants. The Bluebelle study was thus funded to investigate these uncertainties. It consisted of two phases: a preliminary phase to explore current practice and select appropriate comparators (Phase A), and an external pilot RCT (phase B). Informed by phase A findings, the pilot RCT sought to randomise patients to receiving either a 'simple dressing', 'glue-as-a-dressing', or 'no dressing'. The pilot addressed a number of objectives to determine whether a full-scale RCT could be delivered. Two objectives were to investigate whether recruitment was feasible (target of 330 patients), and explore whether the comparison groups were acceptable to patients and health care professionals.
**Methods**
Adults undergoing elective and emergency abdominal surgery were invited to take part in the pilot RCT. Recruitment took place between March-November 2016, across four NHS hospitals in England. Research nurses and surgeons provided information about the study in advance of surgery and obtained written consent. Patients who entered the RCT and health care professionals involved in their care were invited to take part in semi-structured interviews, to explore the acceptability of the dressing strategies under comparison. Results Recruitment figures met or exceeded targets across all centres. The numbers of patients approached and the proportion consenting indicated that a main trial would be feasible (446 approached, 363 consented, and 326 randomised, as of October 2016). Qualitative interviews provided further evidence to suggest that randomisation to the three dressing strategies was acceptable. Patients' wound healing experiences were similar across all groups, with no notable clinical or practical concerns. Contrary to health care professionals' prior assumptions, some patients reported practical advantages of not having a dressing, reflecting on the 'low maintenance' nature of wound care. Health care professionals did not report any particular difficulties in caring for patients in any of the groups, and did not perceive any changes to other aspects of their practice. The number of recorded protocol deviations and retention rates are currently undergoing analysis and will be available at the conference.
**Conclusion**
This pilot RCT demonstrated that it is feasible to recruit patients to an RCT of different wound dressing strategies, including 'no dressing'. A full-scale trial will be designed on the basis of these findings, providing other aspects of trial conduct (e.g. Retention) are acceptable.

### P14

#### Scaling up: lessons from a feasibility study involving people with type 2 diabetes and their families

Vivien Coates[1], Karen McGuigan[2], Alison Gallagher[1], Brendan Bunting[1], Maurice O'Kane[3], Tracy Donaghy[3], Geraldine Horigan[1], Maranna Sweeney[1]
[1]Ulster University; [2]North West Research, NI; [3]Western Health & Social Care Trust
**Correspondence:** Vivien Coates
*Trials* 2017, **18(Suppl 1):**P14

**Background**
The rapid and recent global increase in prevalence of type 2 diabetes (T2D)[1,2] is of great concern. Although adverse lifestyle behaviours (relating to diet and exercise) are recognised as important risk factors for the development of T2D, interventions at the level of the individual to modify these are challenging. Evidence suggests that lifestyle behaviours are passed through families, from one generation to another. Therefore, when designing T2D interventions, it may be important to consider behaviours developed within the shared family environment.
**Aim**
To investigate the impact of the shared family environment on risk factors for T2D, and to determine the feasibility of conducting a fully powered study using this methodology.
**Design**
Cross-sectional feasibility study of index cases diagnosed with T2D and their first degree relatives (siblings and offspring). Index cases were recruited from the diabetes information database (DIAMOND) of a hospital in Northern Ireland.
**Method**
Sample: The DIAMOND database was screened to identify adults with T2D, aged 45–65 years, with at least two siblings and two offspring, willing to participate in the study. For this feasibility study 50 participants were sought (i.e.10 index cases each with 4 first degree relatives, spanning two generations). Measures: A range of lifestyle factors, biochemical and clinical markers were collected for all participants. Location of the Study: The rationale underpinning the suitability of this location for the study was based on existing knowledge: 1. As Northern Ireland comprises the most homogenous population group in the UK, it was believed the majority of offspring would live locally; 2. The close family structure encountered in Northern Ireland would lead to strong support for research projects that involve a family member.
**Results**
Recruitment: Achieving the required sample of n = 50 proved to be impossible over an 18 month recruitment period. For example, during a four-month screening period, coinciding with a relaxation of inclusion criteria 434 patients were screened, 85 were found to be eligible for inclusion, with only 6 successfully recruited. Only 8 index cases were secured across the study duration.
Support: Family support structures were found to be weak, with a number of eligible candidates reporting strained family relationships as a deterrent to participation.
Family size: Many potential index cases did not have enough siblings and/or children required to participate.
Motivation: Index cases lacked motivation, both in relation to their condition and willingness to participate.
Age: The tight inclusion criteria for age of index cases (45–65 years) were found to be restrictive.
Data analysis: This proved difficult due to the small sample size and the clustering of family data.
**Conclusion**
The feasibility study provided key insights, impacting on scaling up decisions. We now know that identifying index cases for this study through a hospital data base is ineffective and would be better suited to a primary care setting. The data gathering methods and instruments worked effectively. In light of the difficulties encountered in the feasibility study, it was agreed that a fully powered study would not be developed.

### P15

#### 40 is the magic number

Laura Pankhurst, Ana Mora, Alison J. Deary, Dave Collett
NHS Blood and Transplant
**Correspondence:** Laura Pankhurst
*Trials* 2017, **18(Suppl 1):**P15

Feasibility studies are routinely performed in a variety of clinical areas to help provide evidence prior to major monetary investment, human resource and patient recruitment to a large randomised controlled trial (RCT). They can assess a variety of aspects including recruitment potential, multi-centre operational coordination and logistical aspects of administering the intervention. As viability is their main aim, small sample sizes are used and so feasibility studies rarely have sufficient power to assess clinically important treatment differences.

Like other research organisations, NHS Blood and Transplant (NHSBT) considers feasibility studies to be essential prior to significant investment in a subsequent full scale RCT. As such, NHSBT have funded a number of feasibility studies, which have then improved the design and conduct of RCTs and larger research projects that have ultimately lead to changes in clinical practice.

The NHSBT Clinical Trials Unit has a growing portfolio of feasibility studies in transfusion medicine with five studies having a sample size of around the magic number of 40 patients: in set up REAL and DRIVE, currently recruiting REDDS (ISRCTN26088319) and EFIT (ISRCTN67540073); and completed CRYOSTAT (ISRCTN55509212). Although formal sample size calculations are not needed for feasibility studies, it is important that required patient numbers are properly justified. Although there is some guidance on this in the literature (for example Julious (2005), Billingham (2013), Teare (2014) and Whitehead (2016)) the background to the sample size for our feasibility studies will be described and illustrated.

Our magic number of 40 is regarded as a compromise between the need for a short timescale in which feasibility can be assessed, sufficient data to allow recruitment rates to be determined in representative centres, and whether the study interventions can be delivered successfully. Some general observations on the design of these studies will also be included, concluding with a summary of the research which has resulted from our completed feasibility studies.

### P16

#### Key design features of pilot and feasibility studies to inform successful surgical trials: a systematic analysis of funded studies

Katherine Fairhurst[1], Jane Blazeby[2], Shelley Potter[2], Amanda Blatch-Jones[3], Ceri Rowlands[2], Carrol Gamble[2], Kerry Avery[2]

[1]University of Bristol; [2]MRC conduct-II Hub for Trials Methodology Research & Centre for Surgical Research, University of Bristol; [3]National Institute for Health Research Evaluation, Trials and Studies Coordinating Centre (NETSCC), University of Southampton

*Trials* 2017, **18(Suppl 1):**P16

**Correspondence:** Katherine Fairhurst

**Background**

Poor research design, conduct and analysis contribute to significant research waste. This is further compounded by the limited reporting and dissemination of results. Pilot and feasibility work has the potential to contribute to the success of subsequent definitive main trials. It allows areas of methodological uncertainty in the main trial protocol to be addressed and resolved before the main trial begins. Whilst it is particularly important to the design of trials of complex interventions such as surgery, little is known about how to optimally design pilot and feasibility work to inform surgical trials.

**Aim**

To systematically analyse the protocols and published papers of funded pilot and feasibility studies of surgical interventions to understand key design features associated with the optimal design and conduct of main surgical trials.

**Methods**

The NIHR Health Technology Assessment (HTA) and Research for Patient Benefit (rfpb) programme databases (as available from the NIHR website) were screened for pilot/feasibility studies of surgical interventions funded between 2005 and 2015. Pilot/feasibility work was defined as: Any research undertaken before a main study intended to inform the design and/or conduct of a future main study. A surgical intervention was defined as: A diagnostic, therapeutic or adjunctive invasive intervention performed by a trained clinician, using hands, instruments and/or devices. Studies which were not pilot/feasibility work or where the surgical intervention was a co-intervention were excluded. It was rationalised that research funded by the NIHR programmes would embrace the higher quality methodological features necessary to identify the key design features of interest and will have been peer-reviewed as part of the funding process. Protocols for all included studies and the associated data sources were collated, including, where available, published papers from the pilot/feasibility work and the consequent main trial. A data extraction form was developed and piloted a priori enabling elicitation of the pilot/feasibility work rationale, and exploration of the associations of key design features of pilot/feasibility work with the planning, conduct and outcome of any subsequent definitive main trial.

**Results**

1341 studies funded by the HTA and rfpb NIHR programmes between 2005 and 2015 were identified and screened, with 73 (5.4%) meeting the inclusion criteria. 30 (41%) were rcts with an internal pilot phase and 43 (59%) were other feasibility work. This included 28 (65%) randomised pilot studies, 3 (7%) non randomised pilot studies and 12 (28%) other types of feasibility study, of which 8 (66%) were systematic reviews. Further findings, including the rationale for pilot/feasibility work and the associations of key design features with main trial design and/or conduct, will be presented.

**Conclusions**

The findings will inform a qualitative study comprising in depth semi-structured interviews and consensus methods to explore the perceptions and experiences of key stakeholders involved in pilot/feasibility studies of surgical interventions. This work is important to develop future recommendations for the optimal design and conduct of pilot/feasibility work of surgical interventions.

### P17

#### Estimating the cost of prescribed medications in economic evaluation: does the current method reflect the true cost to the English NHS? Evidence from the comet feasibility study

Kirsty Garfield, Matthew J. Ridd, Sandra P. Hollinghurst

University of Bristol

**Correspondence:** Kirsty Garfield

*Trials* 2017, **18(Suppl 1):**P17

**Background**

Economic evaluation guidance states that resource use should be valued using relevant unit costs. The most frequently used source for valuing prescribed medication use in the UK is the British National Formulary (BNF). However, from the perspective of the UK National Health Service (NHS), it is not clear whether this source reflects the true cost to the NHS.

**Methods**

The COMET study sought to determine the feasibility of conducting a randomised controlled trial in young children with eczema. Children were recruited from primary care and randomised to one of four commonly used emollients. The study also explored the feasibility of both collecting and costing the data required to perform an economic evaluation in this setting. As part of this we explored whether published prescribed medication costs from the BNF and Prescription Cost Analysis (PCA) represented the true cost to the NHS. In order to estimate the cost to the NHS we identified the method by which community pharmacies are reimbursed for the medications they prescribe. Unit costs of the four intervention emollients were estimated using this method and compared to unit costs from the BNF and PCA. The total cost of study emollients prescribed over the trial period were also estimated and compared using the different methods.

**Results**

We identified a method for estimating the NHS cost of prescribed medications dispensed by community pharmacists. This method incorporates the basic price of the medication, pharmacy discounts, dispensing fees, payments for consumables and containers, and other associated costs. The unit cost of all intervention emollients estimated using the alternative method were higher than costs listed in the BNF and PCA. The largest difference in unit costs was for Aveeno lotion, whereby the cost listed in the BNF and PCA was £5.33 and the cost estimated using the alternative method was £7.23. The smallest difference was for Doublebase gel at £6.09 in the PCA and £6.22 using the alternative method.

**Conclusions**

Using this method may lead to more accurate estimates of the true cost to the NHS of prescribed medications, however assumptions around pharmacy discounts were required to estimate costs. Estimating costs using this method is more time intensive when compared to applying published unit costs from the BNF or PCA. Whilst using this method for intervention medications can provide sensitivity analyses around intervention costs, the value added of using this method to cost concomitant medications may be limited when considering the researcher time required.

The COMET study was independent research funded by the National Institute for Health Research (Research for Patient Benefit Programme, PB-PG- 0712–28056). The views expressed in the publication are those of the author(s) and not necessarily those of the NHS, the National Institute for Health Research or the Department of Health.

### P18

#### Methods for extrapolation from clinical trial data to inform economic evaluations: a taxonomy

Iryna Schlackow[1], Alastair Gray[2], Linda Sharples[3], Chris Jackson[4], Nicky Welton[5], Borislava Mihaylova[2]
[1]University of Oxford; [2]Nuffield Department of Population Health, University of Oxford); [3]Leeds Institute of Clinical Trials Research, University of Leeds; [4]MRC Biostatistics Unit, Cambridge; [5]School of Social and Community Medicine, University of Bristol
**Correspondence:** Iryna Schlackow
*Trials* 2017, **18(Suppl 1):**P18

**Background**

Lifetime economic evaluations are often performed alongside randomised clinical trials, to incorporate long-term effects of interventions. However, due to the limited duration of most randomised controlled trials, extrapolation of components such as survival, beyond study data is required.

**Aim**

To review extrapolation methods that are currently used in economic evaluations and to provide a taxonomy of these methods while discussing motivation, advantages and limitations behind each approach in the context of a cost-effectiveness framework.

**Methods and interim results**

A pearl growing strategy was applied to identify manuscripts that contained novel extrapolation methods, with the emphasis on methods largely based on a single randomised clinical trial. Firstly, a scoping search of the PubMed database was performed to identify recent methodological papers. Subsequently, reference lists of included manuscripts were checked, and finally, a panel of experts was asked to suggest further potentially relevant published methods. Method description was extracted using a pre-defined template. Extracted information included the context that motivated method development (e.g. the need to incorporate cause-specific mortality); type of data used for the extrapolation (e.g. from an RCT, general population or a matched cohort); detailed statistical/modelling methodology; comments on generalisability and usability (e.g. necessary assumptions, incorporation of uncertainty and sensitivity analyses, compatibility with a cost-effectiveness framework, implementation in standard software); main strengths and comparison with other methods. A reviewers' opinion, based on a consensus between at least two reviewers, was provided on whether the method accommodated aspects commonly of interest in cost-effectiveness analyses, such as heterogeneous population as well as the main driver behind the extrapolated survival (eg major nonfatal adverse events).

Based on the identified manuscripts, and the reviewers' comments, a taxonomy of methods will be suggested, with methods classification based on the main driver of survival (e.g. a single cause of death, cause-specific mortality, non-fatal disease events or other disease markers); underlying epidemiological disease model (e.g. Natural history of the disease and competing risks); and assumptions about the treatment effect over time. Interdependence between these factors, with the appropriateness, advantages and limitations of each approach and implications for performing cost-effectiveness analyses will be discussed.

**Conclusions**

The choice of an appropriate method depends on a range of factors, including presence of competing risks, specifics of the disease natural history and assumptions on the treatment effect. Care must be taken in understanding the available options and their limitations prior to embarking on extrapolation. The increase in availability of relevant data is likely to contribute to emergence of novel approaches to support extrapolation efforts.

### P19

#### Cost-effectiveness analysis of clinical trials with missing data: using multiple imputation to address data missing not at random

Baptiste Leurent, Manuel Gomes, James Carpenter
London School of Hygiene & Tropical Medicine
**Correspondence:** Baptiste Leurent
*Trials* 2017, **18(Suppl 1):**P19

**Background**

Cost-effectiveness analysis (CEA) of randomised controlled trials provide key evidence to inform health care decision making. Missing data is a particularly challenging issue in CEA because a large proportion of patients may not complete resource use or quality of life questionnaires. Multiple imputation (MI) is commonly used to impute the missing values by conditioning on the observed data, assuming the data are "missing at random" (MAR). However, a major concern is that the missing data are often related to the unobserved values, a mechanism also known as "missing not at random" (MNAR). For example, patients whose health is relatively poor may be less likely to complete quality of life questionnaires, even after conditioning on the observed data. Unless missing data are addressed appropriately under transparent assumptions, CEA studies may provide misleading estimates of effectiveness and cost-effectiveness, and potentially lead to wrong decisions.

**Aim**

To provide an accessible framework to perform sensitivity analyses in CEA of clinical trials with data missing not at random.

**Methods**

We first conducted a review of recently published CEA to assess the extent of missing data and approaches commonly used to address them. We also held discussions with various stakeholders (conducting or using trial-based CEA) to identify the main barriers and strategies to wider use of these methods. Based on these findings, we proposed a practical framework to conduct sensitivity analyses when data are anticipated to be MNAR. We applied this framework to the Ten Top Tips trial, which evaluates an intervention for weight management in primary care. This study illustrates a typical trial-based CEA, in which key endpoints such as self-reported QOL are likely to be MNAR.

**Results**

Our review provided further evidence that missing data was a common issue in trial-based CEA (median complete cases was 63%), and that sensitivity analyses under MNAR assumptions were rarely conducted (4%). During our discussions with stakeholders, the main barrier identified were the lack of practical guidance and software code to perform such analyses. We found that the pattern-mixture model was a desirable approach in CEA because it frames the sensitivity analysis in terms of differences between observed and missing data, which is readily understood by the different stakeholders. We illustrated how this approach can be easily implemented with standard missing data methods such as MI, and provided a framework for conducting sensitivity analyses under a broad range of assumptions. This framework also addressed the elicitation of the plausible missing data mechanisms, and the reporting of results. Application to the Ten Top Tips trial showed that results can be very sensitive to the assumptions about the missing data. For example, the intervention was likely to be cost-effective under the MAR assumption, but appear not cost-effective for some of the MNAR scenarios.

**Conclusions**

Missing data in CEA of clinical trials can result in misleading conclusions. This study proposes an accessible framework to perform CEA under a wide range of missing data assumptions, which will help future studies provide more transparent and robust evidence to inform decision-making.

### P20

#### Value of sample information as a tool in clinical trial design

Anna Heath, Gianluca Baio
University College London
**Correspondence:** Anna Heath
*Trials* 2017, **18(Suppl 1):**P20

The Expected Value of Sample Information (EVSI) quantifies the expected monetary value of a specific future trial. Theoretically, this could be an important tool for trial design for two reasons. Firstly, it would be possible to compare the monetary value of the trial directly with its cost to determine whether the trial is worthwhile. More importantly the EVSI could find the optimal trial design in terms of monetary benefit by comparing the trial value and cost for different trial designs.

Despite these useful features, the practical application of the EVSI in trial design has been restricted due to computational issues. However, recently methods been developed to overcome these computational barriers allowing researchers to use the EVSI when designing clinical trials. This will become more important as economic considerations come to the forefront of decision making for Clinical Trials. We will discuss the interpretation of the EVSI and how it can be used to aid trial design by finding economically viable designs. We will then discuss the recent computational advances for the EVSI that allow researchers to use this tool in practice to aid their decision making.

### P21

#### Development of a health informatics working group to enhance the conduct of clinical trials in primary care

Sarah Lawton[1], Simon Wathall[2]
[1]Keele Clinical Trials Unit; [2]Keele Clinical Trials Unit and NIHR Clinical Research Network: West Midlands
**Correspondence:** Sarah Lawton
*Trials* 2017, **18(Suppl 1):**P21

**Background**

Achieving and maintaining participant recruitment to clinical research, and specifically, clinical trials in primary care, is known to be challenging [1]. Experience gained from research supported by Keele Clinical Trials Unit (CTU), shows that targeted Health Informatic (HI) support early in the design phase of clinical trials may enhance the conduct of research and improve recruitment and retention rates. A collaborative approach involving Keele CTU and the NIHR Clinical Research Network: West Midlands (CRN WM) in the use of HI has been developed to embed clinical research within primary care settings. Background: Primary care infrastructure is complex and requires a number of different strategies which are innovative, efficient and transferable in order to successfully coordinate, recruit and retain both sites and participants in primary care research.

Keele CTU is a registered UKCRC CTU, specialising in the development and delivery of both feasibility and definitive multicentre randomised clinical trials, an increasing portfolio of Clinical Trials of Investigational Medicinal Products (CTIMPs) and epidemiology studies in both primary care settings and at the secondary care interface. Keele CTU has a strong HI function, with over 12 years' experience in utilising primary care clinical systems and strong links with CRN WM. CRN WM is one of 15 clinical research delivery arms of the NHS. They are responsible for ensuring the effective delivery of research within the primary care infrastructure throughout the WM area.

**Methods**

A joint HI Working Group (HIWG) between Keele CTU and CRN WM has been established to oversee, develop, support, track and quality assure the HI operational activity for research. A range of innovative methods have been developed by the working group, which can be embedded into existing GP clinical systems, to include; eligibility and recruitment searches, data collection templates, pop-ups and electronic tools to aid referrals and clinical assessments. These methods are tailored on a bespoke basis to the requirements of individual clinical research teams to perform feasibility, identification, eligibility, screening, recruitment, tagging and data collection functions and are provided together with instructions for use.

**Results**

100% of Keele CTU supported research activity involving general practices has utilised the HIWG. The groups' innovations assist to implement a robust, standardised and automated method of performing research activity in primary care settings. Greater precision of sample identification, reduced paperwork and increased efficiencies can be achieved, assisting with the retention of research participants, resulting in accessible interrogation and interpretation of research data.

**Conclusions**

Whilst there is variability in CRN resourcing nationally, the HIWG standardises the conduct of research in primary care settings, improving consistency and engagement with the primary care research infrastructure. Utilising GP clinical systems to embed research tools, results in simple, efficient and effective methods for primary care partners to conduct research. Scaling up of the HIWG over time will allow the group to provide a service for other clinical research teams conducting research in the primary care setting.

### P22

#### Use of primary care electronic records to monitor and improve intervention delivery of a GP practice level intervention

Clare Thomas[1], Rebecca Barnes[1], Helen Cramer[1], Sandra Hollinghurst[1], Sue Jackson[2], Charlie Record[3], Chris Metcalfe[1], David Kessler[1]
[1]University of Bristol; [2]University of Surrey; [3]Frome Valley Medical Centre
**Correspondence:** Clare Thomas
*Trials* 2017, **18(Suppl 1):**P22

**Background**

The routine use of electronic patient records (EPRs) in primary care provides opportunities and challenges for researchers conducting clinical trials in this setting. Although the use of EPRs to search for eligible patient populations is well established they can also be used as a resource to improve trial conduct and quality. The Footprints in Primary Care study is a feasibility study and pilot cluster randomised trial exploring the acceptability of a GP practice level intervention for frequently attending patients. Two key components of the intervention are; increased continuity of care with a named GP, and delivery of a psychosocial consultation technique called BATHE.

**Methods**
Automated searches were set up within the EPR system in the four intervention practices. These were designed to collect consultation data, such as the number and type of consultations and name of consulting GP, for patients eligible for the Footprints in Primary Care study. Information on study GP use of the BATHE technique, denoted by the GP adding a pre-specified read-code to the EPR when they had used the technique in consultations with study patients, were also collected. These automated searches were run in the practices every 6 weeks during the 12 month intervention period and anonymised data emailed to the research team. Consultations data were also collected for the same patients for the 12 months prior to the start of the study to provide a baseline comparison.
**Results**
The collection of data from EPRs at regular time points allowed the research team to monitor intervention delivery whilst the study was ongoing. This included assessment of the extent to which continuity of care had increased and the reach and dose of the BATHE consultation technique i.e. with how many patients had BATHE been used and on how many occasions. This made it possible for issues with intervention delivery, such as the low uptake of the BATHE technique amongst GPs or difficulty booking appointments with the named GP, to be followed up with study practice staff. Individualised feedback could also be provided to practices during top-up training sessions with the aim of improving intervention delivery. Furthermore the positive impact of these training sessions could be demonstrated by looking at subsequent EPR data.
**Conclusions**
Within the Footprints in Primary Care study the use of data from eprs has been important for monitoring intervention delivery, reach and dose, in providing feedback to participating practice staff, and in helping to select a maximum-variation sample of staff and patients for interview. This information, alongside qualitative interview and observational data, has been instrumental to our understanding of the feasibility and acceptability of the intervention. This approach however is not without its challenges and further consideration is needed regarding how the process of data collection and the collation of feedback would be delivered on a larger scale or in real-world implementation.

### P23
### Biospecimen management system that streamlines processes and reduces inherent challenges
Ella Zadorozny, David E. Hallam, Tamara Haller, Sharon M. Lawlor
University of Pittsburgh
**Correspondence:** Ella Zadorozny
*Trials* 2017, **18(Suppl 1):**P23

Developing procedures for biospecimen collection, processing, shipping, and storage that yield high quality research samples and data present many challenges in multi-center studies. Studies that require real-time and batch shipments from clinical sites to numerous central testing laboratories or biospecimen repositories increase the complexities required to assure integrity of the biospecimens and related data.
The data management development team in the Epidemiology Data Center (EDC), Graduate School of Public Health, at the University of Pittsburgh has designed a web-based Sample Tracking System (STS) to streamline sample tracking and shipping from point of collection to testing laboratories and repositories. The system is flexible, scalable, and can be customized easily to meet the needs of individual studies.
Modules included in the STS are: entry and editing via barcode scanner or keyboard, generation of shipping manifests, and receipt confirmation at the batch and sample level, with database audit trails for all modules. Automated email notifications alert laboratory/repository personnel of incoming shipments and clinical site personnel of shipments received.
The STS can be implemented as a stand-alone system or integrated with a data management system. It is efficient in regard to database setup and implementation and is user-friendly and intuitive for site and laboratory/repository personnel, facilitating smooth study startup. It was designed to accommodate unlimited clinical sites, laboratory/repository destinations, sample types, and samples/aliquots with minimal setup time or expertise on the part of EDC data management personnel. Data management personnel use administrative tools to define study name, site codes, sample types, sample names/titles, sample states (e.g. Frozen, ambient), barcode formats, laboratory/repository names, and protocol timepoints, and to define the relationships among samples, studies, sites, and laboratories/repositories. Optional settings are provided for default volume, volume/unit (ml, µg, slide, image), minimum and maximum volume/unit, and earliest sample date. There are options to initialize barcodes in the database and then utilize initialized barcodes to provide validations (e.g. Site, participant ID, sample type, timepoint) at the time of sample entry. At the time of receipt of shipments, the system allows receiving personnel to resolve issues and input comments at the batch or sample level.
The STS is in use on several EDC projects and has facilitated biospecimen-related processes, reduced data management effort for system setup, maintenance, and monitoring, streamlined site and laboratory/repository sample-related processes, and has improved real-time validations and the quality of sample-related data.

### P24
### A targeted approach to drug-supply in RCTs limits wastage and can reduce costs. The experience of the MS-smart trial
Allan Walker, Moira Ross
University Of Edinburgh Clinical Trials Unit
**Correspondence:** Allan Walker
*Trials* 2017, **18(Suppl 1):**P24

The MS-Smart trial is a four-arm phase IIB randomised, double-blind placebo controlled clinical trial comparing the efficacy of neuroprotective drugs in secondary progressive multiple sclerosis.
Treatment allocation is by minimisation without a site stratification element. Participant follow up is over two years and each participant has at least 6 post-randomisation clinic visits where trial drugs are provided.
The cost of the trial drugs is significant so all reasonable steps should be taken to limit oversupply at site leading to drug wastage.
Sending equal amounts of each of the four drugs to site pharmacies leads to wastage as the treatment allocation method does not guarantee a balance of allocated treatments among recruits at each site. In addition, site pharmacies often have limited storage space and find it difficult to accommodate deliveries of large volumes of trial drugs.
We propose that a more targeted approach to drug re-supply will address these issues by both reducing the volumes of drugs delivered to sites and at the same time reducing the amount of drug wastage. Utilising the central trial database allows us to identify exactly which post-randomisation visits are upcoming at each site and to assign deliveries to sites based upon this. So, if a site had a run of equal treatment allocations then our supply algorithm will dictate that drug supplies at this site six months later be weighted accordingly rather than issuing equal amounts of each drug to the site. Using this mechanism will help planners more easily determine how many drugs will be needed for a trial and allow them to reduce the amount of contingency required and hence reduce the costs of running a drug trial.

### P25
### MS sharepoint - using collaborative software to support collaborative research
Claire Kerr, Mairi Warren
University of Glasgow
**Correspondence:** Claire Kerr
*Trials* 2017, **18(Suppl 1):**P25

**Background**
The Robertson Centre for Biostatistics conducts and supports collaborative research in clinical trials through the design, conduct, analysis and interpretation of clinical trials and other well conducted studies. The Centre's staff consists of biostatisticians, database managers, software developers, technicians, health informaticians, health economics, project managers and administrative staff contributing to some 120 clinical studies at present. Involvement in this volume of clinical studies has led the Centre to identify a software solution to more effectively project manage our involvement in these studies whilst supporting the requirements of the Centre's internal Standard Operating Procedures (sops) and Good Clinical Practice (GCP).

**Methods**

Over the past 6 years the Centre, in consultation with staff, has customised and developed an MS sharepoint site to manage key project information and activities relating to clinical studies including: Project planning and management; Change management; Document control; Study communication; Management reporting

The MS sharepoint site has been further developed to support: Functional areas; Archival; Audit Management; Centre Communication; Risk Management

**Conclusion**

MS sharepoint has been a key tool in providing a consistent approach to managing projects however, it has been recognised that the system should continue to evolve in order to meet changing regulatory and Centre requirements.

The Centre continues to identify other areas where MS sharepoint could be used to aid process and quality improvement.

### P26

#### Automated solution for tracking electronic case report form completion

Elizabeth Hill, Joanna Illambas, Eddie Heath, Charlotte Friend, Hassan Nawrozzadeh, Emma Hall, Claire Snowdon, Judith Bliss, Rebecca Lewis
The Institute of Cancer Research
**Correspondence:** Elizabeth Hill
*Trials* 2017, **18(Suppl 1):**P26

**Background**

The ICR-CTSU introduced electronic data capture (EDC) in 2012. This necessitated development of a solution to automatically monitor electronic case report form (ECRF) completion and track timely completion of ECRFs.

**Challenges**

Prior to the introduction of EDC, sites posted paper CRFs to the ICR-CTSU. Once received, CRFs were manually tracked onto an ICR-CTSU legacy system which also provided CRF compliance reports. With the introduction of EDC, a solution was required to record real-time completion of ECRFs within the EDC system and to calculate ECRF compliance data for review and reporting purposes.

**Solution**

A two part solution was developed:

1. Schedule forms were created within the EDC clinical database. These forms display details of ECRF expected and completed dates per trial participant for every visit and form (dependent on the participant's treatment allocation and pathway within the trial). The expected date of each ECRF can be calculated from any date field captured within the EDC system and is tailored as needed depending on requirements for each individual ECRF. The ECRF completed date uses a standard ECRF field "date form submitted". As forms are completed by site staff, ECRF completion progress can be viewed in real-time on the schedule forms.

2. An in-house C#.net Windows application was developed for use by ICR-CTSU to read the schedule form data from the EDC system and calculate ECRF compliance data as required. Compliance data can be provided per trial to produce outstanding ECRF reports for provision to site and to review ECRF response rates by form and participating site.

**Conclusion**

This solution provides a real-time automatic ECRF tracking system that allows central review of ECRF compliance data as required. The user-friendly schedule forms within the EDC clinical database also assist trial staff at sites with monitoring expected ECRF completion time points for individual trial subjects.

### P27

#### Ascertaining study participant safety using centralized electronic medical records in a clinical trial setting — lessons learned from the veterans affairs NEPHRON-D trial

Yuan Huang, Gary Johnson, Tassos Kyriakides, Jane H. Zhang
CSPCC, VA Connecticut Healthcare System West Haven; Yale University
**Correspondence:** Yuan Huang
*Trials* 2017, **18(Suppl 1):**P27

**Background**

Electronic medical records (EMRs) are now frequently used for collecting patient-level data for clinical trials. With the Veterans Affairs (VA) Healthcare System, EMR data have been widely used in clinical trials to assess eligibility and facilitate referrals for recruitment, and to conduct follow-up and safety monitoring. More recently, the EMR is being used for point-of-care randomization trials and for conducting trials from central location. Despite the great potential efficiency of using the EMR, it is of interest and importance to evaluate the integrity of data captured from the EMR through a centralized monitoring algorithm without involvement of research personnel compared to that collected by local investigators or coordinators under protocol conditions. This investigation assesses the verification of safety data collection.

**Design**

The VA NEPHRON-D study was a multi-center, double blind, randomized clinical trial to assess the effect of ACEI and ARB combined vs. ARB alone on the progression of kidney disease in individuals with diabetes and proteinuria. The safety endpoints of the trial included serious adverse events (SAE), acute kidney injury (AKI), hyperkalemia and mortality. A subset of the participants (~62%) who enrolled in a long-term follow-up substudy were consented for data collection via the EMR. For those participants with consent, data accumulated in their medical records during the study period were extracted from the VA Corporate Data Warehouse (CDW). We accessed the CDW centrally, captured the safety data and compared these records with those collected by the study personnel at VA Medical Centers participating in the VA NEPHRON-D trial. This assessment examines both general and study-specific safety endpoints, and more importantly, provides evidence for how to use extracted EMR data for documenting SAE and study outcomes in futures studies.

**Result**

Hospital admission data were obtained from CDW's acute care, extended care, and observational care records. Study-collected SAEs were consolidated into a single hospital stay for comparison with EMR records. A high level of matching was found using the CDW to verify SAE reported during the active trial for hospital admissions within the VA healthcare system. Hospitalization records that were stored as scanned notes from non-VA admissions were not included as CDW records, which is an issue that still needs to be addressed for obtaining a more complete data collection. Also, identifying individual SAEs during the same hospitalization stay requires further investigation. AKI was a major safety endpoint in the study. Different definitions of AKI based on ICD-9 codes and change of creatinine during hospitalization were applied in the CDW data searches. The search results varied significantly depending on the AKI definition applied. Likewise, hyperkalemia identified by the CDW laboratory datasets had some discrepancies from the active trial setting where diagnosis of hyperkalemia was a combination of potassium level and other clinical factors. Details of the comparisons for each safety endpoint will be presented.

**Conclusion**

This investigation identifies several factors that affect the quality of EMR-mediated safety data collection compared to active study conditions and establishes the importance of an additional level of clinical review of EMR data.

### P28

#### Producing CDISC compliant data and metadata for regulatory submissions

William Stevens, Karl Wallendszus, Martin Landray
University of Oxford
**Correspondence:** William Stevens
*Trials* 2017, **18(Suppl 1):**P28

**Background**

The purpose of the data-related components of an FDA regulatory submission is to enable an FDA reviewer to understand the clinical trial data that was collected, check the consistency of the data, understand how analysis datasets were produced, and recreate selected analyses.

Our experience is based on using the Clinical Data Interchange Standards Consortium (CDISC) standards (http://www.cdisc.org/) for preparing regulatory submissions for 3 trials totalling 65,000 randomized participants. Study Data Tabulation Model (SDTM) datasets represent trial data in a standardised form. Analysis Data Model (ADAM) datasets are derived from SDTM datasets, and represent data in a form that is easy to analyse and report on. 'define.xml' documents contain metadata for SDTM and ADAM datasets. Brief guidance notes accompany the datasets, explaining anything that cannot be understood using the metadata.

**Steps**

The main tasks involved in producing these items are: – Assess how collected data maps to SDTM datasets and outline this in annotated case report forms (CRFs). - Decide which ADAM datasets are needed for analysis, based on the Protocol and Data Analysis Plan. - Transform SDTM data into relevant ADAM datasets. - Generate 'define.xml' metadata documents. - Validate all datasets and metadata, correcting or documenting errors. - Produce guidance notes for the SDTM and ADAM datasets.

We use bespoke software tools for these steps (except validation, which is performed using industry standard software).

**Software**

SDTM and ADAM datasets are stored in a relational database. Datasets are defined and produced using a domain-specific language that permits XML elements to be associated with parameterized units of software which generally perform SQL code generation (which can be executed to perform a data transformation), but which may also do other things, such as the generation of documents. Some examples are: – Conversion of units for a defined set of lab results, while checking that there are no unexpected combinations of lab test and units. - Estimating dates from partial dates and upper and lower limits. - Generating CDISC define.xml documents.

The core language has a small codebase (approx. 2000 lines of code) and few non-standard dependencies. Most of the functionality of the system is expressed in well-documented parameterized units (approx. 4000 lines of code). An automated test suite (approx. 4000 lines of code) verifies the functionality of each unit.

**Conclusion**

Bespoke, modular and light-weight tools were useful during the development of our process for generating regulatory submission packages because these tools are rapidly adaptable. The automated test suite helps prevent changes from having unanticipated consequences. From the perspective of programming and data modelling, the CDISC standards have some limitations which could be readily addressed in future versions of the standards.

### P29

#### How do you detect and deal with compromised EDC accounts?

William Aitchison, Sharon Kean, Jonathan Gibb
Robertson Centre for Biostatistics
**Correspondence:** William Aitchison
*Trials* 2017, **18(Suppl 1):**P29

**Objective**

The objective is to devise solution approaches given the scenario where, despite all best security practises being employed there exists the possibility that malicious parties could still gain access to some element of the system architecture, how can systems be designed to detect malicious activity by legitimate but compromised user, application or system accounts? Furthermore the question - when malicious activity is detected, what automated and external processes should occur must be explored.

**Background**

There is numerous security measures that can be employed to safeguard online systems, however due to the complex layered architecture of today's applications there are various potential weak points. While following best practices should reduce the risk of malicious parties gaining access to systems, often there are financial or bureaucratic obstacles to following best practices. Keeping all software and hardware components maintained with current patches represents a considerable amount of work and cost. Despite all this effort there is always the potential of previously unknown zero day exploits being discovered, new strains of malware being created and a dizzying array of new ways to trick computer users into disclosing their credentials or granting access to third parties. An intrusion detection system (IDS) monitors a network or systems for malicious activity or policy violations. The use of an IDS, or combination of different IDS systems are generally considered best practice. There are very diverse approaches to IDS implementation ranging from configurable rule based systems to machine learning adaptive systems therefore it can be advantageous to employ more than one IDS. An IDS is an important security tool however they are of limited use if a malicious party compromises a system account and performs similar actions e.g. Accessing the trial database. Worse still an IDS is entirely blind to application level activity as most web applications utilise a single system account to perform all actions.

**Method**

We propose integrating simple IDS methods into the application and database layers. By identifying simple activity rules to identify unusual usage the application and database can react in an appropriate manner based on the associated level of risk.

**Conclusion**

The authors will present an overview of IDS style methods suitable to clinical EDC systems, how to implement them and how to structure a framework for responding to them.

### P30

#### A review of the essentials and pitfalls of the Lugano classification in malignant lymphoma trials

Dewen Yang, David Raunig
ICON Clinical Research
**Correspondence:** Dewen Yang
*Trials* 2017, **18(Suppl 1):**P30

To facilitate the comparison of patients and results by providing a standardized guidance on how data should be analyzed for therapy, response criteria for non-Hodgkin lymphoma (NHL) were published in 1999 by an international working group (IWG). The revision for both NHL and Hodgkin lymphoma (HL) was published in 2007 to incorporate PET and bone marrow biopsy in response assessment. After years of experience with the 2007 criteria and recognizing the imaging technique progress, the 2nd revision called the Lugano classification was published in 2014 to assess lymphoma therapeutic response in clinical trials. The Lugano guidelines have enhanced interpretation of CT assessment, imaging schedules, and PET scoring implications, rules for handling missing anatomy and challenging scenarios for the given therapeutic under investigation. The Lugano classification provides a renewed opportunity to guide lymphoma diagnosis and clinical management based on imaging findings. The new criteria also have been increasingly adopted in many lymphoma trials since its publication. Nevertheless, certain aspects of the new criteria lack sufficient detail for explicit interpretation and a few features open to potential pitfalls which need particular attention and further discussion. For instance, the five-point scale (5-PS) for FDG-PET assessment was incorporated to evaluate tumor metabolic response assessment in FDG-avid lymphoma types, but the 5-PS, copied from Deauville criteria, relies on a vague description of qualitatively assessing “Change of the hottest lesion” And no definitive guidance on “Significant change in FDG uptake”; moderately/markedly higher than liver or whether quantitative uptake measurements are allowed as the cut-off reference for the score 4 or 5; beside of the imaging scan window, imaging findings on CT and FDG PET-CT can be rarely conflicting. In addition, progressive disease with regard to splenomegaly assesses response with regard to both the baseline and to ‘prior increase’ which, if interpreted one way, can lead to extreme enlargement without progression. On the other hand, splenomegaly can be caused by lymphoma-unrelated causes such as portal hypertension or use of hematoietic growth factors which make a question if splenomegaly alone can be used to define the progressive disease. To provide the most accurate assessment of response to therapeutic intervention, it is essential that trial oncologists and radiologists not only have a tangible understanding of the Lugano Classification, but also proper insight into the practical limitations of the criteria. We will review the essential elements and provide few examples to illustrate the limitations and ambiguity that can arise from different interpretations of the Lugano classification. Furthermore, some suggestions will be made to stimulate further improvement in clinical trial settings.

### P31

#### Will you walk a little faster? - joining the database development dance

Mary Rauchenberger[1], Kenneth Babigumira[2], Chiara Borg[2], Emma Little[2], Nancy Tappenden[2], Matthew R. Sydes[2], Nadine Van Looy[2]
[1]MRC Clinical Trials Unit at UCL, Institute of Clinical Trials and Methodology, UCL, London, UK; [2]MRC London Hub for Trials Methodology Research, London, UK
**Correspondence:** Mary Rauchenberger
*Trials* 2017, **18(Suppl 1):**P31

**Problem**

Trialists often feel that the release of a validated database is a limiting factor in the timeline of opening a clinical trial. There is an inherent tension between (i) the desire to be able to change requirements (such as Case Report Forms, eligibility and validation checks) as late as possible and (ii) the need for those requirements to be finalised early on so that development and testing can take place. We will describe several approaches we have taken to address this dilemma. These focus initially on technical solutions, using our bespoke clinical data management system, developed using MS SQL Server and.NET. We will then expand to look at how these can be enhanced with process changes. This has led to a culture change resulting in much wider participation in the database development project, a livelier dance with more partners on the dancefloor.

**Our approach**

The technical solution concentrates on auto-generation. In our model, Excel is used to document the user requirements as metadata. This allows users to engage with a familiar tool to specify conditions in a structured method. Once reviewed and finalised, the spreadsheet is uploaded into the database and the metadata is used to generate the database tables, triggers and procedures that provide the necessary functionality (such as audit trail, query generation, etc.). The metadata also provides the input to a customised code generator which produces the front-end code for data entry screens and validations. Common code modules and standard field names produce a consistent interface, with core functionality and generic elements that can be easily reused across projects. The tempo of the dance for the database developers moves from a waltz to a quickstep. We have also looked at development methodology, moving away from waterfall approaches and adopting elements of agile project management and development into our processes. Key to this is the phased approach, concentrating on what needs to be included in the initial release and keeping to firm release dates, prioritising the product backlog for each release. Self-organising teams, feasible in larger trials units, bring more resource to a project at critical timepoints. Workshops with the trial team help with metadata development and encourage ownership. Rapid coding approaches, using group sessions and peer review, and group testing sessions, implementing fixes in real-time, have also been implemented to accelerate progress. The more agile dance is now perhaps a casual group samba, involving developers, data managers, data scientists, and business analysts.

**Discussion**

Ultimately though, there is a limit to how fast you can dance. The culture change needed requires much earlier penetration into the trial project timeline, looking at team resourcing and key decision timepoints. Involving the database team at the earliest stages helps with understanding how the proposed trial flow can best be implemented, and with prioritisation of agreements needed for timely delivery of the validated database. The dance becomes a unit-wide quadrille, with multiple partners and movements. Or, maybe more appropriately for this conference, a ceilidh!

### P32

#### Presentation and publication system (PNP): a tool to facilitate efficient tracking and reporting of the presentation and publication process

Tamara Haller, David E. Hallam, Sharon M. Lawlor, Ella Zadorozny
University of Pittsburgh
**Correspondence:** Tamara Haller
*Trials* 2017, **18(Suppl 1):**P32

Writing of a manuscript, abstract, or other document in a research environment is a collaborative effort which oftentimes involves individuals from academia, industry, and government agencies. Topics are proposed and must be managed according to study timelines and may require considerable time and resources to track over the course of a study. The data management development team in the Epidemiology Data Center (EDC), Graduate School of Public Health, at the University of Pittsburgh has designed a web-based Presentations and Publications System (PNP) to streamline work flow, provide a repository for completed works, and facilitate tracking and reporting of the presentation and publication process.

The system is comprised of a Pre-proposal module, which allows users to quickly enter potential topics, and a Proposal module, which begins when a more fully developed topic is submitted.

The Pre-Proposal module facilitates sharing topic ideas and allows topics to be ranked and prioritized. Potential collaborators use this module to indicate an interest in participating in a writing group. Approved topics are moved into the Proposal module for development into an abstract, manuscript or preliminary analysis proposal.

The Proposal module is used to submit a more detailed description of the topic, set priorities for the proposal, and track and manage the activities and content. The main page of the Proposal module provides access to all abstracts, manuscripts, and grant proposals submitted for the project and contains key information such as the status of the proposal and the latest activity. Proposals are managed via a tracking page, which has tabs for the submitted proposal form, summary information (e.g. Stage and status of the proposal), detailed proposal tracking activities, and a reference library. Proposal activities are grouped into pre-defined categories (e.g. Writing group, reviewers, scientific meetings, and journals); these categories are also available as individual tabs to allow reporting all manuscripts in a particular category.

The PNP system was designed to allow the user to quickly configure the system based on the study’s requirements through a set-up wizard. The wizard does not require that all elements be pre-defined, but allows the users to configure the system throughout the process. Reports created in Crystal Reports or SAS are supported by the PNP system and allow the users to create customized reports at the project and proposal level. Access to the PNP system is restricted according to a user’s project role and permissions assigned.

In summary, the PNP system is a tool that can help to organize the process of writing a manuscript, abstract, or research grant while reducing the personnel time and effort needed for communication and coordination among the collaborators.

### P33

#### More than the emperor's new clothes: enhancing meaningful patient and public involvement in trial oversight committees through qualitative research with eight clinical trials facing challenges

Alex Nicolson[1], Anne Daykin[1], Karen Coulman[1], Clare Clement[1], Helen Cramer[1], Carrol Gamble[1], Rhiannon Macefield[1], Sharon McCann[1], Gillian W. Shorter[2], Mathew R. Sydes[3]
[1]University of Bristol; [2]Ulster University; [3]Institute of Clinical Trials and Methodology
**Correspondence:** Alex Nicolson
*Trials* 2017, **18(Suppl 1):**P33

**Background**
The value of Patient and Public Involvement (PPI) in trial oversight is increasingly recognised; at present it is a requirement for most UK research funding bodies to involve PPI members in Trial Oversight Committees (TOCs) including Trial Steering Committees (TSCs) and Trial Management Groups (TMGs). However, there is little evidence-based guidance to optimise their roles and inputs. The actions and experiences of TOC members including PPI representatives were captured to inform recommendations about enhancing PPI contribution to trial oversight. This was carried out within the context of a larger multi-method study which aimed to explore the role and function of TOCs, and their contribution to trial conduct.
**Methods**
TOC meetings of eight large phase III UK trials that were undergoing challenges (e.g. Recruitment issues, protocol deviation or amendments) were observed by a qualitative researcher and audio-recorded. Interviews explored PPI in interviewees' trials and where they thought PPI contributors were best placed. PPI representatives also reflected on their personal experience of TOC meetings, their understanding of their roles and how they felt they had influenced trial conduct. Data (meeting transcripts, field notes and interview transcripts) was analysed thematically using techniques of constant comparison.
**Results**
Seven TSC and six TMGs (n = 13) were observed and six of the meetings had PPI present (3 TSC, 3 TMG). Sixty-six semi-structured interviews were carried out with fifty-two members of these TOCs which included three PPI representatives.
Analysis revealed the importance interviewees placed on the role of PPI to provide a patient voice within trial oversight. PPI was generally favoured within TOCs, but several tensions arose relating to meaningful PPI implementation at TSC and TMG levels. Lack of clarity about what PPI is and whether it was needed led to inclusion of those representatives who, perhaps, were not best equipped with the appropriate skills, experience and attributes. Representatives who lacked detailed knowledge or familiarity with trial methodology and technical language found it difficult to understand and contribute to meetings. Interviewees felt it was important when selecting representatives to consider whether they truly had empathy for the trial population or had possible 'hidden agendas'. Consideration of PPI representatives' commitments and circumstance outside of trial oversight was important for ongoing engagement and attendance. Participants saw a need for training and or mentoring of PPI representatives to foster appropriate involvement and contribution. However, there was no clear consensus of who was or should be responsible for enabling or providing such training and support.
**Conclusion**
To truly enable PPI representatives to speak on behalf of patient or public voice and ensure meaningful contributions of such representatives within trial oversight, more thought needs to be given to designing the involvement of PPI in TOCs. This includes clarification around roles and what would constitute optimal involvement at different oversight levels and stages of trials. To ensure ongoing worthwhile PPI, training and support needs of contributors needs to be reflected upon and provided, and consideration needs to be given to PPI selection and TOC meeting conduct to ensure attendance and engagement is maintained.

### P34

#### NIHR research design service

Mark Mullee
University of Southampton
*Trials* 2017, **18(Suppl 1):**P34

**Background**
The NIHR (National Institute for Health Research) is the research arm of the NHS and is the most integrated clinical research system in the world. It invests about one percent of the NHSD budget in research to improve the health and wealth of the nation.
The NIHR funds the RDS (Research Design Service) to provide design and methodological support to health and social care researchers across England to develop grant applications to the NIHR (Programme Grants for Applied Research, Research for Patient Benefit, Health Technology Assessment, Public Health Research, Invention for Innovations, Health Services and Delivery Research etc.) And other national peer-reviewed funding programmes.
The RDS is a national service delivered by ten regions covering England.
**NIHR RDS (research design service) expertise**
Methodological advice is provided to researchers by teams of Advisers whose expertise includes statistics, qualitative research methods, health economics, systematic reviews, health psychology and behavioural science.
The RDS has an important role in referring individuals to appropriate sources of advice, outside of the RDS, where appropriate. For example, referrals to those with specialist expertise in intellectual property or to a local Clinical Research Network for practical help in identifying and recruiting patients to studies.
**Public involvement in research**
The RDS has been at the forefront of the NIHR drive to ensure that members of the public play an important role in developing successful grant applications. The RDS has been particularly active and pioneering in the area of Public Involvement, from design of the research study through to dissemination of research findings.
The RDS recently worked in partnership with the Wessex Institute, University of Southampton on a successful bid to host INVOLVE (funded by NIHR to support active public involvement in research). The expertise and regional networks of the RDS were recognised as an important component of the partnership.
**NIHR RDS metrics**
The RDS remit includes increasing the quality and quantity of research applications. Since 2009, the RDS has supported:
17,949 projects, 2,705 outline applications submitted with 1,111 shortlisted (43% success rate), 6,432 full applications submitted with 2,209 funded (36% success rate).
The RDS also provides triage for under-prepared or misplaced funding applications. Thus, reducing waste in terms of the time and resource used by NIHR funding programmes to review poor quality applications.
**One NIHR**
The RDS is recognised as the 'local face of the NIHR'. It has become an intermediary between national NIHR structures (Collaboration for Leadership in Applied Health Research and Care (CLAHRC), Clinical Research Networks, Clinical Trials Units, Biomedical Research Centres and NHS Trust R&D etc.) and local investigators and organisations.
The RDS has often facilitated local partnerships, to pursue 'One NIHR'. It has brought together various components of the NIHR at local and regional levels, to share good practice, look for efficiencies of delivery and to enable investigators and organisations to have a more streamlined access to support and advice.

### P35

#### A program for training the next generation of biostatisticians in japan: developing on-the-job training at NCVC

Toshimitsu Hamasaki[1], Haruko Yamamoto[1], Shiro Tanaka[2], T. Shun Sato[2]
[1]National Cerebral and Cardiovascular Center; [2]Kyoto University School of Public Health
**Correspondence:** Toshimitsu Hamasaki
*Trials* 2017, **18(Suppl 1):**P35

Statistical contributions to clinical trials and medical product development have been well-recognized in Japan since the ICH-E9 guideline "Statistical Principles for Clinical Trials" Was implemented in 1998, where the guideline helped trigger the revelation that there is a shortage of qualified statisticians who can comprehend and implement the principles outlined in the guideline and improve the quality and integrity of the trials being conducted. Although the number of educational programs for Master and phd level biostatisticians at universities have been greatly increased during the last two decades, at this period, the supply of new graduates in biostatistics in Japan is relatively steady while the demand is increased dramatically.
Different level of efforts including government, society, university, and industry have been devoted to increasing the number of "qualified" biostatisticians in Japan, and in October 2016, Japan Agency for Medical Research and Development (AMED) have decided to fund the two universities, Kyoto University (KU) and University of Tokyo to develop a new program for training the next generation biostatisticians with emphasis in clinical trials, under the public and private partnership with the Japan Pharmaceutical Manufacturers Association (JPMA). Each of two universities formed the alliance to develop the program: KU with KU Hospital and National Cerebral and Cardiovascular Center (NCVC), and UT with UT Hospital and National Cancer Center.
In this presentation, we briefly review the current issues in MPH-level biostatistical education and training in Japan, and outline our plan and activities for developing the educational and training program for the next generation biostatisticians. Our developed program is very unique to combine the two learning approach to gain skill and knowledge of clinical trials-related biostatistics: learnings (i) by being taught, by studying it, or by researching it through structured courses/modules at KU School of Public Health and (ii) by experiencing it in practical situations (i.e., On-the-Job (OJT) Training) at KU Hospital or NCVC. We describe our developed OJT training program at NCVC.

### P36

#### Efficient group-sequential designs for monitoring two time-to-event outcomes in clinical trials

Toshimitsu Hamasaki[1], Koko Asakura[1], Tomoyuki Sugimoto[2], Scott R. Evans[3], Haruko Yamamoto[1], Chin-Fu Hsiao[4]
[1]National Cerebral and Cardiovascular Center; [2]Kagoshima University; [3]Harvard T.H. Chan School of Public Health; [4]National Health Research Institutes
**Correspondence:** Toshimitsu Hamasaki
*Trials* 2017, **18(Suppl 1):**P36

We discuss logrank test-based methods for early efficacy or futility evaluation in group-sequential clinical trials designed to compare two interventions using two time-to-event outcomes. We consider three typical situations (1) both events are non-composite and non-fatal, (2) both events are non-composite but one event is fatal, and (3) one event is composite but other is fatal and non-composite. We outline strategies for rejecting the null hypothesis associated with two inferential goals, evaluating if a test intervention is superior to a control intervention on: (1) both outcomes (multiple co-primary endpoints: MCPE), and (2) at least one outcome (multiple primary endpoints: MPE). We provide an example to illustrate the methods and discuss practical considerations when designing these trials.

### P37

#### Clinical and psychometric validation of a new outcome measure: methods to assess measurement properties in the absence of a 'gold' standard

Rhiannon Macefield, on behalf of the Bluebelle Study Group
University of Bristol
**Correspondence:** Rhiannon Macefield
*Trials* 2017, **18(Suppl 1):**P37

**Background**

Patients' health outcomes and experiences are often measured using validated questionnaires. Responses are usually scored and values over a certain threshold can be interpreted as clinically meaningful or "problematic". Standard methods to identify such thresholds require an established reference standard and the use of receiver operating characteristic (ROC) curves. We have developed a new questionnaire to assess wounds for surgical site infection (SSI), with a view to it being used as an outcome measure in a future trial. Validation, however, is challenging because the diagnostic accuracy of the established reference standard is imperfect and estimates of sensitivity and specificity may therefore be biased. The aim of this study is to explore the clinical validity and measurement properties of the new measure in the absence of a "gold" standard.

**Methods**

A 16-item questionnaire assessing signs, symptoms and interventions potentially indicative of SSI was developed using standard methods. Patients undergoing general abdominal surgery and women undergoing caesarean section were recruited from three UK hospital trusts. Participants were sent the new questionnaire to complete approximately 30 days after surgery and return by post (self-assessment). Short "debriefing" questions to assess ease of completion were included. Healthcare professionals attempted to contact participants approximately 30–35 days after surgery and complete the new questionnaire via telephone (observer assessment). A proportion of participants (limited by study resources) were seen face-to-face 4–8 weeks after surgery and classified as having an SSI or not using the Center for Disease Control (CDC) classifications for wound infection (reference standard). These assessors were blinded to participants' self-assessment and observer assessment. Analyses, which are ongoing, will: 1) compare participant (self-assessment) and healthcare professional (observer assessment) responses, 2) examine the sensitivity of the questionnaire for identifying symptoms compared to similar criteria in the reference standard, 3) test a clinician-lead hypothesised scale structure and scoring system for determining SSI outcome, 4) examine the discriminative ability of the questionnaire to identify potential SSI "problems" Using a set of receiver operating characteristic (ROC) curves and 5) assess the reliability of the questionnaire.

**Results**

416 participants were recruited. Participants completed and returned 300/414 (72.5%) questionnaires (self-assessments). Healthcare professionals successfully contacted 306/414 (73.9%) participants and completed questionnaires via telephone (observer assessments). Face to face assessments were made for 115 (27.7%) participants (reference standard). Participants found the questionnaire quick and straightforward to complete, with few missing data. Initial analyses of participant and healthcare professional responses show that symptoms are reported a little more severe in self-assessments compared to observer assessments; a consistent trend observed for all eight symptom-related items. Other planned analyses are ongoing, pending additional data from a pilot RCT where all participants (n = 330) were scheduled to receive a reference standard assessment.

**Conclusion**

Examination of the clinical validity and measurement properties of a new SSI outcome measure is ongoing. Different thresholds for SSI "problem" scores may be needed when assessments are made by participants or healthcare professionals. Qualitative work to further understand the difference in agreement between participant and healthcare professional reports of symptoms would be beneficial.

### P38

#### Assessing the impact of a funder's recommendation on consideration and uptake of core outcome sets in funding applications

Karen Barnes[1], Jamie J. Kirkham[1], Mike Clarke[2], Paula R. Williamson[1]
[1]University of Liverpool; [2]Queen's University Belfast
**Correspondence:** Karen Barnes
*Trials* 2017, **18(Suppl 1):**P38

**Background**

A systematic review published in 2014 [1] identified 198 published core outcome sets (COS) and a recent update found that this figure had increased to 227 by the end of that year [2]. The details of these

COS, along with others that are planned and in development, are recorded in the COMET (Core Outcome Measures in Effectiveness Trials) database. As the number of COS grows, it is important to assess their uptake by clinical trialists because the continued development of COS, without their implementation, could add to waste in research, and would mean that those using the results of trials to make decisions about healthcare will not realise the benefits that using COS can provide.

In January 2012 the guidance for NIHR HTA funding recommended 'details should include justification of the use of outcome measures where a legitimate choice exists between alternatives. Where established Core Outcomes exist they should be included amongst the list of outcomes unless there is good reason to do otherwise. Please see The COMET Initiative website at www.comet-initiative.org to identify whether Core Outcomes have been established.' This study will assess the extent to which this recommendation has been followed by NIHR HTA applicants from January 2012, when the recommendation was introduced, to December 2015.

**Method**

The completed application form and detailed project description of each NIHR HTA application will be examined for:

Evidence that the COMET database had been searched to establish whether or not a COS exists
Reference to a COS study published in the COMET database
Evidence that a COS was included in the application if one exists
Evidence that a COS was not included in the application where one exists
Reasons given for not including a COS where one exists
Rationale for outcome choice in the absence of a COS

**Analysis**

Following extraction of the above data, the following analysis will be performed:

- Assessment of the number and proportion of NIHR HTA applications referencing the COMET database or a COS published in the COMET database
- Assessment of the number and proportion of NIHR HTA applications using a COS, if one exists, in their research

These assessments will be used to draw conclusions about the potential impact on the use of COS of a research funder's recommendation about their use.

**Results and Conclusions**

Results and conclusions will be presented following examination of all funded and non-funded applications to the NIHR HTA researcher-led, commissioned and themed call funding streams from January 2012 to December 2015 ($n = 281$). The sample consists of applications for both randomised trials ($n = 189$) and evidence syntheses ($n = 92$).

### P39

#### Variability in composite outcomes reported in cardiac surgery studies: a literature review

Rachel Maishman[1], Barnaby C. Reeves[1], Umberto Benedetto[2], Chris A. Rogers[1]
[1]University of Bristol Clinical Trials and Evaluation Unit; [2]Bristol Heart Institute, University of Bristol
**Correspondence:** Rachel Maishman
*Trials* 2017, **18(Suppl 1):**P39

**Background**

Composite outcomes are often reported in randomised controlled trials, particularly for safety endpoints. Use of a composite endpoint can allow a study to provide information about safety when the rates of component adverse events are low, but risks aggregating events that are not affected by the intervention. We undertook a literature review to explore the variability in composite outcomes used in cardiac surgery studies, to inform the development of an objective measure of recovery.

**Methods and results**

All published articles reporting at least one short-term composite outcome assessed within three months of cardiac surgery were identified. One hundred and fifty four papers were identified, reporting 166 composite outcomes; 64 different adverse events were included across the composite outcomes. Death was a component in the majority of composites (135/166, 81%), as were cerebrovascular events (105/166, 63%), myocardial infarction (MI) (81/166, 49%), renal failure/acute kidney injury (AKI) (78/166, 47%) and reoperation/revascularisation (42/166, 25%). Two "established" composite outcomes were identified in the review, Major Adverse Cardiac Events (MACE) and Major Adverse Cardiac and Cerebrovascular Events (MACCE), but the definitions for both differed across studies. Assuming MACCE includes death, cerebrovascular events, MI and reoperation/revascularisation, 16/166 composites included these four components; 12 of these 16 also included other adverse events, suggesting that the currently used composite outcomes are based on, but not restricted to, existing MACCE definitions. Other adverse events that were commonly included together in composite outcomes were renal failure and death/cerebrovascular event, and prolonged ventilation and death/cerebrovascular event.

The majority of composite outcomes were binary outcomes (any event vs. none) that gave equal importance to all components. Two studies investigated the relative weighting assigned to adverse events in MACCE, both among patients and one among trialists, and reported that respondents assigned different weightings to each of the adverse events within the composite. Differences between the weightings assigned by patients and clinical trialists were also reported, with patients rating MI and stroke the same as or worse than death, but trialists rating death as the most severe.

**Discussion**

This review has highlighted the variability in the way composite outcomes for cardiac surgery studies have been defined. The range of events included supports the need for the development of a composite outcome including a range of adverse events to give a more complete picture of recovery. Furthermore, these findings support the need for composite outcomes to incorporate weightings, particularly when adverse events differ in their impact on patient recovery, and for the views of both patients and clinicians to be considered when assessing the relative importance of different adverse events if the composite outcome is intended to give an overall assessment of recovery. Variation was seen in the definitions used for some events (e.g. renal failure) across studies; there is a need for consistent definitions to be agreed to aid synthesis of results from different cardiac surgery studies in meta-analyses.

### P40

#### Evaluation of interventions for informed consent for randomised controlled trials (ELICIT): results from a systematic review and interviews towards developing a core outcome set

Katie Gillies[1], Heidi Gardner[1], Alex Duthie[1], Cynthia Fraser[1], Vikki Entwistle[1], Shaun Treweek[1], Paula Williamson[2], Marion Campbell[1]
[1]University of Aberdeen; [2]Liverpool University
**Correspondence:** Katie Gillies
*Trials* 2017, **18(Suppl 1):**P40

This abstract is not included here as it has already been published.

### P41

#### Surrogate endpoint evaluation in a personalized medicine framework

Jared Foster[1], Ranran Dong[2], Qian Shi[1]
[1]Mayo Clinic; [2]The Ohio State University
**Correspondence:** Jared Foster
*Trials* 2017, **18(Suppl 1):**P41

Most contemporary methods in the field of surrogate endpoint evaluation involve assessing the degree to which average treatment effects on the surrogate and true endpoints are correlated (i.e. The trial-level surrogacy), using data from a (generally) small number of randomized clinical trials RCT). Because the number of relevant clinical trials is generally small, these methods may produce estimates of trial-level surrogacy that are highly variable. To this end, we consider the evaluation of potential surrogate endpoints within a personalized medicine framework. In particular, we consider a two-step procedure. In step 1, the surrogate and true endpoints are modeled as a function of treatment received, and other patient characteristics. Using these models, we obtain estimated, conditional (on patient characteristics), subject-specific treatment effects on the true and potential surrogate endpoints for each patient. In step 2, the estimated, subject-specific treatment effects on the true endpoint are modeled as a function of those on the surrogate endpoint using linear regression, and the trial-level surrogacy is estimated using the R-Squared from this model. Preliminary simulation studies suggest that, in many cases (when appropriate models are selected for the surrogate and true endpoint, and when certain other assumptions hold), this estimate of trial-level surrogacy has dramatically lower variance than some more traditional estimates of trial-level surrogacy.

### P42

#### Comparison and impact of prospective and retrospective falls data completion methods in the prefit trial: results of a randomised methodology study within a trial (SWAT)

James Griffin[1], Emma J. Withers[2], Ranjit Lall[2], Julie Bruce[2], Susanne Finnegan[2], Sallie E. Lamb[3], prefit Study Group[2]
[1]Warwick Clinical Trials Unit; [2]Warwick Clinical Trials Unit, University of Warwick; [3]Kadoorie Centre for Critical Care Research and Education, John Radcliffe Hospital, Nuffield Department of Orthopaedics, Rheumatology and Musculoskeletal Sciences, University of Oxford
**Correspondence:** James Griffin
*Trials* 2017, **18(Suppl 1):**P42

##### Background

Falls are a substantial health risk in older people. The collection of accurate falls data is problematic within clinical trials at several levels1. In particular there are issues with reporting falls when these events are associated with recall bias. Different data collection methods have been proposed to minimise bias. In the prefit trial2 we performed a study within a trial (SWAT3) to compare two common methods' daily falls diaries and retrospective reporting within quarterly questionnaires. Swats are an increasingly popular method to investigate uncertainties faced by researchers when conducting and designing randomised controlled trials.

##### Methods

The prefit trial recruited community dwelling older people from primary care. We compared alternative falls reporting methods to assess the impact on the likelihood of response, prevalence and pattern of missing values, and agreement between data sources. We also compared baseline participant characteristics by completion status. Participants were asked to complete a four month period of prospective fall diary completion; participants were randomly allocated to one of the periods (baseline to 4 months, 5 months to 8 months or 9 months to 12 months). Falls diaries were produced in a calendar format, posted to participants in a pack of four, with a covering instruction letter. Participants also completed follow-up questionnaires, containing a retrospective question on number of falls in the preceding months at 4, 8, 12 and 18 months post randomisation.

##### Results

A total of 9375 participants were requested to complete diary cards over the three time periods. Generally, diaries were well completed with 69% of participants completing all four diaries, and 83% completing at least one diary card. Completion rates were consistent across each of the three time intervals. There was a small but statistically significant increase in the proportion of people not returning a diary over the three successive time periods ($p < 0.001$). Those allocated to complete diary cards were more likely to withdraw from follow-up questionnaires than those not allocated to complete diaries in the same 4 month period. This was a small but consistent effect over the entire study (difference in rates of ~2%). In those participants who returned all diary cards and a corresponding questionnaire, falls were underreported in the questionnaire. People who returned no diaries were older, had poorer levels of physical and mental health, and had poorer cognitive function as well as a higher number of falls and fractures reported in their corresponding follow up questionnaires.

##### Conclusions

This SWAT provides evidence that allocation to complete prospective diary cards alongside four-monthly retrospective postal questionnaires has a small but significant effect on withdrawal from the main trial. Retrospective and prospective falls data are not consistently reported when collected simultaneously. People who did not return diaries were systematically in poorer health than those who completed all allocated diary cards. Swats are an efficient additional component of RCT design and should be considered to improve the design of future trials.

### P43

#### Designing trial outcomes for rare diseases

Eftychia Psarelli[1], Trevor F. Cox[1], Lakshminarayan Ranganath[2]
[1]Liverpool Cancer Trials Unit, University of Liverpool; [2]Royal Liverpool University Hospital
**Correspondence:** Eftychia Psarelli
*Trials* 2017, **18(Suppl 1):**P43

The selection of appropriate endpoints is of paramount importance for a clinical trial to meet its objectives. For some diseases it is difficult to choose a single endpoint or a few multiple endpoints that measure the disease from which a comparison of treatments can be made. This can be especially true for some rare diseases, where a major challenge in clinical trial design is the lack of a validated well-characterised efficacy endpoint. In order to assess disease severity in people with a rare condition such as alkaptonuria (AKU) - an orphan inborn homogentisate dioxygenase enzyme deficiency resulting in accumulation of homogentisic acid - a new tool was developed. The AKU Severity Score Index (AKUSSI) incorporates multiple, clinically meaningful outcomes that can be described in a single score. AKUSSI consists of both subjective and objective features that have been selected on current knowledge of the disease and it is sensitive to all morbid features of the condition. This score is a quantifiable, multidisciplinary assessment system, with the potential of reflecting changes in disease severity over time. Clinical experts, patients and statisticians were part of the development team. Tools like AKUSSI that describe disease manifestations can be used to compare disease across patients at different time points for other complex and multi-systemic diseases. Details and rationale of the AKUSSI tool that is now used as an outcome in a Phase III efficacy study (SONIA 2) will be described, with special attention to issues arising from the rarity of the disease.

### P44

#### A systematic search of clinicaltrials.gov to assess the uptake of core outcome sets

Jamie J Kirkham[1], Mike Clarke[2], Paula R Williamson[1]
[1]MRC North West Hub for Trials Methodology Research, Department of Biostatistics, University of Liverpool, Liverpool, UK; [2]Northern Ireland Hub for Trials Methodology Research, Centre for Public Health, Queen's University Belfast, Belfast, UK
**Correspondence:** Jamie J Kirkham
*Trials* 2017, **18(Suppl 1):**P44

This abstract is not included here as it has already been published.

### P45

#### Concealing the randomised allocation in trials: experience from the thermic trials

Julia Edwards[1], Katie Pike[1], Sarah Baos[2], Massimo Caputo[3], Chris A. Rogers[1]
[1]Clinical Trials and Evaluation Unit, School of Clinical Sciences, University of Bristol; [2]School of Social and Community Medicine, University of Bristol; [3]Bristol Royal Hospital for Children, Division of Women and Children, University Hospitals Bristol NHS Foundation Trust
**Correspondence:** Julia Edwards
*Trials* 2017, **18(Suppl 1):**P45

**Background**
In paediatric open-heart surgery body cooling during cardiopulmonary bypass (CPB) is commonly used to help protect vital organs. However, hypothermia can have detrimental effects. Thermic-1 was a parallel-group open randomised controlled trial which recruited 59 children undergoing heart surgery between 2002 and 2004. Patients were randomised to receive either hypothermic (28 °C) or normothermic (35 °C –37 °C) CPB. Thermic-2 followed on from Thermic-1, randomising 141 patients between 2012 and 2014. The co-primary outcomes included intubation time and length of post-operative stay.
**Methods**
Randomisation: The 10-year gap between phases saw changes in randomisation systems. In Thermic-1 allocations were placed in opaque sequentially numbered sealed envelopes, which were given to the clinical fellow managing the study. Thermic-2 was managed by the clinical trials unit with allocation determined by secure computerised system. Data capture: Data capture processes also changed between the two phases. A clinical fellow collected data on Excel spreadsheets in Thermic 1, whereas data were collected by research nurses in Thermic-2 and then entered into a purpose-designed database. Statistical analysis: Data from the two trials were pooled in one overall analysis adjusted for study phase. Interaction terms were added to the models to examine differences between trial phases.
**Results**
Baseline characteristics: Imbalances in patient demographics were observed in Thermic-1; participants allocated to the normothermic group were on average 3 years older (median 7.5 years [IQR 3.5-10.6] vs 4.3 [2.2-11.5]) and more likely to be male (68% vs. 48%). In contrast, in Thermic-2 no imbalance was observed; the median age was 2.3 years (0.5-5.2) in the normothermic group vs 2.9 (0.5-6.0) and there were similar proportions of males in the two groups (43% vs. 44%). Primary outcomes: Pooling the data across both phases, intubation time was slightly shorter in the normothermic group (median 10.6 hours [IQR 5.9-25.3] vs 16.4 [6.1-26.6]), although this was not statistically significant (hazard ratio [HR] 1.14, 95% CI 0.86-1.51, $p$-value = 0.36). The median duration of post-operative stay was 6.0 days in both groups (IQR 5.0-7.0); HR 1.06 (95% CI 0.80-1.40), $p$-value = 0.70. Examining the results by phase found no difference in treatment estimates for intubation time. However a significant difference between the two phases was found for length of stay (p-value for interaction = 0.079). The estimated HR was 1.57 (95% CI 0.93-2.64) in Thermic-1, i.e. Marginally favouring the normothermic group, compared to 0.90 (95% CI 0.65-1.26) in Thermic-2.
**Discussion**
The imbalance in baseline characteristics suggests that Thermic-1 results are at high risk of bias due to inadequate concealment of randomisation. Allocation compliance was only collected in Thermic-2, so the true extent of non-compliance could not be determined. Additionally, the differing results for post-operative stay suggest the study was also at risk of detection bias; the age and gender differences did not account for the difference observed. While the decision to extubate is protocol driven, the decision to discharge patients lies with the clinical team. The results illustrate the importance of methodological rigour in the design and conduct of clinical trials and provide a valuable example of the importance of working with methodologists.

### P46

#### Using a logic model and a triangulation protocol for integrating quantitative and qualitative research data in a mixed-methods feasibility study incorporating an external pilot RCT

Daniel Hind
University of Sheffield
*Trials* 2017, **18(Suppl 1):**P46

**Background**
Funders often encourage the use of both qualitative and quantitative data in evaluations. Such evaluations are sometimes seen as limited without formal approaches to the integration of qualitative and quantitative data [1], and dismissed as multi-method rather than truly mixed-method. Qualitative research is encouraged during feasibility/pilot work [2]. We used a version of a protocol suggested by Farmer and colleagues [3] to integrate and compare quantitative and qualitative findings (methodological triangulation of data sets) in a mixed-methods feasibility study of a hydrotherapy intervention for Duchenne Muscular Dystrophy (NIHR HTA 12/144/04).
**Methods**
A logic model, a tool used to evaluate the implementation of a care programme [4], was developed with collaborating interventionists. We reviewed qualitative and quantitative datasets to identify components of the intervention logic model ("sorting"). A convergence coding matrix summarized similarities and differences between data sets for each of 17 logic model components, selecting examples to demonstrate how each had contributed to the intervention's success or failure ("convergence coding"). We applied a convergence coding scheme: "agreement"; "partial agreement"; "silence"; or, "dissonance". We quantified the level of agreement between data sets ("convergence assessment") And highlighted their different contributions to the research question ("completeness comparison"). We shared the triangulated results with team members and other selected stakeholders at a face-to-face meeting, for feedback, allowing points of disagreement to be discussed and changes in interpretation incorporated.
**Results**
There was agreement on six components, silence on eight (areas only amenable to qualitative assessment), and dissonance on two. The areas of dissonance concerned session attendance and intervention optimisation. In each case, a naïve reading of the quantitative data could lead to an overly simplistic attribution of cause. For session attendance, quantitative sub-studies pointed to illness or simple non-appearance of the family; the qualitative data revealed that the convenience of available timeslots played a strong role in non-attendance for some families. Similarly, quantitative data identified an apparent failure, on the part of several physiotherapists, to optimise the intervention; the qualitative data revealed this to be part of a misunderstanding, with therapists wrongly assuming that the study required them to apply the manual prescriptively or extensively, rather in a focused and more achievable way proposed at training. Those same therapists were aware and concerned that therapy was not optimised. Qualitative research contributed data to 15/17 logic model components; quantitative components contributed to nine. Samples from the convergence coding matrix are presented in the presentation. Feedback from stakeholders confirmed the account offered and adequate explanation of events observed in the study.
**Discussion**
We selected a different methods appropriate to the commissioning brief, but did not implement methods independently. A formal mixed-methods approach allowed the robust use of qualitative data used to explain quantitative findings.

**References**
1. O'Cathain A: BMJ 2010, 341(sep17 1):C4587-C4587.
2. O'Cathain A. Health Technol Assess 2014, 18:1–197, V-Vi.
3. Farmer T. Qual Health Res 2006, 16:377–94.
4. Mclaughlin JA. Eval Program Plann. 1999 Mar;22(1):65–72.

### P47

#### Exploring discrepancies between long-term condition review consultation audio-recordings and computer template data (the ENHANCE pilot trial)

Jennifer Liddle, Sarah A. Lawton, Carolyn A. Chew-Graham, Emma L. Healey, Christian D. Mallen, Clare Jinks
Keele University
**Correspondence:** Jennifer Liddle
*Trials* 2017, **18(Suppl 1):**P47

**Background**
The ENHANCE pilot trial aimed to test the feasibility and acceptability of integrating case-finding for osteoarthritis, anxiety and depression within extended primary care nurse-led long-term condition (LTC) review consultations. Training was delivered to general practice nurses (PNs) to deliver the ENHANCE reviews, supported by an adapted EMIS LTC computer template.
**Objectives**
This analysis explored the extent to which data recorded by the PNs in the ENHANCE EMIS template reflected the content of discussions and case-finding in ENHANCE LTC review consultations. The findings form part of a process evaluation exploring the ways in which PNs delivered ENHANCE LTC reviews.
**Methods**
Patients and PNs in four general practices were asked to give consent for their ENHANCE consultations to be audio-recorded for fidelity checking (24 patients and seven PNs consented). 12 patients also gave consent for the research team to access their medical record data, which included the ENHANCE template data (entered by six PNs during ENHANCE reviews). Consultation recordings for these 12 patients were compared with corresponding ENHANCE EMIS template data entered by the PNs, to identify and explore any discrepancies.
**Results**
Use of the ENHANCE case-finding questions in the audio-recorded ENHANCE LTC review consultations was high. The majority of patient responses to case-finding questions/tools in the audio-recordings matched those recorded by PNs through the new ENHANCE EMIS template, however, 12 discrepancies between the audio-recordings and EMIS computer template data were identified, arising from five of the consultations (with three PNs). Discrepancies included: responses to case-finding questions not matching; responses recorded in the template data for questions not asked in the audio-recording; missing template data for questions that were in the audio-recording. Some discrepancies appeared to arise from PNs' understandings of what constituted a legitimate "Yes" or "No" response to the case-finding questions for depression and anxiety. There was also evidence that PNs sometimes attempted to question, dismiss or normalise patients' initial responses.
**Conclusions**
Data demonstrate that PNs were generally recording responses to case-finding questions using the ENHANCE EMIS template as intended, suggesting that this process within the ENHANCE study was feasible and accurate. PNs were asked to record patient responses on a new computer template while maintaining a patient-centred dialogue and completing an integrated ENHANCE review within the available timeframe, so it is unsurprising that some typing errors or discrepancies may occur. Nonetheless, it is helpful to acknowledge that these may exist, as template data is often used for fidelity checking of intervention delivery within trials. We have identified difficulties in the use of case-finding questions that could be addressed through PN training in a future main trial.

### P48

#### Effective teamwork is crucial to maximising recruitment to randomised controlled trials in surgical oncology

Sean Strong, Sangeetha Paramasivan, Nicola Mills, Jenny Donovan, Jane Blazeby
University of Bristol
**Correspondence:** Sean Strong
*Trials* 2017, **18(Suppl 1):**P48

**Background**
Trials in surgical oncology frequently experience issues with recruiting adequate numbers of participants. This is particularly difficult within RCTS involving interventions which are routinely delivered by different clinical specialties (such as surgery and oncology based treatments). Teamwork between individual healthcare professionals and specialty and research teams has been highlighted as a significant factor in recruitment. This study evaluated aspects of teamwork which were important in recruitment to three RCTs in surgical oncology.
**Methods**
In depth semi structured interviews were conducted with a purposeful sample of healthcare and research professionals responsible for recruitment in three RCTs in different disease sites in surgical oncology (oesophgago-gastric, thoracic and colorectal). Interviews were audio recorded, transcribed verbatim and analysed thematically. Sampling, data collection and analysis were undertaken iteratively and concurrently.
**Results**
Thirty six interviews were conducted with recruiters at seven different hospital sites. Sites in which a culture of clinical collaboration within and across disciplines existed recruited more participants than those in which individual clinicians tended to work in isolation. The multidisciplinary team meeting (tumour board meeting) appeared to facilitate cross disciplinary collaboration and was an important factor in determining the ability of individual sites to effectively recruit. The degree to which individual specialty teams within each centre were in equipoise influenced study engagement.
**Discussion**
This study has demonstrated several aspects of teamwork that appear to be important for recruitment in trials in surgical oncology. Understanding these aspects of teamwork will aid the development of guidance on team relevant issues that should be considered in trial management and the development of interventions that will facilitate teamwork and improve future recruitment to RCTs.

### P49

#### Exploring understanding of neural stem cell transplantation (NSCT) as an intervention for Huntington's Disease (HD)

Richard Hellyar
School of Healthcare Sciences, Cardiff University
*Trials* 2017, **18(Suppl 1):**P49

**Background**
Neural Stem Cell Transplantation (NSCT) has been identified as a potential therapeutic intervention for the treatment of Huntington's disease (HD) (Dunnett and Rosser, 2007). This neurosurgical procedure utilises stem cells, which are injected into the mid-brain of affected individuals and are intended to improve symptoms (Lindvall and Bjorkland, 2000). Previous research, utilising NSCT, has briefly acknowledged ethical sensitivity including the source of cells utilised, alongside the hopes of HD patients surrounding the intervention (Bachoud-Levi et al., 2000). The understanding of NSCT amongst potential beneficiaries has however yet to be explored in depth. With future clinical trials being planned to explore the intervention, this proposed qualitative project seeks to redress this gap and it is envisaged that the understanding gained will inform information giving, recruitment strategies and care pathway planning in such a way as to augment any future participant experience.
**Method**
The primary aim of this research is to gain insight into the perceptions and understanding about Neural Stem Cell Transplantation (NSCT) amongst potential recipients. The information gained is then intended to inform and underpin the development of information giving approaches for potential NSCT recipients and ensure their issues are addressed in the development of consent procedures and care pathways. Firstly three purposively targeted Specialist Professionals, from the field of NSCT, have been approached via email, consented and interviewed (semi-structured) in order to explore their past experience. The recorded interviews addressed their recollections of the recipient experience, their understanding, questions, queries and concerns with regards to NSCT. A thematic analysis of these interviews has been undertaken and used to inform and guide the development of minimally-structured interviews with six, genetically positive, individuals who have yet to show symptoms of HD. Emergent themes thus far include Making Sense via Contrast, Chronological Risk, Ethical Dissonance and Familial/Community Drivers and Brakes. This second phase, using minimally-structured qualitative interviews, is intended to elicit the perceptions and understanding surrounding NSCT as an intervention amongst potential recipients. These participants will be recruited from an Asymptomatic Huntington's Disease clinic.

**Results**

As future clinical trials of NSCT are due to be undertaken in the United Kingdom in the near future, it is important that this (anonymised) information from this thesis is shared with Professionals, working within Clinical Trials, in order to support recruitment strategies and information provision.

### P50

#### Development and first evaluation of the atlas training toolkit for clinical trials

Athanasia Gravani[1], Marcus Jepson[1], Caroline Wilson[1], Athene Lane[1], Chris Rogers[2]
[1]MRC conduct-II Hub for Trials Methodology Research, School of Social and Community Medicine, University of Bristol, Bristol, UK; [2]Clinical Trials and Evaluation Unit, School of Clinical Sciences, University of Bristol, Bristol, UK
**Correspondence:** Athanasia Gravani
*Trials* 2017, **18(Suppl 1):**P50

**Background**

Trial-specific training is highly varied across trials and there is great uncertainty on the best ways to provide training to facilitate trial conduct.

**Aims**

To develop and validate the ATLAS training toolkit that can be utilised by trial management teams when planning training of staff within clinical trials. Part of the training toolkit also aims to evaluate the process of trial-specific training provided to site staff during the site initiation process.

**Methods**

The content of the training toolkit was developed by combining i) qualitative data obtained from semi-structured interviews with trial managers (n = 6) and healthcare professionals (n = 13) working on six purposefully selected case studies of the ATLAS project; ii) responses to questionnaires (n = 120) used to evaluate site staff and facilitators' experience of site initiation training sessions, iii) a review of existing regulations and guidance documents from various regulatory bodies (MRC, HRA, MHRA, FDA) on training requirements in clinical research; and iv) a review of existing literature on the processes of learning, training and development. The training toolkit was then evaluated in two-stages. Firstly, semi-structured follow-up interviews with the trial managers (n = 6) facilitating trial-specific training sessions in the six participating ATLAS studies were undertaken and the toolkit was amended in light of the feedback received. At the second stage, feedback was sought on the revised toolkit from trial managers attending scheduled meetings (n = 2) in two established Clinical Trial Units in Bristol.

**Results**

The toolkit has five components each focusing on a particular element of the training cycle: i) Specifying initial training needs and selecting appropriate mode of delivery; ii) Designing the training plan; iii) Delivering and documenting training; iv) Evaluating training and v) Identifying additional training needs and re-training of staff. Each element is supplemented by support documents (including flowcharts and template documents) that can be utilised by trial managers as guides when planning trial-specific staff training. Overall, the training toolkit was positively received and was considered a useful reference document encouraging active thinking of staff training during the early stages of study design. Most trial managers felt that the decision-making flowchart provided useful prompts to assist trial managers in selecting the appropriate mode of training during the decision-making process. The training plan template was also viewed as a helpful document for recording decisions about the appropriate level of training required for each study. The training feedback forms were regarded as invaluable documents in identifying key areas where additional training is required and improving future training sessions.

**Conclusions**

To the best of our knowledge this is the first training toolkit that has been developed to assist trial managers in planning, designing, documenting and evaluating staff training within clinical research. Further validation of the toolkit is required to assess its practical use in a variety of clinical trial settings.

### P51

#### Use of simulations to explore recruitment and the impact of late availability of experimental arms in the pivotalboost trial

Nuria Porta[1], Clare Griffin[1], Isabel Syndikus[2], John Staffurth [3], Peter Hoskin[4], David Dearnaley[5], Ann Henry[6], Brendan Carey[6], Alison Tree[5], Rebecca Lewis[1]
[1]The Institute of Cancer Research; [2]The Clatterbridge Cancer Centre; [3]Velindre NHS Trustl; [4]Mount Vernon Cancer Centre; [5]Royal Marsden NHS Foundation Trust; [6]Leeds University Teaching Hospitals
**Correspondence:** Nuria Porta
*Trials* 2017, **18(Suppl 1):**P51

**Background**

pivotalboost (CRUK/16/018) is a 4-arm phase III randomised trial in patients with node negative, high or intermediate risk localised prostate cancer currently in set-up. 1952 patients will be randomised to receive (A) standard prostate intensity modulated radiotherapy (IMRT); (B) A with pelvic node IMRT; (C) A with a prostate boost; or (D) A with pelvic node IMRT and a prostate boost. The prostate boost can be delivered by IMRT or by high dose rate brachytherapy (HDRB). Availability of the boost arms C and D depends on: (1) A suitable boost tumour volume identified by functional MRI. (2) Availability of boost technologies - participating sites can open initially to A vs B randomisation with the boost arms (i.e. 4-arm randomisation) opening later. (3) Patient suitability and fitness. The study is powered to compare each experimental arm with control. Due to the above recruitment restrictions for the boost arms, power was reduced for the boost comparisons (85% power for A vs B, 80% A vs C and A vs D), giving a design where the trial population will be split 9:9:8:8 across arms.

**Aims**

To investigate, via simulation of recruitment, how the three elements above impact recruitment and imbalance between treatment arms and how adaption of the allocation ratio could minimise imbalances.

**Methods**

We assumed recruitment would take 54 months and with staggered opening, all 40 sites would be open by 24 months. Expected site-specific monthly accrual rates and availability of boost technologies were obtained via site feasibility questionnaires. An expected month of opening and when the boost arms would be available at each site was inferred using survey results and clinician input. Recruitment was assumed to follow a Poisson distribution with site-specific monthly accrual rates. For each patient accrued at a specific month we simulated the boost volume, risk group and suitability for receiving a boost, so the patient could be allocated to the 2-arm or 4-arm randomisation accordingly. We performed initial simulations with a

1:1:1:1 allocation ratio and reviewed monthly mean recruitment per treatment, allocation ratio to control (X:A) and probability of completing recruitment before the planned time. We explored the impact of delays in opening the boost arms on prolonging the recruitment period.

**Results**

Simulations showed that using a 1:1:1:1 allocation ratio causes an initial imbalance with more patients allocated to A and B as expected by late opening of boost arms, which could result in an imbalance at the end of recruitment in favour of the control arm; though infrequent, some simulated trials had up to 25% more patients in one group than another. Initial use of 2:2:3:3 allocation ratio appeared to protect against such imbalances. Recruitment by arm will be monitored as the trial progresses with a planned adaption to a 1:1:1:1 allocation ratio part way through recruitment.

**Conclusions**

Simulation of recruitment proved useful to understand the potential imbalances that may occur during the trial and led to a cost-effective strategy of different allocation ratios during the trial to correct for initial imbalance.

### P52

#### A pilot randomised controlled trial of community led anti-psychotic drug reduction for adults with learning disabilities: ANDREA-LD

Elizabeth Randell, Rachel McNamara, Lianna Angel, David Gillespie, Andrea Meek, Mike Kerr
Cardiff University
**Correspondence:** Elizabeth Randell
*Trials* 2017, **18(Suppl 1):**P52

**Background**

Data suggests there are 50,000 adults with learning disabilities (LD) in England and Wales currently prescribed antipsychotic medication. Illness in this population is high, including significant rates of challenging behaviour and mental illness with particular concern over use of anti-psychotic drugs prescribed for reasons other than treatment of psychosis. Control of challenging behaviour is the primary reason why such medications are prescribed, despite the absence of good evidence of therapeutic effect for this. This innovative study was initially conducted in primary care however due to complexities surrounding set up and recruitment, continued in community learning disabilities teams as a feasibility study. The primary objective was to assess feasibility of recruitment and retention, and explore non-efficacy based barriers to a blinded anti-psychotic medication withdrawal programme for adults with LD without psychosis compared to treatment as usual. A secondary objective was to compare trial arms regarding clinical outcomes.

**Method**

ANDREA-LD was a two arm individually randomised (1:1) double blind placebo controlled drug reduction trial. The majority of recruitment was through community learning disabilities teams in South East Wales and South West England. Participants were adults with LD prescribed risperidone for treatment of challenging behaviour with no known current psychosis or previous recurrence of psychosis following prior drug reduction. Carers also consented to their involvement in the trial.

The intervention was a double blinded drug reduction programme leading to full withdrawal within six months. The control group maintained baseline treatment. Treatment achieved at six months was maintained for a further three months under blind conditions. The blind was broken at nine months following final data collection.

Feasibility outcomes were number and proportion of: (i) sites progressing from initial approach to participant recruitment (ii) recruited participants who progressed through the trial. Trial arms were also compared regarding; Modified Overt Aggression Scale; Aberrant Behaviour Checklist; PAS-ADD checklist; Antipsychotic Side-effect Checklist; Dyskinesia Identification System Condensed User Scale; Client Service Receipt Inventory; use of other interventions for challenging behaviour; use of as required medication; psychotropic medication use.

**Results**

Of the 22 participants randomised, 13 (59.1%) achieved progression through all four stages of reduction. Follow-up data at six and nine-months post-randomisation was obtained for 17 participants (77.3% of those randomised) with 10 intervention and seven control participants followed up. There were no significant changes in participants' levels of aggression or challenging behaviour at the end of the study.

Methodological challenges faced in setting up and delivering the trial included: recruitment of principal investigators and sites equipped to distribute medication; recruitment of participants and carers; obtaining consent according to regulations surrounding trials for this vulnerable population; ensuring maintenance of the blind and dispensing medication to participants; carers subjective assumptions of trial arm allocation.

**Conclusions**

Results indicate that drug reduction is possible and safe. However focused support and alternative interventions are required. The results of the qualitative study and reflections on the challenges faced provide important insights into the experiences of people taking part in drug reduction studies that should influence future trial development.

### P53

#### Barriers to recruitment from primary care into a trial in secondary care settings: experience from the feasibility study of IBIS-3 trial

Adedayo Oke[1], Jill Knox[1], Jermaine Tan[1], Benoit Aigret[1], Peter Schmid[1], Robert E. Coleman[2], Jack Cuzick[1], Mangesh A. Thorat[1]
[1]Queen Mary University of London; [2]University of Sheffield
**Correspondence:** Adedayo Oke
*Trials* 2017, **18(Suppl 1):**P53

**Background**

Oestrogen Receptor (ER) positive breast cancer (ERBC) is now a chronic disease with high 5-year survival rates. However, a large proportion of cases continue to be at a substantial risk of recurrence up to 20 years from diagnosis. Trials of interventions designed to prevent late recurrences in ERBC face a unique challenge. These interventions often need to be carried out in secondary care setting when patients have already been discharged back into primary care. Therefore recruitment from the primary care setting is important for such trials. The IBIS-3 feasibility study is a randomised controlled trial (RCT) of interventions in long-term survivors of ERBC with recruitment rates from primary case as one of its objectives.

**Method**

Five Clinical Research Networks (CRNs) invited GP practices close to trial's participating Secondary Care Sites (SCS) to join as Participant Identification Centres (PICs) on our behalf. GPs who agreed to participate as PICs screened their databases to identify potentially eligible patients and wrote to these patients inviting them to participate in the trial by contacting the trial's central coordinating office (CCO). The CCO further checked eligibility and referred patients to their local SCS. After 6 months of original request, a brief survey to identify main reasons for non-participation was sent to all GPs who declined participation.

**Results**

The level of support provided to both the CCO and GPs varied across 5 CRNs potentially impacting GP participation rate. Overall, only 5% GPs agreed to participate and only 23 of 800 (3%) subsequently responded to the survey. The main reasons identified for non-participation were lack of time/resources to carry out database search (61%) and/or review medical records to confirm eligibility (48%), request coming at a busy time (9%) e.g. Calendar or financial year-end, and insufficient funding (26%). Encouragingly, 26% of GPs that completed the survey indicated willingness to participate at the time of the survey.

**Conclusions**

Wide variations exist in the level of support provided to GPs across CRNs. Ensuring uniform and higher levels of support including funding to help overcome time/resources scarcity barriers is likely to improve GP participation as PICs for trials in secondary care settings. A re-request for participation from CRNs, made at a time when practices are less busy should also be considered as a measure to improve participation.

### P54

#### Does advertising patient and public involvement in a trial to potential participants improve recruitment and response rates? An embedded cluster randomised trial

Adwoa Hughes-Morley[1], Mark Hann[2], Claire Fraser[3], Oonagh Meade[4], Karina Lovell[3], Bridget Young[5], Chris Roberts[6], Lindsey Cree[3], Donna More[3], Neil O'Leary[7]
[1]University of Manchester and University of York; [2]NIHR School for Primary Care Research, University of Manchester; [3]School of Nursing, Midwifery and Social Work, University of Manchester; [4]School of Health Sciences, The University of Nottingham; [5]MRC North West Hub for Trials Methodology Research, University of Liverpool; [6]Department of Biostatistics, University of Manchester; [7]Centre For Medical Gerontology, Trinity College Dublin
**Correspondence:** Adwoa Hughes-Morley
*Trials* 2017, **18(Suppl 1):**P54

**Background**
There is emerging evidence that patient and public involvement in research (PPIR) may increase participant recruitment into randomised controlled trials. However, it is not clear how to use PPIR to improve trial recruitment. Whilst publicly funded trials in the UK and elsewhere routinely use PPIR to improve design and conduct, such trials on the whole do not advertise their use of PPIR to potential participants. Effective advertising of PPIR in trials to potential participants might increase enrolment rates, through trials being perceived to be more trustworthy, relevant and socially valid.
**Aims**
We aimed to develop an intervention directly advertising PPIR in a trial to potential participants and evaluate its impact on trial recruitment and response rates.
**Methods**
We undertook a cluster randomised controlled trial, embedded in an ongoing "Host" Mental health trial (the "EQUIP" Trial). EQUIP was a cluster randomised controlled trial recruiting service users with a diagnosis of severe mental illness. In EQUIP, mental health teams in England were randomised to an intervention group to receive training to improve service user and carer involvement in care planning, or to a "no training" control group. The recruitment intervention advertising PPIR was informed by a systematic review, a qualitative study of patients who declined a trial, social comparison theory and a workshop that included mental health service users and trialists. Using Participatory Design methods, we collaborated with PPIR partners (service users and carers) to design a recruitment intervention using a leaflet format to advertise the nature and function of the PPIR in EQUIP to potential participants. Professional graphic design aimed to optimise the intervention's readability and impact. Service users being approached into EQUIP were randomised to the PPIR intervention or not, alongside the standard trial information. The primary outcome was the proportion of participants enrolled in EQUIP. The secondary outcomes included the proportion expressing interest in enrolling. Analysis was by intention to treat and used generalised linear mixed models.
**Results**
We randomised 34 mental health teams and 8182 potential participants were invited. For the primary outcome, 4% of patients receiving the PPIR leaflet were enrolled vs. 5.3% in the control group. After adjusting for mental health team cluster size, levels of deprivation and care quality rating, the intervention was not effective for improving recruitment rates (adjusted OR = 0.75, 95% CI = 0.53 to 1.07, p = 0.113). For the secondary outcome 7.3% of potential participants receiving the PPIR leaflet responded positively to the invitation to participate, vs. 7.9% in the control group. The intervention was not effective for improving response rates (adjusted OR = 0.74, 95% CI = 0.53 to 1.04, p = 0.082). The intervention was not effective for any other outcomes measured.
**Conclusion**
This is the largest embedded trial to test the impact of a recruitment or PPIR intervention. Advertising PPIR using a leaflet had no benefits for improving recruitment or response rates. Our findings contrast with the literature suggesting advertising PPIR benefits trial recruitment. We will discuss implications of our findings for trial recruitment, research and policy.

### P55

#### How do recruiters present randomisation during trial recruitment? An evaluation of current practice

Carmel Conefrey, Julia Wade, Marcus Jepson, Daisy Elliott, Jenny Donovan, The Quintet Team
University of Bristol
**Correspondence:** Carmel Conefrey
*Trials* 2017, **18(Suppl 1):**P55

**Background**
Recruitment to randomised controlled trials (RCT) remains one of the key challenges in trial management. Patient aversion to randomisation is often cited as a reason why patients choose not to enroll in RCTs. For many recruiters and patients alike, 'randomisation' appears a challenging concept, yet one that requires communicating and understanding given its centrality to informed consent and trial recruitment. The UK National Research Ethics Service (NRES) has produced guidance on how to describe randomisation simply and clearly in written patient information. We investigated how recruiters described randomisation in recruitment appointments and compared this with a framework based on the NRES guidance.
**Methods**
A maximum variation sample of 64 audio-recorded recruitment appointments was purposefully sampled from five RCTS to encompass a range of recruiters, surgical and non-surgical trials and cancer and non-cancer conditions. Using the NRES guidance for written patient information as a hypothesised ideal explanation of randomisation, an analytical framework was developed identifying five interlinked concepts considered necessary for a clear exposition of randomisation. This analytic framework was applied to extracts from consultations during which randomisation was discussed using content analysis, assessing whether the concepts were absent or present and explicit or implicit, according to coding rules derived from the data.
**Results**
Two key findings emerged. Firstly, recruiter explanations of and for randomisation tended to be incomplete when evaluated against the NRES informed framework: in nearly 45% (29) of cases, three or fewer components were present. Only five of the 64 encounters included mention of all five concepts and in only two of these were all five concepts made explicit. Secondly, recruiters referred to some concepts more frequently than others to articulate the rationale for randomisation. Whilst most recruiters referred to 'clinical equipoise' and 'the need for a number of patient treatment groups', few referred to 'the need for patient groups to be similar except for the treatment allocated'. Where expressed, recruiters tended to convey 'the need to compare treatment effects' and 'that chance determines assignment to a treatment allocation', implicitly rather than explicitly.
**Conclusion**
An evaluation of recruiter practice during recruitment consultations across a range of trials showed that recruiters did not explicitly communicate key concepts identified by NRES as fundamental to a clear definition of randomisation. There is a need to understand whether all aspects of the NRES guidance are necessary for the communication of randomisation, and which are the key concepts that are essential to facilitate patient understanding and assure informed consent.

### P57

#### Obtaining consent in the pre-hospital setting: consent procedures from the rapid intervention with glyceryl trinitrate in hypertensive stroke trial-2 (right-2)

Polly Scutt, Mark Dixon, Jason P. Appleton, Philip M. Bath
University of Nottingham
**Correspondence:** Polly Scutt
*Trials* 2017, **18(Suppl 1):**P57

**Background**
Stroke is a severe and often fatal or disabling condition. Despite treatment effects in acute stroke being predominantly time dependent (e.g. Thrombolysis and thrombectomy), proven treatments are hospital based. Commencing treatment in the pre-hospital setting could dramatically reduce time to treatment. The rapid intervention with glyceryl trinitrate in hypertensive stroke trial-2 (RIGHT-2) recruits patients in the pre-hospital setting within 4 hours of stroke onset. Obtaining consent in emergency situations can be difficult, especially when the time window for recruitment is short. Proxy consent allows patients to be recruited when they lack capacity to give consent themselves, a common scenario in people suffering a stroke. Conversely, a waiver of consent offers the opportunity to include all eligible patients but may disregard the initial choice of patients who have capacity to make an informed decision regarding participation in research.
**Methods**
Ethics approval was obtained to allow proxy consent to enable randomisation into the RIGHT-2 trial within the 4 hour recruitment window. Informed consent or proxy consent is taken at the stroke scene or in the ambulance. Brief assessment of capacity is performed by the paramedic explaining to the patient that they have had a suspected stroke, their BP may need lowering, and that a patch will be applied that might lower their BP. The paramedic then asks the patient what the suspected diagnosis is ('stroke'), what might need to be done to their BP ('lower'), and how this will be done ('patch'). Patients with capacity give written or witnessed oral consent to the paramedic. If a patient lacks capacity, proxy consent is obtained from a relative, carer or friend acting as a personal consultee, if available, or by the paramedic witnessed by another member of the ambulance staff at the scene. For participants who did not have capacity at the time of randomisation, consent is verified in hospital with themselves or a relative (if the participant still lacks capacity).
**Results**
As of 28th October 2016, 247 participants have been enrolled into the RIGHT-2 trial. 127 (51.4%) participants gave their own consent. Proxy consent was given by a relative/carer/friend for 97 (39.3%) participants, and by a paramedic for 23 (9.3%) participants. The median time to consent for all participants was 58 minutes. After the participants reached hospital, 141 (61.8%) gave their own consent, 45 (19.7%) had continued consent by a relative or close friend and 42 (18.4%) had no further consent after proxy consent was taken in the ambulance. Patients who had proxy consent in the ambulance had a more severe stroke, median [IQR] National Institutes of Health Stroke Scale score 12.5 [5, 18] versus 4 [2, 7] for those who gave consent themselves.
**Conclusion**
Proxy consent can ensure patients are enrolled rapidly into emergency clinical trials. In the RIGHT-2 trial, patients with a severe stroke, who may benefit from the intervention, are able to take part in the study when they would otherwise be excluded, which boosts recruitment and ensures the trial population is representative of the population the intervention is intended for.

### P58

#### Methods for training paramedics to recruit patients into the rapid intervention with glyceryl trinitrate in hypertensive stroke trial-2 (right-2)

Polly Scutt, Mark Dixon, Jason P. Appleton, Philip M. Bath
University of Nottingham
**Correspondence:** Polly Scutt
*Trials* 2017, **18(Suppl 1):**P58

**Background**
Paramedics are equipped to assess and recognise patients with suspected stroke in the out-of-hospital setting. Treatment is highly time-dependent but definitive intervention for stroke is currently limited to in-hospital therapies. Commencing treatment in the pre-hospital setting could dramatically reduce time to treatment. The rapid intervention with glyceryl trinitrate in hypertensive stroke trial (RIGHT-2) is assessing the safety and efficacy of pre-hospital ambulance-based paramedic-delivered glyceryl trinitrate (GTN) when administered ultra-acutely after stroke. Whilst ambulance-based paramedic-delivered stroke trials have been done in the UK in single site pilot trials, they have not been done across multiple ambulance services and hospital sites in the UK. It is important for paramedics to have awareness of Good Clinical Practice (GCP) principles and to be trained in trial procedures. Methods used to train paramedics need to account for the fact that paramedics have little time at work not on shift to complete training and may be reliant on them completing training in their own time.
**Methods**
Paramedics working in ambulance services involved with RIGHT-2 who express interest in recruiting patients into the trial are invited to watch the training video. The training video lasts for 1 hour and contains details on trial procedures and elements of GCP relevant to paramedics recruiting patients. The video is available over the internet so paramedics are able to watch and revisit it whenever they choose so they are able to complete the training in the small amount of time they have available. Training has been delivered in face-to-face small group sessions by Research Paramedics from participating ambulance trusts and members of the RIGHT-2 team. Training has also been delivered by a remote webinar, where the RIGHT-2 team deliver the training over the internet, which allows for interaction between paramedics and those delivering the training. Once paramedics have been trained they must complete an online assessment questionnaire based around the content of the video before they are able to recruit patients.
**Result**
Five ambulance services are currently recruiting into the RIGHT-2 trial. From these, 958 paramedics expressed interest in being involved with the RIGHT-2 trial, of which 628 (65.6%) have completed the online training assessment. Feedback from paramedics suggests that face-to-face is their preferred method of training, however, sessions need to be repeated several times to allow paramedics who are on rotating shift patterns to attend. This takes up a considerable amount of time for the research paramedic who has to travel large distances across the ambulance service. Remote webinars were well attended, with some paramedics attending multiple sessions to recap on key points. As with face-to-face sessions, multiple sessions are required for a reliable uptake of paramedics.
**Conclusion**
Training for paramedics who recruit patients into clinical trials needs to cover the necessary elements of GCP as well as trial procedures. It must be easy to access and succinct in order for them to complete training around their normal work schedule. A remote webinar provides balance between accessibility for paramedics and ability to interact with those delivering the training.

### P59

#### Sample size adaptations in a randomised controlled biomarker based strategy (hybrid) trial: experience from the OUTSMART trial

Dominic Stringer, Kimberley Goldsmith, Janet L. Peacock, Leanne M. Gardner, Caroline Murphy, Anthony Dorling
Kings College London
**Correspondence:** Dominic Stringer
*Trials* 2017, **18(Suppl 1):**P59

**Background**
OUTSMART is an ongoing multi-centre randomised controlled trial testing whether a combined structured biomarker screening programme and optimized immunosuppression treatment regimen can reduce risk of graft failure in kidney transplant patients. HLA antibodies (HLA Ab+), particularly if they contain donor specific antibodies against the graft (DSA+) are prognostic biomarkers for graft failure. However it is unclear how best to treat patients positive for the biomarkers.

The study design involves screening kidney transplant patients to determine if they are positive or negative for HLA antibodies and if HLA Ab+, whether they have donor specific antibodies (DSA+) against the graft. Antibody screening results are initially blinded, and then participants are randomised 1:1 to blinding or unblinding of their HLA Ab status. The blinded group remain treated as they were at baseline (standard care) and the unblinded HLA Ab + group are treated with an optimized immunosuppression intervention.
The first strategy used to improve efficiency was the incorporation of rescreening HLA Ab- participants in the trial design, with participants converting to HLA Ab + in the unblinded group moving on to optimised immunosuppression.
The known prevalence of HLA Ab + in renal transplant recipients is approximately 25%, DSA account for 1/3rd of these, and the known incidence of de novo DSA development is 3%. Sample size calculations based on these known rates estimated requiring 2800 randomised participants for 324 DSA+ participants to allow comparison of the effect of biomarker led immunosuppression optimisation in these patients. Sixteen months into recruitment both the prevalence and incidence of DSA+ was lower than expected. This led to the application of a second strategy for efficiency improvement; a change in the primary outcome from binary to time to event (time to graft failure, approved by the DMC and TSC). The sample size calculation was amended both to reflect this change and to take into account the lower than expected DSA+ rates, retaining trial power. The amended sample size required 165 DSA+ participants from an estimated 2357 to be recruited. This change also meant there was already sufficient numbers of HLA Ab + DSA- and HLA Ab- participants recruited.
As recruitment continued, the overall proportion of DSA+ participants in the trial increased as the DSA prevalence and incidence rates normalised, and also as a consequence of the increasing pool of participants to screen as more HLA Ab- participants were randomised. The latter sort of complexity would generally only be simplistically accounted for in sample size calculations. Close monitoring of biomarker rates and ongoing sample size updates can address such issues and improve trial efficiency. This has led to outsmart meeting recruitment targets earlier than expected, avoiding unnecessary randomisation of participants.

**Conclusions**

Pre-planned ongoing rescreening of biomarker negative participants and flexible reconsideration of the primary outcome allowed both enrichment for individuals with the biomarker of interest and dynamic modification of the sample size, leading to improved trial efficiency. Where possible, time-to-event or continuous primary outcomes should be used in trials, especially where recruitment might be difficult and/or biomarkers of interest are rare.

### P60

#### Strategies to improve participant recruitment to randomised controlled trials: a systematic review of non-randomised evaluations

Heidi Gardner, Cynthia Fraser, Graeme MacLennan, Shaun Treweek
University of Aberdeen
**Correspondence:** Heidi Gardner
*Trials* 2017, **18(Suppl 1):**P60

This abstract is not included here as it has already been published.

### P61

#### Surveying patients: views on trial information provision and decision making using the 'accept/decline' clinical trials questionnaire

Kathryn Monson[1], Tom Treasure[2], Chris Brew-Graves[3], Valerie Jenkins[4], Lesley Fallowfield[4]
[1]Brighton & Sussex Medical School, University of Sussex; [2]Clinical Operational Research Unit, University College London; [3]Surgical & Interventional Trials Unit, University College London; [4]Sussex Health Outcomes Research & Education in Cancer (SHORE-C), Brighton & Sussex Medical School, University of Sussex
**Correspondence:** Kathryn Monson
*Trials* 2017, **18(Suppl 1):**P61

**Background**

The Pulmonary Metastasectomy in Colorectal Cancer (pulmicc) trial completed its feasibility phase in 2015. Surgically treated colorectal cancer patients, with newly diagnosed lung metastases, were randomised to continued active monitoring or pulmonary metastasectomy followed by active monitoring. Randomisation was a two stage process; Stage 1 investigations assessed fitness for surgery and eligibility for Stage 2 randomisation. A key trial criterion was clinician uncertainty regarding the benefit of surgery in the light of the patient's test results. The trial was anticipated to be challenging for both clinicians and patients. Both patient information, and healthcare professional, training DVDs were produced to assist with trial discussions and decision making. Additionally a patient survey was conducted to examine patients' views about the trial.

**Method**

Following pulmicc stage 1 tests, patients eligible for randomisation (pulmicc stage 2) were offered an 'Accept/Decline' Questionnaire to complete following their decision to either proceed to randomisation or decline pulmicc stage 2. This 16 item, Likert scale, self-report questionnaire explores aspects of trial information provision, patients' concerns about their illness, influence of friends, family and doctor, and concerns regarding randomisation (V Jenkins, L Fallowfield, 2000). It enables the collection of patients'views on key issues surrounding trial information provision and decision-making in a structured, quantitative manner. Patients also identify their most important reason for accepting or declining study participation. The questions are worded generically to enable widespread use in randomised trials.

**Result**

Questionnaires were returned by 54 randomised patients and 57 who declined randomisation. The majority 106/111 (95%) indicated that they had received sufficient written information about the study and 110/111 (99%) indicated that the doctor had told them what they needed to know about the trial. Of patients who agreed to randomisation, 43/54 (80%) thought the trial offered the best treatment available and 48/54 (89%) were satisfied that either treatment in the trial would be suitable for them. Twenty five patients (44%) who declined randomisation were satisfied that either treatment in the trial would be suitable for them but 40/57 (70%) wanted the doctor to choose their treatment rather than be randomised by a computer. The results did not highlight significant problems such as patients feeling unable to say 'no' or concerns that their illness might get worse unless they joined the study. We have been able to use the information, together with clinicians' views on their experiences of the feasibility phase of the trial, to identify potential barriers to recruitment and enable strategies to be put in place to address these. Conclusion: We found the questionnaire easy to administer and acceptable to both patients who declined or agreed to join pulmicc stage 2. It is an efficient tool for collecting relevant views from patients regarding potential drivers and barriers to recruitment.

### P62

#### Perceptions of the utility and barriers of using electronic health records for patient screening in clinical trials: an in-depth interviews study of site coordinators

Emily O'Brien[1], Diane Bloom[1], Carol Koro[2], Jamie Lorimer[3], Sudha R. Raman[1], Robert J. Mentz[1], Bradley G. Hammill[1], Lisa G. Berdan[1], Ty Rorick[1], Salim Janmohamed[3]
[1]Duke University; [2]Glaxosmithkline & Merck & Co.; [3]Glaxosmithkline
**Correspondence:** Emily O'Brien
*Trials* 2017, **18(Suppl 1):**P62

**Background**

The Electronic Health Record (EHR) is a rich source of clinical data that holds promise for improving clinical trial conduct. However, little information is available on site-level barriers to optimal use of EHR systems in contemporary trials, particularly with respect to screening and enrollment. More data is needed on the current use and associated challenges of using the EHR to identify trial participants.

**Objective**

We described existing site-level processes for using the EHR for screening and recruitment of potential participants for an ongoing clinical trial. We also ascertained information on successful recruitment strategies and key barriers to using the EHR for trial recruitment from the perspective of site coordinators.

**Methods**

Qualitative focus groups were conducted with 18 study coordinators and site investigators at sites actively participating in the global multicenter HARMONY Outcomes trial, an ongoing randomized controlled study to evaluate the effect of albiglutide on cardiovascular events in patients with Type 2 diabetes (Clinicaltrials.gov ID: NCT02465515). Interviews were conducted by a professional moderator using a semi-structured, open-ended topic guide and were analyzed to identify common cross-site themes. Focus group participants represented research sites in the United States (n = 14), the United Kingdom (n = 2), Canada (n = 1), and Denmark (n = 1), with the majority based in multi-physician or hospital-based practices.

**Results**

Most focus group participants reported that the EHR was the primary modality used for screening, with the application of study-specific EHR queries in conjunction with medical chart review to generate a list of potentially eligible patients. In addition to EHR-based screening, most site coordinators reported using a multipronged approach of high- and low-yield trial recruitment strategies, including asking non-study investigators at the site to refer potentially eligible participants, posting fliers in clinics, sending mass mailings, and consulting lists of names of past participants for future studies. Several key barriers to use of the EHR system for recruitment were reported, including limitations on accessing individual patient records without informed consent, access to billing-only modules rather than research modules, limitations on the number of search parameters, and site-level restrictions on cold-calling patients meeting study criteria. Coordinators reported that, despite these barriers, using an EHR system has dramatically improved the perceived yield and timeframe relative to traditional, paper-based screening methods.

**Conclusions**

The majority of study coordinators in an ongoing diabetes trial reported that the EHR was the primary modality used to identify potential trial participants. Key barriers to full use of the EHR for recruitment included limitations on access to medical records and lack of research modules designed to support screening. Despite these barriers, the use of EHR systems for screening is viewed as an improvement over non-EHR-based methods.

### P63

#### The importance of informational items in a randomised controlled trial participant information leaflet: a mixed method study

Karen Innes, Katie Gillies, Seonaidh Cotton
Marion Campbell Health Services Research Unit, University of Aberdeen, Aberdeen, UK
**Correspondence:** Karen Innes
*Trials* 2017, **18(Suppl 1):**P63

A Patient Information Leaflet (PIL) is a requirement for UK health-related research studies. Health Research Authority (HRA) guidance lists 36 topic areas for inclusion in a PIL. However, there is limited evidence about whether stakeholders believe these items to be of importance when considering participation in a Randomised Controlled Trial (RCT). This study identified and assessed which items of information trial stakeholders ranked as most important and the reasons for this.

Our mixed method study used aspects of Q-methodology (a card sort technique) and simultaneous cognitive interviews (think aloud). This mixed methods approach captures data on subjective opinions held around a particular area of interest. The card sort technique provides participants with a set of "cards" (statements describing specific information items) which they rank according to their opinion of relative importance. A specially formatted grid is used to capture the relative rankings. While the participant completes the card sort, they are encouraged to use the think-aloud technique to verbalise their thoughts.

In this study, the statements included on the cards relates to the information items recommended by HRA for inclusion in a PIL. Twenty trial stakeholders were recruited (10 potential trial participants [PTPs] and 10 research nurses [RNs]) completed the card sort within one-to-one think-aloud interviews. To contextualise the card sort, PTPs were asked to imagine they had been approached to participate in a phase III RCT comparing treatments A and B for a chronic condition. RNs were asked to think about potential participants making the decision to take part in the same phase III RCT.

Both stakeholder groups ranked the following three statements in their top four most important statements: "possible disadvantages and risks of taking part", "possible advantages of taking part" and "possible side effects of trial treatment". Both stakeholder groups ranked "who is funding the research" among their least important statements. There were differences between groups in the other statements ranked as least important. RNs included "who has approved the study", "how have patients and the public been involved in the design of the study" and "has the scientific quality of the study been checked" among their least important statements. PTPs ranked "will expenses be reimbursed", "will there be any impact on any insurance policies" and "will I receive any payments for taking part" among their least important statements.

This study is one of the first to explore how different stakeholder groups rank the information contained in an RCT PIL. Similarities exist between both stakeholder groups in statements ranked as most important, but there are differences in the least important statements. These results have implications for researchers developing PILs for RCTs. Further research is required to identify any association between the information provided in PILs and the decision-making process around RCT participation.

### P64

#### Recruitment and retention of participants in randomised controlled trials: a review of trials funded by the United Kingdom health technology assessment programme

Stephen Walters, Inês Bonacho dos Anjos Henriques-Cadby, Oscar Bortolami, Laura Flight, Daniel Hind, Richard M. Jacques, Christopher Knox, Nadin, Joanne Rothwell, Michael Surtees
University of Sheffield
**Correspondence:** Stephen Walters
*Trials* 2017, **18(Suppl 1):**P64

**Background**

Substantial amounts of public funds are invested in health research worldwide. Publicly funded randomised controlled trials (RCTs) often recruit participants at a slower than anticipated rate. Many trials fail to reach their planned sample size within the envisaged trial timescale and trial funding envelope. A recent survey amongst the Directors of the Clinical Trials Units registered with the UK NIHR Clinical Research Network identified priorities for research into the methodology of trials. The top three priorities were improving recruitment, choice of outcomes, and improving retention.

**Objectives**

To review the consent, recruitment and retention rates for single and multi-centre randomised control trials funded by the United Kingdom's National Institute for Health Research (NIHR) Health Technology Assessment (HTA) Programme.

**Methods**

HTA reports of individually randomised single or multi-centre rcts published from the start of 2004 to the end of April 2016 were reviewed.

Data extraction Information was extracted, relating to the trial characteristics, sample size, recruitment and retention by two independent reviewers.

Main outcome measures Target sample size and whether it was achieved; recruitment rates (number of participants recruited per centre per month) and retention rates (randomised participants retained and assessed with valid primary outcome data).

**Results**

This review identified 151 individually randomised controlled trials from 778 NIHR HTA reports. The final recruitment target sample size was achieved in 56% (85/151) of the RCTs and more than 80% of the final target sample size was achieved for 79% of the RCTs (119/151). For 34% (52/151) of trials the original sample size target was revised (downward in 79% (41/52)). The median recruitment rate (participants per centre per month) was found to be 0.92 (IQR 0.43 to 2.79) and the median retention rate (proportion of participants with valid primary outcome data at follow-up) was estimated at 89% (IQR 79% to 97%).

**Conclusions**

Based on this review for most publicly funded trials the recruitment rate is likely to be between 1 and 2 participants per centre per week (4 to 10 a month). There is considerable variation in the consent, recruitment and retention rates in publicly funded RCTs. In practice, recruitment rates will vary, depending on whether the target population is acute, where opportunistic recruitment will target incident cases, or chronic, where database recruitment can effectively target prevalent cases. It will also vary according to whether the intervention is therapeutic or preventive and the base incidence and prevalence rate of the condition. Investigators should bear this in mind at the planning stage of their study and not be overly optimistic about their recruitment projections.

### P65

#### Do higher monetary incentives improve response rates part-way through a randomised control trial?

Jessica Wood[1], Jonathan A. Cook[2], Jemma Hudson[1], Alison McDonald[1], Hanne Bruhn[3], Angus J. M. Watson[4]

[1]Centre for Healthcare Randomised Trials (chart), Health Services Research Unit, University of Aberdeen; [2]Nuffield Department of Orthopaedics, Rheumatology & Musculoskeletal Sciences, University of Oxford; [3]Health Economics Research Unit, University of Aberdeen; [4]Department of Surgery, NHS Highland

**Correspondence:** Jessica Wood

*Trials* 2017, **18(Suppl 1):**P65

**Background**

During routine monitoring in the ethos study (a surgical trial comparing Stapled Haemorrhoidopexy with Traditional Haemorrhoidectomy for the treatment of grade II-IV haemorrhoids), it was found that the response rates to the 12 and 24 month follow-up postal questionnaires were lower than expected. Literature reviews looking at methods to increase response rates identified monetary incentives as one potential way to boost response rates1-2. Two Studies With-in a Trial (swats) were conducted within ethos to assess the effectiveness of a small unconditional voucher and a higher value conditional voucher on response rates to the postal questionnaires. Following no effect of a lower value voucher incentive (£5.00) being found in increasing response rates in the study (SWAT1), the team designed an additional study to evaluate if a higher value monetary incentive would be more effective in increasing questionnaire response rates.

**Methods**

Participants enrolled in ethos who had not yet received their 12 and/or 24 months follow-up questionnaires were included in SWAT2. All participants were sent a covering letter with their postal questionnaires which informed them that they would receive a £30 high street voucher as a token of appreciation upon receipt of a completed questionnaire. The primary analysis was a before and after analysis of the effect of the voucher in increasing response rates at each time-point. A sensitivity analysis was also carried out due to the overlapping influence of SWAT 1.

**Results**

In total 586 and 562 participants were included in the 12 and 24 month analyses respectively in SWAT2. Results showed no statistical evidence of an effect on the response rates at both 12 and 24 month time-points. Similarly, the sensitivity analyses results showed no evidence of a difference in the 12 month response rates after the incentive was given. At 24 months there was a slight increase in response rates (before 71.4%, after 75.9%) but it was not statistically significant, 95% CI [0.87,1.80].

Discussion

Both studies highlight that, despite current literature to the contrary, the use of monetary incentives may not increase questionnaire response rates in all study populations and could even have a negative impact. There are a number of contextual aspects which may explain this unexpected finding. Care is needed when introducing a new intervention into an ongoing trial. Future evaluations of incentives are needed to explore the impact of contextual issues which may moderate their impact and influence in different study settings.

### P66

#### Recruitment aids for a phase II randomised trial in low risk bladder cancer

Steven Penegar[1], Rebecca Lewis[1], James Catto[2], Joanne Cresswell[3], Leyshon Griffiths[4], Micki Hill[1], John Kelly[5], Allen Knight[6], John McGrath[7], Laura Wiley[1]

[1]The Institute of Cancer Research; [2]University of Sheffield; [3]South Tees Hospitals NHS Foundation Trust; [4]University Hospitals of Leicester NHS Trust; [5]University College London; [6]Patient representative; [7]Royal Devon & Exeter NHS Foundation Trust

**Correspondence:** Steven Penegar

*Trials* 2017, **18(Suppl 1):**P66

**Background**

Non-muscle invasive bladder cancer (NMIBC) is a locally recurring disease for which patients undergo long term surveillance following initial diagnosis. CALIBER is a multicentre phase II feasibility study comparing intravesical chemotherapy (chemoresection) with surgery (standard of care) in patients with recurrent low risk NMIBC (2:1 chemoresection:surgery randomisation). The primary aim is to assess complete response to chemoresection and the trial is randomised to test feasibility of recruitment to a larger randomised phase III trial.

It was anticipated that patient recruitment would be challenging due to the need to identify potential participants at the time of recurrence prior to treatment, complex risk stratification criteria and varied treatment pathways across participating sites. As such we developed recruitment aids with the aim of raising awareness amongst potential participants, ensuring site staff remain aware of the trial and promoting effective liaison between site staff when suitable patients are identified.

**Methods**

From the outset of the trial, ethics approved short patient information leaflets and posters have been available to highlight the trial to patients attending surveillance visits. A staff poster was also provided to raise awareness amongst staff conducting surveillance. A CALIBER specific risk calculation tool was introduced in March 2016 as an aid to assess eligibility. We surveyed 34 participating centres about their use of these aids and their use of the tools was compared to their average recruitment.

**Results**

Responses were received from 26/34 centres. 25/26 (96%) are using at least one of the short patient information leaflet, patient poster, clinician poster or eligibility. Average monthly recruitment does not appear to increase with increased use of the tools, with a median recruitment of 0.21 for the 8/26 (31%) sites using two tools and 0.03 for the 6/26 (23%) sites using all four.

Since distributing the CALIBER risk calculator, the number of eligibility queries received by the coordinating clinical trials unit has substantially decreased. Initial feedback from centres suggests it is a useful tool for local pre-screening. Centres are advised to print the completed score calculation and retain in the patient notes to document this eligibility assessment.

#### Limitations

The impact of introduction of different tools on recruitment could not be confirmed as most have been available since the trial commenced. The reduction in eligibility queries since introduction of the recurrence calculation tool may be a result of increased centre experience. In addition, the use of tools may be confounded with factors such as centre size and frequency of patient screening for the trial.

#### Conclusions

With provision of targeted recruitment aids, centre staff training and ongoing support from the coordinating clinical trials unit, potential barriers to recruitment in a trial with challenging patient identification pathways and complex eligibility criteria can be managed effectively. However there is no obvious increase in recruitment with increased use of recruitment aids. In order to robustly evaluate the impact of recruitment aid interventions they should be introduced in a controlled manner to facilitate assessment of within and between centre pre- and post- intervention accrual rates.

### P67

### Research site mentoring: a novel approach to improving study recruitment

Marcus Johnson, Margaret Antonelli, Lynn Tommessilli, Beata Planeta
US Department of Veterans Affairs
**Correspondence:** Marcus Johnson
*Trials* 2017, **18(Suppl 1):**P67

#### Background

The VA Cooperative Studies Program's (CSP)1 Network of Dedicated Enrollment Sites (NODES) is a consortium of 9 VA medical centers (VAMCs) that have teams (Nodes) in place dedicated to conducting CSP studies to enhance the overall performance, compliance, and management of CSP multi-site clinical trials. Each Node site has a Director (MD/PhD), Manager (Clinical Trial Nurse, Research Project Manager), and Research Assistant(s).

CSP NODES piloted a "mentoring" (or hub-spoke) model in which a Node site would more directly work with a study site to identify and overcome barriers to recruitment, compliance, and data quality.

#### Aims

1. Determine the impact of an external research site mentoring model on study recruitment. 2. Examine the study site-level characteristics that facilitate or impede study recruitment.

#### Methods

The Colonoscopy Versus Fecal Immunochemical Test in Reducing Mortality From Colorectal Cancer (CONFIRM) (CSP #577)3 study is a large, simple, multicenter, randomized, parallel group trial directly comparing screening colonoscopy with annual FIT screening in average risk individuals. Each of the 9 Node sites was paired with a low performing (recruitment) CSP #577 study site. One Node site was assigned two low recruiting sites for a total of 10 pilot sites. The respective Node Manager then worked with the study site and the West Haven CSP Coordinating Center (WHCSPCC) 4 to perform the following:

Created a site management checklist to determine the current state of local study operations; Used the site management checklist to conduct interviews with site study staff; Used the feedback that was gathered during the site interviews to create study improvement plans that contained performance metrics to measure criteria related to recruitment, compliance, and data quality; Held regular conference calls independently with study sites and WHCSPCC to monitor progress. The pilot was conducted over a 6-month period from February 2016-June 2016.

#### Results

The ten Study sites that participated in the pilot mentorship had an average improvement of 4.9 participants enrolled per month vs. An average improvement of 1.3 participants enrolled per month at the 27 study sites that were not part of the pilot. Some common issues/barriers to recruitment that the pilot sites faced are as follows: lack of recruitment at community-based outpatient clinics, lack of utilization of the full spectrum of recruitment materials e.g. Letters, flyers, participant screening algorithms (electronic medical records), etc., unmotivated/disengaged study staff, lack of clinical referrals, and uneven distribution of duties across study teams. Having a subject matter expert that was external to the CSP coordinating center and could serve as a mentor was beneficial for the pilot sites. The pilot provided a resource to the site that worked within a similar environment and could provide specific, site-level guidance on how to resolve some of the recruitment issues/barriers that they faced.

#### Conclusion

The site mentoring model was successful in increasing participant recruitment at study sites in a large, simple, multicenter, randomized, parallel group trial in the VA healthcare system.

### P68

### A cross-cutting approach to clinical trial success: the US Department of Veterans Affairs' network of dedicated enrollment sites (NODES) model

Marcus Johnson[1], Aliya Asghar[2], Tawni Kenworthy-Heinige[3], Debra Condon[4], Karen Bratcher[5], Meghan O'Leary[6], Danielle Beck[7], Cyenthia Willis[8], Grant D. Huang[9]
[1]US Department of Veterans Affairs; [2]VA Long Beach Healthcare System, US Department of Veterans Affairs; [3]VA Portland Health Care System; [4]Minneapolis VA Health Care System; [5]VA Palo Alto Health Care System; [6]Durham VA Medical Center, US Department of Veterans Affairs; [7]VA San Diego Healthcare System, US Department of Veterans Affairs; [8]VA North Texas Healthcare System, US Department of Veterans Affairs; [9]VA Cooperative Studies Program Central Office, US Department of Veterans Affairs
**Correspondence:** Marcus Johnson
*Trials* 2017, **18(Suppl 1):**P68

#### Background

Clinical trials are a critical component of biomedical research and provide valuable insights into effective means for enhancing patient care and establishing new therapies. Recruitment into clinical trials remains a key determinant to study completion and success. Barriers to achieving enrollment targets include distrust of the medical community and clinical research, lack of awareness or understanding about clinical trials and eligibility criteria, and concerns about the logistics of participation, such as required travel, the time involved with participating, and potential costs. While various strategies have been proposed, it is unclear how broadly they apply when different populations, diseases, and/or study goals are involved. The ability to effectively overcome challenges may require approaches that focus more on addressing shared interests among sites in overcoming clinical trial barriers.

#### Methods

The Department of Veterans Affairs (VA) Cooperative Studies Program (CSP) is a clinical research infrastructure embedded within the nation's largest integrated health care system. The VA Network of Dedicated Enrollment Sites (NODES) is a consortium of nine sites intended to provide systematic site-level solutions to issues that arise during the conduct of VA CSP clinical research [1]. Each NODES site is represented at each VA Medical Center (VAMC) by a Director (or team of co-directors/associate directors) and a Manager. Additionally, each site has clinical support staff, including nurses and research assistants, designated to assist multiple CSP clinical trials locally. Within the context of a large, integrated health care system, NODES goals are to: 1) enhance recruitment for clinical trials; 2) create study efficiencies; 3) improve communication and disseminate best practices; and 4) provide broader expertise in the design and conduct of VA clinical research. Initial pilot activities were conducted for establishing more cross-cutting approaches to improving recruitment.

#### Results

NODES addressed key barriers affecting clinical trial outcomes at study-specific and organizational levels. Results of these activities are presented in categories related to 1) implementing innovative participant recruitment strategies, 2) creating site-level efficiencies for study operations and management, and 3) establishing metrics to more effectively evaluate site and network performance. Initial network efforts produced several lessons and best practices for common clinical trial problems. Additionally, innovations for wider adoption across CSP studies were developed. Such strategies include mobile recruitment, algorithmic inclusion/exclusion data programs for recruitment activities, staff cross-training and mentorship, and standardized performance reporting. Some metrics were also used for overall network performance.

**Conclusion**

NODES addressed barriers in various aspects of clinical trial recruitment and management by working collaboratively to solve problems with multiple stakeholders. Varied practices and operational changes in CSP research related to recruitment, staff training and research methodology were implemented by taking an overall, system wide approach. Many challenges with patient recruitment experienced within CSP are similar to those encountered by other multi-site government or private industry clinical trials. As a result, the solutions to these recruitment problems presented by NODES may be transferable to other healthcare settings.

### P69

#### No common denominator: a review of outcome measures in IVF RCTs

Jack Wilkinson[1], Stephen A Roberts[1], Marian Showell[2], Daniel R Brison[3], Andy Vail[1]
[1]University of Manchester; [2]University of Auckland; [3]Central Manchester University NHS Foundation Trust
**Correspondence:** Jack Wilkinson
*Trials* 2017, **18(Suppl 1):**P69

This abstract is not included here as it has already been published.

### P70

#### Small sample corrections for cluster randomised trials: implications for power and type I error rate

Clemence Leyrat[1], Katy Morgan[1], Baptiste Leurent[1], Brennan Kahan[2]
[1]London School of Hygiene and Tropical Medicine; [2]Queen Mary University of London
**Correspondence:** Clemence Leyrat
*Trials* 2017, **18(Suppl 1):**P70

**Background**

Cluster randomised trials (CRTs) are increasingly implemented to assess the effectiveness of interventions in settings in which individual randomisation is impossible or challenging. Three main analysis strategies have been proposed to analyse CRTs: cluster-level analysis, mixed-models and generalised estimating equations (GEE). Whereas the former approach maintains the nominal type I error rate, that is, the chance to detect an effect when there is not, the last two lead to inflated type I error rates when the number of clusters is small or the cluster size varies. Small sample corrections have been proposed for mixed models and GEE to circumvent this problem, but the impact of these methods on power is still unclear.

**Methods**

We performed a simulation study to assess both the type I error rate and the power of parallel two-arm CRTs with a continuous outcome analysed with cluster-level methods, mixed models or GEE. For cluster-level analysis, we studied the performance of a linear model of cluster means without correction, a linear model weighted by the cluster size or weighted by the variance components and a Wilcoxon test. For mixed models, we assessed the performance of a Z-test, as well as three degree of freedom corrections: Satterthwaite, Kenward-Roger and the between-within method. Finally, for GEE, we compared the performance of a Z test using model-based and robust standard errors and a small sample correction proposed by Fay and Graubard. We studied the impact of varying the intraclass correlation coefficient (ICC), the number of clusters randomised and the variability in cluster size.

**Results**

The results confirmed that when only few clusters are randomised, inflated type I errors are observed and this inflation increases with the ICC and with the variability in cluster size. Amongst the compared methods, only the cluster-level model weighted on the variance components and mixed models with Satterthwaite or Kenward-Roger corrections maintained the type I error rate at or below 5% in all scenarios. Second, in terms of power, individual-level analyses outperformed cluster-level analyses, but the power remained low for fewer than 20 clusters randomised. Moreover, when the number of clusters was very small (<8), the degree of freedom corrections lead to very low type I errors (<2%), thus reducing the power to between 30 and 50%.

**Conclusions**

When the number of randomised clusters is small, appropriate corrections must be used to maintain an appropriate type I error rate. GEE approaches are not recommended, but a weighted cluster-level analysis or a mixed model with a Satterthwaite or Kenward-Roger degree of freedom correction can maintain an appropriate type I error. However, these approaches lead to an important decrease in power when fewer than 20 clusters are randomised, so adjustment of the sample size is required at the design stage when such corrections are used.

### P71

#### The Glasgow deep end links worker programme evaluation: analysis implication of an unusual randomised study design

Andisheh Bakhshi, Alex McConnachie, Bridie Fitzpatrick, Kate O'Donnell, Mhairi McKenzie, Kathyrn Skivington, Nai Chng, Mathew Smith, Sally Wyke, Stewart Mercer
University of Glasgow
**Correspondence:** Andisheh Bakhshi
*Trials* 2017, **18(Suppl 1):**P71

**Background**

Tackling health inequalities has been a Scottish Government priority since 2007. General Practitioners (GPs) at the Deep End is a collaboration of general medical practices serving the 100 most deprived populations in Scotland, based on the proportion of patients on the practice list with postcodes in the most deprived 15% of Scottish data zones. Eighty-six of these 100 practices are in the NHS Greater Glasgow and Clyde Health Board area. The Deep End Links Worker Programme was designed to prevent and reduce health inequalities in Scotland, and support people living in the most deprived areas of Scotland to 'live well' In their communities. It provides resources to General Practices serving these populations to develop a 'links approach'. Practices can refer patients to a Links Worker, who works with the patient to identify and enable access to appropriate community-based resources. The argument is that increasing and enabling local activities can enhance community connection, trust and cohesion. Hence, the aim of Links Worker is to act as catalyst to hope and self-determination, by using the strong relationship with patients that exists in General Practices as a natural community hub. Part of the evaluation of the programme involved a comparison of patient and staff outcomes measured in 7 Intervention practices, with those from 8 Comparator practices. These 15 practices were allocated to the Intervention and Comparison arms at random, prior to the involvement of the evaluation team.

**Objective**

The primary aim of the evaluation was to assess the effectiveness of the Links Worker Programme in achieving the intended outcomes at patient, practice and community levels.

**Methods**

A quasi-experimental evaluation design was decided by the Scottish government as part of their funding conditions. Among 15 General Practices that submitted a formal expression of interest in having a Links Worker attached to their team, patients' outcomes in 7 Generals Practices with an attached Links Worker will be compared with those from 8 General Practices that do not have an attached Links Worker. The intervention group are patients who were referred to the Links Worker, but the comparator group are a random sample of the practice population. By design, the intervention group is very different from the comparator group. Therefore, simple between-group comparisons, which would be appropriate for most randomised studies, will not be appropriate. The main statistical analyses have been defined in terms of mixed effects regression models, to account for clustering of outcomes at the practice level, with adjustment for differences in baseline covariates. In addition, other methods will be explored, aiming to control for the selection bias inherent in the study design (e.g. With respect to age, gender, deprivation and comorbidities), such as nearest neighbour matching and propensity score analysis.

**Results**

The final analysis is underway. Some results of the evaluation will be presented, with particular focus on the implications of this unusual design, and the results obtained using alternative analysis approaches.

### P72

#### Group sequential methods for monitoring multi-arm clinical trials

Danni Liu[1], Michele Melia[1], for the Diabetic Retinopathy Clinical Research Network[2]
[1]Jaeb Center for Health Research; [2]DRCR.net
**Correspondence:** Danni Liu
*Trials* 2017, **18(Suppl 1):**P72

In comparison with conventional two-arm clinical trials, multi-arm clinical trials provide investigators with the ability to directly compare more than two competing treatments in a single trial setting. The multi-arm trial design optimizes the efficiency of a trial, and even reduces the required total sample size if all experimental treatments share a single control arm. It has been shown that a multi-arm trial design can have economic and ethical benefits, such that it allows for more efficient resource allocation and cost savings compared with two separate trials, as well as the possibility of dropping an inferior experimental treatment in the interim or even stopping the study early if there is strong evidence for efficacy or futility.
It is common in multi-arm trials that researchers are more interested in simultaneously monitoring all pairwise comparisons than just a global test. Given that the probability of falsely rejecting the null hypothesis is inflated when multiple pairwise hypothesis tests are performed repeatedly in a single study, several multiple comparison procedures have been generalized to the context of group sequential testing to preserve the experiment-wise type I error rate, even when an inferior treatment arm is dropped during interim monitoring. Some examples are the generalization of the simple Bonferroni procedure, the Pocock's procedure, the O'Brien-Fleming procedure, the Fisher's Least Significant Difference method, and the Newman-Keuls procedure.
In this presentation, we will discuss group sequential methods for monitoring clinical trials with multiple treatment groups. Two recently completed Phase III multi-center randomized clinical trials evaluating treatments for diabetic macular edema conducted by the Diabetic Retinopathy Clinical Research Network (DRCR.net) will be used as examples. We will apply several group sequential methods to these two fixed-sample studies and demonstrate the design and conduct of statistical interim monitoring for efficacy under two distinct scenarios: comparisons with a control group and all pairwise comparisons.

### P73

#### Calibrated phase II oncology design in a bayesian setting

Jessica Kendall, Duncan Wilson, Sarah Brown, Andrew Hall
University of Leeds
**Correspondence:** Jessica Kendall
*Trials* 2017, **18(Suppl 1):**P73

Phase II oncology designs commonly seek to assess the potential efficacy of a treatment. Single-arm trials can be utilised comparing against a historic control, with decision-making cut points indicating whether the treatment warrants further investigation. A limitation of single-arm designs is that the results of the trial may be unreliable predominantly due to selection bias. Therapeutic benefits may generally be smaller than differences in outcome due to baseline characteristics. Detailed description of the baseline prognostic factors is of little help in determining for example whether a group of patients with poor prognosis has been recruited into the trial or whether the treatment is not truly worthy of further study. Buyse (2000) argues that randomisation should be considered more often for early trials of experimental treatments.
The calibrated design (Herson & Carter 1986) is a randomised single-stage design utilising a binary endpoint. The design differs from the typical randomised trial, as the "control" Group is not employed to provide a comparison for the experimental treatment, but to evaluate whether the sample population who receive the experimental treatment has the capability of showing a response. The design essentially constitutes two separate designs; one for the experimental arm and one for the calibration arm. The experimental arm is set up using a single-stage design using an exact binomial distribution (A'Hern 2001) with corresponding "recommend" and "abandon" cut points specified against a historic control. Outcomes in the calibration arm are used to assess whether the assumptions for the historic control are acceptable and whether the response rate lies within the expected range. If the response rate is not comparable, the conclusions from the experimental arm are questioned.
The Myeloma UK Clinical Trials Network developed the muktwelve trial; a randomised phase II study to assess the progression-free survival of a potential treatment regimen in relapsed and refractory multiple myeloma. A calibration group will receive a control treatment, and will be used to evaluate the validity of the historic control used in the experimental group. Sixty patients are randomised on a 3:1 basis to experimental and control arms respectively. The sample size was based primarily upon the experimental arm. Due to pragmatic constraints, there is insufficient power to conduct formal hypothesis testing in the calibration arm. However the inclusion of this arm is necessary to safeguard against selection bias and give context for the historic control rate.
We review the use of a calibration arm in phase II oncology trials and the reasons for implementing such an approach, with an application to the muktwelve study. We also assess the possibility of designing the trial in a Bayesian setting, with the intention of more formally using the calibration arm. It is postulated that the uncertainty around the historic control rate can be better modelled by using this approach. One possibility would be to adapt the use of commensurate priors, which can be utilized in a two-arm setting with a formal comparison to assess historic and concurrent heterogeneity (Hobbs, Carlin & Sargent 2013).

### P74

#### Sample size re-estimation in cross-over trials: a simulation study investigating the type I error, power and sample size in the AIM HY-INFORM study

Julie Wych, Adrian Mander
University of Cambridge
**Correspondence:** Julie Wych
*Trials* 2017, **18(Suppl 1):**P74

Hypertension is the single biggest contributor to the global burden of disease in the UK. It is widely accepted that ethnicity is one factor that is associated with hypertension and which influences the response to existing first-line antihypertensive treatments. In the UK hypertension treatment is stratified by age and self-defined ethnicity (SDE), problems associated with this include a lack of data from UK populations supporting the current SDE stratification and no reference to South Asians – the largest ethnic minority group in the UK. The primary objective of the AIM HY-INFORM study is to determine if the response to existing first-line anti-hypertensive drugs differs by ethnic group for patients on mono- or dual therapies. The work presented here will consider patients on a monotherapy treatment regime. The study design is a 3-period 3-treatment cross-over trial in a multi-ethnic cohort of hypertensives using a linear mixed effects model with subject as a random effect. With the absence of prior estimates of the within-subject SD, one problem with this multilevel design is the calculation of the required sample size to ensure the desired power to detect a single ethnic by treatment interaction. Sample-size re-estimation designs can be used in this context to change the sample size according to accrued information in a statistically robust way. Ahead of trial recruitment, a simulation study was carried out with the main aims of (i) Assessing the properties of the hypothesis tests for the sample size defined in the AIM HY-INFORM study protocol; (ii) Estimating the fixed (single ethnic by treatment interaction) and random (within-subject SD) estimates of interest along with their summary and performance measures; (iii) Simulating an interim analysis after 50 subjects have completed their treatment sequence to re-assess the sample size calculation. Results and performance measures will show that the hypothesis-generating procedure attains a size of 0.05 for 1000 simulations with a protocol sample size of 660 subjects for different within and between-subject SDs. The estimated power is in line with that achieved in the study protocol and an underestimated assumed within-subject SD requires a larger than planned study sample size; providing a means of preserving the power of the study, a distinct advantage over a fixed design.

### P75

#### Methodological challenges in the react study, a randomized controlled trial of real time continuous glucose monitoring in neonatal intensive care

Julia Forman[1], Simon J. Bond[2], Catherine Guy[3], David Dunger[3], Kathryn Beardsall[3]
[1]University of Cambridge and Cambridge Clinical Trials Unit, Addenbrooke's Hospital; [2]Cambridge Clinical Trials Unit; [3]University of Cambridge and Cambridge University Hospitals NHS Foundation Trust
*Trials* 2017, **18(Suppl 1):**P75

**Background**
In utero, glucose levels are normally maintained between 4–6 mmol/L. Infants born preterm are at risk of both hyperglycaemia (20-86%) and hypoglycaemia (17%), both of which have been associated with increased mortality and morbidity. In neonatal intensive care, clinical practice relies on intermittent blood glucose monitoring. The REACT Trial aims to evaluate the role of real time continuous glucose monitoring (CGM) in the detection and management of hyperglycaemia and hypoglycaemia in these babies. REACT will thus provide a wealth of information, combining both continuous and clinical data, and present challenges in statistical analyses regarding how to optimally interpret and use these results.
**Trial design**
REACT is an international multicentre randomised controlled trial and will recruit 200 subjects within 24 hours of birth, with a birth weight <1200 g. Subjects will be randomized equally to two parallel arms. All babies will have an Enlite sensor (Medtronic, Northridge, CA, USA) inserted and linked to a Medtronic Minimed 640G monitor for data collection. In the intervention arm the real time sensor data will be used for clinical management. In the control arm the data will be collected blind to the clinical team caring for the baby. In both study arms the data will be recorded every five minutes for six days. The primary endpoint for the trial is the difference between the arms in the percentage of time sensor glucose is in the target range of 2.6-10 mmol/l within the first six days of life.
**Methodological challenges**
Evaluating continuous data, upon which dynamic treatment decisions are made, presents challenges for analysis. The primary analysis has a clear interpretation, but it ignores the time dependence of the data, and dichotomises the results: within/outside the target range. Therefore it cannot detect any time-dependent treatment effects. Furthermore, as with all dichotomisations, it is unduly influenced by the choice of boundaries that define the target range. There is a paucity of alternatives that correct both of these issues, and offer a clinically valuable interpretation.
We propose to compare the result of the primary analysis with alternative methodologies, including time series analyses, which treat the data continuously and incorporate the time-dependent correlation between measurements. In addition, we are interested in determining if the CGM data and clinical data on subject treatment can be used to further optimize the current clinical algorithm and improve glucose control.
**Trial registration and funding**
This trial is registered with the ISRCTN (number 12793535) and funded by the NIHR Efficacy and Mechanism Evaluation Programme.
The Efficacy and Mechanism Evaluation programme is funded by the MRC and NIHR, with contributions from the CSO in Scotland, NISCHR in Wales and the HSC R&D, Public Health Agency in Northern Ireland. This report is managed by the NIHR Evaluation, Trials and Studies Coordinating Centre (NETSCC) (Efficacy and Mechanism Evaluation, 11/133/07 Real Time Continuous Glucose Monitoring in Neonatal Intensive care). The views expressed in this publication are those of the author(s) and not necessarily those of the MRC, NHS the National Institute for Health Research or the Department of Health.

### P77

#### A model-free Bayesian adaptive design for combination therapy dose-finding trials with censored time-to-toxicity data

Graham Wheeler[1], Michael J. Sweeting[2], Adrian P. Mander[3]
[1]University College London; [2]University of Cambridge; [3]MRC Biostatistics Unit Hub for Trials Methodology Research
**Correspondence:** Graham Wheeler
*Trials* 2017, **18(Suppl 1):**P77

Many strategies for treating cancers use combinations of drugs or multiple forms of treatment (e.g. Concurrent chemoradiotherapy). In most early phase trials of two therapies (labelled as A and B, say) a fixed dose of agent A is administered and only the dose of agent B is escalated. The aim is to identify a recommended phase II dose combination (RP2DC), i.e. The largest dose of agent B that, when combined with the fixed dose of agent A, has a chance of causing a dose-limiting toxicity (DLT) close to some predefined limit. Currently, more combination therapy trials allow both treatments to be escalated; patients entering the trial receive the best dose from a grid of possible combinations, and one or more RP2DCs can be considered for use in phase II. However, modelling the relationship between dose combination and toxicity can be challenging since most parametric models require many parameters to be flexible enough, and model assessment is impossible given the limited data available in phase I trials.
The product of independent beta probabilities escalation (PIPE) design, a model-free approach for identifying multiple RP2DCs in dual-agent phase I trials, is being used in ORCA-2, a phase I trial seeking optimum dose and schedule combinations of olaparib in patients with advanced head and neck cancer. However, the DLT observation period for each patient is 13 weeks, with up to 40 patients planned for a dose-escalation phase and another 10–30 patients planned for a dose-expansion cohort. In trials using radiotherapy, some toxicities can occur months after treatment, so long DLT observation periods are required to ensure dlts are accurately captured and future patients are not at risk of excessive toxicity. In both settings, it is impractical to wait until all current patients have completed follow-up before treating the next patient. Using data from patients that have partially and fully completed DLT follow-up at the current time could potentially reduce study duration and costs, particularly for trials of combination therapies where sample sizes should be larger than single-agent trials and recruitment is faster than expected.
We therefore propose an extension to the PIPE design that uses censored toxicity and complete follow-up outcomes to assist with dose-escalation decisions. We show how different recruitment rates affect both trial conduct and results relative to a trial that requires complete patient follow-up. We consider scenarios where time-to-toxicity is distributed earlier, uniformly, or later in the DLT observation window and use an adaptive weighting procedure that alters the amount of information each patient provides based on previous DLT times. In the context of the ORCA-2 trial, we obtain substantial reductions in trial duration, with comparable experimentation and recommendation properties to the planned design.

### P78

#### A two-stage, phase II clinical trial design with nested criteria for early stopping and efficacy

Michelle DeVeaux, Michael J. Kane, Daniel Zelterman
Yale School of Epidemiology and Public Health
*Trials* 2017, **18(Suppl 1):**P78

We propose a two-stage design for a single arm clinical trial with an early stopping rule for safety. This design employs different criteria to assess early stopping and efficacy. The early stopping rule is based on a criteria that can be determined more quickly than that for efficacy. These separate criteria are also nested in the sense that efficacy is a special case of, but usually not identical to, the early stopping criteria. The addition of a curtailed sampling scheme allows for early decisions to be made before all of the data has been observed. The design readily allows for planning in terms of statistical significance, power, and expected sample size. This method is illustrated with a Phase II design comparing patients treated for lung cancer with a novel drug combination to a historical control. In this example, the early stopping rule is based on the number of patients who exhibit progression-free survival (PFS) at 2 months post treatment follow-up. Efficacy is judged by the number of patients who have PFS at 6 months.

### P79

#### Analysis challenges in blood stream infection: focus on outcomes study

Rebecca Evans[1], Katie Pike[1], Alasdair MacGowan[2], Chris A. Rogers[1]
[1]Clinical Trials and Evaluation Unit, University of Bristol; [2]Southmead Hospital, North Bristol NHS Trust
**Correspondence:** Rebecca Evans
*Trials* 2017, **18(Suppl 1):**P79

**Background**
Infection is a major concern in UK hospitals and it is estimated that up to 8% of inpatients have an infection. The death rate from these infections can reach 10-30% depending on the patient and pathogen. A number of non-modifiable patient factors (e.g. Comorbidities and infection severity) are known to impact adversely on outcome. However there are limited data available from large multicentre studies to investigate the importance of modifiable risk factors. 'Blood Stream Infection: Focus on outcomes' is a multicentre, prospective observational study with the primary aim of identifying modifiable risk factors associated with all-cause mortality in patients with one of six key pathogens. Modifiable data collected includes staffing levels, antibiotic use (in particular the timing of appropriate therapy) and use of intravenous lines.
**Dataset**
A total of 1,676 patients were included in the study across 5 centres. The dataset comprises 48 modifiable and 38 non-modifiable factors. The overall mortality rate is 20.8% (95% CI: 18.8% - 22.8%).
**Analysis approach**
Although the primary purpose of the study is to identify and estimate the effect of modifiable factors, the analyses need to take the non-modifiable risk factors into account. To maximise the degrees of freedom available, the analysis was split into two stages. For the first stage, a Cox model was fitted for the non-modifiable risk factors only with the aim of deriving a risk scorer which quantifies each patient's risk of mortality. The second stage of the modelling is investigating the modifiable risk factors and the risk score derived in the first stage is included as a covariate (analysis underway).
**Analysis challenges**
The first challenge encountered was missing data; data were missing for between 10% and 45% of patients for some of the key data items. Secondly, in order to ensure the calculated risk score provides the best summary of the relationship between the non-modifiable factors and survival, it was important to ensure the most suitable functional form of each continuous covariate was used. Finally, the modelling required a survival analysis framework, which included ensuring that the appropriate model assumptions were met.
To overcome these challenges multiple imputation using chained equations ("Ice" Command in Stata v14.0) was used to impute the missing data items. Subsequently multivariable fractional polynomial models were fitted on the imputed dataset ("Mfpmi" Command), within a Cox model ("Stcox" Command). This command performs backwards selection to select variables that are predictive of mortality, whilst finding the most suitable functional form of such covariates, within a time to event model. Finally, the proportional hazards assumption was checked in the final model, using standard methods.
Discussion
The use of multivariable fractional polynomials within survival analysis models using imputed data can be useful to find a best fitting model for a dataset with missing data. The code in order to achieve this is available in standard statistical packages, such as Stata. Fractional polynomials can be difficult for non-statisticians to grasp and may not be the best choice in settings where straightforward interpretability of coefficients is required.

### P80

#### An oxford knee score cut-off point to identify patients with chronic pain after knee replacement for a complex intervention trial

Sara Khalid[1], Vicky Wylde[2], Rachael Gooberman-Hill[2], Andrew Judge[1], Rafael Pinedo-Villanueva[1]
[1]University of Oxford; [2]University of Bristol
**Correspondence:** Sara Khalid
*Trials* 2017, **18(Suppl 1):**P80

**Background**
About 20% of patients who undergo primary total knee replacement (TKR) surgery in the UK experience chronic pain after their operation. The provision of healthcare services for these patients has been found to be patchy and inconsistent in the NHS. Although chronic pain is understood to be pain persisting for several months, the level that pain must persist for a patient to be considered in chronic pain is not defined. The aim of this work was to identify a cut-off point in the pain component subscale of the Oxford Knee Score (OKS) that could be used to identify patients in chronic pain following a primary TKR.
**Methods**
We used patient-level data from the English NHS Patient Reported Outcome Measures between 2012/13 and 2014/15. We included data from 126,064 patients who responded to all seven pain component questions of the six-month post-operative OKS questionnaire. The pain component includes questions on level of pain, night pain, pain while walking, standing up, limping, interference with work, and confidence. All questions had five response levels from 0 (most pain) to 4 (no pain) so that the pain component subscale ranges from 0 (most pain) to 28 (no pain).
We adopted a data-driven approach in order to derive groups with different levels of pain using cluster analysis. We applied a hierarchical method of clustering whereby a multi-level hierarchical tree was created by repetitively splitting data into clusters. "similar" observations (based on inter-observation distance) were placed in the same cluster. Clusters were then split until no further dissimilarity could be found, or until the maximum number of clusters was reached. The cluster analysis was run for an increasing maximum number of clusters from two to 10. Clusters were then examined based on their distribution over the range of the pain component subscale to identify if the cluster with the lowest scores (highest pain) was stable as the number of clusters increased. If a stable cluster was found, its highest values would be identified as the cut-off point.
**Results**
The distribution of the hierarchical clusters over the pain component subscale showed a changing shape for the highest pain group when the maximum number of clusters was set to two or three, but a largely consistent distribution was observed when the number of clusters was set to four or higher. The highest score for the pain component subscale for the high-pain cluster was 24 for two or three clusters, but it converged to 14 for four clusters and above.

**Conclusions**
Our study identified a stable high-pain group with consistent OKS pain component scores between 0 and 14 using the hierarchical cluster method. This cut-off point will provide a useful way to identify patients for our trial on post-operative management of chronic pain after TKR in the UK, and we expect that it will be equally useful for other trials focused on patients with chronic pain after knee replacement. Further work to better understand the uncertainty around this cut-off point is recommended before adoption.

**Acknowledgement**
This abstract presents independent research funded by the UK NIHR.

### P81
### Two new residuals in survival analysis with full likelihood
Susan Halabi, Sandipan Dutta
[1]Duke University
**Correspondence:** Susan Halabi
*Trials* 2017, **18(Suppl 1):**P81

Residuals in the proportional hazards (PH) model are useful in detecting outliers or influential points in clinical trials by testing the proportional hazards assumption and exploring functional form. Assuming proportional hazards and non-informative censoring, the full likelihood approach is used to obtain score and deviance residuals. The first residual is based on the ideas used in obtaining the score-type residuals in partial likelihood approach. The second type of residual is based on the concept of the deviance residuals. We conduct simulations and compare the performance of the full likelihood residuals with other common residuals that are based on the partial likelihood approach. In addition, the graphical approaches are used to illustrate the applications of these residuals using some real life examples in clinical trials.
Susan.halabi@duke.edu

### P82
### Application of recent NEJM recommendations for supplementary analyses of positive or failed randomized clinical trials
Mark Van Natta, Laura Wilson, Katherine Yates, James Tonascia
Johns Hopkins Bloomberg School of Public Health
**Correspondence:** Mark Van Natta
*Trials* 2017, **18(Suppl 1):**P82

**Background**
Recently, Pocock and Stone (NEJM, 2016) proposed recommendations for reporting the results of randomized clinical trials depending on whether the primary outcome was "positive" and another set of recommendations when the outcome was "failed." We show that these recommendations are difficult to implement in the body of papers for journals with limits on words, tables, and figures (e.g., 2700 words and 5 tables + figures for NEJM) in the main article; however, the recommendations can be effectively implemented in online supplementary tables and figures to add value to the reporting of the RCT.
**Methods**
The analysis recommendations for 'positive' RCTs include: 1) display of both relative and absolute risk of primary outcome; 2) use of 95% confidence intervals; 3) analysis of parts if a composite primary outcome; 4) analysis of secondary outcomes; and 5) subgroup analysis. The recommendations for 'failed' RCTs include: 1) display of exact *p*-value to assess trend; 2) use of 95% confidence intervals; 3) power calculations; 4) subgroup analysis; 5) analysis of secondary outcomes; 6) alternative analyses including covariate adjustment and as-treated analysis; and 7) meta-analysis using external data.
**Results**
We show how these recommendations can be implicated in practice using data from two published trials sponsored by the National Institute of Diabetes and Digestive and Kidney Disease (NIDDK), one positive and one failed: Farnesoid X nuclear receptor ligand obeticholic acid for non-cirrhotic, non-alcoholic steatohepatitis trial (Lancet, 2014) and Nortriptyline for Idiopathic Gastroparesis trial (JAMA, 2013).
**Conclusions**
Recommendations from Pocock and Stone can be incorporated into the original article's RCT's main or supplementary analyses.

### P83
### A web application for optimal selection of adaptive designs in phase I oncology clinical trials
Sheau-Chiann Chen, Yu Shyr
Vanderbilt University Medical Center
**Correspondence:** Sheau-Chiann Chen
*Trials* 2017, **18(Suppl 1):**P83

**Background**
In phase I oncology clinical trials, the operating characteristics of adaptive designs are used to evaluate the performance of adaptive designs via a simulation study. Research has shown that no single escalation method has proven superior in all circumstances. Thus, an interactive web application with a comprehensive score has been developed to find an appropriate adaptive design for conducting an oncology phase I trial.
**Methods**
The web application evaluates twelve different designs: two versions of the 3 + 3 design, accelerated titration design (ATD), biased coin design (BCD), k-in-a-row (KIR) design, two versions of the continual reassessment method (CRM) design, escalation with overdose control (EWOC) design, escalation based on toxicity interval (EBTI) design, the modified toxicity probability interval (mtpi) design, Bayesian optimal interval design (BOIN) and T-statistics design. The dfcrm, bcrm and BOIN packages in R software are used for CRM, EWOC, EBTI and BOIN designs. Through simulation studies with a matched sample size, a comprehensive score is used to evaluate the performance of selected adaptive designs with desired parameters as well as different scenarios.
**Conclusions**
The web tool provides an interactive graphical user interface that allows users to easily conduct simulations and assess the best design for meeting the primary objective of the proposed trial. Adaptive designs and further information are available at http://cqs.mc.vanderbilt.edu/shiny/adaptivedesigns/and will be updated soon.

### P84
### The cohort multiple randomised controlled trial: methodological lessons from a practical application (the protects trial)
David Reeves[1], Kelly Howells[2], Amy Blackmore[3], Mark Hann[3], Maria Panagioti[3], Peter Bower[3]
[1]University of Manchester; [2]Centre for Primary Care, University of Manchester; [3]Manchester, Manchester UK, University of Manchester
**Correspondence:** David Reeves
*Trials* 2017, **18(Suppl 1):**P84

**Background**
Controlled Randomised Trials often struggle to recruit and there is interest in innovative trial designs that can more effectively recruit and retain patients and make the trials more efficient and patient-centred. One innovation is the 'cohort multiple randomised controlled trial' (CMRCT). Under a standard pragmatic RCT (pRCT) all patients are told about the different treatments in the trial arms, including any new treatment, but only half are randomised to that new treatment. The CMRCT design aims to make the trial consent procedure more like standard health care, where people are only asked to consent to treatments they are being offered and are not told about treatments they cannot access. Under this design a substantial cohort of participants is recruited, then followed up at regular time intervals. To trial a new treatment, all eligible participants are identified and a random sample offered the treatment. The remaining eligible patients (those not offered the treatment)

constitute the control arm. These patients are not informed about the trial or about treatments they will not receive. Advocates of the CMRCT design claim significant advantages regarding recruitment, patient centredness, and efficiency. Since the design was first proposed a number of patient cohorts and related CMRCT s have been established but very few have yet reported and good evidence for these claims is lacking. We established the CLASSIC cohort of 4,377 patients with long-term conditions and are currently conducting a CMRCT (PROTECTS) of a telephone-based health coaching intervention. In the process of conducting PROTECTS, use of the CMRCT design has raised many methodological and statistical issues so far not addressed in the literature. In this paper we consider these issues, report how we tackled them within CLASSIC and PROTECTS, and their implications for the design, conduct and analysis of CMRCTs.

**Results**

We have discovered many challenges to the use of the CMRCT design in actual practice. Primary amongst these are issues around power and sample size calculation, and the nature of the treatment effect being estimated, which have not previously received adequate attention. The rate of patient non-consent to treatment is a critical factor in determining sample sizes for both the CMRCT and the host cohort, and also efficiency relative to a pRCT. We have also found that some sampling practices commonly applied in pragmatic trials, when applied to a CMRCT, can result in selection bias and the introduction of unintended differences between trial arms. The fixed data collection points that are a feature of CMRCTs can result in high variation in the intervals between measurement and treatment that is less controllable than in more conventional designs and can cause problems in analysis. CMRCT -specific CONSORT guidance may be indicated.

**Conclusions**

The CMRCT research design is an intriguing development that may offer several potential advantages over conventional designs. However, there are many challenges to the use of this design in actual practice. Further research and methodological developments are needed to determine whether, and in which contexts, the design can live up to its initial promise.

### P85

#### Split-plot designs: sample size considerations

Beatriz Goulao[1], Graeme Maclennan[2], Craig Ramsay[2]
[1]University of Aberdeen; [2]Health Services Research Unit, University of Aberdeen
**Correspondence:** Beatriz Goulao
*Trials* 2017, **18(Suppl 1):**P85

**Background**

The split-plot (S-P) design is historically associated with agriculture studies, but more recently used in healthcare research. The S-P is a complex design that has both cluster randomised and factorial elements, but is distinguished by two levels of randomisation: one at a cluster-level and one at a lower or individual level. In a previous review, we identified twelve S-P randomised controlled clinical trials (RCTs). Nine reported a sample size calculation and all 9 based the sample size on the cluster randomisation element ignoring the individual randomisation element of the S-P design.

**Objective**

To estimate the optimal sample size for S-P designs and how sample size calculations should be reported.

**Methods**

We used Monte-Carlo simulations to investigate the relationship between the number of clusters (5–45 per arm) with: intra-cluster correlation (0.02, 0.06 and 0.1); intervention target differences at the cluster-level and individual-level (0, 7.5, 15 for each); and statistical power in a S-P design for both for the cluster and patient-level interventions. Current simulations assumed no interaction between interventions and a fixed cluster size of 25 but this will be extended to varying levels of interaction and cluster sizes. Simulated data sets were analysed using a mixed-effects model with a random-effect at the cluster level in Stata 14.

**Results**

We found that power for the cluster and individual-level depended on the intervention target differences expected. If both target differences were similar, a sample size based on the cluster-level intervention leads to an overpowered comparison at the individual –level. The cluster-level power is similar using simulation or applying a standard cluster RCT formula. For a fixed cluster-level target difference, as the ICC increased, there was an increase in power to detect target differences at the individual –level due to increased overall sample size. However, if the individual-level target difference was smaller than the cluster-level there was a point at which the sample size and power should be based upon the individual rather than the cluster-level target difference. For example, we observed that when the individual-level target difference is smaller than the cluster-level by 10% or more for an ICC of 0.01, the sample size should be based on the individual-level difference.

**Conclusion**

Current sample size calculations in S-P RCTs are either non-existent or incomplete. Researchers calculating a sample size for an S-P design should: 1. Indicate explicitly the target differences expected for the cluster and individual-level interventions 2. Base sample size calculations on the cluster-level intervention if the target differences expected at both levels are similar 3. Use simulation if a smaller target difference at the individual-level is expected to estimate the number of participants that need to be recruited The S-P design is an efficient way to assess two interventions, when one of the interventions needs to be randomised at the cluster-level.

### P86

#### Observational versus randomised data: using a fractional factorial RCT to compare results

Jennifer Bell[1], Rhian M. Daniel[2], Pollyanna Hardy[1], Peter Brocklehurst[3]
[1]University of Oxford; [2]London School of Hygiene and Tropical Medicine; [3]University of Birmingham
**Correspondence:** Jennifer Bell
*Trials* 2017, **18(Suppl 1):**P86

**Background**

In clinical research randomised controlled trials (RCTs) are seen as the 'gold standard' for providing evidence to evaluate health care interventions. Randomisation can ensure balance of baseline characteristics between the intervention groups. In observational studies there are often systematic differences between groups, typically analysed using regression adjusting for measured confounders. However, propensity scores (PS) are an increasingly used alternative. This analysis uses the unique opportunity provided by a large fractional factorial RCT to investigate whether similar conclusions can be reached from the results of the randomised interventions and their equivalent observational data from the non-randomised interventions within the same RCT. The observational data are analysed using regression adjustment and propensity scores methods. The RCT investigated was CORONIS, a 2x2x2x2x2 fractional factorial RCT comparing caesarean section techniques on 15,935 women.

**Methods**

One intervention pair from CORONIS (repair of uterus: exteriorisation vs. Intra-abdominal) is assessed for its effect on the primary outcome, death or maternal infectious morbidity. The results of five analyses are presented: using the randomised intervention: the unadjusted, marginal risk ratio (RR), and using the non-randomised intervention: logistic regression to derive the marginal RR, and an inverse probability of treatment weighting propensity score (IPTW PS) model to derive the marginal RR. The results of these analyses are compared.

**Results**

The unadjusted analysis of the randomised interventions provides no evidence of a difference in effect of repair method on the primary outcome (RR 0.94, 95% CI (0.80 to 1.11), SE = 0.08, n = 5925). Regression analysis on the non-randomised interventions adjusting for measured confounding shows similar results to that of the randomised interventions, but is less precise (RR 1.01, 95% CI (0.72 to 1.44), SE = 0.18, n = 5894) and very similar to the IPTW PS analysis (RR 0.98, 95% CI (0.70 to 1.39), SE = 0.18, n = 5925).

**Conclusions**

The results from the analysis on the non-randomised interventions illustrate the importance of adjusting for confounders when analysing observational data. Results based on regression adjustment and propensity score analysis are comparable, but the latter has the added benefit of greater transparency when assessing balance of the baseline characteristics between the groups. It is possible for confounding to be controlled for using standard regression adjustment or IPTW propensity scores, though this may depend on data quality, which this study benefited from.

### P87

#### A comparison of methods to handle missing data in the analysis of an area under the curve outcome

Jemma Hudson[1], Jessica Wood[1], Angus Watson[2], Jonathan Cook[3]
[1]University of Aberdeen; [2]NHS Highland; [3]University of Oxford
*Trials* 2017, **18(Suppl 1):**P87

**Background**

Often randomised controlled trials measure an outcome repeatedly over the study period. An area under the curve (AUC) approach summarises serial measurements using a single measure. Missing data can occur at one or more time-points; it is unclear what the optimum method to use is when missing data are present. The aim of this work was to explore different analysis strategies for dealing with missing data for an AUC outcome in the ethos trial that compared two surgical interventions, stapled haemorrhoidopexy (SH) and traditional haemorrhoidectomy (TH) for treating haemorrhoids. An AUC approach was used because it was hypothesised that the interventions would have different recovery trajectories with respect to quality of life.

**Methods**

The primary outcome in ethos was health related quality of life measured using the EQ-5D over a 24-month follow-up period (baseline, 1, 3 and 6 weeks post-surgery and 12 and 24 months post-randomisation). The AUC was derived using the trapezoidal rule. There was a substantial amount of participants with missing data (30%). The analysis strategies were; 1) complete-case analysis (defined as data available at each time-point); 2) minimal data analysis (including all participants with at least one shorter-term and one longer-term follow-up measure); 3) last observation carried forward (LOCF); 4) simple imputation (intervention group mean at that time-point); and 5) multiple imputation (MI). We used linear regression with adjustment for design variables in Stata 14. Simulations will be carried out to assess the statistical properties of each methods for a range of follow-up profiles and missing data patterns.

**Results**

Seven hundred seventy-seven participants were randomised to SH (389) or TH (388). Analysis of 570 participants with minimal data favoured TH: mean difference in AUC −0.073 95% CI (−0.140, −0.006); $p = 0.034$. The complete case ($N = 400$: −0.057 95% CI (−0.113, −0.001); $p = 0.046$), simple imputation ($N = 774$: 0.054 95% CI (−0.089, −0.019); $p = 0.004$) and MI ($N = 774$: −0.054 95% CI (−0.107, −0.000); $p = 0.049$) analyses were similar. The LOCF analysis was not consistent with other approaches ($N = 774$: 0.025 95% CI (−0.058, 0.109); $p = 0.538$).

**Conclusion**

The complete-case, minimal data, simple imputation and MI analyses were in broad agreement, but LOCF was not. This was because SH had a shorter recovery compared to TH, using LOCF to impute the longer-term missing outcome biased estimates. LOCF should not be used to impute missing data for an AUC outcome when interventions have potentially different recovery trajectories.

### P88

#### Overall success rate of a safe and efficacious drug: results using six phase I designs, each followed by phase II and III trials using standard designs

Amy Ruppert, Abigail B. Shoben
The Ohio State University
**Correspondence:** Amy Ruppert
*Trials* 2017, **18(Suppl 1):**P88

**Background**

The standard 3 + 3 Phase I design remains the most widely used Phase I design in practice, despite an increasing number of both rule-based and model-based designs that range in complexity, but generally outperform the standard (Yuan et al., 2016). Few have integrated common measures of Phase I design performance such as correct selection of the maximum tolerated dose (MTD), average number of dose-limiting toxicities and average number of patients treated above the MTD with a measure of overall success. We aimed to characterize the ability to recognize a safe and efficacious drug by the end of Phase III using six Phase I clinical trial designs, each followed by a standard Phase II and Phase III design.

**Methods**

Six Phase I designs (3 + 3, CCD, BOIN, mtpi, mtpi2, and CRM), each followed by a standard Phase II (Simon's optimal 2-stage) and Phase III (1:1 randomized group sequential with two interim analyses) study were implemented. Dose limiting toxicity and response data were assumed binomially distributed, and survival data exponentially distributed. Eight toxicity profiles representing gradually increasing toxicity across dose levels, fairly constant toxicity that is considered safe, and a jump in toxicity between two adjacent dose levels were each evaluated with a linear response/survival profile using 4000 simulations.

**Results**

As anticipated, results varied depending on the true underlying toxicity profile. Under the conventional assumption that toxicity gradually increases with increasing dose level, all non-standard designs had higher MTD and overall selection rates than the standard 3 + 3 design. However, the non-standard design with the highest MTD selection rates, the CRM, was too aggressive and over-estimated the true MTD most frequently, resulting in the lowest overall selection rates among the non-standard designs. Overall selection rates for the CCD, BOIN, mtpi, and mtpi2 were all within two percentage points of one another and consideration of logistical complexities as well as design familiarity in choosing among these is reasonable. For a fairly constant toxicity profile that is safe, all non-standard designs resulted in higher MTD and overall selection rates than the standard 3 + 3 design. The CRM appeared most aggressive in escalating and recommending the highest dose level which resulted in higher overall selection rates, followed in order by BOIN, CCD/mtpi, and mtpi2. With a jump in toxicities between adjacent dose levels, the standard 3 + 3 design more often recognized when the MTD had been exceeded compared to the non-standard designs; non-standard designs too frequently over-estimated the MTD, resulting in overall success rates that were either similar to or lower than the overall success rate when using the standard 3 + 3 design. Thus, the conservative nature of the standard 3 + 3 design was preferable under these scenarios. In short, selection of a Phase I design should be based on the underlying toxicity profile that is anticipated, with appropriate safeguards, and should consider the MTD selection rate in conjunction with over-dosing and under-dosing errors that influence the overall selection rate of a favorable drug.

### P90

#### Extending the two-stage patient preference design for binary outcomes with stratification

Denise Esserman, Briana Cameron, Peter Peduzzi
Yale University
Denise Esserman
*Trials* 2017, **18(Suppl 1):**P90

Patient preference plays a role in clinical practice, and is at the heart of patient-centered outcomes research, thus ignoring the impact on outcomes would be unrealistic. Furthermore, a patient may have a different psychological response to a treatment he/she deems more favorable. Patient preference can have a substantial impact on a study's outcome, particularly when it is not feasible to conduct a blinded study. While the completely randomized design, the traditional clinical trial setting where individuals or clusters are randomly allocated to one of multiple treatment groups, is the gold standard for assessing a treatment effect (the average effect a particular treatment will have in a specified population), this design ignores the role patient preference may have on study outcomes; they are not estimable in this design. The two-stage trial design first proposed by Rucker, also known as the doubly randomized preference trial, enables researchers to disentangle the treatment effect from those effects resulting from choosing a treatment. While the use of the two-stage trial design is becoming more prevalent, especially as the emphasis for use of decision aids continues to grow and the number of trials testing behavioral interventions increases, there is still a large gap in the methods available to design and ultimately analyze these trials. Often the primary outcome of interest is not measured on a continuous scale; typically, binary outcomes (e.g. Are patients satisfied with their treatment) are used. In addition, we are often interested in accounting for important covariates (e.g. Age, gender, and/or type of insurance coverage) that may have an impact on the outcome of interest, or may influence the preference rate (e.g. Men may have a stronger preference for a surgical intervention, while woman may have a stronger preference for a medical intervention). Currently, no methods exist to accommodate these scenarios. We present our extensions of the two-stage clinical trial design for sample size determination and analysis for binary outcomes with stratification and give closed form sample size formulas.

### P91

#### Pragmatic verification of cancer clinical trial efficacy findings using the surveillance epidemiology and end results (SEER) database

Brian Egleston[1], Elin Sigurdson[2], Elizabeth A. Handorf[2], Eric A. Ross[2], Slobodan Vucetic[3], Yu-Ning Wong[2], Richard J. Bleicher[2]
[1]Temple University Health System; [2]Fox Chase Cancer Center; [3]Temple University
Correspondence: Brian Egleston
*Trials* 2017, **18(Suppl 1):**P91

**Background**
Well-run clinical trials represent the gold standard for assessing efficacy of interventions. Using observational (i.e. Non-randomized) data to determine treatment efficacy requires statistical adjustment and often untestable assumptions.
**Objective**
We determined the degree to which SEER (a large generalized cohort) could replicate findings from the Early Breast Cancer Trialists' Collaborative Group Meta-Analyses of Randomized Trials (a highly selective cohort). This was in part motivated by a research letter on the subject (McGale et al. Can Observational Data Replace Randomized Trials, Journal of Clinical Oncology 2016, 34(27):3355–3356).
**Methods**
We identified women diagnosed with node positive breast cancer treated by mastectomy from 1990 to 2008 in the SEER database. We examined the effectiveness of radiotherapy on survival outcomes using adjusted Poisson regressions, confirmed by Cox or Fine & Grey competing risk regressions. We compared our findings with those reported in a meta-analysis of clinical trials as reported in the Journal of Clinical Oncology.
**Results**
The published meta-analysis found that radiotherapy had a protective effect on breast cancer specific survival (Rate Ratio [RR] = 0.84, 95% Confidence Interval [CI] 0.76 to 0.94), and overall survival (RR = 0.89, 95% CI 0.81-0.97). In the SEER data, we found that the unadjusted effect of radiotherapy on breast cancer specific death was harmful, RR = 1.29 (95% CI 1.25-1.33), but that the adjusted effect was beneficial, RR = 0.91 (95% CI 0.88-0.94). The unadjusted effect of radiotherapy on overall survival was RR = 1.08 (95% CI 1.05-1.11), while the adjusted effect was 0.87 (95% CI 0.84-0.89). Adjustment for the number of positive nodes had a primary confounder impact responsible for the qualitative discrepancy between the unadjusted and adjusted findings.
**Discussion**
Although our adjusted SEER estimates were less beneficial in magnitude than the clinical trial estimates, they were consistent in direction and significance. Of note, clinical trial data often provides evidence of efficacy, while observational data provides evidence of effectiveness (Piantadosi 1997). It is not surprising that the overall effectiveness of a treatment would be attenuated when used in the general population. The discrepancy between the unadjusted and adjusted findings highlight the importance of developing standards for evaluating quality of observational data analyses to ensure proper control of confounding. We are currently developing a software assistant that will assist in standardization of studies using linked SEER-Medicare data. Recently published trial findings have similar comparisons with observational data. Hamdy et al. (10-Year Outcomes after Monitoring, Surgery, or Radiotherapy for Localized Prostate Cancer, NEJM, Advance release) found no statistically significant differences in mortality among men with localized prostate cancer randomized to monitoring, surgery, or radiotherapy. However, fewer than 1.2% of men in all groups died from prostate cancer at 10 years. The point estimates were similar to a SEER-Medicare analysis (Wong et al. Survival Associated with Treatment vs Observation of Localized Prostate Cancer in Elderly Men JAMA 2006). Unlike the randomized trial, the observational data findings were statistically significant, perhaps because the sample size was over 50 times larger.
**Conclusion**
Using properly analyzed observational data can provide generalizable effectiveness information in a non-trial population.

### P92

#### Decomposing the effects of the study population on null results: the look ahead trial

Peter Kaufmann
University of Colorado College of Nursing
*Trials* 2017, **18(Suppl 1):**P92

Obesity continues to be a major risk factor for diabetes and is estimated to cause 365,000 deaths annually in the US, thus placing it as the third cause of death after Heart Disease and Malignant Neoplasms. The Look AHEAD trial sought to determine whether the highly successful lifestyle intervention employed in the Diabetes Prevention Program to reduce incident diabetes among pre-diabetic individuals is effective in reducing the incidence of major acute cardiovascular events. The Look AHEAD trial randomly assigned 5,145 overweight or obese individuals with type 2 diabetes to either a control intervention consisting of Diabetes Support and Education (DSE) or an intensive lifestyle intervention (ILI) consisting of a weight loss program and increased physical activity. The six-month intensive intervention was tapered to a maintenance phase for the remainder of the trial, which had a maximum follow-up of 13.5 years and 90% statistical power to detect an 18% reduction in cardiovascular disease. After the trail was launched, the event rate in the control arm for the primary outcome measure (a composite of death from cardiovascular causes, non-fatal MI and non-fatal stroke) was observed to be only 0.7%, per year, much lower than the design event rate of 3.125% per year. In spite of an adjustment to the primary outcome by adding hospitalization for angina as defined by American Heart Association criteria, and extending the design follow-up from 10.5 to 13.5 years, the trial was stopped for futility after 11 years maximum follow-up.
In an effort to understand the null results, we examined the baseline characteristics of the enrolled trial participants and compared them to baseline characteristics of participants in other contemporary major clinical trials in diabetes. Look AHEAD participants were found to be of younger age, with a higher proportion of females, a lower proportion of history of cardiovascular disease, and shorter duration of diabetes that those enrolled in comparable clinical trials. The profile suggests a much lower risk for cardiovascular events than had been anticipated, approaching a floor effect for benefit of an intervention. The Look AHEAD experience has considerable implications not only for estimating event rates for the purpose of sample size calculations but for methods of managing safety concerns, screening for eligibility as well as monitoring the characteristics of enrolled participants in real time. These factors will be discussed in the context of other clinical trials with null results.

### P93

#### What barriers exist to statisticians recommending alternative methods to permuted block randomisation?

Colin Everett
University of Leeds
*Trials* 2017, **18(Suppl 1):**P93

**Background**
Permuted Block Randomisation (PBR) was first suggested in 1952 by Austin Bradford Hill [1] to restrict the possible degree of imbalance that might occur in a stratified randomised trial. In the following sixty years, several alternative methods have been proposed and well-studied as regards the trade-off between imbalance control and predictability. Such methods include - but are not limited to - the class of biased coin designs, the big stick design, the class of urn designs, the maximal procedure and the block urn design, almost all of which have shown to have lower predictability than their permuted block counterparts. Despite the developments, recent review articles have found that individually-randomised trials performing stratified randomisation (using randomisation within mutually-exclusive strata, rather than covariate-adaptive randomisation methods) almost exclusively use PBR to generate the allocation sequence. In addition, a recent article on the subject [2] stated that 'there is no argument in the literature to suggest that the permuted block design is better than or even as good as the [Maximum Tolerable Imbalance] procedures'. The question must be asked as to why statisticians continue to recommend the use of the inferior PBR method over any other that has been shown to be better at reducing the risk of selection bias.
**Methods**
Presentations were given to statisticians at the Leeds Institute for Clinical Trials Research. In one, presented to an unscientifically-selected sample of statisticians, two alternatives were introduced in a scenario where members of independent oversight committees objected to use of Varying-size PBR in an open-label randomised trial, requesting justification for not using either of the suggested alternatives. (Soares and Wu's Big Stick Method and Zhao and Weng's Block Urn Design) In the second - open to all statisticians - any alternatives were presented in a meeting, and statisticians were invited to discuss reasons for current PBR use and invited to discuss justifications either to continue using PBR, or to use other methods in trials involving restricted randomisation sequences within mutually-exclusive strata.
**Results**
Justifications given by statisticians for using PBR and varying-size PBR included questions around the motivation of clinicians to subvert the randomisation sequence, ability to correctly implement the alternative methods, a belief that weaknesses of fixed-size permuted blocks are overcome by use of randomly-varying block sizes and that alternative methods do not yield sufficient reductions in predictability to justify their use.
**Conclusions**
Since statisticians make recommendations about appropriate methods of allocation sequence generation, take up of better randomisation methods depends on educating statisticians as to existence of alternative methods, that they can be readily implemented, and that the convergence strategy is as big a risk of selection bias as the 'perfect prediction strategy', if not moreso.

**References**
[1] Hill AB. The Clinical Trial. N Engl J Med 1952; 247:113–119.
[2] Berger VW, Bejleric K, Agnor R. Comparing MTI randomization procedures to blocked randomization. Statistics in Medicine. Statist. Med. 2016, 35 685–694. doi: 10.1002/sim.6637

### P94

#### Considering sex as a biological variable in research design, data analyses and reporting

Eve Jelstrom[1], Abigail G. Matthews[1], Dikla Shmueli-Blumberg[1], Robert Lindblad[1], Paul Van Veldhuisen[1], Carmen Rosa[2]
[1]The EMMES Corporation; [2]The National Institute on Drug Abuse (NIDA)
**Correspondence:** Eve Jelstrom
*Trials* 2017, **18(Suppl 1):**P94

In January 2016, a new National Institutes of Health (NIH) policy went into effect requiring that researchers consider sex as a biological variable in animal and human studies (see NOT-OT-15-102 Guidance). This requires grant applicants of NIH-funded research studies to explain how sex will be factored into research design, data analyses and reporting, requiring strong justification for studying only one sex. The policy is meant to increase the quality and generalizability of biomedical research, thus enhancing the reproducibility and translatability of research in the biomedical field.
We will present best practices for implementation of sex as a biological variable in the various phases of a clinical trial, and will include examples from the National Institute on Drug Abuse (NIDA)-funded grants. The first step in implementation involves a rigorous literature review that would explain how sex may influence the study design based on previous preclinical or clinical research. If there is a reasonable foundation of existing research, sex-specific hypotheses could be generated, including primary, secondary or exploratory hypotheses. The next step entails developing a study design, which should include a statistical analysis plan that provides for subgroup analyses identifying differences in the intervention effect by sex. Such subgroup analyses should be implemented using interaction models to test whether the treatment effect differs across sexes, or an analogous approach if there is no modeling. Recognizing that most clinical trials are not powered to detect differences based on gender, these analyses are exploratory in nature. In progress reports and publications, the study findings should include whether sex differences were, or were not, detected. Grantees are also required to report annually on the enrollment of males and females so that this can be monitored throughout the implementation of the trial.
This new policy guides researchers to take into account whether there are biological factors related to sex which should be explored in the study design or approached differently than originally planned. The NIH policy will have an impact on the planning and the conduct of clinical trials in humans and potentially new relationships between disease entities and sex will be explored and given more weight.

### P96

#### Developing a Bayesian adaptive design for a phase I clinical trial: a case study for a novel HIV treatment

Alexina Mason[1], Alan Winston[2], Deborah Ashby[2]
[1]London School of Hygiene and Tropical Medicine; [2]Imperial College London
**Correspondence:** Alexina Mason
*Trials* 2017, **18(Suppl 1):**P96

Phase I clinical trials are typically set up to establish the safety of a proposed drug, study the pharmacokinetics and pharmacodynamics of this drug and to identify a dose which is suitable for taking forward to a further trial. Good design of Phase I studies is often challenging, due to limited evidence to inform study protocols. The traditional approach for Phase I trials uses prespecified rules to assign patients to dose levels and choose the recommended dose for the next study, typically the '3 + 3' design or variations. However, while easy to implement, the operating characteristics of rule-based designs tend to be unattractive. Not only can such designs lead to poor decision making regarding the future investigation of the drug, but they may also expose unnecessary numbers of participants to inappropriate doses. Adaptive designs, such as the Continual Reassessment Method (CRM), which seek to model a dose–response curve using all available information offer an alternative, and can be conveniently carried out within a Bayesian framework. These model-based designs are now well-established in cancer, but much less so in other clinical areas.
A phase I study to assess the safety, pharmacokinetic profile and antiretroviral efficacy of C34-PEG4-Chol, a novel once-weekly peptide fusion inhibitor for the treatment of HIV-infection, was set-up with MRC funding. During the study work up, Bayesian adaptive designs based on the CRM were compared with a more standard rule-based design using simulation studies based on seven test scenarios, with the aim of choosing a design that would maximise the scientific information gained from the study [1]. A dose-inefficacy curve rather than the more usual dose-toxicity curve was modelled. In determining the implementation details, five key questions were addressed: 1) how is the endpoint defined? 2) how will the target dose be identified? 3) what are plausible scenarios? 4) which drug doses should be available? And 5) what cohort sizes are practical? While the results showed no optimal design for all circumstances, the trial team concluded there were clinical advantages in choosing an adaptive design over the originally proposed rule-based design.
The process of specifying and evaluating the design options was time-consuming, and required the active involvement of all members of the trial's protocol development team. However, the effort was worthwhile as the originally proposed rule-based design was replaced by a more efficient Bayesian adaptive design. While the outcome to be modelled, design details and evaluation criteria are trial specific, the principles behind their selection are general. This case study illustrates the steps required to establish a design in a novel context.

1. Mason AJ, Gonzalez-Maffe J, Quinn K, Doyle N, Legg K, Norsworthy P, Trevelion R, Winston A and Ashby D. Developing a Bayesian adaptive design for a Phase I clinical trial: a case study for a novel HIV treatment. Stats Med in press

### P97

#### Tradeoffs of a randomize, then consent approach to improving cluster participation rates in cluster randomize trials

Abigail Shoben
The Ohio State University
*Trials* 2017, **18(Suppl 1):**P97

Cluster randomized trials (CRTs) are increasingly being used in public health research to test interventions that are delivered at a cluster level (e.g., in schools, hospitals, clinics, etc.). From a statistical perspective, the ideal for these trials is first to consent (recruit) participating clusters, then obtain consent from individuals participating from each cluster (if needed), and finally randomize each cluster to a treatment arm. This ideal minimizes the possibility of bias in the estimated treatment effect and preserves the internal validity of the trial. However, this ideal requires that clusters agree to study participation and randomization, which in practice may be difficult and instead result in clusters simply choosing not to participate in the study. Such nonparticipation by clusters may limit the external validity of study findings. Motivated by a school-based trial of physical activity promotion programs in Ohio Appalachia, we discuss strengths and weakness of an alternative approach where clusters are randomized first and then approached for consent to study participation. This approach has the potential to improve cluster consent rates, but risks bias due to differential participation rates by factors that may also influence the outcome. By providing a framework for when this bias may occur and its potential magnitude, we provide guidance for future studies about the statistical tradeoffs between (1) the traditional consent then randomize approach and (2) the alternative randomize then consent approach under various assumptions about cluster participation rates and factors influencing a cluster's decision to consent.

### P98

#### A system for real-time integration of laboratory data into trials of targeted therapy with applications to acute myeloid leukaemia

Sophie Betteridge[1], Robert Kitching[1], Helen Clark[1], Sarah Burns[1], Robert K. Hills[1], Nigel Russell[2], Sylvie Freeman[3], David Grimwade[4]
[1]Cardiff University; [2]University of Nottingham; [3]University of Birmingham; [4]King's College
**Correspondence:** Sophie Betteridge
*Trials* 2017, **18(Suppl 1):**P98

While randomised controlled trials remain the gold standard for the evaluation of new therapies, the development of targeted therapies, disease monitoring, and the concomitant increase in stratification of patients presents new challenges in trial design. Increasingly, patient eligibility becomes dependent on on variables which are known after trial enrolment. Especially in conditions such as haematological malignancies, where delays in therapy can prove costly, treatment modalities may need to be amended in real time to take into account molecular or genetic factors, where the test result is only available some days after the start of treatment, or a patient's minimal residual disease status, again only available at the start of the next course of treatment. It is therefore imperative to be able to direct therapy appropriately, and allow prompt randomisation to the correct targeted therapy. In the context of Acute Myeloid Leukaemia, there are a number of potential targeted therapies which would typically start after a 7–10 day course of chemotherapy. To avoid the dilution of results from patients not suitable for the therapy, randomisation needs to take place once eligibility is known, but before targeted treatment is due to start. Similarly, in an evaluation of minimal residual disease monitoring, only those patients with a suitable target should be randomised between monitor and no monitor. There is therefore a need to ensure communication between clinicians, trials office and the different accredited laboratories so that prompt and appropriate randomisation takes place, allowing patients access to an appropriate targeted therapy, and giving maximal power to detect treatment differences. We present a generalised approach to managing such trials, based upon an integrated computer system, automated notification emails, and monitoring of take-up rates. The approach is flexible enough to allow for several different targeted therapies given in addition to chemotherapy in a factorial design, real-time risk adapted therapy options, and the evaluation of the benefits of sequential disease monitoring using digital PCR. The approach builds upon the AML15 trial, which was an early randomised evaluation of targeted therapy, and has been successfully used with a network of over 100 sites in the UK, Europe, Australia and New Zealand.

### P99

#### Biomarker-guided clinical trial designs: sample size calculations with survival endpoints

Miranta Antoniou, Ruwanthi Kolamunnage-Dona, Andrea L Jorgensen
Department of Biostatistics, Institute of Translational Medicine, University of Liverpool & MRC North West Hub For Trials Methodology Research
**Correspondence:** Miranta Antoniou
*Trials* 2017, **18(Suppl 1):**P99

Biomarker-guided treatment is a rapidly developing area of medicine, where treatment choice is personalised according to one or more of an individual's biomarker measurements. A number of biomarker-guided trial designs have been proposed in the past decade, including both adaptive and non-adaptive trial designs which test the effectiveness of a biomarker-guided approach to treatment with the aim of improving patient health. A better understanding of them is needed as challenges occur in terms of trial design, analysis and practical application, including the control of the false-positive rate, power of the study, prevalence of the biomarker, treatment effect estimation and the potential increases in cost and time. We have undertaken a comprehensive literature review based on an in-depth search strategy with a view to providing the research community with clarity in definition, methodology and terminology of the various reported biomarker-guided trial designs from a total of 211 included papers. Of these 211 included papers, 107 papers related to biomarker-guided adaptive trial designs were reviewed in our published paper Antoniou et al. (2016) [1]; biomarker-guided non-adaptive trial designs were referred to in 100 papers and are reviewed in our more recent paper to be published shortly.
Navigating the literature to gain an understanding of which trial design to implement in a given situation, and the practical implications of doing so is difficult as our reviews revealed. Hence, in order to improve the understanding of the biomarker-guided trial designs and provide valuable and much-needed guidance on their implementation we are developing a user-friendly online tool (www.bigted.org) informed by our literature review. Bigted will provide an easily accessible resource to inform on the most optimal design when embarking on a biomarker-guided trial including easy to navigate graphical displays of the various trial designs. Knowledge on how to design, implement and analyse these trials is essential for testing the effectiveness of a biomarker-guided approach to treatment. Hence, in this study, we focus on key statistical aspects of several of the identified trial designs with particular focus on examining the sample size requirement under different settings where outcome is time-to-event. To achieve this, we applied statistical simulation methods and here we report on our findings.

### P100

#### A systematic mapping review of methods used for the evaluation of implementation fidelity in primary care trials

Rebecca Barnes, Catherine Jameson, Alyson Huntley, Cindy Mann, Alison Heawood, Athene Lane
University of Bristol
**Correspondence:** Rebecca Barnes
*Trials* 2017, **18(Suppl 1):**P100

**Background**
Implementation fidelity has been described as the extent the intervention-as-delivered matched the intervention-as-planned. The primary goal is to increase scientific confidence that an intervention under evaluation has been adequately tested and that the measured outcomes are a reliable indication of its effectiveness. This decreases the likelihood of incorrectly accepting the null hypothesis as a result of inadequate implementation, and of potentially effective interventions being discarded. Under-evaluation or under-reporting of fidelity can also make it difficult to replicate an intervention, and to be sure the outcomes could be reproduced. Primary care may be particularly vulnerable to low implementation fidelity due to a tendency towards pragmatic trials of complex interventions being delivered by multiple implementers across multiple settings. Although important reasons exist for why we should invest in high quality evaluations of implementation fidelity, there is little guidance available, and little evidence for which might be the optimal method in various contexts. The aim of this study was to conduct a systematic mapping review of methods currently in use for the assessment of implementation fidelity across primary care trials.
**Methods**
A search strategy was developed and agreed with input from all members of the research team, two information specialists, representatives from a local group of trial managers and the extant literature. The databases tested for citations were Medline®, Excerpta Medica Database (Embase), and Cumulative Index to Nursing and Allied Health Literature (CINAHL®). For each database, search terms were adapted according to the search capabilities of that database. To be included in the review, studies or trial protocols had to have been published in the last 10 years and report on any primary care intervention undertaken in a general practice setting in the context of a complex effectiveness trial. This included full trials, feasibility studies and/or pilot RCTs. Studies had to state in title or abstract that they had included assessment or reported on implementation fidelity. The initial searches resulted in 6253 citations across the three databases. Electronic abstract screening by two reviewers is underway.
**Next steps**
Discordant decisions will be discussed and persistent discordant decision will be referred to a third reviewer for decision. Following the retrieval and screening of full papers and reference checking, data extraction will be performed by one reviewer and checked by a second. Data extraction will include information on study type, the nature of the planned intervention, the extent of implementation fidelity assessment including methods of data collection and analysis, and level of integration into outcomes evaluation. Preliminary results suggest that implementation fidelity has been under-evaluated and under-reported however these results will be refined through the formal mapping.
**Discussion**
In light of the evidence produced by our review, we will share our recommendations for practical steps towards high quality evaluation of implementation fidelity in the design of future primary care trials. We will also discuss the strengths and limitations of methodological reviews of trial conduct and how the quality of implementation fidelity evaluation might be more formally appraised going forward.

### P101

#### A systematic review of analytical methods applied for discarded organs in kidney and liver transplantation research

Richéal Burns, Peter Skerritt, Simon Knight, David Nasralla, Ally Bradley, Virginia Chiocchia, Rutger Ploeg, Peter Friend
University of Oxford
**Correspondence:** Richéal Burns
*Trials* 2017, **18(Suppl 1):**P101

**Background**
The feasibility and effectiveness of novel procedures for improving viability of organs for transplantation is a growing area of research and assessing the various outcomes of these procedures is vital to understanding the overall benefit relative to current practices. Understanding the implications of reducing discard rates, particularly in kidney and liver transplantation, would aid in the development of more practical analytical frameworks for assessment of these novel procedures.
**Objective**
The primary objective of this systematic literature review is to identify research which assesses the clinical and economic impact of kidney and liver discard rates in transplantation research in order to understand how kidney or liver discard rates impacts on the risk of disease progression and mortality as well as cost of care.
**Methods**
The following databases were searched: MEDLINE via PubMed, EMBASE, the Cochrane Central Register of Controlled Trials (CENTRAL), the Transplant Library and the National Institute of Health Clinical Trials database. Reference lists were also hand searched for other relevant trials/reviews. Research focusing on Kidney or Liver transplantation was reviewed. To be included in the review, the research results needed to specify the status of and/or analytical assessment of discard rates. Extracted information from studies meeting the inclusion criteria included: author, year, title, organisation, country, study type, organ type, donor type, categorisation of reference to discarded organs within research and outcomes reported. The quality of the studies included was assessed using the Cochrane Risk of Bias Tool; for assessing the quality of reviews, the ROBIS bias assessment tool was applied.
**Results**
TBC

### P102

#### Outcome measurement in paediatric proton beam radiation therapy studies: is greater standardisation needed?

Caroline Main[1], Simon P. Stevens[1], Roger E. Taylor[2], Barry Pizer[3], Nick Thorp[4], Keith Wheatley[1], Pamela R. Kearns[5], Robert Phillips[6], Martin English[5], Sophie Wilne[7]
[1]University of Birmingham; [2]Swansea University; [3]Alder Hey Children's NHS Foundation Trust; [4]The Clatterbridge Cancer Centre; [5]Birmingham Children's Hospital NHS Foundation Trust; [6]Centre for Reviews and Dissemination; [7]Queen's Medical Centre
**Correspondence:** Caroline Main
*Trials* 2017, **18(Suppl 1):**P102

**Aims**

The Core Outcome Measures in Effectiveness Trials (COMET) initiative was instigated to develop the minimum set of standardised key outcomes that should be assessed in studies, making it easier for results of different studies to be compared, contrasted and combined. Within a systematic review of the effects of proton beam radiation therapy (PBT) in children with malignant Central Nervous System (CNS) tumours we assessed the standardisation of outcome measures utilised.

**Methods**

Twelve electronic databases were searched from 1985 onwards. Comparative and non-comparative studies were included. Outcomes included overall survival (OS), surrogate survival outcomes, local/distant failure rates (LFR and DFR), response rates, toxicities, long-term adverse events, neurocognitive outcomes and quality of survival. Standard systematic review methods were used to minimise bias in study identification, selection and data extraction.

**Results**

Seventeen studies (one single arm phase II trial and 16 case series) included a total of 492 patients. Mean sample size of 29 (range: 6–109), with a mean length of follow-up of 3.1 years (range: 0.1-27.2 years). All treatment regimens assessed were multi-modality. Of the 492 patients, 442 (90%) were newly diagnosed and 53 (10%) had relapsed disease. Tumour types included were low-grade gliomas, medulloblastoma or supratentorial primitive neuroectodermal tumours(SPNET's), ependymoma,atypical teratoid rhaboid tumours (AT/RT), germ cell tumours, and pineoblastomas.

Fifteen studies (460 patients; 93%) reported OS, eight progression-free survival (265 patients; 54%), three disease-free survival (144 patients; 29%) one event-free survival (8 patients; 2%), one time-to-progression (59 patients; 12%), 12 LFR (342 patients; 70%), eight DFR (190 patients; 39%) and five response rates (39 patients; 8%). However, all survival outcomes were measured from different time points across the studies. Two studies (5%) measured survival from diagnosis, five (38%) from the start of PBT, six from completion of PBT (28%), whilst the remaining three studies (29%) did not report baseline timing of survival outcomes. This can potentially alter results. One study of 31 patients (6%) reported 2-year OS from both the time of diagnosis [68.3% (95% CI: 52.9%-88.1%)] and the end of PBT [52.9% (95% CI: 36.0% - 77.8%)] indicating the differences in results obtained from when baseline PBT is measured. Endocrinopathies and ototoxicities were the most commonly reported medium-term adverse events, both reported in seven studies with 229 (47%) and 126 (26%) patients respectively. There was little standardisation in the measures used to assess either outcome. In relation to endocrinopathies six different scales, including any type of endocrinopathy (unspecified), had been utilised, and the studies employed different criteria to define these outcomes. In terms of ototoxicities, a total of five different scales had been used across seven studies, with only two using standardised outcome measures. Results on neurocognitive outcomes (85 patients) were highly limited.

**Conclusions**

There is a need to standardise outcomes and their measurement within studies of children undergoing radiotherapy including PBT. In particular, survival outcomes need to be measured from the commencement of PBT in order to facilitate comparisons between different studies. Long-term adverse events need to be better defined and measured using standardised scales.

### P103

#### Handling missing or incomplete data in a Bayesian network meta-analysis framework

Danila Azzolina[1], Ileana Baldi[1], Paola Berchialla[2], Clara Minto[1], Dario Gregori[1]
[1]University of Padua; [2]University of Turin
**Correspondence:** Danila Azzolina
*Trials* 2017, **18(Suppl 1):**P103

A Bayesian NMA model is often used to estimate the effect of each intervention compared to others synthesizing results using rank probabilities. In several cases, a NMA is associated to a loss of information due to incomplete study data retrieved through a systematic review, which are therefore excluded from the analysis. Several methods are provided in literature to handle missing or incomplete data in a NMA. It is often the case that only baseline and follow-up measurement are available; to obtain data about mean change it is necessary to consider pre-post study correlation. In a Bayesian framework, some authors (Abrams, 2005), suggest imputation strategies of pre-post correlation. In other cases, a variability measure associated to mean change score might be unavailable. Different imputation methods are suggested, as those based on maximum standard deviation MSD imputation. The purpose of this study is to verify the robustness of Bayesian NMA with respect to different imputation strategies through simulations. Fifty trials are simulated in full databases by including baseline, follow-up and Delta variation information. Baseline data are obtained by sampling from bounded 0–100 normal distributions ($X \sim N(41.8,21.5)$) (Cannon, 2000), to mimick the support of WOMAC score. Delta variation data are simulated from normal distributions with parameters provided by a review about 6 FANS treatments. Follow up variability data are provided from generated Delta and baseline variability measures setting hypotheses on pre-post correlation and considering, in each scenario, a sequence from 0.3 to 0.95 by 0.05. Sample size are obtained by sampling from an uniform 50–100 distribution. Between trial heterogeneity has been included as a variability measure by following, for each simulation setting, a sequence from 0.1 to 5 by 0.1. Each scenario provides different combinations of heterogeneity between trials and pre-post correlation creating 700 scenarios. For each scenario 2 imputed databases are generated. In the first case, information about Delta variation are randomly removed, from full database, leaving only baseline end follow up data, then variability of mean change is imputed using the correlation method. In the other case, also information at baseline and follow up are removed, then Delta variability isimputed with maximum standard deviation method. On simulated dataset, NMA, with random effect and Uniform (0,5) prior on heterogeneity parameter, has been performed (MCMC method, 200000 iterations, 4 chains). To investigate robustness of NMA, under several scenarios and different imputation methods, the bias of rank probabilities estimates has been computed in order to check models performance in ranking treatments. For each scenario, the mean, bias and the standard deviation of the first rank probability, for full and imputed databases, have been computed. The results show that the bias is very small for every scenario, then ranking provided by models is robust with respect to different imputation methods. The method is more robust to imputation in a low heterogeneity framework, especially if considered trials are conducted on similar population. Small bias is observed also for heterogeneity values similar to expectation of NMA heterogeneity prior, indicating more robustness if a priori knowledge is well specified.

### P104

#### Cluster randomized clinical trials: can they be found in pubmed and clinical trial registries and do they adhere to consort guidelines?

Elizabeth Wright, Kenneth J. Wilkins
National Institute of Diabetes Digestive and Kidney Diseases/NIH
**Correspondence:** Elizabeth Wright
*Trials* 2017, **18(Suppl 1):**P104

**Background**

A cluster randomized clinical trial (CRCT) is a trial that randomizes clusters of people, but collects data on individuals. Concerns about the quality of reporting of results from CRCTs led to the publication of Consolidated Standards of Reporting Trials (CONSORT) statements for CRCTs in 2008 and 2010. Additional CONSORT criteria for CRCTs include: identification as a CRCT (title), the numbers of clusters and individuals randomized to each group (abstract), the intracluster correlation used for sample size calculation, and how clustering was taken account in the statistical methods. A review of CRCTs published in 2000–2008 concluded that reporting improved after CONSORT publication but remained suboptimal. No reviews have looked at the identification of CRCTs in clinicaltrials.gov and other registries. We sought to determine whether adherence to CONSORT guidelines has improved and whether CRCTs could be identified in trial registries.

**Methods**

We focused our review on CRCTs designed to improve the care of patients with diabetes through interventions aimed at either patients or health care providers. We searched pubmed in September 2016 using the terms Diabetes AND ((cluster randomized) OR (cluster randomised) OR (group-randomized) OR (group-randomised)). Reviews, bibliographies, and registries were searched for additional publications. Publications were classified as: diabetes treatment, diabetes prevention, or not diabetes and as CRCT or not. We extracted data on the adherence to CONSORT guidelines and determined the trial registration status for each publication (included in the publication, registered but not included, or not registered). This information was used to group publications by trial and we selected the primary results publication, if any, for each trial.

**Results**

Our search identified 557 English language publications between 2000 and 2015, 349 of which included patients with diabetes. 262 (75%) were reports of CRCT. Excluded publications used terms like "parallel group randomized", "cluster of risk factors", "cluster sampling", and "Cluster analysis" or were reviews. A few excluded publications called themselves CRCT but were trials where individuals were randomized to receive treatment in groups. An additional 54 publications were found for a total of 316 publications from 186 trials: 143 primary results, 81 design, and 92 secondary. We grouped the 143 results publications by year published: 44 in 2000–2007, 39 in 2008–2011, and 60 in 2012–2015. The percent with CRCT in the title (18%, 51%, 63%) and the percent registered (11%, 79%, 83%) increased over time. 86% had the number of clusters in the abstract, 78% discussed clusters in the statistical analysis plan, and 49% included the sample size intracluster correlation. Only 39/86 registered trials included the word cluster in the registration (clinicaltrials.gov 17/47, ISRCT 14/26, ACTRN 4/6, others (4/7).

**Conclusions**

The quality of CRCT publications has increased, but there continue to be publications that are underpowered and do not account for the effect of clustering in the analysis. Trial registries do not currently include a code for CRCT or a structured means of recording the number of clusters in results. The description of CRCTs in registries needs to be improved so these trials can be included in systematic reviews.

### P105

#### Building on the past: systematic identification, data extraction and synthesis of pre-existing individual stroke patient datasets to inform the development and design of future clinical trials

Andrew Elders[1] the RELEASE[2]
[1]NMAHP Research Unit, Glasgow Caledonian University [2]Nursing Midwifery and Allied Health Professions Research Unit
Collaboration, The RELEASE (www.aphasiatrials.org/index.php/research/release)
*Trials* 2017, **18(Suppl 1):**P105

**Background**

The number of stroke rehabilitation trials reported is rapidly increasing. Efficient trial design contributing to advances in rehabilitation should be informed by completed trials in the field. More than 50,000 people in the UK each year acquire aphasia: a stroke related language impairment affecting the ability to speak, understand speech, read and write with significant consequences for quality of life. Existing Cochrane systematic review evidence indicates that speech and language therapy (SLT) benefits language recovery in people with aphasia, however, the specific patient and intervention factors which predict optimal recovery and rehabilitation are unclear. By using a wider dataset with individual patient data (IPD) analysis we are enhancing the evidence synthesis process with the aim of addressing these evidence gaps. RELEASE (rehabilitation and recovery of people with Aphasia after stroke) is an international collaboration of aphasia researchers which seeks to achieve this goal.

**Objectives**

Funded by the National Institute for Health Research (Health Services and Delivery Research - 14/04/22) we have systematically gathered IPD from pre-existing aphasia research datasets to examine the natural history of recovery from aphasia, the predictors of recovery and optimal interventions (by rehabilitation regimen, delivery model and the aims and content of treatment).

**Methods**

We invited contributions of primary datasets from members of the Collaboration of Aphasia Trialists (cats). We also conducted a systematic search of existing published research to identify a comprehensive set of potentially existing aphasia research datasets which met our inclusion criteria. Research datasets were required to include a minimum of 10 people, a measure of aphasia severity as a consequence of stroke and information on time since stroke. We invited researchers from these studies to contribute data and to create a unique multilingual, international, interdisciplinary resource in this clinical field.

**Results**

Following a systematic search of the literature, we screened 5276 titles (including 2346 abstracts and 1152 full texts), from which we identified 874 eligible studies. We have received 76 study datasets contributing IPD from 4597 people with aphasia (56 through the systematic search and 20 via cats). These data have been contributed from 23 countries and we have identified a further 2400 IPD in the public domain. The substantive challenge is our planned IPD meta-analysis to examine recovery, predictors of recovery and effectiveness of intervention approaches. Our statistical analysis plan states that a one-stage approach will be conducted for the primary analyses, although a two-stage approach will also be explored. Network meta-analyses and meta-regression (some of which includes subgroup analyses) are also planned. We will discuss the methodological challenges, particularly which arise when there are non-standardized data, some non-randomized data, a large number of outcome measurements and some degree of sparse data.

**Conclusions**

RELEASE is the largest systematically developed, evidence synthesis study in the field of aphasia, and is more complex than most IPD trial meta-analyses. Our research will not only provide important evidence relating to the recovery of people with aphasia, but will also be an exemplar to researchers who plan to create databases to analyse complex individual patient data.

### P106

#### A literature review of the use of adaptive design methods in oncology trials

Pankaj Mistry, Janet Dunn, Andrea Marshall
Warwick Clinical Trials Unit, University of Warwick
**Correspondence:** Pankaj Mistry
*Trials* 2017, **18(Suppl 1):**P106

**Background**

The implementation of adaptive design methods in phase II, phase III or phase II/III has increased over the years [1]. There is a need for a set of guidelines to report adaptive design methodology used in clinical trials in addition to the CONSORT guidelines [2] to ensure full transparency of trials implementing predetermined or concurrent adaptations. The aim of this literature review is to understand the current application of adaptive design methodology in oncology trials, and to ascertain how this methodology is reported.

**Methods**

A literature search of PubMed, Embase and Ovid databases for full text publications of phase II, phase III or phase II/III cancer trials using adaptive design methodology during 2015 was conducted. The key words used for the literature search are as follows: adaptive design, flexible design, group sequential, sample size re-estimation, MAMS, adaptive randomisation, interim analyses, adaptive seamless, biomarker adaptive, two-stage adaptive, dose escalation, 'Drop the loser', 'Pick the winner', multiple adaptive, adaptive enrichment.

Relevant full text articles were reviewed to identify the type of adaptive methodology applied, if the publication explicitly stated the use of adaptive designs and whether adaptive design methodology was applied prospectively or concurrently.

**Results**

The databases produced a total of 14544 phase II, phase III, phase II/III cancer trial related articles that were published in 2015. Of which 99/2127 (5%) for the PubMed database, 519/9573 (5%) for the Embase database and 116/2844 (4%) for the Ovid database articles included the search terms related to adaptive design methodology. After the removal of duplicates, 464 articles were remaining. Of the 464 articles, 92 (20%) were full text trial related publications, 261 (56%) were abstracts, 32 (7%) were methodology or review papers and 79 (17%) were not related to the search criteria.

The adaptive design methodology used in over half of the trials (48/92) was applied by performing interim analyses due to safety, efficacy or futility, 21 out of 92 trials incorporated dose escalation methods and 14 out of 92 implemented a two-stage design. The remaining 9 trials applied the following methods: Bayesian adaptive design (3/92), group sequential design (3/92) change in primary endpoint (1/92), seamless phase II to phase III (1/92), multiple adaptive (1/92). Despite using adaptive design methodology, only four trials explicitly stated that it had an adaptive design. There were 89 out of 92 trials that had prospectively planned adaptations, of which two of these also incorporated an ad-hoc interim analysis.

**Conclusion**

This review has highlighted that adaptive design methodology is rarely explicitly stated and hence supports the argument for needing a set of guidelines to report the adaptive design methodology used in clinical trials. Furthermore specific reporting guidelines will assist in the consistency of reporting and ensure the ease of future identification of trials implementing any prospective or concurrent adaptive design methodology.

1. Hatfield, Isabella. Adaptive designs undertaken in clinical research: a review of registered clinical trials. Trials (2016): 1273–9
2. www.consort-statement.org/consort-2010

### P107

#### Evidence based evaluation of practice using the ideal-physio framework. A strategic method of evaluating innovation and current practice

David Beard[1], Arsenio Paez[2], Loretta Davies[1], Jonathan Cook[1], Allison Hirst[1], Peter McCulloch[1]
[1]University of Oxford; [2]Northeastern University
**Correspondence:** David Beard
*Trials* 2017, **18(Suppl 1):**P107

**Background**

Whilst there have been recent improvements, the practice and profession of physiotherapy (Physical Therapy) has suffered from the uncontrolled introduction and proliferation of treatments which have an inadequate scientific basis, little or poor evaluation, and underexposure to rigorous scientific method. New treatment modalities can be developed and introduced without evidence of efficacy, regulation or governance. There is no requirement to collect prospective data to support any claims or demonstrate efficacy. This approach has resulted in numerous disparate practices which may not stand up to rigorous evaluation or evidence based commissioning.

**Description**

The IDEAL framework is an established method of formalising the systematic evaluation of innovation (and existing practice) in complex clinical interventions. It has been useful for setting out an internationally based evaluation framework for surgical procedures. This framework lends itself to other complex, non-pharmacological interventions such as Physiotherapy (Physical Therapy). We outline the application of this framework to Physiotherapy (Physical Therapy) in a new IDEAL-Physio framework.

Similarly to IDEAL for surgery, five stages exist; each representing a letter of the acronym.

Stage 1, the Idea phase where formal data collection should begin. This requires quality recording of data using standardised outcome measures. The emphasis is on explanation and description. Stage 2a, the Development phase, is a period of iterative improvement and adjustment with thorough prospective data recording. It focuses on technical details and feasibility. Stage 2b, the onset of formal Exploration evaluation using systematically collected group or cohort data. This stage is a bridge or a pilot to a full RCT. It further refines outcome measures and takes account of learning curves. Stage 3, is a formal comparative Assessment phase of treatment usually involving randomised studies. It involves a full assessment of efficacy. Stage 4; Long term follow up involves monitoring outcome, particularly in long term conditions.

**Recommendations**

We recommend the use of IDEAL - Physio to help guide and evaluate innovation with the overall strategy of providing better evidence based care and foster innovation in Physiotherapy (Physical Therapy). This paper outlines the principles of IDEAL - Physio and describes its utility in changing practice on a global level.

Keywords: Physical Therapy, Innovation, Evaluation,

### P108

#### A journey from statistical consultation to funded adaptive trial

Toby Prevost
Imperial College London
*Trials* 2017, **18(Suppl 1):**P108

**Background**

A funding board assessed an application for a Phase 1 three-doses dose escalation trial of a treatment in Crohn's Disease, but disliked the deterministic 3 + 3 design. The principle applicant sought statistical advice which led to an adaptive design with extra doses. Another board disliked this adaptive design and the 3 + 3 was reinstated. The boards jointly requested a three-dose adaptive design which was funded. This incorporated a Phase II stage, and a crossover design to allow placebo-controlled periods in blocked patient pairs. The aim is to describe the use of the adaptive Bayesian Continual Reassessment method available in BCRM Software [1], for designing dose escalation trials.

**Methods**

There was a consensus to keep the sample size near 20. A 20% target toxicity event rate for the Maximum Tolerated Dose was set, below the 33% relevant in oncology trials. The performance of the 3 + 3 and BCRM methods was assessed through setting a range of likely and unlikely scenarios for the event rates at the 3 doses, followed by simulation (3000 trial repetitions). Primary assessment was the percentage of trials recommending each of the three doses. The 3 + 3 with a sample size of 18 was contrasted with a BCRM of sample size 16 specifying a one-parameter power model and a Gamma prior.

**Results**

Within the software, we specified five scenarios for the event-rates across doses. For the 'upper dose is just safe' scenario, BCRM outperformed 3 + 3 in discovering the highest dose (72% versus 62%). For the 'upper dose is just unsafe' scenario, the BCRM recommended the upper dose 54% versus 48% times, but 3 + 3 incorrectly recommended the lowest dose more often (22% versus 10%). For the 'all event rates are ascending but lower than the target' scenario, BCRM recommendations across doses were superior (5%,14%,81%) relative to 3 + 3 (34%,35%,31%). For the 'highest dose is unexpectedly high' scenario, both approaches recommended it in just under 10% of simulations. For the 'highest dose is plausibly high' scenario, both approaches recommended it approximately one-third of the time, and BCRM recommended the correct dose more frequently than 3 + 3 (41% versus 30%).

**Conclusions**

Some funding boards are encouraging of researchers to adopt and exploit the advantages of novel designs. The availability of the BCRM software enabled a range of event-rate scenarios to be examined for a three-dose example with moderate target rate. Across scenarios, this adaptive approach was seen generally to outperform the 3 + 3 design, with a smaller sample size. The software was flexible in allowing patients to be recruited and dose-allocated in pairs, accommodating a crossover element. The scenario results indicate that with samples of a typically small size in Phase 1, there is surprisingly quite a lot of sampling variability in the dose recommended. Simulation is therefore an important part of adaptive and deterministic design planning. Consideration could be given to exploring modestly raised sample sizes, and/or a further stage of dosing around the initially recommended dose, and/or carrying forward an extra dose to Phase 2.

### P109

#### Translation of a single-center cellular therapy manufacturing approach to a multi-center, center-specific manufacturing platform: the experience of the blood and marrow transplant clinical trials network (BMT CTN) protocol #1401

Courtney Nelson[1], Kelly O'Brien[1], Adam Medizabal[1], Iris Gersten[1], Lynne Uhl[2], David Chung[3], Nina Shah[4], David Avigan[2], Marcelo Pasquini[5]
[1]The Emmes Corporation; [2]Beth Israel Deaconess Medical Center; [3]Memorial Sloan-Kettering Cancer Center; [4]University of Texas, MD Anderson Cancer Center; [5]Medical College of Wisconsin
**Correspondence:** Courtney Nelson
*Trials* 2017, **18(Suppl 1):**P109

Cellular therapy for hematologic malignancies is an emerging area of cancer therapeutics. One of the major challenges of cellular therapy research is the ability to make specialized processes widely available. BMT CTN protocol #1401 was designed to translate a single-center manufacturing process of a vaccine with dendritic cell/myeloma fusions to a model where the investigational product is manufactured locally at each participating institution. The study provides a framework for implementing multi-center cancer vaccine studies requiring a unique approach for clinical protocol and manufacturing process development, Investigational New Drug (IND) submission, site selection, training and qualification, and data collection. The primary challenge was adapting a single-center process to a multi-center clinical protocol. Study specific standard operating procedures (sops) for cell collection and manufacturing were developed utilizing the institutional sops from the single center study and managed centrally via an SOP management process. Laboratory staff at participating sites performed an in-depth review of the manufacturing sops and identified processes requiring generalization based on available equipment and institutional-specific processes. Participating sites were invited based on past participation and experience with dendritic cell vaccine clinical research. Following site selection, each institution attended training in-person and via teleconference to discuss SOP development and familiarize key staff with the production process. Two successful mock runs of the vaccine product were required prior to site activation. Initiating these training procedures concurrent with protocol development and FDA submission allowed for faster site activation upon protocol release. The IND application included an in-depth overview of the site training, qualification, and selection procedures to assure that all sites were adequately trained in vaccine manufacturing. There are several unique components of data collection and monitoring in the context of cellular therapy. It is integral to the integrity of the study and safety of the participants to monitor compliance with the manufacturing sops to ensure that each product meets criteria for release. An electronic system was developed to monitor vaccine production and release centrally in real time. Sites are required to submit electronic reports of any deviation from the study sops via the data system within 24 hours of knowledge of the event. These reports are reviewed by medical monitors, and recommendations for corrective actions and future prevention of deviations are provided to the site within five business days. All sites will enter electronic data on the vaccine release criteria and upload the completed study specific worksheets. These data will be reviewed and approved centrally prior to local release of the vaccine for administration to patients. Any deviations noted in the worksheets will be reported and reviewed by the medical monitors to ensure the final product is not compromised. While there continue to be ongoing challenges, BMT CTN 1401 provides a framework for the successful implementation of a multi-center cellular therapy clinical trial by utilizing a single-center experience in conjunction with an established clinical trials network. Further efforts are needed to explore the application of this framework to additional therapeutic areas.

### P110

#### Streamlining SAE handling in a busy CTU

Eleanor Mitchell, Kirsty Sprange, Clare Brittain, Isobel Hawley
University of Nottingham
**Correspondence:** Eleanor Mitchell
*Trials* 2017, **18(Suppl 1):**P110

#### Background

Serious Adverse Events (SAEs) are routinely collected in many trials and processed by Clinical Trials Units on a daily basis. In the past at Nottingham Clinical Trials Unit (NCTU), saes were processed by the Trial Manager (TM), however this could lead to problems if they were absent, with the potential that Ss were not handled in a timely manner. In 2013 a database was developed to track all incoming SAEs Unit-wide, so that members of the team could efficiently process SAEs on any trial within the Unit. This is done on a rolling rota basis and TMs are responsible for ensuring cover if they are unable to undertake their duties. This ensures all TMs have exposure to SAE handling, irrespective of whether their own trial collects SAEs or not and is therefore good for staff development. It also ensures full cover across all trials, irrespective of whether the specific trial team are available. The roll-out of this system was initially successful, however problems later developed with standard procedures being followed inconsistently and a lack of consistent oversight with no formal procedures in place.

#### Methods

Updates were made to the database design to enhance the user experience and additional reports were added. Emails were then changed from manual to automated to speed up the process of receipt acknowledgement and sending SAEs for medical review. A thorough training session was provided to TMs on the rota, delivered by the Senior Trial Managers and the database developer. All trials now have trial-specific SAE handling instructions stored centrally for easy access; these instructions give step-by-step guidance on how to handle an SAE for a particular trial.

An oversight process was then put in place which involves, at a trial level, the specific-Trial Manager and, at a Unit-level, the team of Senior Trial Managers and QA Manager. A range of easy-to-use checklists were produced to ensure consistent regular oversight, undertaken on a rota basis. Oversight also includes running a series of regular reports through the tracking database and ensuring, via the TMs, that the database and other documentation is kept fully up-to-date. TMs are also encouraged to use in-built reports to allow accurate and timely reconciliation of SAEs.

#### Results

Streamlining the existing process took approximately 6 months and was fully introduced in August 2016. Feedback from users has been positive. The trial-specific handling instructions have been helpful to all safety handlers and design and functionality changes to the database have been received positively. Oversight is now done consistently across all trials with appropriate accompanying documentation filed, essential for audit and inspection purposes.

#### Conclusion

Implementing the database and rota system in 2013 was a step in the right direction. However, streamlining the process and implementing more robust oversight ensures that SAEs are handled appropriately and efficiently to ensure regulatory timelines are met. Including TMs in reviewing and updating the tracker database and relevant documentation has been essential to ensure buy-in from users. Clear communication and training whilst updating the process has also been crucial.

### P111

#### Operational advantages and challenges with pragmatic precision medicine trials: the TAPUR study

Pam Mangat, Kaitlyn R. Antonelli, Suanna S. Bruinooge, Richard L. Schilsky
American Society of Clinical Oncology
**Correspondence:** Pam Mangat
*Trials* 2017, **18(Suppl 1):**P111

The American Society of Clinical Oncology's Targeted Agent and Profiling Utilization Registry Study (TAPUR) is a Phase 2, non-randomized, precision-medicine, basket trial founded in real-world clinical practice. The TAPUR study includes investigational therapies that are approved by the U.S. Food and Drug Administration to target genomic alterations in patients with advanced cancer known to be a drug target or predict drug sensitivity. TAPUR is a large multi-site study with an innovative design that allows for assignment of 1 of 15 possible study regimens by the treating physician according to protocol defined drug-genomic alteration matching rules or guidance from the study Molecular Tumor Board (MTB). TAPUR has broad eligibility criteria, flexibility in treatment administration, and collection of data that mirrors routine clinical care. Analysis of study endpoints requires completion of cohorts defined by study drug, genomic variant, and tumor type according to a Simon 2-stage design where cohorts are closed or expanded based on response rate.
TAPUR's pragmatic approach incorporates the clinician and patient perspective into the design. For example, enrollment is expanded through use of broader eligibility criteria, including participants with performance status (PS) 0–2, prior malignancies or HIV, and previously treated, but stable brain metastases. To date, 19% of participants have PS = 2 and 22% reported prior malignancies. This approach not only provides greater trial access to patients and maximizes accrual, but also increases generalizability. Drug dosing is according to the drug label and modification is allowed through clinician judgment. Data collection centered around routine clinical care reduces site burden and costs, while also allowing for rapid enrollment since the trial can leverage existing data, critical for patients and clinicians looking for timely treatment options.
However, there are operational challenges associated with this level of flexibility. For example, due to the heterogeneity of tumor types and genomic alterations, dozens of cohorts are generated that enroll slowly (to date 82 cohorts exist for 102 participants). Therefore, total sample size for any cohort, site or drug are difficult to estimate. In turn, an overall study budget is also difficult to assess since many costs, such as drug distribution and per-case reimbursements, are related to the number of enrollments. It is important to allow for ongoing modification, inform collaborators about the need for fluidity of the project and consider novel operational approaches. For example, due to challenges in estimating allocation of drug quantities to clinical sites, the study utilizes a specialty pharmacy as the drug distributor, with drug provided on a per-participant basis at the time of order to reduce waste and negate the need for pre-defined estimates. Lastly, while the broad eligibility criteria is a clear advantage, it does require regular retraining of clinicians to utilize their best clinical judgment when assessing eligibility. Leniency in measures such as performance status may result in an enrolled participant unable to meet all study requirements or achieve key study endpoints, like response evaluation.
This presentation will highlight the innovative features of the study design, as well as design-related operational advantages and challenges for consideration.

### P112

#### Is there anyone so wise as to learn by the experience of others? Developing and implementing a 'Lessons Learnt' database in a clinical trials unit

Helen Myers[1], Ian Thomas[2], Stephanie Tooth[1], Tom Shepherd[2], Jo Smith[1], Rachael Heath[1], Sarah Lawton[1], Kris Clarkson[1]
[1]Keele Clinical Trials Unit; [2]Institute for Primary Care and Health Sciences
**Correspondence:** Helen Myers
*Trials* 2017, **18(Suppl 1):**P112

**Introduction**
Staff within large research organisations who are responsible for managing a number of complex studies gain a vast amount of knowledge and experience that is not always systematically and effectively shared with others. Embedding a 'Lessons Learnt' culture within an organisation can provide staff with the opportunity for reflection and promote a culture of problem-solving, ensuring that best practice is applied and mistakes are not repeated. A systematic approach to capturing and communicating learning can help to streamline and improve the quality of research procedures.
**Background**
Keele Clinical Trials Unit (CTU) aims to promote a culture where staff learn from previous experiences and apply best practice to become more efficient. The aim of the 'Lessons Learnt' database developed at Keele CTU is to capture and learn from positive and negative events or processes which occur during the life of a research study, and to communicate these to others. The overall objective is to make improvements to, and streamline the processes of setting up and running a research study.
**Methods**
A 'Lessons Learnt' database specification was written following the generation of ideas by a group of experienced Trial Managers and Data Managers. The database was built using Access, and iterative piloting was undertaken until the group were satisfied with the database functionality.
Within the database, lessons are grouped by study and are recorded by: study registration details, stage of study (e.g. Planning and design), process area (e.g. Consent), summary of the event, impact of the event, statement of the lesson learnt and action taken. Lessons are searchable by others using these key items. Each lesson has a blog, which all staff are encouraged to use to make comments, for example, to share similar experiences or suggest solutions. Lessons are reviewed by a core review team on a monthly basis. The review team record their recommendations, request further actions and/or disseminate findings to all research staff. A lesson is closed once all actions and dissemination have been completed. Reports can be printed from the database by the review team which, amongst other functions, provide metrics such as stage of study where lessons are reported, making it easier to identify recurring issues.
**Results**
The 'Lessons Learnt' database has been implemented, with staff starting to record their positive and negative lessons. The review team assesses the reported lessons and facilitates actions required to implement changes needed to improve procedures. Best practice is disseminated to all staff by e-mail, preventing reinvention of the wheel and raising awareness of any changes to processes as a result of learning from lessons.
**Conclusions**
Lessons learnt from past studies can prevent problems being repeated and promote adoption of positive experiences. Keele CTU is in the process of engaging staff fully with the 'Lessons Learnt' process by embedding a culture of sharing and learning using a systematic approach. Future work will aim to measure the impact of the database on the quality of research processes.

### P113

#### Tailoring a comprehensive website to support a clinical research network

Lauren Yesko, Dikla Blumberg, Paul vanVeldhuisen, Jennifer McCormack, Michael Frasketi, Jordan McNeil, Heather Sabina
The Emmes Corporation
**Correspondence:** Lauren Yesko
*Trials* 2017, **18(Suppl 1):**P113

The Emmes Corporation, which serves as the Data and Statistics Center and the Clinical Coordinating Center for the National Institute on Drug Abuse (NIDA)-sponsored National Drug Abuse Treatment Clinical Trials Network (CTN), has recently updated its website with the goal to improve overall study conduct, accessibility to critical information, and overall network cohesiveness and efficiency within the multiple-protocol NIDA CTN environment.

Each active CTN protocol has a designated dashboard which houses critical documents for study implementation and conduct, including training information, official communications, the protocol, the manual of operations, and materials for study medications and/or other interventions. Within each protocol-specific area of the website are comprehensive sets of web reports, updated nightly, that describe important study progress metrics of a protocol implementation status timeline, recruitment, retention, data quality measures, treatment exposure, availability of primary outcome, and regulatory compliance measures, presented in tabular and graphical formats. Summary reports that compare data from each protocol to the a-priori-defined expectations of the sponsor with color codes highlight study performance for the protocol investigative teams and sponsor. Similarly, regulatory reports are developed to display the status of important regulatory documents, identifying expired or soon-to-expire documents such as IRB approvals. These reports allow for continuous monitoring, prompt identification and correction of problems by investigators, site staff, the sponsor, and coordinating centers.
The website also serves as a resource for network-wide activities, including a monthly Web Seminar as a forum for network members to share and exchange clinical research knowledge. This webinar is highlighted on the website and materials are stored for future reference. Committees within the network manage their own dashboard where agendas, minutes, and other communications are posted. There is also a website page known as the investigator toolbox where templates, tools, sample forms, and policies and procedures are stored and serve to guide investigators in the preparation and execution of their clinical trials.
The design and implementation of this updated website for the NIDA CTN has been effective in meeting the needs of both protocol-specific as well as network-wide activities.

### P114

#### NeuroNEXT study design working group experiences

Eric Foster[1], Katy Mahoney[2]
[1]The University of Iowa; [2]Massachusetts General Hospital
**Correspondence:** Eric Foster
*Trials* 2017, **18(Suppl 1):**P114

##### Background
The Network for Excellence in Neuroscience Clinical Trials, or NEURONEXT, is a National Institute of Neurological Disorders and Stroke (NINDS, part of the U.S. National Institutes of Health, or NIH) initiative created to conduct studies of treatments for neurological diseases through partnerships with academia, private foundations, and industry. NEURONEXT is just one of several clinical trials networks supported by NINDS (i.e., strokenet and NETT). In discussions among the members of the networks, it has been useful to share solutions and processes addressing problems that arise in the setting of a clinical trials network. A dialog with researchers external to NINDS-supported clinical trials networks may yield further benefits.
#### Objective
At any given time, there are likely to be multiple proposals for studies to be conducted within a clinical trials network. How these proposals move from an initial concept to a full grant submission varies by network as different solutions are crafted to the common problems of how and when to spend a network's proposal development resources. This work addresses the NEURONEXT approach to proposal development. An overview of the pathway for a proposal is given alongside a discussion of how the process has changed as NEURONEXT has matured. Full protocol development requires spending a great deal of the network's proposal development resources. The decision of when to spend resources on multiple proposals can be difficult with complexity increasing as the number of proposals in the pipeline increases. This work discusses the timing of NEURONEXT protocol development assistance and the corresponding ramifications. Also discussed are several common misperceptions that investigators have experienced during the NEURONEXT design working group process.

### P115

#### Adverse outcomes in non-drug intervention trials: what and how these are recorded

Vichithranie Madurasinghe, Sandra Eldridge, Ayesha De Costa, Anitha Manivannan, Ann Thomson
Queen Mary University London, UK
**Correspondence:** Vichithranie Madurasinghe
*Trials* 2017, **18(Suppl 1):**P115

##### Background
RCTs are an essential part of providing safe healthcare. They should be designed, conducted and analysed according to sound scientific principles to achieve their objectives; should be reported appropriately [1], and should not cause unnecessary harms to trial subjects. As such trial protocols should include a clear safety section, with definitions, procedures and responsibilities for recording and reporting adverse outcomes. The aim of this work is to understand the current practice regards to what and how these outcomes are captured in non-drug intervention trials.
#### Methods
We identified eight non-drug trials from the Pragmatic Clinical Trials Unit (PCTU) administrative database for inclusion. These were selected because their trial documents were available to researchers electronically. Trial protocols, standard operating procedures (SOP) for adverse event reporting and the trial reports (where available) were reviewed. Data were extracted on 1) study design, population and intervention characteristics, 2) stated study objectives and outcomes, 3) data recording, managing and reporting process regards to adverse outcomes and 4) the use of data monitoring committees.
#### Results
All were multi-centre trials involving at least two sites, and conducted in the United Kingdom. Six were individually randomised and two cluster randomised, and all but two were conducted in hospitals or specialist centres. Assessing safety outcomes was a stated objective in two studies and one study included outcome measures on safety, though there were no specific study objectives relating to these. All trials included a section on safety reporting in their protocols, and the procedures for complying with reporting requirements were described in adverse events sops. However, what adverse events were recorded and how these data were captured varied across the studies. Six studies reported capturing data on all "adverse events" (i.e. any untoward medical occurrence as per standard definition), one captured data on related adverse events only and one study was capturing serious adverse events (i.e. Adverse event which is fatal: Results in death, is life-threatening, requires inpatient hospitalisation or prolongation of existing hospitalisation, results in persistent or significant disability/incapacity or is a congenital anomaly/birth defect) only. All used adverse events forms or logs for doing so. Two studies described implementing additional processes for collecting adverse outcomes as supplementary to the main procedure. These included regular data downloads from routine data collection systems, questionnaires and contacts (by telephone, text or email) from research team. Only two studies described how the standard criteria for assessing seriousness were applied in that particular trial. All but one trial described expected serious events in adverse events SOP, but none provided insights into how these were researched or decided upon. Six studies had data monitoring committees in place for assessing safety parameters.
#### Conclusions
All trials attempted to assess adverse outcomes, but there was little evidence of a clear or a consistent approach to doing so; what adverse outcomes were captured, how these were captured varied across trials, and how the expected adverse outcomes were researched and decided upon was unclear. Including these aspects in trial documentation can improve clarity.

### P116

#### Collecting trial quality data about complications routinely: a study within a study to maximize efficiency

Lucy Culliford[1], Emma Hopkins[2], Rachel Maishman[1], Barney Reeves[1], Chris Rogers[1]
[1]University of Bristol; [2]University Hospitals Bristol NHS Trust
**Correspondence:** Lucy Culliford
*Trials* 2017, **18(Suppl 1):**P116

**Background**

At our institution we are establishing a research nurse-led programme to collect in-hospital complication data on all cardiac surgery patients who provide consent. This initiative is part of a wider programme of research to investigate associations between phenotypic and genotypic characteristics with complications and speed of recovery after cardiac surgery.

Data will be extracted from participant's paper and electronic medical notes and collated using a purpose-designed dedicated data collection tool. Information can be obtained by either i) visiting the ward daily, with the opportunity to talk to nurses involved in participants' care, and recording events almost as they happen or ii) extracting data after the patient has been discharged home.

Nurses who collect data for randomised trials believe that method i) takes longer but method ii) results in events being missed. To investigate these beliefs we are planning to randomise nurses (and patients) to collect the data either by method i) or method ii).

**Methods**

Research nurses will be assigned a random selection of patients and told the method of data collection to use for each. All nurses will use both methods (for different patients) and each patient will have their data collected by different nurses using the different approaches. Patients and nurses will be assigned using a balanced incomplete block design to ensure balance across the nurses and by collection method. Each nurse will be blinded to the data collected by another member of the team on the same patient. We propose to compare data collected for completeness as well as the time taken to collect the data.

The study is planned to run over 2 months from December 2016 and will capture data on 96 patients. 72 patients will be reviewed twice, once by each method and 24 patients will be assessed 4 times, twice by each method. Six nurses in the cardiac surgery team have agreed to take part. Each nurse will undertake 40 reviews on 16 patients.

**Results and Conclusion**

The study is ongoing. Full results will be presented at the meeting.

This research was funded by the National Institute for Health Research Biomedical Research Unit in Cardiovascular Disease at the University Hospitals Bristol NHS Foundation Trust and the University of Bristol.

**Disclaimer**

This abstract presents independent research funded by the National Institute for Health Research (NIHR). The views expressed are those of the author(s) and not necessarily those of the NHS, the NIHR or the Department of Health.

### P117

#### Comparing the challenges of the management of trials of medical tests versus randomised controlled trials of health care interventions

Caroline Rick, James Brown, William McKinnon, Ryan Ottridge, Andrew Palmer, Victoria Parker, Lee Priest, Susan Mallett, Jon Deeks
University of Birmingham
**Correspondence:** Caroline Rick
*Trials* 2017, **18(Suppl 1):**P117

Trials of medical tests present a series of challenges that differ, or have differing significance, from randomised controlled trials (RCTs) of interventions. A lack of understanding of the test-specific challenges in the design, set up and management of trials can lead to erroneous results: tests can appear more or less accurate due to inappropriate application of the eligibility criteria and differences in care pathways; study conclusions may be compromised if disease prevalence differs in the study from the actual target population. Birmingham Clinical Trials Unit (BCTU) manages and/or provides statistical support for a wide range of test evaluation trials as well as RCTs of interventions. We have set up a working group to compare and contrast the different challenges of the management of these trial types to improve future trial design and management. The ten test evaluation trials under consideration cover a wide range of medical conditions with different tests being evaluated for screening, diagnosis and monitoring, including: BUS - Accuracy of Bladder Ultrasound (BUS) in the diagnosis of Detrusor Overactivity (DO): a study to evaluate if ultrasound can reduce the need for urodynamics. Egfrc - Accuracy of glomerular filtration rate (GFR) estimation using creatinine and cystatin C and albuminuria for monitoring disease progression in patients with stage 3 chronic kidney disease: Prospective longitudinal study in a multi-ethnic population. ELATION - A randomised trial of the efficacy and cost effectiveness of Real Time Ultrasound Elastography in The Investigation Of Thyroid Nodules and the diagnosis of thyroid cancer. ENDCAP development and evalutions of a biomarker panel to detect enhanced neoplasia in chronic colitis, GBS-2- Accuracy of a rapid intrapartum test to screen for maternal group B streptococcal colonisation and its potential to reduce antibiotic usage in mothers with risk factors MEDAL - MRI to establish diagnosis in women with pelvic pain rockets - Evaluation of diagnostic tools to diagnose ovarian cancer in women referred with symptoms from primary care METRIC - Diagnostic accuracy for the extent and activity of newly diagnosed and relapsed Crohn s disease STREAMLINE-COLON, STREAMLINE-LUNG. Comprehensive staging of newly diagnosed lung and colorectal cancer MROC - Impact of multiparametric MRI on staging and management decisions in women with ovarian cancer.

From initial discussions, common themes of the challenges of test evaluation trials are appearing, including: Challenges of site set up, site finances, patient pathway, recruiting consecutive participants, eligibility biases, obtaining reference standard diagnoses, the role of adverse event monitoring, trial modifications, sample handling and future-proofing sample collections. While some of these themes also occur in RCTs, the relative importance or risks vary. These themes will be explored in more depth and strategies used to resolve or minimise the impact on the project will be reviewed.

### P118

#### Informed consent process enhancement approaches for research participants who may be considered vulnerable populations

Matthew Wright, Julia Lynne, Eric Hardter, Radhika Kondapaka, Kayla Williams, Dagmar Salazar, Dikla Shmueli-Blumberg, Eve Jelstrom, Robert Lindblad
The Emmes Corporation
**Correspondence:** Matthew Wright
*Trials* 2017, **18(Suppl 1):**P118

The informed consent process is paramount to the legal and ethical conduct of clinical trials. Adequately explaining study details to potential participants in a manner that permits comprehension and promotes study retention is challenging; this is compounded when recruiting populations that Institutional Review Boards (IRBs) may view as potentially vulnerable populations, such as persons facing concurrent mental or physical illness resulting in diminished capacity for comprehension, or with socioeconomic issues such as unstable housing, limited educational status, or low functional literacy. Notwithstanding any applicable legal thresholds for a legally authorized representative's involvement, these factors may not negate the participant's autonomy and capacity to make an informed decision on trial participation. In planning studies that involve such potentially vulnerable populations, investigators should consider and implement additional safeguards to ensure that informed consent materials are readily understandable, and that the consent process balances adequate coverage of required elements while avoiding overwhelm for the participant and burden on the research team.

The National Institute on Drug Abuse National Drug Abuse Treatment Clinical Trials Network Clinical Coordinating Center (CCC) and the National Cancer Institute's AIDS Malignancy Consortium (AMC) develop clinical trials for populations that IRBs may consider vulnerable: people living with HIV/AIDS (PLWHA) and/or persons who inject drugs (PWID). Common challenges that CCC and AMC investigators face during the consenting process include designing informed consent materials that communicate required regulatory elements simply yet effectively, engaging the individual in the consent discussion, and assessing comprehension. Methods to be discussed include:

Consent design factors for readability; Ancillary visual and written aids; Consent process standard operating procedures; Patient advocate feedback and involvement; Participant comprehension assessment.
This presentation will highlight these challenges and offer tools and best practices for research teams to develop informed consent materials and augment consenting processes.

### P119

#### Demonstrating the external validity of a randomised controlled trial: the design and generalizability of the protect prostate cancer trial

Jenny Donovan[1], Grace Young[2], Eleanor Walsh[3], Chris Metcalfe[4], Athene Lane[5], Richard Martin[6], David Neal[7], Freddie Hamdy[8], on behalf of CAP and protect study groups
[1]Professor of Social Medicine; [2]RA Statistics; [3]SRA Data management; [4]Reader in Statistics; [5]Reader in Trials; [6]Professor of Epidemiology; [7]Professor of Surgical Oncology; [8]Professor of Surgery
**Correspondence:** Jenny Donovan
*Trials* 2017, **18(Suppl 1):**P119

**Background**
Randomised controlled trials deliver robust and internally valid evidence, but external validity is often neglected. This can limit the relevance of trial findings for routine practice and hinder the implementation of robust evidence. External validity is a complex concept involving reflection on trial evidence in relation to prior knowledge, statistical understanding, biological plausibility and the interpretation of the impact of the trial's eligibility criteria. Design features built into the protect trial of prostate cancer treatments facilitated the assessment of its external validity, and provided an opportunity to consider how trial designs should be used to improve the external validity of trials.
**Methods**
A population-based cluster-trial of PSA screening using a Zelen design created an intervention arm comprising a prospective cohort study of 80,000 men undergoing PSA testing leading to an embedded trial of treatments for localised prostate cancer (protect), and a control arm comprising usual care without formal PSA testing. All men agreeing to participate in the intervention arm entered a prospective cohort study of PSA testing and prostate cancer diagnosis. Socio-demographic, clinical and patient-reported symptom and quality of life data were collected at recruitment and when eligible for prostate biopsies. Men diagnosed with localised prostate cancer who agreed to be randomised, and those who declined and chose a treatment ('preference' group) were followed up in a comprehensive-cohort design. This was extended to include those diagnosed with prostate cancer but excluded from the treatment trial with advanced cancer or excluded other group. We investigated differences in response and/or clinical eligibility at each stage of testing and diagnosis in the prospective cohort study. The characteristics of the randomised men were compared with the preference, advanced and excluded other groups in the extended comprehensive cohort study.
**Results**
The Zelen design produced balanced intervention and control groups. Prospective cohort study participants were more likely to be healthy and from urban and less-deprived areas than non-responders or non-attenders. At subsequent stages in the PSA testing and diagnostic pathway, there were few differences between those eligible or ineligible to proceed, or who continued or declined participation according to socio-demographic and clinical history characteristics. Men who declined randomisation 'preference group' were more likely to have managerial/professional occupations and less deprivation than those agreeing to randomisation. Expected clinical differences were found between the randomised men and the 'excluded other' and 'advanced' groups; 'preference' men were clinically very similar. Conclusion The data from the prospective cohort study and extended comprehensive-cohort study provide detailed information to enable consideration of the external validity of the protect trial. The data suggest that those randomised are broadly representative of the source population, with limitations in relation to the inclusion of more deprived and ethnic group participants. The extended comprehensive-cohort provides assurance that the outcomes of the randomised group will be clinically relevant and widely generalizable. Design features to enhance external validity could be adopted more widely in pragmatic trials to investigate selection issues during eligibility assessment and the clinical relevance of the randomised group.

### P120

#### Informed consent in acute trauma resuscitation: experiences of a major trauma centre

Claire Rourke, Ross Davenport, Karim Brohi
Queen Mary University of London
**Correspondence:** Claire Rourke
*Trials* 2017, **18(Suppl 1):**P120

**Background**
Research investigating the resuscitation and management of unstable trauma patients is essential to improve care and save lives. The decision to take part in a research study is voluntary and the ethical and legal codes that govern medical practice also apply to clinical trials. Informed consent is a vital part of the research process, however, in emergencies this is challenging. In this review the issue of consent in emergency research is presented with emphasis on experiences from a major trauma centre.
**Methods**
Since 2008, all adult trauma patients (16 years) who met local criteria for trauma team activation at The Royal London Hospital have been screened by the Centre for Trauma Sciences Clinical Trial Unit for eligibility into a portfolio of clinical trials. In all trials, agreement to enrol a patient into a trial was obtained using a professional legally appointed representative (PLAR) or nominated consultee (NC), in all cases this was the trauma team leader (a physician independent of the research study). Written consent from the patient or next of kin was sought as soon after enrolment as appropriate by a GCP trained researcher. All studies were reviewed and approved by a Regional Ethics Committee (REC).
**Results**
More than 5000 trauma admissions have been screened for eligibility into the following trials; Activation of Coagulation & Inflammation in Trauma (ACIT –a prospective, observational study), CRYOSTAT (feasibility study, non-CTIMP), itactic (Phase II non-CTIMP), EFIT1 (feasibility study, non-CTIMP), MP4OX (Phase iia & iib CTIMP) and synapse (Phase III CTIMP). Over 1500 patients have been enrolled into ACIT and a further 125 patients recruited into one of the randomised controlled trials (RCT). Following our consent procedures only 70 (5%) of participants in the observational study and 2 (2%) subjects in the combined RCTs were withdrawn due to patient or relative refusal of consent. There was no difference in withdrawal rates between observational studies versus rcts and no discrimination between ctimps and non-CTIMPs. Using PLAR/NC agreement rather than seeking consent from the patient or legally appointed representative, the average time from admission to randomisation in the CRYOSTAT and EFIT RCTs was 13 minutes (0–58 mins, n = 52).
**Discussion**
Less than 5% of total enrolled patients or their next of kin declined consent to continue participation in a research trial. In recent years we have experienced a switch in REC opinion with discussions increasingly involving the use of waiver of consent in such trials to avoid potential delays in delivering an intervention or adversely affecting recruitment rates. Furthermore, feedback from the ethics committees has questioned the procedure used for obtaining informed consent as being potentially distressing and insensitive to relatives at a difficult time. In response to this, our protocols no longer pursue consent from bereaved relatives and the participants are allowed to remain in the study using the PLAR/NC agreement.

### P121

#### Funding innovative study designs - The efficacy and mechanism evaluation programme

Lisa Douet
University of Southampton
*Trials* 2017, **18(Suppl 1):**P121

**Introduction**

To discuss the role of the Efficacy and Mechanisms Evaluation Programme in the funding of clinical research. The research funded by the EME Programme has the potential to make a step-change in the treatment of disease. EME research supports research primarily aimed at establishing clinical efficacy but also embedded within this, the Programme encourages the further understanding of treatment and disease mechanisms.

**Background**

An overview of the EME Programme, in terms of its development, purpose, objectives and vision will be presented. The Programme is seeking to encourage studies which have novel methodological designs that deliver results more efficiently, reduce the study timeline and maximise the knowledge gained. Collaboration from academic, clinical groups, industry and charities is encouraged.

**Methods**

The programme supports translational research evaluating a wide range of novel or re-purposed interventions. The interventions may include diagnostic or prognostic tests and decision-making tools, drugs or biological compounds, psychological treatments, medical devices, and public health initiatives delivered within the NHS. The EME Programme primarily supports clinical trials, and other robustly designed studies that test the efficacy of interventions. The interventions should have the potential to improve patient care or benefit the public.

Innovative study designs involving stratification, the use of routinely collected digital data or novel methodologies are strongly encouraged. Hypothesis-driven mechanistic studies, integrated within the efficacy study that explore the mechanisms of action of the intervention or the disease, the cause of differing responses, or improve the understanding of adverse effects are also encouraged.

**Results**

A wide range of studies have been funded to date and examples of studies with novel study designs will be discussed, along with examples of mechanistic studies and what the Programme means by the term ‘proof of concept’.

**Conclusions**

Studies funded through the EME Programme have the potential to have a considerable impact on the treatment of patients in the NHS. The Programme is keen to drive forward novel or infrequently used study designs that increase the value of the study, by maximising the chances of demonstrating the benefit of an intervention, increasing the knowledge gained or by making the study more efficient.

### P122

#### The UK plasma based molecular profiling of advanced breast cancer to inform therapeutic choices (plasmamatch) trial: a multiple parallel cohort, open-label, multi-centre phase IIa clinical trial aiming to provide proof of principle efficacy for designated targeted therapies in patients with advanced breast cancer where the targetable mutation is identified through ctDNA screening (CRUK/15/010)

Judith Bliss[1], Sarah Kernaghan[1], Charlotte Fribbens[2], Laura Stevenson[1], James Morden[1], Claire Snowdon[1], Iain Macpherson[3], Andrew Wardley[4], Rebecca Roylance[5], Richard Baird[6]
[1]The Institute of Cancer Research Clinical Trials & Statistics Unit (ICR-CTSU); [2]The Institute of Cancer Research; [3]The Beatson West of Scotland Cancer Centre; [4]The Christie NHS Foundation Trust; [5]University College London Hospitals NHS Foundation Trust; [6]Cambridge University Hospitals NHS Foundation Trust
**Correspondence:** Judith Bliss
*Trials* 2017, **18(Suppl 1):**P122

Circulating tumour DNA (ctDNA) is found in the plasma of over 90% of patients with advanced breast cancer (BC). Screening for the presence of mutations in ctDNA provides a current assessment of the genetic profile of the patient’s recurrent cancer. This is important because the mutations present in cancer cells often change over time when the disease spreads to other sites in the body or after treatment. Treatment of recurrent disease is often based on results from primary tumour biopsy as recurrent BC may not be re-biopsied in routine practice. Where a repeat tumour biopsy is performed due to the heterogeneity of cancer the genetic aberrations driving the tumour may not be present in the single biopsied sample to inform treatment decisions. ctDNA screening is more practical and may be more accurate than analysing tumour samples obtained through biopsy, is suitable for all patients including those with inaccessible disease, more economical and more acceptable to patients. Ctdna screening can be carried out in a greater number of patients and could lead to a substantial reduction in the number of patients undergoing invasive biopsies.

Plasmamatch is a multi-centre phase IIa umbrella trial platform consisting of a ctDNA screening component and a therapeutic component. The primary objective is to assess the safety and activity profile of targeted therapies in patients with targetable mutations identified by ctDNA screening. Patients with metastatic or recurrent locally advanced BC who have received prior systemic treatment in the advanced setting will be invited to participate. Consenting patients will be registered for ctDNA screening and a sample of their blood will be sent to the central laboratory for analysis. Patients with a targetable mutation identified will be invited to enter a treatment cohort and consenting patients will receive treatment targeted to the specific mutation identified.

Plasmamatch will be opened across a network of ~50 UK Screening Sites, of which ~25 sites will also be designated as Treatment Sites. Screening Only Sites will refer patients to Treatment Sites for treatment cohort entry and trial treatment administration. After completion of trial treatment patients will be transferred back to the Screening Only Site for follow-up.

Patients with specific targetable mutations identified in tumour sequencing performed outside of plasmamatch will be eligible to enter one of the treatment cohorts, thereby providing a therapeutic option for patients participating in alternative tumour sequencing initiatives. Plasmamatch ctdna screening will also report on mutations to facilitate entry into trials outside of plasmamatch, should the patient not be eligible to enter a treatment cohort in plasmamatch.

The plasmamatch umbrella trial platform is dynamic in design, such that further genetic aberrations or molecular subtypes paired with targeted therapies may be added in the future, providing the addition would not compromise the completion of recruitment to existing cohorts.

Plasmamatch adds to a growing portfolio of trials assessing the utility of ctDNA and will seek to demonstrate the feasibility of ctDNA as a screening tool for patients with advanced BC with the potential for future integration into routine clinical practice.

### P123

#### Practicalities of obtaining long term follow up data from international sites

Sharon Ruddock, Julie Croft, Helen Howard
Leeds Clinical Trials Research Unit
**Correspondence:** Sharon Ruddock
*Trials* 2017, **18(Suppl 1):**P123

Separate long term follow-up studies may be considered where there is interest in longer term outcomes that extend beyond the follow-up period of a trial.

There is a worldwide interest in collecting long term cardiovascular and recurrence/survival data in breast cancer patients with ‘triple negative’ disease. BEATRICE was an international phase III trial assessing adjuvant treatment in ‘triple negative’ early breast cancer, in which patients were followed up to 5 years post randomisation. LOTUS is a separate long term follow up study which is prospectively evaluating longer term outcomes (up to 15 years post BEATRICE randomisation) in this specific population of patients. BEATRICE recruited over 2000 patients across 400 sites worldwide, providing a large, already identified population of potential patients with for LOTUS. The LOTUS trial management group agreed at the outset that recruitment into LOTUS would be limited to patients already recruited to the BEATRICE trial due to the availability of baseline data available from BEATRICE and to utilise the established research network of sites. In order to focus resources and efforts, only BEATRICE sites with 10 or more patients still in follow-up were invited to participate in LOTUS (except in the UK, where all sites were approached). This amounted to 53 sites across 19 countries and still yielded a potential pool of up to 901 patients to reach our planned LOTUS sample size of 250–500 patients.
LOTUS is designed to present minimal burden to aid recruitment of both participating sites and patients for example, patient data is collected annually to coincide with routine clinic visits, or can be collected by phone. BEATRICE patient trial numbers are used in LOTUS for easier data collection at sites, and to enable data linkage between the BEATRICE and LOTUS datasets at analysis. Screening logs for sites were pre-populated for sites to facilitate screening and recruitment of patients. Out of the 53 BEATRICE sites that were approached to take part in LOTUS, 32 sites agreed to take part.
We will present further details on our experience of implementing the LOTUS trial and considerations for design of future longer term follow-up studies.

### P124

#### Telephone support for mail based recruitment and follow-up in a large randomized trial

Fang Chen, Marion Mafham, Lucy Fletcher, Allen Young, Jill Barton, Louise Bowman, Jane Armitage
University of Oxford
**Correspondence:** Fang Chen
*Trials* 2017, **18(Suppl 1):**P124

**Background**
Mail-based recruitment and follow-up is a cost-effective method of conducting randomized trials, but requires telephone support for queries from participants and their doctors. It is important to understand the resource implications of such a telephone service.
**Methods**
ASCEND is a randomized trial of aspirin, and of omega-3 fatty acids, for the primary prevention of vascular disease in people with diabetes. Between 2005 and 2011, 423,403 potential participants were invited and 121,254 returned a screening form. Of these 26,462 participants entered a 2 month placebo run-in and 15,480 were subsequently randomized. Follow-up is by 6-monthly mailed questionnaires (since November 2014 participants can complete their questionnaires online). A Freefone number is available for trial related queries including a 24-hour service for urgent medical issues. A record is made of calls using a standard telephone summary within the trial participant management system. For information governance reasons calls relating to people invited who did not consent to join the study are not included in this analysis. Furthermore, until March 2014, unsuccessful attempts to contact participants or their doctors were not recorded. Therefore, this analysis underestimates the total activity during the trial. For each call, the role of the staff member (medical [including doctor or nurse] or administrative), the individual calling or called (the participant, their GP or other person), the time and date, whether the call reached the intended recipient (for outgoing calls after March 2014) and whether the call occurred after randomization was extracted from the study database. The proportion of successful calls before and after 5 pm was compared using a chi squared test.
**Results**
Up to November 2016, 52,696 calls were recorded. Of these, 8828 were recorded during the run-in period (0.33 calls per run-in participant) and 43,868 after the participant was randomized (0.42 calls per randomized participant per year). Of 16,112 incoming calls to the trial co-ordinating centre, 51% were recorded by medical staff and 49% by study administrators. Of the 36,584 outgoing calls, 24% were made by medical and 76% by administrative staff. Among 20,946 outgoing calls with information about whether the intended recipient was reached, 41% were 'successful'. The success rate was higher for calls after 5 pm compared to those at other times (56% after 5 pm vs 40% before 5 pm; 2 138, $p < 0.001$) irrespective of the participant's age.
**Conclusion**
The telephone support for this mail-based trial requires substantial input from both medical and administrative staff, but allows a large study to be run cost-effectively. Contacting trial participants is more effective outside working hours. These findings have resource implications for those planning similar studies.

### P125

#### Implementation into practice: the development, enhancement and delivery of an online training programme to support clinicians in the replication of the back skills training (best) programme in practice

Helen Richmond[1], Amanda M Hall[2], Bethan Copsey[3], Gill Jones[3], Esther Williamson[3], Zara Hansen[3], Sarah E Lamb[4]
[1]University of Warwick; [2]The George Institute for Global Health, University of Oxford
[3]Centre for Rehabilitation Research, University of Oxford; [4]Centre for Rehabilitation Research, University of Oxford/University of Warwick)
**Correspondence:** Helen Richmond
*Trials* 2017, **18(Suppl 1):**P125

**Objective**
To describe the development and delivery of an online cognitive-behavioural education and training package for health professionals who treat patients with low back pain. The programme is intended support implementation of the evidence-based Back Skills Training programme in clinical practice.
**Background**
The Medical Research Council (MRC) recommends that once the effectiveness of a fully-defined intervention has been established in a definitive Randomised Controlled Trial (RCT), the next phase is to determine whether others can replicate the intervention and results in clinical settings over the long-term. The Back Skills Training (best) programme is an intervention based on a cognitive-behavioural approach for treating low back pain in primary care settings, and was found to be both clinically and cost-effective in a large RCT (Lamb et al., 2010). In order to deliver the intervention, clinicians in the trial underwent a 2-day training workshop delivered face-to-face. Thus, in order to replicate the intervention in clinical settings, the training needed to be available to clinicians. Given the large number of clinicians and patients worldwide that could benefit from using this intervention, face-to-face training was deemed unfeasible.
To ensure scalability and accessibility we translated the face-to-face training into an online format, entitled ibest. We pilot-tested the online training prototype (ibest) for feasibility, acceptability and credibility with health professionals. During the pilot testing, we identified that despite being acceptable, feasible and credible compared to the face-to-face training, clinicians continued to struggle to implement the intervention in routine practice. Building on this evidence-base, we describe how we enhanced the ibest prototype to support clinicians to use the evidence-based best programme in their practice.
**Methods**
We tailored ibest to address implementation barriers using the Theoretical Domains Framework and the Behaviour Change Technique (BCT) taxonomy. The selection of implementation strategies was informed by evidence, clinician and expert opinion and suitability for a sustainable online learning training package.
**Results**
We chose 11 BCTs to target domains of knowledge, skills, beliefs about capabilities, beliefs about consequences, professional role, motivation and goals, and environmental context and resources. The enhanced version of ibest has been accredited with the British

Psychological Society and is currently being evaluated as part of a national level implementation study in the UK.

**Conclusions**

Following guidance from the World Health Organisation, we have described the selection of strategies and how these were used to enhance ibest for implementation. This process will inform the design and evaluation of further implementation interventions and replication of complex interventions found to be effective from Phase III trials.

Lamb et al., Group cognitive behavioural treatment for low-back pain in primary care: a randomised controlled trial and cost-effectiveness analysis. The Lancet, 2010;375:916–923.

Funding

This research was carried out at the Centre for Rehabilitation Research in Oxford and was supported by the National Institute for Health Research (NIHR) Collaboration for Leadership in Applied Health Research and Care Oxford at Oxford Health NHS Foundation Trust. The views expressed are those of the authors and not necessarily those of the NHS, the NIHR or the Department of Health.

### P126

#### A systematic review of the different approaches to intervention development

Katie Sworn[1], Alicia O'Cathain[1], Liz Croot[1], Edward Duncan[2], Pat Hoddinott[2], Katrina Turner[3], Lucy Yardley[4]
[1]University of Sheffield; [2]University of Stirling; [3]University of Bristol; [4]University of Southampton
Correspondence: Katie Sworn
*Trials* 2017, **18(Suppl 1):**P126

Background

Increasingly researchers are developing complex interventions which are evaluated later in randomised controlled trials. Researchers adopt a range of approaches when developing interventions. However, in this rapidly developing field there is little understanding of the rationales, and strengths and weaknesses, of different approaches to intervention development for different contexts.

**Aims**

As part the INDEX study (identification and assessment of different approaches to developing complex interventions), we aimed to review the range of approaches to intervention development and construct a typology to help researchers select the appropriate approach for their context. We also aimed to identify an initial set of core principles and processes of intervention development for use in a future Delphi Study.

**Methods**

We undertook two interlinked systematic reviews. First, we conducted a Best Fit Framework Synthesis by developing an a-priori typology of approaches to intervention development based on the team's knowledge base. For our first iteration, we searched Medline, psycinfo, CINAHL, ERIC and ASSIA from July 2015 to June 2016 for methodological literature and primary studies on intervention development. We then reviewed our typology and for each 'type' identified the rationale for its use, and its strengths and weaknesses. Further search iterations will be used to refine the typology. Secondly, we the undertook Realist Synthesis on each 'type' to hypothesise and test key methodological principles and processes by considering the mechanisms at work to successfully inform intervention development in different contexts.

**Results**

We identified 144 relevant primary study papers from a total of 317 records in the first search iteration. Intervention development papers were international: USA (56%), UK (25%), Europe (5%) and rest of the world (14%). We identified 7 approaches to intervention development ('type' in the typology) including theory-based, participatory, person-based and pragmatic. Each type included sub-types of approaches. For example the theory-based approach included 'Intervention Mapping' (n = 5) and 'The Behaviour Change Wheel' (n = 7). Following an analysis of core methodology papers and books and primary research using each type, an initial core set of principles and processes for developing interventions was identified; these are being refined using further search iterations.

**Conclusions**

We have developed a typology to facilitate intervention developers in selecting the most appropriate approach to developing interventions for their context. This will feed into further parts of the INDEX study - Delphi, consensus workshops and qualitative interviews - to generate guidance for researchers on how to develop interventions.

### P127

#### Treatment success in randomised controlled trials of rehabilitation: a review

Victoria Goodwin, Claire Pentecost, Katie Finning, Angelique Hilli, Jacqueline Hill, David Richards
[1]University of Exeter
**Correspondence:** Victoria Goodwin
*Trials* 2017, **18(Suppl 1):**P127

**Background**

Randomised controlled trials (RCTs) of rehabilitation interventions are generally complex, involve a broad population, multi-faceted interventions delivered in a complex healthcare system, and measure multiple outcomes. The National Institute for Health Research Health Technology Programme (NIHR-HTA) has funded a number of UK trials of physical rehabilitation interventions. To date, no research has reviewed and synthesised the outcomes of this body of work. In this review we aimed to (a) establish the treatment outcomes of RCTs of physiotherapy (PT), occupational therapy (OT), or speech and language therapy (SLT) funded by the HTA, and (b) assess how often those interventions that undergo testing in RCTs result in establishing the effectiveness of new treatments.

**Methods**

We included all phase III superiority RCTs funded by NIHR-HTA from 1997 until July 2016, that evaluated a physical rehabilitation programme of PT, OT or SLT and that had reported their main findings either in a peer-reviewed journal or as an NIHR-HTA monograph. We extracted data on trial design, target population, intervention descriptions, primary outcome(s) and time point(s), any minimally clinical important difference (MCID) identified in support of the sample size, proposed and achieved sample size and between-group primary outcome results with 95% confidence intervals. We categorised primary outcome data into one of six options as described by Djulbegovic et al. (2008): (i) statistically significant in favour of the new treatment; (ii) statistically significant in favour of the control treatment; (iii) true negative; (iv) truly inconclusive; (v) inconclusive in favour of new treatment; or (vi) inconclusive in favour of the control treatment. We summarised the extracted data descriptively.

**Results**

We included 15 studies that recruited 9035 participants, 7834 of whom provided data primary outcomes data, of which five were symptom-based or clinical outcomes, seven were functional measures, two were combined measures and one assessed quality of life. Primary time points varied from immediately post-intervention to one year. Thirteen of the studies utilised a two-arm, parallel RCT design, one used a four-arm factorial design of which only 2 arms related to physical rehabilitation and one was a cluster RCT. The target populations, interventions and settings were diverse. Applying Djulbegovic's classification, four studies were significantly in favour of the new treatment, one was significantly in favour of the control treatment, eight studies had a true negative outcome, one was inconclusive in favour of the new treatment was and one inconclusive in favour of the control treatment.

**Conclusions**

Although most trials reported conclusive findings (13/15) very few interventions tested in these trials achieved superior outcomes compared with controls (4/15). Despite, therefore, a considerable research effort in rehabilitation interventions, there are just a handful of new interventions that outperform existing approaches available for clinicians to use in their routine practice. Even in a situation of genuine uncertainty or clinical equipoise, the reasons why so few experimental interventions in rehabilitation that are brought to trial achieve the results expected of them by trialists and intervention developers requires further investigation so that future trials can be targeted at potentially more fruitful interventions.

### P129

#### Design and analysis of the learning curve and clustering effects in randomised surgical trials - A review of current practice

Conroy[1], J. Blazeby[2], G. Burnside[1], J. Cook[3], C. Gamble[1]
[1]Department of Biostatistics, MRC North West Hub for Trials Methodology Research, University of Liverpool; [2]Centre for Surgical Research, School of Social & Community Medicine, University of Bristol; [3]Nuffield Department of Orthopaedics, Rheumatology and Musculoskeletal Sciences, University of Oxford
**Correspondence:** Conroy
*Trials* 2017, **18(Suppl 1):**P129

Randomised controlled trials (RCTs) are considered the highest level of evidence. Consequently, it is important to ensure that they are designed, conducted and analysed to the highest possible rigour so that the clinical decisions that they are used to inform are valid.
There are many practical and methodological difficulties that a medical researcher must overcome to conduct a successful RCT. In trials that involve a surgical intervention, these difficulties are often magnified. However, there are also additional challenges specific to surgical trials. This work focuses on two highly prevalent challenges:
1. Surgical learning curve 2. Clustering within site and surgeon
Statistical methods have been developed with low uptake to account for the surgical learning curve and clustering effects separately in surgical trials. This research aims to evaluate these and determine when and how to apply them, by considering these methodologies individually and then jointly.
The ultimate aim of this project is to improve the design, analysis, and generalizability of surgical trials. This will be achieved by developing understanding about when learning curves and clustering effects impact on the conclusions of an RCT by determining; the available methodologies and the impact of incorporating these methodologies. This will inform the impact of adjusting for these effects and their impact on interpretation. This will be done considering each effect in isolation and then in combination.
To inform this development, current practice has been established as to how surgical learning and clustering are accounted for in trial design and analysis. A survey to establish current practice and requirements within UK registered Clinical Trials Units has been undertaken. A cohort of published RCTs within medical journals and HTA monographs has been obtained establishing how statistical methodologies are currently being applied and reported.
Results of this work will be presented with how these findings impact development of methods and guidelines.

### P130

#### Machine perfusion in liver transplantation: practical issues to consider in the design and conduct of a complex multinational interventional trial and the potential impact on the analysis

Virginia Chiocchia[1], David Nasralla[2], Ally Bradley[2], Richeal M. Burns[3], Susan Dutton[4], Rutger Ploeg[2], Peter Friend[2]
[1]Centre for Statistics in Medicine (CSM) and Surgical Intervention Trials Unit (SITU), University of Oxford; [2]Nuffield Department of Surgical Sciences, University of Oxford; [3]Health Economics Research Centre (HERC), University of Oxford; [4]Oxford Clinical Trials Research Unit (OCTRU) and Centre for Statistics in Medicine (CSM), University of Oxford
**Correspondence:** Virginia Chiocchia
*Trials* 2017, **18(Suppl 1):**P130

**Background & Methods**
The Consortium for Organ Preservation in Europe (COPE) is conducting several clinical studies, one of which is a multicentre, randomised, controlled, open label trial to compare the efficacy of ex-vivo normothermic machine perfusion (NMP) with static cold storage (SCS) for organ preservation prior to liver transplantation (ISRCTN 39731134). Following confirmation of donor and recipient eligibility, livers were randomised (1:1) to either NMP or SCS, stratified by donor type (donation after brain death (DBD) or circulatory death (DCD) and centre. At the end of preservation, if not discarded, the liver was transplanted in the consented recipient who was managed according to standard local practice and protocols.
Primary outcome of the study is the difference in peak serum aspartate transaminase level (AST) within 7 days post-transplant between the two treatment arms. Secondary outcomes include Primary Non Function, Early Allograft Dysfunction and Patient and Graft Survival.
**Results**
334 livers were randomised with 222 transplanted into recipients from June 2014 to March 2016 in seven European liver transplant centres (UK (4), Spain, Germany and Belgium). Recipients have been followed up for 6 months and the main analysis is currently ongoing.
**Discussion**
Introducing a complex intervention using a non-CE marked medical device into what is already a complex organ retrieval, preservation and transplant process provides many methodological hurdles to overcome. Furthermore, as the trial adopts an international multicentre setting with different regulatory practices in place, several challenges were highlighted during the conduct of the trial which impact directly on the planned analyses, results and interpretation. We will describe the different challenges encountered and how these can be tackled so that it will be of help in the setting of other complex intervention trials in transplantation. The topics in this discussion include: trial design and regulatory affairs, statistical and health economic analysis.

### P131

#### Breaking up is hard to do: letting go of paper case report forms (CRFs) and the challenges of adapting data management processes to remote data entry (RDE)

Paul McGarry
Leeds Institute of Clinical Trials Research (LICTR)
*Trials* 2017, **18(Suppl 1):**P131

The Clinical Trials Research Unit (CTRU) at the Leeds Institute of Clinical Trials Research (LICTR) is a large academic trials unit organised into three divisions; Cancer, Complex Interventions and Comprehensive Health Research. The unit was established in 1991 and now has almost 200 staff running over 30 studies as well as a strong methodological portfolio and other applied health research in each of the three divisions. All of our current studies collect their data on paper CRFs and we receive approximately 240,000 pages of data and manage approximately 12,100,000 data items per year. In 2006 initial attempts at remote data entry proved unsuccessful largely due to poor internet connectivity at many recruiting hospitals and systems that were not intended to be used remotely. With recent improvements in connectivity, systems and more widespread use of RDE, particularly in the commercial setting, a decision was taken within the LICTR to reinvest resource in conducting RDE trials. For the data management team tasked with taking this change forward at LICTR the decision came with a series of demands and challenges to overcome.
This presentation will describe how CTRU adapted its data management processes from data submitted on paper CRFs to data submitted via RDE. Several key topics of this adaptation to RDE will be covered comprising discussion of the central questions behind the change, challenges faced and solutions found. Specifically, these topics will include addressing the background questions, why we delayed the decision to move to RDE, what factors eventually prompted the change and how we might interact differently with sites in terms of training. They will also cover the challenge of replacing Microsoft Excel based data management logs on which data management processes, such as data verification, had often become entirely dependent especially on larger studies. As a solution to these challenges, the design and creation of a data management dashboard will also be discussed. Finally, the presentation will look forward and outline our ideas for further adaptations and innovations from paper crfs to RDE including plans for a site facing dashboard to be used by site research staff and data managers.

### P132

#### Central chart review of complex outcomes

Trisha Boekhoudt[1], Rebecca Clifton[2]
[1]George Washington University; [2]George Washington University Biostatistics Center
**Correspondence:** Trisha Boekhoudt
*Trials* 2017, **18(Suppl 1):**P132

Prior experience has shown the importance of performing central chart reviews of complex outcomes to ensure correct classification. We conducted a randomized controlled trial of 10,000 pregnant women across 16 clinical centers. The primary outcome was any pregnancy associated hypertension associated with serious maternal and infant complications, and the foremost secondary outcome was preeclampsia. Both outcomes had many different criteria (e.g., hypertension severity, proteinuria, thrombocytopenia) that would lead to a diagnosis. Each clinical center completed an Outcome Diagnosis Form that captured each of the elements included in the outcome definitions. One of the more complex criteria was the abstraction of qualifying blood pressures to determine the severity of hypertension as each patient had multiple intrapartum measurements and some of these measurements were deemed too labile to count towards the definition (e.g. During labor or cesarean). This meant the start and end of labor and cesarean were critical to the diagnosis and any inaccuracy in the qualifying time period could result in errors. Due to the complexity of the primary outcome and to confirm the diagnosis of preeclampsia, the protocol subcommittee planned a central chart review for all women whose Outcome Diagnosis Form reported pregnancy associated hypertension. De-identified participant medical records were reviewed by a team which consisted of two medical doctors, a nurse coordinator, and one coordinating center representative. A total of 3,161 charts were reviewed by 21 chart review team members who were blinded to treatment assignment. During the chart review, data elements were discussed and concurrent responses were recorded on a new study form by the coordinating center representative. The adjudicated outcomes were then compared with the original outcomes reported by the site. Discrepancy listings were sent to each of the clinical centers to affirm the review committee decision. There were a total of 553 (17.5%) participants which required changes to the diagnosis of pregnancy associated hypertension and/or preeclampsia. Chart reviews were an important part of the outcome adjudication and resulted in the correct classification of the primary and major secondary outcomes.

### P133

#### Initial experience with a virtual molecular tumor board in a pragmatic precision medicine study

Kaitlyn Antonelli, Pam K. Mangat, Suanna S. Bruinooge, Richard L. Schilsky
American Society of Clinical Oncology
**Correspondence:** Kaitlyn Antonelli
*Trials* 2017, **18(Suppl 1):**P133

The American Society of Clinical Oncology's (ASCO) Targeted Agent and Profiling Utilization Registry Study (TAPUR), a non-randomized, pragmatic precision medicine basket trial, provides a virtual multidisciplinary Molecular Tumor Board (MTB) to support participating clinical sites in identifying the appropriate study drug to target a genomic alteration in a patient's tumor. The TAPUR study offers patients with advanced cancer access to FDA-approved, targeted drugs in non-indicated cancers while capturing safety and efficacy outcomes. Drugs are matched to genomic alterations based on a set of genomic matching rules to help guide the physician's treatment decision. If a proposed drug-variant match is not accepted by the rules engine or the treating physician wishes guidance in interpreting the tumor genomic profile, TAPUR offers a virtual MTB to review cases and identify treatment options.

The MTB is available to all participating sites and meets weekly by webinar. MTB members at each session include at least two clinical oncologists, one molecular pathologist, and one patient advocate. The treating physician or other representatives from the clinical care team also participate in the session. The MTB offers interpretation of genomic test results and identifies potential treatment options either within TAPUR, on other clinical trials, or with other drugs outside of TAPUR.

MTB members for each session are identified from a large pool of experts who have been recruited to participate. Clinical site requests for MTB review are facilitated through the study's electronic data capture (EDC) system. The TAPUR study team is notified of a pending case and materials, including a clinical case summary, genomic test report, and the pathology report for the specimen tested, are prepared and distributed in advance. Following the session, the treatment options with rationale are captured and reported back to the clinical site through the EDC.

To date, 172 patients have been registered to the TAPUR study, and 102 have found a match to a TAPUR study drug and received treatment. Approximately 75% of enrolled participants matched to a study drug through the automated genomic matching rules and 25% of participants matched through the MTB review process. In 8 of the 34 cases reviewed, although no TAPUR drug match could be identified, the MTB provided treatment options for drugs outside of TAPUR and on other clinical trials, if available. One of TAPUR's goals is to describe the concordance of the treatment proposed by the clinical site with the options identified by the MTB. Thus far, proposed treatment options are concordant in 38% of cases reviewed by the MTB.

Some challenges with convening a weekly MTB include staff time involved in the coordination of sessions and variability in reviews due to rotating volunteer participants. To minimize variability in MTB case review outcomes, the study provides training to MTB members, and begins each session clarifying the MTB's role, authority, and responsibilities.

The presentation will discuss the rationale through which ASCO formed the TAPUR MTB, its positive impact on the study, and challenges to date. The governing and guiding processes and workflows will also be described.

### P134

#### Navigating the clinicaltrials.gov database: a case study

Gillian Gresham, Jill Meinert, Stephan Ehrhardt, Lawrence J. Appel, Curtis L. Meinert
Johns Hopkins School of Public Health
**Correspondence:** Gillian Gresham
*Trials* 2017, **18(Suppl 1):**P134

**Background**

The establishment of databases to store information and results about a clinical trial, registered under an index number, has allowed for the identification and tracking of clinical trials. Consequently, the download and analysis of clinical trial information, whether published or unpublished, is possible and has allowed methodologists to take a closer look at characteristics and trends of clinical trials. While many trial databases exist, this report will focus on the clinicaltrials.gov registry. Clinicaltrials.gov became operational in 2000 and is mandated by the United States Government.

**Objective**

Using a case example, we will describe some of the challenges encountered when identifying and analyzing trials registered in clinicaltrials.gov.

**Methods**

We downloaded the clinicaltrials.gov dataset and imported it into two statistical programs (SAS and Stata). We limited the dataset to interventional studies funded by the National Institutes of Health (NIH). All methods and tabulations were performed and compared by two independent authors.

**Results**
In attempts to characterize NIH-funded clinical trials, we encountered: (1) Difficulty importing files into the statistical programs due to formatting issues (e.g., presence of special characters, hard returns, commas, xml text); (2) Misclassification of trials due to misinterpretation of registration fields (e.g., "funder" vs. "collaborator"; "interventional" vs. "observational"); (3) Duplicate counts of same trial as a result "obsolete" NCT identification numbers; (4) Misclassification of trials as to funding source (e.g., trials that were funded by NIH were not listed as being NIH-funded in registry); (5) Missing registration fields (e.g. Number of sites, number of publications). While some of the limitations listed are dependent on how registrants interpret and enter information, there are steps that can be taken to improve registration. One such step would involve improving the editing process to keep files "clean." Providing a test environment for developers to explore data entry options and allowing for the creation of scripts to extract specific data from the website would also be useful. Second, clarifying definitions of registration fields and including examples would help avoid misinterpretation. Furthermore, refining the "other" Categories and using drop-down menus and checkboxes to avoid free-text would reduce incorrect entries and inconsistencies. Third, allowing "obsolete" NCT ids to be filtered out and providing reasons for why the ids were listed as obsolete would avoid double-counting trials. Finally, continuing to expand and update the registry will be important as more trials are registered in coming years, with an ultimate goal of having a worldwide registry for all trials.
**Conclusion**
Trial registries, such as clinicaltrials.gov, have allowed people to access, download and analyze trials data that otherwise would not be possible. However, improvements can be made to increase its usefulness as a tool to describe characteristics of trials and trends over time.

### P135
### Using trainee-led, collaborative research to generate hypotheses for multi-centre clinical trials
James Glasbey[1], Dmitri Nepogodiev[1], Richard Wilkin[2], Dion G. Morton[1], Laura Magill[3], Aneel A. Bhangu[1], Thomas Pinkney[1]
[1]University of Birmingham; [2]West Midlands Research Collaborative; [3]Birmingham Clinical Trials Unit
Correspondence: James Glasbey
*Trials* 2017, **18(Suppl 1):**P135

**Background**
Over the past 8 years, trainee-led surgical research collaboratives have evolved across the UK, giving national coverage across both general surgery and all surgical subspecialties [1]. Several of these groups have undertaken protocol-driven, "Snap-shot" audit and research projects across multiple centres. Patient identification and follow-up periods are deliberately short, facilitating trainee involvement alongside clinical schedules. This approach allows for a large number of patients to be included in less time, prevents repetition, and permits greater generalisability than single-centre studies. The collaborative model of authorship dictates publication of research output under a single, corporate authorship. In the UK, the NHS Research Ethics Committee do not require formal ethical approval for this study type, as they are fully anonymised and do not alter patient care.
The first regional research collaborative within general surgery was the West Midlands Research Collaborative. The methodological expertise acquired within this region has been used to design and conduct a number of observational cohort studies, through the Royal College of Surgeons-funded Birmingham Surgical Trials Consortium. In this project, we collate our progress to date.
**Methods**
Protocols for collaborative studies conducted with the Birmingham Surgical Trials Consortium were collated from group websites, and publication records. Recruitment figures, data points, and validation statistics were extracted from secure electronic records, and published data sets. Data was analysed using descriptive statistics.
**Results**
Nine observational cohort studies in gastrointestinal surgery were run through the Birmingham Surgical Trials Consortium between 2013–2016. Six (66.6%) were conducted internationally, across a total of 92 countries, with the others being UK-only. Study questions spanned topics in surgery and perioperative medicine; surgical site infection, post-operative mortality, analgesia, acute kidney injury, obesity, stoma closure, cholecystectomy and colectomy. A total of 58,500 patient-level records were included, containing 1,308,000 individual data points. The 6 studies that underwent data point validation confirmed a >95% data accuracy, and >90% case ascertainment rate. Hypotheses generated from these studies have directly informed the preparation of two major grant applications; globalsurg Surgical Site Infection Trial (pilot work funded by a Wellcome Trust/ Medical Research Council grant), Modifying Inflammation using Drugs Around Surgery (MIDAS, unfunded), and 3 further grant applications are in progress. All grant applications have included principle investigators from prominent sites contributing to observational data collection.
**Discussion**
We have demonstrated the ability to generate high-quality, patient-level data across diverse settings, generating hypotheses for randomised clinical trials. Validated, observational data serves as high-quality internal pilot, and provides accurate baseline rates to power trial interventions, across specific collaborating centres. By successful submission of data to observational studies, site investigators demonstrate their capacity for research leadership and "Self-select" for involvement in future clinical trials. Corporate authorship flattens the traditional hierarchical model research delivery and publication, and fosters an environment of collaboration for high quality prospective studies and their consequent clinical trials.

**Reference**
[1] Bhangu A, Kolias AG, Pinkney T, Hall NJ, Fitzgerald JE. Surgical research collaboratives in the UK. Lancet. 2013 Sep 28;382(9898):1091–2. doi:10.1016/S0140-6736(13)62013-9. Pubmed PMID: 24075040.

### P136
### Footprints in primary care: using mixed methods to assess fidelity to a complex intervention for frequently attending patients
Rebecca Barnes[1], Helen Cramer[1], Clare Thomas[1], Marcus Jepson[1], Sandra Hollinghurst[1], Sue Jackson[2], Charlie Record[3], Chris Metcalfe[1], David Kessler[1]
[1]University of Bristol; [2]University of the West of England; [3]Frome Valley Medical Centre
**Correspondence:** Rebecca Barnes
*Trials* 2017, **18(Suppl 1):**P136

**Background**
At present the NHS is struggling to meet the demands on the service. The idea for this study originated in a local primary care practice who felt that improvements could be made regarding how it was managing and caring for its most frequent attenders. The idea was developed into a RCGP award-winning intervention consisting of several components including matching eligible patients with a named GP, and training GPs to unpack background psychosocial issues in a contained way during the consultation using the 'BATHE' technique.
**Aim**
The aim of our feasibility study was to explore the main uncertainties to designing a full trial to evaluate effectiveness and cost-effectiveness of this intervention in a pilot cluster RCT involving six practices (4 intervention, 2 usual care). Two of our key objectives were to optimise the content and delivery of staff training to support the intervention; and to assess the extent of implementation fidelity.
**Methods**
As part of the feasibility work, qualitative interviews were held with the stakeholders to build a clear description of the intervention, how it was implemented and expected to work. To ensure a high quality evaluation of implementation fidelity our study design included the collection a diverse range of implementation data (quantitative and qualitative) at multiple time points. Observations of implementer training sessions and of appointment-making between patients and reception staff, were conducted. A varied sample of face-to-face and telephone consultations of GPs using the BATHE technique with patients were video and audio recorded and transcribed in all intervention practices. Conversation analytic methods were applied to assess fidelity to BATHE and the nature and extent of patient response in the consultation recordings Routine monitoring data was collected from practice records to determine how often study patients were being matched with their named gps and patient consultation records were audited for presence of a BATHE code. All quantitative data was analysed descriptively to determine intervention dose and reach.

**Results**

Analysis of electronic medical records data throughout the 12 month intervention period enabled us to monitor dose, reach, provide motivational feedback and document the effects of subsequent implementer trainings. Observations of appointment-making in all four intervention practices and video-recordings of all 12 implementer trainings elicited practical barriers and facilitators that could be addressed, as well as success stories. Conversation analyses of 20 consultation recordings enabled a dynamic assessment of the delivery and receipt of BATHE in situ, that revealed common pitfalls in delivery; specified and added new dimensions to the underpinning theoretical assumptions of the intervention; and provided valuable real-world examples for future training.

**Discussion**

The findings were used to provide tailored top-up trainings, to clarify and help address misunderstandings and problems in implementation and to encourage implementer engagement via whole practice and individual level feedback. Mixed methods were valuable at different timepoints in enabling a full exploration of what might determine the success or failure of a future trial; to optimise training and implementation fidelity; and to understand how and why future participants might resist or engage with the intervention.

### P137

#### When is the best time to internally assess recruitment in a cluster randomised controlled trial?

Lauren Bell, Doris Lanz, Richard Hooper
Queen Mary University of London
*Trials* 2017, **18(Suppl 1):**P137

A common objective of an Internal Pilot is to assess the trial's recruitment rate at a pre-specified time. This is with the aim of reassuring the Trial Steering Committee, Funders and Sponsors that the trial is well received by patients and health practitioners, and is likely to be completed on time and within budget. However, it is possible that a trial's recruitment rate cannot always be assumed to be constant over time. Another issue is that on-going individual recruitment after cluster randomisation will likely introduce selection bias.

For some medical conditions, such as rare, chronic conditions, the majority of patients may already be registered at the site, and there are very few patients presenting or leaving the care of the service. It is possible that assessing the recruitment from the number of patients already registered at each site may tell us almost as much about final recruitment to the trial as if we had waited until the end of the planned recruitment period.

This raises the question: 'When is the best time to internally assess recruitment in a Cluster RCT?' We consider two different stages in a trial's life to execute an assessment of recruitment, through an Internal Pilot Study and an Internal Feasibility Study.

Internal Pilot Study: Randomise clusters, then start recruiting and following up individual participants, and have a go/no-go decision based on the recruitment rate, at a point early in the planned recruitment period.

Internal Feasibility Study: Start recruiting individual participants, assess a pre-specified go/no-go decision point at the end of the planned recruitment period based on recruitment numbers, then randomise clusters and start following up individual participants.

Through the use of Gantt charts, and 3 contrasting research questions recently commissioned by the National Institute of Health Research, we ask which internal study would be the most appropriate for a Cluster RCT. We evaluate this in terms of minimising bias, the confidence of the recruitment estimates, de-risking the investment made into a potentially unfeasible trial, capitalising on preliminary feasibility research, additional information gained (if a sample size re-estimation could also be done), the validity of site registry lists to inform recruitment estimates, and other consequences of the timing in relation to the trial's population needs, intervention delivery and measured outcomes.

### P138

#### Estimating the underlying overall and centre recruitment rates in external pilot trials- how many centre-months of recruitment data do we need? A case study of the BEADS pilot trial

Laura Mandefield[1], Stephen Walters[1], The BEADS Trial Team[2]
[1]University of Sheffield; [2]University of Sheffield & University of Nottingham
**Correspondence:** Laura Mandefield
*Trials* 2017, **18(Suppl 1):**P138

**Background and Aims**

Pilot trials are recommended to estimate certain key parameters for a definitive randomised controlled trial (RCT). The recruitment rate (number of participants recruited in a unit time period) is usually of particular importance as it will inform the number of centres and recruitment period required for a full trial. The BEADS external pilot trial intended to recruit over 12 months at 3 centres (36 centre-months of recruitment) to assess the feasibility of a definitive trial comparing Behavioural Activation Therapy (BAT) and usual care for treatment of post-stroke depression. Due to delays during the trial set up, recruitment was delayed in all centres. A total of 28 centre-months of recruitment were completed. It was suggested that recruitment period should be extended to meet the original target of 12 months per centre. It was decided that the recruitment period would not be extended and we should use the centre-months completed to estimate recruitment rate. We aimed to investigate how many centre-months would be required to estimate the underlying recruitment rate using simulations.

**Methods**

The number of participants recruited to the BEADS trial was recorded and presented by centre and by month. Overall recruitment rate was calculated by centre-month along with the 95% confidence interval. Simulations were carried out to simulate extending the recruitment period up to 80 centre-months. Data were simulated from a Poisson distribution and further simulations were carried out using a negative binomial distribution due to overdispersion in the pilot data. Recruitment rates and their 95% confidence intervals were calculated for each month using the simulated data to examine the precision of estimates from an extended pilot recruitment period. Three values of the mean (1, 2 and 4), the recruitment rate, were used. The moving recruitment rate was plotted against centre-month for each simulation. Percentage bias and percentage coverage were calculated for each centre-month. Bias and coverage were plotted against centre-month to examine how these changed as recruitment period increased.

**Results**

The number of participants recruited to the BEADS trial per month at each centre ranged from 0 to 6; with a mean of 1.75 (1.12, 2.38) per month. Results from the simulations showed that although the level of precision increased as centre-months increased, the level of precision gained by extending pilot recruitment period from 28 to 36 centre-months was not substantial. This was the case for all values of underlying recruitment rate and both the Poisson and negative binomial simulations. Furthermore, the decrease in level of bias and increase in coverage were both relatively small. Conclusions: Although a reduction in the pilot recruitment period was not planned, an extra eight centre months would not have greatly increased the precision or accuracy of our estimate of underlying recruitment rate and its 95% confidence intervals. More general recommendations for pilot recruitment can be made based on our different expected recruitment rates and proposed underlying distributions.

### P139

#### The use of feasibility studies for stepped-wedge cluster randomised trials: a review of impact and scope

Caroline Kristunas[1], Karla Hemming[2], Helen C. Eborall[1], Laura J. Gray[1]
[1]University of Leicester; [2]University of Birmingham
**Correspondence:** Caroline Kristunas
*Trials* 2017, **18(Suppl 1):**P139

**Background**
The stepped-wedge cluster randomised trial (SW-CRT) is a complex design for which many decisions must be made during the design stage, such as the required number and length of steps. Feasibility studies might help to inform these decisions and increase the likelihood of the main trial's success. However, there is currently no guidance on how feasibility studies for SW-CRTs should be conducted. This review, the first in a series of related projects, aims to establish how often feasibility studies are being conducted for SW- CRTs and determine which feasibility issues are currently being investigated. Ultimately this work will lead to guidance on how feasibility studies in SW- CRTs should be conducted.
**Methods and analysis**
Searches for feasibility studies for SW- CRTs were conducted in Ovid MEDLINE, Scopus, and psycinfo. Relevant studies were identified via titles, abstracts and full-text retrievals according to pre-defined study inclusion criteria. Data were abstracted on the aims of these studies and how these studies were able to inform the main trial. In order to also identify unpublished feasibility studies for SW- CRTs, fully published SW- CRTs were identified from the most recent systematic reviews. The authors of these studies were contacted with the aim of determining whether any unpublished feasibility work was conducted prior to the main trial. In addition, the lead statisticians for registered UK clinical trials units were contacted to acquire information on feasibility work that is being undertaken by these units to inform SW- CRTs.
**Conclusion**
This review, which is pending final results, will determine how often feasibility studies are being used to inform SW- CRTs and identify which feasibility issues are being investigated. Any information that is gained on how these feasibility studies have informed the main trials, will allow us to gain an insight into how feasibility studies can benefit SW- CRTs. Future qualitative work will determine which aspects of feasibility studies are considered most useful and what barriers are commonly encountered when conducting a SW-CRT.

### P140

#### Identification of participants for a questionnaire and interview study investigating the feasibility issues encountered during stepped-wedge cluster randomised trials

Caroline Kristunas[1], Karla Hemming[2], Helen C. Eborall[1], Laura J. Gray[1]
[1]University of Leicester; [2]University of Birmingham
**Correspondence:** Caroline Kristunas
*Trials* 2017, **18(Suppl 1):**P140

**Background**
The stepped-wedge cluster randomised trial (SW-CRT) is a complex design for which many decisions must be made during the design stage. If mistakes are made when making these decisions then the trial might prove to be unsuccessful. If the barriers to success are known prior to the trial then a feasibility study can be conducted. Unsuccessful trials are unlikely to be published and so the issues that they faced, which might be common, may go unreported and therefore steps cannot be taken to prevent them from occurring again. We have conducted a review of feasibility studies for SW- CRTs which identified the feasibility issues that are currently being investigated for SW- CRTs. However, further issues are likely to exist given the possible publication bias. We aim to identify conference participants to take part in an online questionnaire which aims to identify further feasibility issues that are encountered by SW- CRTs.
**Methods**
The questionnaire will consist of both closed questions and free-text responses and will be informed by the findings of our review. Participants will be asked about the type of involvement that they have had in SW- CRTs, as well as the issues that they have known SW-CRTs to encounter and those that they are concerned that SW- CRTs may face. These questions will ask the participant either about a particular issue that was identified by our review or will be open for the participant to discuss additional issues that we have not identified.
We are interested in obtaining responses from stakeholders with a wide range of involvements in SW- CRTs. These may be individuals that have been involved in the design, conduct or analysis of SW-CRTs, individuals that are conducting methodological research into SW- CRTs, individuals that have sat on funding panels where decisions have been made on grant applications for SW- CRTs or individuals with any other involvement in SW- CRTs. In addition to conference attendees, invitations to complete the online questionnaire will be sent to the authors of SW- CRTs and feasibility studies for SW- CRTs identified by our review, authors of methodological papers on SW- CRTs, funding panel members, advisors (such as the Research Design Service) and the wider community (from lists of SW-CRT conference attendees, the Allstat mailing list, clinical trials units etc.). The snowball technique will be implemented by encouraging recipients to forward the questionnaire to their contacts who are also involved in SW- CRTs.
Participants with complementary experiences of SW- CRTs may be invited to take part in an interview study that will aim to build upon the findings of the questionnaire study by gaining a greater depth of information on the issues faced by SW- CRTs.
**Conclusion**
The questionnaire and interview studies will gain both a breadth and depth of information on the issues affecting the feasibility of SW-CRTs. These studies form the second part of a series of related projects which will ultimately lead to guidance on how feasibility studies in SW- CRTs should be conducted.

### P141

#### Patient-level information and costing systems (PLICS) as a source of routinely collected cost data for trial-based economic evaluations

Colin Ridyard, Dyfrig Hughes
Bangor University
**Correspondence:** Colin Ridyard
*Trials* 2017, **18(Suppl 1):**P141

**Introduction**
Trial-based economic evaluations rely on a number of methods for estimating resource use and costs. The use of routine data has hitherto been limited, with accuracy of coding, confidentiality, ownership and access having been previously identified as significant barriers to access. The Department of Health in England has recommended the use of Patient Level Information and Costing Systems (PLICS) to understand financial drivers at patient, specialty and hospital levels. These provide an opportunity for estimating secondary care costs within economic evaluations.
**Background**
As part of a randomised controlled trial comparing the use of multiple daily injections of insulin with pumped infused insulin in newly-diagnosed paediatric Type 1 Diabetes Mellitus patients, we investigated the availability and feasibility of PLICS data for estimating diabetes-related hospital inpatient stays.
**Method**
We obtained consent to access patients' electronic records from 15 participating sites. Diabetes-related, patient-level data were recorded, on: HRG codes PA67Z and PA68Z, lengths of hospital stay, total cost and, where available, full disaggregation of PLICS data on items such as critical care and drug costs. Inpatient stays were costed using 3 methods based on: (i) bed days alone; (ii) Payment by results (PBR) National Tariff reimbursement; and (iii) PLICS-reported total cost. Confidence intervals were calculated using non-parametric bootstrap analysis with 10,000 replications.

**Results**

Data relating to 82 hospital admissions were obtained for 74 patients at 5/15 sites. The remaining hospitals (10/15) were still in the process of setting up their PLICS systems and could only provide routine patient admissions or legacy finance database outputs in time for the study. The diabetes-related inpatient stays (N = 67/82 episodes) were comprised of the codes PA67Z (admission related to diabetic ketoacidosis) (12/67) and PA68Z (admission related to diabetes mellitus, without ketoacidosis or coma) (55/67). Mean costs (95% confidence intervals) for the diabetes-related codes were: (i) bed days: £662 (£587, £741); (ii) PBR: £1252 (£1230, £1278); and (iii) PLICS: £1839 (£1339, £2425). Disaggregated PLICS costs comprised medical/specialist nursing staff (47%), wards/overheads (30%), critical care (8%), other clinical supply and services (6%), pharmacy/drug costs (5%), therapies (1%) and pathology (1%) with the remainder comprising blood supplies, imaging, operating theatre and other diagnostic tests.

**Conclusion**

There is no agreed gold standard for estimating inpatient costs for economic evaluations. Reliance on bed day costing alone risks underestimating the total cost of an inpatient stay, especially if the daily rate does not account for staff, critical care, wards and overhead costs. PBR, whilst giving a more accurate cost based on hospital reimbursement, lacks granularity and does not include unbundled costs such as critical care, expensive drug costs and overheads which are reimbursed at a local level. PLICS outputs have sufficient detail to account for these shortfalls and could provide a more robust method of inpatient cost estimation in trial-based economic evaluations, especially where the stay involves additional expensive bundles of care such as long operation times and intensive care admission.

**Acknowledgements**

Funded by the MRC North West Hub in Trials Methodological Research (MR/K025635/1) and the NIHR Health Technology Assessment programme (08/14/39).

### P142

#### Comparison of regression methods for the cost effectiveness analysis of clinical trials with a small population size: the sycamore and folated trials

Giovanna Culeddu[1], Nicky J. Welton[2], Dyfrig Hughes[1]
[1]Bangor University; [2]University of Bristol
**Correspondence:** Giovanna Culeddu
*Trials* 2017, **18(Suppl 1):**P142

**Background**

The Incremental Cost Effectiveness Ratio (ICER) is widely used to inform resource allocation decisions concerning health technologies. However, the ICER is a point estimate and subject to considerable variability. This variability is often presented in Cost-Effectiveness Acceptability Curves (CEAC), which have underlying assumptions relating to both the correlation between the incremental costs and the incremental effects and their distributions. Statistical methods have been proposed to account for this in trial-based economic evaluations. These include non-parametric bootstrapping, and frequentist approaches such as Ordinary Least Square regression (OLS). Bayesian methods such as Generalised Linear Models (GLM) with Normal, Beta and Gamma distribution in cost and effects are scarcely used in trial-based economic evaluation, but may have utility in certain contexts, such as when the patient-level data is non normally distributed, the analysis of the trial is based on a small sample size, and when dealing with imbalanced covariates.

**Objective**

The aim of this research is to explore the applicability of alternative regression methods, determine their precision in calculating the cost-effectiveness analysis of clinical trials, and to assess the merits and disadvantages of each method to reflect uncertainty.

**Method**

Data from two randomised controlled trials are modeled. SYCAMORE is a double-blind, multicentre, randomised controlled trial in which 114 children with severe uveitis associated with juvenile idiopathic arthritis are randomised in a ratio 2:1 to receive adalimumab or placebo in conjunction with methotrexate. Health utilities are obtained from the HUI2 questionnaires completed by the participants or their parents (or guardians). Healthcare resource use and costs are obtained from the patients' diaries and hospital data (PLICS and HES). Folated was a three-centre, double-blind, randomised controlled trial in which 358 patients with moderate to severe depression were randomised on a ratio 1:1 to receive folic acid or placebo in addition to their routine antidepressants. Health utilities were measured with the euroqol EQ-5D-3 L, EQ-VAS and SF-6D questionnaires. Healthcare resource use was collected from patients' self-completed questionnaires, GP records of prescribed medications and hospital data. Frequentist and Bayesian regression methods were employed for the health economic analysis of the trials. Results and robustness of the models were assessed and compared.

**Results**

This research is currently ongoing and findings will be presented at the conference. Discussion Frequentist regression methods are widely used in trial-based economic evaluations of health technologies; however they have several limitations that may be overcome using Bayesian methods. These include factors intrinsic to the clinical trial data, such as population characteristics, study design, sampling methodologies, skewness of cost and utility data, etc. Bayesian methods, however, require their prior belief is generated. This research will contribute to the understanding of approaches for more efficient and robust health economic analysis of clinical trials.

### P143

#### Quantitative benefit-risk modelling of infliximab biosimilar inflectra versus reference product remicade in the treatment of Crohn's disease

Heather Catt[1], Dyfrig Hughes[2], Keith Bodger[1], Jamie Kirkham[1]
[1]University of Liverpool; [2]University of Bangor
**Correspondence:** Heather Catt
*Trials* 2017, **18(Suppl 1):**P143

**Background**

Crohn's disease (CD) is a chronic condition causing inflammation of the digestive tract, requiring ongoing treatment. Biological drugs have improved the quality of life for many patients. However, they are expensive and are linked to serious adverse events. Biosimilar is the term given by the European Medicines Agency (EMA) to a biological drug that contains a version of the active substance of an authorised biological reference medicinal product (RMP). As the patents for RMPs expire, applications are being made for biosimilars. The first CD biological treatment patent to expire was for Remicade (infliximab).

The EMA takes a 'Totality of evidence' approach to assessing the benefit-risk balance by requiring that a comparability exercise demonstrates similarity in terms of quality characteristics, biological activity, safety and efficacy. This involves a stepwise approach to conducting non-clinical and clinical studies. Clinical studies are conducted in one population with extrapolation allowed to other indications given adequate scientific justification.

Sponsor data for infliximab biosimilars has highlighted differences to RMPs vis-à-vis the ability to induce antibody-dependent cellular cytotoxicity (ADCC), which is a potential mechanism of action for anti-TNF-alpha drugs in CD, and the number of adverse events. Whilst regulators in Europe, Japan and Australia approved the biosimilar Inflectra, the USA and Canada were more risk averse, first rejecting the biosimilar before approving following sponsor submission of further explanatory evidence. More recently, an application for another biosimilar, Flixabi, has been approved by the EMA but with a significant divergent position statement.

**Aims and objectives**

To date there has been no approach to measure quantitatively the benefit-risk balance of biosimilars. This project aims to quantify the benefit-risk balance for Inflectra versus Remicade in CD.

The objectives of the study are to:

- Develop a model to estimate the benefit-risk balance of Inflectra versus Remicade.
- Use statistical methods to evaluate the impact of the surrogate outcome ADCC on efficacy outcomes and serious adverse events.
- Carry out, where necessary, elicitation of probability distributions for uncertain model parameters from experts and/or patients.

**Potential impact**

The 'Totality of evidence' approach to benefit-risk balance assessment is subjective and leads to uncertainties. Sponsor-provided data shows differences in the ability of infliximab biosimilars to induce ADCC and there is evidence to support ADCC as a mechanism of action in CD; certolizumab pegol, for instance, is unable to induce ADCC and has minimal efficacy in CD. However, etanercept does induce ADCC but is not effective, adding to the uncertainty.

The main concern with biosimilars is the potential for developing anti-drug antibodies (ADAs), something which clinical trials for other indications are unable to rule out for CD. The consequences of developing adas include reduced efficacy and adverse events, particular serious events that are peculiar to CD including some cancers. The main benefit is the great potential for biosimilars to offer significant cost savings.

This work will identify the conditions that impact the benefit-risk balance for the biosimilar and will provide a methodology that could be developed to assess the benefit-risk balance of future biosimilars.

### P144

#### Health economics analysis plans: the current state of play

Joanna Thorn[1], Colin Ridyard[2], Dyfrig Hughes[2], Sarah Wordsworth[3], Borislava Mihaylova[3], Sian M. Noble[1], William Hollingworth[1]
[1]University of Bristol; [2]Bangor University; [3]University of Oxford
**Correspondence:** Joanna Thorn
*Trials* 2017, **18(Suppl 1):**P144

**Background**

The use of statistical analysis plans (SAPs), produced prior to unblinded analysis, is an accepted means of reducing bias in randomised controlled trials (RCTs) by minimising selective analysis. However, while health economics analysis plans (HEAPs) to guide economic evaluation analysis alongside RCTs are becoming more common, they lag far behind saps in terms of their acceptance and standardisation, and there is a fundamental question over the value they add to trials.

**Aims**

(a) To map current practice and beliefs about the appropriate implementation (or otherwise) of heaps, with a view to drawing up good practice guidelines in future work and (b) to provide a forum in which health economists and other interested parties engaged in economic evaluations could open a dialogue on the need for heaps and methods of standardisation.

**Methods**

A workshop was held in Bristol, UK in October 2015, to discuss issues associated with heaps. 50 predominantly university-based participants heard presentations from speakers before breaking into smaller groups for discussion sessions. Presented sessions included accounts of practical experiences of using heaps in RCTs, alongside perspectives from SAP guidelines, NICE and wider non-trial based economics. In the discussion sessions, participants debated topics including the appropriate content of heaps, the circumstances in which deviations are permissible, and the appropriate oversight and governance of heaps.

**Results and discussion**

Few guidelines are available to aid health economists in compiling heaps. Currently, substantial variation exists in the structure, format and content of heaps, and there are questions over their purpose and appropriate methods of oversight. Heaps may be published as part of a SAP, or as a standalone appendix, but are commonly unpublished. Although concerns remain over the impact of the research and bureaucratic burden involved in producing a plan in advance (particularly given the relatively small health economic workforce), the potential loss of useful post hoc analyses if a plan is too rigid, and the timing of completion, there was a general feeling that heaps would be useful. The majority (approximately 65%) of health economists at the workshop were in favour of a combined SAP and HEAP, rather than a standalone HEAP.

**Conclusion**

HEAPs are currently developed inconsistently and there is an appetite for formal guidance. As it seems likely that the use of heaps will continue to increase in the future (and potentially, be required by funding bodies or regulators), clarity on the appropriate usage and content would be advantageous. We therefore plan to conduct a Delphi survey of practising health economists and other trialists to determine suitable content for a HEAP.

### P145

#### LENS - a clinical trial embedded in routine clinical practice to reduce the burden of diabetic eye disease

David Preiss[1], Jane Armitage[1], John Olson[2], Graham Scotland[3], Graham Leese[4], Helen Colhoun[5], Naveed Sattar[6], Kevin Murphy[1], Jennifer Logue[6]
[1]University of Oxford; [2]NHS Grampian; [3]University of Aberdeen; [4]NHS Tayside; [5]University of Edinburgh; [6]University of Glasgow
**Correspondence:** David Preiss
*Trials* 2017, **18(Suppl 1):**P145

Embedding clinical trials in routine clinical care provides the opportunity to enhance efficiency and reduce costs. Diabetic retinopathy (DR) remains a common cause of blindness and impaired vision and treatment of advanced DR is costly. However, few trials have been designed to investigate treatments which may retard progression from observable DR to advanced DR because the condition usually progresses slowly, it is challenging to identify patients at risk of progression to clinically significant DR without retinal imaging and studies require medical professionals and specialist equipment to capture and grade retinal images. This highlights the need for streamlined trials which can identify large numbers of eligible patients and follow them cost-effectively for extended periods.

The infrastructure of NHS Scotland provides a unique setting in which to conduct a trial with (i) low cost recruitment and (ii) record linkage for assessments of treatment efficacy and safety. Key elements include NHS Scotland's Diabetic Retinal Screening service (DRS) and SCI-Diabetes. DRS provides regular retinal screening for all 250,000 patients with diabetes in Scotland. Retinal images are graded centrally in health boards, results are posted to patients and automatic referral to specialist eye clinics occurs if a patient develops clinically significant DR. SCI-Diabetes is NHS Scotland's diabetes information system which collects and records diabetes-specific data from primary and secondary care.

The LENS (Lowering Events in Non-proliferative retinopathy in Scotland) trial is a streamlined randomized double blind placebo-controlled study of fenofibrate. The aim of LENS is to investigate the effect of fenofibrate on progression of observable DR to either clinically significant DR or to DR which requires specialist treatment. Fenofibrate is a generically available cholesterol-lowering medication and pooled findings from previous cardiovascular outcome trials have suggested that it may reduce DR progression by 30-40%. We aim to randomise 1,060 participants in LENS and follow them for an average of 4 years.

For recruitment, DRS will include a trial information leaflet with a FREEPOST reply slip along with retinal screening results mailed to patients whose DR grading indicates they are eligible (about 10% of all diabetic patients). After two face-to-face visits for screening and randomisation, either side of an eight-week active run-in, follow-up will be conducted remotely. This will involve regular linkage by pseudonymisation to multiple national registries and completion of questionnaires by computer or telephone. Registries will include DRS (for pre-specified eye outcomes), Scottish Morbidity Records (for hospitalisations and outpatient visits), National Records of Scotland (for flagging deaths), the Prescribing Information System and SCI-Diabetes (for various diabetes-related outcomes). Furthermore, SCI-Diabetes will be used after randomisation for central monitoring of biochemical safety in almost real-time, based on the availability of results from routinely collected blood samples. Study medication will be mailed to participants by registered post.
LENS is co-ordinated by the Clinical Trial Service Unit and Epidemiological Studies Unit, University of Oxford, and run in collaboration with the Universities of Glasgow, Aberdeen, Edinburgh and Dundee, DRS and mainland NHS Scotland health boards. It represents the first trial to be embedded within an existing national DR screening program.

### P146

#### Decisions, decisions: bespoke EDC?

Sharon Kean, Jonathan Gibb, Jane Aziz, Ian Ford
Glasgow Clinical Trials Unit
**Correspondence:** Sharon Kean
*Trials* 2017, **18(Suppl 1):**P146

##### Background
Making a decision regarding which system to host your clinical trial data presents a number of challenges. The scope of the system: is it just data entry, is it web enabled, does it include randomisation and is there a requirement for other trial management functionality, has to be considered. The cost of the system is very important to academic clinical trials units (CTUs) not just at the stage of initial purchase but the resource to maintain and setup each trial. As technology develops multiple platform capability is becoming increasingly key to the service a CTU can provide in a competitive landscape.
#### Method
A review of commercial systems, open source solutions versus the merits of developing a bespoke system was undertaken by Glasgow Clinical Trials Unit. A list of requirements was compiled in order to compare each system reviewed and inform the decision. It was also considered whether a combination of systems would fulfil the entire list even if this was not the most elegant solution. The process was concluded by a decision to develop a bespoke system as this was the only solution that could match all of the requirements in a single system.
#### Conclusion
The authors will discuss the implications of this decision on their Unit resource, future business and the justification for their decision. Have they bitten of more than they can chew? Have they developed their perfect system? What decisions have other CTUs made?

### P147

#### Leveraging electronic data capture (EDC) systems to optimize pharmacovigilance in clinical trials

Radhika Kondapaka, Anne Hoehn, Paul Van Veldhuisen, Dikla Shmueli-Blumberg, Robert Lindblad
The Emmes Corporation
**Correspondence:** Radhika Kondapaka
*Trials* 2017, **18(Suppl 1):**P147

Pharmacovigilance (PV), also referred to as drug safety, is defined as the pharmacological science and activities related to the detection, assessment, understanding and prevention of adverse effects or other drug-related problems (WHO, 2002). Monitoring patient safety is an essential component of conducting clinical trials and members of the PV team are highly skilled medical and safety professionals with regulatory and safety experience. However, due to the sheer magnitude of Adverse Events (AEs) collected in many clinical trials, it is vital to utilize innovative EDC technologies in order to improve quality and efficiency of PV activities. Developing the safety electronic case report forms (ECRFs) in a thoughtful and systematic way before the start of a trial can enhance data quality, facilitate coding procedures, and enable more rapid drug safety decisions. Certain adverse event terms can be predetermined on the ECRFs to reduce the number of varied but similar terms, and to categorize into protocol-specific events of interest (e.g., seizures) to allow for desired organization of large amounts of data. This can greatly reduce the preparation time for Data and Safety Monitoring Board (DSMB) review and other reporting needs. Technical capabilities also allow for automated notifications to be triggered based on the use of a specific term, severity grade, or causality designation. For example, the PV team can be notified whenever a "related" AE is entered in the EDC system, or when lab values exceed a certain threshold. Leveraging the EDC capabilities optimizes the function of the clinical trial pharmacovigilance practices, provides real time monitoring of safety events, and can be made accessible for sponsors or DSMB members as needed. This presentation will discuss best practices for utilizing EDC systems to facilitate clean well-organized safety data and increasing the efficiency of pharmacovigilance activities throughout the life of a trial.

### P148

#### Usefulness of in-house electronic health record and task-shifting in a pediatric HIV cohort, Bangkok, Thailand

Torsak Bunupuradah[1], Chavalun Ruengpanyathip[1], Chowalit Phadungphon[1], Yupawadee Jummanee[1], Thanyawee Puthanakit[2], Kiat Ruxrungtham[3]
[1]HIV-NAT, Thai Red Cross AIDS Research Centre; [2]HIV-NAT, Thai Red Cross AIDS Research Centre and Research Unit in Pediatric Infectious Diseases and Vaccines, Faculty of Medicine, Chulalongkorn University; [3]HIV-NAT, Thai Red Cross AIDS Research Centre and Department of Medicine, Faculty of Medicine, Chulalongkorn University
**Correspondence:** Torsak Bunupuradah
*Trials* 2017, **18(Suppl 1):**P148

##### Background
HIV-NAT 015 study is a pediatric HIV cohort, aimed to collect long term efficacy and safety data of treatment in HIV-infected children, established since 2004, at HIV-NAT, the Thai Red Cross AIDS Research Centre, Bangkok, Thailand (clinicaltrials.gov number NCT00476606). In the first few years of this study, we collected clinical and laboratory data through the data entry person after each clinic visits. However, this process took time for data entry, monitoring, and had some transcription errors.
#### Methods and Results
Since 2010, we developed an in-house HIV-NAT electronic health record (EHR) to use in this study. Physician can review all medical history and see overall growth parameters, and real time laboratory results including CD4, plasma HIV-RNA through tables and graphs format. This EHR can capture all adherence data by self-report and percent adherence by pill count of antiviral therapy, HIV disclosure status, parental vital status which is used for adherence counselling and care. The EHR can auto-calculated body surface area after entering body weight and height which minimized error when calculating dosage of antiviral therapy in young children. Physician can directly print the auto-filled prescription after seeing each patient. The EHR is directly integrated to HIV-NAT laboratory reporting systems which reduced workload for data entry and ensuring data safety and completeness. Nurse assistance can process the lab request through the EHR, and make the next clinic appointment in this paperless EHR. Due to limited man-power in number of pediatric study nurse and monitor team, this EHR is helpful in task shifting i.e. A trained nurse assistance can replace some roles of study nurse for administrative work, a trained data entry person can do basic data monitoring by identify missing or out range data. Moreover, we can perform a data search for a new research question and feasibility survey for the new trials within a few minutes.

#### Conclusion
This in-house EHR is efficient, reduces transcription error and illegible data issues, saves time and cost, requires less storage space, and provides a broader set of research questions and future data analysis. We recommend using this in-house EHR in both clinical trial center and in general hospital.

### P149

#### Managing our TEMPER: monitoring triggers and site matching algorithms for defining triggered and control sites in the temper study

Carlos Diaz-Montana[1], Rahela Choudhury[1], Will Cragg[1], Nicola Joffe[1], Nancy Tappenden[1], Matthew R. Sydes[1], Sally Stenning[1]
[1]MRC Clinical Trials Unit at UCL, Institute of Clinical Trials and Methodology, UCL, London, UK; [2]MRC London Hub for Trials Methodology Research, London, UK
**Correspondence:** Carlos Diaz-Montana
*Trials* 2017, **18(Suppl 1):**P149

#### Background
The TEMPER study (results submitted separately) evaluates the use of trial-specific triggered site monitoring, where centrally collected data inform the choice of which sites should undergo monitoring visits. It compared findings at triggered monitoring visits to findings at visits to matched, untriggered control sites that would not otherwise have been visited. Three multi-centre cancer trials at the MRC Clinical Trials Unit at UCL participated in the TEMPER Study.

#### The TEMPER management system
To meet the specified requirements, we developed a data management system to allow: 1) the extraction of tailored trigger data from the participating trials databases; these had been developed in a third-party Clinical Data Management System; 2) the ranking and selection of triggered sites based on the extracted trigger data; 3) the pairing of the chosen triggered sites with control sites based on similarity criteria; 4) the collection and management of findings data gathered in the monitoring visits to the triggered and control sites. The system was developed in Visual Basic.NET with a SQL Server database, which served as the main study database for TEMPER and was used for analysis.

#### Trigger data
For each participating trial, a set of specific triggers was defined by the trial team; each consisted of a narrative explaining the conditions under which it fires. An example of a trigger narrative is: 'More than 0.5% of the values in the open forms are missing or queried based on total number of fields to be entered'. The majority of trigger narratives were implemented as automatic triggers in the system. When data was not available in the trial database to implement an automatic trigger, for example data was from external sources or based on subjective interpretation, manual triggers were instead created in the system allowing users to set their status to fire as necessary. The TEMPER system allowed the trigger data to be summarised following extraction, ranked by a trigger score based on the triggers fired, and presented to the trial teams to inform the selection of triggered sites to visit.

#### Matching Algorithm
A matching algorithm was also implemented in the system which ranked best matches to the selected triggered sites based on site similarity while prioritising sites with lower trigger scores. Site similarity was defined by participation: number of patients randomised and number of days since first randomisation.

#### Results
A total of 38 triggers, 31 automatic and 7 manual, were specified for the three participating trials. The matching algorithm paired 42 triggered sites with corresponding control sites.

#### Conclusions
The TEMPER system implemented a novel approach on how to manage monitoring trigger data and how to match triggered sites with control sites; this allowed the realisation of the TEMPER study protocol.

#### Implications
If the TEMPER study results show the triggered monitoring strategy to be effective, the system could be introduced to other trials in the unit. The TEMPER system design will also inform future implementations and improvements on monitoring systems at the unit, including further research.

### P150

#### Access and security considerations of using EPROMS to collect follow-up data for clinical trials

Jane Aziz, Sharon Kean
Robertson Centre for Biostatistics
**Correspondence:** Jane Aziz
*Trials* 2017, **18(Suppl 1):**P150

#### Background
If patients can be followed up electronically rather than in person, then the cost of running a clinical trial can be significantly reduced. At study design it was estimated that 75% of patients would complete their follow-up online, with the other 25% opting for either postal or telephone.

#### Methods
As the study design allowed for three different methods of collecting patient data, a system was required that would allow very different types of user to access and enter data. These users would require different access levels to the system and to the data being entered. Ensuring patients could only access their own data was imperative, while study staff would need access to the data of all patients that they were in contact with. Additional complexity was introduced, as the staff creating, maintaining and supporting the system never deal with patients, so needed to remain blind to the patient's personal contact details.

#### Conclusion
We will discuss the different levels of access required for the different roles, how access was provided to patients and the challenges in creating a system to be used by patients, while remaining blind to their identity.

### P151

#### Using OWL to classify adverse events

William Stevens, Karl Wallendszus, Salvador Girbau, William Herrington, Michael Lay, Martin Landray
University of Oxford
**Correspondence:** William Stevens
*Trials* 2017, **18(Suppl 1):**P151

#### Background
For defining clinical trial endpoints and for producing reports, it is necessary to group adverse events. For example a line reporting the number of participants who had a stroke includes participants who had "Haemorrhagic Stroke", "Ischaemic Stroke", "Cerebral Infarction", and several other types of stroke. Classification of events into groups can be done using the event code, but often other properties also need to be used. These properties include: "hospitalization required", "cause of death", "urgent or non-urgent". Large trials can have 10,000 s of events, and hundreds of event classes. Defining event classes and classifying events are important problems.

#### Methods
We describe how to use the Web Ontology Language (OWL) to address these problems. OWL software tools exist that can represent OWL in both human-readable and machine-readable forms; an OWL document defining event classes can serve as both specification and implementation. OWL permits a class to be defined as a Boolean combination of other classes. For example STROKE can be defined as HAEMORRHAGIC STROKE or ISCHAEMIC STROKE. Medical coding dictionaries such as meddra can serve as predefined event classes. We have converted meddra into an OWL representation so that it can be used as the basis for defining other event classes. We deliberately use only those features of OWL that correspond to basic set theory because this is already understood by clinicians who define event classes, and programmers who use them. During the process of defining event classes, OWL tools can be used to check for errors. For example, it is possible to specify that two event classes should have no events in common, or that a group of event classes should be equivalent to some other event class. In order to use OWL event class definitions during the production of analyses and reports, some short 'glue' programs are required. Events (and their properties) that occur during the trial are converted into an OWL document and fed into an OWL reasoning tool. The output from this is a list of which event classes contain which other event classes. This is converted into a flat tabular form that can be stored in a database and easily queried to find out which events are in which classes.

**Pros**

This approach uses well-supported public domain software, so little in-house coding is required. Since OWL has a well-defined meaning, event class definitions can be communicated unambiguously to others. Performance is reasonable: in a large trial (85,000 events, 200 event classes) it takes a few minutes to check class definitions for inconsistencies, and one minute to classify all events.

**Cons**

It is sometimes necessary to take account of the open-world semantics of OWL in order to define some event classes correctly; it is reasonable to expect programmers to take the time to understand this, but not so reasonable to expect this of clinicians.

**Conclusion**

We found this to be a practical way of classifying events. We expect that OWL can be used for other classification tasks, such as the classification of drugs into related groups.

### P152

#### Embedding randomized clinical trials within registries: how feasible?

Pamela Tenaerts[1], Steven J. Mikita[2], Sara B. Calvert[1], Christopher Dowd[3]
[1]Clinical Trials Transformation Initiative; [2]Patient Advocate; [3]Cystic Fibrosis Foundation
**Correspondence:** Pamela Tenaerts
*Trials* 2017, **18(Suppl 1):**P152

**Background**

A Registry is an organized system that uses observational methods to collect uniform data on specified outcomes in a population defined by a particular disease, condition or exposure. At their core, registries are data collection tools created for the purpose of generating clinically usable information and evidence. The data captured in a registry typically includes information such as medical history, demographics, disease diagnosis and outcome data.

Patient data collected in registries often overlaps with data gathered for clinical trials. Integrating clinical trials within observational data registries may offer opportunities to avoid duplicative data collection, increase operational efficiencies, reduce time to database lock and accelerate time to critical decision making, while decreasing clinical trial costs. The objective of the Clinical Trials Transformation Initiative (CTTI)'s Registry Trials project was create recommendations to support the practice of leveraging registries to facilitate high quality clinical trials.

**Methods**

The CTTI Registry Trials project team conducted a literature review, interviewed 25 experts, and then convened a multi-stakeholder expert meeting. At the expert meeting attendees discussed recommendations for best practices to increase the value, acceptance, and success of registry based clinical trials.

**Results**

Depending on its characteristics and capabilities (e.g. Interoperability, connectivity, flexibility, sustainability), a registry can be used either as an observational data source for generation of clinically actionable evidence and hypothesis generation, or as a critical reusable component of the clinical trial infrastructure within which prospective randomized studies can be performed. Questions exist about identifying appropriate registries, ensuring data quality/comparability, meeting regulatory requirements, and processes for implementing a randomized registry trial.

If the historical data created by a registry are well established to be relevant/fit to purpose, robust and reliable, then the registry can have a clear role in creating a sustainable infrastructure within which regulatory trials can be conducted. Identifiable requirements and practical considerations have not been defined in the use or modification of existing registries and/or in design of new registries in order to make them fit for the purpose for conducting a Randomized Registry Clinical Trial (RRCT). Such normative standards are essential for consistent evaluation of a registry's suitability for generating the clinical evidence needed for regulatory decision making in the various phases of drug and device development. CTTI has created recommendations and tools to assist in 1) evaluation of an existing registry's suitability for conducting clinical trials and 2) designing a new registry in which to conduct a clinical trial.

**Conclusions**

CTTI Registry Trials recommendations and tools can assist researchers in evaluating new and existing registries to determine if embedding randomized clinical trials is appropriate.

### P153

#### Developing novel endpoints, generated using mobile technology, for use in clinical trials: a clinical trials transformation initiative (CTTI) project

Martin Landray
University of Oxford
*Trials* 2017, **18(Suppl 1):**P153

The appropriate inclusion of mobile technology into clinical trials offers significant opportunity to improve clinical endpoint ascertainment. Mobile technology can provide unprecedented access to real-world situations where multiple precise measurements on trial participants could be made without interference to their daily life. Such endpoints can reduce patient participation burden, increase trial feasibility and address unmet need for endpoints in certain therapeutic areas and patient populations. We propose recommendations to clarify the pathway for developing novel endpoints, generated using mobile technology, for use in clinical trials. We describe steps for appropriate novel endpoint selection and development, along with an analysis of how this approach differs to traditional endpoint development and recommendations for reducing friction in this process. Our approach is designed to meet the needs of clinical trialists, regulators and trial participants. Our multi-stakeholder team of experts pursued a two-pronged approach to evidence gathering. Our recommendations have been informed by both new, empirical evidence generated by writing four use cases and a synthesis of existing, published approaches across therapeutic areas. Four discrete use case teams developed proposed novel endpoints for Parkinson's disease, heart failure and Duchenne's muscular dystrophy using data generated using accelerometers and a novel endpoint for diabetes derived from data from a continuous glucose monitor. Each team included investigators and patient reps with expertise and experience in the disease state, engineers and mathematicians with expertise in the specified device, regulators, nonprofit consortia and statisticians. The systematic literature review identified 101 manuscripts where novel endpoints were included in clinical studies.

### P154

#### Matching the right patient to the right clinical study: the opportunities for more efficient clinical studies using selective-recruitment designs

James Barrett, Catey Bunce
King's College London
**Correspondence:** James Barrett
*Trials* 2017, **18(Suppl 1):**P154

**Background**

Big databases of patient health information offer a unique opportunity to be selective about which individuals are invited to participate in clinical studies. In particular, large databases can be searched for the "most informative" individuals. Conventional study designs typically recruit individuals at random, usually by waiting for eligible patients to present at one of the study centres. Electronic health records allow researchers to immediately target the most relevant and informative patients for further investigation.

**Methods**

Informativeness depends on an individual's covariates (or "risk factors"). The statistical measure of entropy is used to quantify how much statistical information an individual is expected to provide on the study. More informative individuals have a higher probability of being selectively recruited onto the study.

**Results**

Preferential recruitment of informative individuals can potentially lead to successful observational studies with smaller cohort sizes. Results from numerical simulations have shown that a desired level of statistical power can be achieved with an informative cohort of 200 individuals compared to a randomly selected cohort of 300 individuals. In order to illustrate how our methodology can be used in practice we simulated studies of patients with cardiovascular disease using electronic health records from the Clinical Practice Research Datalink.

**Conclusion**

The advent of large databases of patient records represents an unprecedented opportunity for more efficient and cost effective studies. Our approach can also be applied to follow-up studies after a clinical trial has ended in which an informative subset of the trial participants are followed for a longer period of time. In situations when it is prohibitively expensive to follow the full trial cohort over an extended time period our methodology may offer a more feasible alternative.

### P156

#### Vibrating vaginal balls to improve pelvic floor muscle performance in women after childbirth: a randomised controlled feasibility trial

Claudia Oblasser[1], Christine McCourt[1], Engelbert Hanzal[2], Shashivadan P. Hirani[1]
[1]City, University of London; [2]Medical University of Vienna
**Correspondence:** Claudia Oblasser
*Trials* 2017, **18(Suppl 1):**P156

This abstract is not included here as it has already been published.

### P157

#### Patient and public involvement: an investigation

Lucy Selman, Clare Clement, J. Athene Lane, Chris Metcalfe, Joanne Simon, Jeremy Horwood
University of Bristol
**Correspondence:** Lucy Selman
*Trials* 2017, **18(Suppl 1):**P157

**Background**

There are ethical, sociopolitical and scientific reasons for patient and public involvement (PPI) in research, and many funders now require applicants to include PPI to improve the relevance, accountability and quality of research. However, there is evidence of challenges in implementing PPI, and formal guidance on how to involve service users in the conduct of trials is lacking.

**Aim**

To systematically investigate how PPI is approached within the Bristol Randomised Trials Collaboration's (BRTC's) clinical trials unit (CTU) portfolio of trials, and identify barriers to and facilitators of its successful implementation, to contribute to our understanding of PPI in trials and guidance and training in this area.

**Methods**

We included all currently active BRTC portfolio trials, plus those which ended in the previous 2 years. A mixed-methods study design was developed, involving: (1) An online survey of Trial Managers (TMs), conducted August-September 2016, to determine how trials included PPI and the support required from CTUs for PPI; (2) Interviews with Trial Management Group members and PPI representatives from case study trials (estimated n = 10). Case studies were selected purposively to represent a range of trial designs, funding streams and trial initiation dates. Interviews explored the perceived value of PPI involvement and barriers to/facilitators of PPI. Quantitative survey data were summarised using descriptive statistics and interview transcripts analysed thematically. A project PPI group advised on interview topic guides, provided feedback on findings and assisted with dissemination.

**Results**

21/26 TMs completed the survey. 15 trials (71%) included a PPI representative on the trial team, 5 used another method of PPI (e.g. Via input to advisory group (n = 3)/Trial Steering Committee (n = 2), consultation of patient group (n = 1)), and one TM reported s/he did not know of any PPI in the trial. The 15 trials that included PPI representatives recruited 1–20 PPI representatives (mean 6.4, 1 missing), but in practice 0–10 were regularly involved (mean 4.8, 2 missing). None used a formal process to recruit PPI representatives.

The most common tasks undertaken by PPI representatives were review of participant-facing materials and other study documents and advising on recruitment/retention strategies. Changes made as a result of PPI related to trial documentation and design; 2 TMs reported no changes had been made. Twelve TMs reported that PPI representatives were paid for their time, 4 said payment was not offered and 3 did not know. Payments ranged from £10-£50/hour, with 3 trials paying in vouchers. Only 5 TMs reported that training was provided for PPI representatives (12 reported no training, 3 did not know, 1 missing).

TMs reported that CTUs could assist with recruitment of PPI representatives and provide guidance on integrating PPI in grant applications. Challenges reported included 'professional PPI members' having a different agenda to the study population, and lack of continuity in trial staff. The interviews are currently underway; findings will be presented at the conference.

**Conclusions**

Survey findings show that PPI involvement in trials is currently highly variable. PPI representatives are recruited informally, are rarely provided with any training, and are paid inconsistently across trials.

### P158

#### 'Is that it?' Using 'explorachoc' to engage the public with clinical trials and health services research

Heather Morgan, Becky Bruce, Gordon Fernie, Heidi Gardner, Beatriz Goulao, Joanna Kaniewska, Clare Robertson, Sharon Wren, Katie Gillies
University of Aberdeen
**Correspondence:** Heather Morgan
*Trials* 2017, **18(Suppl 1):**P158

**Background**

Public engagement is the interface between research and wider society. By helping to bring these two sometimes disparate worlds together, engagement can increase public trust and enhance relevance, accountability and transparency of, and in, research process(es) and researchers. Engagement is also important because it can empower people to become involved through offering their insights into and feedback on our work to help ensure that the research we conduct is relevant to the societies in which we live and are striving to improve. Bringing together colleagues' experiences of, and interest in, public engagement with research, our team's aim was to employ a strategic approach to deliver public engagement activities around clinical trials and health services research to demystify our work and facilitate public involvement.

**Methods**

We designed a two-arm trial, which we called 'explorachoc' to: demonstrate the randomisation process used in clinical trials; engage members of the public in conversations about clinical trials and health services research to solicit their perceptions and views; and recruit to a public involvement panel. We piloted this activity at the University of Aberdeen's May Festival (28–29 May 2016) and ran a modified version as part of the University's European Researchers' Night/Explorathon event (30 September 2016). The trial involved: double-blinded selection of either a blue or yellow ball (with equal chance of selecting either); depositing the ball in a large transparent jar to demonstrate the distribution of selection; ringing a randomisation bell; being given a chocolate with a blue or yellow (white or milk chocolate) wrapper, corresponding to the selected ball's colour; and ranking the chocolate on a scale of 1 (low) - 4 (high). We then engaged participants in a range of conversations about the history of clinical trials (using costumes and props to re-enact James Lind's scurvy trial) and our research portfolio (supported by flyers and printed information). Finally, we asked participants whether they would be willing to be contacted to contribute to our public involvement panel.

**Results**

We randomised 365 people (48.5% blue) across the two events (83% Explorathon). The median in the blue group was 4.0 Interquartile range- IQR (3.0-4.0) and 3.5 IQR (3.0-4.0) in the yellow group (Mann Whitney U p-value = 0.633). The resounding response was 'Is that it?' Regarding randomisation, which suggests that we were able to somewhat demystify the process of randomisation. Most participants understood the connections between clinical trials, health services research and the health and social care they receive. Many recounted anecdotes of surgical, drugs and other therapies they have experienced themselves or within their families and acknowledged the importance of engaging and/or being involved in the types of research we do. 71 people volunteered their details for future contact regarding public involvement.

**Conclusions**

We have established an approach to delivering public engagement activities around clinical trials and health services research designed to demystify our work and facilitate public involvement. We have a proof of concept for an effective engagement model, enhanced by the use of chocolate, and are building on this to develop public engagement and involvement strategies.

### P159

#### Exploring the impact of priority setting partnerships in skin disease

Joanne Chalmers, Natasha K. Rogers, Kim S. Thomas
University of Nottingham
**Correspondence:** Joanne Chalmers
*Trials* 2017, **18(Suppl 1):**P159

**Background**

The purpose of conducting a Priority Setting Partnership (PSP) is to identify and prioritise uncertainties for a specific condition. PSPs usually follow a standardised transparent methodology, often supported by the James Lind Alliance (JLA). They are a true collaboration between clinicians and patients/carers. It is expected that the results of a PSP will influence the subsequent research agenda for that condition, ensuring that research meets the needs of both patients and clinicians and therefore contributes to reducing research waste. The overall aim of this study is to assess the impact on the research agenda of all PSPs that have been conducted for skin conditions.

**Objectives**

We will determine what proportion of clinical trials address one or more of the prioritised research uncertainties, and whether there are differences between different sponsor/funding types. We will also investigate what proportion of the National Institute for Health Research (NIHR) commissioned calls and themes reflect the priorities identified by PSPs. Furthermore, we will look at the wider picture of impact of PSPs, including how findings can be used to support non-research activities (e.g. Services), development of diagnostic criteria and outcome measures, and whether PSPs can act as a gateway for individual patients/cares to progress to further involvement in research.

**Methods**

We will search relevant databases and websites to identify all skin-related PSPs that have been conducted, and then identify ongoing or published clinical trials in these disease areas. Sources will include clinical trial registries, funder databases, PubMed, and the JLA website. Where necessary, we will contact the clinical trial teams to clarify where information is unclear, particularly around timing, to determine whether or not the trial was funded prior to the PSP results being available. We will also survey the authors of PSPs and subsequent research teams to assess any wider impact of the PSPs such as successful fellowship applications and involvement of patients as partners in further research.

**Results**

Preliminary searches have identified seven PSPs in the field of skin disease; acne, cellulitis, eczema, hair loss, hidradenitis suppurativa, pressure ulcers and vitiligo. Work on this study is ongoing and the full results will be available by the time of the conference.

**Implications**

The results of PSPs are increasingly being used by funders to prioritise research of importance to both patients and clinicians. However, it is acknowledged that conducting a PSP requires considerable time and resources, so we will evaluate the value of PSPs by assessing the evidence of impact of those conducted in dermatology. The results of this study should therefore be helpful for researchers considering undertaking a PSP, and for potential funders of PSPs.

### P160

#### REPORT-UK (real-time electronic patient outcome reporting of adverse events in UK cancer trials): methods to optimise data collection

Jane Blazeby[1], Fiona Kennedy[2], B. Clayton[2], Kate Absolom[2], Elaine O'Connell Francischetto[1], Louise Flintoff[3], K. Gordon[2], W. Crocombe[2], F. Samy[2], V. Hiley[2]
[1]University of Bristol; [2]University of Leeds; [3]University Hospitals Bristol NHS Foundation Trust
**Correspondence:** Jane Blazeby
*Trials* 2017, **18(Suppl 1):**P160

**Background**

Adverse events (AEs) reporting is essential in clinical trials. The current system for reporting (Common Toxicity Criteria and Adverse Events, CTCAE) relies on clinicians' interpretation of symptoms. The value of patient self-reports of AEs and Patient Reported Outcome Measures (proms) is recognised but robust data collection methods are needed. Here we report the REPORT-UK study which developed and evaluated an electronic (internet/telephone) system for self-reporting AEs and proms during trials.

**Method**

249 varied diagnosis cancer patients undergoing treatment (chemotherapy/targeted agents/hormone therapy/radiotherapy/surgery, and an ECOG group with performance status 2) were recruited. For 12 weeks patients were reminded (text/email) to complete weekly AEs (NCI PRO-CTCAE) and monthly proms questionnaires (EORTC QLQ-C30) on their preferred system. Acceptability and feasibility was measured by recruitment rates, attrition, compliance, and patient and staff feedback at end-of-study.

**Results**

Overall, the consent rate was 48%. System preference was 82% internet/17% IVR (telephone). Only 13 participants withdrew and 6 died whilst on study. 192 returned end of study questionnaires. Overall patient compliance was good for weekly AE and monthly proms reporting, but differed between treatment groups, and dropped over time. Both systems were perceived as easy-to-use. Time to complete was perceived by patients to be acceptable, although actual times show the internet is quicker (median time 9 minutes vs. 21.5 minutes). Baseline comparisons between patient vs. Clinician-reporting of some AEs differed substantially.

**Conclusion**

The study demonstrates a user-friendly electronic data collection system, which provides information on patient compliance in a general oncology setting but we recognise this is different to a real trial setting. The system could be implemented in practice in clinical trials alongside traditional approaches to improve data quality and safety.

### P161

#### Tocilizumab in pulmonary hypertension

Jules Hernandez-Sanchez, Mark Toshner
Papworth Hospital
**Correspondence:** Jules Hernandez-Sanchez
*Trials* 2017, **18(Suppl 1):**P161

**Objective**
Bayesian estimation the proportion of pulmonary arterial hypertension patients with a 30% reduction in pulmonary vascular resistance from baseline after Background Pulmonary arterial hypertension comprises a grouping of diseases associated with a poor prognosis. Four classes of drug therapy targeting vasoactive pathways have been studied in randomised controlled trials (RCTs) and licensed for the treatment of predominantly group 1 pulmonary arterial hypertension (PAH). These therapies have demonstrated moderate success, with meta-analyses of all RCT data suggesting a short-term improvement in mortality at 14 weeks [1]. Despite this, PAH in the UK still carries a 5 year survival in idiopathic PAH of 61% and as low as 49% for PAH associated with connective tissue diseases. Therefore, there remains an urgent need for the development of new treatments, particularly as the results from combination studies of these different classes of vasoactive therapies has been to date mixed and disappointing.
**Methods**
The study will be a 6-month open label phase II trial of IV tocilizumab (8 mg/kg) in 21 patients with group I PAH. Subjects will be assessed for safety and efficacy at screening, baseline, week 4, week 8, week 12, week 16, week 20 and study end. The main outcomes are: safety (incidence and severity of adverse events) and pulmonary vascular resistance (dynes's Cm-5) measured using invasive haemodynamic assessment by right heart catheter. Prior elicitation techniques will be used to transform experts' knowledge about the effect of the drug onto a distribution. Bayesian analysis will take into account the experts' prior to predict the mode and 95% credible interval of the effect of tocilizumab in pulmonary vascular resistance [2].
**Results**
This is an ongoing project. Potential posterior distributions given different priors (dashed lines) and any possible result.
**Conclusions**
Prior elicitation is particularly useful in small trials because the amount of information contain in the data is limited. However, the prior can have strong effects on the posterior so removing biases in the elicitation process is paramount.

**References**
1. Galie N, Manes A, Negro L, Palazzini M, Bacchi-Reggiani ML, Branzi A. A meta-analysis of randomized controlled trials in pulmonary arterial hypertension. Eur Heart J. 2009 Feb;30(4):394–403. Pubmed PMID: 19155250. Pubmed Central PMCID: 2642921.
2. Anthony O'Hagan, Caitlin E. Buck, Alireza Daneshkhah, J. Richard Eiser, Paul H. Garthwaite, David J. Jenkinson, Jeremy E. Oakley, Tim Rakow (2006) Uncertain Judgements: Eliciting Experts' Probabilities. Wiley.

### P162

#### A comparison of adjudication approaches for clinical trials

Brennan Kahan[1], Vipul Jairath[2]
[1]Queen Mary University of London; [2]Department of Medicine, Epidemiology and Biostatistics, Western University
**Correspondence:** Brennan Kahan
*Trials* 2017, **18(Suppl 1):**P162

**Background**
Many clinical trials involve binary outcomes which require adjudication to determine whether an event occurred or not (e.g. Myocardial infarction, disease progression, patient response, etc.). Incorrectly classifying the outcome (e.g. By incorrectly adjudicating an event when the true outcome was no event, and vice versa) can lead to biased estimates of treatment effect and reduced power. Using adjudication approaches which minimise the misclassification rate of outcomes is therefore important, however there is little evidence on which to base this decision.
**Methods**
Under the assumption of non-differential assessment (i.e. That misclassification rates are the same in each treatment arm, as would typically be the case when outcome assessors are blinded), we addressed three questions about adjudication: (a) How many assessors should we use? (b) When is it better to use on-site or central assessment? And (c) Should central assessors adjudicate all outcomes, or only suspected events?
**Results**
We found that no one adjudication approach worked best across all situations. The best approach will depend on specific trial characteristics, mainly the misclassification rates of the site and central assessors, and the correlation between assessors. In general, there will rarely be much benefit to using more than three assessors, unless the correlation between assessors is extremely low; for outcomes with very high correlation between assessors, using one assessor should be sufficient. Both site and central assessors can be appropriate, and the best choice depends on which type of assessor has lower misclassification rates. Using a combination approach in which both the site and central assessors are involved in adjudication may be useful when misclassification rates are unknown. Having central assessors adjudicate only suspected events will typically increase bias, unless the threshold for sending suspected events to the central assessor for adjudication is extremely low.
**Conclusions**
No one adjudication approach works best across all situations. Trialists should choose the most appropriate adjudication approach based on the specific characteristics of their trial.

### P163

#### A multi-centred study validating a bleeding assessment tool (BAT) developed by the biomedical excellence for safer transfusion (BEST) collaborative for use in adult patients with haematological malignancy

Claire Dyer
NHS Blood and Transplant
*Trials* 2017, **18(Suppl 1):**P163

**Background**
Many platelet transfusion trials now use bleeding as a primary outcome. There are two important considerations when bleeding is used as an outcome measure: how signs and symptoms of bleeding are documented and the translation of this information into a clinically significant grade. This is fundamental to the robustness of results reported and the ability to draw comparisons between different studies with confidence (Estcourt et al., 2013). Currently because of the heterogeneity in the methods used to assess, document and grade bleeding it is not always possible to compare studies with any great confidence. If bleeding is to be used as a main outcome measure for platelet transfusion trials, it is important that it is defined and documented in a consistent and standardized way. This validation exercise was a prospective multi-centred, observational study for which the main objective was to validate a Bleeding Assessment Tool (BAT) that had been developed by the international Biomedical Excellence for Safer Transfusion (BEST) collaborative. The BEST BAT is designed to describe the site, severity, duration and clinical consequences of bleeding events in a standardized manner and is intended for use in the malignant haematology patient population.
**Methods**
Study objectives were addressed through a repeat assessment of bleeding exercise. Once the participants' platelet count was $30x10^9/L$ they had their bleeding status assessed using the BAT by trained assessors. The aim was for the participant to have an assessment of bleeding repeated by two assessors on a maximum of three consecutive days, which would generate six individual assessments. Repeat assessments were then compared for concordance i.e. Agreement in site and severity of any bleeding observed. The two assessors were blinded to the findings of one another's assessments and a repeat assessment had to take place as soon as possible after the first to try to ensure that both assessors were observing the same 'bleeding window'. A qualitative survey was used to collect feedback from bleeding assessors as to how easy they found using the tool. Feedback was used to refine the design of the BAT.

#### Results
Forty patients consented to participate. Thirteen trained bleeding assessors collected the data. Bleeding assessments were carried out on 113 separate days, 225 bleeding assessments were compared for concordance. The study found good concordance (79%) overall in observations of bleeding. The study also highlighted key areas of focus such as training and the importance of well laid out documentation to facilitate a standardized approach to the assessment of bleeding.
### Conclusion
The ultimate aim of this study is for the BEST collaborative to use their international influence to promote use of the validated and refined BAT by researchers and clinicians working in the field of transfusion medicine and clinical haematology. The standardized use of a BAT in studies using bleeding data as a main outcome measure will make it possible to draw reliable comparisons and to pool data from different studies. Ultimately this has the potential to answer research questions and to improve care for patients at a faster rate.

### P164
## Concurrent versus sequential addition of diethylstilbestrol with dexamethasone in the treatment of castration-resistant prostate cancer: an exploratory analysis
Shah-Jalal Sarker[1], Jo Whelan[1], Stuart Mealing[2], Jonathan Shamash[3]
[1]Queen Mary University of London; [2]Oxford Outcomes; [3]St Bartholomew's Hospital
**Correspondence:** Shah-Jalal Sarker
*Trials* 2017, **18(Suppl 1):**P164

#### Background
Diethylstilbestrol (DES) and dexamethasone (Dex) can be used alone or in combination to treat castration-resistant prostate cancer. This paper applies up-to-date reporting criteria and alternative statistical analyses to the data from a randomised trial carried out to determine optimal treatment sequencing (Dex plus deferred or immediate DES).
### Methods
PSA data were reanalysed and reported according to the Prostate Cancer Working Group 2 criteria, using waterfall plots. The ability of PSA changes from baseline at 4, 8 and 12 weeks to predict survival outcome was assessed. Individual patient level survival and health related quality of life (HRQOL) data were analysed using a flexible parametric model and a mixed effects model for repeated measures, respectively.
### Results
PSA changes from baseline at 4, 8 or 12 weeks did not predict overall survival ($P = 0.966$, 0.589, 0.415, respectively). Maximum PSA decline was associated with prolonged survival ($P < 0.001$), but the effect was clinically insignificant. The flexible parametric model showed that the hazard function was not completely proportional throughout the trial. Fitting a parametric function that better reflected the underlying hazard function resulted in a wider difference in median survival (3.9 months) between the two arms than seen in the original analysis, but this was not statistically significant ($P = 0.14$). Immediate DES was associated with a greater improvement in 'global health status' HRQOL score (difference vs. Deferred DES = 7.85, $P = 0.009$).
### Conclusion
The data were sensitive to the statistical approaches used. Interesting additional information was obtained. In particular, deferring DES may slightly reduce rather than improve patients' HRQOL overall. All results are exploratory only.

### P165
## The minimum clinically important difference (MCID) of the patient oriented eczema measure (POEM): do different methods of calculation give different MCIDs?
Laura Howells, Sonia Ratib, Joanne Chalmers, Lucy E. Bradshaw, Kim S. Thomas
University of Nottingham
**Correspondence:** Laura Howells
*Trials* 2017, **18(Suppl 1):**P165

#### Background
Knowing the Minimum Clinically Important Difference (MCID) enhances the interpretability of a patient-reported outcome measure and is necessary for sample size calculations in clinical trials. The Patient Oriented Eczema Measure (POEM) is a measure of eczema symptoms completed by patients or carers with a score of between 0 (no eczema) and 28 (very severe eczema). Previous studies have shown that the MCID for POEM is around 3 points.
MCID calculation methods are often described as two broad categories: anchor-based methods and distribution-based methods. Anchor-based methods ascertain how change in the measurement instrument corresponds to another measure of change using an external criterion. Distribution-based approaches are based on the distributional characteristics of the sample. Different methods measure a different type of change, therefore it is recommended a variety of methods are used.
Little research to date has explored the impact of applying different methodologies and anchors when calculating the MCID of the POEM, and there is currently no consensus on the best anchor measure to use to calculate the MCID for patient-reported outcomes. There has also been no exploration into whether the use of different data time points to calculate the MCID for the POEM may affect the results.
### Objectives
36 To assess if the MCID for the POEM is convergent for different anchor-based methods and distribution-based methods 2) To assess whether using a patient or investigator assessment as an anchor measure produces a different MCID for the POEM. 3) To compare the MCID for the POEM calculated using different time points.
### Methods
Secondary analysis utilising an existing trial data set (CLOTHES Trial ISRCTN77261365). A range of methods to determine the MCID will be compared. Anchor-based methods (including: within-patient score change, between-patient score change, the sensitivity and specificity method and the predictive modelling approach) will be assessed. These anchor-based methods will be used with both a 6 point Likert scale patient/parent global assessment (How is your/your child's eczema today?) And a 6 point Likert scale investigator global assessment (How is the child's eczema today?) As the anchor measure. Distribution-based methods (including: effect size estimate and half standard deviation of the baseline distribution of POEM scores) will also be used. Where appropriate, there will be separate calculations for the MCID in improvement and worsening of the eczema. All analyses will be repeated looking at the MCID for POEM scores at 2 months and 6 months of follow-up. Results will be available to present at the conference.
### Implications
This work will add to the knowledge around the MCID of the POEM, which can help inform sample size calculations in future clinical trials as well as enhance the interpretability of trial data and clinical practice records. The findings of this study will also be informative for calculating the MCID of other patient-reported outcomes as it will further understanding of how the methodology used can affect MCID calculations. The results will determine to what degree the methods used, the anchor measure used and the time point selected affect the calculated MCID.

### P166
## Assessing the impact of independent adjudication of serious adverse events on the safety results of the efficacy of nitric oxide in stroke (ENOS) trial
Peter Godolphin[1], Lisa Woodhouse[1], Daniel Bereczki[2], Alan A. Montgomery[1], Philip M. Bath[1], Nikola Sprigg[1]
[1]University of Nottingham; [2]Semmelweis University
**Correspondence:** Peter Godolphin
*Trials* 2017, **18(Suppl 1):**P166

#### Background
Independent adjudication of serious adverse events is common in clinical trials, especially in open label studies where outcome assessment can be prone to bias. Few studies have investigated the impact that adjudication of serious adverse events has on the results of a trial. The objective of this study was to explore the effect of independent adjudication of serious adverse events on the safety results of the Efficacy of Nitric Oxide in Stroke (ENOS) Trial.

**Methods**

ENOS was an international multicentre trial which randomly assigned patients with acute stroke and raised blood pressure to receive either transdermal glyceryl trinitrate (GTN) or no GTN. Non-serious adverse events were not recorded due to their high incidence in stroke patients and the established nature of the trial interventions. Serious adverse events (SAEs) were reported by local investigators who were not blinded to treatment allocation using a web-based SAE form. The local investigators report included event classification, event diagnosis and evidence used to determine diagnosis, expectedness of event, and likely causality. Independent adjudicators, blinded to treatment allocation, reviewed the investigators reports and used evidence available to confirm or alter the classification of event, with the adjudicator's decision being treated as the gold standard and used in the trial analysis. As well as event classification, adjudicators independently assessed causality, diagnosis and expectedness of event. A list of known adverse reactions was defined in the protocol and provided to investigators and adjudicators.

We used unweighted and weighted kappa respectively to estimate agreement between local investigators and independent adjudicators on diagnosis and relatedness to treatment of SAEs. The safety analysis of ENOS (chi-squared tests between treatment arms for SAE diagnosis) was replicated using investigator reported events, and these were compared to adjudicator reported events with a test of homogeneity. Preliminary results are provided in this abstract, with full results available for presentation in May.

**Results**

Preliminary results show that of 4011 patients enrolled in ENOS, there were 1473 SAEs reported by local investigators, reduced to 1444 after review by adjudicators (unweighted kappa, k = 0.85). There was fair agreement between investigators and adjudicators on relatedness of event to treatment with 808 agreements and 644 disagreements (weighted kappa, k = 0.30). However, when the relatedness to treatment categories were dichotomised into Definitely not or Unlikely versus Possibly, Probably or Definitely, then there were 1305 agreements and 147 disagreements (90% crude agreement, kappa = 0.32). Repeating the trial safety analysis with investigator reported events indicated that adjudication made little impact to the majority of the results, with a similar number classified by both investigators and adjudicators.

**Conclusions**

Serious adverse events were largely classified correctly by local investigators with the largest disagreements arising between relatedness of event to treatment. In a large trial, with many serious adverse events reported, independent adjudication of these events had little impact on trial conclusions.

### P167

#### Outcome selection in clinical trials – Looking back at the problems and moving forward with solutions

Paula Williamson[1], Jamie K. Kirkham[1], Carrol Gamble[1], Kerry Dwan[2]
[1]University of Liverpool; [2]Cochrane Collaboration
**Correspondence:** Paula Williamson
*Trials* 2017, **18(Suppl 1):**P167

Selection of outcomes to measure in trials designed to compare different interventions is crucial. It has been estimated that less than half of all outcome data collected in trials is fully reported, with missing data due to unpublished trials, poor reporting, and choosing not to include particular results within trial reports.

Difficulties caused by heterogeneity in outcome measurement across studies are well known. Empirical research provides strong evidence that outcome reporting bias (ORB), defined as the results-based selection for publication of a subset of the recorded outcome variables, is a significant problem in a quarter to a third of randomised trials and can have major impact in a fifth of systematic reviews. In interviews, trialists seemed unaware of the implications for the evidence base of not reporting all outcomes and protocol changes.

Systematic reviewers facing these challenges should contact trialists to try to obtain the missing data. They may subsequently apply a statistical approach as part of a sensitivity analysis. Bias bound estimation, multivariate meta-analysis, and modelling the selection process have been proposed.

Trial registration and improved reporting should help to reduce ORB, but for findings to influence policy and practice, outcomes chosen for measurement need to be relevant to patients, public, healthcare professionals and others making decisions about health care. So much could be gained if an agreed core outcome set (COS) of appropriate and important outcomes was measured and reported in all clinical trials of effectiveness in a specific condition. The COMET (Core Outcome Measures in Effectiveness Trials) Initiative, http://www.comet-initiative.org/, an innovative global project, brings together people interested in COS development and application.

This talk will review progress made with both statistical and non-statistical solutions to this problem.

### P168

#### Patient reported outcome measure development in epilepsy and intellectual disability: a systematic review of measures of anti-epileptic drug side effects

Rachel McNamara[1], Lauren Copeland[2], Andrea Meek[1], Mike Kerr[3], Michael Robling[1], Kerenza Hood[4]
[1]South East Wales Trials Unit (SEWTU), Centre for Trials Research; [2]Division of Population Medicine, Cardiff University; [3]Institute of Psychological Medicine and Clinical Neuroscience; [4]Centre for Trials Research
**Correspondence:** Rachel McNamara
*Trials* 2017, **18(Suppl 1):**P168

**Background**

Prevalence of epilepsy in adults with an intellectual disability (ID) is up to 20 times greater than in the general population. There is however little research assessing side effects of anti-epileptic drugs (AEDs) in adults with ID and epilepsy. Screening tools are available to assess AED side effects in the general adult population, and research suggests that active monitoring is sufficient to change management and improve quality of life (QOL). It is not known however whether such tools can be used to identify side effects in adults with ID, or whether included items are important and relevant to patients and carers. Furthermore, available instruments tend to focus on the more theoretical concept of QOL rather than on side effects of medication per se, and their validity or suitability for use in ID populations has not been established. A Cochrane review concluded that measurement of side effects in this population was hampered by reliability of available measures. The aim of this systematic review is therefore to identify research on measurement and impact of AED side effects in the adult epilepsy population. The review seeks to identify measures developed for adults with ID where available, and also in the general epilepsy population (i.e. Without ID), in order to identify measures that could be adapted for an ID population. The review is the first stage of a larger study to develop Patient Reported Outcome Measures (proms) to assess AED side effects in adults with epilepsy and ID. The aim of the larger study is to develop appropriate versions for patient and carers.

**Methods**

A systematic literature search was conducted in MEDLINE In-Process, MEDLINE, EMBASE, SCOPUS and Web of Knowledge. The review identified studies in adults with epilepsy (and a subset of adults with ID) taking an AED, which included a scale/outcome measure of a potential AED side effect. Studies focused on seizures as a side effect of medical treatment (brain surgery or medication) and seizure disorders not specified as epilepsy were excluded.

**Results**

460 papers were identified and 93 met inclusion criteria. Of 107 measures identified, six were appropriate for use with adult ID populations. Seven studies investigated adults with epilepsy and ID and examined side effect domains of behaviour, functionality and quality of life. Side effects of AEDs are inconsistently and inadequately measured in ID populations and are overly reliant on carer report. The overall burden of side effects is therefore likely to be under-reported.

**Conclusions**

Screening tools are available to assess AED side effects in the general adult population, however only two outcome measures are designed for use in ID populations. The focus of these measures is broader than side effects alone and therefore may not pick up the full range of side effects of importance in this group. There is a clear lack of established and validated assessment scales for patients with ID and epilepsy, and a need to consistently measure and report patient-reported side effects of medication, both in clinical practice and in trials of new medication regimes.

### P169

#### Mixed methods feasibility work to inform data collection in a main study: designing case report forms to capture variation in surgical techniques

Charlotte Murkin[1], Jane M. Blazbey[2], Leila Rooshenas[2], Conor Jones[3], Ian R. Daniels[3], Neil J. Smart[4], Natalie S. Blencowe[2], on behalf of the CIPHER study group
[1]University of Bristol; [2]University of Bristol, Centre for Surgical Research, School of Social and Community Medicine; [3]Exeter Surgical Health Sciences Research Unit (HESRU), Royal Devon & Exeter Hospital; [4]Peninsula College of Medicine and Dentistry, University of Exeter
**Correspondence:** Charlotte Murkin
*Trials* 2017, **18(Suppl 1):**P169

**Background**

Surgical procedures are considered to be complex healthcare interventions. As such, multiple variations in the delivery of a procedure with the same label are possible. Variations can be small or large, and known or unknown, because there is often a difference between what surgeons say they do and what is done. This project developed and tested methods for identifying all possible variations in a particular surgical procedure to inform the design of case report forms for use in a main study.

**Methods**

To identify variations in the surgical procedure, two phases of work were undertaken: i) systematic literature review (to identify 'known' variations in technique) and ii) qualitative work (to identify 'unknown' variations and explore clinical professionals' views on how these variations may influence outcomes of interest). The qualitative work comprised of case studies using digital video data capture and non-participant observation, and interviews with healthcare professionals. To date, 6 case studies have been conducted, as well as 13 interviews. Observation field notes and textual descriptions of the video-captured operations were coded and categorised into themes and subthemes. Verbatim transcripts of the interviews and observations were analysed through constant comparison approaches. Interview and observation themes were compared and contrasted to inform new lines of enquiry for exploration in further case studies and interviews. A long-list of themes and subthemes was synthesised from the data collected in both phases of work. The final phase of the project (yet to be undertaken) will refine and rationalise these themes through consensus methods, to finalise the data items to be included in the case report forms.

**Results**

The literature review identified 138 themes relating to technical variations in the surgical procedure of interest, and 50 non-technical factors including patient characteristics (e.g. Obesity), contextual operation factors (e.g. Emergency surgery), and the grade of the operating surgeon. The case studies and interviews identified 150 themes relating to technical variations and 64 non-technical factors. The themes identified in both phases were combined, duplicates and overlaps excluded, leaving 180 technical and 85 non-technical factors. Of these, 137 were common to both phases of work and 77 were identified during the case studies. These factors will be discussed at a consensus meeting, during which senior clinicians will agree on the data to collect during the main study.

**Conclusion**

This novel methodology incorporates multi-modal data collection to provide insights into the 'black box' of complex interventions such as surgery. It can be successfully used to identify and summarise technical variations in the delivery of complex interventions, and non-technical factors that may influence this delivery. Both phases of this study identified themes that would have otherwise remained 'unknown' if performed in isolation. Resulting data can subsequently be used to comprehensively and systematically design case report forms. This study design therefore adds value by identifying and documenting all key data and variations of an intervention.

### P170

#### Maximising the relevance of randomised trials to primary care

Gordon Forbes[1], Kirsty Loudon[2], Megan Clinch[1], Stephanie J. C. Taylor[1], Shaun Treweek[3], Sandra Eldridge[1]
[1]Queen Mary University of London; [2]University of Stirling; [3]University of Aberdeen
**Correspondence:** Gordon Forbes
*Trials* 2017, **18(Suppl 1):**P170

**Background**

Randomised trials are difficult and costly. Like most things that are hard, the effort expended is only worth it because we hope to make a difference. Sadly, the benefit to potential users such as patients, healthcare professionals and policy makers is often smaller than it should be because trial design decisions reduce the relevance of the trial results to their intended users and the contexts in which they work.

To ensure trial results match the needs of potential users it is recommended that researchers design pragmatic trials, testing their intervention under conditions similar to those found in the real world. PRECIS-2 is a trial design tool which encourages trialists to consider how pragmatic their trial is across nine different domains covering the population in the trial, how the intervention is delivered and the outcomes measured. Whilst in principle, a pragmatic trial should aim to emulate routine practice across the PRECIS-2 domains, in practice compromises will have to be made when designing trials. Little research exists to guide these decisions.

This research explores the views of people who influence practice in primary care towards assessing and using evidence from clinical trials. The aims of the study are to assess what is important in designing randomised trials, and to ascertain how the PRECIS-2 tool can be used to make research more relevant to primary care.

**Methods**

We carried out semi-structured interviews with individuals or small groups of people involved in implementing research in primary care in the UK. We interviewed people involved with journals, guideline development, research charities, research funders, primary care educators, clinical commissioning groups, GPs and clinical effectiveness research. A thematic analysis of the data from the interviews was carried out using the framework approach.

**Results**

We conducted 12 interviews across the target groups. We identified four themes in the data, how evidence is used, aspects of trials which are considered important when assessing evidence, views on trial design across the nine domains of PRECIS-2, and attitudes towards pragmatic trials. Most interviewees were aware of pragmatic trials however different views existed as to what the term meant. For some aspects of a trial, such as the flexibility given to those delivering the intervention, or the level of resources and expertise made available to deliver the intervention, emulating routine practice may not be the best way to make results relevant to primary care. Across other aspects of a trials design, for example the population and trial setting, our work indicates a pragmatic approach is more appropriate.

### Conclusions

Funders and those designing trials in should carefully consider design decisions across the PRECIS-2 domains to maximize the relevance of research to primary care. Across most aspects of their designs trials should aim to be pragmatic however there are some important exceptions where design decisions are more complex. Differing perceptions about what it means for a trial to be pragmatic could be helped by the use of the PRECIS-2 tool by those using evidence from clinical trials to influence practice in primary care.

### P171

#### Systematic review of the use of mediation analysis in randomised controlled trials in the primary healthcare setting

Gordon Forbes[1], Ceire Costelloe[2], Patty Chondros[3]
[1]Queen Mary University of London; [2]Imperial College London; [3]University of Melbourne
**Correspondence:** Gordon Forbes
*Trials* 2017, **18(Suppl 1):**P171

##### Background

Mediation analysis is a way to investigate the mechanisms by which an intervention affects an outcome. It has been proposed as a way to 'open the black box' of traditional epidemiology, shedding light on the causal pathways between interventions and outcomes. Methodological work, based on the causal inference framework, has helped formalise the assumptions required for mediation analysis to give valid causal conclusions. For most common approaches to mediation analysis of randomised trials, a key assumption is that there is no confounding of the mediator outcome association that is not controlled for in the analysis. The UK Medical Research Council recommend mediation analysis be used as part of a process evaluation of complex interventions. Trials which are conducted in the primary healthcare setting are often of complex interventions, so mediation analysis could be an appropriate method to include in any analysis plan of clinical trials in this setting. The aim of this systematic review is to examine applications of mediation analysis in clinical trials taking place in primary health care settings and use the findings to provide guidance for future analysis.

##### Methods

We searched the Medline, Embase and Cochrane Library databases using keywords that identify randomised trials, mediation analysis and primary healthcare settings. Our eligibility criteria were papers published between 2004 and 2014 which described a mediation analysis of data from a randomised trial conducted in a primary healthcare setting. Abstracts were screened for eligibility, where eligibility was unclear from the abstract we performed full text screening. Data extraction related to the study design features and type of mediation analysis and assumptions was carried out independently by two authors. Any disagreement was resolved with a third reviewer.

##### Results

A total of 138 references were identified using the electronic search strategy and 23 studies were found to be eligible for the review. Most studies were published post 2010 (70%), were set in the USA (65%) and involved an intervention targeting a mental health condition. 48% of analyses involved a single mediator measured at one time point and the others included multiple mediators or measurements over time. Ten (44%) studies reported adjusting for any covariates and only 2 studies discussed unmeasured confounding as a potential limitation of their results.

##### Conclusions

Mediation analysis is used in research conducted in primary healthcare settings to understand the mechanisms of how an intervention works. Most mediation analyses identified in this review failed to adequately control for confounding of the mediator outcome association. Even when potential confounding factors are included in the analysis the consequences of unmeasured confounding are rarely acknowledged as limitations. This could lead to invalid conclusions being drawn from many mediation analysis of randomised trials in primary health care settings. When planning a mediation analysis investigators should collect data on potential mediator outcome confounders and adjust for these variables in the analysis.

### P172

#### Motives, meanings and misconceptions: exploring the understandings and experiences of advanced lung cancer patients participating in a non-placebo clinical IMP trial

Emily Harrop[1], Simon Noble[2], Michelle Edwards[1], Stephanie Sivell[2], Barbara Moore[4], Annmarie Nelson[2]
[1]Cardiff University School of Medicine; [2]Marie Curie Research Centre, Cardiff University; [3]DECIPHER, Cardiff University; [4]Health and Care Research Wales Support Centre
*Trials* 2017, **18(Suppl 1):**P172

This abstract is not included here as it has already been published.

### P173

#### Challenges of conducting clinical trials in community pharmacies and their methodological implications: pilot trial of a training intervention for smoking treatment optimisation in pharmacies (STOP)

Vichithranie Madurasinghe, Sandra Eldridge, Ratna Sohanpal, Wai James, Liz Steed, Stephanie Taylor, Chris Griffiths, Robert Walton
Queen Mary University of London, UK
**Correspondence:** Vichithranie Madurasinghe
*Trials* 2017, **18(Suppl 1):**P173

##### Background

Expanding the role of community pharmacists is a cornerstone of UK government policy and health promotion activities such as smoking cessation are fundamental to this expanded role. We developed a service optimisation and training intervention (STOP intervention), and a cluster randomised trial was planned to evaluate the effectiveness of this intervention. In 2015, a pilot trial was carried out to assess its feasibility.

##### Methods

Twelve community pharmacies were assigned at random to STOP intervention or usual practice using simple randomisation (allocation ratio 2:1).

##### Results

STOP training intervention has potential to increase the number of smokers retained in smoking cessation services (89.5% in intervention arm vs 75.0% in usual practice arm) and subsequently quit smoking (52.6% in intervention vs 21.9% in usual practice arm). It had limited impact on pre-specified primary outcome, throughput (on average 43 vs 80 service users per pharmacy joined the smoking cessation service in intervention and usual practice pharmacies, respectively).

More importantly, the pilot was useful in highlighting the challenges of conducting clinical trials in this important but under-researched healthcare setting and how best to overcome them. The key issues we identified include: complex organisational structures between and within community pharmacies; pharmacies as businesses vs healthcare providers; achieving balance for individual characteristics in cluster randomisation; selecting trial outcomes; identifying factors influencing outcomes; data collection -using case report forms vs routine data.

##### Conclusions

The pilot was useful in establishing the potential impact of the intervention on smoking cessation outcomes, and testing the processes and procedures in place for definitive trial. We will discuss the challenges we encountered and their methodological implications.

### P174

#### I struggle with equipoise: a qualitative study exploring clinicians - Views of a randomised controlled trial (ACL SNNAP) prior to trial conduct

Loretta Davies[1], Francine Toye[2], Jonathan Cook[1], Andrew Price[1], David Beard[1]
[1]University of Oxford; [2]Oxford University Hospitals NHS Foundation Trust
**Correspondence:** Loretta Davies
*Trials* 2017, **18(Suppl 1):**P174

**Background**
Strong treatment preferences of both clinicians and patients have been shown to impact on recruitment, adherence to treatment allocation and ultimately the success of a randomised controlled trial (RCT). This is particularly evident when the interventions being evaluated are markedly different, such as the comparison of surgery with a non-operative approach such as physiotherapy. The aim of this study was to explore clinicians' views at the pre-trial stage of NIHR HTA funded RCT of anterior cruciate ligament (ACL) deficiency management comparing surgery and rehabilitation (ACL SNNAP Surgical Necessity in Non-Acute Patients) and to identify issues that may influence trial feasibility.
**Methods**
Semi-structured qualitative interviews were undertaken with a purposive sample of surgeons (n = 6) and physiotherapists (n = 6) from 6 NHS hospitals. All clinicians were experienced in the management of ACL injuries and had expressed an interest in participating in the ACL SNNAP trial. Interviews were analysed using an Interpretative Phenomenological Analysis (IPA) approach.
**Results**
Several issues were identified which may impact on the feasibility of this trial. Despite indicating a willingness to randomise, clinicians expressed varying levels of uncertainty and preferences which may impact on their ability to deliver balanced descriptions of the treatment options. This was especially evident in relation to certain patient subgroups: young and highly active patients with the potential to affect which patients clinicians approach to participate. Clinicians indicated that patients often have strong preferences for treatment of this injury, particularly towards surgery, and considered this as a potential barrier to trial recruitment. Various sources were thought to influence patients' views towards treatment, such as how information on the injury and its management are currently portrayed through the internet and media.
**Conclusion**
Exploring clinicians' views at the pre-trial stage enabled potential trial specific issues to be identified. As a result of these findings, appropriate training and support for recruiting teams prior to the start of recruitment is being developed. Trialists may wish to consider the use of pre-trial qualitative studies, particularly in trials where the interventions being evaluated are markedly different, to enable issues specific to a particular trial to be identified and addressed.

### P175
### Recruitment monitoring and predictions: data from the tranexamic acid for intracerebral haemorrhage (tich-2) trial
Katie Flaherty, Lee Haywood, Lelia Duley, Zhe Law, Philip M. Bath, Nikola Sprigg
University of Nottingham
**Correspondence:** Katie Flaherty
*Trials* 2017, **18(Suppl 1):**P175

**Background**
Recruitment in large scale clinical trials can be hard to predict during planning and difficult to maintain throughout the study. Many trials fail to finish to time and target; hence, better methods for recruitment predictions are needed. Two phase studies with internal pilots and a stop-go decision before the main phase allow time for re-estimation of recruitment.
**Methods**
The NIHR HTA Tranexamic acid for intracerebral Haemorrhage (TICH-2) trial, is a large multi-centre international randomised controlled trial, aiming to recruit 2,000 patients over two phases. The start-up phase was planned to take 18 months, recruiting 17 patients a month to reach a target of 300; actual recruitment was higher than this and the start-up phase stopped six months early, when it reached target. The main phase originally aimed to recruit 68 patients a month, reducing down to 55 patients a month after the main phase was brought forward. Recruitment was monitored continuously throughout the study in order to predict if and when the desired sample size would be reached. Methods to predict future recruitment, using existing trial data and recruitment trends, were developed. A system was developed for the trial website which shows live recruitment predictions; changing with the numbers of new participants and active sites. Averages over different periods throughout the trial were also looked at; including cumulative averages, monthly averages and phase averages.
**Results**
The average recruitment over the 12 month start-up phase was 28 patients a month from 53 centres; the mean (SD) per centre was 0.17 (0.19) patients a month, with a range of 0.02 to 0.7. The monthly averages from the main phase ranged from 29 to 61 patients a month, from 108 centres; centre averages ranged from 0.03 to 3.04 patients a month with a mean (SD) of 0.42 (0.45). The largest 15 centres recruited at least one patient a month per site, whereas, some of the smaller centres did not even recruit one patient in five months. The average for the first 12 months of the main phase lies near the middle of the main phase averages at 42 patients a month, suggesting this could be an ideal time to re-evaluate recruitment predictions; a yearly average will also ensure any seasonal fluctuations are captured. After analysing the available data the trial team found that they were unlikely to recruit to target and would need at least another nine months of recruitment to reach the target sample size.
**Conclusion**
Predicting recruitment when planning a clinical trial is difficult, methods for monitoring and predicting future recruitment should be performed throughout the study. When designing trials, flexible recruitment phase and end dates should be considered to allow for differing recruitment rates than originally planned. Monthly centre averages showed larger variation in the main phase compared to the start-up, reflecting differences between recruiting sites. Twelve months into the main phase of a study appears a reasonable time point to re-evaluate recruitment predictions. Similar systems are used in our other large trials: HTA TARDIS and BHF RIGHT-2.

### P176
### Evaluation of recruitment procedures to the self-management of osteoarthritis and low back pain through activity and skills (SOLAS) cluster randomised controlled feasibility trial [ISRCTN 49875385]
Deirdre Hurley, Isabelle Jeffares, James Matthews, Ricardo Seguardo
University College Dublin
**Correspondence:** Deirdre Hurley
*Trials* 2017, **18(Suppl 1):**P176

**Background**
The SOLAS cluster randomised controlled feasibility trial aims to assess the (1) acceptability of the novel 6 week group-based education and exercise SOLAS complex intervention to patients and physiotherapists (PTs) compared to usual individual physiotherapy, (2) feasibility of trial procedures and sample size for a definitive trial and (3) effect on secondary outcomes. The aim of this study was to evaluate the feasibility of trial recruitment procedures.
**Methods**
It was proposed to recruit 12 to 14 clusters (PT clinics) to test feasibility across a range of settings; six clusters in each arm participating in two waves of recruitment and enrolling six participants in each cluster per wave [i.e. 144 participants, 72 per arm]. A minimum of 96 participants [48 per arm] was required for sample size calculations. The recruitment procedure agreed with PTs involved their screening waiting lists to identify potential participants for an invitation letter, and researcher led telephone and face-to-face screening. The recruitment rate [total, study arm and wave] and ratio of number screened: number enrolled and reasons for exclusion at each step was calculated after each wave.
**Results**
14 clusters were recruited (7 per trial arm), each site participated in two waves of recruitment, resulting in three study waves (W1-W3). The average cluster size in each arm was below six (Intervention: mean (SD) = 4.92 (1.31), range 2–7; Control: mean (SD) = 5.08 (2.43), range 1–9) with no significant difference between arms (df = 16.909, t = −0.209, p = 0.837). The cluster size increased from W1 (Intervention: 4.25 (1.71), 2.00-6.00; Control: 4.40 (1.95), 3.00-7.00) to W2 in both arms (Intervention: 5.17 (1.17), 4.00-7.00; Control: 5.80 (1.79), 4.00-8.00), with a further increase in W3 in the intervention arm only (Intervention: 5.50 (0.71), 5.00-6.00; Control: 5.00 (5.66), 1.00-9.00). 120 participants (83.3%; of n = 144 expected) were recruited (Intervention n = 59; Control n = 61). The recruitment rate according to target increased in subsequent waves (W1 Target: 54, Actual: 39; W2 Target: 66, Actual: 60; W3 Target: 24, Actual: 21). Overall, 1708 potential participants were identified from the waiting list: 1136 (66.5%) were excluded predominantly due to diagnosis (n = 879), age (n = 158), exclusion criteria (n = 133) and symptom duration (n = 53). Of 572 invitation letters, 375 (65.6%) participants responded [W1: 42.6%, W2: 76.3%, W3: 78.3], with 224 (59.7%) excluded by telephone screen [W1: 35%, W2: 81.3%, W3: 85.1%] mainly due to exclusion criteria (n = 69), preference for individual PT (n = 62), inability to attend SOLAS group (n = 30) or poor English (n = 30). A further 31 (20.5%) were excluded at face-to-face screening with 120 participants recruited, representing 21% of invitation letters [W2: 20.2%,W3: 21.6%, W4: 19.8%]. The invitation letter was simplified after W1 following communication with PTs, and telephone screening refined after W2.

**Conclusions**

The sample size was below target but sufficient for sample size calculations. Recruitment rate, cluster size and response to invitation letters increased across waves as procedures were improved, but the enrollment rate remained unchanged. Recruitment to trials of complex interventions outside routine practice is challenging and warrants further research with patients to address their barriers and enablers to trial participation.

### P177

#### Do multimedia resources increase the quality of information provision and rate of recruitment in trials involving children and adolescents?: protocol for the TRECA study

Jackie Martin-Kerry[1], Peter Bower[2], Bridget Young[3], Jonathan Graffy[4], Ian Watt[1], Rebecca Sheridan[1], Paul Baines[5], Catherine Stones[6], Jenny Preston[5], Steven Higgins[7]
[1]University of York; [2]University of Manchester; [3]University of Liverpool; [4]University of Cambridge; [5]Alder Hey Children's Hospital; [6]University of Leeds; [7]University of Durham
**Correspondence:** Jackie Martin-Kerry
*Trials* 2017, **18(Suppl 1):**P177

**Objectives**

The first objective of the TRECA (trials Engagement in Children and Adolescents) study is to use participatory design to develop multimedia information resources for use in healthcare trials. The study will also evaluate the potential for multimedia information resources to improve the quality of decision-making about participation in healthcare trials involving children and adolescents with long-term health conditions, and then assess the impact of these multimedia information resources on rates of trial recruitment and retention.

**Background**

Randomised controlled trials are the best method for testing health interventions whilst minimising bias. However, recruitment and subsequent retention of children and adolescents in healthcare trials can be challenging. Printed participant information sheets for potential trial participants are often lengthy and difficult to read and understand. Presenting key information about trials using multimedia may help to overcome these limitations and better support children, adolescents and their parents in deciding whether to participate in a trial.

**Methods**

The TRECA study has two phases. The first TRECA phase involves a qualitative study with children and adolescents and their parents to inform the development of multimedia information resources, followed by rounds of iterative user testing to refine the resources. The multimedia information resources will contain elements of audio, video, text and animations, including some aspects that are trial-specific and others that pertain to any trial. The second TRECA phase will embed the use of the multimedia information resources into six host trials in the United Kingdom. Patients and their parents approached to participate in the host trials will be randomly allocated to one of three arms: to use the standard printed participant information sheet; or the multimedia information resource; or both the standard printed participant information sheets plus the multimedia information resource. The primary outcome will be the effect of the multimedia information resources on rate of recruitment into the host trials. Other outcomes measured include the effect of multimedia information resources on retention of participants in the host trials and the impact on the quality of decision making about participation of the patient (child or adolescent) and the parents, when compared to standard printed participant information sheets alone. A prospective meta-analysis of the outcomes from the six host clinical trials will be undertaken.

**Conclusion**

This study will inform whether multimedia information resources, when developed using participatory design principles, are able to increase rates of recruitment and retention of children and adolescents in healthcare trials. There is also the potential for patients to make better informed decisions about participation in trials (whether or not they decide to take part) through the use of multimedia information resources. The multimedia information resources also have the potential to assist with providing information on other healthcare decisions outside of clinical trials.

### P178

#### Children and young people's views on research without prior consent in life threatening situations: a qualitative study

Louise Roper[1], Frances Sherratt[2], Paul McNamara[3], Bridget Young[2], Richard Appleton[4], Esther Crawley[5], Angus Dawson[6], Lucy Frith[7], Carrol Gamble[8], Kerry Woolfall[2]
[1]University of Liverpool; [2]Department of Psychological Sciences, University of Liverpool; [3]Department of Child Health, University of Liverpool; [4]Neurology Department, Alder Hey Children's Hospital; [5]School of Social and Community Medicine, University of Bristol; [6]School of Public Health, University of Sydney; [7]Department of Health Research, University of Liverpool; [8]Department of Biostatics, University of Liverpool
**Correspondence:** Louise Roper
*Trials* 2017, **18(Suppl 1):**P178

This abstract is not included here as it has already been published.

### P179

#### How evidence is presented to potential participants in RCT recruitment consultations

Daisy Elliott, Leila Rooshenas, Samantha Husbands, Sangeetha Paramasivan, Marcus Jepson, Caroline Wilson, Jenny Donovan
University of Bristol
**Correspondence:** Daisy Elliott
*Trials* 2017, **18(Suppl 1):**P179

**Background**

The importance of evidence based medicine is now widely recognised, and randomised controlled trials (RCTs) are the gold standard method for evaluating health technologies. Comprehensive and systematic evidence reviews are essential before embarking on RCTs, and this information should be clearly presented to potential participants to ensure that they are fully informed about the rationale for the trial and treatment options. However, little is known about how health professionals present current evidence to eligible patients in RCT consultations.

**Methods**

Five UK-based RCTs were purposefully selected to include a range of trials from different clinical contexts and with different types of recruiters. Consultations in which recruiters presented information about the RCT to eligible patients were audio-recorded (n = 117). Data relating to any presentation of uncertainty or evidence regarding optimal treatment were transcribed verbatim and analysed thematically by DE using constant comparative techniques derived from grounded theory methodology. A subset of consultations were independently coded by LR and SH to confirm reliability of coding.

**Results**

Preliminary results suggest that there was considerable variation within and across RCTs in how recruiters discussed evidence. Some recruiters did not introduce the concept of uncertainty about optimal treatment or discuss any empirical evidence about treatment options at all. These recruiters had a tendency to instead draw on anecdotes, most commonly in the form of their experiences of patients' treatment outcomes. The majority of recruiters alluded to an absence of evidence to introduce the RCT but did not elaborate further. Where recruiters provided information about previous studies, they tended to summarise the findings without referring to the quality of the research. Final results will be presented at the conference.

**Conclusions**

Clinicians and nurses play a vital role in providing detailed and accurate evidence-based information to patients to facilitate informed consent for participation in RCTs. However, this study highlights considerable variability in how recruiters present evidence. It therefore demonstrates the need for support and training to enable recruiters to present information clearly, both in regards to summarising findings and appraising the quality of this research.

### P180

#### Planned implementation of a study within a trial using a stepped wedge trial design evaluating the introduction of a patient decision aid on decisional conflict in patients considering entry into the primetime study

Indrani Bhattacharya[1], Charlotte E. Coles[2], Lisa Fox[3], Anna Kirby[4], Lesley Turner[5], Hilary Stobart[5], Lesley Fallowfield[6], Judith Bliss[3]
[1]Institute of Cancer Research; [2]Oncology Centre, Cambridge University Hospitals NHS Foundation Trust; [3]Institute of Cancer Research - Clinical Trials and Statistics Unit; [4]Royal Marsden Hospital; Independent Cancer Patients' Voice; Sussex Health Outcomes Research & Education in Cancer, University of Brighton
**Correspondence:** Indrani Bhattacharya
*Trials* 2017, **18(Suppl 1):**P180

**Background**

The study within a trial (SWAT) concept enables trialists to assess different ways of designing, conducting, analysing and evaluating studies through the conduct of research embedded within a larger trial. PRIMETIME is a prospective biomarker directed cohort study aiming to identify a subgroup of breast cancer patients who can safely avoid adjuvant breast radiotherapy following breast conserving surgery. This subgroup is deemed to be at such a low risk of local relapse that the potential benefits of radiotherapy are unlikely to outweigh the known risks. The current uncertainty regarding the absolute benefit of adjuvant radiotherapy (in this subgroup), the concept of avoiding treatment, and the offer of entry into a clinical trial can be overwhelming and challenging for patients to cope with. The uncertainty patients face regarding healthcare decisions is known as 'decisional conflict'. We would like to optimise the decision making process for patients facing this uncertainty. Decision aids are 'interventions designed to help people make specific and deliberative choices among options by providing information about the options and outcomes relevant to a person's health status'. Evidence suggests decision aids reduce decisional conflict. This SWAT is designed to investigate the effect of a decision aid on patients' decisional conflict over their uncertainty regarding the absolute benefit of radiotherapy and therefore their decisional conflict over whether or not to enter PRIMETIME.

**Proposed Method**

The PRIMETIME SWAT will utilise a stepped wedge trial design. The decision aid will be in video format. Decisional conflict will be assessed using a validated decisional conflict scale in centres prior to and following implementation of the decision aid. All centres will receive the standard patient information sheets and be randomised to receiving the decision aid video at increasing intervals from when their centre began recruiting to PRIMETIME using minimisation. The primary outcome is to assess whether the addition of a decision aid video to standard patient information giving reduces patients' decisional conflict. The secondary outcome is to assess acceptance of entry into PRIMETIME.

**Discussion**

Investigating the impact of a decision aid on decisional conflict in a SWAT allows us to answer important questions in an economic and efficient manner where we are able to conduct research within the context of a larger study. Given our hypothesis that the decision aid will reduce decisional conflict, the choice of a stepped wedge trial design ensures that by the end of the study all centres will have use of the decision aid as opposed to a parallel design which may be considered less favourably as some centres would never introduce the decision aid. If we are able to determine that the introduction of a decision aid reduces decisional conflict this would provide the evidence required to support increasing resources into the development and, ultimately, routine use of decision aids for patients facing complex treatment decisions.

### P181

#### Factors influencing recruitment in large randomised trials

Danielle Edwards, Michael Lay, Martin Landray, Louise Bowman, Jane Armitage
University of Oxford
**Correspondence:** Danielle Edwards
*Trials* 2017, **18(Suppl 1):**P181

**Background**

Recruitment into large clinical trials is difficult, with many trials not recruiting to target. This can cause financial, ethical and practical problems and undermine the quality of the research as large numbers of participants are needed to accurately assess moderate effect sizes. Previous research has identified age, ethnicity, socioeconomic status, and education as important influences on the success of trial recruitment but little has been done to quantify these effects.

**Methods**

Using data from the MRC/BHF Heart Protection Study (HPS), the influence of demographic factors was explored at each stage of the trial recruitment process (invitation, screening, pre-randomisation run-in, and randomisation). The HPS dataset was chosen as patients had been identified as potentially eligible from their hospital records and then were sent a postal invitation to attend a screening visit. This method of invitation allows large numbers of patients to be invited, and provides a true, unselected denominator for the recruitment effort.

**Results**

Throughout the recruitment process, women were significantly less likely than men to continue to the next stage: of those invited, 45% of women vs. 51% of men attended the screening visit; of those attending screening, 70% of women were eligible and consented to enter run-in compared to 78% of men; and of those entering the run-in period, 82% of women vs. 87% of men were randomised (all $p < 0.001$). This led to only 18% of all women invited agreeing to enter run-in compared to 28% of men, and 11% of the overall total of women being randomised vs. 19% of men (both $p < 0.001$). These gender differences were still significant after adjusting for age, ethnicity, deprivation index as a measure of socioeconomic status, and distance from screening site. After adjustment, women were still significantly less likely than men to attend screening (OR: 0.79, 95% CI: 0.77-0.81), to enter run-in (OR: 0.57, 0.55-0.59), and to be randomised (OR: 0.69, 0.66-0.73). The REVEAL study began recruitment 17 years after HPS. There has been a decline in the proportion of patients agreeing to take part in CTSU trials when identified from electronic records. Whilst the overall proportion of patients invited that were randomised was much smaller (3% in REVEAL vs. 16% in HPS), the observed differences between men and women, after adjustment, at screening (OR: 0.43, 0.41-0.44), at run-in (OR: 0.55, 0.51-0.59) and at randomisation (OR: 0.59, 0.53-0.66), were at least as great as in HPS highlighting that this is an ongoing issue.

**Conclusion**

Further research into demographic differences in adherence to randomised treatment and completeness of follow-up will provide a comprehensive view of the influence of gender on participation throughout these cardiovascular trials. In addition, qualitative research might provide insight as to why women are less likely to participate, and how this can be addressed to maximise the relevance of the results to both men and women.

### P182

#### What are the barriers and facilitators to patient and carer recruitment to randomised controlled trials in palliative care? A systematic review with narrative synthesis

Lesley Dunleavy, Catherine Walshe, Nancy Preston
Lancaster University
**Correspondence:** Lesley Dunleavy
*Trials* 2017, **18(Suppl 1):**P182

**Background**
Why so many RCTs fail to achieve their recruitment targets is an important area of clinical practice that is poorly understood. This is especially so in the field of palliative care as patients are often 'hard to reach'. Palliative care patients have a diverse range of conditions, they are cared for in a wide variety of clinical settings and have unpredictable and complex needs.
**Aims**
To identify and synthesise knowledge about barriers and facilitators to recruitment to palliative care RCTs to develop recommendations to increase recruitment.
**Methods**
A systematic review with narrative synthesis. Social marketing theory provided a theoretical framework for the review. Medline, Cinahl, pscyinfo and Embase databases (from Jan 1990 to early October 2016) were searched. Papers included: interventional and qualitative studies addressing recruitment, primary palliative care RCTs or reports containing narrative observations about the barriers, facilitators or strategies to increase recruitment to palliative care RCTs. Themes within the literature were developed using thematic analysis.
**Results**
3832 records were screened and data extraction was carried out on 48 papers. The key barriers: gatekeeping by professionals and family care givers, high refusal rates, the need for intensive resources and participants not meeting eligibility criteria. Key facilitators included lead clinician support and key messaging. Research staff on site, regular contact with clinicians and the use of scripts/role play were seen as important recruitment strategies. Most evidence is based on researchers own reports of experiences of recruiting to palliative care RCTs rather than independent evaluation.
**Conclusion**
More methodological research is needed to try and reduce the waste of resources associated with RCTs that fail to reach their desired recruitment targets. Embedded clinical trials of recruitment strategies are a possible way forward to help to quantify whether potential strategies suggested in the literature truly have an impact.

### P183

#### Recruitment to randomised controlled trials in patients receiving unplanned hospital care: a systematic review and in-depth analysis using the ORRCA database

Ceri Rowlands[1], L. Rooshenhas[1], Jonathan Rees[2], Katherine Fairhurst[1], Carrol Gamble[3], Jane M Blazeby[4]
[1]MRC conduct-II Hub for Trials Methodology Research, School of Social & Community Medicine, University of Bristol; [2]Division of Surgery, Head & Neck, University Hospitals Bristol NHS Foundation Trust; [3]MRC North West Hub for Trials Methodology Research, Institute of Translational Medicine, University of Liverpool; [4]MRC conduct-II Hub for Trials Methodology Research, School of Social & Community Medicine, University of Bristol and Division of Surgery, Head & Neck, University Hospitals Bristol NHS Foundation Trust
**Correspondence:** Ceri Rowlands

**Background**
Recruitment to randomised controlled trials (RCTs) in unplanned hospital care (UHC) settings is more challenging than in the elective setting because there is less time and patients are often unwell. Methods to optimise this have not been previously explored. The aim of this study is to summarise methods to optimise recruitment into trials in UHC settings to inform future research.
**Methods**
The ORRCA (Online Resource for Recruitment Research in Clinical Trials; www.orrca.org.uk) database includes studies of all designs, systematically extracted from the literature, reporting on recruitment into RCTs and non-randomised clinical studies.
Included in this review were any studies within ORRCA that reported on recruitment to RCTs in an UHC setting in patients >18 years. Abstracts, editorials, reports of mental health RCTs and studies of recruitment to non-RCTs were excluded. UHC was defined as the care received during an unpredictable admission to hospital at short notice because of clinical need. This includes pre-hospital care, intensive care (ICU) admissions and A&E attendances. Screening was performed by one author (CR) with duplicate screening of 10% of the database performed by a second author (KF).
All papers were categorised according to the recruitment study design (randomised or non-randomised) and whether an intervention to optimise recruitment was evaluated. Additional categorisation addressed whether the paper evaluated recruitment to a real clinical RCT (host RCT) or potential recruitment to a RCT that did not yet exist (a 'hypothetical RCT').
Data extracted included i) perceived barriers to recruitment which formed the rationale for the study, ii) barriers to recruitment identified as the result of the recruitment stud and iii) types of intervention evaluated.
**Interim results**
Of 3114 articles within the ORRCA database, 39 were eligible. Duplicate screening did not produce any unresolvable discrepancies. One paper used a randomised recruitment study design to evaluate an intervention, 11 evaluated an intervention through a non-randomised study and 16 recruitment studies did not evaluate an intervention. A further 11 studies report results from community surveys of proposed hypothetical RCTs.
Perceived barriers to recruitment included the clinical condition of the patient, patients impaired ability to provide valid informed consent and a narrow therapeutic time window. Further barriers to recruitment identified as the result of the recruitment study were clinician's refusal for patients to be approached, workload of the clinical team, insufficient approach of eligible participants and the use of surrogate decision makers (SDMs). Types of recruitment interventions included obtaining consent in the pre-hospital setting (n = 3), the use of alternative methods of consent (n = 3), on-site training/support/education for clinical teams (n = 3), modifying the treatment window (n = 1), the use of mobile alert technology (n = 1) and the use of a screening log/site monitoring (n = 2). Further analysis is ongoing.
**Conclusion**
Rigorous comparative methodological studies to evaluate recruitment interventions are lacking in this setting. Informed consent for trial participation was the most commonly identified recruitment barrier but specific methods to optimise this require further research.

### P184

#### Conducting trials with hard to recruit disabled populations: a systematic review of the methodological challenges reported in the literature

Peter Mulhall, Vivien Coates, Laurence Taggart, Toni McAloon
Ulster University
**Correspondence:** Peter Mulhall
*Trials* 2017, **18(Suppl 1):**P184

**Background**
Approximately 15% of the world's population have a disability. Many of these disabilities will have a profound effect of the person's social, cognitive or mental functioning, often requiring high levels of costly health and social care support throughout the person's life. As such, it is imperative that they receive treatments and services that are based upon a sound evidence base. As a case example, the evidence base for medical, health and social care interventions for those with a cognitive or developmental disability is very sparse. One of the reasons for this lack of robust evidence may be because the process of conducting RCTs with disabled or impaired populations is fraught with many methodological challenges. We need a better understanding of these methodological challenges if the evidence bases are to be developed.

**Objective**

To explore the methodological barriers which are hindering the development of the evidence base for treatments and interventions for people with cognitive or developmental disabilities, and to find possible solutions to overcoming the barriers. As a case example, the literature regarding RCTs for people with Intellectual Disabilities (ID) was used to highlight pertinent issues.

**Methods**

A systematic literature review was conducted of internationally published randomised controlled trials with people with intellectual disabilities from 2000 to 2015. From a total of 7795 search results, 34 RCTs with adults with ID were reviewed to ascertain which barriers, challenges and solutions the authors faced and reported. Quantitative data were extracted in the form of frequency of reporting and qualitative data were extracted in relation to the specific barriers faced by the authors.

**Results**

A number of themes arouse including: 1) that there was a lack of detail regarding how trialists made reasonable adjustments to enable consent to be obtained, 2) that there is a lack of validated outcome measures for people with communication or intellectual difficulties, 3) the importance of engaging with family members, carers and support staff when recruiting, and retaining and 4) that sample sizes are regularly small and studies are often underpowered.

**Conclusions**

Conducting RCTs with people with disabilities, particularly intellectual disabilities, can present unique challenges that require creative solutions. To date researchers have not maximised the sharing of their 'experience base' regarding these challenges and solutions. As a result, the conducting of RCTs and the development of robust evidence bases remains slow and the health inequalities of people with disabilities continues to grow. Implications for the dissemination of the 'evidence base' and 'experience base' are discussed.

### P185

#### Maximising participant retention and outcome data in a long term cancer trial (protect)

Athene Lane[1], Michael Davis[1], Julia Wade[1], Emma Turner[1], Eleanor Walsh[1], Peter Holding[2], Sue Bonnington[2], Chris Metcalfe[1], Richard Martin[1], David Neal[2]
[1]University of Bristol; [2]University of Oxford
**Correspondence:** Athene Lane
*Trials* 2017, **18(Suppl 1):**P185

**Background**

Participant attrition and missing data can introduce biases, yet there is limited evidence for successful retention strategies to maximise collection and analysis of clinical and patient-reported outcomes (PROMs).

**Objectives**

The impact of a multifaceted retention strategy developed in a long-term cancer trial was investigated using mixed methods research.

**Methods**

1643 men aged 50–69 years were randomised between 1999–2009 to three localised prostate cancer treatments with a median of 10 years follow-up (protect: ISCRTN 20141297). Prostate cancer mortality (primary outcome) was ascertained by an independent committee following death certificate notification. Clinical secondary outcomes were collected annually in case report forms (CRFs) by research nurses in meetings with participants (or by telephone) and from medical records. Follow-up procedures included nurse training including study meetings every six months, standard operating procedures, annual site monitoring visits, source data verification (SDV, total 161) on a representative sample of participants from each site by data managers with feedback to centres. Proms were collected annually by postal questionnaires with a reminder letter to non-responders. Three interventions to reduce attrition were assessed: firstly, nurses commenced telephoning non-responders. A study pen was later included with reminders and a shortened questionnaire was sent to non-responders by recorded delivery. Questionnaire response rates were compared for a six month period before and after these interventions. There was a study website and annual participant newsletters. 18 participants were also interviewed, including about follow-up, the transcriptions were analysed thematically.

**Results**

The primary outcome was ascertained for all participants and clinical outcome data for 99% (1639) men at a median of 10 years follow-up. Site monitoring and nurse training improved data collection. SDV identified training issues to improve data collection and CRFs, although staff time required was high.

Questionnaire response rates over six years follow-up were over 85% for all proms and did not diminish over time. The reminder letter increased the response rate from 76.4% (1045/1367) to 86.8% (1187) and telephoning non-responders increased rates to 90.5% (1105/1221). The shorter version of the questionnaire had some impact (9/84 posted, 10.7%, overall 1033/1142, 90.5%). The study pen was ineffective (1026/1142, 89.8%).

In interviews, most men found the questionnaires acceptable and understood their purpose although they were less liked than the annual nurse appointment. Some men saw questionnaires becoming less relevant over time either because they felt they were cured or they reported the same information annually, however, they continued to complete them. Participant newsletters were interesting and gave a sense of belonging to a group. The study website was infrequently accessed, partly because it was assumed to contain no additional information.

**Conclusion**

A multifaceted retention strategy led to very low rates of missing clinical outcome data and participant attrition in a long-term cancer trial. Successful retention requires multiple strategies, including ongoing staff training, regular newsletters and questionnaire reminders. These strategies are optimally included at the design stage and maintained throughout follow-up to reduce the potential for bias due to participant attrition and missing data.

### P186

#### Online resource for recruitment research in clinical trials research (ORRCA)

Anna Kearney[1], Nicola L Harman[1], Naomi Bacon[2], Anne Daykin[3], Alison J. Heawood[3], Athene Lane[3], Jane Blazeby[3], Mike Clarke[4], Shaun Treweek[5], Paula R. Williamson[1], Carrol Gamble[1], Peter Bower[1], On behalf of the ORRCA group
[1]North West Hub for Trials Methodology Research/University of Liverpool; [2]Clinical Trials Research Centre/University of Liverpool; [3]Conduct-II Hub for Trials Methodology Research/University of Bristol; [4]Centre for Public Health, Queen's University of Belfast; [5]Health Services Research Unit, University of Aberdeen)
**Correspondence:** Anna Kearney
*Trials* 2017, **18(Suppl 1):**P186

**Background**

With less than a third of UK trials and 40% of US cancer trials failing to achieve their recruitment targets, addressing recruitment challenges has become an important methodological priority. However, while this focus has led to an increase in the quantity of published research, navigating this literature to identify recruitment strategies relevant to different types of trials has remained difficult.

**Aim**

The ORRCA project aims to provide an online searchable database, categorising recruitment research according to key themes.

**Data Sources**

An unrestricted search of Medline (Ovid), Scopus, Cochrane Database of Systematic Reviews (CDSR) and Cochrane Methodology Register, Science Citation Index Expanded (SCI-EXPANDED) and Social Sciences

Citation Index (SSCI) within the ISI Web of Science and ERIC in January 2015. Database specific search strategies were developed based on previous work by Treweek et al. 2010.

**Inclusion Criteria**

Studies reporting or evaluating strategies, interventions or methods used to recruit patients to randomised control trials, early phase trials, qualitative interviews, focus groups, surveys, biobanks and cohort studies. Case reports of recruitment challenges or successes and studies exploring reasons for patient participation or refusal are also included.

**Methods**

Articles were screened by title and abstract before a full text review by researchers from the Hub for Trials Methodology Research Recruitment Working Group (HTMR RWG) in the UK and the Health Research Board for Trials Methodology Research Network (HRB-TRMN) in Ireland. Eligible articles were categorised according to pre-defined recruitment themes and the following types of evidence: randomised evaluations of recruitment strategies; application of recruitment strategies with or without evaluation; observations to inform future recruitment strategies. Additional data were abstracted to enable search functionality.

**Results**

Electronic searches identified over 40,000 articles of which 3979 required full text review. The online database (www.orrca.org.uk) launched in August 2016 and is being updated periodically. We anticipate it will contain over 2000 articles once the review process is completed towards the end of 2016. Inbuilt search functionality allows results to be filtered using categories such as recruitment theme, level of evidence, health area, research methods, age and gender.

With 71% of full text articles reviewed we have identified 87 randomised studies or systematic reviews evaluating recruitment strategies, 458 articles documenting the application of strategies and 1073 articles describing observations to inform future strategies.

Maximising patient consent was the predominant theme amongst the 87 articles evaluating recruitment strategies with 30 (34%) assessing the delivery mode of recruitment information, 15 (17%) reviewing the format or content of patient information sheets and 14 (16%) evaluating other aspects of the consent process.

Analysis of all recruitment themes shows that published literature focuses on describing recruitment barriers and facilitators, exploring trial acceptability to patients and addressing cultural considerations. Few articles explore recruiter training (n = 31) the impact of trial reporting (n = 5) or blinding (n = 6).

We will present an overview of the methods for developing the ORRCA database, a full analysis of recruitment themes following completion of the literature review and suggestions for how trial teams might use ORRCA to improve their recruitment strategies.

### P187

#### Networked for success: the establishment and maturation of a trainee research network within a UK based opthalmology study

Claire Cochran[1], Katie Banister[1], Usha Chakravarthy[2], Craig Ramsay[1], Yan Ning Neo[3], Emma Linton[4], Rachel Healy[5]
[1]University of Abedeen; [2]Queens University Belfast; [3]Hillingdon Hospital; [4]Manchester Royal Eye Hospital; [5]Gloucestershire Royal Hospital
**Correspondence:** Claire Cochran
*Trials* 2017, **18(Suppl 1):**P187

Establishment & maturation of Ophthalmology Trainee Research Networks within the UK Clinical Research Network (CRN) is currently being encouraged. Such trainee networks already exist in surgery, neurology & anaesthetics. Research studies supported by the trainee networks have consistently exceeded targets for recruitment in record time. EDNA (Early Detection of Neovascular Age Related Macular Degeneration) is a publicly funded UK wide prospective cohort diagnostic study for the early detection of neovascular age-related macular degeneration (AMD). Active within 24 sites UK wide, EDNA has struggled to recruit to target within the original timeframe. In addition to existing strategies to boost recruitment, the study management team decided to embark upon the establishment of a trainee engagement exercise in EDNA similar to that seen in other clinical specialties. During summer 2016 the EDNA Study management team asked Principle Investigators at all EDNA sites to nominate a site trainee for the opportunity to co-own EDNA locally. This trainee would typically be in the early stages of their career. In return for local co-ownership of the study, opportunities for authorship, and valuable insight into modern clinical research issues; the Co-PI's are expected to assist practically and clinically at local level to identify ways in which they can positively enhance all study activities. While taking joint responsibility for proactive recruitment to EDNA we expected all Co-PIs to promote and maintain high data completeness and quality as well as attend all key EDNA meetings. In autumn 2016 these Co-PI trainees were inducted to EDNA. This presentation will describe the process and experiences of establishing a Co-PI trainee network within a UK wide diagnostic accuracy study.

### P188

#### An evaluation of the impact of quintet RCT recruitment training on the self-confidence and self-assessed recruitment practice of recruiters to surgical trials

Nicola Mills[1], Jane M Blazeby[1], Daisy Gaunt[1], Daisy Elliott[1], Sam Husbands[1], Peter Holding[2], Bridget Young[3], Catrin Tudor Smith[3], Carrol Gamble[3], Jenny L Donovan[1]
[1]University of Bristol; [2]University of Oxford; [3]University of Liverpool
**Correspondence:** Nicola Mills
*Trials* 2017, **18(Suppl 1):**P188

**Background**

Randomised controlled trials (RCTs) are regarded as the most rigorous study design to evaluate healthcare interventions but recruitment to them can be challenging, particularly to trials involving surgery. Recruiter-related factors are often cited as key reasons for this yet few interventions have been developed to support them. The quintet Recruitment Intervention (QRI) has been embedded in RCTs to understand and address recruitment difficulties. A cross-trial synthesis of findings led to the identification of hidden emotional and intellectual challenges for recruiters. These findings have been translated into training material to improve the practice of front-line health professionals who recruit to surgical RCTs. The aim of this paper is to describe the training and evaluate its impact on recruiters.

**Methods**

Surgeons and research nurses with a range of recruiting experience were offered one of four workshops appropriate to their profession. The 1-day training focused on sharing skills and evidence-based knowledge to promote awareness and tackling of key recruitment challenges, and to enhance self-confidence in recruiting patients to RCTs. The workshops were broadly similar, comprising interactive presentations, group exercises and discussion based around recruitment difficulties and targeting the different needs of the different health professionals. Recruiters-levels of self-confidence in discussing trial recruitment with patients was assessed through 10 self-completed questions on a 0–10 rating scale before and up to three months after the workshop. Awareness of key recruitment challenges and perceived impact of training on practice were assessed through rating and Likert scales after training. Data were analysed using two-sample t-tests, and supplemented with findings from the content analysis of free text comments.

**Results**

99 participants (67 surgeons, 32 nurses) attended a workshop. There was evidence of an increase in self-confidence scores following training (range of mean scores before training 5.1 to 6.9 and after 6.9 to 8.2, with 10 being most confident; p-values all <0.05). The greatest increases in scores were in the areas in which they felt least confident in prior to training, obtaining authentic informed consent (nurses) and discussing trials with suspicious patients (surgeons). Immediately after training, participants felt well aware of the challenges of trial recruitment : Surgeons' mean awareness score 8.8 (SD 1.2), nurses' 8.4 (SD 1.3) (with 10 being very aware). At follow-up, half of the surgeons reported that the training had made a lot of difference to their trial discussions with patients (19/38, 50%), with slightly fewer nurses reporting this (10/25, 40%). Only 2/36 surgeons and 2/25 nurses reported the training as making no difference. Several nurses who had received quintet RCT feedback previously felt the training was preaching to the converted, but valued the opportunity to reaffirm their practice. Attendees felt training had made them aware of their training needs and improved their recruiting skills, offering ideas for different approaches and explanations.

**Conclusion**

Quintet RCT recruitment training increased the self-confidence of surgeons and nurses in discussing RCTs with potential participants and self-assessed recruitment practice. Further research will examine whether they translate into improvements in informed consent and RCT recruitment rates.

### P189

#### Evolution of a recruitment strategy between secondary and primary care during the recruitment phase of the fourfold asthma (FAST) trial

Rebecca Haydock[1], Tim Harrison[1], Lucy Bradshaw[1], Lelia Duley[1], Andrew Skeggs[2], Eleanor Mitchell[1]
[1]University of Nottingham; [2]NIHR CRN: East Midlands
**Correspondence:** Rebecca Haydock
*Trials* 2017, **18(Suppl 1):**P189

**Background**

The Fourfold Asthma (FAST) Trial planned to recruit 2300 participants over 22 months via 5 Research Networks (CRNs) across a mix of 8 secondary care hospitals, GP Participant Information Centres (PICs) and GP Research Initiative Sites (RIS); a strategy used in a previous asthma trial which involved doubling the dose of steroids [1].

Recruitment to the FAST Trial opened in May 2013. After 6 months, recruitment was only 25% of the target due to a combination of delays with contracting and recruitment in secondary care being far lower than expected; it then became imperative to refocus our recruitment strategy on primary care.

**Method**

Recruitment trends showed that GP practices were only active for approx. 6 months after which the pool of potential participants was exhausted. It was agreed by the Trial Management Group (TMG) in February 2014 to open more RIS' to replace inactive sites. In order to do this close communication with the CRNs was essential. Mixed-methods were used to train sites, including the Trial Manager training sites face-to-face or via Skype, and, for larger CRN regions, a train-the-trainer approach was adopted in order to keep costs and time resources down. In addition, participant information was revised, the trial was promoted via various channels and the CI visited many CRN areas to publicise the trial in primary care. In January 2015 (three months before the projected end of recruitment) an 11 month extension of recruitment was agreed with the funder (NIHR HTA). In addition, following a sample size review at the request of the DMC, the target sample size was revised to between 1774 and 1850 due to a higher than expected primary outcome rate.

**Result**

This was a major project management endeavour but on 31 January 2016 the trial completed recruitment. In total 20,695 patients were invited to participate in the trial with a total of 1922 participants recruited, on target of the revised timeline and recruitment objectives, 11 months behind the original schedule. By the time recruitment closed 196 RIS had been opened across 15 regions; 54% of these were opened in just 11 months. Overall 171 RIS successfully recruited. In total 19% of participants were recruited from secondary care sites, 18% from pics and 63% from RIS.

**Conclusion**

This trial demonstrates the importance of monitoring recruitment, and rapidly investigating and responding to poor and unexpected patterns in recruitment. Through close monitoring and clear reporting we were quickly able to take action to adapt the recruitment strategy to meet targets. Throughout this process we worked closely with the NIHR HTA; this communication meant the funder was supportive of the 11 month extension in recruitment.

Although the strategy was successful it was not without its drawbacks. Many of the primary care sites had little or no previous research experience, which meant they required significant support in comparison to the secondary care sites. Lessons learnt also will be presented.

1 Harrison. T. W., J. Obourne et al. (2004). "Doubling the dose of inhaled corticosteroid to prevent asthma exacerbations: randomised controlled trial" Lancet 363 (9405): 271–275

### P190

#### Investigating recruitment imbalance in EXPONATE, a cluster randomised trial

Wei Tan[1], Oye Gureje[2], Alan Montgomery[1], Trish Hepburn[1], Bibilola Oladeji[2], Richardo Araya[3], Lola Kola[2]
[1]University of Nottingham; [2]University of Ibadan; [3]London School of Hygiene and Tropical Medicine
**Correspondence:** Wei Tan
*Trials* 2017, **18(Suppl 1):**P190

**Background**

Cluster randomised trials that recruit individual participants after allocation of clusters may have increased risk of between-arm differences in numbers and/or characteristics of recruited participants. This is particularly the case when recruiters are aware of cluster allocation and may introduce selection bias. The aim of this study was to investigate recruitment imbalance in a cluster randomised trial.

**Methods**

EXPONATE was a two-arm parallel cluster randomised trial of a primary care-based intervention for perinatal depression delivered by community midwives in Nigeria. The unit of allocation was maternal care clinic, stratified by local government area and with a recruitment ratio of 1:1. Fifteen clinics were allocated to the intervention arm, and 14 to usual care. All consecutive attendees were approached about the study, and those scoring over 12 on the Edinburgh Postnatal Depression Scale (EPDS) were invited to participate. When recruitment ceased after 16 months, a total of 686 women had been recruited, 452 and 234 in intervention and control arms respectively. We calculated recruitment fraction for each clinic and compared these between arms, before and after exclusion of outlier clinics. We compared characteristics of clinics and women by treatment arm. We then considered whether the imbalance in recruitment could have been anticipated and avoided.

**Results**

The mean (SD, range) number of women screened per cluster was 334 (331, 56–1384) and 356 (318, 25–1061) in the intervention and control arms respectively. The mean (SD, range) number of women recruited per cluster was 30 (30, 2–99) and 19 (15, 2–52), resulting in recruitment fractions of 9.4% (4.6%, 3.3%-18.8%) and 5.8% (5.6%, 1.3%-24%) in the intervention and control arms respectively. The percentage of women who screened positive on EPDS and were not subsequently recruited to the trial was small and similar between arms: 4.6% (n = 22) and 7.5% (n = 19) in intervention and control arms. A scatter plot of numbers screened versus recruited revealed two clinics both with recruitment fractions twice the mean of other clinics, and one clinic that screened more than five times the mean of other clinics. All three clinics were in the intervention arm. When these three clinics are removed from calculations, mean recruitment fraction and total number recruited in the intervention arm is 7.3% (3.0%, 3.3%-12.5%) and 205. When all 29 clinics are considered, we found no marked between-arm differences in participant characteristics (age/education/marital status/gestational age/clinical outcomes/pregnancy outcomes) at baseline.

**Conclusion**

Although nearly twice as many women were recruited in the intervention arm, there was no evidence of selection bias. It appears that the imbalance in numbers was due mainly to unusual recruitment in three clinics, all of which were in the intervention arm. More extensive feasibility work may have identified these issues. Recruitment imbalance also potentially has implications for data analysis in trials.

### P191

#### Waiting list control designs in trials of non-pharmacological interventions for eczema

Trish Hepburn, Lucy E. Bradshaw, Alan A. Montgomery, Eleanor F. Harrison, Eleanor J. Mitchell, Kim S. Thomas
University of Nottingham
**Correspondence:** Trish Hepburn
*Trials* 2017, **18(Suppl 1):**P191

**Background**

Open studies have a high risk of bias in participant reported outcomes. However, effects on recruitment, adherence and retention are less well defined. In eczema many patients are keen to trial non-pharmacological interventions, and hold high hopes that they are successful. Therefore, they may not want to be randomised to a study with a control arm. If randomised to control, they may actively seek additional therapy and/or withdraw from the study. Waiting list control designs randomise participants to intervention or control, but offer the control group the intervention at a later date. Rationale for this design include enhancing recruitment and adherence and reducing differential loss to follow-up. However, there is the potential for expectation bias as both groups are offered the intervention, and thus exaggeration of the treatment effect, especially with participant reported outcomes. There is also the risk of contamination between treatment groups when the intervention is available to independently source outside of the trial. This abstract considers recruitment, contamination, retention and treatment effects in two studies which compared non-pharmacological interventions to usual care, in children with eczema. One trial (SWET) examined the use of water softeners for 3 months. The other (CLOTHES) examined wearing silk clothing for 6 months. The interventions in both trials, can be independently sourced.

**Methods**

The number of participants recruited, contamination between the intervention and control groups and numbers lost to follow up in each group were examined for each trial. The treatment effects in each of the studies were also observed for objective and participant reported outcomes.

**Results**

Both trials recruited to target (310 in 25 months (SWET), 300 in 18 months (CLOTHES). Contamination was low - No control participants had water softeners installed during the study period (SWET). Six control participants reported wearing silk clothing during the study period (CLOTHES). Follow-up rates were high - 96% (SWET) and 94% (CLOTHES) - and similar between groups. In both trials, there was no evidence of an intervention effect in objective outcome measures (including the primary outcome). Similar differences between intervention and control groups were observed in the mean Patient Orientated Eczema Measure score - a subjective measure of symptoms (SWET-2.0 (95% CI −3.5 to −0.5); CLOTHES −2.8 (95% CI −3.9 to −1.8)).

**Discussion**

In both trials recruitment targets were met, contamination was low, and follow-up rates were high and comparable in control and intervention. It is not known if this was due to the relatively short duration of the studies, a compliant participant population or offering the intervention to the usual care group. The consistent intervention effect on subjective but not objective outcomes be the result of expectation bias due to the design. However, there may be preconceived expectations of benefit regardless of access to the intervention. Research should be performed in this patient population to determine whether the waiting list control design impacts on willingness to participate, non-adherence and withdrawal from the study. This should take into account the availability of the intervention, duration of the study, and participant's prior opinions.

### P192

#### Multiple imputation methods for estimating causal treatment effects in trials with non-compliance

Karla Diazordaz, James Carpenter
LSHTM
**Correspondence:** Karla Diazordaz
*Trials* 2017, **18(Suppl 1):**P192

**Background**

Non-compliance with the treatment assigned is a common problem in randomised controlled trials. In order to obtain unbiased estimates of the causal effect of the treatment received, sophisticated statistical methods are necessary. One popular estimand is the complier average causal effects (CACE). Approaches to estimating the CACE include Bayesian and frequentist methods for principal stratification and instrumental-variables (IV) estimators. These approaches are rarely used in practice, probably because of their perceived complexity.

**Methods**

Here, we propose using multiple imputation (MI) methods, which have been adopted by the clinical trials community to handle missing data problems, to estimate CACEs and causal average treatment effects (ATE). We propose three MI approaches. The first one imputes the potential outcomes directly (MI-Y), assuming the non-compliance is ignorable given the variables in the imputation model, to estimate ATEs. The other two approaches impute the principal compliance classes, which are assumed to be independent of randomized treatment and to have known marginal distribution. The outcome models use to estimate CACEs include an interaction between compliance class and randomized treatment, and it is the coefficient of this interaction which gives us the CACEs. There are two possible ways of handling this interaction term in the imputation step. The first is passive imputation (denoted MI-C). The second is to use rejection sampling for the proposed imputed values. This is known as "substantive model compatible" (MI-C-SMC) imputation. We modify the imputation model probabilities to obtain conditional distribution with the desired marginals. Using a full-factorial simulation, we investigate the finite sample properties of MI-Y, MI-C and MI-C-SMC methods for estimating ATEs/CACEs, in terms of coverage of the 95% confidence interval (CI) and bias. We consider settings where the outcome is (i) normally distributed or (ii) binary, and compare them to competing procedures (IV 2-stage least squares and full Bayesian modelling) in settings where the association between treatment received and outcome is confounded. We have two settings, low or high confounding, i.e. There exists a variable X which is ((i) weakly or (ii) strongly) associated with at the probability of complying and also ((i) weakly or (ii) strongly) associated with the outcome. We considered situations where the confounder X is (i) measured or (ii) unmeasured. We also motivate and illustrate the methods in practice using a real clinical trial.

**Results**

In settings where the confounder is low, and measured, all MI methods perform well, but when there is unmeasured confounding MI-Y results in low coverage rate (89%). For high confounding situations, MI-C performs well when the confounder is measured, but results in biased estimates with unmeasured confounding. MI-C-SMC seems to perform well in all settings considered, as do the Bayesian methods. MI-C and MI-C-SMC appear to be more efficient than 2sls methods in small sample settings.

**Conclusions**

Multiple imputation methods can be used to impute the unobserved compliance classes and then used these to obtain compliance adjusted causal effects of treatment. MI methods may have the advantage of dealing with missing data and non-compliance seamlessly, and increased efficiency.

### P193

#### The assumption of common secular trends across clusters in a stepped-wedge cluster randomised trial: does it matter?

Hemming[1], Alan Girling[1], Andrew Forbes[2]
[1]University of Birmingham; [2]University of Monash
**Correspondence:** Hemming
*Trials* 2017, **18(Suppl 1):**P193

The typical framework for modeling data from a stepped-wedge cluster randomised trial (SW-CRT), the Hussey and Hughes model, assumes a common (piecewise constant) secular trend across all clusters. In some situations this may be a tenable assumption. But, in other situations, for example with clusters set across very different settings (for example countries) this assumption might be implausible.
Through a simulation study we examine the influence on bias, accuracy and coverage of this assumption using real underlying temporal trends from paediatric intensive care outcomes where this assumption does not hold. We also examine the performance of alternative models, including models in which time effects are ignored, common linear time effects, cluster specific linear time effects and models including random time effects. We consider both mixed models and generalised estimating equations, and use a full factorial design to examine a range of scenarios including small and large numbers of clusters and steps; small and large inter-cluster correlations; and small and large cluster sizes.

### P195

#### Estimating intervention effect in cluster randomised controlled trials with non-compliance

Charles Opondo, Katherine Halliday, Stefan Witek-McManus, Elizabeth Allen
London School of Hygiene & Tropical Medicine
**Correspondence:** Charles Opondo
*Trials* 2017, **18(Suppl 1):**P195

##### Background

In a randomised controlled trial the effect of an intervention can be estimated by calculating the difference in outcomes between the groups. The Standard Approach uses an intention to treat analysis (ITT) where all participants are included in the group to which they were assigned, whether or not they received their allocated intervention. An ITT analysis reduces post-randomisation selection bias, and estimates the intervention effect under routine application, its effectiveness. However, an ITT analysis does not estimate efficacy, the effect of an intervention under ideal circumstances. Two approaches are commonly used to estimate efficacy when there is non-compliance (or non-adherence): per-protocol (PP) analysis, in which only individuals who received the intervention they were randomised to are included in the analysis, and an As-treated (AT) analysis in which individuals are analysed according to the intervention they received. Both approaches may lead to biased estimates of the treatment effect since randomisation is broken.
Here we explore alternative estimates of intervention effects in a cluster randomised trial in the presence of non-compliance.

##### Materials and methods

We use data from a trial of learner treatment kits (LTKs), comprising malaria rapid diagnostic test kits (RDTs) and artemisinin-based combination therapy drugs (ACTS) administered by teachers, on school attendance by Malawian children. Observations were clustered within schools.
The primary analysis was an ITT analysis, and additionally an AT and a PP analysis were carried out. We will use causal modelling to explore other possible estimators of the effect of the intervention in the presence of non-compliance. The two possible estimators we consider are: (i) the complier average causal effect (CACE) which estimates the treatment effect among compliers and involves identifying groups of individuals with respect to their group assignment and compliance, and comparing the outcome across groups in those who would have complied with the intervention they were randomised to, and (ii) the average treatment effect in the treated (ATT) which estimates the average effect of treatment on those subjects who ultimately received the treatment.

##### Results, conclusions and future research

The ITT and PP analyses provided no evidence of an effect of the intervention on school attendance. However, an AT analysis suggested that that children who actually used the LTKs were less likely to be absent from school. We will present the CACE and ATT estimates and explore the assumptions underpinning these effect estimators and their applicability in cluster randomised trials with non-compliance.

### P196

#### Multiple imputation for missing covariates in clinical trials with interactions

Soeun Kim
University of Texas Health Science Center
*Trials* 2017, **18(Suppl 1):**P196

Multiple imputation (Rubin, 1978, 1996) is a widely used method for handling incomplete data, and availability of software packages that implement multiple imputation has allowed various practical applications. In randomized clinical trials, the treatment assignment is usually completely observed but other predictors may be missing, and it is important to appropriately account for incomplete data. In this paper we consider an interaction model in a clinical trial setting with a missing covariate and the treatment variable, where outcome can be binary or continuous. With the interaction model, the multivariate normal assumption is no longer satisfied, and the usual implementation of multiple imputation under multivariate normal assumption can lead to biased results. We introduce a joint model approach for imputation of missing covariates in clinical trials for the linear or logistic regression setting, and evaluate various approximation approaches in a simulation study. We recommend specific approaches that incorporates interactions in the imputation procedure. These approaches are applied in the analysis of clinical trial data on randomized blood products for severely injured patients.

### P197

#### Blinded and unblinded sample size re-estimation procedures for stepped wedge cluster randomised trials

Michael Grayling, Adrian P. Mander, James M. S. Wason
MRC Biostatistics Unit
*Trials* 2017, **18(Suppl 1):**P197

##### Background

Like any trial, the ability to accurately estimate the required sample size of a stepped-wedge (SW) cluster randomised trial (CRT) depends upon the precise specification of several nuisance parameters. In practice, providing accurate estimates for these nuisance parameters may be difficult, and thus there is a risk that many SW- CRTs may be conducted with undesirable operating characteristics. Trials could be over-powered; leading to increased cost, or under-powered; increasing the likelihood of a false negative result. We address this issue here for cross-sectional SW- CRTs by proposing methods for blinded and unblinded sample size re-estimation (SSRE).

##### Methods

Blinded estimators for the variance parameters of a SW-CRT analysed using the Hussey and Hughes model are developed, and demonstrated to be unbiased in the absence of treatment and period effects. Following this derivation, complete procedures for blinded and unblinded SSRE after any time period in a SW-CRT are detailed. Explicitly, we address the case where a limited number of clusters for recruitment have been set, but increased recruitment within a cluster is feasible. The performance of both procedures is then examined and contrasted through a simulation study, using a recently completed SW-CRT as motivation. A simple adjustment to more accurately control the type-I error rate is also proposed.

##### Results

For our example scenario, if the two key variance parameters were under-estimated by 50%, the SSRE procedures were able to increase power over the conventional SW-CRT design by up to 26%. The performance of the SSRE procedures is demonstrated to be robust to the choice of re-estimation time point, whilst the proposed adjustment to account for the observed type-I error rate inflation is often able to control to approximately the nominal level.

#### Conclusions
The considered SSRE procedures can bring substantial gains in power when the underlying variance parameters are mis-specified. Though there are practical issues to consider, such as the requirement for data to be collected and stored efficiently for analysis, the procedures performance means researchers should consider incorporating SSRE in to future SW- CRTs when there is uncertainty over the values of the variance parameters.

### 198
## Investigating multiple imputation in cluster randomized trials
Brittney Bailey, Rebecca R. Andridge, Abigail B. Shoben
The Ohio State University
**Correspondence:** Brittney Bailey
*Trials* 2017, **18(Suppl 1):**198

Missing data in cluster randomized trials are often handled with parametric multiple imputation (MI), assuming multivariate normality and using random effects to incorporate clustering. Since data do not always satisfy this assumption, a nonparametric approach to MI is desirable. Predictive mean matching (PMM) is a nonparametric approach where missing outcomes are imputed with observed outcomes in the data from donors that are similar to the missing cases. It is not clear how best to extend PMM to multilevel data. Two possibilities are to ignore clustering in the imputation model or to include fixed effects for clusters. In parametric MI, ignoring clustering in the imputation model leads to underestimation of the MI variance, while including fixed effects for clusters tends to overestimate the variance. A mixed effects imputation model can be used as the basis for matching, but this is computationally intensive and increases reliance on distributional assumptions. To simplify computation and reduce bias in the estimated variance, we investigate a weighted PMM approach that incorporates both the fixed effects imputation model and the imputation model that ignores clustering.

### P199
## R package to implement rank preserving structural failure time models
Simon Bond[1], Annabel Allison[2]
[1]Cambridge Clinical Trials Unit; [2]Medical Research Council Biostatistics Unit
**Correspondence:** Simon Bond
*Trials* 2017, **18(Suppl 1):**P199

The rank preserving structural failure time model (RPSFTM) is a method used to adjust for treatment switching in trials with survival outcomes. Treatment switching occurs when patients switch from their randomised arm to the other treatment during the study. The RPSFTM is due to Robins and Tsiatis (1991) and has been developed by White et al. (1997, 1999).
The method is randomisation-based and uses only the randomised treatment group, observed event times and treatment history in order to estimate a causal treatment effect. The treatment effect, Psi, is estimated by balancing counter-factual event times (i.e. The time that would be observed if no treatment were received) between treatment groups. A g-estimation procedure is used to find the value of Psi such that a test statistic Z(Psi) = 0. Recensoring must be performed as censoring becomes informative on the counter-factual time scale.
An R package titled "Rpsftm" has been developed and is freely available for download on the CRAN website. This package implements the method as described above. The main features are: Building directly on the established "Survival" package to calculate the z-statistic, and the uniroot() function to solve the estimating equation; A familiar formula syntax: Surv(time, status) ~ rand(arm, rx) + covariate, to represent the censored failure time, the rand(arm,rx) representing the randomised treatment arm and observed proportion of time spent on the randomised treatment, plus any adjusting covariates; Implementation of the re-censoring method, when a theoretical censoring time is known; Auto-detection of perfect compliance in a treatment arm, with corresponding adjustment to the re-censoring; Routine output in terms of summary, and print methods; Estimates, and confidence intervals of the causal parameter; Sensitivity analysis to the model assumption of a common treatment effect, allowing the user to vary the magnitude of effect of treatment between patients; Diagnostic plot to help resolve potential numerical non-convergence issues.
We provide worked examples to illustrate the use of the package and the methodology.

### P200
## Statistical analysis strategies for PRO-CTCAE data in oncology clinical trials: a simulation study
Amylou Dueck[1], Jeff A. Sloan[1], Jared Foster[1], Jennifer Le-Rademacher[1], Gita Thanarajasingam[1], Ethan Basch[2]
[1]Mayo Clinic; [2]University of North Carolina
**Correspondence:** Amylou Dueck
*Trials* 2017, **18(Suppl 1):**P200

#### Background
Adverse events (AEs) in oncology trials have historically been reported by clinicians using National Cancer Institute's (NCI'S) Common Terminology Criteria for Adverse Events (CTCAE). Traditional statistical analysis of AE data has primarily involved summary measures (e.g., maximum grade post-baseline) even though a variety of other approaches exist including cumulative incidence estimation in the presence of competing risks. AE data reported directly by patients using NCI's Patient-Reported Outcomes version of the CTCAE (PRO-CTCAE) are similar in ordinal format to CTCAE data. PRO-CTCAE data may introduce statistical challenges due to high baseline symptom rates and non-ignorable missing data.
### Methods
Baseline and six post-baseline scores on a five-level ordinal scale (0 = none, 1 = mild, 2 = moderate, 3 = severe, 4 = very severe) were simulated for 100 patients/arm in 1,000 two-arm trials using a multivariate ordinal distribution for combinations of baseline prevalence rates (5% vs 50%) and change over time (no change vs 30% increase vs 30% decrease). Between-arm comparisons included t-tests and Wilcoxon rank-sum tests of the maximum score post-baseline (maxpb) and a novel baseline adjustment score (bladj); chi-squared tests of the rate of maxpb or bladj >0; general linear mixed models (GLMM) of longitudinal scores; and Gray's tests of the cumulative incidence of score >0 with and without adjustment for baseline. The GLMM modeled all scores allowing for random intercepts and slopes with statistical significance based on the arm-by-cycle interaction effect (unstructured covariance was used to account for within-patient correlation of scores over time). Bladj was computed for each patient as maxpb if maxpb was worse than the baseline score, or as zero if maxpb is the same or better than baseline.
### Results
When baseline prevalence was low, 30% increase vs no change yielded high frequency (>99%) of statistically significant results using all methods. When baseline prevalence was high for comparisons of 30% increase or decrease vs no change, maxpb (45-69%) yielded more statistically significant results than bladj (40-63%) regardless of statistical test, with the modeling approach (80-85%) having higher frequency than chi-squared test (49-69%), Gray's test (45-58%), Wilcoxon rank-sum test (45-66%), and t-test (40-57%). In varying the baseline prevalence (5% vs 10% vs 30% vs 50%) but maintaining the post-baseline scores linearly increasing from 55% to 80%, rate of maxpb >0 was 93% in all simulations compared to 93%, 91%, 83%, and 72% for bladj.
### Conclusions
Existing statistical methods for clinician-reported AE data and PRO data are candidate methodologies for the statistical analysis of PRO-CTCAE data. The general linear mixed model approach appears to provide the most power for between-arm comparisons among the tested approaches. The novel baseline adjustment method appears to account for some but not all pre-existing symptoms.

### P202

#### Structured statistical analysis plans for improved clarity of intended analysis

Colin Everett
University of Leeds
*Trials* 2017, **18(Suppl 1):**P202

##### Background

Prior to any formal analysis of data in a clinical trial, a Statistical Analysis Plan (SAP) must be written, reviewed and approved. This document describes how the data analysis will be performed, lists the endpoints of interests, and defines how they will be derived. Documents and descriptions are typically written in prose, meaning that clarity of the analysis intended by the statistician, and that understood by a reviewer depend on the writing style of the person who drafts the SAP. Assumptions made by a reviewer about the descriptions of the analysis or endpoint derivation may differ from that intended, but not specified. It is important to avoid instances of a derivation needing to be overruled at analysis time due to disagreement on what is meant by sentences both thought had clear and obvious meanings, or where alternative approaches are not defined upfront.

##### Results

Templates for the derivation of variables are proposed for how to make clear how in mathematical and programming terms an endpoint is to be derived, and the analysis is to be performed. Endpoint templates include worked examples and references. Analysis model templates include types of variables (binary, categorical, continuous), expected ranges for continuous variables, or meanings of values for categorical variables, the type of model to fit, whether effects are fixed or random. Procedures for checking assumptions are listed. Strategies for dealing with potential analysis pitfalls are included, including simplifying models in the case of non-convergence, non-positive variance component for random effects and violation of modelling assumptions.

### P203

#### A review of design and analysis methods for pressure ulcer research

Isabelle Smith[1], Linda Sharples[2], Jane Nixon[2]
[1]University of Leeds; [2]University of Leeds, Leeds Institute of Clinical Trials Research
**Correspondence:** Isabelle Smith
*Trials* 2017, **18(Suppl 1):**P203

##### Background

Pressure ulcers (PUs) are defined as a "localized injury to the skin and/or underlying tissue usually over a bony prominence, as a result of pressure, or pressure in combination with shear" [1]. PUS are painful and debilitating for patients, represent a significant cost to the NHS and are a key quality indicator for the Department of Health.

##### Motivation

PUs are categorised using an ordered categorical scale based on the appearance of skin. PU classification requires clinical judgement and misclassification can occur when undertaken by non-specialist staff, particularly for early skin changes. For PU research, investigators assess multiple skin sites for each patient at multiple time points, recording whether skin is healthy or not, and the PU classification where applicable. During analysis these repeated measurements are often aggregated into a single outcome measure, defined as development of at least 1 category 2 PU; therefore many observations are not directly analysed. Consequently large sample sizes are required for trials of PU prevention and intervention strategies. PU trials are further complicated by missing data due to administrative or patient factors and misclassification due to the judgement required for categorisation of skin changes.
Methods that use all observations, including repeated assessments at multiple skin sites, such as multi-state models, have the potential to address these problems. It is important to understand how trials are currently designed and analysed in this context in order to develop recommendations for optimal designs.

##### Aim and objectives

The overall objective of this research is to transform the design and analysis of PU trials by making better use of all data collected from repeated measures of skin changes. The aim of this project is to review currently used PU research designs, focussing on outcome measurements and their analysis.

##### Plan of Research

We will present a review of methods used in PU trials and observational cohort studies including how data are collected and analysed to illustrate the extent of the problem. Key manuscripts were identified through systematic reviews of published PU research. From these a pearl-growing strategy was adopted to identify other trials and large cohort studies. Finally experts in the field were approached to ensure major studies were not overlooked. Data extraction was pre-specified to include study design, frequency of assessments, assessor characteristics, PU definition, primary outcome including derivation, analysis methods including relevant assumptions and accommodation of complications such as censoring or missing data and effect size to quantify differences in study conclusions based on analysis methods used. Summaries of methods used in PU research will be presented and critiqued for quality and information provided from a statistical perspective.

##### Conclusion

Currently used methods for design and analysis of PU trials are inefficient and ignore many complexities that introduce variation into the results. More efficient designs and analysis methods may reduce the numbers of patients required and be less subject to bias. Methods may generalise to other situations in which a disease process can be represented by correlated longitudinal categorical data.

### P204

#### Frequency of data collection in a randomised controlled trial for long term eczema management in children

Lucy Bradshaw[1], Trish Hepburn[2], Alan A. Montgomery[2], Eleanor F. Harrison[2], Eleanor J. Mitchell[2], Laura Howells[3], Kim S. Thomas[3]
[1]University of Nottingham; [2]Nottingham Clinical Trials Unit, University of Nottingham; [3]Centre of Evidence Based Dermatology, University of Nottingham
**Correspondence:** Lucy Bradshaw
*Trials* 2017, **18(Suppl 1):**P204

##### Background

Diary cards and questionnaires are frequently used to collect data in clinical trials. However, data collection can be burdensome and completion may decline over time. Despite the often large volume of data, this may be reduced to summary measures for analyses.
The CLOTHES trial randomised 300 children with moderate to severe eczema to standard care plus silk clothing for 6 months or standard care alone. A nested qualitative evaluation was included: 32 parents participated in focus groups or telephone interviews. Patient-reported symptoms were assessed weekly using online or paper questionnaires for 6 months and during scheduled clinic visits at baseline, 2, 4 and 6 months using the Patient Orientated Eczema Measure (POEM). The mean of participants' weekly POEM scores was a secondary outcome measure. We explored whether the results and conclusions would have changed if only data collected at 2, 4 and 6 months were used in the analyses.

##### Methods

For the trial analysis, the mean of participants' Weekly scores was analysed using a linear model weighted according to the number of weekly questionnaires completed. This analysis was repeated using the scores from week 8, 16 and 24 questionnaires only and the scores collected in clinic at the same timepoints (2, 4 and 6 months).
Results of the nested qualitative study were reviewed to determine whether completion of the weekly questionnaires had been identified as a theme.

**Results**
The difference between the two groups using all of the questionnaire data in the participant mean of the weekly scores was −2.8 (95% CI −3.9 to −1.8) in favour of the intervention group, $n = 147$ control, n = 145 intervention). Repeating this analysis using data only from the questionnaires at weeks 8, 16 and 24 showed a difference of −2.6 (95% CI −3.9 to −1.3), $n = 134$, $n = 137$ respectively, and using clinic data at 2, 4 and 6 months was −2.3 (95% CI −3.5 to −1.1), $n = 141$, $n = 142$ respectively.
Several parents felt that questionnaire completion had been useful in prompting more regular use of usual treatments whilst others felt they were repeating themselves each week and this may not be helpful (Qualitative study).
**Conclusion**
The results were very similar for all three analyses and conclusions would not have changed if less data had been collected. Therefore, weekly data collection may not be needed when summary measures are used to compare groups. More frequent data collection may be useful in other circumstances for example if there is a need to identify sudden flares.
The process of data collection should also be considered. Frequency of data collection needs to be balanced against the potential for data collection itself to act as an intervention and influence behaviour in pragmatic trials.
Further work is needed to determine the optimum frequency of data collection to capture both the chronic relapsing nature of eczema and changes in condition due to an intervention. This is planned as part of the Harmonising Outcome Measures for Eczema long term control outcome domain.

### P206
### Beyond maximum grade: a novel longitudinal toxicity over time (TOXT) adverse event analysis for targeted therapy trials in lymphoma
Gita Thanarajasingam, Pamela J. Atherton, Levi Pederson, Paul J. Novotny, Thomas M. Habermann, Jeff A. Sloan, Axel Grothey, Shaji Kumar, Thomas E. Witzig, Amylou C. Dueck
Mayo Clinic
**Correspondence:** Gita Thanarajasingam
*Trials* 2017, **18(Suppl 1):**P206

This abstract is not included here as it has already been published.

### P207
### Prediction model for developmental outcome at 2 years of age for babies born very preterm
Karan Vadher[1], Brad Manktelow[2]
[1]Oxford Clinical Trials Research Unit; [2]University of Leicester
**Correspondence:** Karan Vadher
*Trials* 2017, **18(Suppl 1):**P207

**Background**
Children who are born preterm are known to be at increased risk of a range of developmental problems. The Preterm and After (PANDA) study aims to provide information about the long term outcome of children born very preterm (less than 31 gestational weeks) admitted for acute neonatal care in the east of England. Within PANDA, the PARCA-R questionnaire is completed by parents in order to measure cognitive and language development at 2 years of age. These data are added to obstetric and neonatal data collected by The Neonatal Survey (TNS), an ongoing study of neonatal intensive care activity in the same geographic area. It includes clinical information on the child and their neonatal care as well as the developmental outcome of the child; alive with no developmental delay (DD), alive with DD and death before 2 years of age.
**Aim**
The aim of this project was to investigate developmental outcome at 2 years of age for children born very preterm. The PARCA-R survey completed by the parent was used and failure to do so led to a missing outcome response. An investigation into the missingness and its assumptions were also investigated as almost half the dataset had a missing outcome which this abstract will concentrate on.
**Subjects**
The dataset is a subset of TNS and contained 2028 participants, which included babies born very preterm admitted to neonatal care between 2009 and 2010.
**Methods**
The three nominal outcomes were modelled using multinomial logistic regression. Missingness was investigated by complete case analysis, Inverse Probability Weighting (IPW) and multiple imputation. To allow for comparison between the three methods, the same covariates were adjusted for in the final multinomial logistic regression outcome models. Probabilities, odds ratios, log odds and standard errors were used to compare the three different approaches.
**Results**
Missing completely at random was disregarded as the IPW missingness model highlighted that the deprivation area, mother's age and mother's ethnicity had an effect on whether the PARCA-R survey was completed. For instance, mothers aged 34+ were 3.4 times more likely to respond than mothers younger than 23 years, when controlling for deprivation area and mother's ethnicity. The imputation model also produced strong evidence of covariates predicting non-responders. Once the investigation of missingness had been conducted the same multinomial logistic regression was produced. The optimal model predicting developmental outcome contained gestational age, sex of the baby and CRIB II score as well as a quadratic term for gestational age. Unsurprisingly, complete case analysis yielded very different results to the models that used IPW and multiple imputation. Odds ratios and probabilities of each outcome were broadly similar with multiple imputation yielding smaller standard errors of the odds ratios in the multinomial logistic regression.
**Conclusions**
IPW and multiple imputation both vary methodologically and there are a number of limitations with both methods, however, it is proven to produce very similar results and can be effective to use the data available to predict the missing outcome. It is concluded multiple imputation is more flexible than IPW when modelling missing outcomes.

### P208
### A comparison of baseline as response and missing indicator methods for missing baseline data in a mixed design cluster randomised control trial
Lesley-Anne Carter, Chris Roberts
[1]Centre for Biostatistics, School of Health Sciences, University of Manchester, UK
Correspondence: Lesley-Anne Carter
*Trials* 2017, **18(Suppl 1):**P208

To investigate treatment efficacy in randomised control trials, repeated observations are taken on a cohort of participants and the change in response following treatment is assessed. The commitment required of participants to stay involved in the study, however, makes this design open to both recruitment issues and attrition. A cross-sectional design may be used in conjunction with the cohort design to protect against these problems, recruiting additional participants who only contribute once to the study, resulting in a 'mixed' design.
The EQUIP cluster randomised control trial was designed to evaluate the efficacy of a training intervention for community mental health teams (CMHT), employing such a mixed design. The 'cluster cohort' sample provided baseline data on service users prior to randomisation and follow up data at six months following baseline assessment, via face-to-face interviews. The 'cluster cross-sectional' sample involved all service users under the care of the cmhts not in the cohort sample to be sent a postal questionnaire six months after randomisation. Comparison of the results of the two designs would allow external validity of the intervention to be investigated. The combined sample was intended to increase power to detect the intervention effect should retention rates not meet expectations. As data were only collected in the cross-sectional design at six months post randomisation, baseline data were missing in this sample, posing a problem for the combined analysis. Two methods for overcoming this issue were considered: using baseline as response, where a joint model of baseline and response is fitted with all observed data, and the missing indicator method in which an indicator variable for the missing data is include in the model as a covariate.
These two methods will be presented with a discussion of the challenges encountered in the application of each to the cluster randomisation trial design of the EQUIP study.

### P209

#### Bayesian methods for informative missingness in longitudinal intensive care data

Shalini Santhakumaran[1], Alexina J. Mason[2], Anthony C. Gordon[1], Deborah Ashby[1]
[1]Imperial College London; [2]London School of Hygiene and Tropical Medicine
**Correspondence:** Shalini Santhakumaran
*Trials* 2017, **18(Suppl 1):**P209

Scoring systems based on multiple components are often used in intensive care trials to characterise disease severity. Missing data in the overall score can be substantial due to the number of contributing components, and the problem is exacerbated if data are collected at multiple time points. A complete case analysis is prone to selection bias, and for component scores is highly inefficient. It is preferable to include individuals with incomplete data in the analysis by imputing their missing values. The imputation process should be based on plausible assumptions about the causes of the missing data and reflect the longitudinal trajectory for each patient. We demonstrate how this is facilitated by adopting a Bayesian framework, using data from the Levosimendan for the Prevention of Acute Organ Dysfunction in Sepsis (LEOPARDs) trial.
In the LEOPARDs trial, the primary outcome was the mean daily total Sequential Organ Failure Assessment (SOFA) score while in ICU. The total SOFA score is the sum of five components and some of these components are determined by multiple variables. Although 6% of scores were missing across components, this led to 17% of the total SOFA scores having a missing component. There was a clinical expectation that measurements may not be taken if there was no change, or if the scores were normal. The assumption of a lack of change is in line with the last observation carried forward (LOCF) approach. This method gives a single imputation, so does not take account of the uncertainty due to the missing data, leading to overprecise estimates. Standard multiple imputation (MI) overcomes this problem, but typically assumes that the probability of a missing score does not depend on the score itself, after conditioning on observed data. This was implausible in the LEOPARDs trial because the decision on whether to take a measurement is informed by clinical judgement about its likely value, and so the missingness is 'informative'.
We used Bayesian Markov Chain Monte Carlo (MCMC) methods to impute missing values at a component level, based on a selection model factorisation which specifies a marginal distribution for the scores (analysis model) and a conditional distribution for the missingness indicators given the scores (missingness model). An autoregressive process was incorporated into the analysis model to take account of the longitudinal structure in the scores, and informative prior distributions specified for the parameters in the missingness model to reflect various assumptions about the missingness mechanism. We applied a bootstrap approach to calculate the difference between treatment groups because of the non-normal distribution of the daily total SOFA scores, with a separate bootstrap sample taken at each MCMC iteration.
Results from the Bayesian analysis showed more uncertainty than those obtained using LOCF, whilst allowing for informative missingness unlike standard MI approaches. In addition, the methods applied here accommodated both bootstrap sampling and the component nature of SOFA score. We recommend that this approach be considered more widely for informative missingness in longitudinal data.

### P210

#### Do trialists adequately pre-specify their statistical analysis approach? A review and re-analysis

Lauren Greenberg[1], Vipul Jairath[2], Brennan C. Kahan[1]
[1]Queen Mary University of London; [2]Department of Medicine, Epidemiology and Biostatistics, Western University
**Correspondence:** Lauren Greenberg
*Trials* 2017, **18(Suppl 1):**P210

**Background**
Well-designed clinical trials are the gold standard for evaluating healthcare interventions. It is essential for the trial methodology to be pre-specified in the protocol in order to avoid issues such as selective reporting of outcome measures. However, little attention has been paid to whether trialists are adequately pre-specifying the method of analysis for their primary outcome in the trial protocol, or what impact inadequate pre-specification might have on trial results.
**Methods**
We re-analysed primary clinical outcome data from the TRIGGER trial to examine the impact that differing analytical approaches could have on the trial outcome. We varied several aspects of the analysis: (a) the patient population included in the analysis; (b) the analysis model used; (c) the set of covariates included in the model; and (d) methods of handling missing data. We then conducted a review of published trial protocols to assess how well the statistical analysis approach for the primary outcome was pre-specified.
**Results**
Our re-analysis of TRIGGER found that the choice of statistical analysis approach had a large impact on both the estimated treatment effect and p-value. Across the different analytical approaches, the estimated odds ratio ranged from 0.40 (95% CI 0.17 to 0.91; p-value 0.03) to 1.09 (95% CI 0.56 to 2.10; p-value 0.80). It was possible to obtain both significant and non-significant results by varying either the patient population included, the set of covariates used in the analysis model or the method of handling missing data. The review of published protocols is ongoing, however preliminary results indicate that most trial protocols do not adequately pre-specify their analysis approach for the primary outcome.
**Conclusions**
The statistical analysis approach can greatly influence trial results. It is essential that the planned analytical method is pre-specified in the trial protocol in order to avoid selective analysis reporting.

### P211

#### Effective graphical analyses of adverse events in DMC reports

Allison Furey, Robin Bechhofer
University of Wisconsin-Madison
**Correspondence:** Allison Furey
*Trials* 2017, **18(Suppl 1):**P211

The primary charge of a Data Monitoring Committee (DMC) is to monitor the safety of clinical trial subjects. Among the most important sources of safety data is adverse events (Aes) reported by investigators.
Often, the Sponsor's statistical analysis plan for the final study analysis simply indicates that Aes will be summarized by meddra system organ class (SOC) and preferred term. Lengthy tables of Aes are comprehensive, but may overwhelm DMC members with detail and fail to highlight relevant treatment differences, important constellations of related Aes, or answer key questions regarding the severity, impact, or timing of events.
The Statistical Data Analysis Center (SDAC) at the University of Wisconsin-Madison specializes in producing interim reports and analyses for DMCs. Our reports are graphically based, allowing DMC members to easily identify differences between treatment groups or over time and to review a large amount of information in a short amount of time. We employ various presentation styles, including graphics produced in R (bar charts, stacked bars, Kaplan-Meier plots, forest plots), and tables and listings produced in SAS; latex is used for layout and report production.

A major challenge in AE reporting is to separate signal from noise, drawing attention to important issues while not sacrificing completeness of reporting. Our standard suite of AE analyses employs a “Drill down” Approach, beginning with an overall summary of Aes falling into selected categories (serious, fatal, related to treatment, leading to treatment discontinuation, etc.), graphical summaries by SOC and of most common preferred terms, followed by incidence tables of preferred terms within SOC and listings of Aes of concern. Our standard displays provide visual information regarding severity as well as incidence, and highlight treatment comparisons between groups.

Flexibility is a key feature of our reports; analyses evolve depending on the stage of the trial as well as in response to DMC concerns, and are often tailored to characteristics of the subjects and/or treatments in the specific trial. We find graphical presentations useful, not only for aggregate data, but also for examining individual subjects – for example, to illustrate the relationship between Aes and other data (e.g., dosing, lab data). Custom graphical displays may also address, in aggregate or by subject, timing of Aes, recurrent events, or events of special interest.

This poster presents examples of innovative displays designed to respond to specific questions posed by the DMC, as well as our standard AE presentations for DMC reports.

### P212

#### A novel approach to analysis of clinical trials for rare cancers assuming symmetry

Emma Wang[1], Peter D. Sasieni[1], Bernard V. North[2]
[1]Queen Mary University, London; [2]Exploristics Ltd
**Correspondence:** Emma Wang
*Trials* 2017, **18(Suppl 1):**P212

##### Background

Rare cancers have complications in analysis due to limited recruitment, meaning the event of interest does not occur enough to accurately discern which treatment arm is better. Due to unclear knowledge of the best way of treating patients suffering from rare diseases, a disproportionately high number of deaths occur.

We propose a method of analysing clinical trials for rare diseases when comparing two treatments already in use, which can give a good indication of which treatment arm is better, that does not require sample sizes of the magnitude of conventionally-powered trials.

Merkel cell carcinoma (MCC), a skin cancer which recorded 1515 cases in the UK in a 10-year period, is one such rare disease. Currently, the main treatment method for MCC is prioritising surgery, then administering radiotherapy to eradicate remaining cancer cells.

It was postulated whether reversing this treatment order would be more efficacious. This question is analogous to comparing two treatments in use, because patients would receive access to both radiotherapy and surgery regardless of the outcome, and there are arguably no losers.

Hypothesis testing using conventional levels of Type I and II error would require in excess of 3000 patients, which is unfeasible to recruit, even across countries, leading such a trial to be underpowered.

We applied our new analysis method using the statistics associated with MCC.

##### Methods

The Type I error was redefined as probability of concluding a treatment was better than the other when in fact it was worse, and the minimum sample size was the sample size needed for this value of Type I error to be to 2.5%.

To conclude ‘superiority’ using our rules, the upper limit of two-sided 95% confidence interval of the hazard ratio (HR) observed had to be below 1.25, and the upper limit of two-sided 50% confidence interval had to be below 1.

Survival analysis was conducted with a patient having the endpoint of interest if they died within 2 years. We simulated the survival time of patients in a two arm trial with the treatment arm as the sole predictor and analysed the data using the Cox hazard model.

In simulations of 10,000, various sample sizes and true hrs of the treatment arms were modelled, with the power to conclude efficacy using the conventional null hypothesis, and the re-definition, compared.

##### Results

In all examples simulated pertaining to MCC, using our rules leaded to substantial gains in power, sometimes even a doubling.

The results of theoretical sample size equations had close concordance with the powers for various sample sizes observed in simulations.

##### Conclusion

By restricting the probability of making a wrong decision to be 2.5%, the analysis method we have proposed is more robust than generic non-inferiority tests. The interpretation of hypothesis testing from our rule is the patient may be informed, “on the balance of probability, this treatment is better”.

Our proposed analysis method means conducting clinical trials for rare diseases is worthwhile after all, potentially leading to better standard of care for patients suffering from them.

### P213

#### Evaluating treatment effect modification on the additive scale for the evaluation of predictive markers

Antonia Marsden[1], Richard Emsley[2], William Dixon[3], Graham Dunn[1]
[1]Centre for Biostatistics, School of Health Sciences, University of Manchester; [2]Centre for Biostatistics, School of Health Sciences, University of Manchester. MRC northwest Hub for Trials Methodology Research, UK; [3]Arthritis Research UK Centre for Epidemiology, Manchester Academic Health Science Centre, University of Manchester
**Correspondence:** Antonia Marsden
*Trials* 2017, **18(Suppl 1):**P213

Predictive markers are variables that identify patient subgroups with differential response to treatment and can be useful in guiding treatment decisions. Practically, predictive markers are those found to moderate the relationship between treatment and an outcome. However, the presence of treatment effect modification is dependent upon measurement scale of the outcome. If the absolute effect of treatment varies across patient subgroups, treatment effect modification is present on the additive scale. Alternatively, if the relative effect of treatment varies across patient subgroups, treatment effect modification is present on the multiplicative scale.

Treatment effect modification on the additive scale is generally perceived to be of primary interest for explaining differential treatment response because absolute treatment effects do not depend on baseline risks which may differ between patient subgroups. For example, if age is, regardless of treatment, associated with the outcome of interest, the baseline risk will vary across age subgroups. If the relative treatment effect, say the relative risk, is found to be similar across the age subgroups, this implies variation in the absolute treatment effect across the subgroups. Specifically, this implies that patients in the subgroup(s) with a lower baseline risk have a smaller absolute treatment compared to patients in the subgroup(s) with a higher baseline risk. Since the absolute treatment effect conveys the absolute magnitude of the treatment response, this variation will likely be of interest.

However, in clinical trials with binary and time-to-event outcomes, treatment effect modification is often assessed only on the multiplicative measurement scale as this corresponds to a comparison of the more commonly presented relative treatment effect measures (relative risks, odds ratios, hazard ratios) across patient subgroups. This is usually obtained from the widely used regression models for these outcome measures, i.e. The logistic regression model and the Cox proportional hazards regression model, by the inclusion of a product term between treatment and the predictor of interest. The analysis of treatment effect modification on the additive measurement scale can be less easy to obtain in these settings, particularly for time-to-event outcomes due to the dependency on time.
This works aims to highlight why an analysis of treatment effect modification on the additive scale is more informative in the evaluation of markers predictive of differential treatment response and to present how this can be performed in practice. We propose the use of a novel measure, the Ratio of Absolute Effects (RAE) measure, as an approach for the assessment of treatment effect modification on the additive scale which can be calculated from the more commonly used multiplicative regression models used for binary and time-to-event outcomes. We suggest this measure to be particularly useful for time-to-event outcomes as it is time invariant. Also discussed is the use of alternative regression models on the additive scale (e.g. The additive hazards model) from which effect modification on the additive scale can be directly assessed.

### P214

#### Comparison of global statistical analyses in patients with hyper-acute stroke: assessment of randomised trials of transdermal glyceryl trinitrate, a nitric oxide donor

Lisa Woodhouse[1], Polly Scutt [1], Stuart Pocock[2], Alan Montgomery[1], Nikola Sprigg[1], Philip M. Bath[1]
[1]University of Nottingham; [2]London School of Hygiene & Tropical Medicine
**Correspondence:** Lisa Woodhouse
*Trials* 2017, **18(Suppl 1):**P214

**Background**
Data from a subgroup of the Efficacy of Nitric Oxide in Stroke trial (ENOS-early; concerning patients randomised within 6 hours of ictus, a pre-specified subgroup) and the Rapid Intervention with Glyceryl trinitrate in Hypertensive stroke Trial (RIGHT), suggest that glyceryl trinitrate (GTN), when given early, improved dependency, death, disability, cognitive impairment, mood disturbance, and quality of life. However, individual outcomes do not provide a global estimate of effect. Previous acute stroke trials have used global tests to assess the overall effect of treatment on a group of outcomes: NINDS and IMAGES (the National Institute of Neurological Disorders and Stroke RT-PA trial and the Intravenous Magnesium Efficacy in Acute Stroke trial; Wald test for binary outcomes) and CARS (Cerebrolysin and Recovery After Stroke trial; Wei-Lachin test for ordinal and continuous outcomes). Transdermal GTN is a candidate treatment for ultra- and hyper-acute stroke, potentially acting through reperfusion, haemodynamic and cytoprotectant effects.

**Methods**
The global effects of ultra- or hyper-acute administration of GTN were tested using three statistical approaches: the Hotelling T2 test (combines continuous variables), and Wei-Lachin and Wald tests. Analyses using ordinal logistic regression and multiple linear regression were also performed to test the individual effects of GTN on each outcome. Raw (and dichotomised) outcome data at 90 days included telephone assessments of dependency (modified Rankin Scale, MRS >2), disability (Barthel index, BI < 60), mood (short Zung depression scale, ZDS > 70), cognition (t-Mini Mental state examination, tmmse < 14) and quality of life (health utility status, HUS < 0.5, as derived from euroqol-5D-3 level). Data are odds ratio (OR), mean difference (MD), Mann–Whitney estimates (MW) and T2 statistic.

**Results**
312 patients (GTN 168, no GTN 144) were randomised within 6 hours of ictus into ENOS-early ($n = 273$) and RIGHT ($n = 39$). GTN improved certain individual and global outcomes for both the ENOS-early and RIGHT trials respectively: Individual tests MRS: OR 0.55, (p = 0.0055); 0.27, ($p = 0.0306$) BI: MD 13.5, ($p = 0.0029$); 25.4, ($p = 0.0724$) ZDS: MD −10.3, ($p = 0.0013$); −14.3, ($p = 0.0631$) tmmse: MD 3.5, ($p = 0.0007$); 4.3, ($p = 0.1151$) HUS: MD 0.09, ($p = 0.0753$); 0.21, ($p = 0.0618$) Global tests Hotelling T2: T2 24.91, ($p = 0.0087$); 9.85, (p = 0.1763) Wei-Lachin: MW 0.64, (p = 0.0018); 0.73, ($p = 0.0301$) Wald: OR 0.52, ($p = 0.0011$); 0.38, ($p = 0.0826$).

**Conclusions**
GTN improved global aggregates of dependency, disability, mood, cognition and quality of life data. This exploratory finding is being tested prospectively in the ongoing 850-patient RIGHT-2 trial. Though individual test results for RIGHT suggest that GTN only had a significant effect on dependency (MRS), global analysis of the data (using the Wei-Lachin test) suggested that GTN improved all outcomes. Reporting global tests adds summary information on overall treatment effects. Further, it may be advantageous to base the primary outcome on a global analysis since global tests are statistically more efficient; in this case, individual outcomes would be presented in pre-specified secondary analyses. The Wei-Lachin test may be preferred since it allows analysis of ordinal and continuous variables; in contrast, the Wald test only analyses binary outcomes, and the Hotelling T2 test does not take account of direction of effect.

### P215

#### Rationale for using an ordinal primary outcome in clinical trials for the prevention of recurrent stroke and transient ischaemic attack

Lisa Woodhouse[1], Jason P. Appleton[1], Stuart Pocock[2], Alan Montgomery[1], Nikola Sprigg[1], Philip M. Bath[1]
[1]University of Nottingham; [2]London School of Hygiene & Tropical Medicine
**Correspondence:** Lisa Woodhouse
*Trials* 2017, **18(Suppl 1):**P215

**Background**
Due to major advances being made in clinical trials for prevention of cardiovascular events (including stroke and transient ischaemic attack, TIA), and the falling risk of recurrent events, cardiovascular prevention trials are increasing in size. Since the number of trials has also increased, it is becoming more difficult to recruit patients into new trials. New strategies are now needed to reduce trial sample sizes and to amplify the potential to demonstrate benefit. The international Triple Antiplatelets for Reducing Dependency after Ischaemic Stroke (TARDIS) trial assessed the safety and efficacy of intensive (combined aspirin, dipyridamole and clopidogrel) versus guideline (aspirin/dipyridamole, or clopidogrel alone) antiplatelets given for one month in patients with acute stroke or transient ischaemic attack (TIA).

**Design**
Vascular prevention studies typically count outcomes as dichotomous events (e.g. Event vs no event) although this is inefficient statistically and gives no indication on the severity of the recurrent event. Recurrent vascular events, such as stroke, could therefore be polychotomised with ordering of outcome events determined by severity. A retrospective analysis of published vascular prevention trials (including antithrombotic, antihypertensive, lipid lowering, carotid surgery, and hormone replacement therapy) suggested that polychotomised outcome measures provide information on both events and their severity, generate smaller numbers-needed-to-treat, and may be more efficient statistically.

**Methods**
In the context of acute stroke trials, the modified Rankin scale (MRS) is often used as the primary outcome measure, due to its sensitivity to treatment effects. The MRS is a seven level ordered categorical scale (0: No symptoms, 1: No significant disability, 2: Slight disability, 3: Moderate disability, 4: Moderately severe disability, 5: Severe disability, 6: Death) that assesses independence, dependency and death. The primary objective of the TARDIS trial was to assess treatment effect on recurrence and severity of that recurrence at 90 days. Therefore, the primary outcome consisted of a combination a) the type of recurrent event (stroke or TIA) and b) the score from the MRS taken at three months. This produced a six level ordered categorical polychotomised scale with the following structure; Fatal stroke (MRS =6)/Severe non-fatal stroke (MRS =4 or 5)/Moderate stroke (MRS =2 or 3)/Mild stroke (MRS =0 or 1)/TIA/No recurrent event. The assessment of this primary outcome measure utilised the shift approach, with the use of ordinal logistic regression analysis.

#### Discussion

The TARDIS trial was the first vascular prevention trial to assess prospectively both recurrence and its severity, rather than recurrence alone. This novel approach both increases statistical power through comparing the difference in the distribution across the whole scale of severity between the treatments, and allows the effect of treatment on severity to be assessed. Such an approach can reduce trial sample size and ultimately costs, whilst improving statistical efficiency and amplifying the potential to demonstrate a treatment effect. Data will be presented once the main findings have been presented in late 2016.

### P216

### Log-likelihood is the best correlative measure to estimate the cutpoint for a continuous prognostic variable: a Monte Carlo simulation study

Mansour Sharabiani, Clare Peckitt, Gerhardt Attard
The Royal Marsden NHS Trust
**Correspondence:** Mansour Sharabiani
*Trials* 2017, **18(Suppl 1):**P216

#### Background

Stratification of patients into high- and low-risk categories using a cutpoint for a continuous prognostic variable has important applications in clinical decision making. Different approaches including biological determination, median value, and clustering as well as using correlative measures such as logrank test, minimum p-value, hazard ratio, and log-likelihood have been used to determine the cutpoint. Here we try to choose the most reliable correlative measure using Monte Carlo simulation. We also apply the chosen measure to biological data (androgen receptor [AR] gene copy number) from castration-resistant-prostate-cancer (CRPC) patients where it is assumed, based on previous studies, that AR-gain (higher number of copies of AR) patients have higher hazard rates of survival than AR-Normal patients.

#### Methods

Assuming log-hazard-ratio is a logistic function of continuous prognostic variable, the midpoint of the sigmoid curve (x_mp) would be a natural choice for the cutpoint. 100,000 survival datasets were generated via Monte Carlo simulations using R language. Each simulated dataset included 200 observations (x) with exponential distribution (similar to the number of the patients and the distribution of AR-copy numbers in the trial) and log-hazard-ratio (y) as a logistic function of x. Parameters of the steepness of the curve and location of the midpoint (x_mp) were randomly assigned in each run. For every simulated dataset, the best cutpoint was sought via the following iterative steps: (i) assigning 0 to all observations below copy number x_i and 1 to all observations equal to or above copy number x_i, (ii) fitting Cox model (for x_i), (iii) using the maximum values of the statistics of survival modelling including Hazard Ratio, Log-Likelihood - or Cox-Snell Pseudo-R-Squared (RSQ) -, Concordance Index, Wald-test, and Log-Rank-test as indicators (correlative measures) of the cutpoint, (iv) calculating the difference between the cutpoints suggested by each correlative measure and the true cutpoint (x_mp). Altogether, six sets of 100,000 differences along with their medians and interquartile ranges (IQR) were estimated. The statistical measure associated with the smallest absolute median and IQR was chosen as the best correlative measure. The chosen measure was used in the trial data to determine the optimal cutpoint for AR-copy number. We also used bootstrapping to increase reliability of the estimated cutpoint in the trial data.

#### Results

Median and IQR of the differences between true cutpoint (x_mp) and the the copy numbers indicated by the highest values of Hazard Ratio, Concordance Index, Wald test, Log-Rank, RSQ, and Log-Likelihood were −13.39 (45.38), −3.13 (5.32), −3.10 (3.60), −2.82 (3.43), −2.06 (3.24), and −2.06 (3.24), respectively. Consistent results were also observed using simulated AR-copy numbers with normal distribution. Thus, Log-Likelihood (or interchangeably RSQ) was chosen as the recommended correlative measure and was used to determine the optimal AR-copy number cutpoint for stratification of CRPC patients.

#### Conclusion

Among various statistical measures of survival, Log-Likelihood is the best correlative measure for estimating optimal cutpoint of a continuous prognostic variable with normal or exponential distribution. Wald and Log-Rank tests are slightly less reliable and Hazard Ratio is the least reliable correlative measure.

### P218

### SWOG s1700: an institutional cluster-randomized trial of a surgical lymph node specimen collection kit in the cooperative group setting

Jieling Miao, Yingqi Zhao, Jim Moon, Mary W. Redman
SWOG Statistics and Data Management Center, Fred Hutchinson Cancer Research Center
**Correspondence:** Jieling Miao
*Trials* 2017, **18(Suppl 1):**P218

#### Background

Approximately 60,000 patients annually undergo resection for non-small cell lung cancer (NSCLC) in the US. Most of them will not achieve long-term survivorship and the status of nodal involvement is the most powerful determinant of prognosis. Accurate pathologic nodal staging requires the combination of surgical dissection of the appropriate hilar and mediastinal lymph nodes and thorough pathologic examination of lymph modes present within the lung resection specimen. S1700 or SILENT (Strategies to Improve Lymph Node Examination of Non-Small Cell Lung Tumors), a trial proposed by SWOG, is designed to evaluate a lymph node specimen collection kit. It is anticipated that this simple intervention on how the surgeon does his/her lymph node sampling, will improve the accuracy of pathologic nodal staging of resected lung cancer. It was determined that a cluster randomized trial (CRT) design is necessary to address this question. Conduct of a CRT is rarely done (to almost never) in the Cooperative Groups within the US.

#### Methods

Institutions will be randomized to implement the intervention versus usual care. Randomization will be stratified by institution characteristics (3 factors: institutional volume, thoracic surgery fellowship training program, dedicated general thoracic surgeon present). In order to randomize all institutions at the same time, a run-in phase will be implemented to allow for sites to obtain institutional and regulatory approvals. In addition, objectives of the run-in phase are to provide a more accurate assessment of local accrual and preliminary estimates of outcomes. The primary objective of this study is to compare the 3-year disease free survival (DFS) among patients at institutions randomized to implement the intervention to those randomized to usual care. The secondary objective is to compare the frequency of patient up-staging (from cn0/1 to pn1/2/3) following surgical resection among patients receiving intervention to those receiving usual care. Given feasibility considerations, the planned goal is to limit participation to 40 institutions (20 randomized to implement the intervention and 20 to continue with usual care). Given historical data, it is estimated that the intraclass correlation coefficient is 0.01. Sample size calculations were based on Xie & Waksman. (Stat Med. 2003 Sep 30;22(18):2835–46).

#### Results

The study design is based on a design with 80% power to detect a 50% improvement in DFS (HR =0.67) at the 1-sided 0.025 level. We assume uniform accrual and an average accrual rate of 15 patients/site/year. Under independence, the total sample would be 568. Accounting for within institution correlation, the total accrual is 670 patients (an inflation of 18%), accrued over 2 years with 3 years of follow-up.

#### Discussion

In an era of increasing costs for cancer care, low-cost and relatively simple interventions such as the one being evaluated in SILENT are very valuable. Careful consideration of design and implementation can lead to a valuable resource and address an important yet simple question.

### P220

#### An assessment of the design and dissemination of phase I clinical trials: where can we improve?

Bethan Copsey[1], Ayodele Odutayo[2], Jonathan Cook[2], Susan Dutton[2], Douglas Altman[2], Sally Hopewell[2]
[1]University of Oxford; [2]Nuffield Department of Orthopaedics, Rheumatology and Musculoskeletal Sciences, University of Oxford
**Correspondence:** Bethan Copsey
*Trials* 2017, **18(Suppl 1):**P220

**Background**
Phase I trials involve the early testing of investigational medicines in humans in order to assess their safety, tolerability and pharmacokinetics. Questionable design and conduct of phase I trials has led to long-term morbidity and mortality. There is limited information publicly available regarding how these trials are conducted, monitored and disseminated. A systematic methodological review of the ethical submission for phase I trials was carried out to address this gap.
**Methods**
A representative sample (n = 426) of clinical trial protocols that received ethical approval by the UK Health Research Authority (HRA) in 2012. We extracted details related to study design and methods from the protocols on phase I studies. Additionally, information on serious adverse events (SAEs) from submitted clinical study reports (CSRs) and searched for publications (by April 2016) of the completed trials was collated. Findings were narratively summarised.
**Results**
Of the 426 HRA-approved trial protocols, 54 were phase I trials (17 oncology; 37 non-oncology). Forty-five (83%) were industry funded and 17 (31%) were first-in-human studies. All trials were registered in a trial registry, although registry details were publicly available for only 21; as per EU regulations. Across the included studies there were 869 participants; the median sample size was 27 (interquartile range 18 to 41).
Of the first-in-human studies, 13 specified an observation period between administration of the study drug to the first and subsequent participants. Only one study provided justification for this observation period. Thirteen first-in-human studies used biological agents but only 5 of 13 used the MABEL (minimum anticipated biological effect level) for calculating the starting dose or justified not doing so.
Of the 54 phase I trials, 32 have been completed and 24 submitted CSRs to the HRA as of April 2016. No deaths occurred but 11 SAEs were reported, of which 3 were deemed potentially related to the study treatment. All treatment-related SAEs occurred in non-oncology trials.
After a median 2.7 years since completion, only 3 of the 32 fully completed phase I trials have been published and only 10 of these 32 trials have a publicly accessible trial registry entry. None of the trials with SAEs have been published.
**Discussion**
These findings suggest that phase I trials are generally safe, however there are important opportunities to improve the design, conduct and dissemination of these studies. Methodological gaps exist which should be addressed when planning phase I trials, particularly for dose escalation studies. Much greater transparency through the public registration and dissemination of findings from phase I trials is needed to improve the safety and conduct of future studies.

### P221

#### Practical sample size re-estimation of propensity score analysis for prospective study

Naoki Ishizuka[1], Takeharu Yamanaka[2], Noriko Tanaka[3]
[1]Cancer Institute Hospital; [2]Yokohama City University; [3]National Center for Global Health and Medicine
**Correspondence:** Naoki Ishizuka
*Trials* 2017, **18(Suppl 1):**P221

**Background**
Sample size must be determined when one start any prospective study regardless of it is intervention or observational. The recent popularity of propensity score rapidly increases its application in prospective observational studies with time-to-endpoint in various areas including cancer or cardiovascular disease and some would expect it as an alternative of confirmatory trials. However, a limited number of papers have discussed sample size calculation. We proposed practical sample size re-estimation in mid-course of the study. The approach provides not only statistical power but also the incorporating with interim analysis which have to adjust type I error.
**Background**
There is a couple of issues in practice. One of issues is that it depends on the distribution of propensity score. The score is usually estimated by logistic regression. However, it is not easy to assume prior to commencing the study. Another one is that some factor which has association with treatment selection but is not correlated with endpoint decrease the precision of confidence interval for the estimate of treatment effect. As result, it leads to decreasing statistical power of test as previous report warned. However, identifying these factors to be excluded would contradict the nature of propensity score analysis which collects data not to miss confounding factors as much as possible. Furthermore simple stratified analysis, ex Cox regression, is enough if it is possible to identify these factors in advance.
**Methods**
We assume the situation that one would assess the new treatment compared to the standard one. Calculate the sample size tentatively if one assumes alpha level, power and an effect size delta. If time to event is a primary endpoint, expected number of events is determined by the method of Schoenfeld and the variation. Estimate propensity score when the sample size or the number of events reaches tentative sample size or expected number of events. Use stratified logistic or stratified Cox model to estimate the parameter of the effect size. Calculate the inflation coefficient - Which is defined as follows = (Observed Standard Error)^2/(1/(_1 × _2 × D)) where _1,_2 are the fractions of each treatment group and D is the tentative expected number events for time to event. =(Observed Standard Error)^2/(Assumed Variance) for binary endpoint. Calculate the target sample size or the number of events as a product of inflation coefficient - and tentative sample size or the number of events. Do the interim analysis ad Information time as 1 if one would like to plan. If the interim analysis is not significant or one has not done it, do the final analysis.
**Results**
The operational characteristics concerning the statistical power for various scenarios in which there is no correlation between the factor of treatment choice and endpoint were examined by the simulation study. The result guaranteed the statistical power as planned.
**Conclusions**
Our approach keeps the statistical power without any assumption of propensity score including the distribution and the correlation between the endpoint and the factors of the treatment choice.

### P222

#### Evaluating personalised treatment recommendations using randomised controlled trials

Matthias Pierce, Richard Emsley
University of Manchester
**Correspondence:** Matthias Pierce
*Trials* 2017, **18(Suppl 1):**P222

**Objective**
To explain, demonstrate and compare methods for evaluating personalised treatment recommendations using a standard, two-arm, parallel randomised controlled trial.
**Background**
The modern paradigm of stratified medicine (also termed personalised or precision medicine) seeks to move beyond a one-size-fits all approach, that treats patient populations as a whole, towards one that identifies patient strata with different disease pathways or responses to treatment. A major aspect of stratified medicine is to provide personalised treatment recommendations (PTR'S): an algorithm that recommends treatment based on the patient's predicted treatment response using biomarkers, a patient's measureable characteristics collected at clinical visit. A PTR may be constructed using a single biomarker, or using multiple biomarkers. After estimating a PTR, the next step is to assess whether the expected outcome under a PTR improves on the expected outcome under an alternative policy – one where either everybody receives the treatment or everybody receives the control condition. The evaluation of a PTR differs from the evaluation of prognostic or diagnostic models because, for any individual, the object of inference (whether a subject benefited from treatment) remains unobserved. This is because the individual treatment effect cannot be separated from prognostic effects. Therefore standard methods of model evaluation, for example ROC-curve analysis, are inappropriate in this context.

**Methods**

This presentation will cover two methods for evaluating a PTR using a standard, two-armed randomised controlled trial. The first, termed the inverse probability weighting (IPW) approach, uses a weighted average of the outcome in those lucky to have been randomised to the treatment they were recommended under the PTR. The second is an augmented version of the IPW (AIPW), developed using semi-parametric theory, that borrows information from a regression model for the outcome under treatment or control, to establish a more efficient estimator. Monte-Carlo simulations are used to compare the statistical properties of the IPW and AIPW methods using a range of data generating scenarios. These methods will be demonstrated with application to data from a randomised controlled trial for Chronic Fatigue Syndrome Patients, using the user-written Stata command ptr.ado. Inference for these parameters will also be discussed.

**Results**

Simulations demonstrate that the AIPW method is consistently shown to be more efficient, even when the parametric model for the outcome used in the AIPW procedure is misspecified.

**Conclusions**

The evaluation of a PTR is qualitatively different from the evaluation of a model used for diagnosis or prognosis. There are two methods available for establishing whether the outcome under a PTR is an improvement (or not) on an alternative policy where everybody is given the treatment/control conditions. These methods are easily implemented in standard statistical software; for example using our user-written Stata command ptr.ado. Of the two methods, the AIPW is demonstrably more efficient than the IPW.

### P223

#### Design, conduct, and analysis of a master protocol within an evolving landscape of standard of care: the lung-map trial

Mary Redman, James Moon, Shannon McDonough, Jieling Miao, Katie Griffin, Michael LeBlanc
Fred Hutchinson Cancer Research Center
**Correspondence:** Mary Redman
*Trials* 2017, **18(Suppl 1):**P223

The Lung- MAP trial (Lung Cancer Master Protocol), launched in 2014, is an umbrella protocol to evaluate targeted therapies in biomarker selected patients for previously-treated stage IV or recurrent non-small cell lung cancer. It is the first precision medicine trial launched with the support of the National Cancer Institute in the United States. Moreover, Lung-MAP is designed as a pathway for FDA approval of investigational therapies that successfully meet study objectives.

Lung-MAP activated with 4 biomarker-driven sub-studies and one sub-study for patients with no matching biomarkers; all sub-studies were randomized with docetaxel as the control in 4 of 5 sub-studies. While the standard of care (docetaxel) had been unchanged for decades in this patient population, within the first year of the study, the Checkmate 017 trial (Brahmer NEJM 2015), demonstrating that nivolumab is superior to docetaxel in this patient, changed the treatment paradigm for this population.

In December 2015, a major revision of the trial was implemented with modifications to the patient population and design of the biomarker-driven sub-studies in response to the approval of immunotherapies in our study population. As of November 3, 2016, 4 sub-studies have been closed to accrual, 1 new non-match sub-study has been activated, 1 new biomarker-driven sub-study is expected to open to accrual by the end of 2016, 1 new non-match sub-study for immune-therapy (IO) exposed patients is expected to activate in the first quarter of 2017, and an additional biomarker-driven sub-study is expected to be activated mid-2017. The anticipated study schema is included below.

The Lung-MAP trial is a continually evolving study. The study team continues to evaluate new biomarker/investigational therapy pairs, including immunotherapy drugs and biomarkers, and combinations of therapies. Conduct of such a study requires a substantial amount of effort and on-going attention beyond the conduct of a stand-alone clinical trial. This presentation will provide an overview of the current status of Lung-MAP, both active and closed studies, discuss some lessons learned in the conduct of these so-called platform trials, and a view into the future of Lung-MAP.

### P224

#### Beyond blinding: a systematic review to explore performance bias in surgical RCTs

Natalie Blencowe, Barry G. Main, Jane M. Blazeby
University of Bristol
**Correspondence:** Natalie Blencowe
*Trials* 2017, **18(Suppl 1):**P224

**Background**

Performance bias arises from unintended deviations from the intended intervention, comparator or co-interventions that occur differentially by allocated group. Conventionally, it can be reduced through blinding of healthcare providers and patients; however, this represents a major challenge in surgical settings and other strategies are therefore required. Standardisation of surgical intervention and co-interventions, and monitoring adherence to these standards, represents one solution for reducing performance bias. The aim of this study, therefore, was to systematically explore the issue of performance bias in randomised controlled trials in surgery, to inform the design and delivery of future studies.

**Methods**

In order to explore the issue of performance bias in depth, a narrow clinical field (appendicitis) was selected. Rcts evaluating at least one surgical intervention (defined as procedures that cut a patient's tissues, involving the use of a sterile environment, anaesthesia, antiseptic conditions, surgical instruments, and suturing or stapling) for patients with appendicitis were identified. Because there is no formal tool for assessing performance bias, information from existing literature relating to various aspects of performance bias was used to guide data extraction: i) blinding (Cochrane Risk of Bias tool), ii) standardisation (CONSORT-NPT and SPIRIT statements). An inductive approach was used, whereby an initial extraction form was used and where new themes relating to performance bias were identified, the form was modified to incorporate these and all trials reviewed using the new form.

**Results**

45 rcts met the inclusion criteria. Six compared surgical and non-surgical treatments, and 39 compared different surgical approaches (open versus laparoscopic surgery, n = 35; laparoscopic versus single-port surgery, n = 4). In the six RCTs comparing surgical and non-surgical treatments, blinding of participants was not undertaken and there was no information relating to healthcare professionals or trial personnel. In the 39 comparing different surgical procedures, information about blinding was rarely reported. Eight, seven, and five studies reported that blinding of participants, healthcare professionals and trial personnel was attempted, respectively. Just one RCT reported that the success of blinding was evaluated. Data extraction and analysis is ongoing and further results (relating to standardisation) will be available for presentation at the conference.

### Conclusion

Preliminary results from this study indicate that surgical RCTs are likely to be at high risk of performance bias. Although blinding of surgeons performing operations was not possible in this clinical area, blinding of patients, other healthcare professionals and trial personnel was plausible yet rarely undertaken. This may be because existing guidance is difficult to apply in a surgical setting. A potential solution would be to improve the process of quality assurance in rcts, by i) clearly defining interventions and co-interventions, ii) standardising their delivery, and iii) careful monitoring and reporting of adherence to these standards. Further work is required to explore how this might be achieved in surgical RCTs.

### P225

#### Network metanalysis benchmarking the technological development of implantable medical devices

Catherine Klersy, Valeria Scotti, Luigia Scudeller, Chiara Rebuffi, Carmine Tinelli, Annalisa De Silvestri
IRCCS Fondazione Policlinico san Matteo
**Correspondence:** Catherine Klersy
*Trials* 2017, **18(Suppl 1):**P225

This abstract is not included here as it has already been published.

### P226

#### Assessment of the reporting quality of rcts conducted in Saudi Arabia: a systematic review

Nada Alsowaida[1], Doaa Bintaleb[2], Hadeel Alkofide[3], Hisham Aljadhey[4], Tariq Alhawassi[5]
[1]Medication Safety Research Chair, Pharmacy services, King Saud University Medical City, Riyadh, Saudi Arabia; [2]Investigational Drugs and Research Unit, King Saud University Medical City, Riyadh, Saudi Arabia; [3]College of Pharmacy, King Saud University, Riyadh, Saudi Arabia; [4]Saudi Food and Drug Authority, Medication Safety Research Chair, Riyadh, Saudi Arabia; [5]Medication Safety Research Chair, College of Pharmacy, King Saud University, Riyadh, Saudi Arabia
**Correspondence:** Nada Alsowaida
*Trials* 2017, **18(Suppl 1):**P226

##### Background

Randomized controlled trials (RCTs) are considered the gold standard to assess the efficacy and safety of new treatment interventions and compare conventional therapies. RCTs are used to support decision-making, and guidelines recommendations. However, despite their clinical importance, RCTs have some limitations as they are at high risk for bias, can over and/or underestimate treatment interventions, which limit their generalizability. It's estimated that poor quality trials have led to 30% - 40% overestimation of the treatment. Therefore, the quality of reported RCTs is still questionable and multiple studies have concluded that RCTs are yet hindered by several limitations making risk-benefit assessment, which is an essential element for RCTs quality, a challenge in certain medical conditions for healthcare professionals. With the largely emerging data and new treatments that required pharmaceutical companies to do more RCTs, the need for assessing the quality of RCT becomes increasingly important. The Consolidated Standards of Reporting Trails (CONSORT) statement is a tool designed to assess the quality of RCTs reported and significantly improve the quality of RCTs. To our knowledge there is no current data in the literature regarding the quality of RCTs conducted in Saudi Arabia (KSA). Given the increasing number of RCTs being conducted in the region, it is essential to gain an understanding on the quality of reporting of these RCTs, which might impact future regulations for conducting such studies in the country.

##### Objective

To assess the reporting quality of RCTs conducted in KSA from 2005 and above using the CONSORT tool.

##### Method

Electronic search of the following databases: Cochrane Central Register of Controlled Trials (CENTRAL), EMBASE, MEDLINE via Ovid will be conducted. An attempt to identify unpublished data by searching clinical trial registries, through clinicaltrails.gov, and the Saudi Food and Drug Administration (SFDA) registry will be conducted. The search strategy will contain a combination of mesh terms and keywords relevant to the study design. Identified RCTs will be exported to Endnote X7 to check and remove any duplication. All titles and abstracts of identified RCTs will be screened by two investigators for potential relevance. Reference lists of potential studies, systematic reviews and meta-analysis will be also reviewed manually to identify relevant original RCTs. Search will be limited to RCTs either phase II, III and IV, published in 2005 and above in both English and Arabic language. Studies conducted in KSA as part of international multicenter RCTs, non-therapeutical RCTs will be excluded. The protocol of this study was submitted for publication to the International prospective register of systematic reviews PROSPERO.

##### Results

Pending

##### Discussion

This study will assess the quality of reporting of RCTs conducted in KSA given the increasing number of RCTs being conducted in the region and the limited data in the literature regarding the quality of RCTs reporting conducted. Findings achieved from this STUDY might help in identifying CURRENT strengths and gaps that may impact the Good Clinical Practice in the clinical setting in KSA.

### P227

#### Network meta-analysis of antiembolic interventions: adjustment for confounders

Larisa Tereshchenko[1], Charles Henrikson[1], Joaquin Cigarroa[1], Jonathan Steinberg[2]
[1]Oregon Health and Science University; [2]Arrhythmia Institute of The Valley Health System
**Correspondence:** Larisa Tereshchenko
*Trials* 2017, **18(Suppl 1):**P227

##### Background

The goal of this study was to compare confounding effect of patient population characteristics on the comparative effectiveness of individual antiembolic interventions in non-valvular atrial fibrillation (AF): novel oral anticoagulants (NOACs) (apixaban, dabigatran, edoxaban, rivaroxaban), vitamin K antagonists (VKA), aspirin, and the Watchman device.

##### Methods

We performed network meta-analysis of randomized clinical trials (RCTs) that enrolled 200 patients with non-valvular AF, mean or median follow-up Six months, with published reports in the English language. NOAC phase II studies were excluded. Placebo/control arm received either placebo or no treatment. All-cause mortality served as the primary outcome. Results of unadjusted and adjusted meta-regression analyses were compared. The following confounders were included, one-by-one: time in therapeutic range (TTR), CHADS2 score, mean/median duration of follow-up, mean age, the percentage of males, the percentage of VKA-naïve, the percentage of secondary prevention patients.

##### Results

A total of 21 RCTs (96,017 non-valvular AF patients; median age 72y; 65% males; median follow-up 1.7y). In unadjusted analysis, in comparison to placebo/control, use of aspirin (OR 0.82 (95%CI 0.68-0.99)), VKA (OR 0.69 (95%CI 0.57-0.85)), apixaban (OR 0.62 (95%CI 0.50-0.78)), dabigatran (OR 0.62 (95%CI 0.50-0.78), edoxaban (OR 0.62 (95%CI 0.50-0.77), rivaroxaban (OR 0.58 (95%CI 0.44-0.77)), and the Watchman device (OR 0.47 (95%CI 0.25-0.88)) significantly reduced all-cause mortality. Apixaban (OR 0.89 (95%CI 0.80-0.99)), dabigatran (OR 0.90 (95%CI 0.82-0.99)), and edoxaban (OR 0.89 (95%CI 0.82-0.96)) reduced risk of all-cause death as compared to VKA. Life-saving effect of Watchman device and NOACs was supported not only by 95% confidence intervals (CIS) but also, importantly, by 95% probability intervals (PRIs). However, 95% PRI for aspirin crossed the 'no effect' line, indicating that life-saving effect of aspirin might not be confirmed in future RCTs if ever conducted. After adjustment for

RCT population characteristics (TTR, duration of follow-up, and CHADS2), no antiembolic intervention was statistically significantly better than placebo/control, and there was no significant difference between VKA and NOACs, or other antiembolic interventions. Conclusion: Adjusted meta-regression analysis allows to study confounding effects of RCT population characteristics on results of network meta-analysis.

### P228

#### Adjusting trial results for biases in meta-analysis: combining generic evidence on bias with detailed trial assessment

Kirsty Rhodes[1], Rebecca M. Turner[1], Jelena Savovic[2], Roy Elbers[2], Hayley Jones[2], David Mawdsley[2], Jonathan AC. Sterne[2], Julian PT. Higgins[2]
[1]MRC Biostatistics Unit; [2]University of Bristol
**Correspondence:** Kirsty Rhodes
*Trials* 2017, **18(Suppl 1):**P228

**Background**
Systematic reviews of randomised controlled trials provide the best evidence on the benefits and harms of healthcare interventions. However, trials within meta-analyses are often affected by varying amounts of internal bias caused by methodological flaws. Currently, there is no consensus over how to make allowance for biases in meta-analysis. Two methods for adjustment for within-trial biases in meta-analysis have recently been proposed. The first uses empirical (generic) evidence on the magnitude of biases observed in a large collection of meta-analyses; the second uses expert opinion informed by detailed assessment of the potential biases affecting each trial. The objectives of this research are to investigate the extent to which these two approaches agree, and to explore how they could be integrated in order to gain the advantages of both.
**Methods**
To investigate agreement between generic evidence and detailed trial assessment, we asked three assessors with access to summary trial descriptions to rank pairs of trials from 30 sampled meta-analyses according to severity of bias. We compared the assessor rankings to rankings based on a bias model fitted to the sampled meta-analyses. Analyses were performed for biases associated with sequence generation, allocation concealment and blinding. Subsequently, we explored methods for bias adjustment based on bias distributions derived from generic evidence, detailed trial assessment or combinations of the two. Generic distributions were derived from a hierarchical model fitted to 64 meta-analyses from the Cochrane Database of Systematic Reviews. Opinion-based distributions were averaged across 12 assessors who read summary information on each trial in a new meta-analysis, and independently gave their opinions on bias. We developed three different approaches to combine generic evidence with detailed trial assessment. The first method statistically combines the generic and opinion-based bias distributions. In two alternative methods, assessors are provided with generic bias distributions and summary trial information, and asked to give their opinion on where in the distribution the particular trial might lie (numerically or by selecting broad areas of the distribution). In two case study meta-analyses, we adjusted for bias according to the set of distributions derived using each of the three approaches.
**Results**
Good agreement was observed between data-based and opinion-based approaches to ranking pairs of trials according to risk of bias. Among the assessor opinions judging that one trial was more biased, the proportion that agreed with the ranking based on evidence-based fitted biases was highest for allocation concealment (79%) and blinding (79%) and lowest for sequence generation (59%). In an example meta-analysis, bias-adjustment based on generic evidence had the effect of shifting the intervention odds ratio towards the null by 28%, and between-trial variance reduced substantially by 56%. Expert opinions have been obtained recently and the final bias adjustment results based on these are pending.
**Discussion**
Adjustment for biases is useful in meta-analyses synthesizing all available evidence. We recommend an integrated approach to bias adjustment, informed by both available generic evidence and elicited opinion. Choice of integrated approach may be based on the preferences of the systematic review authors.

### P229

#### Placebo response is not decreased by enrichment trial designs in randomized controlled trials of triptan medications in the paediatric age group

Lawrence Richer, Ben Vandermeer, Lisa Hartling
University of Alberta
**Correspondence:** Lawrence Richer
*Trials* 2017, **18(Suppl 1):**P229

This abstract is not included here as it has already been published.

### P230

#### Influence of primary outcome change on treatment effect estimates in clinical trials: meta-epidemiological study

Tao Chen[1], Rui Qin[2], Duolao Wang[1], Victoria Cornelius[3]
[1]Tropic Clinical Trial Unit; [2]Department of Health Education; [3]Imperial Clinical Trials Unit
**Correspondence:** Tao Chen
*Trials* 2017, **18(Suppl 1):**P230

**Background**
Online registration of trial protocols has been implemented to support transparency and good clinical practice for the conduct of a trial. One aspect is to ensure that the primary outcome is pre-specified prior to any data being collected and interim analysis performed. This is to discourage the outcomes being selectively chosen for reporting based on significant p-values. We aimed to examine the status of randomised clinical trials (RCT) whose primary outcome changed between protocol registration and published paper, and to quantify the impact of this change on the resulting treatment effect estimates.
**Method**
We searched registered RCT from Medline and EMBASE between 2011 and 2015 and randomly selected 5% of searched articles for each year. Articles were excluded if they are not RCT or trials with multiple primary outcomes. For each included trial, we collected information on the primary outcome reported in the article and in the registered protocol. Trials were classified as having a changed primary outcome if there was an inconsistency between the registered and published outcome. Additional information on effect size, type of outcome, type of study design, post-randomisation exclusions were extracted. For consistency, we inverted the effect estimates where necessary so that each trial indicated an odds ratio less than 1 (where the active group has more favourable result than the control group). The relative odds ratio (that is, the summary odds ratio for trials with a primary outcome change divided by those without) was calculated and a value less than 1 indicated larger treatment effects in trials with changed primary outcome compared to trials whose primary outcome was the same between the protocol and final publication.
**Results**
Among 29,749 searched articles (Medline: 28,810, EMBASE: 939), 1,488 articles were selected in this study. Of the 487 eligible trials, 63 (12.9%) published articles were reported with no or an unclear description of primary outcome. 21(4.3%) studies were registered with no or an unclear description of primary outcome. 75 (15.4%) trials were registered after the completion of the study. Among the remaining trials with primary outcome clearly registered and reported, 29.0% (95/328) showed some discrepancies in primary outcome between trial registration and published article. Further excluding 33 trials due to uncalculated data, there were 295 trials that could be included in the bias assessment and we found a clearly larger intervention effect (pooled ratio of odds ratios 0.79 (95% confidence interval 0.68 to 0.91), $p = 0.0012$) among trials with changed primary outcome compared to trials whose primary outcome was the same. The results were consistent after adjustments for type of outcome, type of study design, post-randomisation exclusions, and variance of log odds ratio (0.79 (0.69 to 0.92), p = 0.0019).

**Conclusion**

Trials that deviated from the originally registered outcome showed larger intervention effects than trials whose primary outcome was unaltered from the original protocol registration. This highlights the important role of trial registration prior to the initiation of trial and the need for clear specification of the primary outcome.

### P231

#### Reducing under- and over-triage in motor vehicle crashes using an injury-based approach

Jennifer Talton[1], Ashley A. Weaver[2], Ryan T. Barnard[2], Samantha L. Schoell[2], Joel D. Stitzel[2]
[1]Wake Forest School of Medicine; [2]Virginia Tech-Wake Forest University Center for Injury Biomechanics
**Correspondence:** Jennifer Talton
*Trials* 2017, **18(Suppl 1):**P231

Advanced Automatic Crash Notification (AACN) systems aim to reduce both over- and under-triage from motor vehicle crashes (MVC) by using vehicle telemetry data to predict risk of serious injury and thus aiding first responders in the triage decision making process. Reducing under-triage (UT) translates into transporting severely injured occupants to a level-I or II trauma center (TC) and reducing over-triage (OT) means transporting occupants with lesser injuries to a non-trauma center (non-TC). Treating more severely injured occupants initially at tcs reduces death and disability, and treating occupants with minor injuries at non-tcs leads to better hospital resource utilization and decreased healthcare costs.
In order to estimate the need for transport to a TC or non-TC, current AACN systems model the risk of severely injured occupants using injury severity scores (ISS) as the outcome. ISS are a well-known measure based on the Abbreviated Injury Scale (AIS) coding lexicon where occupants with ISS > = 16 indicate severe injuries requiring treatment at a TC, while ISS < 16 may be treated at a non-TC. Our group has developed an AACN algorithm, the Occupant Transportation Decision Algorithm (OTDA), using an injury-based approach rather than AIS severity alone. We have identified three facets of injury that contribute to need for treatment at a TC: severity, time sensitivity and predictability. Severity is a measure of an injury's mortality, time sensitivity quantifies its urgency and predictability is its likelihood of being missed upon evaluation by first responders at the scene. These three components are then jointly optimized to create a list of 240 injuries, each with a yes/no indicator of being on the Target Injury List (TIL). We believe that the TIL gives a better picture of the extent of injury severity and need of treatment at a TC or non-TC.
The OTDA was implemented using data from National Automotive Sampling System-Crashworthiness Data System 2000–2011, which included 38,970 cases. The OTDA uses multivariable logistic regression to predict the risk of an occupant sustaining an injury on the TIL for specified crash conditions. In addition to using an injury-based approach for modeling the risk of severely injury occupants, another novel feature of the OTDA compared to other AACN systems is that the OTDA uses a genetic algorithm to optimize each of the components and determines the risk threshold for the decision to transport to a TC or non-TC. The goal of the optimization was to minimize UT and OT, ideally producing UT rates < 5% and OT rates < 50% as recommended by the American College of Surgeons (ACS).
Results of the OTDA produced UT rates ranging from 3-16% depending on the crash mode and OT rates meeting the ACS 50% recommendation. The OTDA also showed improved UT rates compared to other AACN algorithms in literature. We believe the OTDA will aid emergency personnel to make the correct triage decision for an occupant after a MVC. With nation-wide implementation, we estimate a potential benefit of improved triage decision-making for 165,000 occupants annually.

### P232

#### Challenges in implementing and managing clinical trials in developing countries: lessons learned by the national cancer institute's (NCI) AIDS malignancy consortium (AMC)

Megan Wirth, Dikla Blumberg, Kimberly Mosby-Griffin, Don Vena
Emmes Corporation
**Correspondence:** Megan Wirth
*Trials* 2017, **18(Suppl 1):**P232

The AIDS Malignancy Consortium (AMC) is a National Cancer Institute supported multicenter clinical trials group founded in 1995 to support innovative trials for AIDS-related cancers. In 2010 the AMC expanded operations internationally and opened 4 sites located in sub-Saharan African countries with a high prevalence of HIV. The goal of this expansion was to build a cancer clinical trials network in sub-Saharan Africa (SSA) that was capable of conducting contextually appropriate therapeutic and prevention trials in a variety of HIV-associated cancers and contributing to the AMC'S scientific agenda.
The AMC Operations and Data Management Center (AMC ODMC) provides data management and site management support for both domestic and international AMC trials. Over the past 7 years, the AMC ODMC has supported 3 trials in SSA and identified a number challenges to trial implementation and activation.
The key challenges the AMC ODMC faced in implementing these trials included identifying research priorities, developing multicenter trials that are appropriate across a diverse group of trials sites, conducting clinical research trials within the public healthcare system, inadequate infrastructure, availability of qualified staff, and identifying and addressing site logistical barriers such as drug and supply needs. Furthermore, the importance of supporting capacity-building activities such as training of health care staff at the research sites is part of the AMC'S mandate in SSA and requires additional site management support. Currently, there are 2 open trials and 4 trials in expected to open within the next 18 months across 7 sites in Sub-Saharan Africa, including sites in Zimbabwe, Uganda, Kenya, Malawi, Tanzania and South Africa. Site management lessons learned from these trials may be applicable to other international trials and particularly relevant to those designed for implementation in developing countries where both human and material resources may be limited.

### P233

#### Efficiencies in multi-centre RCTs - What lessons can be learned from a trial with more than 100 recruitment sites?

Seonaidh Cotton, Karen Innes, Joanna Kaniewska, Mark Forrest, Graham Devereux
University of Aberdeen
**Correspondence:** Seonaidh Cotton
*Trials* 2017, **18(Suppl 1):**P233

**Background**

We have recently completed recruitment to a large multicentre trial with recruitment sites in both primary and secondary care. Opening a recruitment site is a substantial amount of work: it requires approvals to be in place; a site agreement to be signed by all parties; site initiation/training; copies of CVs, GCP certificates and a completed delegation log to be returned to the study office; for the site to have received a site file and study documentation; and in this study, an estimate from the site of number eligible patients to be invited.
As in previous studies, spreadsheets were maintained to log information about contacts, documents returned, CVs and GCP certificates, such that a green-light form could be populated for sign off prior to opening the site. However, given the number of recruitment sites involved, this logging of information was very time-consuming. We reviewed our processes, and, where possible, have implemented alternative processes that are likely to generate efficiencies in recruitment site set-up. These are described below.

**Sites opened**

In total, 175 recruitment sites were identified (36 secondary care sites and 139 primary care sites). 141 sites were opened to recruitment

(36 secondary care sites and 105 primary care sites). Of the 36 secondary care sites opened to recruitment, 31 recruited and 5 did not. Of the 105 primary care sites opened to recruitment, 86 recruited participants and 19 did not.

There were a number of reasons why sites did not open to recruitment. The most common reason was that the site did not return documentation to the study office (CVs, GCPs certificates, delegation log, site agreement). A few sites actively withdrew from the study before being green-lighted due to staff changeover or perceived lack of eligible patients.

Once opened, some sites failed to recruit any patients to the study. Reasons for this included staff changeover and lack of eligible patients, competing priorities and eligible patients who did not agree to take part.

**Lessons learned**

We identified potential for efficiencies in terms of logging information about sites and staff. Minimal information was already logged onto the study website (to register a site to enable randomisation and collection of study data and to maintain appropriate website access for site staff). For future studies, the website template has been amended to include: (i) an additional web form to log information about the site, including approvals in place, progress of site agreement, etc.; and (ii) a web form to log information about site staff, including CVs received, date of GCP training, along with the facility to upload CVs/GCP certificates onto the website. In addition, the website template facilitates the upload and storage of local documents.

Having such systems in place is likely to generate trial efficiencies: be time-saving for trial office staff, recording information in a single place and allow the green-light forms to be generated automatically; have capacity to run regular reports (for example progress reports on site set-up); and generate notifications for, for example renewal of GCP training.

### P234

#### Why do trials get suspended? A review of data from clinicaltrials.gov and ISRCTN

Seonaidh Cotton, Chloe Brooks, Lynda Constable
University of Aberdeen
**Correspondence:** Seonaidh Cotton
*Trials* 2017, **18(Suppl 1):**P234

**Background**

Within our trials unit two trials have recently been suspended, and then restarted. While there is a fairly mature literature on early termination of studies, there is a paucity of literature about the temporary suspension of studies.

We aimed to document reasons for trial suspensions using data available on publically available registers of clinical trials (clinicaltrials.gov and ISRCTN).

**Methods**

Clinicaltrials.gov define a 'suspended' study as a 'study that has stopped recruiting or enrolling participants early, but may start again'. We searched clinicaltrials.gov for interventional studies which had their recruitment status recorded as 'suspended': the search was run on 29 June 2016. ISRCTN do not have an equivalent term for suspended trials. The closest term is 'stopped' which includes studies that have never started along with those that have stopped prematurely. We searched ISRCTN for interventional studies which had their status recorded as 'stopped': the search was run between 18 July and 12 August 2016.

For each suspended trial, a code was assigned to each trial to classify the reason for the suspension. The coding framework was developed inductively and continually refined during the process of coding.

**Results**

837 trials registered on clinicaltrials.gov had their recruitment status designated as suspended. 403 trials registered on ISRCTN were recorded as 'stopped'. It was not possible to identify the reason for suspension/stopping for 40% of those recorded as suspended on clinicaltrials.gov and 8% of those recorded as stopped on ISRCTN: either no reason was given, or the reason was not clear or ambiguous.

The review of reasons for suspension identified five main themes: drug/intervention issues (drug/intervention safety issues, drug/intervention supply issues); trial evaluation (futility, review of trial/changes to protocol); funding (funding issues); recruitment (primarily slow accrual, but some cases of rapid accrual); and running of the trial (operational issues, staffing, wider organisational issues). The proportions of trials suspended/stopped for each of these reasons differed between the two trial registers. 19% of those suspended on clinicaltrials.gov had been suspended because of drug/intervention safety issues compared to 6% of ISRCTN. The proportions suspended/stopped for the other reasons are: trial evaluation 29% in clinicaltrials.gov vs 18% in ISRCTN; funding 17% vs 31%; recruitment (20% vs 36%), running of the trial (15% vs 9%).

**Discussion**

The observation that there are differences in the relative importance of reasons why trials are suspended/stopped may reflect the types of trials registered on the two registries.

A number of those registered as 'suspended' on clinicaltrials.gov appeared to have been terminated early (with no intention of restarting) or completed rather than suspended. More guidance for those maintaining records on trial registries may aid the consistency of recording.

### P235

#### A reflection on the management of a trial of speech and language therapy

Caroline Rick[1], Carl E. Clarke[1], Natalie Ives[1], Smitaa Patel[1], Rebecca Woolley[1], Francis Dowling[2], Lauren Genders[1], Christina H. Smith[3], Marian C. Brady[4], Keith Wheatley[1]
[1]University of Birmingham; [2]Cambridge University; [3]University College, London; [4]Glasgow Caledonian University
**Correspondence: Caroline** Rick
*Trials* 2017, **18(Suppl 1):**P235

**Background**

Randomised controlled trials (RCTs) of therapy interventions are becoming increasingly common, and provide a series of challenges. Here we discuss our experience from the PD COMM Pilot (A Pilot Randomised Controlled Trial Of Lee Silverman Voice Treatment Versus Standard NHS Speech And Language Therapy Versus Control In Parkinson's Disease) trial. Problems with speech or voice are common with people with Parkinson's (PWP). Miller (2006) noted how changes in communication led to increased physical and mental demands during conversation, an increased reliance on family members and/or carers, an increased likelihood of reduced participation and social withdrawal. Two types of speech and language (SLT) therapy are available to PWP: NHS SLT an individually tailored intervention of ~6-8 sessions per local practice and Lee Silverman Voice Treatment (LSVT) a structured set of 16 sessions over 4 weeks focussed on volume. There is little evidence that either is effective. PD COMM Pilot examined the feasibility of a full scale trial, and to optimise the design. Intervention-based issues and solutions are discussed below.

**Recruitment**

as the intervention was dependent on speech and language therapists (salts) being available to start therapy within 4–6 weeks, bottlenecks occurred e.g. School holidays, staff turnover. Good communication between the research nurses and salts was vital and sites were allowed to pause recruitment if salts were unable to start therapy within the trial timelines. While this slowed recruitment, 95% of 59 participants had received therapy by the 3 month primary endpoint. Staffing: There were a number of potential issues 1. Did the level of experience of staff treating participants in the NHS and LSVT arms differ? 2. Does the beliefs of the salts? Regarding patient suitability for interventions or treatment preference impact the results? 3. Limited research experience of many salts. 17 therapists only saw 1 participant, 11 saw only participants in 1 arm and 8 saw participants in both. The trial provided a supportive network for salts to exchange information. These potential issues will be examined in more detail in the substantive trial where an in-depth process evaluation will also be performed.

#### Intervention

Does the content of the interventions change over time? The trial kept treatment records and has explored the dose and content. The numbers of participants randomised to each treatment arm at individual sites were too small to test for changes over time, however this will be examined in the substantive trial. Logistic issues: Frequently the Trust providing the intervention was different to the recruiting site. This produced a number of issues e.g. Recognition including SFT funding is associated with recruitment not the treatment site. Further, the catchment areas of the Trusts may only partially overlap. In some cases, the only resolution was for sites to only recruit from a subset of potential participants dependent on treatment sites' catchment area. Communication, support and recognition of different perspectives and priorities has built a research group that will form the basis of the substantive trial: 10 of the 11 pilot sites were happy to participate in the substantive trial.

### P236

### Risk based monitoring (RBM) tools for clinical trials: a systematic review

Caroline Hurley[1], Frances Shiely[2], Patricia Kearney[2], Mike Clarke[3], Joseph Eustace[4], Evelyn Flanagan[4], Jessica Power[3]
[1]University College Cork, Ireland; [2]Department of Epidemiology and Public Health; [3]Centre for Public Health; [4]Health Research Board -Clinical Research Facility
**Correspondence:** Caroline Hurley
*Trials* 2017, **18(Suppl 1):**P236

#### Background

In November 2016, the Integrated Addendum to ICH-GCP E6 (R2) will advise trial sponsors to develop a risk-based approach to clinical trial monitoring. This new process is commonly known as risk based monitoring (RBM). To date, a variety of tools have been developed to guide RBM. However, a gold standard approach does not exist. This review aims to identify and examine RBM tools.

#### Methods

Review of published and grey literature using a detailed search-strategy and cross-checking of reference lists. This review included academic and commercial instruments that met the Organisation for Economic Co-operation and Development (OECD) classification of RBM tools.

#### Results

Ninety-one potential RBM tools were identified and 24 were eligible for inclusion. These tools were published between 2000 and 2015. Eight tools were paper based or electronic questionnaires and 16 operated as Service as a System (SAAS). Risk associated with the investigational medicinal product (IMP), phase of the clinical trial and study population were examined by all tools and suitable mitigation guidance through on-site and centralised monitoring was provided.

#### Conclusion

RBM tools for clinical trials are a relatively new, their features and use varies widely and they continue to evolve. This makes it difficult to identify the "best" RBM technique or tool. For example, equivalence testing is required to determine if RBM strategies directed by paper based and SAAS based RBM tools are comparable. Such research could be embedded within multi-centre clinical trials and conducted as a SWAT (Study within a Trial).

### P237

### Tools and processes for tracking IRB approvals as a dcc for large multi-center clinical research networks

Jenna Gabrio, Jeanette O. Auman, Lindsay M. Parlberg, Margaret M. Crawford, Kristin Zaterka-Baxter
RTI International
**Correspondence:** Jenna Gabrio
*Trials* 2017, **18(Suppl 1):**P237

#### Objective

A primary responsibility of Data Coordinating Centers (DCC) for multi-center research networks is tracking individual center IRB approvals. In networks with high numbers of studies and clinical centers (CCs) the amount of documentation can be overwhelming and burdensome to manage. A team of DCC programmers and coordinators developed simple electronic tools and processes to fulfill this responsibility.

#### Background

Since tracking approvals is intended to protect participant data and ensure data was obtained and released to the DCC in an ethical manner consistent with regulatory oversight, we identified ways to innovate and simplify processes. Historically, tracking has been done on paper involving complex filing systems; however, technological advancements expanded options for executing this responsibility.

#### Methods

Programmers developed an in-house Microsoft Access database used to track receipt of IRB approvals for numerous studies from multiple ccs. The custom database has capacity to monitor approval expiration dates for an unlimited number of studies and ccs, and it can generate automated reports displaying information on all documented approvals. The system has functionality to produce reports organized by individual protocol and/or CC, as well as the ability to highlight IRB approvals that must be renewed within the next three months. Coordinators formalized communication procedures for collecting updated approvals and informing ccs of the status of information currently on file. We established a central email account to which ccs submit updated documentation. Upon receipt of documentation a DCC coordinator acknowledges delivery, files documentation, and enters updated information into the database. Monthly, a DCC coordinator generates automated individual center IRB reports and posts them to the research network private website. Ccs receive an email notification from the DCC and can then access their center reports through the website. Based on these reports ccs determine what documentation must be sent to the DCC to keep their records up to date.

#### Results

The development and implementation of a database increased efficiencies both for ccs and DCC s. The processes reduced the volume of email regarding IRB approvals sent to ccs. Individual emails to ccs notifying them an approval is about to expire are no longer necessary. Instead, a single monthly email is sent to all ccs indicating updated IRB reports have been posted and should be reviewed. Automated highlighting of approvals that will expire soon has also reduced the burden on the DCC coordinators and minimized the likelihood of oversight. The creation of a central database and formalized procedures have streamlined internal regulatory processes for DCC staff. If questions arise about an approval for a specific CC, DCC staff can access the database to look up information needed.

#### Conclusions

Although an initial investment is needed to design a database, development and formalization of these processes have resulted in significant time and cost savings throughout the organization's tenure as a DCC. The flexible nature of an Access database makes it an efficient and suitable solution for tracking a growing number of studies in research networks that may have a fluid composition of centers over time.

### P238

#### The role and impact of patient and public involvement and engagement (PPIE) in clinical research: perspectives from Keele CTU project management

Helen Myers[1], Sarah A. Lawton[1], Stefannie Garvin[1], Steven Blackburn[2]
[1]Keele Clinical Trials Unit; [2]Institute for Primary Care and Health Sciences
**Correspondence:** Helen Myers
*Trials* 2017, **18(Suppl 1):**P238

**Background**
Keele Clinical Trials Unit (CTU), based within the Faculty of Medicine and Health Sciences at Keele University, is a UK Clinical Research Collaboration registered CTU specialising in the development and delivery of large multicentre clinical trials testing treatments and health services, as well as conducting large epidemiological studies in primary and secondary care settings. Keele CTU supports the design, delivery and analysis of research studies. Keele CTU works closely with the Patient and Public Involvement and Engagement (PPIE) Team located within the Institute for Primary Care and Health Sciences at Keele University. The PPIE Team have a Research User Group (RUG) which consists of people with experience of, or carers of close relatives with, long-term conditions. The CTU Project Management team are pivotal in ensuring the success of research studies and work closely with the RUG to achieve this.
This abstract provides examples of the role and impact of PPIE in the conduct of research and presents perspectives from the Project Management team on PPIE contribution to the delivery of research studies.
**Methods**
The RUG plays an essential role in each stage of research design and delivery, helping to ensure that the research is ethical and acceptable to research participants. We asked the Project Management team for examples of the ways in which involvement of the RUG had benefitted the studies they managed, and for their perceptions of the impact the RUG had on research. Responses were collated and organised thematically to provide a description of PPIE contribution and its impact.
**Results**
The RUG are involved in a variety of activities including assisting with grant application, intervention development, document design, ethical approval, development of recruitment and retention strategies, patient simulation, quality assurance, study monitoring and dissemination of findings. To highlight the contribution and impact of the RUG two specific examples are presented in this abstract. The RUG played an active role in developing a 'usual care' leaflet for a trial of an intervention for hand osteoarthritis. Their contribution made the leaflet clear, practical and acceptable to patients. The RUG provided valuable ideas about how to approach patients in a GP waiting room to enrol them into a study which involved video-recording a GP consultation. They made realistic and patient-centred suggestions for how this could be achieved ethically.
The overall impact of the RUG involvement is captured in the following quotes from the Project Management team: "Valuable team members", "Enhance research relevance", "Unique contributions and viewpoints", "Patient perspectives", "Essential role".
**Conclusions**
Well managed, high quality research can provide evidence for best practice in diagnosis, treatment, management and prognosis to improve outcomes for patients. RUG involvement in research design and delivery forms an integral role in the pathway which provides the best evidence for both funders and clinicians, and contributes to the best care for patients. The Project Management team greatly value the views, opinions and suggestions made by the RUG. The personal experiences of those living with, or supporting those with, the research condition of interest, strengthens study design and greatly enhances the research relevance for the public.

### P239

#### A good use of time and money? A study of how trial data collection effort is distributed across different categories of data

Gordon Fernie[1], Katie Banister[1], Suzanne Breeman[1], Lynda Constable[1], Anne Duncan[1], Heidi Gardner[1], Kirsteen Goodman[2], Doris Lanz[3], Alison McDonald[1], Emma Ogburn[4]
[1]University of Aberdeen; [2]Glasgow Caledonian University; [3]Queen Mary University Of London; [4]University of Oxford
**Correspondence:** Gordon Fernie
*Trials* 2017, **18(Suppl 1):**P239

**Background**
Data collection consumes a substantial portion of the resources used in any randomised trial. In addition to the participant identifier, the most important data collected is the primary outcome; it drives the sample size calculation and should be the main focus of the research effort. Most trials also collect secondary outcomes to supplement the primary. These additional data are often collected to monitor safety, maintain quality and ensure regulatory and data management requirements are fulfilled. Many trials also collect outcome data not listed in the trial protocol.
Adding ancillary and exploratory data collection can result in a substantial portion of a trial's limited resources, in time, money and participant burden, being devoted to collecting data that are not directly linked to answering the research question. The cost of this is not trivial: a large US study of drug trials estimated that non-core data collection costs $3.7 billion annually. As part of the Trial Forge initiative (a systematic approach to making trials more efficient) here we describe our categorisation of the distribution of data collection effort in a range of trials.
**Methods**
We have developed a list of 16 data categories (e.g. Participant identification, eligibility, demographics, health economics and safety data), along with guidance on what each category might contain. A standard operating procedure describes how to go through a trial's data collection forms to categorise each collected data item. Data categorisation is done independently in pairs, one person having in-depth knowledge of the trial, the other independent of the trial. Any disagreement is resolved through discussion, with the rest of the project team being brought in if necessary. Current work has focused on piloting our materials and method with three trials run from three different UK Trials Units. We will extend this work to further trials run from more Trials Units prior to the SCT/ICTMC conference.
**Results**
Our preliminary results suggest that trial teams spend more time collecting data than they do collecting outcomes: sometimes less than 50% of the data collected is linked to primary and secondary outcomes. The largest single category is almost always secondary outcomes, which range between 28% and 52% in the three trials categorised to date. Primary outcome data ranges from 1.2% to 14.2%. Safety and regulatory data accounted for between 1.1% and 13%. In one of the three trials 2,530 data items were collected, 78.8% of which were mandatory.
**Conclusions**
Our early results suggest that a substantial proportion of trial data is not outcome data. Primary outcomes accounted for less than 15% of all data collected; secondary outcomes were at least 3 times as many but in two trials represented over 20 times as much. Should the remaining trials in our study follow this pattern then, given the expense of collecting, storing and cleaning data, it suggests trialists should have an increased awareness of the burden and costs associated with adding data items to data collection forms. Regulators and others should bear in mind the burden their requirements may place on trial teams.

### P240

#### Staff experiences of closing out a clinical trial involving withdrawal of treatment: qualitative study

David White[1], Julia Lawton[2], David Rankin[2], Jackie Elliott[1], Carolin Taylor[3], Cindy Cooper[1], Simon Heller[1], Nina Hallowell[4]
[1]University of Sheffield; [2]University of Edinburgh; [3]Sheffield Teaching Hospitals NHS Foundation Trust; [4]University of Oxford
**Correspondence:** David White
*Trials* 2017, **18(Suppl 1):**P240

**Background**
The ending of a clinical trial may be challenging, particularly if staff are required to withdraw the investigated treatment(s); however, this aspect of trial work is surprisingly under-researched. To address this gap, we explored the experiences of staff involved in closing out a trial which entailed withdrawal of treatment (insulin pumps) from some patients.
**Methods**
Interviews were conducted with $n = 22$ staff, recruited from seven trial sites. Data were analysed thematically.
**Results**
Staff described a number of ethical and emotional challenges at close-out, many of which had been unforeseen when the trial began. A key challenge for staff was that, while patients gave their agreement to participate on the understanding that pump treatment could be withdrawn, they often found themselves benefiting from this regimen in ways they could not have foreseen. Hence, as the trial progressed, patients became increasingly anxious about withdrawal of treatment. This situation forced staff to consider whether the consent patients had given at the outset remained valid; it also presented them with a dilemma at close-out because many of those who had wanted to remain on a pump did not meet the clinical criteria required for post-trial funding. When deciding whether to withdraw treatment, staff not only had to take funding pressures and patient distress into account, they also found themselves caught between an ethic of Hippocratic individualism and one of utilitarianism. These conflicting pressures and ethical considerations resulted in staff decision-making varying across the sites, an issue which some described as a further source of ethical unease. Staff concluded that, had there been more advanced planning and discussion, and greater accountability to an ethics committee, some of the challenges they had confronted at closeout could have been lessened or even prevented.
**Conclusions**
The same kinds of ethical issues which may vex staff at the beginning of a trial (e.g. Patients having unrealistic expectations of trial participation; staff experiencing conflicts between research and clinical roles) may re-present themselves at the end. To safeguard the wellbeing of staff and patients, greater planning, coordination and ethical oversight should go into the close-out of trials involving withdrawal of treatment(s).

### P241

#### Learning from the OCTET trial - exploring acceptability of clinical trials management

Catherine Arundel[1], Judith L. Gellatly[2]
[1]University of York; [2]University of Manchester
**Correspondence:** Catherine Arundel
*Trials* 2017, **18(Suppl 1):**P241

**Background**
Conducting research can be a time consuming, difficult and challenging process. Guidance and pragmatic advice focusing on randomised controlled trial conduct are available but do not necessarily constitute comprehensive guidance. Standardised trial management tools, have previously outlined key elements constituting a successful trial as a method of ensuring good practice in research trials: initiation, planning, execution, monitoring, and analysis. Despite existing tools and guidance, lessons are also frequently learnt during the development and conduct of trials however rarely are these experiences shared for the benefit of others. For the wider research team, the key focus will always be on the execution and delivery of a study. We therefore evaluated the acceptability of clinical trials management, focusing on study execution and monitoring, as implemented in the NIHR HTA funded Obsessive Compulsive Treatment Efficacy Trial (OCTET).
**Context**
OCTET was a randomised controlled trial investigating the effectiveness of low intensity interventions for the treatment of obsessive-compulsive disorder (OCD). Two trial managers coordinated the study. This included managing and coordinating personnel working across a variety of roles within the study - research assistants, clinical practitioners, site leads, and independent committee members.
**Methods**
Workshops, questionnaires and semi-structured interviews were used to explore acceptability of trials management methods with a specific focus on the execution and monitoring of the study. Members of the OCTET Trial research team were asked to comment, both positively and negatively, on their experience of the management, procedures, training, and their overall involvement in the trial. 9 members participated in the workshop, 10 completed a questionnaire and 20 were interviewed as part of qualitative work for the main OCTET study. Data was collected and analysed using thematic analysis, with the key phases of this approach adhered to.
**Results**
Six key themes associated with study execution and monitoring were identified within the data: support; communication; processes; resources; training and ethos. Clear and open communication and enthusiasm and accessibility of the trial managers and Chief Investigator were noted across all themes as an important facet of the successful running of the trial. Clear resources and training materials were also found to be crucial in helping staff to work within the trial setting however constructive suggestions were also made for improvement.
**Conclusion**
Organisation, openness, and positivity are crucial for executing a trial successfully, whilst clear and focused processes and resources are essential in monitoring and controlling the progress of a trial. Trial managers should therefore consider developing these elements when setting up a study. There is however, always room for improvement and the continued sharing of effective techniques will help to further evolve efficient trial management.

### P242

#### Recruitment estimates provided by sites - are they consistent with observed accrual?

Christy Toms, Alexa Gillman, Clare Cruickshank, Shama Hassan, Emma Hall, Claire Snowdon, Judith Bliss, Rebecca Lewis
The Institute of Cancer Research Clinical Trials & Statistics Unit (ICR-CTSU)
**Correspondence:** Christy Toms
*Trials* 2017, **18(Suppl 1):**P242

**Background**
Integral to the development of new trial proposals and a key consideration for funders is the assessment of feasibility of recruiting the planned sample size. Estimated accrual figures provided by sites are usually based on local clinical experience of the relevant patient population, data from internal audits of number of patients seen and previous experience of recruitment to trials. These estimates are used during trial development to inform trial design and plan recruitment timelines and as such have a substantial impact on the funding support requested. We aimed to assess how close actual recruitment totals were to the estimates provided at the funding application stage to determine if evidence-based correction factors could be defined.
**Methods**
Six oncology trials covering a range of disease sites and treatment modalities were selected from the ICR-CTSU portfolio. Individual sites' estimated annual recruitment was compared with the average annual accrual observed. The proportion of sites which failed to open following initial expression of interest at funding application stage and number of sites which opened which were not included in the original funding application were also reviewed.

#### Results

One hundred & twenty two sites were on the funding applications of the six trials reviewed, representing 82 centres in total, some listed for >1 trial. Sites estimated they would recruit a total of 446 patients per annum. Of those which opened, only 7/77 (9%) exceeded their recruitment estimates. 8/77 sites (10%) recruited 0-40% less than predicted, 28/77 sites (36%) 40-80% less and 31 sites (40%) 80-100% less. Three sites did not provide recruitment estimates. Median percentage reduction between predicted and actual recruitment per site was 74% (inter quartile range 91 to 44). 45/122 of sites did not proceed to open the respective trial (37%). Of the sites which participated in each trial, 48% were not originally included on the funding applications. These sites contributed an average of 17% of target accrual of the six trials. Over all trials, average observed annual accrual was 66% of that estimated by sites.

#### Conclusion

Potential participating sites substantially overestimate accrual at the funding application stage. This has consequences for trial development as it impacts assessments of trial feasibility and planned recruitment period. Sites which express interest and then fail to open can also skew the recruitment estimates; however this appears to be mitigated to a certain extent by those sites which do not provide expressions of interest at funding application stage but proceed to open the trial at a later date. Estimating projected trial accrual is challenging for sites and trials units. Options for improving recruitment estimations, including use of national electronic health records and documenting provenance of recruitment estimates (e.g. Local audits), should be considered. Our data suggests feasibility of accrual should be routinely reassessed per site once funding approval is confirmed.

### P243

### Using electronic healthcare records (I) to screen, locate and recruit participants from primary care

Maimoona Hashmi[1], Kirin Sultana[1], Elizabeth Moore[2], Jennifer Quint[2], Mark Wright[1]
[1]Clinical Practice Research Datalink (Centre of the MHRA); [2]Imperial College London
**Correspondence:** Maimoona Hashmi
*Trials* 2017, **18(Suppl 1):**P243

#### Background

Conduct of clinical studies traditionally involves study teams approaching clinicians to screen and find potential study participants. This can be both time-consuming and labour intensive for clinicians and researchers. Leveraging Electronic Healthcare Records (I) as a resource to locate and screen eligible study participants is often underutilised but significantly reduces pre-screening activities. An example of the advantage of this method is illustrated in the following study investigating the association between air pollution and COPD exacerbations using portable air monitors and symptom diaries. Using I data within the Clinical Practice Research Datalink (CPRD), we screened for eligible patients in primary care practices, based on the protocol inclusion/exclusion criteria. CPRD data are comprised of continually provided anonymised UK electronic primary care records to enable clinical studies into improving public health.

#### Methods

The CPRD I database was interrogated to create a pre-screened list of patients located in practices close to the research sites in central London. The search engine used a study-specific validated codelist and algorithm. Using diagnostic codes alone, this algorithm had a Positive Predictive Value (PPV) of 86.5% which is improved slightly by including use of antibiotics and oral corticosteroids in previous 12 months and spirometry; spirometry was not used as a screening criterion within the I but was subsequently performed at the research site. Patients were excluded if they were either current smokers or aged < 35 years old. Participating GPs were provided with a pre-screened list from which to identify and select suitable patients to receive information about the study. Potential participants could contact the research team directly to be enrolled.

#### Results

Eighty-one practices were approached of which 24 (29%) consented to participate, resulting in a pre-screened list of 904 patients. There were 314 screen-failures (35%) of which 55% were "unable/unsuitable" to participate in the study for reasons such as housebound, dementia and other co-morbidities; a further 29% of screen-failures were excluded for reasons associated with COPD diagnoses and exacerbations, lastly, 16% were either transferred out or deceased. 590 patients were invited of which 209 responded: 141/209 (67%) declined to participate and 61/209 (29%) agreed to participate. The main reasons for declining were: study too demanding (43%); not interested (14%); currently facing health issues (15%).

#### Conclusion

The use of CPRD data enabled site recruitment efforts to be concentrated on those practices with eligible patients close to the research site locations. The provision of CRPD data to pre-screen for patients meeting the study eligibility criteria reduced the amount of work required from GPs. There were however a significant number of screen-failures detected by GPs that were not covered by the search criteria, suggesting further improvements can be made to the search criteria to make this process more efficient. Through CPRD data we were able to successfully screen and recruit patients with COPD from GP practices within central London to participate in research over a 6 month period. This is an effective and novel method of using EHRs to screen and recruit participants for research.

### P244

### Clinical trial data and tissue: considerations for responsible sharing

Claire Snowdon, Emma Hall, Judith Bliss
The Institute of Cancer Research
**Correspondence:** Claire Snowdon
*Trials* 2017, **18(Suppl 1):**P244

Clinical Trials Units (CTUs) oversee a large resource of data and linked samples generated from clinical trials and have a duty to facilitate responsible sharing of these collections with the wider research community. Sharing has the potential to improve scientific and medical knowledge, improve and validate research methods, encourage collaboration and reduce duplication of effort. Sharing must take into consideration the scientific integrity of the original trial and the proposed research, the terms of the consent with which tissue and data were collected, relevant governance and regulatory requirements and the terms and conditions of the sponsors and funders of the original trial.

Clinical trials are conducted to provide a precise unbiased estimate of effect to inform the next trial and influence clinical practice. It's imperative that the integrity of the trial is maintained until the primary research questions have been answered. When considering a request for access to a specific trial cohort, CTUs consider: the clinical importance of the hypothesis; whether the hypothesis requires access to the specific trial collection; whether the relevant data or tissue are held and are of sufficient quality and quantity; the statistical validity of the proposed research; whether the proposed research is validation or discovery; whether sharing would compromise the collection or reporting of the original trial; and whether there are opportunities for collaboration.

There is a wealth of legislation and supporting codes of practice concerning the appropriate use of tissue and data collected from clinical trial participants. At the heart of this is the need to ensure appropriate informed consent and to protect participant confidentiality. The use of data and samples is limited by the scope of the consent and conditions of approval under they were originally collected. Data and tissue may be used without explicit consent if it is fully anonymised and the research has been approved by a research ethics committee. However fully anonymising data may be difficult to achieve and may even reduce the utility of the collection for the intended purpose. Ethics committees now accept the need to seek broad and enduring consent for future use of data and tissue. However advances in technologies and changes in societal expectations can make this difficult to achieve.

Tissue and data collected within clinical trials are of a high quality. They are collected mostly prospectively in a systematic and unbiased fashion and are well curated and documented. They are a precious resource and represent a considerable investment from those involved in the original trial including the clinical trial participants, investigators, CTU, trial oversight committees, funders and the sponsor. Their opinions, terms and conditions must be taken into account when considering proposals for data sharing. This can be managed through formal access policies, processes and agreements.
There is a balancing act between data sharing on one hand and protection of the collection and those who contributed to the collection on the other. However sharing can and should be achieved ethically, legally and with scientific probity with appropriate considerations and controls.

### P245

#### Clinical trials: increasing value, reducing waste – the potential role of ethics committees

Joerg Hasford
University of Munich
*Trials* 2017, **18(Suppl 1):**P245

**Background**
Macleod et al. Criticized in 2014 that there is too much avoidable waste and too little value in biomedical research and identified several relevant issues (Lancet 2014:383:101–104).
**Objective**
As in the European Union all clinical trials have to be reviewed and approved by a competent Ethics Committee prior to the start, it is time to check whether Ethics Committees can play a role re reducing waste and increasing value of clinical trials.
**Results**
Macleod et al., e.g. State that more than 50% of the studies are designed without reference to systematic reviews of already existing evidence. As experimentation and research with humans is ethically only legitimate if the knowledge is not yet available and is at the same time definitely needed, a systematic review should be an absolutely essential part of each application dossier sent to an Ethics Committee. Unfortunately, even the recently passed European Clinical Trial Regulation 536/2014 which specifies the content of the application dossier in Annex I does not require the submission of such a systematic review. Another relevant issue is according to Macleod et al. That adequate steps to reduce bias are not taken in more that 50% of the studies and that there are still too many trials with inaedequate statistical power. Examples and explanations for these flaws and the ethical problems involved will be presented.
**Conclusions**
Ethics Committees can play an important role in improving the quality of clinical trials re substance, content and methods. It seems that Ethics Committees are not yet sufficiently aware of their responsibilities in this context. In addition there should be no Ethics Committee any more without sufficient biostatistical expertise.

### P246

#### Barriers and facilitators to statistical rigour in clinical trials - emerging themes from the literature

Marina Zaki[1], Eilish McAuliffe[2], Marie Galligan[3]
[1]School of Nursing, Midwifery and Health Systems, University College Dublin & Health Research Board - Trials Methodology Research Network; [2]School of Nursing, Midwifery and Health Systems, University College Dublin; [3]School of Medicine and Medical Sciences, University College Dublin
**Correspondence:** Marina Zaki
*Trials* 2017, **18(Suppl 1):**P246

**Background**
Rigorous clinical trial methodology is dependent on a number of factors, including but not limited to: appropriate team communication, funding and compliance with ethical, legal and regulatory frameworks. Factors relating to statistics and clinical data management (CDM) however, are crucial to the planning, design, conduct, monitoring, analyses and reporting of trials. Reliable results from statistical analyses are imperative to ensure confidence in the clinical interpretation of treatments. Some key trial statistical and CDM aspects include: choosing trial design, variables and outcomes, building databases, planning and implementing randomization schedules, sample size calculations, statistical monitoring and quality control, maintaining accurate statistical documentation, Source Data Verification, interim and statistical analysis and translating statistical results into clinically meaningful findings. One key aspect to ensuring this 'statistical rigour', is having competent and enthusiastic inter-disciplinary 'Trialists' - most notably: trial statisticians, data managers and principal investigators (PIs).
**Objectives**
The objectives were to: 1) Develop an understanding of roles and responsibilities of statisticians, PIs and CDM team members in order to 2) Better understand the barriers and facilitators to statistical rigour in clinical trials.
**Methods**
The Cochrane Library Databases, Google Scholar, pubmed and Web of Science were explored using P(Population), E(Exposure) and O(Outcomes) search terms, with no restriction on years. Snowballing yielded Grey literature and international guidelines.
**Results**
The literature discussed roles, responsibilities and rights of statisticians, but also of pis and CDM members - where the aforementioned trial statistical aspects are often a joint effort. Key barriers and facilitators to statistical rigour in trials were then identified from the literature.
A number of authors raise concerns of statisticians only being consulted after data collection - for analyses and reporting. It is strongly recommended however, to have skilled statisticians involved in the design and implementation, which may prevent statistical pitfalls during the trial. Errors in trial design, conduct and analysis may introduce bias and affect patient safety.
Authors emphasize the importance of statistician involvement in 'teaching and learning'. They teach and inform colleagues about important statistical information and interpretation of trial results. Similarly, statisticians have a responsibility to be knowledgeable about their therapeutic field of research, to be up-to-date with novel statistical approaches and have a firm understanding of trial methodology. Statisticians also have a responsibility to ensure final study reports are a 'fair reflection' of trial findings. Some authors also call for statisticians to be recognized as 'full-collaborators' in the decision-making aspects of trials, and maybe even as a 'co-investigator'.
'Barriers' to trial statistical rigour include: lack of availability to statistical expertise, timing and workloads and not adhering to regulations. Facilitators include: understanding clear roles of statisticians and CDM members in the oversight of certain procedures, adequate resources, qualifications and experience.
**Conclusions**
Key factors contributing to statistical rigour, in a trials context, are discussed. These findings support the importance of 'inter-disciplinary' teamwork. Increased understanding of each other's roles and more transparency in communication between statisticians, CDM members and healthcare professionals, is of critical importance to determining and communicating the clinical relevance of statistically significant findings.

### P247

#### Optimising trial recruitment with well-designed screening log: experiences from the ROCS study

Martina Svobodova[1], Lisette Nixon[2], Jane Blazeby[3], Anthony Byrne[4], Dougal Adamson[5]
[1]Cardiff University; [2]Cardiff University, Centre for Trials Research; [3]Centre for Surgical Research, School of Social and Community Medicine, University of Bristol; [4]Cardiff University School of Medicine, Marie Curie Palliative Care Research Centre; [5]Tayside Cancer Centre, Ward 32, Ninewells Hospital
**Correspondence:** Martina Svobodova
*Trials* 2017, **18(Suppl 1):**P247

**Background**

Many RC trials struggle to reach their pre-specified sample size. Screening logs offer an indication of eligibility and why patients do not enter the trials as well as providing required figures for the CONSORT diagram. The ROCS (Radiotherapy after Oesophageal Cancer Stenting) study is a pragmatic RCT of external beam radiotherapy in addition to stent versus stent alone in patients clinically assessed as requiring stent insertion for relief of dysphagia caused by oesophageal cancer.

**Aim**

Re-design a screening log to provide data to optimise study design and recruitment.

**Methods**

ROCS study screening logs were initially designed as a two-stage paper form. The first form included all patients requiring stent insertion, and then those potentially eligible were copied across onto a 2nd stage screening form. Research nurses were involved in the re-design of the screening logs at regular face to face ROCS Nurses Meetings. Modifications were made to criteria and subsequently the two forms were combined onto one Excel sheet. Nurses were advised to include all patients receiving an oesophageal stent for palliative reasons. Reasons for declining the study and for ineligibility could be selected from drop-down options. Completion electronically allowed less writing and nurses could email back the results weekly. Summary data was presented to the ROCS TMG regularly.

**Results**

After implementation, feedback from nurses was very positive, with 100% return rate, following reminders in some cases. The ongoing personal contact with individual sites improved their engagement with the study. Early in the trial, screening logs clearly demonstrated to the funders that the predicted number of patients requiring stent insertion was correct, and that 63% acceptance rate of the trial was above the 50% initially predicted. The reason for lower than expected recruitment was due to low eligibility of 26% against the original estimate of 70%. The main reason for ineligibility was patients identified only after the stent was inserted; 14% of patients were ineligible owing to this reason. The TMG provided this as evidence to funder and sponsor as reason to change the trial design. Since the change to allow patients to be recruited after stent 55% of patients have been randomised post stent. A change was also made to the histology eligibility criterion, but the effect of this has not been seen yet as it was only implemented recently. Screening logs also highlighted low acceptance rates in some centres, which allowed the trial team to provide more advice and support to these sites. One site improved acceptance rate from 35 to 50% following additional support.

**Conclusion**

Comprehensive screening log data can be collected. This is useful to track proportions of incident patients that are eligible and randomised. Data also provide information about non-eligibility and non-participation to feedback to centres, the funders and TMG. Commitment of the study centres played a key role to in the screening data return. This was easier to achieve through direct engagement at the regular investigator meetings and acting accordingly on participating centres feedback.

### P248

#### Mock activation of a pandemic influenza clinical trial: testing for rapid recruitment

Garry Meakin[1], Clare Brittain[1], Lelia Duley[1], Wei Shen Lim[2]
[1]Nottingham Clinical Trials Unit; [2]Nottingham University Hospitals NHS Trust
**Correspondence:** Garry Meakin
*Trials* 2017, **18(Suppl 1):**P248

**Background**

Conducting a clinical trial during a public health emergency creates novel challenges to successful execution and requires an innovative approach to trial design. The ASAP trial (Adjuvant Steroids in Adults with Pandemic Influenza) has been set-up in advance of an influenza pandemic ready to be rapidly activated in such an event. All approvals have been obtained, and documents and materials necessary for the conduct of the trial have been prepared. Upon activation, the trial needs to recruit the first participant within 4 weeks, and to complete recruitment of 2200 participants at approximately 40 sites within the first pandemic wave of approximately 6 weeks.

Even with the best of planning, unexpected barriers and issues affecting recruitment and trial conduct often occur; this is particularly likely to be true of a trial to be conducted during an influenza pandemic. As recruitment to ASAP must be completed within the first wave of the pandemic, it is important that key trial process have been tested, and adjusted if necessary, prior to activation of the trial and that the site activation plan is realistic and deliverable. We therefore conducted and evaluated a mock site activation.

**Methods**

Derby Royal Hospital was chosen as the target site given its close proximity to the coordinating centre and established links with the trial team. The site was alerted to activation via a “Declaration of Activation” letter which provided detailed information on the actions required to receive the “green light” for recruitment of a “patient” (volunteer) to commence.

The mock activation allowed for the assessment of: 1) Investigational Medicinal Product (IMP) manufacture, labelling and supply procedures 2) training material for local investigators and site staff on trial processes, 3) data management processes, 4) channels of communication between the coordinating centre and the mock activation site and 5) the recruitment pathway.

**Results**

The site was mock activated on 15th September 2015, with recruitment “green light” issued within 4 weeks, in conformity with trial targets. The mock activation of the trial provided reassurance to the trial team that trial processes and procedures were adequate for successful site activation and recruitment, it also helped to highlight a number of potential areas in which trial processes could be improved.

As a result changes are being made to the trial including minor amendments to the CRF and third party contracts, the streamlining of IT processes and increasing staff resource; this will help to further build trial resilience in the event of a pandemic, and reduce the burden of any queries generated by unclear processes at activation. An evaluation of the costs of conducting this mock activation will also be reported.

**Discussion**

Mock activation allowed trial processes to be tested and problems addressed before actual patient recruitment. Such activation may have wider relevance for streamlining trials where rapid recruitment is critical, or anticipated to be complex. Although mock activation has cost implications in time and resources, the investment may be worthwhile if it improves recruitment and trial conduct, improving trial efficiency.

### P249

#### Opportunities and pitfalls encountered when using the template for intervention description and replication (TIDIER) to develop a complex intervention to reduce obesity in men

Pat Hoddinott[1], Stephan U. Dombrowski[1], Marjon van der Pol[2], Frank Kee[3],Mark Grindle[1], Cindy Gray[4], Alison Avenell[2], Michelle McKinley[3], on behalf of the research team
[1]University of Stirling; [2]University of Aberdeen; [3]Queens University Belfast; [4]University of Glasgow
*Trials* 2017, **18(Suppl 1):**P249

**Objectives**

TIDIER guidance is an extension of CONSORT 2010 and SPIRIT 2013 statements and aims to improve intervention reporting [1]. Our objective was to use tidier to identify and reduce the uncertainties about the design of a complex behaviour change intervention prior to a feasibility randomised controlled trial to reduce obesity in men.

**Background**

Intervention development is seldom reported and can be viewed as a black box [2]. Many interventions either do not result in a successful trial or are not implemented and therefore do not impact on health outcomes. This contributes to considerable research waste. Carefully designed interventions that are desirable, acceptable, feasible and sustainable are required. In our study, intervention engagement and reach are crucial because the prevalence of obesity in men is high, men infrequently engage with weight loss interventions and there are considerable health inequality consequences.

**Methods**

We considered the literature on i) complex intervention development methods ii) behaviour change theory (psychological and economic); iii) systematic review evidence about weight loss; iv) health inequalities; v) the acceptability to men and the public for similar interventions. With Public Patient Involvement (PPI) and expert opinion, these sources were used to populate the tidier checklist. We then decided how to fill the gaps as robustly as possible in order to produce a replicable intervention manual.

**Results**

Some intervention features had no evidence to inform a decision, so we undertook a primary survey, qualitative research and PPI. Other intervention features e.g. Behaviour change components had informative data from studies of varying quality which generated hypotheses. A team decision was made about whether further primary research data was required or whether PPI and expert opinion would suffice. Some intervention features, particularly those relating to sustainability (e.g. Website, text messages) and future implementation (who delivers), had a strong underpinning logic which was considered sufficient for the team to make a decision. Tidier helped to focus on the decisions to be made. Limitations became apparent in relation to the intervention context, delivery [3] and how the loose categories for intervention features could be interpreted differently. Pragmatic decisions were sometimes required due to limits in funding, time and staff availability.

**Conclusions**

With little literature on how best to develop successful complex interventions that eventually translate into health service implementation, tidier guidance for reporting interventions provides a useful starting point. However, prospective development of guidance on intervention development may be preferable to retrospective approaches. Our study begins to systematically address the uncertainties and decisions involved to develop a complex intervention. This is necessary so that more interventions proceed to become successful trials, are implemented into policy and practice and have impact on health care outcomes.

### P250

#### Development of a complex intervention for patients with chronic pain after knee replacement

Vikki Wylde[1], Nicholas Howells[2], Wendy Bertram[2], Andrew Moore[1], Julie Bruce[3], Candy McCabe[4], Ashley Blom[2], Jane Dennis[1], Amanda Burston[1], Rachael Gooberman-Hill[1]
[1]University of Bristol; [2]North Bristol NHS Trust; [3]University of Warwick; [4]University of West of England
**Correspondence:** Vikki Wylde
*Trials* 2017, **18(Suppl 1):**P250

**Background**

Over 70,000 primary total knee replacements are performed annually in the NHS. People choose to undergo knee replacement with the hope that surgery will improve their pain, but approximately 20% of people who have primary total knee replacement experience chronic pain afterwards. Our research has demonstrated that current UK NHS service provision for people with chronic pain after knee replacement is patchy and inconsistent. This reflects an absence of evidence about effective interventions and highlights the need to develop and evaluate interventions to address chronic pain after knee replacement. We have developed a complex intervention comprising a novel assessment clinic and onward referral pathway for patients reporting moderate-severe pain at 3 months after knee replacement. The initial development of the intervention was informed by a systematic review, survey of NHS service provision, qualitative work with health professionals, consensus meetings with pain experts and patient and public involvement activities. The aim of this work was to refine the design and delivery of this intervention before evaluation in a randomised trial, in keeping with the Medical Research Council’s recommendations for complex intervention development.

**Methods**

Three stages of work were undertaken over a 12 month period. To develop the intervention, the first stage involved consensus questionnaires with 22 health professionals about the appropriateness of individual components within a draft care pathway intervention. Mean appropriateness ratings were calculated and discussed at meetings with 18 healthcare professionals. To refine delivery of the intervention and assess whether it was acceptable to patients, Stage 2 involved scrutiny of the trial intervention with 10 patients who attended an assessment clinic. Stage 3 involved 10 health professional stakeholders to evaluate their views about implementation potential of the intervention using a questionnaire based on the nomad instrument.

**Results**

In Stage 1 a number of substantive changes to the design of the intervention were made, including the addition of a physiotherapy referral pathway and rapid access to suitable medications for neuropathic pain. Running the intervention in Stage 2 found that the assessment clinic was acceptable to patients and highlighted the need for some changes to the clinic processes, including the need for additional self-report screening tools and standardised radiographs. This work also informed development of a comprehensive training package for Extended Scope Practitioners who would deliver the intervention during the trial. Stage 3 found that stakeholders understood the intervention and could see how the intervention would affect the nature of their own work. They were aware of the proposed benefits of the intervention for patients and were keen to engage with the new practices.

**Conclusions**

We have undertaken a comprehensive programme of research to refine the design and development of a complex intervention prior to evaluation in a randomised trial. Our study provides an example of the methods that can be used to address key questions within intervention design in a relatively tight timeframe. The next stage is to evaluate the clinical and cost-effectiveness of the intervention in a definitive multi-centre randomised trial, which will include an internal pilot phase.

### P251

#### Use of routine databases to aid the design of multicentre surgical trials with length-of-stay as the primary outcome

Olympia Papachristofi, Linda Sharples
London School of Hygiene and Tropical Medicine
**Correspondence: Olympia** Papachristofi
*Trials* 2017, **18(Suppl 1):**P251

**Background**

Complex surgical interventions are an indispensable part of modern healthcare and there is increasing recognition that novel procedures should undergo the same rigorous evaluation as other non-invasive treatments. However, the multi-component nature of surgery complicates evaluation. For instance, surgical procedures are delivered by multidisciplinary teams and thus their outcome may vary due to patient characteristics, skill of the operators and the environment within which they are conducted. Recognition and accommodation of this variation is important in order to design adequately powered related trials. The duration of postoperative Length-Of-Stay (LOS) (or incidence of prolonged hospitalisation) has been the focus of many surgical trials as it is a principal driver of surgical costs, and acts as a surrogate for a range of post-operative complications. Therefore it is important to understand how and why this outcome varies, so that recommendations for trial design and analysis can be made. Traditionally, surgical trial design suffered from a lack of detailed multicentre data. The current availability of high-quality, routinely collected administrative databases allows us to explore current practice and outcomes, in order to inform trial design in this context.

**Aims**

This study aims to demonstrate how routine databases can be used to explore variation induced by patients, provider and centre practices in LOS outcomes, in order to inform surgical trial design and estimate key design parameters.

**Methods**

We start by exploring the variation between surgeons and anaesthetists separately, whilst adjusting for patient heterogeneity, using hierarchical (random effects) models. In order to estimate the contribution of different components of care and their interactions to variation in outcomes, a series of hierarchical models with cross-classifications is employed. Using the two most influential providers in the surgical treatment pathway, the surgeon and anaesthetist, for illustration, we show that key components of surgery do not necessarily follow a strict hierarchy e.g. Patients are nested within surgeons nested within centres, but surgeons are not nested within anaesthetists. We extend the proposed models to accommodate an additional Centre level in the hierarchy which introduces further variation due to infrastructure and policy differences. Potential drivers of between-centre variation are further examined through the incorporation of random coefficients. As there may be multiple components that contribute to extended LOS, we demonstrate how we can identify those which can be more effectively manipulated in order to standardise practice in trials. We examine the LOS both as continuous outcome, appropriately addressing its non-normality, and as a binary outcome (prolonged hospital stay).

**Results**

An application of the methods in cardiac surgery, one of the most expensive yet widely used surgery types, is presented using a cohort of more than 100,000 consecutive case series patients from ten UK specialist centres. The implications of the results for the design of related trials are also discussed.

**Conclusions**

High-quality routine databases can be used to identify sources of variation in surgical care and outcomes. The resulting outputs can then be used to inform surgical trial design and analysis to ensure the robust and efficient analysis of intervention effects.

### P252

#### Process evaluation for the PREPARE-ABC study: context mapping, pinchpoints and implications for implementation and theoretical fidelity

Jamie Murdoch[1], Anna Varley[1], Jane McCulloch[1], John Saxton[2], Erika Sims[1], Jennifer Wilkinson[1], Megan Jones[1], Juliet High[1], Allan Clark[1], Sue Stirling
[1]University of East Anglia; [2]Northumbria University
**Correspondence:** Jamie Murdoch
*Trials* 2017, **18(Suppl 1):**P252

**Background**

Process evaluations assess the implementation and sustainability of healthcare interventions within clinical trials, offering explanations for observed effects of trial findings and specifying the circumstances under which interventions are likely to succeed or fail. Such evaluations are particularly needed in trials of complex interventions which contain multiple interacting components. However, while theoretical models are available for evaluating intervention delivery within specific contexts, there is a need to translate conceptualisations of context into analytical tools which enable the dynamic relationship between context and intervention implementation to be captured and understood.

**Methods**

In this paper we propose an alternative approach to the design, implementation and analysis of process evaluations for complex health interventions through a process of 'context mapping.' This innovative technique involves: 1) prospectively mapping contextual features likely to affect intervention delivery; 2) using the mapping exercise to identify likely pinchpoints in delivery; and 3) analysing implementation at the pre-identified pinchpoints during delivery. As an example, we will present ongoing work from PREPARE-ABC - a randomised controlled trial of suportive Exercise Programmes for Accelerating recovery after major abdominal Cancer surgery. PREPARE-ABC, funded by the NIHR, sponsored by Norfolk and Norwich University Hospitals NHS Foundation Trust and co-ordinated by the Norwich Clinical Trials Unit, University of East Anglia, is recruiting 20 hospitals and 1146 patients in the UK requiring surgery for colorectal cancer. Patients are randomised to one of three arms: hospital based supervised exercise; home based supported exercise; or treatment as usual.

**Results**

Data collection is ongoing at the time of submission. We will present findings from our current evaluation of standard care for patients pre and post-surgery for colorectal cancer, conducted prior to main trial recruitment. We will discuss what recommendations can be made from these findings for improving main trial implementation, using qualitative field notes from observations of pre and post-surgery consultations, and quantitative and qualitative data obtained through a telephone scoping exercise conducted at all colorectal units participating in the study.

**Conclusions**

The value of context mapping is that we can predict areas of vulnerability prior to intervention delivery, then make recommendations for adapting flexible elements of the intervention during implementation. In addition, we can target and observe key pinchpoints as they are enacted, thereby offering opportunities for exposing the 'active ingredients' of interventions in action and providing insights into implementation and theoretical fidelity.

### P253

#### Can complex intervention clinical trials capture treatment effects using a single primary outcome?

Ranjit Lall, Chen Ji
The University of Warwick
**Correspondence:** Ranjit Lall
*Trials* 2017, **18(Suppl 1):**P253

For many decades, the randomised controlled clinical trial has been the gold standard of conducting research studies in health care. Its design and aims orientate around proving or disproving hypotheses based on the efficacy or safety of an intervention powered for a single primary outcome measure. However, in the awake of many medical techniques and devices, that reveal the complexity and depth of a disease, treatments such as complex interventions, are often assessed to obtain a comprehensive picture of these multiple manifestations. In order to support this, a single end-point will not provide sufficient information that is adequate in treatment assessment. The MRC complex intervention framework guidance (2008) states "Identifying a single primary outcome may not make best use of the data; a range of measures will be needed, and unintended consequences picked up where possible." The choice of more than one primary outcome measure seems to be perfectly plausible from a clinical view point, but statistically it presents many complexities.

The aim of this article is to present the different types that have been detailed in the literature which aim to assess multiple outcomes in clinical trials, which are considered to be equally important in the assessment of the treatment effect. They include (i) co- primary outcomes and (ii) composite outcomes. We outline the challenges faced in the sample size calculation and the statistical analysis of co-primary outcomes, given different distributions and approaches of analysis. We also illustrate example of clinical trials where co-primary outcomes have shown treatment efficacy, which is not evident with a single primary outcome. For a composite outcome, we summarise the challenges faced in the analysis and reporting and interpretation of results. In addition, we illustrate the pitfalls and strengths of these approaches.

### P254

#### Exploring automated free SMS from email in clinical trials

Amarnath Vijayarangan
Emmes Services Pvt Ltd.
*Trials* 2017, **18(Suppl 1):**P254

Short Message Service (SMS) is one of the basic functionality available in all types of mobile phones. Research had shown that almost all SMS are getting read as soon as they reach. It is the easiest route to anyone to be notified as it does not require computer/internet. It is reliable and secured in all the situations as it is completely monitored and controlled by the mobile service providers. One might be surprised to know that SMS can be sent from Email to mobile number at free of cost. Every mobile number is uniquely attached to an email address with the domain chosen by the mobile carriers. Everyone will be interested to make use of a feature if it is available at free and at the same time reliable. The proposed approach is exploring and automating the feature of sending SMS to mobile phones from Email to serve the following various activities in clinical trials. 1. Reporting/Notifying the stake holders about importance occurrences of events 2. Notifying the programming team about the current program status. These two activities are completely SAS data driven. In clinical trials, SAS is one of the widely used software for the data management, analysis and reporting since the clinical trial datasets are often available in SAS format. The analysis reports (Tables, Listings & Figures) are generated using SAS programming language for the FDA submission. SAS programming language based automation is implemented for these two activities. 3. Engaging Study participants 4. Reminder notifications to the project team on various activities. These two activities are completely Microsoft Excel data driven. Excel is one of the Microsoft applications always available in all the computers and VBA is the language of Excel application. Using Excel VBA programming these tasks are automated. The data based automations are always developed using at least one programming language. In order to send the n number of SMS from Email without any manual intervention, we are making use of SAS and Excel VBA programming languages. The SMS can be triggered from Email at a scheduled interval or whenever certain criterion is met. Even though the whole process can be automated using only SAS programming language, we have come up with Excel VBA based automation as well since SAS is very expensive software and hence cannot be made to be available for all the project team members. As Excel VBA is available in everyone's computer the VBA based proposed approach can be utilized for various activities even if SAS is not available. This proposed approach has wide range of cost effective applications which can be quickly leveraged to perform various activities depending on the study requirement.

### P255

#### Electronic data capture; changing data management at ICR-CTSU

Charlotte Friend, Lisa Jeffs, Deborah Alawo, Leona Batten, Joanna Illambas, Judith Bliss, Emma Hall, Claire Snowdon, Alexa Gillman, Rebecca Lewis
The Institute of Cancer Research
**Correspondence:** Charlotte Friend
*Trials* 2017, **18(Suppl 1):**P255

**Background**

ICR-CTSU introduced Electronic Data Capture (EDC) in 2012; this necessitated a revision of data management systems and processes. In trials using paper case report forms (CRFs) –"paper trials" – data managers (DMs) at ICR-CTSU manually check all paper CRFs during data entry. With the introduction of EDC, they now perform manual checks and additional programmed checks using data review software. Here we describe how data management has changed as a result of the transition from paper to EDC trials.

**Implementation**

For paper trials, CRFs are received by ICR-CTSU and are entered onto the database by DMs. CRFs are stored chronologically in a patient folder, and are available for review at the time of data entry. Manual checks are performed on all CRFs when the DMs at ICR-CTSU transcribe data into the database. In-built database validations flag discrepancies to DMs s during data entry. Resulting queries are documented, printed and sent to sites periodically and several months can pass before query resolution occurs. Data entry of different forms at any one time means trends in errors in data reporting are not always easily identified. In EDC trials, participating sites enter data into an EDC system. Database validations highlight potential discrepancies to sites at the time of data entry so that they can correct issues immediately, reducing the number of data queries required. DMs at ICR-CTSU receive daily automated emails listing newly completed electronic CRFs from the database tracking system. Manual review of specific forms is performed as required, for example, on trial entry forms and important safety and endpoint data. Only one form per patient can be open in the database for review at a time therefore DMs also use advanced data review software to programme checks which identify data discrepancies across forms for all trial participants. DMs run programmed checks at a frequency determined by their priority, to systematically identify potential discrepancies. Data are reviewed in context, and queries are raised electronically. These are immediately available for sites to review and provide a response.
Trends in data entry errors can be readily identified during review of the programmed checks. This allows specific data entry guidance targeting common errors to be provided to sites sooner and changes to the database or CRFs can be considered by the ICR-CTSU trial team earlier. Sites are therefore less likely to make the same errors during future data entry.

**Conclusion**

DMs at ICR-CTSU typically work on a combination of trials which may include both paper and EDC, requiring excellent time management skills and flexibility. Attention to detail, investigational skills and effective communication remain crucial, however the transition to EDC trials requires development of additional competencies and technical expertise in order to support DMs to produce and review programmed listings.

### P256

#### Using central statistical monitoring to drive quality into clinical trials

Erik Doffagne
Cluepoints
*Trials* 2017, **18(Suppl 1):**P256

Following encouragements from FDA guidance and the recent ICH E6 Addendum, many organizations are adopting RBM (Risk-based Monitoring). There is no single solution since RBM usually relies on a combination of on-site monitoring visits and central monitoring methods. CSM (Central statistical Monitoring) can play a major role in the RBM strategy in detecting investigational sites with atypical patterns in the collected data.
The aim of this presentation is to share lessons learned and best practice in terms of integration of CSM within the clinical operation processes. In particular, emphasis will be given on how the outcomes from CSM can be utilized in order to drive on-site monitoring efforts and in identifying areas with potential risk.

### P257

#### Managed access to NIHR-funded research data: an opportunity for all?

Liz Tremain[1], Elaine Williams[1], Tom Walley, CBE[2]
[1]NIHR Evaluation, Trials and Studies Coordinating Centre; [2]University of Liverpool
**Correspondence:** Liz Tremain
*Trials* 2017, **18(Suppl 1):**P257

The sharing and re-use of data for further hypothesis generation, interrogation and analysis is now universally recognised as a key principle in research. Furthermore there is acceptance that data generated by public funding, through participation of patients and the public, should be put to maximum use by the research community and, whenever possible, translated to deliver patient benefit. The National Institute for Health Research (NIHR) is a major public funder of research in the UK, and is committed to transparency. The NIHR position on data sharing and access is an important factor supporting this, with its standard research contract containing a clause regarding data release for many years, and the NIHR Journals Library requiring a specific data sharing statement since 2015 for all its open access publications. Mechanisms and processes for sharing data have recently been subject to a great deal of global debate and discussion. Although a consensus has now largely been achieved on the "Why" Aspect, early activity has been stalled to a certain extent by an inability to address the "How", and provide suitable and affordable repositories to permit data sharing, discoverability and access. Against the backdrop of recent international developments, NIHR has reviewed its requirements and processes on access to data to ensure that NIHR activity was appropriately reflected and that funded researchers were supported. A number of initiatives focused on or related to data sharing have been developed which have all built on the iom/NIH report 'Sharing Clinical Trial Data' published in 2015. Key areas include the publication of the ICJME proposal on data sharing following publication, proposals from the MRCT Center at Harvard for 'Vivli' (as a centre for Global Clinical Research Data), and the ongoing development of the Clinical Study Data Request (CSDR) system. In light of these developments, the NIHR is developing a revised position on the sharing of Anonymised Individual Participant Data (IPD) generated by NIHR-funded research and a managed-access system to support this. This seeks to build upon and strengthen NIHR activity in this area and initially includes; Confirmation that anonymised datasets from NIHR funded research should be available for further analysis wherever possible. NIHR data will be released via a 'Managed Access' System, subject to data use agreement. NIHR protocols should contain a 'Data Sharing Plan' which will be publically available. NIHR publications must include a data sharing statement/access link which clearly explain how data can be requested. The NIHR is aware of the demands placed on researchers in this area, and the need to retain a focus on this area. As a result it is noted that NIHR requirements and support will need to evolve as the wider data agenda develops.

### P258

#### Improving data quality though routine automated reporting using standard statistical software

Rosie Harris, Lauren J. Scott, Chris A. Rogers
Clinical Trials and Evaluation Unit
**Correspondence:** Rosie Harris
*Trials* 2017, **18(Suppl 1):**P258

**Background**

Efficient and timely monitoring of data throughout the conduct of randomised controlled trials (RCTs) is essential to ensure high quality data and robust results; monitoring may include summaries of recruitment, data completeness and data queries. Here we focus on efficient methods for monitoring completeness of trial data.

**Methods**

We have developed a Stata program that enables the user to simply and effectively monitor data completeness rates. The program allows the user to look at the overall completeness of case report forms (CRFs) or at the completeness of individual data fields. To use the program, three variables must be specified: the variable to be summarised; an indicator for the subjects to be included in the summary; and the variable by which the completeness of the variable of interest is to be grouped. The program also handles conditional data (i.e. Where the requirement for a response is conditional on the answer to a preceding question). The program directly outputs the results to Microsoft Excel, where they can be further manipulated if required. The generated output contains the number and percentage of entries with data present and the number and percentage of entries with data missing, by group and overall.

**Results**

An example of the basic output (for one group). In this example, CRF A1 is present for 121 study participants in group 1 (95.3%) and missing for 6 participants in this group (4.7%).

**Discussion**

Writing the code to produce the tables is straight forward to do. The program has been successfully used to produce data completeness reports in several multicentre RCTs. It has significantly reduced the time needed to prepare reports for study meetings and independent oversight committees and has removed the risk of transcription errors. The reports produced have been consistently well received. The program can easily be used for reporting on other key aspects of trial conduct not just completeness of data. Efficient routine central monitoring of trial conduct can serve to highlight issues early and help minimise risks to a trial.

**Disclaimer**

This work was supported by the National Institute for Health Research (NIHR) Health Technology Assessment (HTA) programme and by the NIHR Bristol Biomedical Research Unit in Cardiovascular Disease at University Hospitals Bristol NHS Foundation Trust. The views expressed are those of the authors and not necessarily those of the NHS, the NIHR or the Department of Health.

### P259

#### Chinese herbal medicine (CHM) for recurrent UTIs in women, a feasibility trial

Kim Harman[1], George Lewith[2], Felicity Bishop[3], Beth Stuart [4], Andrew Flower[2]
[1]University of Southampton; [2]University of Southampton, Complementary and Alternative Health; [3]University of Southampton, Faculty of Social, Human and Mathematical Sciences; [4]University of Southampton, Primary Care & Population Sciences
Correspondence: Kim Harman
*Trials* 2017, **18(Suppl 1):**P259

**Background**

Chinese herbal medicine (CHM) has a history of treating the symptoms of UTIs for >2000 years. In the UK UTIs are the commonest bacterial infection presented by women within Primary care. RUTIs have a significant negative effect on QOL, impact hugely on health care costs from outpatient visits, diagnostics and prescriptions. Current treatment of RUTIs relies heavily on antibiotics.

**Objectives**

To explore the feasibility of conducting a clinical trial of CHM within a primary care setting with particular reference to recruitment, referral patterns, compliance, drop out rates, the relevance of outcomes measures, the QOL of participants, adverse effects, and differences between standardised/individualised remedies.

To compare outcomes of duration and severity of acute UTIs, rates of re-infection, measuring acute and prophylactic antibiotic use, and evaluating long-term changes. These preliminary data may be used to inform a future, adequately powered, definitive study.

Variations in the time herbs were taken will be explored with the outcomes as there is no definitive length of time recognised - it will vary from 4 to 16 weeks.

**Method**

A pragmatic, double blinded randomised controlled feasibility study involving 4 groups of 20 women, using standardised or individualised CHM for RUTIs in Primary care and traditional care. Women with a history of RUTIs will be identified by medical record searches and invited to participate. Within the Wessex, Western and Peninsula regions allocation will be to the standardised arm of the trial (Primary care). Women from London and Hove will be allocated to individualised CHM treatment. MHRA approval was needed for the standardised arm but not the individualised arm.

**Results**

Recruitment is challenging and varies greatly by region. Total recruitment to date for the standardised arm n = 26, better in Peninsula than anywhere else, for the individual arm total recruitment n = 21, more participants found in Brighton & Hove by Kent, Surrey and

Sussex CRN compared to two London CRNs. Numbers finishing to date are in the standardised arm $n = 1$ with $n = 5$ loss to follow up and $n = 1$ withdrawal and in the individualised arm $n = 7$ with $n = 2$ lost to follow up.
We have yet to analyse the full data for recruits including diaries for QOL data.
We will be interviewing both NHS staff and participants for their views on CHM and the processes involved.
The trial finishes recruitment at the end of October 2016 as the herb expiry date is the end of November 2016.

### P260

#### A qualitative feasibility study to inform fluids in shock (FISH) - a pilot randomised controlled trial of fluid bolus therapy in septic shock

Caitlin O'Hara[1], David Inwald[2], Ruth Canter[3], Paul Mouncey[3], Mark D. Lyttle[4], Simon Nadel[2], Mark Peters[5], Kerry Woolfall[1]
[1]The University of Liverpool; [2]St Mary's Hospital, Imperial College Healthcare NHS Trust; [3]Intensive care national audit & research centre (ICNARC); [4]Emergency Department, Bristol Royal Hospital for Children; [5]Institute of Child Health, University College London
Correspondence: Caitlin O'Hara
*Trials* 2017, **18(Suppl 1):**P260

This abstract is not included here as it has already been published.

### P261

#### Value of a sister observational cohort alongside a randomised controlled trial with an internal feasibility phase

Janet Dunn[1], Andrea Marshall[1], Maria Ramirez[1], Andy Evans[2], Peter Donnelly[3]
[1]Warwick Clinical Trials Unit, University of Warwick; [2]Ninewells Medical School; [3]South Devon Healthcare NHS Foundation Trust
**Correspondence:** Janet Dunn
*Trials* 2017, **18(Suppl 1):**P261

Observational cohorts alongside randomised controlled clinical trials can be very informative. They enable an assessment of the number of patients that do not want to be randomised and reasons for non-participation in the randomised controlled trial (RCT). They also allow investigation of what is standard practice at each site. They can be extremely useful in hard to recruit trials to gain information about the outcomes of these patients in standard clinical practice. Mammo-50 is a multi-centre RCT of different mammographic surveillance schedules for breast cancer patients aged 50 years or older at diagnosis. A total of 5000 patients are randomised to annual surveillance versus 2-yearly for conservation surgery and 3-yearly for mastectomy patients. There was a 24 month pre-planned internal feasibility study assessing recruitment, acceptability to be randomised and logistical endpoints which included a sister observational cohort. The aim of the cohort was to assess standard practice for non-randomised patients in terms of information given to patients, type of follow-up at each centre and frequency of mammographic surveillance. During the 24 month feasibility phase of the trial, 1354 patients were enrolled into the study; 936 (69%) patients choosing to participate in the RCT and 418 (31%) patients were recruited into the sister observational cohort study. The main reason for not going into the RCT but being part of the observational cohort was patient choice. Patients wanted to remain on their standard mammographic surveillance and didn't want the possibility of changing regardless of whether the standard was more or less frequent. Cohort patients have similar baseline patient characteristics to those entering the RCT, although the cohort did contain slightly more patients who had undergone mastectomy for their breast surgery. The feasibility phase demonstrated that the trial was acceptable by patients and clinicians but the ratio of patients entering the cohort compared to the RCT increased over the duration of the feasibility phase. The cohort demonstrated that standard practice regarding mammographic surveillance and follow-up is highly varied across sites. In conclusion the observational cohort can provide valuable information about a population of patients that are not willing to participate in a RCT. The purpose of the cohort should be clear and informative. Recruiting into a sister observational cohort may be seen by sites as an easier option and thus detract from recruiting patients into the main RCT. It may be advantageous to close the observational cohort at a time when sufficient numbers are recruited and the aims of the cohort fulfilled. Mammo-50 demonstrated the strength of a sister observational cohort alongside the RCT, especially within the internal feasibility stage, leading to the success of the full RCT.

### P262

#### Internal pilot sample size re-estimation in paired comparative diagnostic accuracy trials with a binary response

Gareth McCray[1], Andrew Titman[2], Paula Ghaneh[3], Gillian Lancaster[1]
[1]Keele University; [2]Lancaster University; [3]University of Liverpool
**Correspondence:** Gareth McCray
*Trials* 2017, **18(Suppl 1):**P262

##### Background
The sample size required to power a trial to a nominal level in a paired comparative diagnostic accuracy trial, i.e. Trials in which the diagnostic accuracy of two testing procedures are compared relative to a gold standard, depends on the correlation between the two diagnostic tests being compared. The lower the correlation between the tests the higher the sample size required, the higher the correlation between the tests the lower the sample size required. A priori, we usually do not know the correlation between the two tests and thus cannot determine the exact sample size. Furthermore, the correlation between two tests is a quantity for which 1) it is difficult to make an accurate intuitive estimate and, 2) it is unlikely estimates exist in the literature, particularly if one of the tests is new, as is very likely to be the case.
One option, suggested in the literature, is to use the implied sample size for the maximal negative correlation between the two tests, thus, giving the largest possible sample size. However, this overly conservative technique is highly likely to be wasteful of resources and unnecessarily burdensome on trial participants - as the trial is likely to be overpowered and recruit many more participants than needed. A more accurate estimate of the sample size can be determined at a planned interim analysis point where the sample size is re-estimated - thereby incorporating an internal pilot study into the trial design, with the intention of producing an accurate estimate of the correlation between the tests into the trial.
#### Methods
This paper discusses a sample size estimation and re-estimation method based on the maximum likelihood estimates, under an implied multinomial model, of the observed values of correlation between the two tests and, if required, prevalence, at a planned interim. The method is illustrated by comparing the accuracy of two procedures for the detection of pancreatic cancer, one procedure using the standard battery of tests, and the other using the standard battery with the addition of a PET/CT scan all relative to the gold standard of a cell biopsy. Simulation of the proposed method are also conducted to determine robustness in various conditions.
#### Results
The results show that the type I error rate of the overall experiment is stable using our suggested method and that the type II error rate is close to or above nominal. Furthermore, the instances in which the type II error rate is above nominal are in the situations where the lowest sample size is required, meaning a lower impact on the actual number of participants recruited.
#### Conclusion
We recommend a paired comparative diagnostic accuracy trial which used an internal pilot study to re-estimate the sample size at the interim. This design would use a maximum likelihood estimate, under a multinomial model, of the correlation between the two tests being compared for diagnostic accuracy, in order to more effectively estimate the number of participants required to power the trial to at least the nominal level.

### P263

#### How does prevalence affect the size of clinical trials for treatments of rare diseases?

Siew Wan Hee[1], Adrian Willis[1], Catrin Tudur Smith[2], Simon Day[3], Frank Miller[4], Jason Madan[1], Martin Posch[5], Sarah Zohar[6], Nigel Stallard[1]
[1]University of Warwick; [2]University of Liverpool; [3]Clinical Trials Consulting and Training Limited; [4]Stockholm University; [5]Medical University of Vienna; [6]INSERM
**Correspondence:** Siew Wan Hee
*Trials* 2017, **18(Suppl 1):**P263

**Background**
Clinical trials are typically designed based on the classical frequentist framework constrained to some pre-specified type I and II error rates. Depending on the targeted effect size, the sample size required in such designs range from hundreds to thousands. Trials for rare diseases with prevalence 1/2000 or fewer may find it challenging to recruit patients to trials of large size. In this work, we examine the relationship between prevalence and other factors with the size of interventional phase 2 and 3 trials conducted in the US and/or EU.
**Methods**
We downloaded all trials from Aggregate Analysis of clinialtrials.gov (AACT) in May 2016 and identified rare disease trials by matching mesh terms in AACT with those in Orphadata. Actual sample sizes of completed trials or anticipated sizes of non-completed trials were used for analysis. We investigated effects of trials' characteristics such as: inclusion criteria (e.g. Gender, age), intervention model (e.g. Factorial design, single arm), lead sponsor type (e.g. Industry, US Federal Agency), trial location, number of countries involved in the trial, year that enrolment to the protocol began, number of interventions in the trial, whether or not the trial had a data monitoring committee and whether or not the intervention studied in the trial was FDA regulated on sample size. The effect of prevalence on sample size was tested adjusting for phase, interaction between prevalence and phase, and all other significant covariates.
**Results**
Of the 186941 trials in clinicaltrials.gov, 1567 (0.8%) were studying one rare condition only and with prevalence information from Orphadata. There were 19 trials studying disease with prevalence <1/1,000,000, 126 trials with 1-9/1,000,000, 791 trials with 1-9/100,000 and 631 trials with 1-5/10,000. Of these, 1160 were described as phase 2 trials. All the covariates described above except type of lead sponsor were significant and thus, were included in the regression model. The estimated mean sample sizes in phase 2 trials were similar across all prevalence classes after adjusting for other covariates; 15.7 (95% CI, 8.7-28.1), 26.2 (16.1-42.6), 33.8 (22.1-51.7) and 35.6 (23.3-54.3) for prevalence <1/1,000,000, 1-9/1,000,000, 1-9/100,000 and 1-5/10,000, respectively. Phase 3 trials of rarer diseases (<1/1,000,000 and 1-9/1,000,000) had similar size (estimated mean, 19.2, 6.9-53.2 and 33.1, 18.6-58.9, respectively) to those in phase 2 but were statistically significant lower than the slightly less rare diseases (1-9/100,000 and 1-5/10,000) trials (estimated mean, 75.3, 48.2-117.6 and 77.7, 49.6-121.8, respectively).
**Conclusions**
Our study shows that there was association between prevalence and the size of phase 3 trials with rarest diseases trials noticeably smaller than the less rare diseases trials. However, prevalence was not associated with the size of phase 2.

### P264

#### Predictive power in feasibility study design: implementation in the interim melanoma cancer trial

Wendi Qian[1], Andrea Machin[1], Pippa Corrie[2]
[1]Cambridge Clinical Trials Unit - Cancer Theme; [2]Cambridge Cancer Centre, Cambridge University Hospitals NHS Foundation Trust
**Correspondence:** Wendi Qian
*Trials* 2017, **18(Suppl 1):**P264

Cancer clinical trials are expensive in terms of running costs and time taken to complete, while the success rate of phase III cancer interventional trials is low, around 34%. Many novel trial designs, especially using adaptive and Bayesian methods, have been developed to help reduce costs and improve success rate of such trials. These novel techniques, however, are not always easily implemented in practice. We propose to apply the predictive power concept in the design of feasibility studies.
BRAF + MEK inhibitor drugs are effective treatment for patients with BRAF mutant melanoma, but benefit is limited by secondary resistance and toxicity. Clinical case reports and animal models suggest intermittent dosing may delay onset of resistance and reduce side-effects. The INTERIM trial is an open label two-arm randomised phase II feasibility trial designed to investigate the intermittent BRAF + MEK inhibitor drug dosing. Eligible patients will be randomly allocated to either the intermittent or the standard continuous dosing arm. Since there are concerns whether patients and their doctors will accept allocation to less than standard treatment, the overall objective of the study is to determine the acceptability of randomisation and compliance of patients allocated intermittent BRAF + MEK inhibitor drug dosing. The study will also investigate the probability of a successful follow-on phase III trial, measured using the predictive power.
The definitive phase III trial will be powered to test the hypothesis that intermittent dosing will prolong progression-free survival (PFS) compared with standard continuous dosing. It will need ~1000 patients (846 PFS events) to detect a hazard ratio (HR) = 0.80 with a 5% significance level and 90% power. The log(HR) is normally distributed, and therefore the predictive power could be easily calculated.
Assuming a prior of no difference and 1 PFS event, that is log(HR) ~ N(0, 4), a significance level 5% and 90 PFS events observed in the feasiblity study:
If the observed HR = 0.80 (an improvement in median PFS from 12 to 15 months) in the phase II feasibility trial, the predictive power to reject the null hypothesis of no PFS benefit of intermittent dosing schedule under the planned phase III trial (with 846 PFS events) is 72%. If the observed HR = 0.75 (an improvement in median PFS from 12 to 16 months) in the phase II trial, the predictive power to reject the null hypothesis of no PFS benefit of intermittent dosing schedule under the planned phase III trial (with 846 PFS events) is 81%.
We plan to recruit a total of $n = 150$ patients (75 patients in each arm) to the INTERIM trial in 18 months. It is anticipated that approximately 90 PFS events will be observed with a minimum of 9 months follow up.
The INTERIM trial proposal has received funding from the UK RFPB NIHR programme. The concept of predictive power will provide us with confidence to conclude whether the follow-on phase III trial is justified, while it is easily implemented in practice.

### P265

#### Core items for a standardised resource-use measure (ISRUM): expert Delphi consensus survey

Joanna Thorn[1], Colin Ridyard[2], Ruth Riley[1], Sara Brookes[1], Dyfrig Hughes[2], Sarah Wordsworth[3], Sian M. Noble[1], Gail Thornton[4], William Hollingworth[1]
[1]University of Bristol; [2]Bangor University; [3]University of Oxford; [4]PPI Representative
**Correspondence:** Joanna Thorn
*Trials* 2017, **18(Suppl 1):**P265

**Background**
Economic evaluations are commonly conducted within randomised controlled trials in order to assess value for money by measuring both the costs and the outcomes associated with a particular intervention. However, resource-use measurement by patient recall in economic evaluations is characterised by inconsistency and a lack of validation. A well validated standardised resource-use measure could increase data quality, improve comparability between studies and reduce research burden on patients and health economists.
**Aim**
To identify a minimum set of core items of resource use that should be included in a standardised instrument for health economic evaluations conducted in the United Kingdom (UK).

#### Methods
The content of 59 questionnaires in the Database of Instruments for Resource-Use Measurement (www.dirum.org) was analysed to generate a list of 60 resource-use items relative to an NHS and personal social services perspective (e.g. Visits to healthcare professionals in the community; prescribed medications). An electronic Delphi survey was developed, and health economists with experience of conducting economic evaluations in the UK were recruited through personal approaches and a general request to the Health Economists Study Group mailing list (a professional group based in, though not restricted to, the UK). Respondents were asked to rate items on a scale of 1 to 9 according to the importance of the item in a generic context, and were encouraged to comment on their choices or add additional items. Responses were used to identify items considered less important according to predefined consensus criteria. A second round was developed, in which feedback from round 1 (median responses to each item and summarised comments) was presented to respondents, alongside a reminder of their own scores. An individualised email was sent to each participant from round 1, and round 2 participants were asked to re-rate items. A final item selection meeting of the project team and a representative of the participants was held to discuss the results of the Delphi.

#### Results
45 health economists with wide-ranging trial experience completed round 1. Following application of the consensus criteria, items such as length of outpatient appointments (median = 3) and email/text communications with healthcare professionals (median = 4) were considered less important. The list of 60 items was reduced to 34 items for the second round; no new items were added. 42 respondents completed round 2 (93.3% of the original respondents), and the list of items considered important converged towards a shorter list where there is greater consensus about importance (including hospital outpatient appointments (median = 9) and appointments at GP surgeries (median = 9)). Following the meeting to discuss the results, a list of 10 core items was identified with further items identified as suitable for development as 'bolt-on' modules.

#### Conclusions
The apparent consensus on which items are important to trialists in a generic context suggests that a standardised instrument for core items is feasible, provided it is developed with the flexibility to add 'bolt-on' modules. Although this work was conducted in the context of RCTs within the UK, in principle, the items identified are generalizable to other jurisdictions and study designs.

### P266
## Identifying the optimal clinical pharmacological properties of potential future medicines using clinical trial simulation
Daniel Hill-McManus[1], Scott Marshall[2], Steven Lane[3], Elena Soto[2], Dyfrig Hughes[1]
[1]Bangor University; [2]Global Pharmacometrics Pfizer Ltd; [3]University of Liverpool
**Correspondence:** Daniel Hill-McManus
*Trials* 2017, **18(Suppl 1):**P266

#### Background
Modelling and simulation are increasingly being used to assist in planning and decision making during drug development. This includes clinical trial simulation which has been applied to optimise the design of drug trials; such as calculating sample sizes, determining the optimal dosing regimen or estimating the impact of protocol deviations. This requires constructing a mathematical representation of the trial processes and makes use of data on the population of interest and likely effects of the interventions being studied. These methods are typically applied to a specific compound in clinical development to support decision-making and trial design in subsequent phases of development. Another potential application of modelling and simulation, however, is in informing drug discovery by identifying the theoretical properties of a drug which would maximise the desired trial outcomes.

Knowledge of the clinical pharmacological properties of drugs in development evolves throughout the drug development process. Such properties include the absorption, distribution, metabolism and excretion of the drug (pharmacokinetics) as well as its effect on the body (pharmacodynamics), such as disease progression or biomarker activity. Pharmacometric models can be deployed to describe these processes based on available data and simulation can be used to both evaluate efficacy and safety in differing populations under different conditions to assess the impact of disease, background medication or adherence. These estimates can further be used as inputs to pharmacoeconomic models which compare the costs and benefits of the hypothetical drug to those of existing therapies. This has the added value of, going beyond efficacy, and identifying what properties are most likely to result in a drug being deemed cost-effective.

#### Methods
This study investigates the potential for using modelling and simulation to inform drug discovery and development using a case study of urate-lowering therapies (ULTs) used in the long term management of gout. Gout is characterised by a reliable biomarker, serum uric acid (UA) concentration, which is also used as a primary endpoint in clinical trials. Serum UA concentrations are well correlated with gout symptoms and can also be predicted using pharmacometric models. This case study focusses on estimating the desired clinical pharmacological properties of uricosuric drugs, which stimulate the renal excretion of UA thus lowering the serum concentrations. Successful uricosuric drugs must balance the risks resulting from the renal excretion of large amounts of UA against the benefits of reduced serum concentrations, while being forgiving to poor adherence which is common for ULTs. The linkage to economic modelling comparing the hypothetical drug with current standard ULT is used to provide estimates of cost-effectiveness.

#### Results
The results reveal the optimal combinations of clinical pharmacological properties for any future uricosuric medicine and therefore provide the target characteristics to drive future drug discovery. The results of the economic modelling show the probability that new drugs will be cost-effective for a given willingness-to-pay threshold based on the impact of these properties on the expected efficacy and safety. The findings could serve as a guide of the potential for future innovation in the area of uricosuric treatments for gout.

### P268
## Demographic and baseline characteristics of patients in china registry of hepatitis B (CR-HEPB)
Yuanyuan Kong[1], Lai Wei[2], Jinlin Hou[3], Zhongping Duan[4], Hui Zhuang[5], Hong You[1], Jidong Jia[1], the CR-hepb study group
[1]Beijing Friendship Hospital, Capital Medical University; National Clinical Research Center for Digestive Disease; [2]Peking University People's Hospital; [3]Nanfang Hospital, Southern Medical University; [4]Beijing Youan Hospital, Capital Medical University; [5]Peking University Health Science Center
**Correspondence:** Yuanyuan Kong
*Trials* 2017, **18(Suppl 1):**P268

#### Background & Aims
Chronic infection of hepatitis B virus (HBV) is the predominant cause of cirrhosis and hepatocellular carcinoma (HCC) in China. However, the real-world data on the clinical and treatment profile is still lacking. Therefore, we initiated a nation-wide, hospital-based registry system, China Registry of Hepatitis B (CR-HepB) in June 30, 2012.

#### Methods
This on-line registry system is owned by China Foundation for Hepatitis Prevention and Control and academically supported by Chinese Society of Hepatology. The criteria for inclusion to this registry was any adult patients with HBSAG-positivity for than 6 months, meeting the diagnostic criteria for various stages of chronic HBV infection and consent to receive follow-up per clinical practice guidelines (check HBVDNA, biochemistry, alpha fetoprotein and ultrasonography at least every 6 months). The decision on treatment was at discretion of the patients and the clinicians based on standard of care. The information system and database were developed, maintained and managed by an information technology service provider and professional statistical and methodological terms

**Results**

As of Oct 31, 2016, 38 tertiary or secondary hospitals across China had participated in this registry, and totally122,987 patients and 486,976 visits were recorded. Among them 61.9% were men; the average age at inclusion was 44.3 years, with 67.7% of them being between 30 and 60 years old. Approximately 6.7% were initially diagnosed with immune tolerance phase of HBV infection, 68.5% with chronic hepatitis B, 14% with cirrhosis (compensated 6.3%; decompensated 7.7%), 1.8% with HCC, and 9% with others (including acute-on-chronic failure and recovery phase of HBV infection). Forty seven percent of the patients were positive for HBEAG and 74.1% were positive for HBV DNA. Among 859 patients with liver biopsy the necroinflammation score G0-G4 were seen in 2.6%, 30.5%, 36.2%, 25.6% and 5.1%, respecdtively; and fibrosis stage S0-S4 were seen in 12.5%, 34.8%, 28.9%, 18.3%, and 5.5%, respectively. Totally, 33,533 (27.3%) patients had therapeutic information, among them 10.6% receiving conventional or pegylated interferon-alpha and 88.7% receiving nucleos(t)ide analogues (NAS). The split out of the NAS showed that entecavir accounted for 51.2%, lamivudine for 18.8%, adefovir for 16.1%, telbivudine for 12.5% and tenofovir (approved but reimbursable for HBV therapy until recently) for 1.4%.

**Conclusions**

In this largest registry for HBV patients in China mainland, middle-age men were predominant and less than half of all patients were HBEAG positive. NAS were the most commonly used but nearly half of the patients did not choose the recommended high potent and less resistant agents. Long-term follow-up and judicial evaluation of the antiviral efficacy and clinical outcomes would provide valuable information for decision-making in clinical and public health sector.

### P269

#### A systematic review identifying neonatal core data elements for clinical trials, and assessment of data completeness of identified data elements using the national neonatal research database (NNRD)

Sena Jawad, Daniel Gray, Neena Modi, Chris Gale
Imperial College London
**Correspondence:** Sena Jawad
*Trials* 2017, **18(Suppl 1):**P269

**Background**

Point-of-care registry trials extract data from existing Electronic Patient Records (EPR). Poor accuracy and completeness of EPR data are key barriers to the success of such trials. Neonatal care in the United Kingdom has a well-developed EPR database, the National Neonatal Research Database (NNRD). We hypothesised that within neonatal clinical trials there are a subset of common data items reported across important trials, and aimed to assess the completeness of these items in the NNRD.

**Methods**

We searched the four most cited general medical journals (New England Journal of Medicine, The Lancet, BMJ and The Journal of the American Medical Association) and extracted neonatal clinical trials from 2006 onwards with no restriction on the disease area or treatment type. The following data items were extracted from each identified paper by two independent reviewers: baseline characteristics, items used in stratification or minimisation, and items used to adjust the primary outcome. Data items were combined where clinically appropriate. NNRD data completeness was examined over the period January to June 2015 for infants <32 weeks gestational age in England, Scotland and Wales. Missing was defined as an empty field or an invalid value. This review was pre-registered (PROSPERO: CRD42016046138).

**Results**

We identified 46 studies involving 33,276 infants and 132 data items; 29 studies on pre-term babies, 12 on term babies and 5 studies where either age was unrestricted or undisclosed. Thirty seven trials (80%) were multicentre trials. Gestational age was the most commonly reported data item (96%). Sex (89%) and birth weight (89%) were also common items reported. Antenatal steroids were reported in 25 (86%) pre-term studies and 1 (8.3%) term study. Mother's ethnicity and multiple births were reported in 50% of all studies but the latter was presented in 72% (21) of pre-term studies and 8% (1) of term studies. Gestational age, the most reported data item, was complete for 99.3% of infants in the NNRD. With the exception of maternal ethnicity, none of the commonly reported data items were missing in more than 10% of babies in the NNRD.

**Discussion**

We show that there are a limited number of data elements which are common to recent influential neonatal clinical trials. Such essential data elements can be used to focus the measurement and improvement of data quality in preparation for point of care trials. The NNRD has low rates of missing data for these core data elements. This supports the use of routinely collected NNRD baseline data in UK neonatal clinical trials. Other key requirements are to establish national and international consensus for common outcomes for neonatal trials and assess the quality of corresponding NNRD data.

**Conclusion**

High impact neonatal clinical trials share core data elements. In the UK these data can be obtained reliably from the NNRD, a well-established EPR-derived national database, supporting its use for point-of-care registry trials.

### P270

#### Transition from paper to digital electrocardiograms (ECGs) after 30 years of follow-up in the DCCT/EDIC study

Jye-Yu Backlund[1], Barbara H. Braffett[1], Catherine L. Martin[2], Wanyu Hsu[1], Lisa Diminick[1], Susan Hensley[3], Charles Campbell[3], Yabing Li[3], Elsayed Z. Soliman[3], The DCCT/EDIC Research Group[1]
[1]George Washington University; [2]University of Michigan;
[3]Epidemiological Cardiology Research Center (EPICARE)
**Correspondence:** Jye-Yu Backlund
*Trials* 2017, **18(Suppl 1):**P270

**Background**

Type 1 diabetes is associated with cardiovascular disease (CVD) and the electrocardiogram (ECG) is the most accessible test for screening and detection of subclinical myocardial infarction and CVD. The Diabetes Control and Complications Trial (DCCT) and its follow-up study, the Epidemiology of Diabetes Interventions and Complications (EDIC) is currently following 1,214 of the 1,441 originally randomized participants (94% of the surviving). During the 30 years of follow-up, continual efforts were made to modernize systems and data processing to improve efficiency and reduce cost.

**Methods**

Participants were administered a resting 12-lead ECG at each annual visit during DCCT/EDIC. Paper tracings and accompanying paper tracking forms were sent from the 27 clinics to the Data Coordinating Center (DCC), then forwarded in monthly batches to the Central ECG Reading Center (CERC) for analysis. In 2012 the paper tracking form was replaced with direct entry into a proprietary data management system. In 2014, the study implemented the use of digital ECG recordings and transmission. Unlike paper ECGs, digital scans automatically provide hundreds of waveform measurements and can be stored indefinitely without the risk of deterioration. Digital ECGs are transmitted directly to the CERC via analogue phone lines. Site coordinators were centrally trained on the use of the new digital ECG machines and how to transmit the recorded ECGs electronically to the CERC.

**Results**

Implementation of digital ECG recordings substantially reduced the time between ECG acquisition and receipt by the CERC, with 49% of the ECG's transmitted on the day of the clinical visit (mean $14 \pm 18$ days). The time from ECG acquisition to generation of the ECG clinical report was reduced by 52 days ($120 \pm 39$ and $68 \pm 21$ days for paper and digital ECGs, respectively). Furthermore, the DCC received ECG results from the CERC 51 days sooner ($108 \pm 40$ days and $57 \pm 21$ days for paper and digital ECG, respectively). There was no reduction in ECG quality. The cost of purchasing new ECG machines capable of digital transmission was offset and balanced by the reduction in the cost of processing and reading paper ECG's over time.

**Conclusion**

Digital ECGs have simplified and enhanced the processing of ECGs between the reading center, coordinating center, and clinical sites by reducing the processing time by 43% with less effort and cost.

### P271

#### Building a generic drug supply management system for ECRF systems - Challenges and experience gained

Robbie Wilson[1], Sharon Kean[1], Elizabeth Douglas[2]
[1]Robertson Centre for Biostatistics; [2]R&D, NHS Greater Glasgow & Clyde
**Correspondence:** Robbie Wilson
*Trials* 2017, **18(Suppl 1):**P271

**Background**

As our Centre moves towards a standardised in-house platform for ECRF development we are taking steps to modify supporting processes (such as randomisation, pharmacovigilance and document management) into 'generic' components to build into this system. Another such component is the Drug Supply Management (DSM) tool suite which has been critical in previous systems to ensure allocation and monitoring of supplies across trial sites is accurate and efficient. Experience of developing custom versions of the DSM components has thrown up a number of challenges; these in turn have allowed refinement of functionality across multiple distinct system deployments. Identifying the 'best' features from each system will allow us to move towards the creation of a generic system.

**Methods**

Common DSM components we provide include batch management, order and receipt management (both manual and triggered), post-receipt status updates and summary reporting tools. The processes behind each component can vary slightly or significantly based on the logistical challenges identified when reviewing a trial protocol and after discussion with the trial management team and most importantly the lead pharmacist or their equivalent. Although the basic functionality appears to be the same the changes are usually identified 'under the hood' and we will look to identify the different types of changes that we have had to put in place from system-to-system. Developing systems like this as part of our Clinical Trials Unit (CTU) has allowed for gradual refinement of functionality. However, we have faced increasing challenges when integrating with third-party systems and working to an external set of Standard Operating Procedures. These differences will be considered and compared.

**Conclusion**

We will discuss the different scenarios faced when developing previous DSM systems including why we had to modify the standard platform and how we adapted it. We will also discuss how this experience can be used to produce a generic system that is suitable to adapt to different use cases as required. The difference between open-label and double-blind DSM systems will be considered as this may be the reason for two separate systems instead of just one. The feasibility of a 'truly adaptable' system will be considered after this discussion.

### P272

#### Building a new EDC in 2016

Jonathan Gibb, Sharon Kean
Robertson Centre for Biostatistics
**Correspondence:** Jonathan Gibb
*Trials* 2017, **18(Suppl 1):**P272

**Background**

Compared to most software development projects, clinical trial electronic data capture (EDC) systems have a very long shelf life. It is not uncommon for clinical trials to last ten years. Even when trials themselves may be comparatively short lived, the systems used to collect and collate the data must be usable and provide reproducibility potentially for decades after the trial has finished. This culture of long lived projects and gradual organic evolutionary change in software development has many advantages. Ideally best of breed features and solutions remain and weaker elements are replaced. However legacy artefacts are left over from all evolutionary approaches. In geography there are ox bow lakes, in human anatomy there is the tailbone and in jeans the small inner pocket was originally used to hold a pocket watch. In popular culture, the television show "Lost" featured a character that lived in an underground bunker and types a series of mysterious numbers into a computer every 108 minutes or there would be undisclosed "disastrous consequences". While a fictitious plot device, there are numerous similar occurrences in real business software and processes where the knowledge exchange process is simply - just enter a zero in that field, we don't know why, but it doesn't work if you don't. When faced with the large amount of expertise gathered from past EDC development in a CTU, the large number of processes involved - the reason for which may or may not be understood, the risk of mistaking valid important elements for legacy artefacts and combatting the phrase "that's how it's always been done", creating a new EDC system is difficult.

**Conclusion**

The authors will present the major design decisions faced during the creation of a new EDC, the new features introduced for the different staff roles involved with the EDC usage lifecycle, including: extensible programming language and platform support, internationalisation, multiple means of API access, online/offline versioning system.

### P273

#### The advantages of simple analytics in EPRO environments

Dionne Russell, Jonathan R. Gibb, Sharon Kean
Robertson Centre for Biostatistics
**Correspondence:** Dionne Russell
*Trials* 2017, **18(Suppl 1):**P273

**Background**

For many years Google have offered companies a range of tools to provide analytics on web sites. These tools can be used to track and report on where or when the visitor to the site is having a bad experience and does not complete a transaction or chooses to leave the site. Companies use these tools to identify problem pages and further enhance the users experience with a view to boosting sales and revenue. Clinical trials have become less reliant on paper based systems and more reliant on electronic data capture (EDC) and electronic patient reported outcomes (EPRO). This type of technology enables upfront validation of data, which in turn should provide a cleaner data flow and should lead to less data management queries. If however there is a problem with the design of the system it may result in either partial completion or non-completion this will lead to missing key data in any self-completion questionnaires in the study. This is particularly dangerous in EPRO scenarios where there is often less feedback from participants and there is usually the option to skip most questions. The problem of questionnaire abandonment in EPRO is somewhat similar to the problem of basket abandonment in ecommerce websites in that it can be difficult to know what has caused the abandonment and they both lead to significant loss.

**Methods**

To improve the user experience of EDC and EPRO and ultimately increase the completion rate, a range of existing approaches from mainstream web analytics and observational usability evaluations sessions were combined and embedded within the EDC and EPRO environments.

**Conclusion**

We will demonstrate the significant advantages this approach presents when combined and how it can be used to identify and resolve issues when they arise rather than discovering than facing incomplete datasets at the end of the collection period.

### P274

#### The paperless paradigm: transitioning to direct participant data entry in the search for diabetes in youth study

Julia Robertson[1], Leora Henkin[1], Ken Wilson[1], Jerry Barnes[1], John Hepler[1], Catherine Pihoker[2], Beth Loots[2], Amy K. Mottle[3], Lynne Wagenknecht[1], Ralph D'Agostino Jr.[1]
[1]Wake Forest School of Medicine; [2]Seattle Children's Research Institute; [3]University of North Carolina at Chapel Hill
**Correspondence: Julia** Robertson
*Trials* 2017, **18(Suppl 1):**P274

Web-based, direct data entry by research participant populations is becoming more common. In a long running research study, transitioning away from paper-based data entry provided opportunities and highlighted challenges.
SEARCH for Diabetes in Youth (SEARCH) is an on-going, large, national multi-center and population-based epidemiological study of youth with diabetes. Initiated in 2000, SEARCH was designed to estimate the prevalence, incidence and clinical presentation of diabetes in youth age < 20 years, by age, sex, race/ethnicity and diabetes type. More than 25,000 youth have been enumerated as part of the registry study; 3000 are enrolled in the longitudinal cohort study. These individuals represent diverse racial and ethnic backgrounds helping SEARCH determine the extent of diabetes in the community and its impact on different populations. SEARCH has 5 clinical centers located in South Carolina, Ohio, Colorado, California, and Washington. Funding is provided by the Centers for Disease and Prevention (CDC) and the National Institute of Diabetes and Digestive and Kidney Diseases (NIDDK).
The cohort study sample, followed longitudinally for 16 years, is characterized using a variety of age specific surveys and a physical exam in which complications and laboratory measures are assessed. For the present SEARCH visit, the study took the opportunity to broaden the method of data collection to allow participants the choice of using a web-based direct data entry approach or to have data collected on paper forms. To facilitate this transition, data entry forms were carefully evaluated, re-organized and updated to better accommodate direct data entry. The goals are to facilitate a positive end-user experience, ensure high quality data capture, increase response rates and decrease clinic staff burden. In addition, because of funding limitations, approximately half of the past participants were invited to participate only in the online surveys (e.g., were not offered an in person visit). To date, response rates are on track to meet study goals.
Some of the challenges of adding the option of participant direct data entry include creating an intuitive, seamless data entry flow, encouraging participants to answer all questions and to complete all modules, ensuring invalid or out of range answers are corrected and that the participant understands overall navigation and the meaning of individual questions and facilitating consistency in future analyses after questionnaire updates and re-organization. This poster will further explore the challenges encountered, both in planning and programmatic implementation, and will describe the advantages and outcomes both for clinic staff and study participants.

### P275

#### Using REDCAP's piping feature to aid in longitudinal data collection

Jennifer Talton, Phillip Summers, Kathryn Melius, Thomas A. Arcury
Wake Forest School of Medicine
**Correspondence:** Jennifer Talton
*Trials* 2017, **18(Suppl 1):**P275

REDCAP (Research Electronic Data Capture) is a secure, web-based application designed to support data capture for research studies and is free to non-profit organizations who join the redcap Consortium. Its capabilities include the building of custom databases and surveys (that supports online or offline data capture); it also has data management features, such as participant scheduling calendars. Projects which do not need highly specialized data collection tools or have the resources for programmer support to create such tools can greatly benefit from REDCAP's features, which are continually being advanced. Piping is a capability within redcap that allows inserting values previously entered by the user into a survey's text. This feature enables the generation of dynamic questions that appear to be customized for a specific individual.
The EPHIM (electronic Patient Health Information Management) Project is a research study with the aim of understanding the factors that facilitate and limit use of patient portal systems by older, lower-income adults. Documenting human-technology interaction is a key component of the project. To assess the participants' interactions with technology, we asked them about their e-mail use, and if they had any of the following in their home: stationary computer, laptop computer, tablet, smartphone, or internet. These items were to be asked in a baseline questionnaire and in six follow-up questionnaires spanning 12 months. While we wanted to follow any changes in the status of these items, given the age of the participants and relatively short time frame across visits, we decided it was unlikely there would be many changes in the technology available in participants' homes during the two months between interviews. Thus, in order to prevent discrepancies in responses across time we used a combination of the piping feature and branching logic within redcap to remind the participants how they answered previously. For example, if a participant answered that he/she had a laptop computer in his/her home in the baseline questionnaire, at the next visit the question would read, "When we spoke last time you indicated that you do have a laptop computer in your home. Is this still correct?" The response to this yes/no question was stored and used within the next follow-up visit questionnaire which would have the same wording ("When we spoke last time you indicated that you? Is this still correct?"). This feature was enabled using a series of conditional statement code similar to syntax used within Excel.
In its simplest form, the piping feature within redcap allows for a more personal correspondence with participants and increases efficiency in the data collection process. We have highlighted a more advanced example of the piping feature which, when used in combination with branching logic, can be used to customize data collection and help prevent discrepancies across time.

### P276

#### Using dedicated study laptops to enhance data quality, participant safety, and trial efficiency: experience from a large-scale, international, randomized clinical trial

Michelle Nunn, John Nolan, Simon Gilbert, Alex Baxter, Bob Goodenough, Mike Lay, Louise Bowman, Martin Landray
Clinical Trial Service Unit (CTSU) and MRC Population Health Research Unit, Nuffield Department of Population Health, University of Oxford
**Correspondence:** Michelle Nunn
*Trials* 2017, **18(Suppl 1):**P276

##### Background

The REVEAL study is a streamlined randomized controlled trial (RCT) investigating the effects of adding the CETP inhibitor anacetrapib to LDL-lowering treatment with atorvastatin among 30 000 study participants at high risk of vascular disease. It is coordinated by the CTSU at the University of Oxford (the trial Sponsor) with 6 Regional Coordinating Centres responsible for the conduct of the trial in 431 sites in 10 countries. Laptops are provided to all sites so that participant visit data can be captured directly into the electronic Case Report Form (ECRF) using software specifically designed for this purpose by CTSU. The laptop database therefore contains the source data.

##### Methods

Features of the laptop system used include: Consistency in laptop setup by utilising a Windows 7 image which also allows for regional variations where required. Remote central management of laptops by CTSU, including user accounts and passwords plus distribution of software updates. Timely, secure and regular transfer of data between the laptops and the designated FTP & HTTPS servers via a reliable internet connection. Robust laptop & data security achieved through a combination of data encryption, whole-disk-encryption, passwords, 'Locked down' user accounts and staff training. Participant identifiable data is retained on the laptop but only transferred to CTSU if permitted by local data protection laws.

**Discussion**

Using laptops in REVEAL offers a highly effective and user-friendly method of collecting good quality participant data with significant advantages over paper-based systems. Careful scripting of the user interface enhances compliance with the study protocol and procedures, and prompts any necessary actions related to participant safety. Data entry (which can be done offline) directly into an ECRF provides immediate validation of data and a full audit trail, whilst site investigators retain access to their participants? Data held locally and securely both during and after the trial. The use of a 'thick client' rather than a web-browser provides resilience against internet outages whilst enabling timely data management across multiple countries and time zones. Potential drawbacks include reliance on a good internet connection for regular data transfer (ideally at least daily), central study databases not being updated in real time (although this is much less of a problem than systems that rely on transcription of data from paper forms), and restrictions on moving participant identifiable data out of some countries. In addition the provision of laptops can be expensive and resource intense requiring remote support and maintenance over many years.

**Conclusion**

In comparison to paper-based clinical trials, the use of carefully programmed and managed study laptops has significant benefits for participant safety, data quality and regulatory compliance. However, it is probable that in the future web-browser based systems (including tablet and smartphone implementations) will become the norm, with advantages in terms of cost, convenience, familiarity, and usability. However, the lessons learned from delivering a large-scale, laptop-based, direct data entry system will be valuable if the potential of these newer approaches is to be realised in future clinical trials.

### P277

#### Tracking data requests from initiation to publication through the use of a web-enabled database system

Michael Ham[1], Elizabeth M. McClure[1], Kevin A. Wilson[1], Edward A. Liechty[2], Norman Goco[1], Lori Schwarze[1], Carolyn M. Petrie Huitema[1]
[1]RTI International; [2]Indiana University
**Correspondence:** Michael Ham
*Trials* 2017, **18(Suppl 1):**P277

The Global Network's (GN) clinical trials produce rich data sets to inform investigators about treatments to prevent, diagnose, or treat human diseases. From these datasets, the clinical investigators publish results from their preplanned investigation to inform colleagues of the outcomes of the clinical trial. These same datasets can be used for secondary analyses to leverage the information available. All of these publications enrich the understanding of a disease state and possible therapies as well as serve as measurable deliverables, demonstrating productivity to the funding source. Therefore, we needed a system to track 1) data requests (for secondary analyses), 2) pre-publication development (includes all primary and secondary reports using a clinical trial dataset) and 3) post-publication tracking (citation counts, journal impact factor and author h-index).

Since the GN is a collaborative network, comprised of several sites located across the U.S and in other countries, the tracking system had to be centrally accessible. To accomplish this, we chose to allow access through our website. We developed this tracking tool such that the investigators could submit concepts, requesting data from a completed clinical trial, in an easy and trackable way. By collecting additional information about the concept, all stakeholders could access the information and understand the current status of the progress. The system was also developed to track primary manuscript development as the first dissemination of a clinical trial as a journal publication. By inputting information about the process, the status of the manuscript is transparent. Finally, the system needed to provide an easy way for investigators and other staff to show the effects that their completed clinical research has had on the overall scientific community.

These challenges were addressed by looking at a number of offline tracking systems that had failed to meet the needs of various clinical networks, and finding ways to improve upon them. An extensible.NET module was developed to store and display all relevant data for publications tracking. Web services were employed to connect the system to popular publication data stores such as The United States National Library of Medicine's PubMed database, Thomson Reuters's Web of Science database, and the International DOI Foundation's digital object identifier (DOI) to retrieve and store accurate information about publications at any point in the creation process. The presentation will discuss the different technical challenges of this approach and how they were resolved. Pros and cons of the approach will also be discussed along with methods used to report information out of the system.

### P278

#### Incorporating electronic health records data into efficient trial interventions – cluster randomised trial to reduce antibiotic prescribing in primary care

Dorota Juszczyk, Judith Charlton, Martin C. Gulliford, the REDUCE Trial Research Group
King's College London
**Correspondence:** Dorota Juszczyk
*Trials* 2017, **18(Suppl 1):**P278

**Background**

Antibiotic resistance is a growing problem that transcends national boundaries. 80% of all antibiotic used in the UK are prescribed within primary care, and up to 50% of prescriptions may be unnecessary. Interventions are needed that can be delivered to prescribers at low cost.

**Objectives**

This study utilises electronic health record data (EHR) on antibiotic prescribing for specific indications to provide feedback to prescribers. This intervention is being evaluated in a cluster randomised trial.

**Study setting**

The study is currently being conducted in the Clinical Practice Research Datalink (CPRD).

**Methods and results**

There were 80 CPRD general practices randomised. The trial interventions include a webinar and decision support tools that are delivered into general practice systems. In addition, intervention trial arm practices are sent monthly-updated reports on their prescribing of antibiotics for respiratory tract infections. During the 12 month intervention period, electronic health records data are analysed from each month's CPRD release to provide detailed feedback on the number of RTI consultations, number of antibiotic prescriptions and proportion of consultations with antibiotics prescribed. Prescribing-report templates were designed through qualitative research with non-trial practices. An R program was written to extract, analyse and summarise electronic health records data for intervention trial arm practices. The "reporters" package in R was used to create personalised "Pdf" reports, based on the templates, for each practice.

**Conclusions**

As well as informing the design of the cluster trial and outcome evaluation, electronic health records were used as a key component of the trial intervention. The automated system for writing feedback reports was time-consuming to establish, but then worked efficiently. The most time consuming elements of the report production were those that could not be automated, such as updating the meta-files and adjusting the template design and content.

Trial Registration and Funding: NIHR HTA Trial registration: ISRCTN95232781

### P279

#### Using optical character recognition for processing paper forms in a large randomised clinical trial

Aleksandra Murawska, Jolyon Cox, Jane Booth, Jill Barton, Kevin Murphy, Youcef Mostefaiv, Michael Lay, Louise Bowman, Jane Armitage
Clinical Trial Service Unit (CTSU) and MRC Population Health Research Unit
**Correspondence:** Aleksandra Murawska
*Trials* 2017, **18(Suppl 1):**P279

**Background**
ASCEND is a randomised 2x2 factorial study of aspirin versus placebo, and of omega-3 fatty acid supplementation versus placebo, for primary prevention of cardiovascular events in people with diabetes. The study used a mail-based approach to identify and randomise 15,000 people with diabetes. The project is now approaching its final follow-up stage. In the course of the study, the vast majority of data collection was done via paper designed to be processed with Optical Character Recognition software. In previous paper based studies, double data entry was used, which meant sending paper forms to a commercial company for data entry and finding and resolving any data or keying discrepancies in-house (often with the necessity to see the paper form). For ASCEND, a more streamlined and cost effective approach has been developed.
This presentation will discuss the methodology, costs and benefits of this approach.
**Methods**
A third party OCR software, FORMS, from Readsoft (now Lexmark) was identified as the best tool to scan, review and save form content for further use in the study. The software gives a rich selection of ways the process can be customised. An option to review different parts of the form by users with different roles was required, e.g. Lists of medication or medical events to be reviewed and coded by medical staff, the rest of the form to be reviewed by administration staff. Using the FORMS' API, extra validations and background calculations were performed and bespoke dialogues were added. The dialogues made it possible to save handwritten medical events and medications as Read codes. Another required outcome was a digital archive of form images with links to relevant participants for viewing at any later point (instead of pulling a form out of the paper archive).
Study paper forms were designed to fit FORMS requirements and printed in a professional printing house using specific Pantone colours, as we wanted to drop-out some elements during scanning. Once the form stationery was available, we could start a long process of implementation of our own validation rules into the, unfamiliar at that time, FORMS processing flow. Later on frequent maintenance work was needed. The trial was extended in length and size and additional stocks of form stationery had to be ordered. The set-up had to be tuned each time a new printout of a known form was to be used.
**Results**
In total, about 340,000 forms of seven types (with multiple printouts in each type) were processed by a team not larger than 4 administrative and 2 medical staff and this task was only one of their many duties. Coding of medication and medical events reported was done from the form image, while with a double data entry system it was possible only after the keyed data was transferred into the database.
**Conclusions**
The choice of the OCR system to process the ASCEND form over the traditionally used double data entry proved to be more efficient, although the path to a working system was strenuous and full of pitfalls.

### P280

#### A toolkit for patient and public involvement in a clinical trials unit: an update & next steps

Heather Bagley[1], Paula Williamson[1], Carrol Gamble[1], Nicola Harman[1], Hannah Short[1], Priya Francis[1], Helen Hickey[1], Bridget Young[1], Kerry Woolfall[1], Delia Muir[2]
[1]University of Liverpool; [2]University of Leeds
**Correspondence:** Heather Bagley
*Trials* 2017, **18(Suppl 1):**P280

This abstract is not included here as it has already been published.

### P281

#### Integration of patients, caregivers and healthcare providers into a hypertension management research priorities process

Simon Bacon[1], Nadia Khan[2], Hypertension Canada Priorities Setting Process Group[3]
[1]Concordia University & CIUSSS-NIM HSCM; [2]University of British Columbia; [3]Hypertension Canada
**Correspondence:** Simon Bacon
*Trials* 2017, **18(Suppl 1):**P281

**Background**
Despite the tremendous strides in hypertension management over the last 4 decades, blood pressure remains poorly controlled in 35-50% of patients with hypertension. This research agenda has generally been led by the scientific community and pharmaceutical industry. Integrating patients and other key stakeholders as co-builders in the development of research priorities may offer a new strategy to increase the direct relevance and applicability of research to ultimately close the current gaps in hypertension management.
**Objective**
We aimed to identify the 10 most important research priorities for hypertension management across all stakeholder groups: patients, caregivers and healthcare providers (family physicians, nurses, pharmacists and dieticians).
**Methods**
Using the James Lind Alliance approach, a national web based survey asked patients, care givers and health care providers to submit their unanswered questions on hypertension management. This questionnaire was distributed through a variety of partner organisations to try and reach as wide a population as possible. Questions already answered from randomized controlled trial evidence were removed. A second questionnaire containing 42 distilled unique questions were distributed in a 2nd round for feedback and ranking. An in-person priority setting meeting of patient, caregivers and healthcare providers then ranked the final top 10 research priorities.
**Results**
There were 386 respondents who submitted 598 questions (after exclusions). The majority of respondents were patients or caregivers (78%). In addition, 29% lived in rural areas, 78% were aged 50–80 years and 75% were women. The 598 questions were distilled down to 42 unique questions and from this list, the top 10 research questions prioritized included: determining the combinations of healthy lifestyle modifications to reduce the need for antihypertensive medications, stress management interventions, evaluating treatment strategies based on out-of-office blood pressure compared with conventional (office) blood pressure; education tools and technologies to improve patient motivation and health behavior change, management strategies for ethnic groups, evaluating natural and alternative treatments and the optimal role of different healthcare providers and caregivers in supporting patients with hypertension.
**Conclusions**
These research priorities can be used to guide researchers and funding bodies on hypertension management research considered most relevant to patients, caregivers and healthcare providers. Given the challenging nature of these priorities, consideration of what kinds of innovative clinical trial methodologies to answer these questions is also needed.

### P282

#### Building a relationship with industry - An academic's experience working with Zimmer Biomet

Cushla Cooper[1], Rachel Dorman[1], David Beard[1], Jing Xie[2], Andrew Price[1]
[1]University of Oxford; [2]Zimmer Biomet
**Correspondence:** Cushla Cooper
*Trials* 2017, **18(Suppl 1):**P282

#### Introduction
Investigator Initiated Trials (IITs) are academic sponsored trials funded by industry. IITs expand product knowledge and are often seen to provide more robust data in delivering evidence based practice. They provide evidence for clinicians, funders and industries about what happens in the real world (Suvarna, 2012) and can be used to show proof of principle. Our academic unit is currently collaborating with industry (Zimmer Biomet) on the All Ligaments Left in Knee Arthroplasty Trial (ALLIKAT). We report the challenges and benefits of the collaboration in a device based trial and how a mutually acceptable study is being delivered.
### Background
ALLIKAT compares the clinical and patient reported outcomes of the new bi-cruciate retaining knee system (Vanguard XP) to the current single cruciate-retaining knee system (Vanguard CR). It is sponsored and managed by the Surgical Intervention Trials Unit (SITU) at the University of Oxford and funded by Zimmer-Biomet. As a key stakeholder in the trial, the needs of Zimmer Biomet, with its product-based approach, had to be carefully considered alongside an academic assurance of protocol integrity (Goldenberg et al., 2011). Challenges identified in our industry collaboration include:

– Ensuring compatibility of research questions.
– Developing a mutually acceptable protocol.
– Identifying and being explicit about potential bias.
– Developing solutions for potential bias.
– Keeping abreast of market forces.
– Ensuring the study is patient-focused.
– Agreeing resource allocation and ownership for data collection

#### Methods
Regular meetings were arranged with Zimmer Biomet, with updates on progress and feedback on issues could be discussed. The trial was developed as a study providing evidence for current practice variations. Previously within industry, surgical implant-based research has been limited and undertaken essentially for marketing purposes. ALLIKAT moved the focus to providing robust evidence for the use of new implants. We also ensured the protocol supported the funder's objective to comply with the Beyond Compliance programme, which was set up by the BOA (British Orthopaedic Association) to monitor safety of new products.
Working together allowed us to manage the inevitable product ?A3B2 show $132#?>design modifications, so that the study was not jeopardised or over-shadowed by market pressures from within the company. As a pragmatic study within the NHS it also needed to be patient-focused. This was achieved by utilising patient reported outcome measures, rather than subjective clinical reports and ensuring patients were not referred to as 'subjects' In study related documents.
### Discussion
A multi-disciplinary collaborative team have been involved in the design and set-up of the study and have produced a protocol that considers academic, patient and industry needs, which is practicable and will provide evidence for proof of principle of a new marketable device to be used in clinical practice. Maintaining a patient focus and promoting evidence-based health care has been key to working with industry and the open communication dialogue has been helpful in achieving objectives. There has been a corollary advantage in the increased network links with industry, orthopaedic consultants and NHS Trusts around the UK and a greater understanding of the needs of these different parties.

### P283
## Using photographic images to aid wound assessment within a randomised controlled trial of standard wound management versus negative pressure wound therapy: UK WOLLF trial
Julie Bruce[1], Amy Verdun[2], Sonia Davis[3], Juul Achten[3], Nick Parsons[3], Susie Hennings[3], Matt Costa[4]
[1]University of Warwick; [2]University Hospitals Coventry & Warwickshire; [3]Warwick Clinical Trials Unit; [4]Nuffield Orthopaedic Centre, University of Oxford
**Correspondence:** Julie Bruce
*Trials* 2017, **18(Suppl 1):**P283

#### Background
Patients who sustain open lower limb fractures are at high risk of postoperative surgical site infection (SSI); rates of up to 27% are reported for deep SSI. The type of dressing applied after initial debridement may influence postoperative wound infection and healing. The aim of WOLLF is to investigate the effectiveness and cost-effectiveness of standard postoperative wound dressings with negative pressure wound therapy in adult patients undergoing surgical management of open lower limb fracture. We supplemented wound assessment data collection with digital photographic images of surgical wounds at six weeks postoperatively to investigate the feasibility and utility of this methodology to aid the diagnosis of wound healing and SSI.
### Methods
Setting: This multicentre pragmatic 2-arm randomised controlled trial (RCT) was conducted in 24 trauma centres in the UK. Participants: All adult patients presenting with an open lower limb fracture, with a Gustilo and Anderson (G&A) grade 2/3, were eligible for inclusion. Initial wound management in Emergency Departments involved removal of gross contamination, followed by surgical debridement under anesthesia, where contaminated tissue is removed and the open fracture is washed out. The fracture is then immobilized using internal or external fixation. Interventions: Participants were then randomised to treatment allocation at the end of surgery, to either usual wound care or to negative pressure wound therapy.
### Outcomes
The primary outcome is the Disability Rating Index (DRI) score at one year. Secondary outcomes include incidence of deep infection of the limb, quality of life and postoperative complications. Superficial and Deep SSI was assessed at six weeks after surgical repair of open fracture. Research associates (ras) observed wounds at six weeks and completed data collection forms. Clinical and patient-reported criteria were used, based upon the Centers for Disease Control diagnostic criteria (CDC, 2015). Photography: Digital cameras were used to capture a one or more images of trial wounds at six weeks postoperatively; no upper limit on number of images was applied. A protocol was developed for ras. Photographs were taken in a variety of clinical settings whereby light conditions were optimised to reduce glare. A 15-cm ruler with clear millimeter divisions was placed next to the wound, with participant identification number. Images were returned to and stored by Warwick CTU.
### Results
A total of 1515 images were obtained from 358 participants (mean 4.2 images per participant). Two assessors independently judged photographs; an experienced tissue viability nurse and senior researcher with clinical background. A third independent clinician acted as final arbiter where there was lack of agreement. Assessors were blinded to treatment allocation. Wounds were judged for healing and visual evidence of infection, without knowledge of clinical and patient-reported criteria recorded by RAs. We report inter-rater and intra-rater reliability (kappa values) for agreement between clinical assessors and compare rates of healing and SSI with and without photographic data. This poster will present our experience of the feasibility of supplementing standard data collection methods by the addition of a 2-dimensional image to aid the accurate diagnosis of postoperative infection in a large pragmatic trial.

### P284
## Dementia outcomes after addition of proxy-based assessments for deceased or proxy-dependent participants
Sarah Gaussoin, Dan P. Beavers, Mark A. Espeland, Katelyn R. Garcia, Beverly M. Snively, Sally A. Shumaker
[1]Wake Forest School of Medicine
**Correspondence:** Sarah Gaussoin
*Trials* 2017, **18(Suppl 1):**P284

This abstract is not included here as it has already been published.

### P285

#### Intra and inter-observer agreement in the interpretation of preterm neonatal cranial ultrasound scans

Lucy Bradshaw[1], Jon Dorling[2], Lelia Duley[3], Lindsay Armstrong-Buisseret[3], Joe Fawke[4], Bernard Schoonakker[5], Eleanor Mitchell[3], Rob Dineen[6]
[1]University of Nottingham; [2]Early Life Research Group, University of Nottingham; [3]Nottingham Clinical Trials Unit, University of Nottingham; [4]Leicester Neonatal Service, University Hospitals Leicester NHS Trust; [5]Nottingham Neonatal Service, Nottingham University NHS Trust; [6]Division of Clinical Neuroscience, University of Nottingham
**Correspondence:** Lucy Bradshaw
*Trials* 2017, **18(Suppl 1):**P285

**Background**
Variation in ascertainment of diagnosis based on radiological imaging has implications for randomised trials that report outcome based on radiological diagnosis. This variation is particularly important for neonatal trials where diagnosis of intraventricular haemorrhage (IVH) and its severity based on cranial ultrasound scan are important outcomes.
As part of the preparation for independent adjudication of cranial ultrasound scans in a perinatal trial (Cord pilot trial), we measured the intra- and inter-observer agreement of interpretation of cranial ultrasound scans by expert adjudicators.
**Methods**
Eight experienced neonatologists or radiologists with expertise in cranial ultrasound scans completed standardised training from a paediatric neuroradiologist. To measure intra- and inter-observer agreement each trained assessor rated the same 64 anonymised cranial ultrasound scan images; comprising two sets of 32 scans. The second set was a duplicate of the first with order and anonymisation code changed. These scans were prepared by the neuroradiologist. Six different diagnoses (IVH grades 1 to 4, periventricular leukimalacia (PVL) and ventriculomegaly) were represented at least five times in the 32 scans, with some scans showing more than one target pathology and at least five with no abnormality. Assessors were unaware of the duplicates. Results are presented for any IVH, mild/moderate IVH (grade 1 or 2), severe IVH (grade 3 or 4), PVL and ventriculomegaly. The multiple rater Kappa statistic was used to assess inter observer agreement. The intra-observer agreement was assessed by calculating the intra-observer Kappa statistic between the two sets of scans for each adjudicator.
**Results**
Inter-observer agreement for severe IVH was substantial (multiple rater Kappa 0.62), fair for mild/moderate IVH and all IVH (multiple rater Kappa 0.26 and 0.37 respectively), moderate for ventriculomegaly (0.59) and poor for periventricular leukomalacia (multiple rater Kappa 0.11). Intra observer agreement was substantial for severe IVH (mean Kappa 0.74) and ventriculomegaly (mean Kappa 0.78), fair to moderate for mild/moderate IVH and all IVH (mean Kappa 0.44 and 0.53 respectively) and moderate for PVL (mean Kappa 0.46).
**Conclusions**
This study has shown that there is considerable variation in the classification of scan results between individual assessors and between the same assessor on repeated review. This suggests that for perinatal and neonatal trials it is important to use standardised criteria and centralised adjudication with a consensus process for interpreting cranial ultrasound scans. This has implications for other trials where radiological images are used to assess outcome.

### P286

#### Patterns of adverse events after cardiac surgery

Rachel Maishman[1], Barnaby C. Reeves[1], Gianni D. Angelini[2], Chris A. Rogers[1]
[1]University of Bristol Clinical Trials and Evaluation Unit; [2]Bristol Heart Institute, University of Bristol
**Correspondence:** Rachel Maishman
*Trials* 2017, **18(Suppl 1):**P286

**Background**
After cardiac surgery, post-operative adverse events are common and many patients experience several events. Some are frequent and less severe, others are rare but serious. Most trials are under-powered to detect differences in specific adverse events and event rates are often described but not formally compared. We have examined the timing and ordering of adverse events to inform the development of a composite objective measure of "recovery" for use in cardiac surgery trials.
**Methods**
A cohort of 1102 patients having coronary artery bypass grafting in four trials was assembled. Data on 11 adverse events, recorded consistently across trials, were analysed. For each event, the timing was compared between patients with and without other adverse events. For the subgroup with multiple events, the timing of other events experienced relative to a target event (before, on the same day as, or after the target event; each event considered as the target in turn) was summarised. Sequential pattern mining techniques were used to identify common sequences of events in patients with at least two adverse events.
**Results**
Common adverse events were supraventricular tachycardia/atrial fibrillation (SVT/AF) (341/1102, 31%) and suspected infections (312/1102, 28%); half of those with both complications (62/124) had the SVT/AF prior to infection. Reoperation occurred less frequently (56/1102, 5%), and typically occurred alongside other events (44/56, 79%). Where reoperation and reintubation both occurred, they typically happened on the same day whereas, when reoperation and suspected infection both occurred, reoperation usually preceded the infection. Trends were less apparent for rarer complications, but patients who had both a myocardial infarction (MI) and SVT/AF (10/13 of those who had an MI) all had the MI first.
Most events occurred early during the post-operative stay, and timings were similar between patients with one or multiple events. Exceptions to this were reoperation, which happened later in patients with multiple events (although most reoperations were still within one day of surgery) and gastrointestinal complications (which tended to occur later in patients with multiple events). Unlike other events, tracheostomy happened at any time during the post-operative stay but, when this occurred, it was typically the final event reported.
Twelve frequently occurring "event sequences" were identified, two of which included three events. 5% of patients with two or more events had SVT/AF and suspected infections following a reoperation; 25% of patients who needed reoperation went on to have SVT/AF and suspected infections. Also, suspected infections and reintubation were followed by a tracheostomy in 6% of after.
**Discussion**
The collection of comprehensive adverse event data across trials has allowed us to identify temporal relationships between different events. Some patterns were expected while others were less so. Some events are complications (e.g. MI), while others represent actions taken as a result of complication(s) occurring (e.g. Reoperation or reintubation). Working with clinicians, we are using these observations to inform discussions about which events to include in a composite outcome describing "post-operative course" and the relative importance of events.

### P287

#### Development of the standardising measures in arm rehabilitation trials for stroke (SMART) toolbox (work in progress)

Julie Duncan Millar[1], Myzoon Ali[1], Frederike van Wijck[2], Alex Pollock[1]
[1]Nursing Midwifery and Allied Health Professions Research Unit, Glasgow Caledonian University; [2]School of Health and Life Sciences, Glasgow Caledonian University
**Correspondence:** Julie Duncan Millar
*Trials* 2017, **18(Suppl 1):**P287

**Background**
Stroke is the leading cause of complex disability worldwide and up to 77% of stroke survivors experience impairments in arm function. Rehabilitation of arm function is a research priority for stroke survivors, carers and healthcare professionals. Randomised controlled trials (RCTs) of arm rehabilitation measure numerous outcomes (e.g. Strength, pain, ability to use arm) hindering comparisons and synthesis of trial data for efficacy analyses to inform clinical practice. However, arm function is a complex concept and a variety of outcomes and measurement tools may be warranted. Therefore, we aim to develop consensus recommendations on a toolbox of key outcome measures for use in arm rehabilitation RCTs.

**Objectives**

Describe current outcome measures used in arm rehabilitation RCTs and their psychometric properties. Identify outcomes important to stroke survivors with arm function problems, their carers and healthcare professionals. Produce final consensus recommendations to support selection of outcome measures for use in future arm rehabilitation RCTs.

**Method**

Phase 1: systematically explore trial data within a Cochrane Overview of arm rehabilitation RCTs, extracting data on assessment tool use. Tools must be clearly defined and reproducible to be considered as an outcome measure in phase 2 and 3.

Phase 2: using nominal group technique (NGT) identify and agree on outcomes relevant to life after stroke with arm impairment. Eight NGTs will be undertaken with stroke survivors and carers, and eight NGTs with healthcare professionals experienced in arm function rehabilitation. This will be supplemented by eight semi-structured interviews with stroke survivors and carers. Data will be analysed using content analysis. Outcome measures identified (Phase 1) will be linked with outcomes from Phase 2 followed by systematic exploration of outcome measures psychometric properties.

Phase 3: edelphi to achieve consensus amongst stroke arm rehabilitation researchers on important and feasible outcome measures from phase 2. A final consensus meeting with stakeholders (stroke survivors, carers, researchers, trialists, and healthcare professionals) will determine which outcome measures will be recommended as part of the SMART toolbox.

**Results**

Phase 1: We extracted data from 254 RCTs; 208 assessment tools were identified of which 146 met the criteria of reproducible outcome measure. The Fugl-Meyer (arm function section) was used most frequently (79/254 (31%) RCTs). 120/208 (58%) outcome measures were only used in one RCT.

Phase 2: 43 stroke survivors and carers, and 58 health professionals participated in the NGT sessions. Ten stroke survivors and carers participated in eight interviews. Data analysis will be completed by January 2017.

**Conclusions**

Phase 1 highlighted wide variation and lack of consistency in use of arm function outcome measures in RCTs. Consensus recommendations that account for psychometric properties, and the perspective of stroke survivors, carers, and healthcare professionals, will enable valid, reliable and meaningful measurement in future RCTs of arm rehabilitation. Therefore, recommendations for priority outcome measures that measure important outcomes are warranted. By agreeing on a toolbox of key outcome measures for inclusion, subsequent RCTs' outputs will enhance comparability of RCT results and facilitate comprehensive meta-analyses of the effectiveness of interventions.

### P288

#### Using proxy responses from carers to collect quality of life data on patients with brain metastases: an analysis from the UK MRC quartz trial

Matthew Nankivell[1], Paula Mulvenna[2], Rachael Barton[3], Corinne Faivre-Finn[4], Paula Wilson[5], Elaine McColl[6], Barbara Moore[7], Iona Brisbane[8], David Ardron[9], Richard Stephens[10]

[1]MRC Clinical Trials Unit at UCL AND MRC London Hub for Trials Methodology Research; [2]Northern Centre for Cancer Care; [3]Queen's Centre for Oncology and Haematology; [4]Institute of Cancer Sciences; [5]Bristol Haematology and Oncology Centre; [6]Newcastle Clinical Trials Unit and Institute of Health and Society; [7]Wales Cancer Research Network; [8]The Beatson West of Scotland Cancer Centre; [9]Patient representative; [10]MRC Clinical Trials Unit at UCL (retired)

**Correspondence:** Matthew Nankivell

*Trials* 2017, **18(Suppl 1):**P288

**Background**

Clinical trials requiring patient reported data involving patients with multiple symptoms and/or a poor prognosis are often considered challenging. There is concern about over-burdening trial participants, either through more regular follow-up or by using longer questionnaires. One possible solution is to obtain data about the patient indirectly by asking their carer.

The QUARTZ trial assessed the use of whole brain radiotherapy (WBRT) in patients with inoperable brain metastases from non-small cell lung cancer. This is a very poor prognosis group, and patients can experience rapid changes in condition, which necessitated frequent data collection. As the trial focused on quality of life, patients were asked to complete the EQ-5D questionnaire on a weekly basis. At the same time, their carer was asked to complete the same questionnaire from the point of view of the patient, so that the potential use of proxy scores could be assessed.

**Methods**

QUARTZ randomised 538 patients to receive either WBRT, or supportive care alone, with 407 carers also agreeing to participate. Here we compare the baseline responses to the EQ-5D-3 L questionnaire of the patients and carers. The trial's primary outcome measure of quality adjusted life years (QALYs) was also calculated separately from patient and carer data, and the results compared.

**Results**

Overall levels of agreement between patient and carer responses to the EQ-5D at baseline were 82% for mobility, 79% for self-care, 71% for usual activities, 78% for pain/discomfort, and 66% for anxiety/depression. For anxiety/depression, carers reported more problems than patients in 25% of cases, with 9% reporting fewer problems. For the other questions carers reported more problems as often as they reported fewer: mobility 9% vs 8%; self-care 11% vs 11%; usual activities 15% vs 15%; pain/discomfort 13% vs 9%.

QALYs were calculated for the 397 patients where both patient and carer data were available. The average QALY was slightly higher using patient data (45.3 days) than carer data (39.0 days). When assessing the treatment effect, the difference in average QALY (95% CI) was 3.2 days (−13.1, 7.4) when calculated from patient responses, and 5.3 days (−15.4, 3.9) from carer responses.

**Conclusions**

The level of agreement between patients and carers was reasonably high on most questions. The agreement was lowest for the question about anxiety and depression, with carers tending to report more problems compared to the patient's own assessment. The level of agreement seen means it may be reasonable to use the carer response in some situations where it is not appropriate to ask the patient directly. Caution is advised though as even for the question on mobility there was disagreement in 18% of cases. The difference in the analysis of the trial's primary endpoint was minor and did not change the main conclusion of the study. Therefore within the confines of a clinical trial, it could be appropriate to use these proxy assessments to assess treatment effects. QUARTZ is one of few trials in this setting, and further studies looking at the use of proxy responses in poor prognosis populations are warranted.

### P289

#### Reaching a consensus on causes of pain: use of an expert independent panel to determine a reference standard

Jane Daniels[1], Konstantinos Tryposkiadis[1], Lee Middleton[1], Lee Priest[1], Khalid Khan[2], Jonathan Deeks[1]

[1]University of Birmingham; [2]Queen Mary University of London

**Correspondence:** Jane Daniels

*Trials* 2017, **18(Suppl 1):**P289

**Background**

In a test evaluation study, where there are a number of target conditions to be considered, not all of which have a perfect reference standard, there is a risk of partial or differential verification of the underlying causes, with the inherent bias. One approach is to use an expert independent panel (EIP) to determine the presence or absence of the target condition based on several sources of information, ideally using a predetermined algorithm.

**Case study**

The MEDAL study asked whether magnetic resonance imaging (MRI) could replace or triage the use of laparoscopy in establishing gynaecological diagnosis among women with chronic pelvic pain (CPP). The primary analysis assessed the accuracy of the MRI for identifying the condition(s) causing CPP, using an EIP. The panel consisted of 15 Consultant Gynaecologists, not involved in the study recruitment, with each meeting involving three members. The reference diagnosis was made in two stages. 1. Using patient history and reported symptoms, clinical examination, ultrasound, laparoscopy and follow-up 2. As above with MRI scan report For both stages, each member individually recorded the condition(s) for which they were >50% certain were the cause of pain, prior to a group discussion to achieve a final consensus diagnosis for the reference standard. To assess the reliability of the EIP diagnoses we considered the agreement between the three individual ratings, using Kappa, made by the panel members prior to any group discussion. We faced several challenges: 1. The quantity of information to assimilate and use of summary or complete data 2. Eliciting a binary response, when there are degrees of uncertainty 3. Multiple potential causes of pain which are not directly correlated with the extent of pathology observed 4. Avoiding incorporation bias from knowledge of MRI results 5. Laparoscopy cannot identify some target conditions, only MRI can 6. Potential dual purpose of laparoscopy as diagnostic and therapeutic.

**Results**

The EIP could produce the same diagnosis with summary data as for complete data, although the EIP preferred the complete data, or used in conjunction with the summary report, where cases were considered complex. Sometimes it was felt necessary to select two or more conditions where they could equally be the cause of pain, although the EIP member was required to be at least 50% certain. The panel were in excellent agreement in identifying deep infiltrating endometriosis and endometrioma of the ovary as causes of CPP, and in good agreement for superficial peritoneal endometriosis and adenomyosis when the MRI reports were used in the reference standard. Lower levels of agreement were noted for deciding that adhesions, fibroids and pelvic inflammatory disease were the cause of pelvic pain. The EIP failed to demonstrate adequate reliability in determining whether any of the non-gynaecological causes were the cause of CPP. Conclusion There is little evidence on how panels should be convened, how information should be presented or what the best methods for consensus are and the MEDAL study highlights many potential challenges.

### P290

#### Investigating the effect of independent blinded digital image assessment on the STOP GAP trial

Peter Godolphin[1], Emily Patsko[1], Kim S. Thomas[1], Trish Hepburn[1], Eleanor J. Mitchell[1], Fiona E. Craig[2], Alan A. Montgomery[1]
[1]University of Nottingham; [2]NHS Grampian
**Correspondence:** Peter Godolphin
*Trials* 2017, **18(Suppl 1):**P290

This abstract is not included here as it has already been published.

### P291

#### A systematic review of core outcomes for Crohn's disease in adults

Heather Catt[1], Keith Bodger[1], Dyfrig Hughes[2], Jamie Kirkham[1]
[1]University of Liverpool; [2]University of Bangor
**Correspondence:** Heather Catt
*Trials* 2017, **18(Suppl 1):**P291

**Background**

Crohn's disease (CD) is a chronic condition causing digestive tract inflammation. Treatments may be used in combination and include corticosteroids, immunosuppressives, 5asas, biologics and antibiotics. All carry risks of adverse events (AEs).

The most important outcomes and AEs are not well defined, especially long term. There is no CD core outcome set (COS). One is proposed for Inflammatory Bowel Disease (IBD) using the International Classification of Functioning, Disability and Health (ICF) 1.

The study aim was to identify the important outcomes and AEs resulting from treatments for CD.

The study objectives were to:

- Perform a systematic review of trials, extracting the reported outcomes and AEs for CD.
- Classify the outcomes and AEs into a conceptual framework proposed for developing COSs (OMERACT Filter 2.03, in such a way as to rank their importance.
- Identify longer term AEs from the Summary of Product Characteristics (SPCs) 2 for the identified treatments.

**Methods**

MEDLINE, EMBASE, CENTRAL and CINAHL were searched. The inclusion criteria were: 1. Randomised controlled trials, 2. Adult patients with CD, 3. All interventions to treat CD and its complications.

AEs were standardised using the Medical Dictionary for Regulatory Activities (MEDDRA)5 terminology. Outcome and AEs were classified into domains using the ICF, the Wilson and Cleary model of health-related quality of life (QOL)6 and other domains recommended by OMERACT. The domains were used to populate the Filter 2.0 framework, creating the 'Crohn's Filter'.

**Results**

98 unique outcomes were identified from 182 studies. The outcomes were mapped to 37 outcome domains in Filter 2.0. Outcomes were most common in the domains of the structure of the intestine, defecation functions, pain in the stomach and abdomen and quality of life.

Disease activity indices and QOL questionnaires were used extensively in the included studies. Their coverage of the Crohn's Filter framework domains is dependent upon the combinations in which they are used.

Five hundred ninety-three unique AEs were identified in the trials, which mapped to 46 outcome domains in Filter 2.0. AEs were most common in domains of infections, sensations of pain, the structure of the intestine, pain in the stomach and abdomen and sensations associated with the digestive system.

**Conclusions and recommendations**

There is large variation in the outcomes and AEs reported across RCTs for CD, which supports the need for a core outcome set to be developed.

- Using an existing conceptual framework to organise outcomes data into domains and assessing their importance by frequency of reporting is a pragmatic solution in lieu of a consensus approach.
- Making use of AEs data is complicated as they may reflect disease process, failure or side effects of drugs and unrelated events.
- Researchers should make decisions on the use of disease activity indices and QOL questionnaires by considering the outcomes that will be captured. The mapping conducted here will aid this.
- Further recommendations will follow when the work is concluded (analysis of withdrawals and SPC data).

### P292

#### Outcome measures in clinical trials of traditional Chinese medicine for stable angina pectoris

Junhua Zhang[1], Dongmei Xing[2], Mingyan Zhang[1]
[1]Tianjin University of Traditional Chinese Medicine; [2]The first teaching hospital of Henan University of TCM
**Correspondence:** Junhua Zhang
*Trials* 2017, **18(Suppl 1):**P292

**Objectives**

To analyze the outcome measures in clinical trials of Traditional Chinese Medicine (TCM) for stable angina pectoris and provide data to develop core outcome sets (COS) for clinical trials of TCM.

### Methods
Three Chinese electronic biomedical literature databases (sinomed, CNKI and Wanfang) were searched to identify randomized clinical trials (RCTs) of TCM for stable angina pectoris published in 2010. RCTs evaluated the effectiveness of TCM for stable angina pectoris were considered to be eligible. There was no limitation to the type of TCM intervention and control. All reported outcomes and related information of included trials were extracted. Two authors screened literatures and extracted information dependently.

### Results
Finally ninety-four RCTs (with 9111 subjects) were included for analyses. There were totally 79 outcome measures were reported in all included RCTs. The ten mostly reported outcomes were efficacy rate of ECG, efficacy rate of angina pectoris, efficacy rate of TCM syndrome, fasting lipid profile, withdrawal rate of nitroglycerin, total scores of TCM syndrome, rate of clinical efficacy, nitroglycerin consumption, hemodynamic indexes, endothelin, efficacy rate of individual symptoms of TCM, frequency of angina attack. There were also several significant problems about outcomes in the included trials: (1) Significant heterogeneity of outcomes. The number of outcomes ranged from 1 to 21 with an average number of 5. For a same outcome, the evaluation points and detecting methods varied (2) Continuous data were arbitrarily transformed into ranked data. More than 90% of the trials reported the efficacy of outcome measures with percentages. (3) Only a few trials reported the outcomes associated advantages and characteristic of TCM. (4) Outcomes were randomly selected and reported with bias.

### Conclusions
Inappropriate outcomes with wrong evaluating points and methods will impaired the value of clinical trial. Establishing a core outcome set (COS) of TCM for stable angina are warranted.

### P293
### Standardisation and competency assessment of transanal total mesorectal excision (TATME) for rectal cancers: surgical quality assurance in the context of a randomised controlled trial
Alice Tsai, Stella Mavroveli, George Hanna
Imperial College London
**Correspondence:** Alice Tsai
*Trials* 2017, **18(Suppl 1):**P293

### Aim
Transanal total mesorectal excision (TATME) is a new innovative technique and theoretically allows more precise dissection with lower rates of incomplete mesorectum and involved circumferential resection margins comparing to laparoscopic TME. An international multicentre randomised controlled trial (RCT) comparing the two operations has been designed. Studies have shown the importance of standardisation of interventions in surgical rcts, however it is poorly conducted. The aim is to standardise the surgical steps and quality of tatme and develop a competency assessment tool (CAT) for Surgical Quality Assurance (SQA) of the trial.

### Method
A robust 4-round Delphi methodology was applied with peer-nominated international expert consultants in TATME. Semi-structured interviews were conducted in Delphi round 1 where surgeons gave details on how they perform TATME. Interviews were transcribed and analysed using a qualitative analysis software. The result was used for hierarchical task analysis (HTA) to identify the key stages and steps. The HTA result was subsequently used in Round 2, 3 and 4 as questionnaires, which contained all the variations and were distributed to the same group of experts. Each step was as rated mandatory, optional or prohibited. The steps that have reached 70% agreement were used to develop a competency assessment tool and operation manual for SQA.

### Result
The 4 rounds of Delphi achieved 96.4% of response rate. Three main phases were identified: peri-operative, abdominal phase and transanal phase. Four main stages were identified within the transanal phase: (i) Transanal platform set up, (ii) Purse-sting placement, (iii) TME dissection, and (iv) Specimen extraction and anastomosis. A seventy percent agreement level was achieved in 78 (83.4%) of procedural steps. For example, it is mandatory to place transanal purse-string suture with small bites (78.6%) to achieve a tight closure and stable pneumorectum, and begin transanal TME with circumferential full thickness mucosal dissection (92.9%). A Competency assessment tool (CAT) of TATME was developed incorporating the mandatory steps and quality from the standardisation process.

### Conclusion
Standardisation of techniques is crucial in a RCT in order to assess surgical competency for pre-trial recruitment and monitor compliance during the trial period. We have developed an objective and robust methodology to perform standardisation, which was used in developing an operation manual and a competency assessment tool (CAT) for SQA in an international multicentre RCT. Validation of TATME CAT will be performed to ensure usability, reliability and validity.
The CAT will be used in video analysis during the trial entry process. Surgeons that wish to participate will be invited to submit 2 unedited full-length videos for transanal TME and 1 for laparoscopic TME. There should be at least one male case for TATME; this is due to the fact that pelvic dissection in a male patient is usually more challenging. Both the abdominal and transanal components should be recorded and submitted. Two assessors approved by the expert panel will assess the videos independently using CAT.

### P294
### The EAGLE study: improving the wellbeing of men by evaluating and addressing the gastrointestinal late effects of radical treatment for prostate cancer
Kirsty Cockle[1], Ann Muls[2], Jervoise Andreyev[2], John Green[3], Sam Ahmedzai[4], Catherine Ferguson[5], Lesley Smith[6], Sara Pickett[7], Damian Farnell[8], Susan Campbell[1]
[1]Marie Curie Palliative Care Research Centre, Cardiff University; [2]The Royal Marsden NHS Foundation Trust; [3]Cardiff and Vale University Health Board; [4]Academic Unit of Supportive Care, Department of Oncology, University of Sheffield; [5]University of Sheffield Medical School; [6]Macmillan Cancer Support; [7]Swansea Centre for Health Economics, Swansea University; [8]School of Dentistry, Cardiff University
**Correspondence:** Kirsty Cockle
*Trials* 2017, **18(Suppl 1):**P294

### Background
Prostate cancer survival is improving, in the UK it has tripled in the last 40 years. Radiotherapy is an effective treatment with the aim of cure, however, many patients experience a change in bowel habit, such as urgency and incontinence, developing months to years after completion of treatment. Evidence suggests that management of these symptoms by a gastroenterologist can be effective when following a detailed investigative and treatment algorithm and an equivalent level of care can be given by a trained nurse. The EAGLE study focuses on the early identification and management of radiotherapy-induced bowel symptoms, with the aim of improving the quality of life of prostate cancer survivors and their partners and/or families.

### Methods
Successful evidence-based healthcare interventions are often challenging to embed into local clinical practice. In this effectiveness-implementation hybrid study, a multi-component intervention consisting of: a simple screening tool to identify men with late effects of radiotherapy in oncology follow-up clinics; rapid referral to a specialised gastroenterology service; and enhanced algorithm-led assessment leading to targeted advice and treatment, was implemented in three UK centres. Mixed method approaches to data collection and analyses are used to assess the effectiveness of the intervention and the implementation strategy at baseline, six month and twelve month intervals. Implementation outcome variables such as, acceptability to patients and healthcare professionals, large scale adoption and sustainability are addressed via longitudinal semi-structured interviews, alongside the introduction of possible solutions to facilitate local implementation. A control group of prostate cancer survivors with radiotherapy-induced bowel symptoms from another local health board are followed up for twelve months to assess the cost effectiveness of the intervention.

**Results**
The screening tool used in oncology to identify men suffering from the late effects of radiotherapy has been validated against the Gastrointestinal Symptoms Rating Scale (GSRS). Qualitative interview data from patients suggests that the screening tool is quick and easy to understand, addresses symptoms that are of most concern to patients, and facilitates open discussion of their bowel symptoms with healthcare professionals. Final results will measure the effect of the new service in terms of acceptability to staff and patients, quality of life improvements, symptom control and cost effectiveness. Qualitative interview data from healthcare professionals across the three UK sites will address how each team is working towards sustainability of the service.
**Conclusion**
This study evaluates the effectiveness of an enhanced assessment and treatment service, spanning oncology and gastroenterology departments, in improving the outcome of prostate cancer survivors post-radiotherapy. The factors needed for successful implementation into local practice, which are essential for new initiatives in healthcare settings, will act as examples of best practice for a network of centres of excellence in this field.

### P295
### Patients' experience in adaptive oncology clinical trials: trial acceptability, feasibility, efficiency, processes and outcomes
Annmarie Nelson, Mirella Longo
Cardiff University
**Correspondence:** Annmarie Nelson
*Trials* 2017, **18(Suppl 1):**P295

**Background**
Qualitative studies of participant experience embedded within oncology ctimps are rarely undertaken but can usefully explain why recruitment is slow, or why participants are unlikely to comply with the intervention in a real world setting, or why they might decline to enter or withdraw from the trial. Previous studies undertaken by the Marie Curie Research Centre, using patient experience as a secondary trial outcome, have: Highlighted patient preferences for treatment in a non-inferiority trial, Exposed issues of clinical equipoise in two feasibility trials that failed to recruit, Supported strategies for recruitment in a primary care trial, Explored participant's understanding of complex trial processes in a stratified trial of personalised therapies, Articulated the potential reasons that patients decline surgical trials. Additionally, participant interview data may be used in real time for presentation to TMG and TSC meetings to allow timely protocol amendments in order to improve recruitment and retention of participants. This concurrent approach is being successfully used in an HTA funded, ongoing clinical trial (ROCS: NCT01915693), and has enabled protocol amendments, such as timing of recruitment and assessments, that have improved recruitment and improved data capture.
**Aims**
An integrated qualitative component within an adaptive design trial can inform the primary and secondary trial objectives in the recruitment and testing phases of the trial and, for the purposes of an adaptive MAMS design, the stop/keep criteria per arm by assessing, for example: The feasibility of patients; Recruitment to the trial by examining their experience of consent and recruitment, including reasons for declining participation; Participants' motivation to accept randomisation to different interventions; Potential improvements to recruitment processes; Participants' understanding and experience of each trial arm; Participants' experience and tolerability of toxicities; Participants' attitudes to the value of predicted treatment benefit.
**Methods**
Qualitative data sets can be analysed for common themes in relation to real-time participant experience of the trial processes and treatment protocols. The analysis takes into account spontaneously reported participant experience, which reflects idiosyncratic attitudes and personal contexts, to enable patient reported outcomes supplementary to the stopping criteria in an adaptive design and main trial outcomes. A range of qualitative analytic frameworks and sampling strategies may be used according to the context and sensitivity of topic and the quality of the data. Our previous trials have utilised Thematic Analysis, Framework Analysis, and Interpretative Phenomenological Analysis, amongst others.
**Discussion**
Trials with adaptive designs attempt are intended to be efficient and streamlined, by modifying parameters, as appropriate, usually based on objective outcomes. The addition of participant experience as a secondary outcome measure allows for contextual factors and participant preference to be included in decisions to adapt, therefore increasing the likelihood of trial completion, and adoption and compliance with interventions in a real world setting..Patient experience of the intervention as a secondary outcome, combined with real time reporting of patient experience of trial processes can provide both immediate benefit to trial processes, and a further understanding of the acceptability and effectiveness of the intervention when aligned with the primary and secondary trial outcomes.

### P296
### Recruitment and participation in the stem cell trial of recovery enhancement-3 (stems-3): experiences of stroke survivors and their carer's
Claire Diver[1], Rebecca O'Connor[2], Nikola Sprigg[3], Louise Connell[4], Marion Walker[5], Philip Bath[3]
[1]School of Health Sciences; [2]Nottingham University Business School, University of Nottingham; [3]Stroke Trials Unit, University of Nottingham; [4]Allied Health Research Unit, University of Central Lancashire; [5]Division of Rehabilitation and Ageing, University of Nottingham
**Correspondence:** Claire Diver
*Trials* 2017, **18(Suppl 1):**P296

**Background**
Many stroke survivors have residual disability, even after discharge from post-hospital community rehabilitation. Advancements in acute stroke management suggest a role for the use of pharmacological agents in enhancing the neurological response to rehabilitation therapy. STEMS-3 (ISRCTN16714730) was a feasibility, 2x2 factorial randomised controlled trial of granulocyte colony stimulating factor (G-CSF) and/or physiotherapy at least 6 months after stroke. Recruitment and retention to clinical trials can be influenced by participant views, research design, contextual and environmental factors. It is reported stroke survivors believe continued physiotherapy will be beneficial, but it is not known whether these beliefs influence participation in clinical trials of rehabilitation. Evidence suggests willingness to participate in drug trials is directly related to the presence of an illness the drug is designed to improve. It is not known if this is true amongst stroke survivors or when drug therapy is offered in conjunction with rehabilitation interventions. Family members and carers frequently support older patients in decision making about participation in clinical trials; this has not been studied amongst stroke survivors. The aim of this study was to explore recruitment and participation in STEMS-3 from the perspective of participating stroke survivors and their carers.
**Method**
Qualitative interview study underpinned by theoretical perspectives of pragmatism and critical reflection using a purposive sampling strategy to identify stroke survivors who had been recruited to the trial and their carers. Semi-structured interviews were conducted, recorded and transcribed verbatim. Thematic analysis using an iterative and emergent approach was adopted. Results 16 participants (11 stroke survivors, 5 carers) were recruited from 60 participants in STEMS-3. 3 themes were identified: personal, practical and procedural. Decisions to participate were influenced by: opportunity for further treatment, potential benefits to others, practical factors including treatment and assessment location; side effects were considered worth the risk. Information given to stroke survivors prior to consent was perceived as too long and difficult to comprehend, and did not influence participation decisions. Carers supported decision making and in contrast to participants, valued detailed information. The principles of randomisation and blinding were understood by participants, and participants thought the trial team would not give them a treatment that was ineffective or harmful. There were concerns trial recruitment was used to facilitate discharge from existing services and trial completion was considered the ‘end of the road’; this was most marked for those that received no active intervention. Outcome measure assessments were often the first time anxiety, depression and personal relationships were discussed and heard by the carers.

**Discussion**

This study has highlighted how trial design results in multiple factors that impact on stroke survivors and their carers’ decisions to take part in clinical trials and their subsequent experiences. Feasibility and acceptability studies are essential in the evaluation and development of trials of complex interventions. They should include a qualitative investigation of study design that focusses on the procedural, practical and personal factors that impact upon individuals’ decisions to participate, and remain, in clinical trials. For trials involving stroke survivors consideration should be given to incorporating the views of caregivers who support stroke survivors.

### P297

#### Treatment preference and recruitment to a paediatric randomised controlled trial: managed activity graded exercise in teenagers and pre-adolescents (MAGENTA)

Lucy Beasant, Nicola Mills, Esther Crawley
University of Bristol
**Correspondence:** Lucy Beasant
*Trials* 2017, **18(Suppl 1):**P297

**Background & Objective**

Effective recruitment and retention in randomised controlled trials (RCTs) is hugely important. Reduced patient recruitment rates may prevent trial completion [1] and post randomisation dropout leads to the loss of statistical power [2]. Recruitment to an RCT can be affected if patients hold a preference for one of the treatments offered in the trial [3]. Qualitative research methodology embedded in adult RCTs has demonstrated that acknowledging and addressing patient concerns about preference can enhance the acceptability of randomisation and improve informed consent [4–6]. There is limited evidence on whether or not preferences are expressed by young patients and their parents during recruitment to paediatric trials, which have the added complexity and interplay between patient, parent and health professional equipoise [7]. The aim of the current research was to investigate whether effective communication approaches used to discuss treatment preference in adult trials were also effective in paediatric RCTs.

**Methods**

Recruiters were offered one-to-one training and feedback on four separate occasions as the feasibility trial progressed over a period of 12 months [8]. Recruitment discussions were recorded, transcribed and analysed thematically using techniques of constant comparison [9].

**Results**

Thematic analysis revealed: 1. Acceptance of preference at face value without exploration; this resulted in families declining the trial with little understanding of the reasons that underpin preference:

Mum 140: if we were given the graded exercise, I think he would probably withdraw Recruiter: I think, if you feel very strongly that you would want one treatment rather than the other, I think it’s better not to be in the study…[patient declined trial].

2. Identification and exploration of preference; in-depth discussion about reasons for preference allowed families to make a more informed decision about study participation:

Patient 3: It’s the exercise one I don’t really like the sound of

Recruiter: graded exercise therapy doesn’t mean that you’ve got to go and do, sort of a jog round the block, or anything like that, umm what it’s doing is monitoring your activity and helping you to build that up. We’re trying to sort out a better treatment for children in the future…some of our patients when we’re talking about exercise, umm, exercise for them is brushing their hair[patient accepted randomisation].

3. ‘We’ Or ‘I’; parents often expressed preference on their child’s behalf:

Mum 137: I think for us it’s probably gonna be the activity one, rather than the graded, umm, exercise, because, err, we’ve been doing the pacing, it’s been quite good, so that’s the one I’d probably lean against but I’d like to know a bit more about them both.

**Conclusions**

Communication strategies and approaches used to train recruiters in adult trials can be used in a paediatric setting with young patients (aged 10-17 yrs) and their parents, but care should be taken to explore reasons underlying both patient and parent preferences. Providing recruiters with training and guidance can ensure families are more fully informed about treatment options at recruitment, but further investigation of the effect on retention is required.

### P298

#### Framing the conversation: use of PRECIS-2 ratings to advance understanding of pragmatic trial design domains

Paula Darby Lipman[1], Kirsty Loudon[2], Leanora Dluzak[1], Rachael Moloney [3], Donna Messner[3], Catherine M. Stoney[4]
[1]Westat; [2]Stirling University; [3]Center for Medical Technology Policy; [4]National Heart, Lung, and Blood Institute, National Institutes of Health
**Correspondence:** Paula Darby Lipman
*Trials* 2017, **18(Suppl 1):**P298

**Background**

The intended purpose of the Pragmatic-Explanatory Continuum Indicator Summary (PRECIS-2) tool is to help trialists consider the impact design choices across nine domains have on the applicability of their results in the clinical setting: from “1” Very explanatory (intervention is tested under ideal conditions) to “5” Very pragmatic (intervention is tested under usual conditions). Added value of increased adoption of the tool is the emergence of a framework for investigators to communicate trial design decisions. PRECIS-2 is currently being used in a National Institutes of Health (NIH), National Heart, Lung, and Blood Institute (NHLBI) Pragmatic Trials Collaborative Project to explore whether a rating adjustment from the planning phase to the first year of implementation in a group of low-cost, pragmatic trials reflects true shifts in trial design.

**Methods**

PRECIS-2 ratings were collected at two annual in-person meetings of the Pragmatic Trials Collaborative Project. After a presentation and brief training at the first meeting (Time 1), Principle Investigators (PIs) rated their trials as originally designed (prior to trial initiation). After a refresher session at the second annual meeting (Time 2), PIs again rated their trials on current status on each domain, without access to their ratings from the first meeting. Qualitative telephone interviews were conducted with each PI subsequent to the meeting (summer 2016) to review the domains with a rating change; domains that were stable (no change in rating) were not discussed. The interview protocol included questions about the PI’s experience with and perception of the value of the PRECIS-2 tool. Interviews were recorded and transcribed.

**Results**

Five PIs completed paired ratings for Time 1 and Time 2 for 9 PRECIS-2 domains, resulting in a total of 45 paired ratings. Of these, over half (N = 24) demonstrated changes in ratings from Time 1 to Time 2, and each trial had changes on at least three domains (range “3” To “5”). Domains with the fewest changes were Recruitment Path, Primary Outcome, and Primary Analysis; and those with the most were Eligibility, Organization, and Flexibility of Adherence. Some domains were rated as more pragmatic at Time 2 (11/24 domains with higher rating), and others as more explanatory (13/24). Qualitative analysis of the interview data identified three reasons for a rating change: true change in design; change in interpretation of the domain; and misunderstanding of the PRECIS-2 domain. All PIs agreed that the tool is useful during the planning phase of trial design, and most have used the tool in subsequent work including proposals and protocols.

**Conclusion**

The NIH Pragmatic Trials Collaborative Project demonstrates how the PRECIS-2 tool can frame a conversation around initial trial design choices and provide clarity regarding where these decisions fall along the pragmatic-explanatory continuum. In addition to advancing our understanding of pragmatic trial design, discussions around application of the tool can be used to reflect on drivers of genuine adjustment to trial design as well as to help to clarify misunderstandings about the definitions of the tool domains which could be addressed through training.

### P299

#### Adapting trial design for challenging populations: how the rational MCC trial was designed to account for a rare, aggressive cancer in an elderly and diverse population

Rachel Blundred[1], Christina Yap[2], Sarah Pirrie[2], Sarah Bowden[2], Oliver Cassell[3], Carie Corner[4], Catherine Harwood[5], Pat Lawton[6], Clair Mcgarr[7], Ian Zealley[8]
[1]University of Birmingham; [2]Cancer Research UK Clinical Trials Unit, University of Birmingham; [3]Oxford University Hospitals NHS Trust; [4]East and North Herts NHS Trust; [5]Queen Mary University of London; [6]Nottingham University Hospital NHS Trust; [7]University Hospitals Birmingham NHS Foundation Trust; [8]NHS Tayside
**Correspondence:** Rachel Blundred
*Trials* 2017, **18(Suppl 1):**P299

**Background**

People with rare cancers need evidence-based treatment. Trials require large samples to disprove no difference in treatment effect. Rare cancers, like commoner malignancies, can be diverse affecting population selection, stratification and applicability of trials. The aggressive skin cancer, Merkel cell carcinoma (MCC) affects <300, predominantly elderly, UK patients annually. Standard management with surgery and/or radiotherapy for loco-regional MCC is based on retrospective data. Rational MCC aims to compare surgery and radiotherapy as first definitive treatment for MCC in control of loco-regional disease. The challenges encountered in designing this trial can be applied to many disease areas.

Challenge 1 - Accommodating diverse populations: Rational MCC accommodates biological diversity including presence of an integrated virus, immune dysfunction, immune infiltration and somatic mutations and multiple management pathways driven by clinical variation, uncertainty and opinion. This has been achieved through relaxed eligibility criteria and a focus on one question early in the treatment pathway. An inbuilt 3-year feasibility study allows adaptations to be made based on current data resulting in a more homogeneous population.

Challenge 2 - Design for rare populations: A conventional trial design would require >3000 patients to prove superiority beyond reasonable doubt. Instead, using a Bayesian probabilistic approach, observed data from 250 patients can still be informative to guide the decision-making process; providing clinicians and patients with probabilities that either treatment out-performs the other in reducing risk of loco-regional failure; aiding individual decisions.

Challenge 3 - Methodology for aggressive cancers: Rapidly progressing diseases leave little time for screening. Rational MCC has minimal screening requirements and collects most baseline data from routine investigations. In addition, sites are required to hit Protocol-defined treatment target times that mirror optimal clinical practice, ensuring that randomisation does not produce a delay to front line treatments.

Challenge 4 - Overcoming clinical preconceptions: It can be difficult for clinicians to consider alternative options when one treatment is used more commonly. Rational MCC has an observational arm mirroring the interventional arm, which patients can enter if they are unsuitable/unwilling to be randomised. This produces prospective data even if no patients are randomised. Sites will be provided with a checklist to guide SSMDT discussions to facilitate consideration of treatment options.

Challenge 5 - Working with elderly populations: It is important to address potential barriers in communication when working with elderly populations. Rational MCC patient literature has been reviewed by PPI representatives and multiple documents have been produced that reflect the information required at different time points. Large font versions of documents are available and information will also be provided in DVD format Randomised trials are expensive and failure to recruit wastes funding. The observational arm produces a bank of biological and clinical data even if patients are not randomised. Additional questions will be reviewed during the feasibility study to further maximise value. Data from the feasibility phase will be used to guide operational adaptations to ensure the Protocol continues to be informed by best practice The Rational MCC trial is funded by the Efficacy and Mechanism Evaluation (EME) Programme, an MRC and NIHR partnership.

### P300

#### Using a patient registry to conduct non-clinic engaged studies

Stephanie Dubose, Nicole C. Foster, Kellee M. Miller
Jaeb Center for Health Research
**Correspondence:** Stephanie Dubose
*Trials* 2017, **18(Suppl 1):**P300

This abstract is not included here as it has already been published.

### P301

#### Increasing follow-up response among parents of very preterm infants: personalised contact, external promotion, and web-based questionnaires

Madeleine Hurd[1], Louise Linsell[1], Ed Juszczak[1], Oliver Hewer[1], Ursula Bowler[1], Samantha Johnson[2], Jon Dorling[3], all on behalf of 'The SIFT Collaborative Group
[1]University of Oxford; [2]University of Leicester; [3]University of Nottingham
**Correspondence:** Madeleine Hurd
*Trials* 2017, **18(Suppl 1):**P301

**Background**

In randomised control trials where outcomes are collected via questionnaire, maximising the response rate is paramount. Loss to follow-up reduces the effective sample size and can place added assumptions on analysis, potentially leading to bias and compromising the validity of results. SIFT (ISRCTN76463425) is a multicentre randomised controlled trial of a feeding intervention in very preterm or very low birthweight infants (gestational age at birth <32 weeks; birth weight <1500 g) in neonatal units in the United Kingdom and Ireland. Primary outcome is the proportion of infants surviving without moderate or severe disability at 24 months of age corrected for prematurity. This is assessed via a questionnaire sent directly to parents asking for information on their infant's health and development.

Having confirmed address details and survival status, the SIFT Trial Coordinating Centre sent participants' parents a card for their child's second birthday and a thank-you note reminding them of the questionnaire and encouraging them to update their contact details. Staff then posted a questionnaire to the parent (including a sticker set for their child) 17 days before the child's age-corrected second birthday. If there was no response, another copy of the questionnaire plus reminder letter was posted on their age-corrected birthday. Two weeks later, another copy of the questionnaire was posted, and two weeks after that, SIFT staff attempted to call parents about the questionnaire.

Follow-up began in August 2015. By February 2016, response rate remained low: approximately 50%.

**Objective**

To outline measures taken to increase follow-up response rate during the trial and their efficacy.

**Methods**

The following adaptations were made to the follow-up process:

1) One week prior to sending the first questionnaire, the SIFT Trial Coordinating Centre telephoned parents to confirm address details and inform them that the questionnaire was to be dispatched. Parents were also telephoned within a day of the second reminder being posted (1 February 2016 onwards).

2) A link to an electronic version of the questionnaire was sent to parents with a valid email address or mobile number within a day of posting the first paper version (26 February 2016 onwards).
3) Parents were contacted by text message as well as or instead of by telephone at second reminder stage (29 March 2016 onwards); charity for premature and sick babies BLISS promoted the follow-up via Facebook and Twitter (5 April 2016).
4) Posters publicising follow-up were sent to recruiting sites for display, accompanied by a list of local participants with their response status (20 June 2016 onwards).

**Results**

Response rate prior to all interventions was 51.0%. On 30 September 2016 it was 68.0%, a significant increase. The response rate also increased (non-significantly) after implementation of each new initiative. SIFT intends to initiate an incentives programme to further increase the rate (subject to ethical approval).

**Conclusion**

Contacting parents prior to dispatching questionnaire; reminder to parents via text message; availability of questionnaire electronically; and promotion via hospitals or charities are approaches which, when combined, can significantly increase follow-up rate.

### P302

#### Learning lessons from trial decliners about improving recruitment: qualitative study

Adwoa Hughes-Morley[1], Bridget Young[2], Roelie J. Hempel[3], Ian T. Russell[4], Waquas Waheed[5], Peter Bower[2]
[1]University of Manchester and University of York; [2]MRC North West Hub for Trials Methodology Research, University of Liverpool; [3]School of Psychology, University of Southampton; [4]Swansea University Medical School, Swansea University; [5]NIHR School for Primary Care Research, University of Manchester
**Correspondence:** Adwoa Hughes-Morley
*Trials* 2017, **18(Suppl 1):**P302

This abstract is not included here as it has already been published.

### P303

#### An intervention to optimise recruitment in challenging RCTs: implementation of the 'quintet recruitment intervention' across eight pilot/feasibility RCTs

Leila Rooshenas[1], Kerry Avery[1], Daisy Elliott[1], Sangeetha Paramasivan[1], Alba Realpe[2], Caroline Wilson[1], Jenny L Donovan[1], on behalf of the quintet study group
[1]University of Bristol; [2]University of Warwick
**Correspondence:** Leila Rooshenas
*Trials* 2017, **18(Suppl 1):**P303

**Background**

Randomised controlled trials (RCTs) are the most rigorous approach for evaluating health care interventions, but many fail to recruit to target, requiring costly extensions. Anticipation of recruitment challenges can also deter investigators and funders from tackling important, potentially practice-changing clinical questions. Developing interventions to optimise recruitment is a priority in trials methodology research, but few interventions are transferrable across RCTs. The quintet Recruitment Intervention (QRI) aims to optimise recruitment in 'difficult' RCTs, whilst safeguarding informed consent. The intervention comprises in-depth investigation of recruitment obstacles (Phase I), followed by implementation of strategies to address recruitment challenges as the trial proceeds (Phase II). Having integrated the QRI in eight rcts anticipated by funders/trial investigators to encounter recruitment challenges, we undertook a preliminary evaluation of the QRI's implementation.

**Methods**

The QRI was integrated into the recruitment processes of eight 'difficult' feasibility/pilot RCTs that addressed controversial issues or compared very different treatment arms (including 'no treatment'). Phase I of the QRI involved collecting and analysing interview and audio-recorded consultation data to understand recruitment challenges. Phase II involved tailored interventions to improve recruitment, including components such as trial-specific feedback/training and suggestions to clarify patient-facing documentation. Data provided by clinical centres were aggregated and displayed graphically to compare targets with achievements over time, in relation to eligibility assessment and numbers approached, consented, and randomised. QRI intervention-components were also plotted over time.

**Results**

Recruitment in the initial periods of these RCTs rarely hit targets (Phase I of the QRI), with the exception of one trial where recruiters had previously received QRI training. Recruitment rates moved closer to targets after implementation of QRI interventions (Phase II), with the scale and patterns of improvement varying across RCTs. A sharp increase in recruitment was evident in an RCT following a feedback/ training session attended by all centres, whereas smaller improvements were seen in a multi-centre trial where a subset of centres attended sessions. Other evidence of recruitment improvement included centres moving from 'no recruitment' over weeks/months, to recruiting first participants after feedback (four RCTs). Despite their challenging nature, four pilot/feasibility RCTs recruited to target and progressed to main trials, and two are currently recruiting well. Two feasibility studies did not progress to main RCTs: one, because the QRI enabled a nuanced understanding of equipoise issues previously unbeknown to the trial management group, which were discussed during a feedback session and found to be unsurmountable; and the other, because a main trial was not feasible due to insufficient incident cases of eligible patients. Recruiters exposed to QRI training in the latter trial went on to successfully recruit to a subsequent RCT with integrated QRI, which consistently recruited above target despite anticipated difficulties.

**Conclusion**

These preliminary observational data provide some evidence to show an association between QRI interventions and improved recruitment in very challenging RCTs. The QRI is increasingly appreciated by RCT investigators and funders. Formal evaluation of the QRI is itself challenging, and will need to carefully consider appropriate markers of success that include recruitment rates and levels of informed consent.

### P304

#### Generating student recruiters for randomised trials in surgery (GRANULE): a one-day course to empower a cohort of future surgical trialists

James Glasbey[1], Dmitri Nepogodiev[1], Jane Blazeby[2], Dion G. Morton[1], Nicola Fearnhead[3], Simon Bach[1], Thomas Pinkney[1], Aneel A. Bhangu[1]
[1]University of Birmingham; [2]University of Bristol; [3]University of Cambridge
**Correspondence:** James Glasbey
*Trials* 2017, **18(Suppl 1):**P304

**Background**

Surgical trials are complex; patient populations are heterogeneous, interventions are difficult to standardise, and outcome measures are difficult to assess. Fewer patients are recruited to trials in surgery and anaesthesia than any other medical specialties. There is an urgent need to train a cohort of future surgical trialists, equipped with skills to convey clinical equipoise and appropriately recruit patients into well-designed randomised studies.

**Method**

A national, one-day course (GRANULE) was conducted with support from Birmingham Clinical Trials Unit, and the Bristol Medical Research Council-funded conduct-II hub. Undergraduate medical students were competitively selected from a national UK student research group (Student Audit & Research in Surgery). Completion of Good Clinical Practice (GCP) certification was required prior to commencement.
GRANULE course content was framed around active trials within colorectal and oesophagogastric surgery, with a faculty of chief investigators and active trial recruiters. The course featured; 1. A series of interactive lectures to give clinical context (STAR-TREC (Birmingham), ACCURE-UK (Birmingham) and By-Band-Sleeve (Bristol)); 2. Structured, small group discussions surrounding challenges in trial recruitment; 3. Simulated patient recruitment, with formative assessment of actor interactions. Confidence and experience in trials recruitment was collected pre- and post-intervention using paired, self-reported, 15-point online tools. 5-point Likert scales were used to quantify confidence across four domains (understanding of equipoise, ability to communicate equipoise, communicating risks and benefits of research involvement, gaining consent for participation in trials). A mix of positive and negatively weighted statements were used to minimise acquiescence bias. Changes in perceived confidence were tested to a 5% level of significance using McNemar's paired chi-squared test. Free text feedback underwent thematic analysis.

**Results**

Twenty-four delegates (10 female, 14 male) were invited to complete the course. All 24 completed GCP certification prior to attendance, and completed feedback (response rate = 100.0%). After course completion, participants felt more confident in their understanding of clinical equipoise ($P < 0.001$), ability to communicate clinical equipoise effectively ($P < 0.001$), ability to communicate risks and benefits of each trial intervention ($P < 0.001$) and gain consent for participation in trials ($P = 0.004$). In free-text analysis, the simulated patient interactions, and the high facilitator: student ratio were highly commended. Lessons learnt included reducing the complexity of included trial interventions, and giving extended time for single delegates' simulated patient interactions.

**Discussion**

Feedback from the GRANULE course demonstrated that undergraduate medical students in the UK can be trained to confidently recruit patients to high-quality clinical trials. As an early-years intervention, this course has the capacity to change the culture of trials recruitment within surgery at a junior level. Course delegates can access active networks of postgraduate, trainee-led surgical research networks in the UK to foster new mentoring relationships and actively recruit patients into portfolio trials. Further course iterations are planned for Spring 2017 (UK) and September 2017 (Germany).

**Acknowledgements**
With special thanks to the Bowel Disease Research Foundation.

### P305

#### Managing the challenge of recruiting foster carers into a trial of a supportive group-based social care intervention: lessons learned from the confidence in care trial

Gwenllian Moody[1], Lucy Brookes-Howell[1], Rebecca Cannings-John[1], Sue Channon[1], Elinor Coulman[1], Mandy Lau[1], Alyson Rees[2], Jeremy Segrott[3], Jonathan Scourfield[2], Michael Robling[1]
[1]South East Wales Trials Unit (SEWTU), Centre for Trials Research, Cardiff University; [2]Children's Social Care Research and Development Centre (CASCADE), Cardiff University; [3]South East Wales Trials Unit (SEWTU), Centre for Trials Research & Centre for the Development and Evaluation of Complex Interventions for Public Health Improvement (decipher), Cardiff University
**Correspondence:** Gwenllian Moody
*Trials* 2017, **18(Suppl 1):**P305

**Background**

The Confidence in Care (CIC) trial is evaluating Fostering Changes (FC), a training programme for foster and kinship carers which aims to increase their skills and coping strategies and improve their relationship with their child. The CIC consortium of third-sector providers train groups of 12 carers in both trial and non-trial settings. Groups are timed to commence with each new school term, which is then reflected by waves of recruitment to the trial. Previous evaluations have found filling groups to be challenging, both Pallett et al. (2002) and Briskman et al. (2012) ran groups with as few as six carers, which impacts fidelity. FC requires carers to attend twelve 3-hour weekly sessions, a considerable time commitment and possibly a recruitment barrier. Randomised controlled trials in social care settings are also uncommon compared to public and clinical health and in two pilot sites we tailored, tested and revised our recruitment strategy.

**Methods**

We piloted at two sites prior to the first main recruitment wave, and further optimised recruitment strategy in initial main trial waves. Strategies introduced both preceded and followed the piloting phase. These included 1) supplementing postal approaches to carers from their respective Local Authority (LA) or Independent Fostering Provider (IFP) by asking LAs and IFPs to select eligible carers from their databases for direct invitation, 2) increasing lead time from approaching provider to group initiation to up to four months, 3) modifying the allocation ratio from 1:1 to 2:1 (intervention:control) to increase the chances of obtaining the required training group size, 5) revising participant materials to better align with the expectations of foster carers.

**Results**

Since the changes were fully implemented after the pilot and the first main recruitment wave, the average number of carers recruited to each site increased from 9.17 (5.83 intervention: 3.33 control) to14.25 (9.25 intervention: 5.00 control). While group sizes have increased, it remains difficult to reach target group size. Most consenting participants are now sourced via the direct approach from providers (as opposed to the ongoing open approach letter). During the second main recruitment wave, 85.09% of consented participants were sourced directly from providers, whereas the remaining 14.91% were self-nominated.

**Conclusion**

Recruitment to the CIC trial has improved since the above amendments were made and we expect to reach our recruitment target. Modifying the allocation ratio increases the target sample but we considered on balance that this made the study both more likely to achieve clinically viable groups and was more acceptable to providers. However, the impact of remaining sub-optimal group size on fidelity will need to be determined. Similarly, the quality of communication about the trial to carers and also local providers will be assessed in our process evaluation. One pilot site did not continue into the trial phase but has enabled an on-going carer contact group to provide timely input to both our recruitment and retention strategy. Recruiting participants potentially well in advance of group onset introduces, as well as avoids some problems which will be reviewed in our presentation.

### P306

#### Reported reasons for missing data

Anna Kearney[1], Anna Rosala-Hallas[2], Naomi Bacon[2], Anne Daykin[3], Alison J. Heawood[3], Athene Lane[3], Jane Blazeby[3], Mike Clarke[4], Paula R. Williamson[1], Carrol Gamble[1]
[1]North West Hub for Trials Methodology Research/University of Liverpool; [2]Clinical Trials Research Centre/University of Liverpool; [3]conduct-II Hub for Trials Methodology Research/University of Bristol; [4]Centre for Public Health, Queen's University of Belfast
**Correspondence:** Anna Kearney
*Trials* 2017, **18(Suppl 1):**P306

**Background**

Missing data in clinical trials is common and can reduce trial efficiency and bias the estimate of treatment effect. Higher levels of missing data have been associated with specific study areas (e.g. Mental health, substance abuse), longer lengths of follow up, trials with more than two arms, and poor allocation concealment. However, little is known about the prevalence of different causes of missing data. Analysis of the reported reasons for missing data within published literature could provide valuable insights to inform the development of effective strategies to mitigate the problem.

**Methods**

Two cohorts were identified: A search of Medline (Ovid) for randomised trials published within the four major medical journals in 2013; National Institute of Health Research Health Technology Assessment Programme (NIHR HTA) monographs published between 2009 and 2014. Parallel, two arm randomised control trials were included. Early phase trials and pilot studies were excluded. Data on the number of patients randomised, analysed, excluded and imputed was independently abstracted by two authors along with reported reasons and trial characteristics. Missing data was defined as the number of randomised patients who did not contribute primary outcome data to the analysis either because it was not available or because the data was excluded.

**Results**

In 7/166 (4%) journal articles and 2/36 (6%) HTA monographs it was not possible to determine the levels of missing data. 141/159 (89%) and 34/34 (100%) trials had missing primary outcome data with median [IQR] levels of missing data of 5.5% [1.5, 11.0] and 11% [2.9, 19.8].

The impact of the missing data within the journal cohort meant that 454 months were wasted across 126 trials recruiting patients that did not contribute outcome data (median [IQR] per trial: 1.5 [0.7, 3.2]). Trials reported multiple reasons for missing data: patient withdrawals (64%, n = 141); patients lost to follow up (60%); investigator exclusions (53%) and failure of clinical staff to measure the outcome (45%). 91/159 (57%) imputed data and 91 (57%) trials excluded randomised patients with protocol deviations or missing data from the analysis population. 41 (26%) trials used both approaches. Abstraction of the levels of missing data was challenging. CONSORT statements were often misleading around imputation of data and did not clearly report the number of outcomes known.

**Conclusion**

The percentage of missing data within the leading journals was on average lower than expected. However, a comparison with the HTA monographs suggests this may reflect a difficulty publishing RCT's with missing data in the top journals. Patient withdrawal and loss to follow up were the leading causes of attrition, although the failure of researchers to measure the outcome in retained patients was cited in nearly half of the trials. Reporting missing data was often inadequate. We would recommend stricter adherence to the CONSORT flow diagram and suggest revisions to ensure that the flow diagram could be standalone with additional categories to allow distinction between the numbers analysed and the numbers for whom the outcomes were known and imputed.

### P307

#### Identifying effective retention strategies: a research agenda

Anna Kearney[1], Anne Daykin[3], Alison J. Heawood[3], Athene Lane[3], Jane Blazeby[3], Mike Clarke[4], Paula R. Williamson[1], Carrol Gamble[1]
[1]North West Hub for Trials Methodology Research/University of Liverpool; [2]Clinical Trials Research Centre/University of Liverpool; [3]conduct-II Hub for Trials Methodology Research/University of Bristol; [4]Centre for Public Health, Queen's University of Belfast
**Correspondence:** Anna Kearney
*Trials* 2017, **18(Suppl 1):**P307

**Background**

Identifying strategies to minimise missing data was the second highest methodological research priority in a Delphi survey of the Directors of UK Clinical Trial Units (CTUs). However, a Cochrane Methodology Review of nested randomised studies of missing data strategies shows a substantial evidence gap. The review demonstrates an emphasis on improving questionnaire response rates and an absence of evidence that address the full range of causes of missing data. In addition, published case studies frequently describe the use of strategies which have not been evaluated. Our aim was to assess current retention practices within the UK and identify priorities for future research to evaluate the effectiveness of strategies to reduce attrition. Methods: 75 Chief Investigators of National Institute of Health Research Health Technology Assessment (NIHR HTA) funded parallel randomised control trials starting between 2009 and 2012 and 47 UK Clinical Trial Units (CTUs) were surveyed to identify what approaches and strategies were used to mitigate missing data in trial design and conduct.

Responses from the current practice survey were used to inform a subsequent two round Delphi survey with CTUs to gain consensus around research priorities to assess the effectiveness of missing data interventions.

**Results**

50/75 (66%) Chief Investigators and 33/47 (70%) CTUs completed the current practice surveys. 78% of Chief Investigators were aware of retention challenges and implemented strategies at trial design. Patient initiated withdrawal was the most common cause of missing data. CTUs routinely used newsletters, timeline of participant visits, and telephone reminders to mitigate missing data. CTUs reported evaluating 36 of the 59 strategies presented using nested studies or a comparison of retention before and after implementation. However, some frequently used strategies such as site initiation training have had no research to inform practice.

35 CTUs (74%) participated in the Delphi survey of which 34 (97%) completed both rounds. Pre-defined consensus was reached on seven topics. Six retention strategies met consensus that further research was of critical importance: site initiation training; frequency of patient contact during a trial; the use of routinely collected data; the frequency and timing of reminders; triggered site training and the length of time needed to complete questionnaires. In contrast, 82% reached consensus that research into the effectiveness of Christmas cards for site staff was of low importance.

**Conclusion**

The survey of current practices demonstrates a variety of strategies are being used to mitigate missing data but with little evidence to support their use. This Delphi survey has identified a consensus of research priorities to be evaluated.

### P308

#### Optimising the recruitment of underrepresented ethnic minority patients to telehealth diabetes trials: examining the role of language and research reporting practices

Talia Isaacs[1], Daniel Hunt[2], Danielle Ward[3], Leila Rooshas[3], Louisa Edwards[3]
[1]University College London; [2]University of Nottingham; [3]University of Bristol
**Correspondence:** Talia Isaacs
*Trials* 2017, **18(Suppl 1):**P308

**Background**

Randomised controlled trials (RCTs) are often considered the gold standard of health intervention research. However, most RCTs under-recruit ethnic minority patients, potentially jeopardising the external validity of their findings. One recruitment challenge relates to assessing whether patients have sufficient language proficiency to provide informed consent and engage with the intervention. However, little is known about how trial recruiters assess potential participants' language proficiency. Using diabetes telehealth intervention RCTs as a case study, we investigated the proportion of published trials that include language proficiency as part of their inclusion criteria, including how and why they do so. A secondary objective was to explore any links between the inclusion of language-related eligibility criteria and the proportion of ethnic minorities recruited.

**Methods**

A systematic review was conducted on telehealth intervention RCTs that focused on type 2 diabetes and excluded ethnically-targeted studies. Two reviewers independently conducted abstract and full-text screening, risk of bias assessment, and data extraction.

**Results**

Of 3358 records identified in the search, 79 articles consisting of 58 distinct RCTs were included in the review. Half of the included RCTs (29/58) referred to patients' language proficiency as an eligibility criterion. However, there were no common procedures across RCTs to determine if patients had the requisite language ability to participate. Whereas some studies listed different combinations of language skills as being necessary (speaking, listening, reading, writing), others referred to patients' need to be native speakers. In two RCTs, there was a mismatch between the telehealth medium used and the language skills cited (e.g., telephone intervention requires writing but not speaking ability), whereas four underspecified the language skills required (e.g., speaking but not reading ability stated as necessary for a telephone and computer-text intervention).

The 29 RCTs that referred to language as a patient eligibility criterion tended to be larger-scale, recruiting nearly 1.7 times the total number of recruited patients, compared to the 29 RCTs that made no reference to language at all. Twenty-one RCTs in the former group and 17 in the latter provided ethnicity information and recruited a median of 24.6% versus 18.0% ethnic minority patients as a proportion of the total sample respectively. The RCTs that included language proficiency as an eligibility criterion recruited a greater proportion of ethnic minority participants (37.8% of all recruited participants) compared with those that did not (13.9%).

**Conclusions**

Approaches for assessing patients' language proficiency were found to be inconsistent in the context of diabetes telehealth intervention RCTs. Studies referring to language in patient screening might report more on ethnicity because some ethnic minorities are also linguistic minorities. Or it may be that these studies are more robust in terms of research reporting and sample size. There was no evidence that reference to language screening is associated with lower participation from hard-to-reach groups, although the soundness and consistency of individual inclusion/exclusion decisions on language grounds could not be ascertained. Future research should focus on developing and validating a language assessment tool that could be consistently applied across RCTs to screen patients' language proficiency during recruitment.

### P309

#### Does appearance matter? A study within a study

Lucy Culliford, Rachel Brierley, Jonathan Betts, Jenny Lamb, Rachel Maishman, Barney Reeves[1], Chris Rogers[1]
[1]University of Bristol
**Correspondence:** Lucy Culliford
*Trials* 2017, **18(Suppl 1):**P309

**Background**

A central tenet of recruitment to clinical studies is that participants take part freely, armed with full information about the study. There has been little research into how the appearance of the information may affect recruitment. A study of Patient Information Leaflets (PILs) concluded that PILs need 'to be well structured and designed in an appealing manner'. These aspects have not yet gained sufficient attention [1]. In the case of paper information leaflets, production of a high quality attractive leaflet is possible, but may require specialist software, and incur extra costs for colour printing. Without the evidence of benefit, the additional resources may not be justified.

**Methods**

To investigate if the appearance of pils affects recruitment, we chose to embed a randomised controlled trial (RCT) within the Outcome Monitoring After Cardiac Surgery (OMACS) study. OMACS uses routine NHS data alongside participant questionnaires, and consent is sought by post at 3 months post-surgery. OMACS was chosen as the 'host' study as around 120 patients are approached for participation per month, allowing evidence to be collected quickly. Participants are randomised to receive one of 3 PILs: a tri-fold coloured leaflet produced using a graphic design package, indesign, (PIL A), a coloured A4 sheet produced in Microsoft Word (PIL B), and a standard A4 black and white sheet (PIL C). Both coloured leaflets are printed professionally. The information contained in each leaflet is identical and participants do not know about the randomised element of OMACS. The sample size is 1590 which, assuming a consent rate of 70% (based on a previous similar postal questionnaire study that achieved this (personal communication)), will provide 90% power to detect a 10% difference in consent rate between any pair of PIL formats, with an overall significance level of 5%.

**Results**

After 5 months, we have sent out 436 invitation letters and have 182 consented participants. Consent rates for each PIL are: A - 68/181 (38%) B - 76/180 (42%) C - 76/180 (42%) An unexpected finding is that consent rates are much lower across the study than was anticipated. We are currently investigating possible reasons for this. If the current trend continues we will review the implications for the sample size and power of the study.

There are a number of differences between OMACS and the previous study which may explain the difference in response rates. Previously, the timing of the approach for consent is different (1 year versus 3 months) and patient packs were simpler with fewer documents than used in the OMAC study. In attempt to cater for participants' preferences, OMACS invites participants to elect for alternative response methods (e.g. Postal versus internet questionnaire) and also allows them to opt out of some aspects of the study. The previous study did not include this diversity of options.

**Conclusion**

At this early stage, no formal conclusions can be drawn as to the effect of the appearance of the PIL. One year results with a formal comparison between the rates of each group will be presented.

### P310

#### Reducing missing data in palliative care randomised controlled trials: a mixed-methods study

Jamilla Hussain[1], Martin Bland[2], Miriam J. Johnson[3], David C. Currow[4], Ian R. White[5]
[1]Hull York Medical School, University of York; [2]Health Sciences Department, University of York; [3]SEDA, University of Hull; [4]Palliative and Supportive Services, Flinders University; [5]MRC Biostatistics Unit, University of Cambridge
**Correspondence:** Jamilla Hussain
*Trials* 2017, **18(Suppl 1):**P310

**Background**

To reduce the risk missing data (MD) pose to the power, precision and validity of trial findings, MD should not only be handled appropriately at the analysis stage, but more importantly potentially reversible MD risk factors must be identified and modified at the design and conduct stage. This mixed-methods study used palliative care trials, where MD due to death and disease progression are expected, to explore the association between primary outcome MD and participant, trial site and trial-level MD risk factors.

**Methods**

(i) Trial-level factors: systematic review and meta-regression of primary outcome MD in 108 palliative care trials; (ii) Participant and site-level factors: multi-level cross-classified modelling using individual participant-level data (IPD) from 10 multi-site trials; (iii) Identification and exploration of factors in more depth: thematic analysis of interviews with 27 research personnel and participants.

**Results**

(i) Systematic review: MD was associated with increasing numbers of questions/tests requested (odds ratio (OR) 1.19 per-doubling, 95%CI 1.05, 1.35) and longer study duration (OR 1.09 per-doubling, 95%CI 1.02, 1.17). (ii) IPD: At the participant-level (n = 1,846), MD was associated with baseline missingness (OR 17.19, 95%CI 8.55, 34.53) and poorer Karnofsky Performance Status (10-unit increase: OR 0.78, 95%CI 0.70, 0.87); at the site-level (n = 35), MD at the end of follow-up was associated with sites that randomised a greater number of participants (per 10-randomisations: OR 1.08, 95%CI 1.01, 1.16) and with fewer research personnel (4 personnel compared to 1: OR 0.07, 95%CI 0.01, 0.84). (iii) Interviews: themes included "attention-to-detail vs. attention-to-person", "clinical vs. research-role tension", and "beyond GCP training".

**Conclusion**

There is the potential to reduce MD in palliative care trials by modifying the factors associated with MD identified from this study. Further development of the theoretical framework is required, prior to developing an intervention to reduce MD that will be tested within trials.

*In accordance with the submission guidance for the Sylvan Green Travel Award the word count has been limited to 300.

### P311

#### Use of a non-diabetic spouse control group in the DCCT/EDIC hearing study

Barbara Braffett[1], Gayle Lorenzi[2], Xiaoyu Gao[1], Kathy Bainbridge[3], Annette Barnie[4], Catherine Cowie[5], Karen Cruickshanks[6], Dayna Dalton[7], Rose Gubitosi-Klug[8], John Kramer[9]
[1]George Washington University; [2]University of California, San Diego; [3]National Institute on Deafness and Other Communication Disorders; [4]University of Toronto; [5]National Institute of Diabetes and Digestive and Kidney diseasesniddk; [6]University of Wisconsin; [7]Episense, University of Wisconsin; [8]Case Western Reserve University; [9]University of Iowa
**Correspondence:** Barbara Braffett
*Trials* 2017, **18(Suppl 1):**P311

**Introduction**
Hearing impairment is common among the general public >50 years of age however the relationship between type 1 diabetes (T1D) and hearing impairment in this age group is not well-studied. To examine this question, the Diabetes Control and Complications Trial/Epidemiology of Diabetes Interventions and Complications (DCCT/EDIC) Hearing Study is examining the prevalence of hearing impairment among a well-phenotyped T1D cohort (mean age 55 years).
**Objective**
To determine the feasibility and comparability of using randomly selected spouses of surviving DCCT/EDIC participants as a non-diabetic control group.
**Background**
Use of spouses for the control group was based on unique factors. Most spouses were familiar with the DCCT/EDIC study and staff by virtue of their partner's long-term participation and frequent accompaniment to study visits. Most important, the spousal group was expected to be similarly distributed in age, race and socioeconomic status to the DCCT/EDIC cohort. Additionally, practical efficiencies in recruitment, scheduling, and travel were expected.
**Methods**
Of the total of 875 spouses, 510 were randomly identified for screening. Enrollment of 270 spouses would provide 90% power to detect a clinically significant difference in hearing impairment between the EDIC surviving cohort and controls. Permission from the EDIC participant was needed prior to contacting his/her spouse. Spouses with known diabetes, or illness/disability that precluded travel to the clinical center were excluded. A self-administered hearing assessment, brief medical history, physical measurements, hba1c and audiometry were performed on consenting participants and spouses. All data collection methods and equipment were standardized and consistent with DCCT/EDIC methods. Testing was performed by trained and study-certified personnel. All audiograms were scored centrally.
**Results**
Of the 510 spouses identified, 39 (7.7%) were ineligible, 97 (19%) were not approached (due to participant request, distance, illness/disability, work demands, marital discord), and 88 (17.3%) were approached but declined (work demands, travel, illness/disability, disinterest). A total of 289 spouses and 1150 (86.7% of surviving, 94.5% of active) EDIC participants were evaluated. Spouses determined to have diabetes based on hba1c (n = 5) were excluded from the analyses. The spousal group was similar in age, race, education, smoking status and systolic blood pressure.
**Conclusion**
Spouses of research participants may be a resource for studies requiring a comparison group with similar demographic characteristics. Potential obstacles to spouse participation, such as participant refusal to allow contact spouse, distance/travel, and illness/disability need to be considered. Clearly defined eligibility criteria, recruitment strategies and testing procedures are needed to ensure valid comparisons between groups. Standardized evaluations by trained staff may yield stronger results compared to the use of published comparison groups.

### P312

#### Challenges in the analysis of a randomised controlled trial with retrospective consenting: the RAPIDO study

Helena Smartt[1], Katie Pike[1], Rosy Reynolds[2], Margaret Stoddart[2], Chris A. Rogers[1], Alasdair MacGowan[2]
[1]University of Bristol; [2]North Bristol NHS Trust
**Correspondence:** Helena Smartt
*Trials* 2017, **18(Suppl 1):**P312

**Background**
Studies in which patient consent cannot be obtained prospectively represent a particular methodological challenge. Approval can be sought to include patients in a randomised study without their consent, but every effort must be made to seek informed consent when the patients has recovered sufficient capacity or to seek consent from a consultee if this doesn't occur. We describe our experience of seeking retrospective consent in the context of the RAPIDO trial, and the implications for the study analyses.
**Methods**
RAPIDO is a multi-centre RCT comparing the effect on mortality of conventional versus rapid diagnostic pathways for suspected blood stream infections in hospitalised patients. In the conventional pathway, infective micro-organisms in a blood sample are identified within 3–5 days, whereas the rapid diagnosis pathway takes 1 hour or less. Identifying the infective micro-organisms then allows an appropriate antibiotic to be chosen for treatment. It has been suggested that earlier antibiotic therapy could improve patient outcomes including 28 day mortality (primary outcome), length of hospital stay and time to resolution of fever (secondary outcomes). Due to the time-sensitive nature of this study, participants were consented retrospectively; where patients had left the hospital before consent was obtained, postal consent was sought.
**Results**
A total of 8628 patients were randomised across 7 UK centres, 6692 of which were found to be eligible for the study. Consent was obtained before hospital discharge for 2606 (39%) patients and postal consent was successfully sought for a further 521 (8%). 1142 (17%) declined. Of the remainder, 1341 (20%) died before consent could be sought and consent was not obtained for 1082 (16%) survivors. The research approvals granted for the study allowed only very limited data to be retained and used for this latter group. By definition their survival status was known (allowing analysis of the primary outcome) but secondary outcome data were missing not at random. Pre-specified sensitivity analyses were undertaken to estimate the bias associated with not having data on up to 33% of the study population. The results of these analyses and their impact on the study conclusions will be discussed.
**Discussion**
The proportion of patients for whom consent was not obtained was higher than had been predicted when the study was designed. The requirement to obtain consent to use data collected in a trial after the intervention is complete and when no further participant involvement is required needs to be challenged. Disclaimer RAPIDO was funded as part of an NIHR Programme Grant for Applied Research. The views expressed are those of the authors and not necessarily those of the NHS, the NIHR or the Department of Health.

### P313

#### Imaging endpoint eligibility in oncology trials: what works and what doesn't

David Raunig
ICON Clinical Research
**Correspondence:** David Raunig
*Trials* 2017, **18(Suppl 1):**P313

**Background**
There is almost nothing that will destroy a relationship with a site than disagreement on the eligibility of a patient. Oncology studies are particularly vulnerable to this problem because site investigators are very interested in saving their patients' lives and may inadvertently be biased toward inclusion. For example, disease free survival requires that a patient have no detectable disease at baseline. If eligibility review done by the site is then sent to central review for efficacy imaging analysis, there is a small but real percentage of patients that will be determined to have had disease at baseline. These patients will, in the final analysis of risk, will be deleted from the analysis since they would have progressed at baseline, an infinite risk. Another example is the requirement in RECIST to have measurable lesions to determine response. Site determination of measurable often does not coincide with the independent reader's assessment of measurable. Central confirmation of eligibility decreases the risk of these patients being included but only if a certain amount of due diligence is paid to the method of confirmation.

**Methods**

Methods to assess eligibility are site alone, site + central, central alone and site with central confirmation. Each method will be reviewed and case studies as well as simulations will show the risks and benefits of each. Case Studies Several anonymized case studies will be used to demonstrate the effects of each of the methods. Additionally, parameters derived from these case studies will be used to simulate clinical trials under different hazard ratios to demonstrate the impact of inappropriate eligibility on the final results.

### P314

#### Methods of approaching general practices for trial participant information: feasibility cluster-randomised trial nested within a large multi-centre cluster-randomised controlled cross-over trial

Denise Forshaw[1], Chris Sutton[1]
[1]University of Central Lancashire
**Correspondence:** Denise Forshaw
*Trials* 2017, **18(Suppl 1):**P314

There is limited evidence as to the time taken by general practices to respond to data requests for individual patients as research participants. In stroke trials, as patient mortality in the first 12 months is high, it is common practice to ascertain participant status (dead/alive) before contacting for follow-up; primarily to avoid emotional distress to relatives. GPs are usually informed that a patient is participating in a trial via a letter sent directly from the admitting NHS Trust. The letter details that they will be contacted at a given time (dictated by data collection time-points) to ascertain patient status and verify address and contact details. We have observed considerable variation in the time taken to reply to requests and significant variation in how practices deal with such requests. Some practices are happy to give the details over the phone following basic checks with the researcher, some practices ask for a copy of the consent form and some practices ask for a covering request by letter to be sent via fax, with varying degrees of success in eventually getting the information requested. Although numbers lost or delayed substantially are relatively low, they may still have an impact on loss of valid outcome data and are resource-intensive but important. It is therefore important to identify the approach that elicits the best response from practices to inform the design of future studies. If checking status is too costly or ineffective, then studies will adopt a pragmatic model of contacting participants directly, which may not be in the best interests of the patient or their families. We have therefore designed a feasibility cluster RCT, nested within a larger trial, using 12 sites and 4 different methods of approach. 1. Telephone contact first and then Fax if requested using the letter format already in use 2. Telephone contact first and then Fax using a new letter format reminding them that they have already received confirmation of consent from their secondary care provider 3. Fax contact first using the letter format already in use and then Telephone contact if no response 4. Fax contact first using the new letter format reminding them that they have already received confirmation of consent from their secondary care provider Three sites were randomised to each of these methods, with all general practices from which a site's participants were recruited being allocated to that method. To reduce differences in initial approach the same member of staff was used for all requests. We have collected details of the amount of extra information or processes requested by the practices and the time interval from request to provision of the information (or to termination of our request). Analysis will be descriptive and inform the feasibility and design of a full nested trial of these methods. Feasibility questions include whether there is the potential to detect an important difference on a key outcome and whether it is feasible to design a trial for which randomisation is by secondary care provider rather than general practice.

### P315

#### Brief consent forms enable rapid enrolment in acute clinical trials: data from the on-going tranexamic acid for hyperacute primary intracerebral haemorrhage (TICH-2) study

Katie Flaherty, Lelia Duley, Zhe Law, Philip M. Bath, Nikola Sprigg
University of Nottingham
**Correspondence:** Katie Flaherty
*Trials* 2017, **18(Suppl 1):**P315

**Background**

Intracerebral haemorrhage (ICH) is often complicated by haematoma expansion (HE) with devastating consequences. The NIHR HTA TICH-2 study is a randomised controlled clinical trial that is testing whether tranexamic acid arrests HE and improves outcome. Typically, stroke treatments have greater efficacy if given early and so delays should be avoided. Obtaining consent in the emergency situation is difficult since many stroke patients lack capacity to consent and relatives are often not present.

**Methods**

Ethics approval was obtained to allow full informed consent or verbal assent (using a brief information sheet) followed by full written consent at a later date. The brief information sheet is used when the therapeutic time window is short and the use of full written consent would inhibit recruitment into the trial. Where patients lacked capacity, approval was obtained to enrol them with permission from a relative, carer or friend acting as legal representative. If no one was available to act as a legal representative, permission could be obtained if two clinicians (one unconnected with the trial) agreed to enrol the patient. Permission from legal representatives could be given using a full information sheet, or the brief information followed by full written consent.

**Results**

Of 1682 patients enrolled, 387 (23%) gave full informed consent and 201 (12%) gave brief verbal assent. Many patients lacked capacity (65%) and were enrolled after proxy consent from a legal representative; full informed relative 720 (43%), brief relative 255 (15%), independent physician 119 (7%). The mean (SD) time from stroke onset to recruitment (in hours) for patients enrolled with full consent were 3.8 (1.6) for patient and 4.0 (1.7) for relative consent; this went down to 3.4 (1.7) for brief patient and 3.6 (1.5) for brief relative. The quickest consent group was independent physician, with an average time to recruitment of 3.2 (1.5) hours.

Use of a range of methods for consent enabled rapid enrollment. Participants unable to consent had dysphasia and higher stroke severity. Thirty nine participants who gave verbal assent died before full written consent could be obtained, and two participants declined to give further consent and later withdrew from the day 90 follow-up; no participants who used brief consent were lost to follow-up.

**Conclusion**

Abbreviated information sheets supporting verbal assent and proxy consent can ensure patients are enrolled rapidly into emergency clinical trials. The use of brief consent or proxy consent did not lead to large numbers of withdrawals or losses to follow-up, thus the use of two stage/or proxy consent for emergency clinical trials should be considered.

### P317

#### Multiply efficient trials: combining multiple trial arms and critical secondary questions increases trial efficiency

Kimberley Goldsmith[1], Peter White[2], Trudie Chalder[1], Michael Sharpe[3], Andrew Pickles[1]
[1]King's College London; [2]Centre for Psychiatry, Wolfson Institute of Preventive Medicine, Barts and the London School of Medicine, Queen Mary University; [3]Psychological Medicine Research, Department of Psychiatry, University of Oxford
**Correspondence:** Kimberley Goldsmith
*Trials* 2017, **18(Suppl 1):**P317

**Background**

Designing explanatory trials to answer additional questions such as how and for whom treatments work should be a priority for improving trial efficiency. Multiple arm trials are also more efficient, as they provide more information about treatments over a shorter time span [1]. We studied the benefits of multiple trial arms and explanatory design using the Pacing, Graded Activity, and Cognitive Behaviour Therapy: A Randomised Evaluation (PACE) trial as an example. This trial studied three complex therapies and a specialised medical care comparison arm for the treatment of chronic fatigue syndrome. The study of how the treatments worked - mediation analysis - was built into the trial design. In terms of mediation, one interest was whether different treatments with some disparate components might vary in mechanism. In other words, might the effects of different treatments on a mediator (a paths or action theories) be associated with different mediator-outcome relationships (b paths or conceptual theories)?

**Methods**

Longitudinal structural equation models (SEM) for mediation were applied to two-arm subsets and the overall four-arm trial dataset to study longitudinal mediation of the effects of the PACE trial treatments. Fear avoidance (FA) was used as an example mediator and physical functioning (PF) as an example outcome [2]. A single model was fitted to the dataset in each case with the pertinent contrasts obtained. Treatment by mediator interaction terms were used to assess differences in mediator-outcome effects (conceptual theories) for different treatments. Informal comparisons of the standard errors were used to assess precision.

**Results**

The multiple arms/explanatory design combination provided both practical and statistical advantages. From the practical point of view: A) the results from several two arm trials were obtained from one trial, B) an explanatory design meant this was true for important secondary analyses as well, C) this particular design allowed for comparisons between active treatments and with the specialised medical care comparison arm. One statistical advantage was increased power. This may have been especially important for the mediation analysis, as there is often low power to detect mediated effects [3] and some useful methods for studying mediation suffer from lower precision. For example, there was 25% gain in the precision of the parameter estimate for the mediator-outcome relationship in the full four arm dataset as compared to the two arm subset. Another statistical feature was the ability to test whether the mediator-outcome effect (conceptual theory) was similar across treatment arms (action theories). This assumption held, and making this assumption allowed for further precision gains.

**Conclusions**

Where indicated and/or sensible, designing trials to answer explanatory questions using a multiple arm design will be more efficient, and provide more statistical power as well as a rich source of information about treatments. Such designs should maximise efficiency for primary outcome comparisons, and provide important information about secondary questions of interest across multiple treatments within a single study.

### P318

#### Analysis strategies for two-level clustering in one arm of a randomised group therapy trial set in Cardiff prison

Rebecca Playle, Michael Robling, Rachel McNamara, Yvonne Moriarty, Zoe Meredith, Hannah John-Evans, Pamela Taylor
Cardiff University
**Correspondence:** Rebecca Playle
*Trials* 2017, **18(Suppl 1):**P318

**Background**

The GASP study (Groups for Alcohol Misusing Short-terms Prisoners) is a randomised trial of an intervention to improve participants' sense of control and motivation to make changes. Men were allocated to the control arm (standard prison regimen) or a programme of nine group sessions over three weeks facilitated by an experienced clinical psychologist and psychology assistant. Clustering by group session is therefore present only in the intervention arm. There were 8 facilitators in total, 5 psychologists and 3 assistants, different pairs of facilitators ran the groups during the study. Further clustering is therefore present by facilitator which varied over the course of the study.

**Objectives**

To compare strategies for the analysis of clustered data where clustering by group and facilitator is only present in the intervention arm.

**Methods**

Recruitment and then randomisation to the intervention and control arms was carried out in small blocks over the course of the study due to a limitation on the maximum size of the group sessions. The primary outcome, Locus of Control of Behaviour (LCB), was collected for all men prior to randomisation and at the end of the group sessions in the intervention arm and an equivalent time point in the control arm. One analysis strategy could therefore involve the creation of control clusters contemporaneously equivalent to the intervention group clusters. A second strategy would be to create clusters of sample size one in the control arm and is the current standard strategy for trials of this design. A third strategy would be to randomly create control arm clusters of equivalent size, and variation in size, to the intervention arm clusters, the 'artificial cluster method'. A forth strategy would be to group all the control men into a single control arm cluster. The primary analysis is a two level general linear model adjusted by baseline LCB. Secondary analysis will include the additional level of facilitator in a three level model if the ICC warrants its inclusion.

**Results**

All group programmes for the GASP study have now been completed and longer term follow-up data collection and data cleaning is underway. Two hundred and thirty eight men were randomised on a 1:1 basis and there were 15 intervention group programmes (cycles) completed over 2 years. The total sample size target was 120 for the primary analysis. Primary outcome LCB data is available for 68 in the intervention arm and 61 in the control arm. Results of the statistical analysis of the primary outcome will be presented discussing any advantages or disadvantages of the analytic strategy employed.

**Conclusions**

The prison environment is a constantly changing and challenging environment for research. Prisoner numbers, prisoner mix, prison transfers, staff to prisoner ratios, access to services and researcher access to prisoners varied during the course of the group cycles, therefore creating contemporaneously equivalent control arm clusters may be preferable to the standard strategy in this trial. A discussion of any bias that may be introduced or controlled for by any one of these methods will also be addressed.

### P319

#### Sensitivity analysis assessing the impact of patient self-reported follow up in the react trial

Holly Tovey[1], Judith Bliss[1], Charles Coombes[2], Gunter von Minckwitz[3], Jan Steffen[3], Lucy Kilburn[1]
[1]The Institute of Cancer Research; [2]Imperial College London; [3]German Breast Group
**Correspondence:** Holly Tovey
*Trials* 2017, **18(Suppl 1):**P319

**Background**

REACT is a phase III, multicentre trial of celecoxib vs placebo in primary breast cancer patients in the UK and Germany. Patients receive blinded treatment for 2 years and are followed up every 6 months, then annually up to 10 years for Disease Free Survival (DFS). This creates burden on the site and the patient. For some sites it has been necessary to move towards self-reported follow up (FU) via questionnaires to reduce the burden. Not all sites have taken up the opportunity and it is not an option in the UK. There was concern within the trial committees that self-reported FU could result in a loss of data or accuracy, producing bias in the principal analysis. It was agreed that the validity of self-reported FU compared to conventional centre-based methods should be retrospectively assessed within the trial.

**Methods**

A univariate Cox-model was fitted for time to first DFS event with FU method as a time-varying covariate; observations were split at the time a patient consented to self-reported FU unless an event occurred prior to this. It was assumed that following consent patients could not revert back to conventional FU. The model was repeated excluding the first 2 years of FU and a landmark analysis was also carried out looking at 0–2 years, 2–5 years and 5 years + separately. This was done to reduce bias from early events; patients cannot switch to self-reported FU until they have completed treatment (usually at 2 years) so events prior to this could only occur on conventional FU. For each analysis the hazard ratio for FU type and event rates in each group were calculated. Analysis was repeated separately for each type of DFS event; local relapse (LR), distant relapse (DR) and death.

**Results**

FU data was more complete for self-reported FU patients. No significant difference in event rate was observed for first DFS event. When looking at event types separately, death rates for self-reported patients were significantly lower compared to conventional patients (HR = 0.31, 95% CI = 0.17-0.72). Although not statistically significant DR rates were lower for self-reported FU (HR = 0.62, 95% CI = 0.22-1.72) and LR rates were higher for self-reported FU (HR = 3.39, 95% CI = 0.72-15.87).

**Conclusions**

Within REACT self-reported follow up is a suitable alternative to collect data for the primary endpoint of DFS. However for accurate reporting of secondary endpoints a more robust method for reporting of deaths needs to be considered. Further research is also required into whether patients reliably report the correct type of relapse. When reviewing impact of FU methods which can change it is important to use a landmark analysis or other methods to reduce the risk of bias if it's possible for events to occur before the method can change (e.g. If the method can't change until treatment is complete). For multi-event outcomes it is also important to analyse separately by event type in case the overall result masks a difference between event types. This is particularly an issue when secondary endpoints use single events.

### P320

#### DMC report production: considering a risk-based approach to quality control

Amber Randall, Bill Coar
Axio Research
**Correspondence: Amber** Randall
*Trials* 2017, **18(Suppl 1):**P320

Quality control is fundamental to ensuring both correct results and sound interpretation of clinical trial data. Most QC procedures are a function of regulatory requirements, industry standards, and corporate philosophies. However, no one should underestimate the importance of independent, thoughtful consideration of relevance and impact at each step in the process from data collection through analysis.
Good QC goes far beyond just reviewing individual results and should also consider monitoring data throughout the course of a study. In particular, QC is essential when supporting a Data Monitoring Committee. Given the nature of interim and incomplete data, inherent challenges exist when it comes to generation of DMC reports. Many of the usual practices associated with quality control need to be adapted to accommodate the repetitive nature of DMC review on accumulating data that may have outstanding queries.
This presentation will explore adaptations to a typically rigid QC process that are necessary when reviewing interim/incomplete data. Such adaptations focus on a risk-based approach to QC to ensure that a DMC can make informed decisions with more confidence in the data and programming.

### P321

#### The impact of sample size re-estimation using baseline ICC in cluster randomized trials: 3 case studies

Kaleab Abebe[1], Kelley A. Jones[1], Elizabeth Miller[1], Daniel J. Tancredi[2]
[1]University of Pittsburgh; [2]University of California Davis
**Correspondence:** Kaleab Abebe
*Trials* 2017, **18(Suppl 1):**P321

The Coaching Boys into Men (CBIM) Middle School study is a cluster-randomized trial of a middle school gender violence prevention program. The primary goal is to examine the effectiveness of a program for the primary prevention of adolescent relationship abuse (ARA) and sexual violence among middle school sports teams in Western Pennsylvania. Initially, 26 middle schools were randomized to receive either a) the CBIM intervention, which trains athletic coaches by providing concrete strategies for discussing sexual violence as well as how to respond to disrespectful behaviors, or b) control (standard coaching). According to initial sample planning assumptions, this would yield 1980 students and provide 80% power to detect meaningful differences in the primary outcome, positive bystander behavior.
In the fall of 2015, it was noted that within-cluster recruitment was slower than expected, so the decision was made to increase the number of clusters to 40. In Spring 2016, available baseline data was used to estimate the intra-cluster correlation coefficient (ICC) in order to gauge whether the initial assumption of a 0.02 ICC was correct. With an updated baseline ICC of 0.007 (95% CI: 0.0001-0.433) the necessary sample size decreased to 908 students. While favorable, this left the study team with the following choice: a) assume the updated ICC was closer to truth and proceed with the lower, more favorable sample size; or b) assume the original ICC and continue with the more conservative sample size of 1300. Given the instability of the ICC estimate, the latter decision was made, but it raised the question of whether previous cluster RCTs in adolescent medicine may have benefited from sample size re-estimation using baseline ICC.
In this talk, we will review sample size re-estimation methods for cluster RCTs and describe three completed studies: CBIM High School, SHARP (School Health Center Healthy Adolescent Relationships Program), and ARCHES (Addressing Reproductive Coercion in Health Settings). After providing an overview of the study designs and primary outcomes, we will discuss the initial sample size calculations with the assumed ICC as well as the final ICC at the end of study. Additionally, we will highlight the impact of post-hoc sample size re-estimation methods on the target sample size as well as the primary results.

### P322

#### Point and interval estimation for predicting individual patients treatment effect based on randomized clinical trial data

Kukatharmini Tharmaratnam, Thomas Jaki
Lancaster University
**Correspondence:** Kukatharmini Tharmaratnam
*Trials* 2017, **18(Suppl 1):**P322

### Background

Individual populations within a research study are typically heterogeneous. Characteristics such as genetics, disease etiology and severity vary between individuals and potentially affect the response to treatment. Treatment effectiveness is, however, typically assessed using the average treatment effect or at most treatment effect within (prespecified) subgroups. Recently, developed approaches allow researchers to predict an individual's response to treatment allowing individualized treatment instead of relying on averages from a group or subgroup. Lamont et al. (2016) de ne Predicted Individual Treatment Effects (PITE) and introduce the framework of PITE. The objective of this work is to propose, derive and evaluate prediction interval for PITE.

### Methods

The PITE can be estimated utilizing multiple imputation to obtain treatment effect estimates on a patient level. Based on this approach we develop a method to compute prediction intervals on an individual patient level. To ensure adequate estimate of the variability which is required to obtain such intervals, we investigate different model selection methods.

### Results

We used continuous response variable and binary covariates to fit regression model in all simulation studies. The simulation results show that, using no variable selection leads to an under estimation of the variability and hence under coverage. But the prediction intervals achieve good coverage when we use variable selection methods stepbic or Lasso. Lasso variable selection method works better even with small sample sizes. We have considered different set of selected variables used to get the PITE, those are separately selected variables from each arm, union of selected variables from both arm and the selected variables from joint model. Our simulation results indicates all of these sets are perform well. In practice, the variables selected from joint model would be more reasonable to use because we will have new patients from any of the arms. We considered uncorrelated, perfectly correlated and partially correlated responses in the simulation studies. Our proposed approach performs well with all of these correlation structure. To illustrate proposed method, we used PRO-ACT data (http://nctu.partners.org/proact) in ALS clinical trials. We used patients with equal number of individuals (n = 1500) from the placebo and active treatments group. We used the response variable ALSFRS slope as used in Kuffner et al.(2015) compute from repeated measure of ALSFRS score (ALS Functional Rating Scale) and covariates are several baseline information, Age, Gender, Ethnicity, etc.… for each patient. We applied lasso regression and selected the variables Gender, Age, Race, onset-delta and onset-site to get good PITE for each individuals. We calculate 95% prediction intervals for each patients. The estimated PITE and its intervals are reasonably good. Discussion: Our proposed approach to find the prediction interval for PITE performs well in the simulation studies and real data example. We could use other type of response variables and covariates in the model to estimate PITE and also we may use interaction models and more complex mixed models to get PITE in future work.

Kuffner et al. (2015), Crowdsourced analysis of clinical trial data to predict amyotrophic lateral sclerosis progression. Nature Biotechnology, 33:51–57.

Lamont et al. (2016), Identification of predicted individual treatment effects (PITE) in randomized clinical trials. Statistical Methods in Medical Research.

### P323

#### An evaluation of statistical methods for predicting timelines for reaching target number of events in clinical trials with time-to-event endpoints

Emma Clark[1], Hans-Joachim Helms[2], Sven Stanzel[2], Fan Xia[3]

[1]Roche Products Ltd; [2]Hoffmann-La Roche Ltd; [3]Roche Product Development

**Correspondence:** Emma Clark

*Trials* 2017, **18(Suppl 1):**P323

In clinical trials with time-to-event outcomes, interim or final analyses are often planned after a pre-defined target number of events has been reached. At the planning stage of such studies, the number of events required for statistical analysis and predictions of the expected date when this target number of events will be reached, are typically based on protocol assumptions and conducted by use of a simple parametric model. A blinded re-evaluation of these predictions is recommended to obtain more accurate predictions as the trial progresses and events accumulate. Different statistical approaches have been proposed in the literature for making such predictions, including parametric approaches assuming smooth underlying survival functions, nonparametric approaches and hybrid methods applying a non-parametric model where data are available, complemented with a parametric tail for regions where no data are yet available. Factors such as study design and ratio of number of events in relation to sample size can impact the model estimates derived from the various statistical methods, thereby making the choice of the optimal prediction method for a particular study a key decision which can influence the reliability of the predictions. We report results obtained from a systematic comparison of the different methods via simulation studies. The point estimates of the predicted analysis times and number of events, along with their variability as measured by confidence interval, are investigated under varying study scenarios and findings are discussed.

Keywords: time-to-event, event prediction, parametric, hybrid.

### P325

#### A comparison of statistical approaches for analysing missing longitudinal patient reported outcome data in randomised controlled trials

Ines Rombach[1], Alastair Gray[1], Crispin Jenkinson[2], Oliver Rivero-Arias[3]

[1]Health Economics Research Centre, University of Oxford; [2]Health Services Research Unit, University of Oxford; [3]National Perinatal Epidemiology Unit, University of Oxford

**Correspondence: Ines** Rombach

*Trials* 2017, **18(Suppl 1):**P325

### Background

Missing data are a potential source of bias in the results of randomised controlled trials (RCTs), which can have a negative impact on guidance derived from them, and ultimately patient care. However, missing data are generally unavoidable in clinical research, particularly in patient reported outcome measures (PROMs). For longitudinally collected outcomes, often only a small subset of participant will have complete data for all relevant time points. Multilevel mixed-effects linear regression models are commonly used to analyse longitudinal data. A number of methods are available to handle missing data in such analyses, including maximum likelihood (ML), multiple imputation (MI) and inverse probability weighting (IPW). Direct comparisons of such methods for missing proms data in RCT settings are needed to ensure the bias introduced in such analyses is minimised.

### Objective

To compare ML, MI and IPW approaches for handling missing longitudinal proms data in RCTs.

### Methods

Real-life missing data following missing at random patterns were simulated within the follow-up of an RCT using the Oxford Knee Score. Datasets of sample sizes ranging from 100 to 1,000 with missing proms outcome data in 10% to 40% of participants were simulated. Both intermittently missing data and monotone missing data patterns were considered. Missing data was addressed using ML, MI and IPW. Performance of the different approaches was assessed by the bias introduced in the treatment coefficients from multilevel mixed-effects linear regression models obtained for 1000 simulations. Root mean square errors (RMSE) and mean absolute errors (MAE) were used as performance parameters.

### Results

Non-convergence issues were observed for the IPW approach for small sample sizes. Complex MI models needed to be simplified to obtain valid results for combinations of small sample sizes and large proportions of missing data. Bias in the treatment coefficient increased both with decreasing sample size and increasing proportions of missing data. MI and ML performed similarly when similar variables were included in both the imputation and analysis model, and when the imputation model was restricted to baseline variables. However, MI was less biased than ML when additional post-randomisation data were used in the imputation model. Both approaches were less biased when follow-up data was missing intermittently compared to monotone missing data scenarios due to drop-out. IPW introduced more bias in the model results than both ML and MI across all sample size and missing data scenarios.

**Conclusions**

MI can offer benefits over ML for handling missing longitudinal proms data when additional post-randomisation information is available. For RCTs with sample sizes up to 1000, the use of IPW is not recommended to handle missing data. The findings also demonstrate the importance of minimising missing data and continued data collection beyond missed appointments to inform the analysis and imputation models. The results presented in this presentation focus on missing at random mechanisms, and sensitivity analyses to investigate the effect of other missing data mechanisms remains imperative.

### P326

#### Improving precision by adjusting for prognostic baseline variables in randomized trials with binary outcomes, without regression model assumptions

Jon Steingrimsson, Daniel F. Hanley, Michael Rosenblum
Johns Hopkins University
**Correspondence:** Jon Steingrimsson
*Trials* 2017, **18(Suppl 1):**P326

This abstract is not included here as it has already been published.

### P327

#### Comparing different ways of calculating sample size for a randomised controlled trial using baseline and post-intervention measurements

Lei Clifton[1], Jacqueline Birks[1], David A. Clifton[2]
[1]Centre for Statistics in Medicine (CSM), Oxford University; [2]Institute of Biomedical Engineering, Department of Engineering Science, University of Oxford
**Correspondence:** Lei Clifton
*Trials* 2017, **18(Suppl 1):**P327

**Background**

In this paper, we compare different methods of calculating sample sizes for comparing two independent means of a continuous outcome with baseline and post-intervention measurements of a randomised controlled trial (RCT). Sample size calculations typically use published results from similar trials, and we illustrate the different methods using published results from the MOSAIC trial.

**Methods**

The methods we discuss are suitable for sample size calculation using a continuous outcome measure. Suppose the primary continuous outcome measure is Y, with Y_0 and Y_1 denoting Y at baseline and post-intervention, respectively. Let r denote the correlation coefficient between Y_0 and Y_1. We discuss the following two factors:

1. The choice of the primary outcome measure: post-intervention measure Y_1 vs. Change from baseline (i.e. Y_1-Y_0).
2. The choice of statistical methods for sample size calculation for two independent means: t-test without using r vs. Analysis of covariance (ANCOVA) using r.

We show how to use the Variance Sum Law to derive r between Y_0 and Y_1, and then how to use the derived r to calculate sample size by ANCOVA. We discuss the assumptions of the ANCOVA method and its implications for the trial design.

We discuss the existing research on how the value of correlation coefficient r influences the sample size, using change score from baseline vs. Post-intervention score alone.

We perform a sensitivity analysis on different values of r to show the effect of the strength of the correlation on the sample size by ANCOVA. The correlation between the post-intervention and baseline measure is likely to reduce as the duration prolongs; therefore the duration between the post-intervention and baseline need to be taken into account For example, the MOSAIC trial reported the SF-36 energy/vitality score at 6 months, for which we have derived r = 0.7. If the post-treatment time point of this measure in the planned RCT is at 12 months, then we will need to reduce the value of r for the sample size calculation. The resulting sample sizes are shown by the sensitivity analysis in this paper.

**Conclusions**

ANCOVA allows efficient sample size calculation by utilising the correlation between the baseline and post-intervention measurements; however, one must be aware of its implications and consider factors such as duration of the intervention. In comparison, using a t-test produces a more conservative (i.e. Larger) sample size than using ANCOVA.

In the situation when sample size is calculated by a t-test instead of ANCOVA, sample size using change score from baseline can be smaller or larger than that using post-intervention score alone, depending on the value of the correlation coefficient r. The choice of the outcome measure should be driven by clinical knowledge instead of a mere pursuit of small sample size.

We advocate reporting the standard error (SE) of the mean change between the baseline and post-intervention measurements, as did the MOSAIC publication. It provides insight into the correlation between the baseline and post-intervention measurements, and therefore allows the sample size to be calculated and compared in different ways.

### P328

#### A response-adaptive trial to determine the optimal IL-2 dose-frequency to achieve multiple target increases on regulatory T cells in type 1 diabetes: dilfrequency study

James Howlett[1], Adrian Mander[1], Simon Bond[2], Frank Waldron-Lynch[3]
[1]MRC Biostatistics Unit Hub for Trials Methodology Research; [2]Cambridge Clinical Trials Unit, Cambridge University Hospitals NHS Foundation Trust; [3]Division of Experimental Medicine & Immunotherapeutics, Department of Medicine, University of Cambridge
**Correspondence:** James Howlett
*Trials* 2017, **18(Suppl 1):**P328

**Background**

In type 1 diabetes (T1D) there is a deficiency in the interleukin 2 (IL-2) pathway leading to a loss of regulatory T cell (Treg) function. Dilfrequency aims to determine the optimal dosing regimen of ultra-low dose recombinant IL-2 (aldesleukin) to improve Treg function while limiting the activation of CD4 T effector cells (Teff) in participants with T1D.

**Methods**

Thirty-six participants with T1D were administered repeat doses of aldesleukin with the aim of establishing the optimal dose-frequency to deliver drug to increase Tregs, CD25 (Subunit of the IL-2 receptor) expression on Tregs, whilst minimising the increase in Teffs. There was an initial learning phase with six pairs of participants, each pair receiving one of six preassigned dose-frequencies from 0.09-0.47x106 IU/m2 and 2–14 days, in order to model the dose-frequency response. At the first interim analysis following the learning phase, the target increases (30% Treg, 25% CD25, 0% Teff) for each of the endpoints were selected by the dose frequency committee. The subsequent 3 groups of 8 participants were administered dose-frequencies based on the results from statistical analyses of all data from previous groups. When allocating treatment regimens, consideration was given to the probability of the predicted increases fall within the target ranges as well as the distance the predicted increases are from the targets for each dose-frequency.

**Results**

We found at each pre-planned interim analyses, that the optimal dose-frequency was estimated with increasing accuracy, thereby allowing more participants to be allocated dose-frequencies close to the optimal than would be possible in a non-adaptive design. The results from the final interim analysis suggest that the optimal aldesleukin dose to maintain steady state increases in Tregs and CD25 expression is between 0.20 x106 IU/m2 and 0.32x106 IU/m2 at a frequency of every 3 days. Results from the final analysis are ongoing and will be presented when available.

### P329

#### Statistical issues in data monitoring of clinical trials that incorporate assessment of pregnancy loss

Lee Middleton, Konstantinos Tryposkiadis, Versha Cheed
University of Birmingham
**Correspondence:** Lee Middleton
*Trials* 2017, **18(Suppl 1):**P329

**Background**
Independent data monitoring in clinical trials of pregnant women where successful pregnancy is one of the outcomes is hugely important given the sensitive nature of these studies. Recommendations regarding early stopping or protocol modification can only be made with appropriate data available. Our experience in the NIHR-funded TABLET, PRISM and C-STICH studies suggests certain elements of data monitoring - namely oversight of interim estimates of the overall event rate and efficacy estimates - require careful consideration and forward planning.
**Methods**
Primary outcomes for the aforementioned studies are variations of successful pregnancy (either live birth > 34 weeks or pregnancy loss up to the first week of life) and are planned to be analysed at the end of the trial as dichotomous outcomes (success/fail) through the generation of relative risks and associated confidence intervals using standard methodology. However, interim assessment during the recruitment period requires further thought as the failure rate is temporarily inflated due to the fact that treatment failures (e.g. Miscarriage or still births) are ?A3B2 show $132#?>accumulated sooner than successful outcomes (live births). In these circumstances, the Trial Statistician and Data Monitoring Committee need to consider their approach in how to monitor both the sample size assumptions and interim estimates of efficacy.
Three approaches to this problem are apparent: i) analyse any participants that have currently completed the study accepting that the success rate is temporarily reduced; ii) analyse a full cohort of patients that have completed the study using a pre-defined cut-off period, e.g. Only those randomised greater than nine months previously; and iii) switch analytical methods during the interim period to utilise survival analysis methodology (e.g. Kaplan-Meier, Cox Proportional Hazards), censoring participants at the point of last know follow-up if not yet completed the study.
**Results**
We will show that: option i) will not provide appropriate estimates of current success rates and could potentially bias interim efficacy estimates if there is a difference between groups in early failures; option ii) will provide appropriate estimates of current live birth rate but will limit the amount of data available for analysis and therefore potentially hamper decision-making; and option iii) will utilise all available data for analysis of efficacy and also provide appropriate interim estimates of the live birth-rate.
**Discussion**
In studies of this type, Trial Statisticians should consider planning the use of survival analysis methodology during the interim period regardless of planned final analysis methods to enable provision of informative estimates to independent Data Monitoring Committees.

### P330

#### Adjusting trial analyses for continuous stratification variables

Tom Morris, Shaun Barber, Cassey Brookes
Leicester Clinical Trials Unit
*Trials* 2017, **18(Suppl 1):**P330

**Background**
Randomisation in clinical trials is often performed using permuted blocks, in which the randomisation of a patient is dependent on the randomisation of the other patients in the same block. This method guarantees that similar numbers of patients are allocated to each arm, within limits determined by the block size(s). This type of randomisation can be stratified, so that balance is achieved within the strata, rather than overall. The analysis of data from such trials should be adjusted for the stratification variables [1]. Stratification variables must be categorical, and therefore, if randomisation is to be stratified on a continuous variable, the variable must first be split into categories (e.g. BMI is often categorised as underweight/normal, overweight, or obese). It is well known that a continuous covariate should not be categorised in an analysis (or anywhere else) without good reason. The question this investigation aims to answer is: should the analysis of a trial in which randomisation has been stratified on a categorised continuous variable be adjusted for the categorisation, or the underlying continuous variable?
**Methods**
Simulations were performed to assess the effect on the significance level and power of analyses in which the (continuous) outcome depends on a continuous covariate, and the randomisation has been stratified on a categorisation of this continuous variable. Three different relationships between the variable and the outcome were tested: linear, non-linear, and none, where the non-linear relationship meant that different slopes were used for each level of the (binary) categorisation. The slopes in the linear and non-linear cases were varied in magnitude. For each simulation, 10,000 data sets were generated, and each was analysed in two models, one adjusting for the continuous variable and the other for the categorisation. The simulations were also conducted with unstratified randomisation.
**Results**
When the randomisation was stratified, the nominal power and significance levels were maintained unconditionally. When the randomisation was unstratified, the nominal power and significance levels were maintained except when both (i) the analysis was adjusted for the categorisation; and (ii) the effect of the underlying continuous variable on the outcome was large.
**Conclusions**
When randomisation has been stratified on a categorised continuous variable, there is no difference between adjusting for the underlying continuous variable and adjusting for the categorisation. When the continuous variable has a large effect on the outcome and the variable has not been stratified on, power is lost if the analysis is adjusted for the categorisation as opposed to the continuous variable. It is safer to always adjust for the underlying continuous variable.

**Reference**
[1] Improper analysis of trials randomised using stratified blocks or minimisation, Brennan Kahan, Tim Morris, Statist. Med. 2012, 31 328–340.

### P331

#### Understanding, quantifying and reducing recruitment bias in cluster randomized trials

Karla Diazordaz
LSHTM
*Trials* 2017, **18(Suppl 1):**P331

**Background**
Cluster randomized trials (CRTs) are becoming increasingly common in primary care and public health. Often clusters are recruited and randomised before suitable individuals are identified and recruited to participate or consent to data collection, leading to possible bias if the recruited/consented individuals differ systematically from those who do not. It is often thought that this biases may be particularly important when recruitment rates vary across clusters and intervention arms.
**Aim**
The aim of this work is to use formal causal inference techniques to help trial researchers understand when the treatment effect estimate will be biased due to recruitment issues, by using Directed Acyclic Graphs (DAGs) and d-separation, and to give a measure of the size of these biases. Methods: We considered several situations that could lead to recruitment bias in a CRT, namely where individual recruitment is associated with treatment allocation and/or another variable (either measured or unmeasured), and use d-separation to show when the treatment effect is biased due to the associations induced by conditioning the analyses to those individuals in the population who are recruited (or consented). We also conducted a simulation study in which we varied the magnitude of association among the individual-level covariate, treatment allocation at the cluster-level, the probability of recruitment, and the outcome variable. We considered two different individual recruitment rates: 50% and 75%.

**Results**

We have formal results showing when there is bias present, for example when the probability of recruitment and the outcome both depend on a common individual-level covariate, and these associations are differential by treatment allocated. In the simulations, we found that where this bias is present, it can be over half a standard deviation of the true causal treatment effect.

**Conclusions**

Recruitment bias in CRTs happens when recruitment/consent is differential by treatment allocation and associated to a variable which is also associated with the outcome. If this variable is observed, we can control for it in the models, and our treatment effect will be unbiased. However, if the variable is unobserved, the treatment effect will be biased. This bias is small when recruitment rates are high. The possibility that recruitment is associated with treatment assignment can be minimised by identifying/recruiting individuals prior to cluster randomisation, or by blinding recruiters and potential participants as much as possible). In addition, if researchers know which individual characteristic is likely to be associated with the systematic differences in recruitment/consent, measuring this and adjusting for it, can mitigate the recruitment bias.

### P333

#### A bayesian framework to address missing not at random data in longitudinal studies with multiple types of missingness

Alexina Mason[1], Richard Grieve[1], Anthony C. Gordon[2], James A. Russell[3], Simon Walker[4], Nick Paton[5], James Carpenter[1], Manuel Gomes[1]
[1]London School of Hygiene and Tropical Medicine; [2]Imperial College London; [3]University of British Columbia; [4]University of York; [5]National University of Singapore
**Correspondence:** Alexina Mason
*Trials* 2017, **18(Suppl 1):**P333

Missing data can be a serious problem in longitudinal studies because of the increased chance of drop-out and other non-response across the multiple time points, and can be particularly challenging when there are different causes of the missing values. For instance, the reasons that patients completely drop-out of the study (monotone missingness) may be very different from those for failing to attend a particular follow-up appointment (intermittent missingness). Also, for some types of missingness, it is often plausible to assume that data may be "missing not at random" (MNAR), i.e. after conditioning on the observed data, the probability of missing data may depend on the underlying unobserved values. For example, in critical care trials the collection of hourly/daily biomedical data may take place at the local physician's discretion and lead to intermittent missingness that is related to the severity of the patient's illness. Faced with MNAR data, missing data guidelines recommend sensitivity analysis to allow for alternative assumptions about the missing data. A useful approach is to use selection models, which specify a marginal distribution for the outcomes (analysis model) and a conditional distribution for the missing value indicators given the outcomes (missingness model). Selection models are particularly attractive in longitudinal studies, because they can recognise that the missing data mechanism may be distinct across the different types of missingness. This research proposes flexible Bayesian selection models for assessing the robustness of trial results to alternative realistic assumptions about the different forms of missingness. In particular, we consider i) the implications of different model choices to allow for complex longitudinal data structures and ii) the incorporation of clinical expert knowledge about the reasons for the missing values through informative priors in the missingness model. We illustrate the methods using two examples: the Vasopressin and Septic Shock Trial (VASST) and the Protease Inhibitor Monotherapy Versus Ongoing Triple Therapy Trial (PIVOT). For VASST, we reanalyse the cardiac index data, collected at baseline and 9 subsequent time-points over the following 96 hours. Monitoring started after baseline for a third of the patients and was discontinued as a result of both death and recovery. For PIVOT, our interest is in the health-related quality of life outcome, which was collected every 12 weeks over a 3-year period, but suffered from substantial intermittent (35%) and monotone (20%) missingness in both arms. For each outcome, we compare the results from alternative assumptions about the longitudinal missing data mechanisms with the published trial results and assess the implications for decision uncertainty. As an example, provisional results from the sensitivity analysis for VASST find that the average cardiac index over time was 9% higher for patients treated with vasopressin compared with those treated with norepinephrine (95% credible interval: 1%-17%), whereas the original analysis reported no difference. We conclude that this approach to sensitivity analysis provides a flexible framework to assess the implications of the missing data for the trial conclusions.

### P334

#### How and when do competing risks influence results from clinical trials?

John Gregson
London Schoole of Hygiene and Tropical Medicine
*Trials* 2017, **18(Suppl 1):**P334

**Background**

A competing risk is an event that prevents an event of interest, such as a primary trial outcome, from occurring. The most common competing risk is death. Ignoring a competing risk in the analysis of a trial results in invalid estimates of the cumulative incidence (absolute risk) of the event of interest. Ignoring competing risks can also result in invalid comparisons between treatment and control groups, for example by biasing the estimate of the hazard ratio.

**Methods**

We reviewed currently methods for dealing with competing risks. The two most commonly used methods were the Fine and Gray model and cause-specific hazards models. We aimed to illustrate the effect of competing risks on estimates of cumulative incidence or estimates or hazard ratios. We aimed to characterise the scenarios where bias is most likely to occur and most likely to be large.

We used data from 3 large Phase III randomised clinical trials in cardiovascular disease: EMPHASIS-HF, EPHESUS and RALES. We chose heart failure hospitalisation as the event of interest and cardiovascular death as the competing event.

**Results**

Cause-specific hazards over-estimate cumulative incidence, whereas the Fine and Gray method correctly adjusts estimates of cumulative incidence to take into account competing risk.

Both cause-specific hazards models and Fine and Gray models give biased estimates of the hazard ratio for treatment effect. When using the cause-specific hazards model, the likely size of the bias was small or moderate in the examples we studied, but the bias was larger when using the Fine and Gray model. Competing risks caused larger biases when the event occurred in larger numbers of patients; occurred earlier during follow up; or occurred more frequently in either the treatment or control group.

**Conclusions**

The cumulative incidence of a primary outcome can be accurately estimated using the Fine and Gray method. However, when estimating the hazard ratio for treatment effect of an event of interest, current methods do not adequately deal with competing risks.

### P335

#### Improving Kaplan – Meier graphs: better presentation of numbers-at-risk, cumulative events and measures of uncertainty

Matthew Sydes[1], Christopher C. Jarvis[2], Babak Choodari-Oskooei[1], Patrick P. J. Phillips[1], Tim P. Morris[1]
[1]MRC Clinical Trials Unit at UCL, Institute of Clinical Trials Methodology, UCL, London, UK; MRC London Hub for Trials Methodology Research, London, UK; [2]London School of Hygiene and Tropical Medicine, London, UK; MRC London Hub for Trials Methodology Research, London, UK
**Correspondence:** Matthew Sydes
*Trials* 2017, **18(Suppl 1):**P335

##### Background
Kaplan – Meier (KM) graphs are the standard approach for depicting outcomes and risks over time for time-to-event outcome measures, including, for example, survival-based outcome measures which are widely used in many disease areas. In the context of clinical trials using these outcome measures, a KM graph is ubiquitous, and is intended to provide a visual representation of any difference between groups or lack thereof and is therefore critical to the interpretation and impact of the trial results. We believe, however, that the standard version of KM graphs can sometimes mislead. One challenge is that the number of patients contributing information decreases as time increases, but the eye is naturally drawn to the right-hand side of the graph where there are fewer data. Another challenge is the uncertainty in the data underpinning the lines. There is no widely agreed way to depict this information, and is insufficiently clearly presented in most journals' graphs.
#### Methods
We explored a series of ways of modifying the KM graph with two objectives: (1) clearly and accurately representing the numbers censored, experiencing events, and still 'at risk', and (2) displaying uncertainty. We included combinations of often-used basics, such as censoring marks and simple risk tables, to more sophisticated risk tables, companion risk-and-event graphs, area shading graphs which represent at-risk populations, and re-construction of the KM lines themselves with sampling. We used trial data to illustrate the strengths and weaknesses of each possible approach. An international survey is in development which will seek responses during winter 2016 17 from people with a wide range of relevant perspectives, including statisticians, clinicians, journal editors and regulators. A fun, supplemental interactive vote would be undertaken on-site during the conference.
#### Results
Several ways to improve depictions of survival data will be presented on the poster. Results of the survey will be presented at the meeting. We will summarise the strengths and weaknesses.
#### Potential Impact
There is potential to improve the presentation of KM graphs and, furthermore, to convey more information about the results of clinical trials. However, implementation in manuscripts would likely depend on the willingness of editors to make the necessary space.
#### Discussion
If there is agreement on a new standard which is not yet routinely available in the major statistical packages, work will be required to make these routinely and simply available.

### P336

#### Conditionally unbiased estimation in two-stage adaptive trials with unknown variances

David Robertson[1], Ekkehard Glimm[2]
[1]MRC Biostatistics Unit; [2]Novartis Pharma AG
**Correspondence:** David Robertson
*Trials* 2017, **18(Suppl 1):**P336

Two-stage adaptive trial designs offer an efficient way of selecting and validating multiple candidate treatments within a single trial. A common strategy is to select the best performing treatment (according to some ranking criteria) after an interim analysis, and to then validate its properties in an independent sample in the second stage. However, selecting and ranking candidates in this way can induce bias into the naive estimates that combine data from both stages. If the selection rules are not properly taken into account by the estimation strategy, then intuitively one might expect overly optimistic estimates of the treatment effect of the selected candidate, given that it had to perform 'well' in the first stage in order to proceed to the second stage.
To efficiently and completely correct for selection bias in adaptive two-stage clinical trials, uniformly minimum variance conditionally unbiased estimators (UMVCUEs) have been derived for a variety of trial designs with normally distributed data. However, a common assumption is that the variances are known exactly, which is unlikely to be the case in clinical practice.
In this paper, we extend the work of Cohen and Sackrowitz (Statistics & Probability Letters 1989), who proposed an UMVCUE for the best performing candidate in the normal setting with a common unknown variance, but only when the first stage sample sizes are all equal and the second stage sample size is equal to one.
Our extension allows for arbitrary first and second stage sample sizes for the different treatment arms, and can also be used to estimate the outcome measure of the j-th best candidate out of k. We show through a simulation study that the UMVCUE that assumes a known variance and estimates it from the trial data is no longer unbiased, and will have a higher mean squared error than our new estimator if the variance is overestimated.

### P337

#### Issues with over-fitting in predictive models produced for stratified medicine: a case study on an ovarian cancer trial

Meredith Martyn[1], Xinxue Liu[2], Charlotte Wilhelm-Benartzi[3], Robert Brown[4], Deborah Ashby[2]
[1]Imperial College London; [2]Imperial College London, School of Public Health, Imperial Clinical Trials Unit; [3]Cardiff University, College of Biomedical & Life Sciences, Centre for Trials Research; [4]Imperial College London, Department of Surgery & Cancer, Division of Cancer
**Correspondence:** Meredith Martyn
*Trials* 2017, **18(Suppl 1):**P337

##### Background
Clinical trials are valuable resources for biomarker exploration as prospectively collected data and experimental designs minimise bias. Models using multiple biomarkers to predict therapeutic response are of particular interest; however, the high-dimensional, small datasets come with challenges. This study aimed to highlight over-fitting issues with producing predictive models using commonly-used methods for Stratified Medicine in typical clinical trial datasets, using an ovarian cancer trial derived dataset as a case study.
#### Methods
Variable selection methods were performed on SCOTROC4 trial data collected from Scottish Gynecologic Cancer Trials Group. The original cohort included 964 patients, of which 155 patients had both available protein expression data in tumour samples assessed by independent scorers, and evaluable CA125 data which monitored patients' therapeutic response. Following clinical consultation, response was defined as >50% reduction in CA125 baseline post-treatment. A pre-selection method to improve variable selection efficiency reduced 26 candidate proteins to 6: cycline E, SENP2, p53, folr2, larp1 and Ki-67. Backwards selection (BS), Akaike Information Criterion (AIC) and LASSO methods were then applied to create predictive models. Accuracy (sensitivity and specificity) of models was assessed through receiver operating characteristic (ROC) curve and area under the curve (AUC). Discrimination ability was assessed through box-and-whisker plots of predicted probability of responding/non-responding groups (127 and 28 patients respectively). 10-fold stratified cross-validation was applied to BS and AIC to control for over-fitting. Performance ability from the full model, BS, AIC, LASSO, cross-validated BS and cross-validated AIC were compared. To assess clinical usefulness, Positive predictive value (PPV) and negative predictive value (NPV) were calculated by extracting accuracy values from the most accurate model and prevalence of therapeutic response from the original cohort.

**Result**
The full model, BS, AIC and LASSO produced similar performance levels of accuracy (AUC = 0.80, 0.78, 0.80, 0.80). Discriminative ability was also similar, as 75% of distributions between responding and non-responding patients in box-and-whisker plots were distinctly different from each other. LASSO demonstrated advantageous precision in discrimination ability. High correlation between the full model, BS, AIC and LASSO models predictive probability (r ranged from 0.8-1) suggested over-fitting in models produced by these variable selection methods. This was supported by the substantial drop in accuracy once BS and AIC models were cross-validated (AUC = 0.57, 0.54 respectively). Cross-validated models showed limited ability to distinguish between responding/non-responding patients. PPV and NPV calculations implied that 10% of patients in this dataset predicted as responders would not respond to therapy, and 55% patients who would be predicted as non-responders would respond to therapy using the most optimal sensitivity and specificity values from the full model (70%, 75%) assuming the prevalence of response is 77.4%.
**Conclusion**
Evidence of over-fitting issues were present in all variable selection methods, including LASSO which supposedly controls for over-fitting within its algorithm. LASSO proved advantageous with its enhanced precision in dichotomising patients, however, NPV and PPV values suggested that a clinically useful model is unlikely to be found unless a dataset is large, or odds ratios of biomarkers added in models are extreme.

### P338
### How are researchers handling missing data in noninferiority trials? A systematic review
Melanie Bell, Brooke Rabe
University of Arizona
**Correspondence:** Melanie Bell
*Trials* 2017, **18(Suppl 1):**P338

**Background**
Missing data pose a serious threat to the validity and interpretation of noninferiority trials and may result in the rejection of a promising new noninferior agent or the acceptance of what is, in fact, an inferior treatment. While there are recommendations for principled approaches to handling missing data in superiority trials, there are none for NI trials, and missing data can affect them differently.
**Methods**
We carried out a systematic review to investigate how researchers are handling missing data in noninferiority trials: the amount of missing data; the analyses used and the missing data assumptions; whether missing data were considered in the sample size calculation; and whether any sensitivity analyses were carried out.
**Results**
Most trials had missing data, most used a complete case analysis, about half accounted for missing data in the sample size calculation and very few carried out a sensitivity analysis. About one-fifth analyzed both the intention-to-treat and per-protocol sets.
**Conclusion**
There is room for improvement in handling missing data in noninferiority trials. There is also a need to carry out research in sensitivity analyses for noninferiorityi trials with respect to missing data.

### P339
### Bayesian predictive probability design in single-arm cancer phase II trials: is it superior to frequentist design?
Xinxue Liu, Victoria R. Cornelius
Imperial Clinical Trials Unit, Imperial College London
**Correspondence:** Xinxue Liu
*Trials* 2017, **18(Suppl 1):**P339

**Background**
Phase II trials play a vital role in cancer drug development as they determine whether a new drug should continue for further investigation. Most phase II cancer trials apply a single-arm design with a binary outcome, and multi-stage designs are commonly used to stop for futility in these settings. The most common design for single-arm phase II cancer trial is Simon's two-stage design. However, Bayesian design with continuous monitoring is getting popular in recently years as it is flexible and efficient given its intensive statistical input. In this study, we compared the operating characteristics of Simon's two stage design and Bayesian predictive probability (PP) design using a real life cancer phase II trial.
**Method**
The phase II cancer was original designed as a Simon's two-stage Minimax with the primary outcome of clinical benefit, defined as complete response, partial response or stable disease for 6 months. The trial tested H0: $p0 <= 0.05$ versus H1: $p1 >= 0.20$ with type I error of 0.05 and type II error of 0.20.The Bayesian PP design monitors the trial continuously so that the Bayesian posterior probability is updated after the outcome from each participant becomes available. The predictive probability of concluding a positive result by the end of the trial is calculated based on the updated posterior probability. In this study, we used $p0 = 0.05$ and $p1 = 0.20$ to design the trial. If the probability that the clinical benefit rate of p is larger than p0 exceeds a threshold of Theta-T at the end of the trial, the drug will be concluded as effective. During the monitoring, the trial will stop for futility if the PP is less than a threshold of Theta-L, and the trial will not stop for efficacy (Theta-U = 1). To compare with the Simon's minimax design, the minimum sample size is selected among the sample sizes satisfying the constraints of above type I and type II error. The corresponding Theta-L and Theta-T are 0.001 and [0.86, 0.95], respectively.
**Results**
The futility boundaries in Simon's minimax design are 0/13 and 3/27 with a sample size of 27 patients. In Bayesian PP design, the futility boundaries are 0/14, 1/24, 2/26 and 3/27 with the same sample size as Simon's minimax design. The exact type I errors in Simon's design and PP design are both 0.042, while the powers are 0.80 and 0.81, respectively. Although the probability of early stopping under null hypothesis is significantly higher in PP design than that in Simon's design (87% vs 51%), the expected sample size under null hypothesis for the two designs are the same ($E(N|H0) = 19.8$).
**Conclusion**
In this cancer phase II trial, where the clinical benefit rate of standard treatment (p0) was relatively low, the Simon's two stage design had similar operating characteristics compared to Bayesian PP design. In practice, this suggests that if a phase II trial has a stop boundary of 0 in the interim analysis of Simon's design, the Bayesian PP design is unlikely to be superior.

### P340
### A simple relationship between power and expected confidence interval width
Andrew Forbes[1], Richard Hooper[2]
[1]Monash University; [2]Queen Mary University of London
**Correspondence:** Andrew Forbes
*Trials* 2017, **18(Suppl 1):**P340

**Background**
There have been intermittent calls in the health sciences for sample size planning for randomised trials to be based on, or include, the expected width of the 95% confidence interval (CI) for the parameter of interest. The relationship between power of a test at a 5% significance level and the expected 95% CI width has appeared in the literature for trials planned with 80% or 90% power, most notably by Goodman and Berlin over 20 years ago. However, this relationship does not appear to be well known, has not been extended to treatment effect parameters other than differences between randomised arms, and it does not seem to have been realised that an even simpler approximate relationship also exists.
**Methods**
We derive the basic relationship between power and expected CI width, state its underlying assumptions, and illustrate its use in a series of examples for difference and ratio treatment effect measures used in randomised trials. We demonstrate that a linear approximation simplifies this relationship further.

**Results**

The expected 95% CI width calculated from the relationship with power compares very favourably with asymptotic analytical formulae. The simpler linear approximation is appropriate for any level of power between 50% and 95%.

**Conclusions**

One can determine the expected 95% CI width given a certain level of power, or vice versa, using an extremely simple relationship which makes it easy to conceptualise the consequences of one for the other. The relationship can be a useful rule of thumb to consider when planning trials.

### P341

#### Funnel plots for statistical quality control in a large, multi-site registry

Claire Boyle[1], Nicole C. Foster[1], Kenneth Scheer[2], Henry Anhalt[2], Avni Shah[3], Joyce Lee[4], Sarah Corathers[5]
[1]Jaeb Center for Health Research; [2]T1D Exchange; [3]Stanford University; [4]University of Michigan; [5]Cincinnati Children's Hospital Medical Center
**Correspondence:** Claire Boyle
*Trials* 2017, **18(Suppl 1):**P341

The T1D Exchange Clinic Network consists of 81 endocrinology practices throughout the United States. Eighteen of the centers primarily care for adult patients, 38 for pediatric patients, and 25 care for both. Among the more than 100,000 patients with type 1 diabetes (T1D) who receive care at these centers, more than 30,000 have been enrolled in the T1D Exchange clinic registry. The diverse size, resources, and practices among registry clinics may have an impact on the diabetes management and diabetes-related outcomes of participants. Understanding the variation in these resources and practices is an important step in determining how to improve diabetes management and diabetes outcomes. Statistical quality control is a method for monitoring quality of conformance and eliminating distinct causes of variability in a process through the use of graphical displays. One such graphical display is a funnel plot, which plots effect estimates from individuals against a measure of size or precision. Funnel plots also include lines for expected value of the effect and lower and upper control limits. These plots can be useful in assessing the variation in mean, median, or proportion of diabetes management factors and outcomes across clinic size. For example, a funnel plot of the proportion of diabetes patients achieving target glycemic control as measured by hba1c is an effective visual display of glycemic variation across clinical centers. The funnel plot enables identification of high performing centers that may provide insights to inform practice improvements for other participants in the network. Knowledge of variation in glycemic control, current use of advanced diabetes technologies, and occurrence of acute diabetes-related outcomes across varying clinic size is useful for learning and improving practices and resources in delivering diabetes care.

### P342

#### Late stage combination drug development for improved portfolio-level decision-making

Emily Graham[1], Thomas Jaki[1], Nelson Kinnersley[2], Chris Harbron[2]
[1]Lancaster University; [2]Roche Products Ltd
**Correspondence:** Emily Graham
*Trials* 2017, **18(Suppl 1):**P342

**Background**

We are interested in the problem of portfolio level decision making in the context of a pharmaceutical portfolio containing combination therapies. There has been a recent rise in popularity of combination therapies, particularly those containing new molecular entities. Our particular area of interest is oncology, due to the recent development of cancer immunotherapy treatments. While this development is an exciting one, it poses new challenges for pharmaceutical companies. One of these challenges is how to decide which combinations, from the large set of possible combinations, is the most promising and hence which therapies should be added to a company's portfolio. In order to make the best decisions for the portfolio, emerging information should be included alongside the available historical data. However, in the context of combination therapies we have a different source of information: the information from studies involving similar combinations. We believe that incorporating information from similar studies will lead to improved portfolio level decision making.

**Existing Methods**

We outline two conceptually different methods for optimising the expected outcome of a pharmaceutical portfolio from the literature and provide a discussion and comparison of these methods. The first method is based on real options analysis and draws upon the way in which the sequential nature of the investments made in a drug development programme corresponds to a series of call options. The resulting model formulation is a mixed integer linear programme which maximises the real options value of the portfolio. The second method is similar to the stochastic version of the resource constrained project scheduling problem. In this setting, the development programmes for each of the drugs within the portfolio will be treated as projects which are made up of stochastic and deterministic tasks. The resulting model formulation is a multi-stage stochastic programme and has a particular focus on the technical uncertainty involved in the process.

**Our Contribution**

The existing methods for portfolio decision making do not allow information about combination therapies specifically to be incorporated into the decision making process itself. Therefore, we provide a comparison and discussion of these methods in the context of a portfolio containing combination therapies before providing our own extension. Our extension builds on network meta-analytic techniques and allows information to be shared between studies for similar combination therapies. Learning across trials of similar combinations will allow us to improve the accuracy of our treatment effect estimates which in turn will lead to better informed decision making and hence better outcomes for the portfolio.

### P343

#### A Bayesian weighted quasi-likelihood design for phase I/II clinical trial with repeated dose administration in preterm newborns

Moreno Ursino[1], Ying Yuan[2], Corinne Alberti[3], Emmanuelle Comets[4], Tim Friede[5], Frederike Lents[6], Nigel Stallard[7], Sarah Zohar[1]
[1]INSERM, UMRS1138 - team22; [2]The University of Texas MD Anderson Cancer Center; [3]INSERM, UMR 1123; [4]INSERM, CIC 1414; INSERM IAME, UMR 1137; [5]University Medical Center Göttingen; [6]Federal Institute for Drugs and Medical Devices; [7]Warwick Medical School, The University of Warwick
**Correspondence:** Moreno Ursino
*Trials* 2017, **18(Suppl 1):**P343

**Background**

Preterm newborns are a very vulnerable population in which clinical trials are extremely difficult and therefore rarely conducted. A phase I/II trial aiming at finding the recommended dose of Levetiracetam for treating neonate's seizures was planned with a maximum sample size of 50. In the trial, 4 dose levels (consisting in a loading dose and up to 8 maintenance doses) are considered with 3 primary outcomes: efficacy, short term toxicity (Ts) and long term toxicity (Tl). Tl occurs at the same time as short term toxicity but can only be measured at a later time. In the absence of efficacy, physicians could add a second agent as rescue medication, which could differ from centre to centre.

**Materials and methods**

A Bayesian design was developed for this trial. The 3 primary outcomes were modelled via a logistic model for efficacy, a time-to-event quasi-likelihood for Ts and a quasi-likelihood with Ts as covariate for Tl, as Ts is predictive for Tl. The quasi-likelihood method allows us to take into account the fact that toxicity may be due to Levetiracetam or to the added second agent or to both, in case the Levetiracetam shows no efficacy and a second agent is added. Relevance weights were added to the model to avoid stickiness (that is, to be stuck for several patients at the same suboptimal dose level) due to early toxicities along with small target probability. Finally, this model allows sequential analyses on accumulating data. Dose escalation rules were based on adaptive thresholds for posterior probabilities, in the start-up phase considering only Ts while later considering both Ts and Tl. A simulation study was conducted to assess the design under several scenarios for sample size of 30, 40 and 50, respectively. The same design without quasi likelihood part, that is considering all toxicities due to Levetiracetam, and without relevance weight was used for comparison.

**Results**

On average, the proposed design leads to recommendation of the correct dose at about 60% for a sample size of 30, increasing up to more than 80% in many scenarios for a sample size of 50. This model maintains an acceptable number of neonates with toxicities when compared to the same design without quasi-likelihood part and without relevance weights.

**Acknowledgments**

This work was conducted as part of the inspire (Innovative methodology for small populations re- search) project funded by the European Union's Seventh Framework Programme for research, technological development and demonstration under grant agreement number FP HEALTH 2013–602144, but does not necessarily represent the view of all inspire partners.

### P344

#### The use of unequal allocation ratios in the design of randomised phase II trials

Richard Jackson, Paul Silcocks, Trevor Cox
University of Liverpool
**Correspondence:** Richard Jackson
*Trials* 2017, **18(Suppl 1):**P344

**Objectives**

Equal allocation of patients to one of two treatments is accepted almost universally in the design of randomised clinical trials and it is often assumed that this approach provides the most efficient use of available resources.

**Background**

Design of phase II studies with a binary endpoint is often carried out in a two-stages following the principles of Simon and A'Hern and extended into randomised trials by Jung and Sargent. Assessments of efficacy are often made via the odds ratio, the precision of which is only optimal under equal allocation when there is no difference in the response rates between the two treatment arms.

**Methods**

For trials where the response rates are p_x and p_y in the experimental and control arm respectively, we propose to allocate 0 1 patients to the experimental arm such that

= (1 + A)^(−1)

Where

A = (p_x (1-p_x))/(p_y (1-p_y))

**Results**

Sample size calculations based on the exact methodology of Jung and Sargent show that estimates are smaller than those where equal allocation is used, with the discrepancy being greater as the response rate tends towards 0 or 1. In studies where standard sample size calculations are used, for a fixed sample size using unequal allocation will ensure a smaller standard error about the odds ratio for studies where there is a positive response.

### P345

#### Rituximab versus cyclophosphamide for the treatment of connective tissue disease associated interstitial lung disease (*RECITAL*): a randomised controlled trial

Vicky Tsipouri[1], Peter Saunders[1], Greg J. Keir[2], Deborah Ashby[3], Sophie V. Fletcher[4], Michael Gibbons[5], Matyas Szigeti[3], Helen Parfrey[6], Elizabeth A. Renzoni[1], Chris P. Denton[7]
[1]Royal Brompton Hospital; [2]Princess Alexandra Hospital; [3]Imperial College London; [4]Southampton General Hospital; [5]Royal Devon and Exeter Hospital; [6]Papworth Hospital; [7]Royal Free Hospital
**Correspondence:** Vicky Tsipouri
*Trials* 2017, **18(Suppl 1):**P345

**Background**

Interstitial lung disease (ILD) frequently complicates systemic autoimmune disorders resulting in considerable morbidity and mortality. The connective tissue diseases (CTDs) most frequently resulting in ILD include; systemic sclerosis, idiopathic inflammatory myositis (including dermatomyositis, polymyositis and anti-synthetase syndrome) and mixed connective tissue disease. Despite the development, over the last two decades, of a range of biologic therapies which have resulted in significant improvements in the treatment of the systemic manifestations of CTD, the management of CTD-associated ILD has changed little. At present there are no approved therapies for CTD-ILD. Following trials in scleroderma-ILD, cyclophosphamide is the accepted standard of care for individuals with severe or progressive CTD-related ILD. Observational studies have suggested that the anti-CD20 monoclonal antibody, rituximab, is an effective rescue therapy in treatment refractory CTD-ILD. However, before now, there have been no randomised controlled trials assessing the efficacy of rituximab in this treatment population.

**Methods**

RECITAL is a multicentre, randomized, double-blind, double-dummy, controlled trial funded by the Efficacy and Mechanism Evaluation Programme of the Medical Research Council and National Institute for Health Research. The trial, which has to date recruited ~30% of its target recruitment, will compare rituximab 1 g given intravenously, twice at an interval of two weeks, with intravenous cyclophosphamide given monthly at a dose of 600 mg/m2 body surface area in individuals with ILD due to systemic sclerosis, idiopathic inflammatory myositis (including anti-synthetase syndrome) or mixed connective tissue disease. A total of 116 individuals will be randomised 1:1 to each of the two treatment arms, with stratification based on underlying CTD, and will be followed for a total of 48 weeks from first dose. The primary endpoint for the study is change in forced vital capacity (FVC) at 24 weeks. Key secondary endpoints include; safety, change in FVC at 48 weeks as well as survival, change in oxygen requirements, total 48 week corticosteroid exposure and utilisation of healthcare resources.

**Discussion**

This is the first randomised control trial to study the efficacy of rituximab as first line treatment in CTD-associated ILD. To date we have recruited 34 patients from 3 UK sites. Our recruitment accruals represent one of the largest cohorts worldwide in these rare diseases. Herewith, we are presenting baseline characteristics of this unique cohort. The results anticipated at the conclusion of the trial should provide important information on the treatment of a life-threatening complication affecting a rare group of CTDs.

### P346

#### Calculating expected survival from high-dimensional cox models with treatment-by-biomarker interactions in randomized clinical trials

Nils Ternès, Federico Rotolo, Stefan Michiels
Gustave Roussy
**Correspondence:** Nils Ternès
*Trials* 2017, **18(Suppl 1):**P346

**Background**
Thanks to the advances in genomics and targeted treatments, an increasing interest is being devoted to develop prediction models with biomarkers or gene signatures to predict how likely patients will benefit from particular treatments. Despite the methodological framework for the development and validation of gene signatures in a high-dimensional setting is quite well established, no clear guidance exists yet on how to estimate expected survival probabilities. We propose a unified framework for developing and validating a high-dimensional Cox model integrating clinical and genomic variables in a randomized clinical trial to estimate the expected absolute treatment effect according to signature values, and to estimate expected survival probabilities for patients with associated confidence intervals.
**Methods**
Based on a parsimonious selection model in a penalized (lasso or adaptive lasso) high-dimensional Cox model, we investigated several strategies to: estimate the individual survival probabilities at a given timepoint (using single or double cross-validation); construct confidence intervals thereof (analytical or bootstrap); and visualize them graphically (pointwise or spline). We compared these strategies through a simulation study covering null and alternative scenarios and we evaluated them by prediction criteria. We applied the strategies to a large randomized controlled phase III trial in 1574 early breast patients that evaluated the effect of adding trastuzumab to chemotherapy and for which the expression of 462 genes were measured.
**Results**
Simulation results suggest that a penalized regression model estimated using adaptive lasso estimates the survival probability of new patients with low bias and standard error, and that bootstrapped confidence intervals have empirical coverage probability close to the nominal level across very different scenarios. The double cross-validation allows mimicking internally the prediction performance in absence of external validation data. We also propose a visual representation of the expected survival probabilities using splines. In the breast cancer trial, we identified a prediction model with 4 clinical covariates, the main effect of 98 biomarkers and 24 biomarker-by-treatment interactions. This illustration also highlights the high variability of the expected survival probabilities, with very large confidence intervals.
**Conclusion**
We propose a unified framework for developing and validating a gene signature in a high-dimensional survival setting in order to calculate expected survival probabilities at a given horizon for future patients, and to visualize the survival predictions. Based on our simulations, the adaptive lasso penalty can be useful to identify a signature and then, to accurately estimate the expected survival probability of future patients.

### P347
### Considerations in designing equity-relevant clinical trials
Lawrence Mbuagbaw[1], Beverley Shea[2], Theresa Aves[1], Vivian Welch[2], Monica Taljaard[3], George Wells[3], Peter Tugwell[2]
[1]McMaster University; [2]Bruyère Research Institute; [3]Ottawa Hospital Research Institute
**Correspondence:** Lawrence Mbuagbaw
*Trials* 2017, **18(Suppl 1):**P347

**Background**
Disparities in health and health outcomes are a common feature in health research. When these disparities are unfair and avoidable they may be referred to as inequities. Due consideration of inequities is important to inform the design and conduct of trials so that they do not aggravate inequities, but instead capture the role of inequities in a credible and informative way. In light of the lack of evidence on equity and the absence of guidance on how to design a purposefully equity-relevant trial, the Consolidated Standards for Reporting (CONSORT) equity advisory group came together to address these issues.
**Content**
This work is part of a broader project that includes the development of a framework for defining equity-relevant trials and a CONSORT extension for equity-relevant trials. This work discusses approaches to integrating equity considerations in equity-relevant randomized trials by building upon the PROGRESS-Plus framework (Place of residence, Race, Occupation, Gender, Religion, Education, Socioeconomic status, Social capital and other context-specific factors) and covers research questions formulation, two scenarios of equity relevant trials and how the PROGRESS-Plus factors may influence trial design, conduct, and analyses.
**Conclusion**
With an a-priori focus on certain equity items, trials can be designed to optimize their ability to provide actionable and credible evidence on equity, by careful consideration of design, conduct and analytical issues that play a role in equity.

### P349
### Blinding in randomized controlled trials in general and abdominal surgery: a systematic review and empirical study
Pascal Probst, Steffen Zaschke, Patrick Heger, Phillip Knebel, Alexis Ulrich, Markus W. Büchler, Markus K. Diener
University of Heidelberg
**Correspondence: Pascal** Probst
*Trials* 2017, **18(Suppl 1):**P349

**Background**
Blinding is a measure in randomized controlled trials (RCT) to reduce performance and detection bias. There is evidence that lack of blinding leads to overestimated treatment effects. Since, surgical trials use interventions with a physical component blinding is often complicated to apply. The aim of this study was to analyse the actual impact of blinding on outcomes in general and abdominal surgery RCT.
**Methods**
A systematic literature search in CENTRAL, MEDLINE and Web of science was conducted to locate RCT between 1996 and 2015 with a surgical intervention. General study characteristics and information on blinding methods were extracted. The risk of performance and detection bias was rated as low, unclear or high according to the Cochrane Collaboration's tool for assessing risk of bias. The main outcome was the association of a high risk of performance or detection bias with significant trial results and was tested at a level of significance of 5%.
**Results**
Out of 29´119 articles 378 RCT were included in the analysis investigating a total of 62´522 patients of which 15´025 patients were blinded (24.0%). Regarding performance bias 88 of 378 RCT (23.3%) were at high risk of performance bias and 290 of 378 RCT (76.7%) were not. Hereby, 50 of 88 high risk RCT (56.8%) showed significant trial results compared to 134 of 290 non-high risk RCT (46.2%) resulting in non-significant association (OR 1.53; 95%-CI: 0.95 to 2.48; $p = 0.08$) of performance bias and trial results. Further, 59 of 378 RCT (15.6%) were at high risk of detection bias and 319 of 378 RCT (84.4%) were not. Hereby, 28 of 59 high risk RCT (47.5%) showed significant trial results compared to 156 of 319 non-high risk RCT (48.9%) resulting in non-significant association (OR 0.94; 95%-CI: 0.52 to 1.65; p = 0.84) of detection bias and trial results.
**Discussion**
Surprisingly, performance and detection bias do not distort treatment effects in general and abdominal surgery RCT. Therefore, surgical researcher can rely on this evidence and leave out complicated ways of blinding methods. However, easily applicable blinding measures should be taken for the theoretical advantage. During critical appraisal of a surgical RCT the threat to validity of trial results by performance and detection bias should not be overestimated.

### P350

#### Post-trial follow-up methodology in large randomized controlled trials: a systematic review

Rebecca Llewellyn-Bennett[1], Danielle Edwards[2], Richard Bulbulia[2], Louise Bowman[2]
[1]University of Oxford; [2]Clinical Trial Service Unit, Nuffield Department of Population Health, University of Oxford
**Correspondence:** Rebecca Llewellyn-Bennett
*Trials* 2017, **18(Suppl 1):**P350

**Background**
Large randomised trials tend to have a relatively short "in-trial" Follow-up period and hence may underestimate any long-term benefits of the assessed intervention or fail to detect delayed hazards. Post-trial follow-up (PTFU), which we define as extended follow-up either after the scheduled trial period or publication of the primary results, allows detection of both persistent or enhanced beneficial effects following cessation of study treatment (ie, a legacy effect) and the emergence of possible adverse effects (eg, cancer). Despite these advantages, PTFU is not routinely undertaken and, when implemented, methods vary widely. This review describes methods of PTFU used in recent large randomised trials, and will compare retention rates and study costs where such information is available, and may help promote the use of effective PTFU for ongoing and future large trials.
**Methods**
A systematic search of electronic databases and clinical trial registries was conducted using a pre-defined search strategy with the following inclusion criteria: i) randomized trials with 1000 or more participants, ii) published between March 2006–2016; iii) evaluation of medical, surgical or psychological interventions; iv) implementation of post-trial follow-up reported. Two reviewers screened and extracted data from eligible papers with the aim of 95% concordance and any discrepancies were resolved by a third reviewer. Retention rates, costs and other descriptive differences of PTFU were reviewed. The systematic review was conducted following PRISMA guidelines.
**Results**
The search strategy incorporated relevant papers from Cochrane Central Register, Embase, Medline and clinical trial registries yielding 50,153 papers from databases (49,915) and trial registries (218). After excluding duplicates (22,168), studies of children and animals (1649) and papers published before 2006 (9289). 17,027 abstracts were screened by 2 reviewers using a concordance strategy. Reviewers were 73% concordant for the first 10% of abstracts screened, but after discussion concordance rose to 99%. Following abstract screening, 239 papers and 218 protocols were eligible for full review and preliminary results suggest that around half will represent unique studies with relevant data to extract in the review. The length of PTFU ranged from 1–20 years and PTFU methods varied, including direct patient contact via clinic appointments, postal questionnaires, telephone interviews and indirect follow-up via national registries. Some trials used incentives for participant retention, including free healthcare relevant to the intervention. Several PTFUs were prompted by the Data Monitoring Committee because of concerns about potential delayed treatment hazards. Occasionally trials investigated an outcome different to the in-trial primary endpoint. Where industry supported the in-trial period, such funding for PTFU was infrequent. Final results of the review are pending and will be presented.
**Conclusions**
Post-trial follow-up of large RCTs may allow more reliable estimation of the long-term benefits of the study treatment and the detection of any delayed adverse effects which might not emerge during the relative "in-trial" period. This review will describe the methods of post-trial follow-up used in a range of recent randomized trials. We anticipate that PTFU using routinely collected health records will be more comprehensive and cost-effective than studies involving direct patient contact.

### P351

#### Timely and reliable evaluation of the effects of interventions: a framework for adaptive meta-analysis (FAME)

Jayne Tierney[1], Claire L. Vale[2], Sarah Burdett[2], David Fisher[2], Larysa Rydzewska[2], Mahesh K. B. Parmar[2]
[1]University College London; [2]MRC Clinical Trials Unit at UCL, Institute of Clinical Trials and Methodology, UCL; MRC London Hub for Trials Methodology Research
**Correspondence:** Jayne Tierney
*Trials* 2017, **18(Suppl 1):**P351

**Background**
Most systematic reviews are retrospective and use aggregate data (AD) from publications, meaning they can be unreliable, lag behind therapeutic developments and fail to influence ongoing or new trials. Commonly, the potential influence of unpublished or ongoing trials is overlooked when interpreting results, or determining the value of updating the meta-analysis or need to collect individual participant data (IPD). Therefore, we developed a Framework for Adaptive Meta-analysis (FAME) to determine prospectively the earliest opportunity for reliable AD meta-analysis. We illustrate FAME using two systematic reviews in men with metastatic (M1) and non-metastatic (M0) hormone-sensitive prostate cancer (HSPC).
**Methods**
Key principles of FAME are: 1) Start the systematic review process early, before all trials have completed 2) Comprehensively search for published, unpublished and ongoing eligible trials; 3) Develop a detailed picture of these trials, particularly how information and results are likely to accumulate; 3) Predict the feasibility and timing of a reliable meta-analysis; 4) Interpret meta-analysis results accounting for trials that have not yet completed/reported; 5) Determine if an update based on AD or IPD is needed.
In 2014, using FAME, we initiated two systematic reviews to evaluate the effects of adding docetaxel to standard care in men with HSPC. We predicted that, by mid-2015, results of 3 of 5 eligible trials in M1 disease would become available, each with median follow-up of around 4 years. They would represent around 90% of all men randomised, giving 70 to >99% power to detect a 5% -10% absolute difference in 4-year survival. This provided a clear trigger for a robust meta-analysis.
Also, for M0 disease, we anticipated the availability of results from 4 of 11 eligible trials, again with median follow-up around 4 years. Power would be reasonable (60 to >99%) to detect similar absolute effects, but only 60% of randomised men would be represented. Although a meta-analysis would not be definitive, it could provide useful context for the M1 results and for ascertaining when a robust update of the meta-analysis might be feasible.
**Results**
In M1 disease, we found a clear benefit of docetaxel on survival. FAME gave us confidence that the primary question was answered definitively, without needing to wait for results of the remaining 2 trials, or collecting IPD. Collaborating with trialists through FAME gave us access to pre-publication trial results, and facilitated contemporaneous publication of the systematic review and the largest trial.
In M0 disease, there was a clear effect of docetaxel on failure-free survival, but overall survival results were inconclusive. Therefore, FAME provided an early signal of potential benefit, and highlighted the value of a future update that includes longer-term follow-up of included trials and results of currently unreported trials. Ongoing collaboration with trialists will provide up-to-date information, enabling better prediction of the timing and feasibility of a definitive meta-analysis, and whether AD or IPD will be required. It will also facilitate a co-ordinated dissemination strategy.

**Conclusions**

In piloting FAME, we have shown that meta-analysis can be done in a timely and transparent manner without compromising reliability

### P352

#### On synthesis evidence from explanatory and pragmatic trials: a comparison of meta-analytic methods

Tolulope Sajobi[1], Oluwagbohunmi Awosoga[2], Meng Wang[1], Anita Brobbey[1], Guowei Li[3], Bijoy K. Menon[1], Michael D. Hill[1], Lehana Thabane[3]
[1]University of Calgary; [2]University of Lethbridge; [3]McMaster University
**Correspondence:** Tolulope Sajobi
*Trials* 2017, **18(Suppl 1):**P352

Randomized controlled trials of treatments and interventions are typically described as either explanatory or pragmatic. Meta-analysis of RCT studies typically pools evidence of treatment effects from included studies, regardless of their classification as 'pragmatic' or 'explanatory trials. Given that treatment effects in explanatory trials may be greater than those obtained in pragmatic trials, conventional meta-analytic approaches may not accurately account for the heterogeneity among the studies and may result in biased estimates of treatment effects. Stratified meta-analysis of systematically review studies in which treatment effects from explanatory trials are meta-analyzed and reported separately from pragmatic trials is increasingly being adopted in meta-analysis studies. But this approach might not necessarily inform decision-making especially when stratum-specific pooled treatment effects are in opposite directions. In this study we investigate a variety of meta-analytic approaches for synthesizing evidence from pragmatic and explanatory trials, including mixture random-effects meta regression, robust random-effects meta-regression, and hierarchical Bayesian meta-analysis techniques for synthesizing evidence from pragmatic and explanatory trials. Data from a systematic review of 55 published obesity prevention trials, which investigated the effectiveness of public health intervention on reduction of obesity, was used to demonstrate and compare these methods. Discussions about the key statistical and design considerations when pooling evidence from both types of trial designs are provided.

### P353

#### Clinical trial units of medical scientific societies to close evidence gaps

Gabriele Dreier
University Medical Center Freiburg
*Trials* 2017, **18(Suppl 1):**P353

**Background**

In the last 25 years, ebm has increasingly found its way into clinical practice and research. Existing evidence primarily serves doctors to support their decision-making, but is also the basis for providing scientific proof for a health care intervention`s benefit to patients and ultimately payers/health insurances. The closure of existing evidence gaps requires substantial human and financial resources, and can only succeed with the involvement of clinical and methodological expertise.

**Objectives**

Scientific Societies have a natural interest in detecting and closing evidence gaps. Here we report a project of the German Society of Otolaryngology, Head and Neck Surgery (DGHNOKHC) and the German professional association of otolaryngologists (BVHNO) which can serve as a master example for similar projects.

**Methods**

The two institutions have a vested interest in supporting their members in the generation and dissemination of evidence, and to foster the transfer of knowledge into practice. This includes the areas of diagnosis, treatment, prognosis and prevention, comprising the application of medicinal products, medical devices or surgical procedures. The executive committees of DGHNOKHC and BVHNO have together founded the German Clinical Trials Unit for Ear, Nose and Throat medicine, Head and Neck Surgery (DSZ-HNO) to assist their members in the identification of evidence gaps and the planning and conduct of systematic reviews and clinical trials. An interdisciplinary team of statisticians, physicians, project managers, study nurses, data managers and monitors provides the required expertise. The first projects have been started, including a BMBF (German Ministry for Education and Research)-funded clinical trial for the treatment of sudden hearing loss. A survey among all members of both associations to detect evidence gaps was conducted. The results led to a prioritization process and planning of trials, registries, systematic reviews and other projects with industry and academia alike. A presentation at the Guideline Commission of the Working Group of German Medical Scientific Societies led to further Societies wanting to copy the ENT example, thus a Clinical Trial Unit as presented here can be a suitable model for closing evidence gaps and fostering clinical trials.

### P354

#### Review of treatment allocation schemes reported in published clinical trial results

Jody Ciolino, Hannah L. Palac, Amy Yang, Mireya Vaca
Northwestern University
**Correspondence:** Jody Ciolino
*Trials* 2017, **18(Suppl 1):**P354

**Background**

Properly designed and implemented randomized controlled trials (RCTs) serve as the ideal form of evidence-based research to establish efficacy of new therapies; however, substantial debate regarding most appropriate trial designs persists today. Areas of confusion include: appropriate treatment allocation techniques to ensure comparable baseline arms, best reporting practices, and controlling for influential variables at the analysis phase. While randomization literature promotes covariate adaptive methods (e.g., minimization, developed 1974) to protect against baseline imbalance and provide more efficient analyses, many investigators prefer simpler methods (e.g., stratified blocking schemes) for their understandability and ease of implementation. This manuscript reviews recently published rcts to illustrate current practice.

**Methods**

We searched pubmed for articles indexed 'randomized controlled trial', published in the New England Journal of Medicine, Journal of the American Medical Association, British Medical Journal, or Lancet for two time periods: 2009 and 2014 (before and after establishment of updated Consolidated Standards of Reporting Trials [CONSORT] guidelines). Upon completion of screening, articles underwent full review to collect data related to trial characteristics, the type of randomization scheme used, and clarity of reporting.

**Results**

Our search returned 343 articles, 298 of which we included in full review. The majority reported on superiority (86%), multicenter (92%), two-armed (79%) trials. With respect to CONSORT adherence, 68% of trials indicated a 'randomizedr Trial in the title, and the randomization scheme could not be determined in 10% of studies. Consistent with our hypothesis, the majority of articles reported a stratified block method (69%) of allocation, but 81% of trials involved covariates in the treatment allocation procedure. The majority (84%) of trials reported adjusted analyses, with 91% of these adjustments in analyses pre-specified. Trials published in the later time period (2014 vs. 2009) were more likely to have clearer report of randomization scheme (84% vs. 66%, $p = 0.0003$), report adjusted analyses (87% vs. 79%, $p = 0.0100$), and pre-specify adjustment in analyses (95% vs. 85%, p = 0.0045). Study start year significantly predicted whether design involved a covariate adaptive method of allocation, but in the opposite hypothesized direction: odds of adaptive method use decreased for every one-year increase in study start (OR = 0.89 [0.82, 0.96], $p = 0.0045$). However, odds of pre-specified adjusted analyses tended to increase over time (OR = 1.13 [1.02, 1.24], $p = 0.0145$).

#### Discussion

Our findings suggest that while optimal reporting procedures and pre-specification of adjustment in analyses for RCTs tend to be progressively more prevalent over time, we have evidence of the opposite effect on use of sophisticated covariate adaptive methods in clinical trial practice. Many authors suggest covariate adaptive methods as ideal in designing clinical trials, but there is a disconnect between theory and practice. Moreover, our results suggest a widening of this gap as time moves on.

### P355

#### Rolling dose escalation with overdose control: an efficient and safe phase 1 design

Daniel Sabanes Bove, Jiawen Zhu, Ulrich Beyer
Hoffmann-La Roche Ltd
**Correspondence:** Daniel Sabanes Bove
*Trials* 2017, **18(Suppl 1):**P355

This abstract is not included here as it has already been published.

### P356

#### Training the clinical investigators of the future - A clinical trials clerkship

Natalie Ives, Jon Deeks
University of Birmingham
**Correspondence:** Natalie Ives
*Trials* 2017, **18(Suppl 1):**P356

#### Background

To support future clinical trial research it is important that those planning a career in clinical trials are supported and trained to lead on these trials. Training clinical investigators of the future in the design, management and analysis of clinical trials is key [Sackett]. Clinical Trials Units (CTUs) have extensive experience in the design and delivery of clinical trials, and provide an excellent training environment in which to embed researchers of the future. Ctus provide a unique opportunity for researchers to learn about clinical trials in a highly active research environment, alongside staff who work on trials every day.

#### Methods

To develop a clinical trials clerkship for clinical investigators that combines a programme of training with hands-on experience, mentorship and access to experts working in clinical trials.

#### Results

At the Birmingham Clinical Trials Unit (BCTU) at the University of Birmingham, we have developed a clinical trials clerkship where fellows spend on average 15 days (including a 3 day Research Methods Course) in BCTU over a 12 month period. The fellows will have the opportunity to learn about various trial processes from study set-up, protocol and case report form development, regulatory requirements, trial management, database development, statistical aspects of clinical studies, recruitment strategies, data management and monitoring, trial steering committees, interim and final data analysis and submitting for publication. Each fellow is assigned a senior trialist who acts as their mentor, who is responsible for working with the fellow to tailor the training and learning experience. The fellow is expected to maintain a reflective log of their taught and experiential training through completion of a workbook. For those planning to run a trial as part of their fellowship, these can be embedded within BCTU, with mentorship on delivering the project provided by a senior trial manager, trial co-ordinator, database programmer and statistician. Appropriate CTU costs to help the fellow deliver the study can be included in NIHR fellowship applications.

#### Conclusions

Within BCTU, we are currently running our first cohort of fellows following the above programme, and initial feedback is that it is an enjoyable and highly valuable learning experience.

### P357

#### The pros and cons of an 'umbrella' trial design for a rare disease from a trial management and data management perspective

Sue Bell, Jo Copeland, Alexandra Smith
University of Leeds
**Correspondence:** Sue Bell
*Trials* 2017, **18(Suppl 1):**P357

#### Background

Anal cancer is a rare disease, but its incidence is rising rapidly. Approximately 1000 cases in the UK and 5,000 in the USA are diagnosed each year. Standard treatment for anal cancer includes concurrent Mitomycin C, 5-Fluorouracil (or more recently capecitabine) and radiotherapy. Due to advances in radiotherapy technology, a new generation of clinical trials is now required that optimises radiotherapy dose based on a stratified risk assessment of the disease.

#### Methods

To capture as many anal cancer patients as possible, we developed an umbrella protocol that would capture patients across the spectrum of disease. PLATO (personalising Anal cancer radiotherapy dose) (ISRCTN88455282) is an integrated protocol, comprising 3 separate trials (ACT3, ACT4 and ACT5) in which the most relevant clinical research questions are asked across three distinct risk strata. Each trial asks separate questions and has separate eligibility criteria and sample sizes.

The ACT3 trial (n = 90) is a non randomised phase II study for low-risk disease that will evaluate a strategy of local excision only versus local excision plus radiotherapy, depending on the size of tumour margin post local excision (>1 mm versus < =1 mm, respectively). The ACT4 trial (n = 162) is a randomised phase II trial (2:1) for intermediate-risk disease comparing standard dose chemoradiotherapy with reduced-dose chemoradiotherapy. The ACT5 trial (n = 640) is a seamless pilot (n = 60)/phase II (n = 140)/phase III trial (n = 672 total) for patients with high risk disease that will compare standard dose chemoradiotherapy with two increased doses. Only one of the dose escalated experimental arms will be evaluated for the phase III component. The primary end point for each trial is 3 year locoregional failure. PLATO is funded by Cancer Research UK and is due to open to recruitment in the UK and Ireland in Q4 2016.

#### Discussion

Time, money and resources could potentially be saved by incorporating more than one trial under the umbrella of one protocol. The PLATO trial concept allows different research questions across the locoregional disease spectrum to be addressed efficiently using a single protocol and clinical trial funding application. This type of trial design is increasingly important in the era of personalised medicine and the need for clinical studies to address different research questions within the same disease. Sharing the details of this concept should assist other investigators to develop similar future studies. Details of our experience of implementing an integrated protocol along with the pros and cons of this approach from a trial and data management perspective will be presented in more detail.

### P358

#### Partnership of cancer center core facilities with community-based research networks in the coordination and management of multi-center clinical trials

Rani Jayswal, Stacey Slone, Mark Stevens, Rushi Goswami, Lara Sutherland, Kris Damron, Emily Dressler, Brent Shelton, Eric Durbin, Heidi L. Weiss
Markey Cancer Center, University of Kentucky
**Correspondence:** Rani Jayswal
*Trials* 2017, **18(Suppl 1):**P358

The importance of translation of clinical trials into catchment populations of Cancer Centers coupled with the advent of molecularly targeted agents and emphasis in precision medicine resulting in smaller patient pool within a single institution entail the need to engage multiple sites for the design and implementation of clinical trials. The conduct of multi-center studies is necessarily complex, requiring informatics tools and data management processes that need a coordinated effort necessitating an infrastructure akin to Data Coordinating Centers. We present a model whereby biostatistics and informatics core facilities partner with community based research networks to manage multi-center clinical trials. More specifically, we focus on three critical areas in informatics and data management namely i) development of an integrated set of standard operating procedures (SOPs) between the community based network and MRU pertaining to all aspects of data management; ii) improving utilization of a clinical trial management system (CTMS), a biospecimen management system and customized database applications to accommodate multi-center studies and iii) adopting and expanding automated statistical programs to monitor protocol-specific triggers including subject accrual, safety, and efficacy endpoints into a multiple site setting. The community research network focuses on administrative coordination and site communication and management to serve as a clinical coordinating center. We demonstrate this model for the conduct of a therapeutic intervention trial and non-intervention study; provide the specific informatics, data management and statistical tools we have implemented to manage multi-center studies; and discuss challenges and areas of improvement in this partnership infrastructure for provision of an integrated clinical and data science coordination for multi-center clinical studies.

### P359

#### Concepts important to the design of an innovative risk register in general practice databases? Developing methodology from ARRISA-UK

Stanley Musgrave[1], Erika Sims[1], David Price[2], Annie Burden[2], Allan Clark[1], Susan Stirling[1], Mohammad Al Sallakh[3], Gwyneth Davies[3], Estelle Payerne[1], Ann Swart[1]
[1]Norwich Medical School; [2]Research in Real Life, Ltd.; [3]Swansea University Medical School
**Correspondence:** Stanley Musgrave
*Trials* 2017, **18(Suppl 1):**P359

##### Background

Many clinical conditions require the identification and stratification of risk to ensure that interventions can be targeted appropriately. Challenges to identification of 'at-risk' patients using data from electronic health records include identification of relevant characteristics, how data availability informs decision making, coding and storage of data, and how data can be searched for, accessed and managed. Each week in the UK, 22 patients die and 1400 are hospitalised due to asthma (Asthma UK). Sixty per cent of patients with at-risk asthma defined according to British Thoracic Society (BTS) guidelines have an exacerbation requiring prednisolone per year compared to 10% of the total asthma population and BTS guidelines suggest at-risk registers may be useful for asthma. The At-Risk Registers Integrated into primary care to Stop Asthma crises - UK (ARRISA-UK) study group are evaluating the effectiveness and cost effectiveness of generating and implementing an at-risk asthma register.

**Developing a risk profile for an at-risk register: importance of a multi-disciplinary team**

Candidate characteristic values to be included in the risk profile were identified based on expert opinion, prior work and literature review. This list was reviewed by a working group within the ARRISA-UK team to identify additional characteristics based on clinical experience of managing asthma, consider limitations/restrictions of GP Practices. Clinical data systems in relation to the characteristics identified, and evaluate reliability and variability of the characteristics in terms of real world coding of clinical information. The characteristics contributing to the identification of patients with a statistically significant risk of hospitalisation were determined in the Optimum Patient Care Research Database through an iterative process of regression analysis and reassessment. The coefficients of the characteristics (including age, smoking status, comorbidities (rhinitis, diabetes, ischaemic heart disease, anxiety and/or depression, and anaphylaxis), BTS treatment step, paracetamol treatment, lower respiratory tract infection, oral corticosteroid therapy or hospitalisation in the previous year, body mass index and blood eosinophil count) then are used in an algorithm to calculate a risk score which was validated in a second database, the Secure Anonymised Information Linkage databank.

Implementing an At-Risk Register Using this algorithm, the ARRISA-UK search tool identifies at-risk individuals in general practices. Search reports from the GP clinical database system for the characteristics above are analysed, and the risk assessment is flagged in relevant patient records via specific Read or SNOMED codes. These inform the computerised decision support system in the form of popup information boxes prompting clinical action. They can also facilitate care management tasks, data collection and further clinical coding.

**Beyond ARRISA-UK**

These experiences will be used to develop strategies using a multidisciplinary approach for identification and recruitment of at-risk individuals in other disease areas. This will permit development of methodology for efficient trial design, delivery and planning in primary care.

##### Funding

The ARRISA-UK study is funded by the National Institute for Health Research's Health Technology Assessment Programme (13.34.70). The views and opinions expressed are those of the authors and do not necessarily reflect those of the HTA, NIHR, NHS or the Department of Health.

### P360

#### Consolidated trial management: an example of a purpose built clinical trial management system for an academic research organization

Rebecca Mister[1], Seshu Atluri[2], Burcu Vachan[3], Wendy Hague[4]
[1]Head of Site Management; [2]Analyst Programmer; [3]Oncology Program Manager; [4]Clinical Trials Program Director
**Correspondence:** Rebecca Mister
*Trials* 2017, **18(Suppl 1):**P360

##### Background

The NHMRC Clinical Trials Centre (CTC) based in Sydney, Australia and affiliated with the University of Sydney is an Academic Research Organisation (ARO) which develops and co-ordinates multi-centre clinical trials in Australia, New Zealand and internationally. Working across number of different fields including cardiology, oncology, neonatology and endocrinology the group collaborates with a number of institutions including study sites, other international co-ordinating centres, cros, cmos and central laboratories. As central coordinating centre for a number of clinical trials, the CTC frequently works with the same study sites (and personnel) across a number of different trials. A need was identified to collate trial operations information centrally to reduce time for individual trials collecting this information in their own bespoke systems. There was also a need to be able to collate core information (timelines, approvals) across trials and report these centrally in order to generate metrics to review performance. After consideration and review of the cost and functionality of existing commercial software packages it was decided to develop a custom system in-house, tailored to CTC specific trial co-ordination requirements.

**Aim**

To develop and implement a user-friendly Clinical Trial Management System (CTMS) to support the clinical trials team at the CTC that was cost effective to develop and maintain. Project specification: The first step was to develop a requirements document by seeking input from relevant parties. The following key content domains were identified: projects, organisations, people and documents and their relationships specified. Key user requirements were ease of data entry and reporting.

**Project development**
System specifications were then prepared collaboration with a data systems developer. A relational database design and (Oracle) written in Java was used for development. After an initial prototype was developed the system was released for user testing by trials staff from the discrete functions within the trials teams. Once the system was qualified, but prior to rollout, existing study tracking data was imported into the new CTMS system. Reports (both within and across projects) were developed prior to release to enable staff to access key information.
**Project deployment**
Training sessions were conducted on the use of the new system. Staff were also invited to specify what reports would be helpful to their teams. Project Evaluation: After implementation, a process of continual user feedback and enhancement was undertaken to improve system usability and acceptability. Conclusion: Development of the initial system took approximately 12 months from the decision to develop, through specification and user testing, import of existing data to release. Since this time additional functionality and reporting has been developed and released periodically. The system has now been in use for 3 years and feedback from users demonstrates increasing acceptance of the system. However, there were key learnings from the experience of implementing a new software system e.g., unforeseen costs related to the lack of staff dedicated solely to this project (and impact on timelines), resistance to change, and the expansion of the original scope of the project with requests for further functionality.

### P361
### NIHR clinical trials fellowship: reflections from a fellow and a mentor
Phillip Whitehead, Kirsty Sprange, Alan Montgomery
University of Nottingham
**Correspondence:** Phillip Whitehead
*Trials* 2017, **18(Suppl 1):**P361

**Background**
The National Institute for Health Research (NIHR) introduced Clinical Trials Fellowships in 2012 with the aim of further developing existing NIHR trainees' skills and experience in clinical trials. Fellowships are hosted within Clinical Trial Units (CTU) that are in receipt of NIHR CTU Support Funding as these offer the best environment in which to: expose trainees to all aspects/stages of clinical trials; cover multiple studies; understand how proposals are developed from initial concept through to funding application by interdisciplinary, collaborative working; and tailor training to individuals' needs. We reflect on our experience of the fellowship from the perspective of the trainee (PW) and the CTU mentor (KS).
**Case study**
Who's who - PW is a Clinical Trials Fellow and occupational therapist with previous experience of feasibility RCTs in his NIHR Doctoral Research Fellowship. A core aim of the fellowship was to develop skills to become a future CI of a multicentre study. The Nottingham Clinical Trials Unit (NCTU) is a UK Clinical Research Collaboration registered unit, based at the University of Nottingham. The unit currently hosts a number of Fellowships and research training awards.
Application process - Collaborative meetings with NCTU helped balance the learning objectives of the trainee with the learning opportunities available at NCTU and identify suitable trials.
Training programme - NCTU developed an extensive 'menu' Of activities from which a tailored programme was produced covering: trial oversight, quality management and sops, pharmacy, trial set-up, site set-up, recruitment, data management, follow-up, write up and dissemination. KS, as CTU mentor, led in the development and oversight of PW's training programme.
Integration into the unit - PW worked within three trials teams to maximise experience and learning. During the course of the fellowship, regular meetings were held between the fellow and mentor to reflect on personal development and for NCTU to offer feedback and guidance.
Supplementary training - The "hands on" Experience in NCTU was supplemented with formal training opportunities involving methodology and statistical courses, and presenting at national conferences.
**Reflections**
Benefits and Challenges Benefits included experiential learning by involvement and integration into the unit and the various trials teams, involvement in multidisciplinary working, observing multiple chief investigators across multiple studies at various stages of the trials, knowledge of processes and procedures and making contacts' internal and external.
Challenges included capacity and availability of key activities across trials and the unit, time-lag between the application (summer 2014) and the commencement of the fellowship (2016) making it difficult to plan specific activities in the unit. Some flexibility was required due to the uncertain nature of clinical trials particularly in the set-up phase when timelines can be fluid. This resulted in some adaptations to the training package.
**Conclusions**
CT Fellowships offer a unique opportunity for trialists of the future to get hands on experience at an early career stage and also to enable CTUs to develop researchers leading to high quality multi-centre trials. Both fellow & NCTU found the experience highly beneficial and strongly support continuation of this NIHR training programme.

### P362
### Quality assurance (QA) challenges in the development of international trials in rare diseases
Clare Cruickshank[1], Steve Nicholson, Curtis Pettaway[2], Nick Watkin[3], Jelle Teertstra[4], James Gimpel[5], Elizabeth Miles[6], Cathy Corbishley[3], Pheroze Tamboli[2], Stephanie Burnett[1]
[1]The Institute of Cancer Research; [2]M.D. Anderson Cancer Center; [3]St. George's University Hospital NHS Foundation Trust; [4]The Netherlands Cancer Institute; [5]American College of Radiology, Center for Research and Innovation; [6]National Radiotherapy Trials QA (RTTQA) Group
**Correspondence:** Clare Cruickshank
*Trials* 2017, **18(Suppl 1):**P362

inpact (CRUK/13/005, EA8134) is an international trial in penis cancer developed under the auspices of the International Rare Cancers Initiative (IRCI). It evaluates the combination and sequence of four common treatments for penis cancer: Inguinal Lymph Node Dissection (ILND), chemotherapy, chemoradiotherapy, and Pelvic Lymph Node Dissection (PLND). The interventions used within the trial present a number of QA challenges to ensure that any differences in trial outcomes are related to the randomisation schedules and not deviations from the trial protocol. The rarity of the disease means that, whilst networks of specialists have developed, experience at an individual clinical team level can be limited. A number of international specialist subgroups were therefore set up during protocol development to discuss areas of QA need and to agree on QA processes for the trial.
Surgical procedures can be difficult to standardise due to the number of factors involved, including the surgeon's skill and experience, and decisions taken regarding the surgical procedure based on patient characteristics or fitness, variations in anatomy, etc. Each surgical procedure will, therefore, be open to variability which the QA programme within inpact aims to minimise. Discussions among international surgical collaborators have led to agreement on precise surgical details to be included in the trial protocol and supplementary surgical trial guidance notes. Each surgeon will be accredited before participation in the trial. Accreditation will involve independent review of a number of surgical procedures by the inpact surgical QA committee comprising US and UK surgical leads for the trial. During the trial, photographs and operative notes will be reviewed and feedback will be given to individual surgeons at participating sites.
The randomisation schema within inpact requires knowledge of lymph node involvement. Correct interpretation of protocol criteria is crucial. Initially, prospective central review of all patient scans (to assess lymph node involvement) prior to randomisation was envisaged, but during protocol development it became evident that the logistics of this would be prohibitive. The ECOG-ACRIN Cancer Research Group and The Netherlands Cancer Institute shared anonymised images to enable development of a web-based teaching and testing solution using the ACR Radiology Curriculum Management System. Radiologists responsible for assessing patient lymph node involvement at each of the participating sites will be assessed through this training tool and accredited prior to the trial opening at that site.
Other areas identified as requiring international QA consensus were pathology and radiotherapy, the latter being led by the UK's NCRI Radiotherapy Trials QA Team and in the US, the National Clinical Trials Network's QARC. The organisation of separate QA subgroups in addition to the standard trial set-up processes and protocol development has been challenging, but the QA programme ultimately underpins the quality of trial treatment in this rare cancer. Regular international communication and the sharing of knowledge and experience with existing national QA processes and infrastructure have ensured consensus on trial protocol and associated QA. Internationally-harmonised QA programmes should optimise deliverability of this trial across multiple countries.

### P363

#### Variability in adverse event reporting rates per subject by enrollment site in a multicenter acute care clinical trial

Erin Bengelink[1], Valerie Stevenson[1], Jordan Elm[2], Sharon Yeatts[2], Robert Silbergleit[1]
[1]University of Michigan; [2]Medical University of South Carolina
**Correspondence:** Erin Bengelink
*Trials* 2017, **18(Suppl 1):**P363

**Objective**
Substantial variability in adverse event (AE) reporting practices may exist between sites, particularly in multicenter clinical trials involving patient populations for whom AEs are prevalent. Variability is likely to be multifactorial, involving differences in training, culture, documentation, and other parameters, but also, perhaps, upon the quality of trial performance. We hypothesize that sites with very low or very high numbers of AEs reported are more likely to also have excessive data corrections identified during source document review by site monitors.
**Methods**
In a recently completed randomized clinical trial of acute treatment of patients with traumatic brain injury (protect NCT00822900), we retrospectively determined the coincidence of enrollment sites being outliers on both AE reporting and data corrections found by site monitoring. Outlier sites were those outside 95% boundaries on funnel plots of AE reporting and of data corrections. Variability in AE reporting was assessed by examining the average number of AEs reported at each site (the total number of AEs reported at a site divided by the number of subjects enrolled at that site). Data correction at each site was assessed as the average number of data clarification requests (DCRs) written by a site monitor during source document verification visits that resulted in the site correcting erroneous data in the case report form (CRF). Analysis of coincidence was descriptive in this exploratory study. Sensitivity analyses using 90% boundaries and looking at only serious AEs (SAEs) were also visualized.
**Results**
882 subjects were enrolled at 49 sites between 2010 and 2013. 11 sites that did not enroll any subjects in the study were excluded, leaving 38 sites for inclusion. Site enrollment ranged widely from 1 to 85 subjects with a median of 18. The average number of reported AEs by site ranged from 0.5 to 12 (median 3.14). The average number of DCRs resulting in data correction by site ranged from 0.75 to 15 (median 3.56). On funnel plots, 14/38 (37%) sites were outliers with regard to AE reporting outliers (6 low, 8 high), and 7/38 (34%) were outliers with regard to high data correction rate. Coincidence was suggestive but not significant given the small numbers; 4/14 (29%) of the AE reporting outliers were also high data correction outliers, as compared to only 3/24 (13%) of the sites that were not AE reporting outliers. Unexpectedly, among the 4 coincident outliers, 2 were high and 2 were low AE reporting outliers. Findings were similar using 90% boundaries and rates of SAE reporting.
**Conclusions**
Extensive variability in both AE reporting and data collection quality exceed that expected by chance alone in this example trial. AE reporting rates may be useful as a metric to incorporate into risk-based site monitoring plans if similar patterns are found with larger numbers of sites across additional clinical trials.

### P364

#### Regulation in Latin America and its impact on the execution of multinational clinical trials to evaluate vaccines

Sara Valencia
phd Student Science and technology studies
*Trials* 2017, **18(Suppl 1):**P364

**Objective**
The primary objective of this paper is to explain the differences between the Mexican, Colombian, and Brazilian clinical research regulations and how these influenced the evaluation and implementation of multi-national vaccine trials in these three countries.
**Background**
In 2005, the Panamerican network for the harmonisation of pharmaceutical regulation (Red PARF in Spanish), based on the ICH guidelines, introduced the Document of the Americas for the Good Clinical Practices (DA-GCP) with the aim of harmonising clinical research practices in the region. The DA-GCP was not mandatory to all regulatory authorities. Therefore, each country had the independence to develop their guidelines and regulations to allow clinical trials. Colombia and Brazil in 2008 presented their resolution to implement clinical research in the country adopting the ICH-GCP guidelines, and in 2012 Mexico did the same. However, only Colombia and Brazil stated in their regulation the adoption of DA-GCP. Therefore, the question that emerges is how does the difference on the normativity between these countries have influenced the sponsor strategies to coordinate, manage and implement multinational clinical trials in Latin America?
**Methods**
To answer this question, in first place three multi-site clinical trials to evaluate vaccines were studied in Mexico, Colombia, and Brazil to assess the influence of the national regulation on multinational projects. Also in each country, members of clinical research associations were interviewed to understand better the local dynamics and the relationship between local normativity and the pharmaceutical industry. Sixty-six semi-structured interviews were conducted with members of the research site, sponsors, clinical monitors, ethic committees, regulatory agencies, and members of clinical research associations.
**Results and conclusion**
This qualitative study reveals that despite Red PARF efforts to harmonise GCP in the American continent, this objective has not been achieved in practice. Between Colombia, Brazil, and Mexico, it does not exist a harmonisation which is reflected in four aspects. 1) The divergence on requirements and procedures to approve the trial. 2) The number of institutions involved in protocols evaluation. 3) The restriction of Colombia regulation to hire certain professional profiles to be part of research teams 4) The research capabilities requested by each regulatory agency to implement the trial. These differences made that each sponsor had to develop management strategies to implement the vaccine trial in Colombia, Brazil, and Mexico which demanded: 1) coordinate times among different countries to start their trials. 2) Invest in the creation of research capabilities to implement its protocol. 3) Hire smos to coordinate trials at local levels and manage research sites, and 4) design new training strategies to create a knowledge-base among all clinical teams according to the local requirements. In conclusion, despite Red PARF's efforts, harmonisation of clinical trial regulation in Latin America has not been achieved. The difference between regulatory frameworks induced the creation of unique strategies by sponsor to coordinate and management the evaluation and implementation of multinational clinical trials in the region.

### P365

#### A study aimed at improving the conduct and efficiency of trials by developing a standardised set of site performance metrics and a systematic approach to reporting

Diane Whitham[1], Julie Turzanski[1], Alan Montgomery[1], Lelia Duley[1], Shaun Treweek[2], Paula Williamson[3], Lucy Culliford[4], Mike Clarke[5], Julia Brown[6], Louise Lambert[7]
[1]Nottingham University; [2]Aberdeen University; [3]Liverpool University; [4]Bristol University; [5]Queen's University; [6]Leeds University; [7]CRN National Coordinating Centre (CRNCC) | NIHR Clinical Research Network (CRN), Leeds
**Correspondence:** Diane Whitham
*Trials* 2017, **18(Suppl 1):**P365

**Background**

Standardising the collection, reporting and monitoring of data relevant to site performance could improve the effective and efficient oversight of clinical trial delivery. Our surveys of UK Trial Manager Network (UK TMN) members and NIHR chief investigators revealed wide variations in how trial data are used to assess performance. However, without consensus on optimal ways of utilizing performance metrics, trialists may focus on too many or uninformative indicators, causing inefficiency in trial conduct and difficulty in comparing between studies. Ideally,

This project aims to improve trial conduct and efficiency by: Reaching consensus on important metrics that should be monitored routinely in multicentre trials. Establishing initial baseline benchmark indicators for each performance metric for trending and predicting potential issues, so minimizing their impact and improving trial performance and efficiency. Developing a standardised systematic method for reporting and presenting these metrics to trial mangers, tmgs and tscs.

**Research Plan**

Small focus groups of stakeholders will establish an initial list of performance metrics and parameters that could be measured routinely in trials. We will then design a Delphi survey using data from literature searches and the focus groups to develop a comprehensive list of performance metrics and parameters for inclusion in the Delphi survey.

The Delphi survey will be sent to Trial Managers and CTU directors as they play key roles in ensuring the efficient delivery of multicentre trials. Three Delphi rounds will be used to steer the groups towards consensus, on a list of important performance metrics. We will document the reasons for their decision-making with regard to selection of metrics.

Data from the Delphi survey will be presented to stakeholders in a priority-setting workshop with a wide range of trial stakeholders, providing participants with the opportunity to express their views, hear different perspectives and think about monitoring of site performance. We will seek agreement on the top key performance metrics (expected to be around 8–12 in number) and benchmark indicators for each metric to trigger action to improve site performance.

Finally we will develop a simple tool (probably within Excel) for the presentation of key metrics to Trial Managers, Trial Management Groups and Trial Steering Committees in a standardised format.

Key Stakeholders: Trial Managers, Clinical Trials Units, NETSCC, NIHR Clinical Research Network, Chief Investigators, Statisticians.

**Results**

We will present the outcomes of the focus groups and literature search and discuss the design and development of our Delphi survey questionnaire.

**Discussion**

The project will result in a reporting tool showing a standardised set of clear, meaningful and easily accessible performance metrics. The metrics will assist researchers to indicate change over time and identify potential problem areas early, allow better utilisation of resources and timely action to be taken.

### P366

#### Can site recruitment be predicted? Results of a retrospective, blinded evaluation of a site selection questionnaire in five multicentre trials

Diane Whitham, Dawn Coleby, Wei Tan, Julie Turzanski, Lelia Duley
Nottingham University
**Correspondence:** Diane Whitham
*Trials* 2017, **18(Suppl 1):**P366

**Background**

Good sites are vital to ensure that multicentre randomised controlled trials (RCTs) are delivered on time, within budget and to a high standard. For example, over optimistic recruitment targets often mean the trial goes over budget and fails to complete on time. To help improve trial efficiency, site selection questionnaires (SSQs) to gather relevant information about potential sites are considered 'best practice' for selecting new sites in multicentre trials. However, there is limited evidence about their effectiveness in improving trial conduct.

This study aimed to evaluate the performance of an SSQ developed by the Nottingham Clinical trials Unit (NCTU), using data on key metrics collected from five randomised controlled trials. Previously presented preliminary data comparing mean number of days to recruit the first participant found that sites selected by both the Chief Investigator (CI) and by blinded assessment of SSQs were 68% more likely to have recruited their first participant than those where the CI and the blinded assessment disagreed (Trials 2015, 16(suppl 2):P176.).

We now update our study and present data assessing how well the SSQ predicted site recruitment.

**Methods**

For each of the five trials, SSQs were developed using questions that were both generic and protocol specific. The SSQ was emailed to the Principal Investigators (PIs) for potential sites, requesting its completion and return. The Chief Investigators (CIs) had access to these responses, and it was at their discretion whether they used the information to select sites.

For sites selected by the CI, each completed SSQ was assessed by an assessor who was 'blind' to the site and to the PI. This assessment used seven pre-defined criteria: SSQ not returned, potential pool of participants, available staff resources, clinical trials experience of PI, competing trials for target population, number of trials competing for resources, and equipoise for the trial interventions. If any one of the first three criteria was not satisfied, the site was excluded.

For CI selected sites, the monthly recruitment rates (actual/target) of sites that were and were not selected by blinded assessment of the SSQs from these sites were compared.

**Results**

An SSQ had been returned for all sites selected by the CI. There were 105 sites across the five trials. Three trials had completed recruitment, and overall sites had been recruiting for between 0.7 months and 47 months.

The median monthly recruitment (actual/target) was higher in the 54 sites selected by blinded assessment of the SSQs (median recruitment =78.5) compared with the 51 sites not selected by the SSQ assessment (median recruitment =50.0) ($p = 0.0019$, Mann-Whitney U test).

**Conclusion**

For CI selected sites, where the SSQ was reviewed by a blinded assessor, those that were selected based on the blinded assessment seemed to perform significantly better in terms of recruitment than those rejected following blinded assessment. This suggests that SSQs have potential as a tool to improve the selection of sites for clinical trial. They merit further development and evaluation as to whether they can improve efficiency of trial conduct.

### P367

#### Conduct of a precision medicine trial: screening, tissue adequacy, study registration, and reasons for not participating on lung-map (lung cancer master protocol)

Katie Griffin, Shannon McDonough, Jieling Miao, James Moon, Mary W. Redman
SWOG Statistics and Data Management Center at FHCRC
**Correspondence:** Katie Griffin
*Trials* 2017, **18(Suppl 1):**P367

**Background**
The Lung-MAP trial (Lung Cancer Master Protocol), launched in 2014, is an umbrella protocol to evaluate targeted therapies in biomarker selected patients for previously-treated stage IV or recurrent squamous non-small cell lung cancer. The trial infrastructure also includes a "non-match" study or set of studies for patients without any of the biomarkers under study. Lung-MAP, conducted by SWOG, and involving the National Clinical Trials Network of the National Cancer Institute (NCI), is the first precision medicine trial launched with the support of the NCI in the United States.
**Methods**
Lung-MAP has two steps, a screening step followed by sub-study registration step. In the screening step, tissue is submitted to determine patient eligibility for biomarker-selected or non-match sub-studies. For patients with tissue that is determined to not be adequate for biomarker testing, either additional tissue or tissue from a fresh biopsy can be submitted for retesting. Patients can either be screened at progression on therapy or pre-screened while receiving therapy for stage IV or recurrent disease. The trial did not open with the pre-screening option; this option was added at the end of 2015. The protocol-specified targets are that patients screened at progression receive their sub-study assignment within 16 days from tissue submission and pre-screened patients receive their sub-study assignment within one day of notifying the study they have progressed on the prior treatment. If at any point in time it is determined that a patient will not enroll on a sub-study, the site submits a form noting the reasons for not registering.
**Results**
As of November 4, 2016, 1075 patients have registered to be screened (714 (66%) screened at PD, 361 (34%) pre-screened). Upon initial submission, about 12% of submitted tissue was inadequate, with the most common reason being an insufficient amount of tissue (N = 58). Patients resubmit tissue samples about 37% of the time and 79% of those were analyzable; the tissue inadequacy rate overall is 8.8%.
Once a patient's tissue has been successfully tested, the patient is assigned and can then register to a Lung-MAP sub-study. To date, 785 patients have been notified of their sub-study assignment and 387 patients have registered to a sub-study.
Of the total 1075 registrations, 496 (46%) have submitted the form noting that a patient will not register to a sub-study. Of note, patients without a matching biomarker who previously received immunotherapy are not currently eligible for any sub-study.
**Discussion**
Conduct of a complex trial platform including biomarker testing and evaluation of multiple investigational therapies may continue to be a valued approach for evaluating biomarker/investigational therapy combinations. Lessons learned and views into their conduct are important to help inform future endeavors.

### P368

#### Measurement methods for eliciting opinions on treatment benefits, toxicities and acceptable trade-offs of the two, within the PERSEPHONE trial

Louise Hiller[1], Shrushma Loi[1], Anne-Laure Vallier[2], Donna Howe[1], Peter Bell[1], John Carey[1], Uzma Manazar[1], David Cameron[3], David Miles[4], Andrew Wardley[5]
[1]Warwick Clinical Trials Unit, University of Warwick; [2]Cambridge Clinical Trials Unit – Cancer Theme; [3]University of Edinburgh Cancer Research Centre; [4]Mount Vernon Cancer Centre; [5]The Christie NHS Foundation Trust
**Correspondence:** Louise Hiller
*Trials* 2017, **18(Suppl 1):**P368

PERSEPHONE is a phase III non-inferiority RCT comparing six months of trastuzumab to the standard twelve months in patients with HER2 positive early breast cancer. The primary endpoint is disease-free survival (DFS), with cardiac function as a secondary endpoint. It was assumed that the standard 12 months trastuzumab results in 80% DFS at 4 years. With 5% 1-sided significance and 85% power, 4000 patients gives the ability to prove non-inferiority of the experimental arm, defining non-inferiority as no worse than 3% below the estimated 4-year DFS of the standard arm. The trial reached its 4000 patient target in July 2015, making this UK trial the largest of its kind in the world.
Whilst waiting for the follow-up data to mature, we embarked on designing a survey to canvass current clinician's opinions on trastuzumab duration that would provide insight into not only the potential practice-changing impact of PERSEPHONE'S results, but also the most appropriate non-inferiority limits to define for the future meta-analysis of the "twelve month trastuzumab versus less" trials for further investigation into pre-specified sub-groups of patients.
The survey aimed to record opinions on what clinicians considered the effectiveness of each of PERSEPHONE'S two randomised treatment durations, followed by what difference between them they would require the results to prove in order to change their current practice. Opinions on the two randomised arms' rates of cardiotoxicity were also collected. The next section of the survey depicted various hypothetical scenarios of cardiotoxicity differences between treatment arms, with responders asked what trade-off they would require in terms of the primary endpoint of DFS to change their current practice within those scenarios.
Exploration of possible measurement methods to best collect opinions on trade-off levels was undertaken. One option explored was a simple cross-tabulation of hypothesised levels of DFS and cardiotoxicity. Another avenue explored was the use of gaming chips placed on separate continuums of perceived "costs" and "benefits" of the two treatment arms. To assist in interpretation of the trade-off between perceived advantages and disadvantages, graphical aids were also considered. One option investigated used pictures of old fashioned weighing scales with two pans, one representing one treatment arm with a hypothesised DFS and cardiotoxicity level, and the other just a hypothesised cardiotoxicity level. Responders were asked to choose the level of DFS required to make the scales balance.
Eliciting clinician's opinions on acceptable trade-offs within one trial endpoint for various levels of detriment in another endpoint is a complex one. Surveys were sent out to all 152 hospitals involved in the PERSEPHONE trial. Results will be presented of the success of the methodology adopted to undertake this task.

### P369

#### Standard operating procedures for managing adverse events in trials that do not involve an investigational medicinal product: a protocol for a Delphi consensus study

Guy Peryer[1], Catherine Minns Lowe[2], Yoon Loke[1], Catherine Sackley[3]
[1]University of East Anglia; [2]University of Hertfordshire; [3]King's College, London
**Correspondence:** Guy Peryer
*Trials* 2017, **18(Suppl 1):**P369

**Background**
Medical research methods, technologies and tools evolve rapidly. It is essential guidance prioritising the safety of human volunteers is reviewed at timely intervals. This study aims to provide clarity and consistency to the assessment and reporting of adverse events in clinical trials that do not involve an investigational medicinal product (non-CTIMP). Non-CTIMP governance covers a broad spectrum of non-pharmacological disciplines (e.g. Surgery, nutrition, psychological and physical therapies etc.). Currently, this is a neglected area of clinical trial research. The lack of consistent identification, categorization, and reporting of harms prevent researchers from conducting reliable meta-analyses and comprehensive systematic reviews on the benefits and risks of non-drug interventions that help to guide clinical practice. Non-systematic methods of assessing harms increase the potential for reduced effect sizes, resulting in a bias towards the null

(Type II error). Critically, a lack of evidence of harm does not equate to evidence of safety.
The study will address variability in practice, defined in Standard Operating Procedures, that UK Clinical Trials Units (CTU) have in place for: i) defining, ii) classifying, and iii) reporting adverse events in non-ctimps. Compared to drug trials, adverse events in non-ctimps are not managed well. There is considerable inconsistency in reporting styles between trials of similar design and intervention type.
To promote increased consistency, we will conduct a consensus exercise among non-CTIMP experts using a Delphi technique followed by a face-to-face meeting. This method adheres to the recommended sequence outlined by the international network for Enhancing the Quality and Transparency of Health Research (EQUATOR) for developing health research guidelines.
A non-CTIMP expert is defined as: a CTU representative, a Chief Investigator or trial manager of non-ctimps with >3 trials experience in this role, or a senior member of the Health Research Authority's Operations team or Ethics Committee. As such, the participants in the consensus exercises will also be the direct beneficiaries from the project maximising its pathway to impact.
Following the face-to-face meeting, guidance and explanatory statements will be drafted. The guidance statement will focus on: How adverse events should be defined in relation to the non-pharmacological intervention, How CTU standard operating procedures should be designed to reflect the results of the Delphi exercise, How adverse events should be classified following a judicious causal assessment, and Recommended reporting methods that will promote more effective meta-analyses of non-pharmacological interventions that provide a balanced benefit-harm evaluation. Following study completion, we will work with a selection of UK CTUs to evaluate the implementation of any agreed modifications to current practice.
In addition to the protocol design the poster will present preliminary survey data collected with 70 chief investigators of non-CTIMPs. The survey questions and results are attached. Questions covered a series of themes evaluating the range of inconsistency in defining, categorizing and reporting serious adverse events, and evaluated preferences for increased harmonisation in this area.

### P370

#### Improving the quality of NIH funded clinical trials

Carmen Rosa
National Institute on Drug Abuse
*Trials* 2017, **18(Suppl 1):**P370

The National Institutes of Health (NIH), as the largest public funder of clinical trials in the United States, recognizes the importance of clinical trials and well as recognizes the major challenges in the design, efficiency and reporting of clinical trials. Over the years, NIH has funded trials that are too complex, have small sample sizes, rely on surrogate endpoints, have unrealistic enrollment goals, inadequate budgets, etc. Many times these trials are not published nor data submitted to a public site. On September 16, 2016, the NIH announced a series of efforts directed towards the improvement of clinical trials efficiency, accountability and transparency. This presentation will briefly discuss these activities, which are aimed to address the clinical trials process from the time new ideas are generated to sharing data to the public. The initiatives covers NIH review and selection of trials to fund, clinical trials management and oversight, and data sharing. More specifically, this presentation will discuss the variety of new NIH policies, including Good Clinical Practice (GCP) training requirements for investigator and NIH staff, using clinical trials specific Funding Opportunity Announcements (FOAs), including appropriate expertise to review sessions, using a protocol template (required for FDA studies), using a single Institution Review Board (SIRB), and utilizing clinicaltrials.Gov to register and upload results.

### P371

#### Placebo surgery trials in the NHS are possible

Naomi Merritt[1], David Beard[2], Andrew Carr[2], Cushla Cooper[2]
[1]University of Oxford; [2]NDORMS, University of Oxford
**Correspondence:** Naomi Merritt
*Trials* 2017, **18(Suppl 1):**P371

Placebo Surgery Trials in the NHS are Possible Introduction Placebo surgery trials are controversial and are not routinely conducted in the NHS. Evidence related to the management of such studies is limited and teams planning a placebo surgery trial need to carefully consider how to manage such a trial.

**Background**

CSAW is a multicentre randomised placebo controlled blinded surgical trial assessing the effectiveness of arthroscopic sub-acromial decompression surgery versus an arthroscopy alone (the placebo or sham procedure) versus a period of active monitoring with specialist reassessment. Previously, a placebo surgery trial has been deemed difficult to run in the NHS with additional challenges for the study management team. These include (and supported by the literature) increased concerns regarding risk, ethics, perceived patient deception, ability to recruit and the surgical community acceptance of the placebo procedure.

**Methods**

A variety of strategies were utilised for the success of the trial. These included; Inclusion of a medical ethicist on the investigator team; A longer than normal set-up phase of the study for educating sites about placebo surgery; A pre-trial survey where surgeons interested in participating in the CSAW outlined their practices, followed by in-depth interviews between the surgeons and the study's clinical leads; Use of a Prospective Patient Assessment (PPA) at the main site. This involved presenting a hypothetical placebo surgery trial to patients to gain feedback and ask whether they would consider participation. A Qualitative Recruitment Investigation (QRI) was also undertaken in the early phases of the trial to observe transparency of information given to patients and to assess the level of surgeon equipoise. Standard evaluation of the frequency of study procedures was also undertaken.

**Results**

The strategies resulted in successful recruitment to the study. Feedback showed the benefit of involving the participating surgeons in defining the placebo procedure arm. Regular monitoring of the study showed surgeons were fully compliant with the restrictions of the placebo operation (the arthroscopy only). The placebo element was not an issue in relation to recruitment nor in implementation of the study arms. The PPA completed showed 90% of patients would be interested in participating in a placebo surgery trial. Feedback on the hypothetical study also informed the research ethics application. The QRI generated a "top tips for recruitment" list and enabled training on the best approach to patients. CSAW successfully reached their recruitment target of over 300 patients, recruited from 25 NHS sites.

**Conclusion**

With effective strategies in management and monitoring a placebo surgery trial is possible in the NHS. No major challenges were faced in the conduct of the study. CSAW has now successfully completed and results will be published early 2017.

### P372

#### Randomization balance in multicenter clinical trials with short drug life and rapid allocation

Jeff Szychowski, Alan T. N. Tita, Gary R. Cutter
University of Alabama at Birmingham
**Correspondence:** Jeff Szychowski
*Trials* 2017, **18(Suppl 1):**P372

#### Introduction
The logistics of allocating an assigned drug treatment in a multicenter randomized controlled clinical trial may be complicated by a short drug shelf life and by the need for rapid allocation after randomization. We conduct a simulation study to examine the effects on randomization balance under these conditions, where the ratio of rates of drug preparation and recruitment at study centers vary from low to high. We further explore practical strategies to address these logistical needs.

#### Background
Our study is inspired by the Cesarean Section Optimal Antibiotic Prophylaxis (C/SOAP) trial, a double-blind, pragmatic, randomized clinical trial conducted at 14 hospitals in the United States. Women with a singleton pregnancy, at least 24 weeks gestation, and undergoing nonelective cesarean delivery were randomized to receive either azithromycin (500 mg in 250 ml saline) or an identical-appearing saline placebo prior to incision. All women were to receive standard prophylaxis (cefazolin) prior to incision. The 250 ml bags were prepared in advance by investigational pharmacists according to site-stratified randomization schemes, kept in a secure refrigerator, and had a 7 day shelf life. Only the investigational pharmacists who prepared the study drug had access to the randomization scheme through a dedicated password-protected website. Randomized women received the next sequentially numbered study bag in the refrigerator. Expired study bags were discarded.
At each site the investigational pharmacists prepared a pre-specified number of study bags to be used or discarded within 7 days. Given pharmacy costs and constraints, bags were typically prepared once per week. The number of prepared bags was estimated to be the number of patients enrolled over the course of the next 7 days. In successful recruiting weeks, all prepared study bags were used. In lower enrollment weeks, bags were discarded. Because of these constraints, the rates of study drug preparation and randomization were continually monitored and modified as needed.

#### Methods and Results
We simulate the effects of underutilization of prepared study drug on randomization balance and total drug waste. We consider different combinations of randomization schemes (fixed blocks of 2, 4, and 6, and variable block designs), total number randomized by site, and ratio of prepared-to-used study drug. Randomization balance is lost, and waste increases, as the preparation rate exceeds the randomization rate.

#### Conclusions
It is extremely important that we understand the characteristics of potentially suboptimal randomization procedures, as they may lead to increased waste, increased costs, and randomization imbalance. The complications introduced by short drug shelf life and by the need for rapid randomization are important in multiple contexts including labor & delivery and emergency medicine. We discuss strategies to optimize resources and minimize waste.

### P373
### Development of complex interventions to prevent falls and fractures in older people living in the community: the prevention of fall injury trial (PREFIT)
Susanne Finnegan[1], Julie Bruce[1], Emma Withers[1], Dawn Skelton[7], Ranjit Lall[1], Shvaita Ralhan[2,], Ray Sheridan[3], Katherine Westacott[4], Finbarr Martin[5], Sarah Lamb[6]
[1]The University of Warwick; [2]Oxford University Hospitals NHS Trust; [3]Royal Devon & Exeter NHS Foundation Trust; [4]University Hospitals of Coventry and Warwickshire; [5]Guys and St Thomas' NHS Foundation Trust; [6]The University of Oxford; [7]Glasgow Caledonian University
**Correspondence:** Susanne Finnegan
*Trials* 2017, **18(Suppl 1):**P373

#### Background
Falls are the leading cause of accident-related mortality in older adults. Injurious falls, including fractures, are associated with functional decline, loss of independence, disability, and significant health and social care costs. Although numerous trials have been conducted to investigate the efficacy of falls preventions strategies on rate and risk of falls, there is a lack of strong, robust evidence for multifactorial or exercise interventions in preventing fractures. We developed two complex falls prevention interventions for evaluation within the framework of a large multicentre, pragmatic, randomised controlled trial (RCT) (ISCTRN 71002650).

#### Methods
Prefit is a three-arm, cluster RCT, conducted within primary care across England. We aimed to recruit 9000 participants, aged 70 and above, from 63 general practices. Practices were randomised to deliver one of three falls prevention interventions: (1) advice only; (2) advice with exercise; (3) advice with multifactorial falls prevention (MFFP). The Age UK Staying Steady booklet was sent to all trial participants. The process of developing the complex 'active' interventions is described below. Development of the PREFIT Exercise Intervention: We undertook a review of systematic reviews of exercise interventions to prevent falls in community-dwelling older people. Based upon evidence from Cochrane systematic reviews and UK clinical guidelines, we shortlisted three standardised programmes for possible inclusion. To reflect the pragmatic nature of the trial, we also reviewed surveys of NHS falls services and conducted consensus work with clinicians working in falls prevention. We selected the Otago Exercise Programme which targets balance and strength, using ankle weights. The intervention is a six-month programme, which is individualised and supported by trained therapists who prescribe exercises and progress over time.

#### Development of the PREFIT MFFP
Intervention: Multifactorial interventions are defined as that where individuals receive 'an assessment of known risk factors for falling and an intervention matched to their risk profile'. To determine which risk factors to include in PREFIT, we considered evidence from systematic reviews, including a large Cochrane review of 34 RCTs of multifactorial interventions to prevent falls in older people and evidence examining the effectiveness of MFFP delivered in primary care. We referred to clinical guidelines from the American Geriatrics Society (AGS), British Geriatrics Society (BGS) and National Institute for Health and Care Excellence (NICE) to further inform selection of risk factors. In addition, we elicited a range of views from clinical and practice experts within the field of falls and bone health. The final PREFIT MFFP intervention comprised of seven risk factors which were assessed on every participant referred for treatment. The model was based upon individual assessment and onward referral to specialist services where indicated. Results Pilot study We undertook a pilot study in 12 general practices (n = 1801) in Devon to determine the acceptability and feasibility of delivering an exercise programme and a complex MFFP intervention to older adults recruited from primary care. The interventions were then rolled out to other regions within the main trial.

#### Conclusions
These complex interventions are currently being evaluated within the largest multicentre falls prevention clinical trial conducted in the UK.

### P374
### Sample size calculations using bayesian optimisation
Duncan Wilson[1], Richard Hooper[2], Rebecca Walwyn[1], Amanda Farrin[1]
[1]University of Leeds; [2]Queen Mary, University of London
**Correspondence:** Duncan Wilson
*Trials* 2017, **18(Suppl 1):**P374

#### Background
Finding the optimal sample size for a trial is an important step in its design. In many trials of complex interventions (such as psychotherapies and behavioural interventions) this task is complicated by two factors. Firstly, sample size can be defined by several design parameters rather than a single n. For example, a trial which compares a psychotherapy intervention with treatment as usual may be partially nested, with patients nested within therapists in the intervention arm but not in the control arm. The design parameters of such a trial are the number of therapists in the intervention arm, the number of patients seen by each therapist, and the number of patients in the control arm. The second complication is that analytical formulae for calculating power are not always available. As a result power must instead be estimated through Monte Carlo simulation methods, which may be computationally demanding. For example, such a simulation of the TIGA-CUB study would involve the generation of a large number of hypothetical trial data sets and fitting a multilevel model to each one. In combination, these factors make finding an optimal sample size a difficult and time consuming problem. We explore how modern optimisation algorithms can be used to solve these problems in an effective, timely manner.

**Methods**

We propose using Bayesian optimisation to solve the sample size problem. This method allows optimal or near-optimal choices for sample size to be found with minimal computational effort. The general approach involves the careful choice of the design parameter values where power should be estimated using simulation. Conducting the simulations at these points, a statistical model is then fitted to the output to describe the general relationship between the design parameters and the trial power. This model is then used to find the smallest design parameter values which will give power of at least the nominal level. The method is flexible, can be used for almost any problem for which power can be estimated using Monte Carlo simulation, and can be implemented using existing statistical software packages.

**Evaluation**

To illustrate the approach we apply it to a partially nested psychotherapy trial in an illustrative case study. We use the proposed method to identify a set of candidate sample size options, each of which will give power of at least the nominal rate. From this set of options, that which is considered best in terms of its balance between number of therapists and number of patients can be chosen. We compare this with an alternative approach using simpler heuristics, in terms of both the computation time required and the quality of the resulting solutions.

**Conclusions**

Bayesian optimisation can be an effective technique for performing sample size calculations when power must be estimated using simulation, particularly when sample size is characterised by several design parameters. By improving the efficiency of these calculations, increasingly complex sample size problems can be solved without the need for unrealistic simplifying assumptions.

### P375

#### Could adaptive research designs be useful in designing an effective ehealth intervention? A methodological analysis

Ram Bajpai, Josip Car
Lee Kong Chian School of Medicine, Nanyang Technological University
**Correspondence:** Ram Bajpai
*Trials* 2017, **18(Suppl 1):**P375

**Background**

The use of ehealth or digital health interventions has increased due to rapid growth in information and communication technologies (ICTs). This results quick appearance and change in digital interventions that challenge the robust designing of such interventions. The traditional intervention designs are somehow incapable to tackle specific challenges of digital interventions. It is therefore important to explore innovative research designs to handle the unique challenges of ehealth interventions.

**Objective**

This methodological research aims to analyse how different adaptive research designs could be used in evaluation of digital behaviour change interventions (DBCIs) without altering the nature of randomized designs.

**Methods**

An adaptive design allows modifications during the trial based on the reports from interim analysis. We reviewed Medline available literature related to adaptive designs and hand searched relevant medical and statistical journals. We also assessed the published and ongoing (registered at www.clinicaltrials.gov/) ehealth interventions from Jan 2011 to Oct 2016 to search any evidence of adaptive designs in ehealth or digital health interventions.

**Results**

We did not find any evidence so far of using adaptive designs either in ongoing or published digital intervention trials literature. Therefore, we discuss different adaptive design choices conceptually such as adaptive randomisation design, group sequential design, sample size re-estimation design, hypothesis-adaptive design and seamless phase II/III design etc. Literature suggested that sequential, seamless phase II/III and multi-arm multi-stage designs could improve efficiency and maintain ethical considerations; changing- sample size and hypothesis designs could handle the uncertainty and be flexible to define end points; and enrichment designs could handle heterogeneity among responses and ease the data monitoring process. On the other hand, multiple adaptive designs in a single trial require more control in execution and should be handled with care. However, other adaptive designs such as treatment-switching and dose-finding are yet to explore their usability in relation to ehealth intervention. There are some statistical challenges that need addressing when designing such trials. For example, any adoption to the design may increase the Type I error rate, difficulties in the analysis of trial data including Bayesian approach (especially in deciding prior distribution) and interpretation of results.

**Conclusions**

The adaptive designs showed potential to address various ehealth specific challenges. Such designs could lead to simplified operational complexities involved and make these interventions more efficient and cost-effective. There is a need to encourage researchers to use adaptive designs and set regulatory guidelines to handle practical challenges.

### P376

#### Developing a framework to aid purposive sampling in process evaluation of a critical care trial

Lydia Emerson[1], Danny McAuley[1], Mike Clarke[2], Thomas P. Hellyer[3], A. John Simpson[3], Bronagh Blackwood[1]
[1]Centre for Experimental Medicine, Queen's University Belfast; [2]Centre for Public Health, Queen's University Belfast; [3]Institute of Cellular Medicine, Newcastle University
**Correspondence:** Lydia Emerson
*Trials* 2017, **18(Suppl 1):**P376

**Background**

To answer the question ‘does a complex intervention work?’ in a way that distinguishes between failure of the intervention and failure of its implementation, an evaluation of the process of intervention delivery is required. Process evaluation data are collected from a sample of the practitioners involved in implementation and intervention delivery. However, the mechanism of selecting a sample is rarely described in the literature. The aim of this project was to define a framework to purposively sample clinicians in a trial conducted in 22 intensive care units (ICUs) which used a new invasive test for detecting ventilator associated pneumonia in critically ill patients (the vaprapid-2 trial, ISRCTN65937227). Methods Data analysis of context and usual practice collected at the beginning of the trial, alongside qualitative data collected from doctors, nurses, and laboratory staff during the trial provided information on adoption and delivery of the intervention in the ICUs. From this information, we constructed themes describing what worked well, for whom and in what contexts in terms of intervention delivery. These themes were explored with clinicians at the end of the trial in order to identify factors and the mechanisms of their interaction that were likely to impact on trial outcomes. We purposively sampled 40% of ICUs for end of trial interviews, ensuring that we obtained maximum variation in barriers and facilitators to the trial.

**Results**

In the analysis of data collected before and during the trial, we identified five themes. To enable easier sampling, we grouped the themes into two broad categories to form a framework: (1) ICU situation which reflected (positive or negative) issues with laboratories, workload, staff availability, and fitting the trial into the ICU; and (2) perceived risk (classed as high or low risk). This categorisation enabled sites to be

'mapped' onto the framework. In addition to these themes, we also examined recruitment data to assess the reach of the intervention i.e. The percentage of eligible patients who were actually recruited into the trial. We subsequently sampled ICUs from each of the four cells that also captured the variation in reach.

**Conclusion**

Little information is available on the methods that might be used for purposive sampling of practitioners implementing new interventions in critical care research. We suggest a novel and practical method of categorising data to produce a framework to guide maximum variation sampling.

### P377

#### Minimising bias in surgical RCTs through blinding: a systematic review

Sian Cousins, Katy Chalmers, Kerry Avery, Natalie Blencowe, Sara Brookes, Jane Blazeby, M. Kobetic, T. Munder
University of Bristol
**Correspondence:** Sian Cousins
*Trials* 2017, **18(Suppl 1):**P377

**Background**

Blinding, the process of withholding knowledge of treatment allocation from participants and trial personnel, is critical in the design of RCTs. It may reduce differences between trials groups in the assessment of outcomes (detection bias), in the way interventions and co-interventions are delivered (performance bias) and in withdrawals from the trial (attrition bias). In addition, it may minimise bias in the interpretation and reporting of analyses if data analysts are successfully blinded. Indeed if there is inadequate blinding this can exaggerate estimates of treatment effects by up to 25%. Blinding, however, can be hard to achieve and maintain in trials assessing non-pharmacological interventions such as surgery. Challenges specific to surgical trials include, but are not limited to, difficulties in delivering a control intervention indistinguishable from the active intervention, and blinding personal who deliver the intervention. This systematic review will describe the current methods used to blind participants, intervention providers, care givers, outcome assessors and data analysts in trials of invasive surgical interventions across all surgical specialties. In addition, we will present examples of trials where blinding was not attempted but may have been possible, outlining how novel methods of blinding may be utilised. Presently we outline results to date with a complete report available in March 2017.

**Methods**

A systematic search was carried out in Medline (ovidsp), Embase and CENTRAL databases for articles published between January 2006 and June 2016 in the top 10 surgical and general medical journals according to impact factor. Articles eligible for inclusion were RCTs of invasive interventions, in which blinding of any participants or trial personnel had been attempted. We define an invasive procedure to be where a cut is made or access to the body is gained via cutting, instrumentation via a natural orifice or percutaneous skin puncture where instruments are used in addition to the puncture needle. Trials in which a medicinal product is delivered via an invasive procedure and where there is administration to targeted anatomical districts or where an action is performed internally to administer the product, will be included. General study characteristics will be extracted, in addition to data regarding blinding specifically. Blinding status of participants and trial personnel, method of blinding, instances where blinding may have been possible but was not attempted and details of any reported tests of success of blinding will be extracted. Quality of included trials will also be assessed using the Cochrane Risk of Bias tool.

**Results**

The search retrieved 3946 articles. 1873 duplicates were removed and 613 were removed on basis of journal. 1460 titles and abstracts were screened for eligibility using standardised screening forms. 1129 articles were excluded after abstract review and 331 articles were included for full text review. Full results will be available by March 2017 and reported at the meeting.

**Discussion**

We outline and summarise the methods of blinding used in high quality surgical RCTs assessing invasive interventions. We highlight good practice and will make recommendations for future research in this field to minimise bias in RCTs in surgery.

### P378

#### Cost effective interactive live clinical data monitoring dashboards with drill-down

Amarnath Vijayarangan
Emmes Services Pvt Ltd.
*Trials* 2017, **18(Suppl 1):**P378

Periodical data review is very important and highly recommended for all the ongoing clinical studies to ensure the data integrity and quality. Each clinical study requires experts from various functional groups like SAS programming, Biostatistics, Data Management and so on. Each one of them have various data review requirements and also one cannot expect everyone to familiar with SAS programming as clinical datasets are often available as SAS datasets. Statisticians prefer summary level data whereas others might need to look at the summary level as well as granular level data. These reports are static and hence end users do not have any choice to customize or drill down the reports on their own. Currently it is always directed to a SAS programmer to update the reports which is an overall time consuming process. Every clinical study is constrained with budget and it might be expensive for them for a sophisticated tool. These dashboards are created only once for each study using SAS, Excel VBA & Excel Pivot Table and they are –Multi user access at a time –Live Interactive Summary Reports and Graphs –No Programming is required for the end user –100% Menu Driven –Auto Refresh –Custom Filters –Drill Down to Raw Data The master dashboard excel file can be copied by each user to their local machine and each one can play around with the locally saved reports without impacting the master dashboard & source files. The following are the challenges faced during the developmental stage along with their solutions.

1. SAS Formats cannot be applied while creating excel files using proc export Solution: SAS macro program is written to handle the format issue.
2. The Source data path changes of Pivot reports while moving the reports Solution: There is a wide discussion can be seen in various online forums on how to solve the pivot source data path changes while moving the files with pivot tables. None of the solutions discussed online are simple and reliable. The simplest solution is open the file and using SAVE AS option save it in the desired location instead of the coping the master dashboard. How simple it is.
3. Pivot based reports are static with the number of rows in excel. But for an ongoing study, this will be increasing and every time user cannot change their reports for the rows. It becomes tedious to manually do the same on several reports and it is error prone. Solution: While creating the pivot reports, 50000 rows are selected and Whenever any SAS dataset exceeds 50000 rows, email notification is sent.
4. Date based filtering criteria is one of the frequently used criteria for the data monitoring. For example, number of subjects enrolled during last 1 week or 1 month. Excel pivot does not provide an option to filter data using specified date ranges. Solution: Fortunately, this is solved using the concept of SLICER available in excel 2010 onwards.

This paper proposes an easy and cost effective approach to develop an Interactive Live Clinical Data Monitoring Dashboards with Drill-Down Using SAS & Microsoft Excel.

### P379

#### Data management lifecycle evaluation

Michelle Steven
Edinburgh Clinical Trials Unit
*Trials* 2017, **18(Suppl 1):**P379

Data management processes are required to ensure clinical trial data is high-quality and captured according to protocol and regulatory requirements; a critical phase within clinical research. The Edinburgh Clinical Trials Unit (ECTU) Data and IT Systems Team are small yet well-established with a wide ranging remit from CRF ?A3B2 show $132#?>design, data entry, and data cleaning through to the design and hosting of complex bespoke electronic data capture (EDC) systems. Historically, the majority of the EDC systems developed were used in studies fully supported by ECTU, with an identified project manager assigned to the study who completed many of the data management tasks together with the IT Systems Team. However, there has been a recent demand for support only from data management, with ECTU developing EDC systems for trials with external trial coordination. In addition, with the continued growth of the Trial Management teams, there was significant variation in CRF design and data management procedures between the individual trial managers.
Subsequently, there was a need identified within ECTU for a formalised procedure for data management activity, in particular where there is no allocated Trial Manager. By first establishing a designated Data Manager role, ECTU have undertaken a schedule of evaluation and improvement procedures to redefine data management activity within the unit. The entire lifecycle of the evaluation of the existing data management activity and formalisation of new procedures will be detailed, including the development of an ECTU data management plan and its standardised content. Through ongoing monitoring and feedback from Trial Managers and external clients, future plans will also be identified, including suggestions for future discussion with the wider data management community.

### P380

#### Data validation traffic light system: data from the tranexamic acid for intracerebral haemorrhage (TICH-2) trial

Katie Flaherty, Lelia Duley, Zhe Law, Philip M. Bath, Nikola Sprigg
University of Nottingham
**Correspondence:** Katie Flaherty
*Trials* 2017, **18(Suppl 1):**P380

##### Background
Data validation in clinical trials is an important procedure. Real time validation checks may not pick up all errors and these need correcting. A flagging system would help trial staff be more time effective by preventing re-checking of problems that cannot be resolved. A traffic light system was created and implemented at the Nottingham Stroke Trials Unit.
#### Methods
The NIHR HTA Tranexamic acid for intracerebral Haemorrhage (TICH-2) trial is a randomised, controlled, international, multicentre trial aiming to recruit 2,000 patients. Data volume in such a trial is large, and having numerous centres makes solving data queries time consuming. The database in TICH-2 has built in logic checks allowing real time validation and this identifies most but not all data inconsistencies. A program was created that looks at the data and checks it for values which lie outside the normal ranges or are inconsistent between different data fields, this picks up queries that the real time validation missed to ensure data is as complete as possible. The checks are run for one centre at a time and sent to trial coordinators, within the trial coordinating centre, to go through them and resolve with sites. Coordinators highlight each of the checks in either green, orange or red. Green shows the query has been resolved, usually due to incorrect data that has been checked with the recruiting site and updated. Orange means it has not been resolved because the site has yet to respond; such queries may be resolved at a later date. Red indicates that data cannot be resolved or is correct as is and the statistician should remove the value from further data checks.
#### Results
This traffic light system has proven easy to follow and understand and has been effective at getting queries organised. It ensures coordinators are not re-checking queries that cannot be resolved. The data checks were run recently for TICH-2, so all data are not yet available. Twenty seven queries were raised from a sample of 14 centres; 6 (22%) of these were coded green and were data corrected, the remaining 21 (78%) were red and removed from the checks.
This system also led to changes in ECRFs; it was noticed that participant weight was often missing on the baseline form, so this was moved to the day 2 form to give hospital staff more time to get the information. Since the change, the number of completed entries has increased from 69% to 75% ($p = 0.03$).
#### Conclusion
Putting in place small changes and guidelines can improve staff productivity and save duplication of their workload. Having a system which all staff can easily implement and use helps everyone keep track of what has been done to deal with data queries. The investigations that come from these queries improves data quality and efficiency of trial conduct. A similar approach is used in our BHF RIGHT-2 trial.

### P381

#### Changing platforms without stopping the train : a data management perspective on the operational aspects of adaptive platform trials

Dominic Hague[1], Stephen Townsend[1], Lindsey Masters[1], Mary Rauchenberger[1], Matthew R. Sydes[1]
[1]MRC Clinical Trials Unit at UCL, Institute of Clinical Trials and Methodology, UCL, London, UK; [2]MRC London Hub for Trials Methodology Research, London, UK
**Correspondence:** Dominic Hague
*Trials* 2017, **18(Suppl 1):**P381

##### Background
There is limited research and literature on the data management challenges encountered in adaptive platform trials. This trial design allows both (i) seamless addition of new research comparisons, and (ii) early stopping of accrual to individual comparisons that do not show sufficient activity without affecting other active comparisons. FOCUS4 (colorectal cancer) and STAMPEDE (prostate cancer), run from the MRC CTU at UCL, are two leading UK examples of clinical trials implementing adaptive platform designs.
To date, STAMPEDE has added four new research comparisons, closed two research comparisons following pre-planned interim analysis (lack-of-benefit) and completed recruitment to six research comparisons. FOCUS4 has closed one research comparison following pre-planned interim analysis (lack-of-benefit) and added one new research comparison, with a number of further comparisons in the pipeline. We share our experiences from the operational aspects of running these adaptive platform trials, focusing on data management.
[Note: For lessons learnt from a central trial management perspective, see our companion abstract]
#### Methods
We critically reviewed data management challenges in STAMPEDE and FOCUS4. These included implementation of case report forms (CRFs), Clinical Data Management Systems (CDMS), randomisation systems, report development, documentation, and other operational challenges. We also sought specific challenges arising from electronic (FOCUS4) or paper (STAMPEDE) CRFs.
#### Discussion
We found similar adaptive platform trial-specific challenges in both trials. Adding and removing comparisons to open trials provides extra layers of complexity to CRF and CDMS development. At the start of an adaptive platform trial, CRFs and CDMS must be designed to be scalable in order to cope with the continuous changes in this trial design, ensuring future data requirements are considered where possible. When adding or stopping a comparison, the challenge is to incorporate new data requirements while ensuring data collection within ongoing comparisons is unaffected. Some changes may apply to all comparisons; others may be comparison-specific or only applicable to patients recruited during a specific time period. We will discuss the advantages and disadvantages of the different approaches to CRF and CDMS design we implemented in these trials, particularly in relation to use and maintenance of generic versus comparison-specific CRFs and CDMS.
The work required to add or remove a comparison, including the development and testing of changes, updating of documentation, and training of sites, must be undertaken alongside data management of ongoing comparisons. Adequate resource is required for these competing data management tasks, especially in trials with long follow-up. A plan is needed for regular and pre-analysis data cleaning for comparisons that could recruit at different rates and times. We will discuss the ways that these data cleaning activities can be split and prioritised.

**Conclusions**

Adaptive platform trials offer an efficient model to run randomised controlled trials but setting-up and conducting the data management activities in these trials can be operationally challenging. Trialists and Funders must plan for scalability in data collection, and the resource required to cope with additional competing data management tasks.

### P382

#### Operationalizing and testing a minimization randomization algorithm for use in clinical trials

Sheila Burgard[1], James Bartow[1], Hope Bryan[1], Sonia Davis[1], Steven Offenbacher[1], Souvik Sen[2]
[1]University of North Carolina; [2]University of South Carolina
**Correspondence:** Sheila Burgard
*Trials* 2017, **18(Suppl 1):**P382

**Background**

Randomization seeks to balance background factors across treatment groups through random assignment, and stratified randomization imposes structure to help ensure balance of the stratification factors. Yet randomization schemes that are over-stratified may not result in treatment group balances in factors related to disease outcome, sometimes making study interpretations difficult. Using dynamic randomization algorithms in trials with a small sample size relative to the number of stratification groups may improve the co-factor balance, allowing for a clearer study signal. Periodontal Treatment to Eliminate Minority Inequality and Rural Disparities in Stroke (PREMIERS) (NIH Minority Health and Health Disparities Research) is a 2-center phase III randomized, controlled trial that will enroll 400 patients to test whether intensive periodontal treatment reduces the risk of recurrent vascular events among ischemic stroke and TIA survivors. Researchers wanted to ensure balance of 4 different factors: race, stroke severity, socio-economic status, and stroke risk.

**Method**

The Collaborative Studies Coordinating Center (CSCC) in the Department of Biostatistics at the University of North Carolina serves as the data coordinating center for the trial. A dynamically allocated biased-coin minimization algorithm (Pocock & Simon, Biometrics 1975) was designed and programmed at the CSCC as an application called from the randomization electronic case report form (ecrf) from the CSCC-developed, web-based data management system, Carolina Data Acquisition and Reporting Tool (CDART). Each time a patient is to be enrolled, the site enters the enrollment data, including all of the stratification factors into the ecrf. The randomization application within CDART calculates an imbalance score for each purported treatment assignment that is based on stratification co-factor levels of previously enrolled, randomized patients. The lowest imbalance score has some influence when the treatment is assigned.

**Objective**

CDART has the functionality for complex calculations and custom reports, yet operationalizing the dynamic allocation application was new to CDART. A separate application developed in SAS was used to validate the randomization algorithm within the data management system is working as expected. The presentation will show the details of implementing the algorithm, the additional steps to safeguard against changes in stratification data after a patient has been randomized, and the double-programmed validation job confirming that the treatment assignments are being correctly assigned.

### P383

#### Impact case study: using PRECIS-2 to discuss the design of a pilot and feasibility study - summary of swallowing intervention package (SIP)

Kirsty Loudon[1], Mary Wells[2], Emma King[2]
[1]University of Stilring; [2]Nursing, Midwives and Allied Health Professions Research Unit
**Correspondence:** Kirsty Loudon
*Trials* 2017, **18(Suppl 1):**P383

**Background**

The PRECIS-2 tool was developed to help trialists match their design decisions to the information needs of those they hope will use the trial results. PRECIS-2 has a highly visual wheel format with nine design domains including Eligibility, Recruitment, Setting, Organisation and Primary outcome which are scored on a Likert scale from "1" Very explanatory - Ideal world, to "5" Very pragmatic - Just like usual care.
The tool was developed to design Randomised Controlled Trials and is being used internationally by health care professionals in a variety of clinical areas. The aim of our study was to evaluate the usefulness of PRECIS-2 for discussing the design of a feasibility study of an intervention developed by a trial team including speech and language therapists, oncology nurses, and a psychologist: a Swallowing intervention Package (SIP).

**Methods**

The SIP team applied the PRECIS-2 tool to assess the pragmatism of the SIP feasibility study design, using training materials and the software on the PRECIS-2 website www.PRECIS-2.org to create their own sip study wheel. The individual PRECIS-2 scores of the SIP trial team were aggregated into a single PRECIS-2 wheel to indicate the median and range of scores for each of the nine PRECIS-2 domains. The trial team then used aggregated scores to discuss the design of the feasibility study and reach consensus. Discussion focussed on domains with the greatest range of scores (i.e. Widest discrepancy between individuals). Members of the team were asked to complete an evaluation form to determine the utility of using the tool with a view to designing a future randomised trial.

**Results**

Ten out of the 14 members of the sip trial team used the PRECIS-2 tool to assess the pragmatism of the SIP feasibility study. Prior to the meeting, the discrepancy between domain scores was up to 3 points on a scale of 5, suggesting differing views of how explanatory or pragmatic the study design was. The PRECIS-2 domains with greatest consensus were Eligibility - To what extent are the participants in the trial similar to those who would receive this intervention if it was part of usual care, and Setting - How different are the settings of the trial from the usual care setting. Some of the trial group believed that certain aspects of the feasibility study for the sip trial were quite explanatory, with four out of nine of the domains scoring "2": Flexibility (Adherence), Follow up, Primary Outcome and Primary Analysis. Following discussion, the team reached consensus on scoring 7 out of 9 domains, assessing the overall design of the sip feasibility study as more pragmatic than explanatory. With all but one of the meeting participants independently scoring the sip trial using PRECIS-2, this enabled meaningful discussion of the key elements of a future trial design.

**Conclusions**

This exercise was useful for assessing the design of both the SIP feasibility study and a potential future trial. PRECIS-2 is a relevant framework for reaching consensus on design aspects of pilot and feasibility studies as well as full trials.

### P384

#### Family perspectives on the feasibility of a corticosteroid induction regimen randomised controlled trial in juvenile idiopathic arthritis: results of a qualitative study

Frances Sherratt[1], Louise Roper[1], Eileen Baildam[2], Matthew Peak[2], Flora McErlane[3], Simon Stones[4], Bridget Young[1]
[1]University of Liverpool; [2]Alder Hey Children's NHS Foundation Trust; [3]Great North Children's Hospital, Newcastle Hospitals NHS Foundation Trust; [4]University of Leeds
**Correspondence:** Frances Sherratt
*Trials* 2017, **18(Suppl 1):**P384

**Background**
Corticosteroids (CS) are key to achieving rapid disease control in children and young people (CYP) presenting with new or flaring juvenile idiopathic arthritis (JIA). Efficacy, duration of action and side effect profiles vary with the route of administration. Current routes of CS administration are based on physician and patient preference, rather than scientific evidence. A randomised controlled trial is needed to ascertain the most effective routes and doses of CS. This paper will report on the feasibility of a potential CS induction regimen randomised controlled trial (RCT) in JIA from the perspective of CYP and their families.
**Methods**
Semi-structured in-depth interviews were conducted with a purposive sample of CYP with JIA and their families, recruited via rheumatology clinics across the UK. All CYP and their families had recent (<12 months) experience of CS treatment. The Framework Method was used to support thematic analysis of the data.
**Results**
Findings will be reported on CYP'S and parents' experiences of four different CS delivery routes, treatment preferences and willingness to participate in a CS induction regimen RCT, randomising patients between different steroid delivery routes and dose regimes. CYP's and parents' questions about the planned RCT and their recommendations regarding its design will also be reported.
**Discussion**
The findings from this qualitative study will inform judgements about the feasibility and design of a future RCT of CS induction regimens for JIA. Corticosteroids are a very effective treatment for flaring JIA but carry significant and challenging side-effects. The views and experiences of CYP with JIA are an important outcome of this study and are key to informing the feasibility and acceptability of a future RCT from the patient perspective. Methodological issues about the validity of pre-trial feasibility studies for informing decisions about potential future trials will also be considered.

### P385

#### Overtreatment of DCIS – 4 years on from the marmot review - progress with the LORIS trial

Jennie Young[1], Adele Francis[2], Jeremy Thomas [3], Matthew Wallis[4], Andrew Hanby[5], Lesley Fallowfield[6], Valerie Jenkins[6], Lucy Matthews[6], Patricia Fairbrother[7], Maggie Wilcox[7], John Bartlett[8], Lucinda Billingham[9], Sarah Bowden[9], Claire Gaunt[9], Daniel Rea[9], Tracy Roberts[9], Sarah Pinder[10]
[1]University of Birmingham; [2]University Hospitals Birmingham; [3]Western General Hospital; [4]Addenbrooke's Hospital; [5]University of Leeds; [6]SHORE-C, Brighton and Sussex Medical School; [7]Independent Cancer Patients' Voice; [8]Ontario Institute for Cancer Research; [9]University of Birmingham, Cancer Research UK Clinical Trials Unit; [10]King's College London
**Correspondence:** Jennie Young
*Trials* 2017, **18(Suppl 1):**P385

The Marmot independent review of breast screening report published in 2012 concluded that the breast screening programme saves lives but significant overtreatment exists [1]. The review called for randomised clinical trials to address overtreatment of screen-detected DCIS.
More recently, Sagara et al. [2] conducted a retrospective longitudinal cohort study in the USA investigating the survival benefit of breast surgery for DCIS. They found no significant difference in breast cancer specific survival between patients who had surgery and those who did not in the low grade DCIS group.
LORIS is a multi-centre, randomised controlled phase III trial of surgery versus active monitoring in patients with low risk DCIS designed to address the problem of overtreatment.
An independent patient advocate group (Independent Cancer Patients' Voice) have been involved in the study since conception and provided a direct patient perspective throughout the LORIS Trial.
Key eligibility criteria are; women aged 46 years and over with screen-detected or incidental microcalcification with no previous invasive breast cancer, no comedo necrosis and low risk disease confirmed by Central Digital Histopathology Review. The primary outcome is ipsilateral invasive breast cancer free survival up to 5 years.
34 sites have opened to recruitment across the UK, with enthusiasm, commitment and support evident from the site research teams. Patients have responded favourably to the trial and the opportunity to contribute to ground breaking research. Patients found the Patient Information DVD informative and welcomed receiving information early on in the patient pathway.
Reasons patients gave for participating in the trial include "the trial offered the best treatment options" and that "others would benefit from the results of the trial". Patients have also commented that they welcome the opportunity for independent pathology review of their biopsies; a key quality assurance element of the trial. Feedback from sites highlights the difficulty in conveying to patients the rationale of replacing surgery with active monitoring, with 30% of patients stating a preference for surgery. To address this, regular communication workshops are offered, phone-in sessions allow sites to share best practice and frequent newsletters offer hints and tips to aid recruitment. Media channels are being explored to raise public awareness of the overtreatment of DCIS. Dissemination of this information will help to engage future LORIS patients.
Following on from the successful launch of LORIS in the UK, the trial is now leading the way internationally. LORIS is collaborating with COMET (USA) and LORD (Netherlands).
Collaborative efforts and feedback from site staff, patients and oversight committees during the LORIS feasibility phase has allowed us to establish LORIS as a world leading trial addressing the overtreatment of DCIS.
The LORIS Trial is funded by the National Institute for Health Research Health Technology Assessment programme.

**References**
[1] Marmot MG, Altman DG, Cameron DA, Dewar JA, Thompson SG, Wilcox M. The benefits and harms of breast cancer screening: an independent review. Br J Cancer. 2013;108(11):2205–40.
[2] Sagara Y, Mallory MA, Wong S, Aydogan F, desantis S, Barry WT, et al. Survival Benefit of Breast Surgery for Low-Grade Ductal Carcinoma In Situ: A Population-Based Cohort Study. JAMA surgery, 2015.

### P386

#### Statistical analysis plans for internal pilots in randomised controlled trials

Cliona McDowell[1], Evie Gardner[2], David Harrison[3], James McNamee[4], Daniel F. McAuley[5]
[1]Northern Ireland Clinical Trials Unit; [2]Northern Ireland Clinical Trials Unit, Belfast; [3]Intensive Care National Audit & Research Centre, London; [4]Regional Intensive Care Unit, Royal Victoria Hospital, BHSCT and Wellcome-Wolfson Institute for Experimental Medicine, Belfast; [5]Northern Ireland Clinical Trials Unit, Regional Intensive Care Unit, Royal Victoria Hospital, BHSCT and Wellcome-Wolfson Institute for Experimental Medicine, Belfast
**Correspondence:** Cliona McDowell
*Trials* 2017, **18(Suppl 1):**P386

**Background**
It is common practice for clinical effectiveness trials to include an internal pilot but guidance on how they should be analysed is lacking. The REST study is a pragmatic randomised controlled trial to determine whether Veno-Venous Extracorporeal Carbon Dioxide Removal

(VV-ECCO2R) in mechanically ventilated patients with hypoxaemic respiratory failure improves 90 day mortality. An internal 6-month pilot study in 10 sites to confirm both recruitment and adherence assumptions that have contributed to study design will precede the main trial.

**Method**

A statistical analysis plan (SAP) has been written for the internal pilot for the REST trial which will be signed off separately to the full trial SAP but will form an appendix to it once it's written. It covers the analyses required for the internal pilot to assist with making the decision to progress to the full trial. For the REST internal pilot the following analyses will be completed: The overall recruitment rate will be compared to target recruitment of 7 per month. Analysis of separation in terms of tidal volume (ml/kg predicted bodyweight) between the two arms will be undertaken on an intention to treat and a per protocol basis including the patients in receipt of VV-ECCO2R on day 2 and 3. Completeness of datasets with respect to the primary outcome measure of 90 day mortality will be assessed for patients recruited in the first 3 months. Like a full trial SAP, the SAP for the internal pilot was reviewed by the Data Monitoring and Ethics Committee and the Trial Management Group.

**Discussion**

A formal SAP for an internal pilot is not standard clinical trial methodology and there are pros and cons to having one which are open for discussion. In general, the advantages are as for the SAP for the final analysis: to pre-specify the analysis and reporting in order to avoid intentional or unintentional bias caused by multiple unplanned analyses and selective reporting of data. Without a SAP, investigators who are convinced of the usefulness of an intervention may manipulate pilot results to make a convincing case for progression to a full trial ultimately leading to a trial showing no difference and a waste of research funding. On the other hand, one may argue that the risks of multiple analyses and "data dredging" leading to spurious findings are primarily associated with large sample sizes that will not be present in a pilot study. Furthermore, relying too strictly on pre-specified criteria for progression to a full trial could result in stopping a trial of a promising treatment in the presence of practical issues with delivery that may be easy to overcome. One specific pro to having a SAP for the internal pilot that is separate from the full SAP is the ability to have the pilot analyses documented without the need to have the full SAP signed off at an early stage of the trial. The SPIRIT guidelines should give consideration to internal pilot studies and the need to document planned analyses and progression criteria.

### P387

#### Pilot and feasibility studies: a systematic review of studies conducted at the population health research institute

Amparo Casanova, Shun Fu Lee, Chinthanie Ramasundarahettige, Salim Yusuf
[1]Population Health Research Institute, Mcmaster University
**Correspondence:** Amparo Casanova
*Trials* 2017, **18(Suppl 1):**P387

**Background**

Weaknesses in the design, conduct and reporting of pilot/feasibility studies have

Been shown by systematic reviews. Moreover, lack of agreement on the definition of a pilot or a feasibility study has made difficult to propose guidelines on the analyses and reporting of these studies, which often ended up not being published. The working group on developing reporting guidelines on pilot and feasibility studies, as an extension to the Consolidated Standards of Reporting Trials (CONSORT) Statement, created a conceptual framework to propose definitions for pilot and feasibility studies (Eldridge et al. 2016), and has recently published their guidelines (Thabane et al. 2016). We propose to systematically review the pilot and feasibility studies conducted at the Population Health Research Institute, McMaster University (our group) in the last 10 years, and assess the application of the new guidelines.

**Methods**

We will select all studies that were defined as pilot or feasibility studies in the study protocol, as well as any study that agrees with the definition of pilot or feasibility studies under the guidelines. We will follow the guidelines and report all of the elements proposed (e.g. Design, sample size calculation, objectives, type of outcomes, analyses, and whether they were used to estimate parameters that supported sample size calculations for a full trial). We will also report whether the study was an internal pilot or not, and whether the results of the pilot/feasibility study were published separately from the main trial.

**Conclusion**

A systematic review of pilot and feasibility studies under the recently published CONSORT guidelines may improve understating their application in a practical setting, and easy the implementation amongst researchers.

### P388

#### Investigating the impact of phase II trial design parameters on the ability to successfully screen new treatments for phase III trials

Nada Elbeltagi[1], Sarah Brown[1], Nigel Stallard[2], Julia Brown[1], Walter Gregory[1], Fiona Collinson[1]
[1]University of Leeds; [2]University of Warwick
**Correspondence:** Nada Elbeltagi
*Trials* 2017, **18(Suppl 1):**P388

**Background**

The purpose of phase II trials is to assess the potential activity of new therapies, which will then be investigated in larger, more time and resource consuming phase III trials. Currently, in oncology, the success rate of phase III trials is considerably low, suggesting that the phase II trials are allowing the progression of too many inefficacious therapies. Thus, early rejection of inactive treatments and moving therapies that are most likely to be efficacious to phase III testing would benefit patients and the drug development industry. The aim of this research is to investigate the impact of various design parameters on the phase II trials' ability to successfully screen new treatments in phase III.

**Methods**

A literature review was conducted in order to determine which phase II design parameters have previously been considered for impact on phase III outcomes, and to identify any additional parameters that may be considered. It also aimed to identify an appropriate measure of success which allows comparisons of phase II trial efficiency to be made. The impact of the varying design parameters on the identified measure of success will then be evaluated.

**Results**

The literature review identified a number of parameters to be investigated, including phase II sample size, distribution of the underlying true treatment effect and phase II design. Additional parameters we will investigate are the strength of relationship between endpoints at phase II and III, and the population size. From the literature, a number of measures were found to quantify the impact of these parameters on the success of phase II trials, including the number of patients required in both phases II and III before the first successful phase III.This measure takes into account the expected probability of success in phase II, E(P_1), and the expected probability of success in phases II and III, E(P_1 P_2). Since the phase III trial will only commence if the phase II trial is successful, these probabilities can be used to calculate the conditional probability of success at phase III, E(P_1 P_2)/(E(P_1)).Using this measure, we analytically evaluate the impact of each of the design parameters on the ability to successfully screen treatments at phase II. To start with, the phase II and phase III trials are assumed randomised, two-arm trials, with continuous normally distributed endpoints in both phases II and III, and with an underlying true treatment effect that follows a normal distribution.

**Conclusions**

The impact of the strength of relationship between the endpoints, and sample size, on the conditional probability of success of phase III trials will be presented. Understanding the impact of each of the design parameters on phase III trials will allow us to design efficient phase II trials which can more quickly move effective treatments to phase III testing.

### P389

#### A lifetime disease model based on an RCT: development, validation and applications

Iryna Schlackow[1], Borislava Mihaylova[2], on behalf of SHARP Collaborative Group[2]
[1]University of Oxford; [2]Nuffield Department of Population Health, University of Oxford
**Correspondence:** Iryna Schlackow
*Trials* 2017, **18(Suppl 1):**P389

**Background**

Chronic kidney disease (CKD) increases the risk of cardiovascular disease (CVD) but, conversely, CVD may accelerate kidney disease progression.

**Aim**

To develop, validate and make widely accessible a CKD disease model that takes into account interdependence of CKD and CVD and can be used to simulate disease outcomes and (quality-adjusted) life expectancy and incorporate effects of interventions that modify cardiovascular risk in CKD patients.

**Methods**

The model is based on the individual patient-level data from the Study of Heart and Renal Protection (SHARP), which included 9,270 participants with moderate-to-severe CKD but no major coronary disease. Detailed sociodemographic and clinical characteristics at study entry and serious adverse events during the 5 years' follow-up were recorded. A two-stage lifetime Markov model was developed, comprising of a CKD submodel simulating progression of chronic kidney disease, and a CVD component simulating annual experience of fatal and nonfatal cardiovascular events and non-vascular death. Risk equations included baseline characteristics as well as important time-updated covariates (age, cardiovascular event and CKD status). Three external CKD cohorts (an observational study, CRIB, and two randomised controlled trials, 4D and AURORA) were used to validate the model. Additionally, the model performance was compared against an external CVD risk score (the Pooled Cohort Risk Equations, PCREs) and an external risk score of progression to end-stage renal disease (the model by Tangri et al.).

**Results**

Age, recent cardiovascular events and contemporaneous CKD status were the most important determinants of adverse events. Predicted event rates from the model corresponded mostly well to those in the external cohorts. The model was superior to the PCREs in predicting cardiovascular risk, with predictions by the PCREs not adequately capturing variation in risk across CKD stages especially in CKD stage 3B (observed 5-year probability of a major vascular event in SHARP 8.6 [95% confidence interval 6.2-11.0] vs 9.4 predicted by the SHARP model vs 15.3 predicted by the PCREs) and dialysis (19.3 [16.2-22.2] observed in SHARP vs 17.9 predicted by the SHARP model vs 12.4 predicted by the PCREs). In predicting end-stage renal disease, the model performance was comparable to that of the Tangri model ?A3B2 show $132#?>(observed 5-year probability of end-stage renal disease in SHARP 36.5 [35.2-37.8] vs 34.2 predicted by the SHARP model vs 34.3 predicted by the Tangri model).

To facilitate the use of the model, a practical and flexible web interface was developed, which allows the user to execute analyses on individuals or patient cohorts and provides estimates, including the uncertainty, of the disease outcomes, life expectancy and cost-effectiveness of interventions. We will illustrate the model, and its interface, with examples of potential applications.

**Conclusions**

The SHARP CKD-CVD lifetime risk model is a novel resource for simulating lifetime health outcomes, and effects of interventions to reduce cardiovascular risk and kidney disease progression, in people with moderate-to-severe CKD. The freely available web-based interface allows for a wide range of policy relevant analyses.

### P391

#### Extrapolation of utilities between disease progression and death in cancer trials for economic evaluation

Iftekhar Khan
University of Warwick
*Trials* 2017, **18(Suppl 1):**P391

**Background**

In many cancer trials, health related quality of life (HRQOL) are often collected up to disease progression. For health economic evaluation, a lifetime perspective of both costs and utilities is required to assess cost-effectiveness of cancer drugs. Therefore, ideally, utility data is required beyond disease progression. In some cases assumptions are made about the behaviour of utility data (e.g. Such as constant, linear decline, or decaying). Other options include the possibility to determine utilities from historical data and rarely is any attempt made to extrapolate utility data beyond trial follow up, although this is commonly used for survival data.

In this research, we demonstrate the feasibility of extrapolation of utilities beyond disease progression and standard trial follow up for the purposes of estimating quality adjusted life years (QALYs) over a life time horizon through non-linear mixed effects modelling.

**Methods**

Data from an observational study in 100 lung cancer patients followed up for at least 12 months was used to extrapolate EQ-5D-3 L utilities after patients have progressed. Several non-linear models were postulated including a Lorentz, Rational, 5-Parameter, Pareto, Exponential and Linear models. Extrapolation of survival times were generated using a Royston-Parmar (3 Knott) flexible parametric survival model in order to estimate the QALY. Models were compared in terms of AIC and impact on QALY estimates.

**Results**

Utilty extrapolation is feasible. The more complex 5-parameter model appears to be the most useful (lowest AIC value of 92.4) in terms of predictive ability beyond 12 months. Two parameters were statistically significant ($p < 0.001$). The Lorentz, Rational and 5-parameter models generated the most accurate estimates of mean PP utilities and QALYs: 0.474 vs. 0.508, 0.509 and 0.487 respectively for utilities; and 3.176 vs. 3.37, 3.37 and 3.26 for QALYs.

**Conclusion**

Modelling post progression utilities as well as extrapolation of utilities beyond the study follow up appears feasible and is an alternative to mapping or using published utility estimates.

### P392

#### Embedding trials into established service improvement initiatives: challenges and lessons from an implementation laboratory

Suzanne Hartley[1], Robert Cicero[1], Amanda Farrin[1], Jill Francis[2], Liz Glidewell[1], Natalie Gould[2], John Grant-Casey[3], Fabiana Lorencatto[2], Lauren Moreau[1], Simon Stanworth[3]
[1]University of Leeds; [2]City, University of London; [3]NHS Blood & Transplant
**Correspondence:** Suzanne Hartley
*Trials* 2017, **18(Suppl 1):**P392

There are opportunities to embed randomised trials of interventions to promote the uptake of evidence-based practice within established, large-scale improvement initiatives. Such "implementation laboratories" offer an efficient means of producing robust evidence generalizable to service settings. However, there are practical and methodological challenges in delivering such programmes of work.

Audit and Feedback (A&F) - defined as any summary of clinical performance of health care over a specified period of time, to provide healthcare professionals with data on performance - is widely used to improve the quality of healthcare. However, its effects are often unreliable, indicating the need for coordinated research including more head-to-head trials comparing different ways of delivering feedback.

Blood transfusions are a commonly used intervention in hospital care. Repeated national audits in the United Kingdom suggest that up to a fifth may be unnecessary when judged against recommendations for good clinical practice. The AFFINITIE programme

(Development & Evaluation of Audit and Feedback interventions to Increase evidence-based Transfusion practice) aims to test different ways of delivering feedback within the existing UK National Comparative Audit of Blood Transfusion (NCABT). It includes two linked 2x2 factorial, cluster-randomised trials evaluating two theoretically-enhanced A&F interventions to reduce unnecessary blood transfusions in hospitals. Trial outcomes were derived from data collected for the national audit.
A number of challenges related to, or increased by, embedding research within an existing national audit programme have been highlighted to date in the AFFINITIE programme. These include:
Communicating a message about equipoise to clinicians developing, delivering and receiving different feedback interventions who might otherwise feel advantaged or disadvantaged; Identifying and mitigating threats of contamination between the enhanced and standard feedback arms of the trial; Preserving fidelity of intended enhanced feedback interventions on the pathway to their delivery; Ensuring that data quality and governance processes sufficiently meet the needs of both a national audit programme and a rigorous evaluation; Aligning research timelines with those of a rolling and evolving national audit programme.
From a methodological perspective these challenges suggest a tension between internal and external validity (that is, bias and generalisability), characteristic of pragmatic trials, where greater importance is placed on generalisability.
Embedding research within major improvement initiatives is, however, feasible. We will present our recommendations in response to each challenge we have identified. These may assist researchers in optimising the conditions for sustainable implementation laboratories in the context of existing A&F programmes. These include:
Negotiating shared expectations and ground rules for collaboration; Establishing joint processes for assuring the quality of data for audit and research; Aligning audit standards and trial endpoints wherever possible.
Our recommendations will be discussed in the context of a wider literature on designing pragmatic trials.

### P393

#### Clinical trials in organ donation and transplantation in the UK - benefits and challenges

Laura Pankhurst, Dave Collett
NHS Blood and Transplant
**Correspondence:** Laura Pankhurst
*Trials* 2017, **18(Suppl 1):**P393

Clinical trials in organ donation and transplantation are increasing in the UK but each presents their own set of challenges. The centrally maintained UK Transplant Registry, held by NHS Blood and Transplant, prospectively collects data on all organ donors, transplants and transplant recipients, including periodic follow up data, and is a particularly valuable resource for the conduct of clinical trials in this field. In particular it facilitates power calculations, and data collection, especially for longer term outcomes. Alongside this, NHS Blood Transplant has a national network of Specialist Nurses for Organ Donation, who seek consent for organ donation and the use of such organs in a clinical trial. There is very little time from when the organ is deemed suitable for transplantation, the potential recipient is notified and the organ is transplanted. Consent from the recipient to participate, and randomised treatment allocation, usually takes place in this short window. Streamlining and simplifying of these processes is essential. Other challenges arise in trials in kidney transplantation, where deceased donors typically donate both kidneys, which have different anatomy, and are allocated in such a way a centre may receive one or both kidneys from a donor. Randomisation therefore requires careful consideration to take account of features such as this. Typically in transplantation, the key outcome of interest is graft or patient survival years. However, survival rates are high and differences to be detected are generally small, and hence such an outcome can rarely be used as the primary outcome. Composite outcomes, or biomarker data have then to be used.
Many of these challenges, and their resolution, will be illustrated using a randomised controlled trial of ex-vivo normothermic perfusion, compared to standard cold storage in kidney transplantation.

### P394

#### Developing a web-based central laboratory shipment scheduler and information system

Kayla Daniels, Michael Frasketi, Dikla Shmueli-Blumberg, Peter Dawson
The EMMES Corporation
**Correspondence:** Kayla Daniels
*Trials* 2017, **18(Suppl 1):**P394

The use of central laboratories in multi-site clinical trials is common to ensure consistency in reporting and analysis of assay results. The additional time between sample collection and analysis is typically not a burden for frozen or otherwise properly stored specimens. When assay results are time sensitive due either to the sample type (e.g. Fresh cells) or need for immediate results, local laboratories are often utilized. If an assay is novel or proprietary, however, the local laboratory may not be able to perform the assay and a specific central or research laboratory must be utilized. In several recent multicenter studies, an assay required shipment of whole blood samples and immediate labor intensive processing by a specific central laboratory. Despite extensive piloting to reduce time from collection to analysis and obtain quality assay results, an unexpected predicament developed during the trial in which sites were shipping more specimens than the lab could successfully test in a timely fashion due to limited central laboratory resources; resulting in failed assays. The coordinating center for these studies had to find an immediate solution that was convenient and accessible to both central laboratory and site staff. In response, coordinating center staff developed and deployed an access controlled shipment scheduler web site to allow sites and lab staff to more efficiently coordinate. Clinical site users entered shipment details and a validation routine capped each site at a certain number and type of clinical sample shipments. Central laboratory users could then monitor when specimens would be shipped to them and indicate lab closures or other dates shipments could not be accepted, thereby allowing better allocation of lab staff resources. Addition of the shipment scheduler to the laboratory management process resolved the difficulties involving laboratory capacity and the studies were able to continue obtaining novel, high quality assay results. This presentation will highlight some of the infrastructure and functionality of the system that may be relevant and applicable for studies with existing laboratory management systems or those interested in creating such a process. Furthermore, we will discuss the importance of flexibility among the clinical trial management team and the value of adapting processes to correspond with constantly changing requirements of a research trial.

### P395

#### Ensuring a GCP compliant audit trail for EDC/EPROM

Jonathan Gibb, Sharon Kean
Robertson Centre for Biostatistics
**Correspondence:** Jonathan Gibb
*Trials* 2017, **18(Suppl 1):**P395

##### Background

In September 2016 the UK competent authority in relation to clinical trial regulations, the Medicines and Healthcare products Regulatory Agency (MHRA) Ran an annual good clinical practise (GCP) symposium. One of the main topics discussed was data integrity and numerous common problems found during site inspections. The MHRA highlighted numerous deficiencies found in electronic systems, the majority of these were well known and it could be considered surprising that such deficiencies still exist. There was however one particular notice from the MHRA that has wide reaching implications for EDC systems as it is believed almost no systems are currently compliant. The MHRA highlighted the case that when a electronic record is saved, all of the variables on the page are saved with the identical timestamp. The timestamp represents the point of saving, not when the individual fields were actually entered. Furthermore, the MHRA highlighted that the audit mechanism in electronic systems usually operated in such a way that the electronic system could allow unaudited changes to data which would be captured on paper when following GCP guidelines. On paper, an edit to most pieces of data require that the piece of data be scored through once, and then a replacement value written down, dated and initialled. In most electronic systems, only the state of data at the time of saving is recorded; any changes made prior to saving are completely lost. One could argue that keyboard usage is more prone to error, therefore typographical errors should not be recorded. However, how could the system distinguish between typographical errors and change of answers? There is a very real potential cost to this requirement in terms of data storage. A naïve solution would be to capture the answer at every stage of entry; however this leads to huge overheads in data storage. If a surname is 10 characters long then typically that requires 20 bytes of storage, if however you storage each state of the surname as it is entered, assuming there are no actual edits or corrections then the storage requirement balloons to 100 bytes.

**Methods**

Various problems in audit trail mechanisms highlighted by the MHRA will be discussed along with common solutions from production systems. A detailed investigation at the problem of between save auditing will take place including discussion of various solution approaches and their merit on a number of different electronic platforms.

**Conclusion**

All staff involved with the creation, maintenance or usage of EDC systems should be aware of the GCP audit requirements highlighted by the MHRA in the UK. Even to those who deal with other competent authorities, as this is a direct GCP requirement, it is likely the issue will arise across all GCP bound component authorities.

### P396

#### The evil of eval() in CTMS – how to get more from user defined code

Jonathan Gibb, Sharon Kean, William Aitchison
[1]Robertson Centre for Biostatistics
**Correspondence:** Jonathan Gibb
*Trials* 2017, **18(Suppl 1):**P396

**Background**

An eval() mechanism is one which evaluates and executes code at runtime, it exists in different forms in almost every programming ?A3B2 show $132#?>language and platform. In computing it has long been known that the use of eval() mechanisms pose a security threat, the code being executed likely originated with the user of a system and therefore should be treated with caution. A large proportion of Clinical Trial Management Systems (CTMS) and Electronic Data Capture (EDC) systems allow users who design forms to type in snippets of code and then run these during system use by using an eval() mechanism. Eval() is employed despite the security concerns posed because it is an easy way to incorporate user defined rules into a system e.g. Form skip fill rules. The ability to pass the rule content to the underlying platform for execution without the requirement to understand what is being passed, drastically simplifies the development task when building the system. Apart from the security concerns, not understanding the user defined rules significantly limits the EDC'a abilities and function including but not limited to: All user entered code blocks are subject to GCP validation requirements however the EDC is limited in what tooling and support it can provide when the user defined code block is not understood e.g. Automated creation of test scenarios. Code blocks that are not "understood" cannot be translated; therefore one is limited to the language/platform they were written for initially. To run the code on any new platform, one has to essentially emulate the old platform in the new platform.

**Methods**

If the code blocks are "Understood" then they can be further translated to a non-programming language to allow all job roles – not just those who are fluent in the specific programming language – to verify that the code meets the requirements.

**Conclusion**

The authors will present an overview of different mechanisms to breakdown code blocks into understandable units from classic compiler design to more adhoc methods and the various things that can be achieved including: test data generation, automatic test execution, documentation production and programming language translation.

### P397

#### Developing tablet based EDC apps

Jonathan Gibb, Dionne Russell, William Aitchison, Sharon Kean
Glasgow Clinical Trials Unit
**Correspondence:** Jonathan Gibb
*Trials* 2017, **18(Suppl 1):**P397

**Background**

Sales in mobile devices are out stripping traditional PC sales and a number of analysts expect this trend to continue and the gap to widen. In line with this trend, the number of trials that wish to include mobile device based EDC systems has risen significantly in the last five years.

There are two main types of EDC: Native application based or web based. While the web based approach does support offline functionality, there are serious concerns over the security of data in offline web based systems with the current technology. Therefore, if you wish your mobile device EDC to function offline you are limited to native applications.

There are three main mobile device operating systems: android, ios and windows. Therefore having decided to produce a native application one must consider which operating system(s) you will build applications for.

Obviously building the same application for multiple operating systems seems counter intuitive. To this end, a number of products exist on the market offering a middle ground that allows you to build one application that will run natively on all three operating systems.

The authors utilised Xamarin, a system that advertises multiplatform support to allow applications to be delivered for multiple operating systems.

**Methods**

The authors have been involved in the delivery of eight separate mobile device EDC systems, supporting online and/or offline modes, four different languages, web based and native application and have gained quite a lot of experience in this area in a short time due to the nature of the studies involved.

**Conclusion**

The authors will present the best practises learned from delivering these applications in their various modes and explain why as an organisation we have decided to develop future native applications in their operating system specific development environment, despite the duplication of work, rather than rely on an intermediary platform like Xamarin.

### P398

#### The cocoa supplement and multivitamin outcomes study in the mind (cosmos-mind): design and rationale

Sarah Gaussoin, Mark A. Espeland, Sally A. Shumaker, Stephen R. Rapp, Darrin Harris, Debbie Pleasants, Julia Robertson, Laura D. Baker
Wake Forest School of Medicine
**Correspondence:** Sarah Gaussoin
*Trials* 2017, **18(Suppl 1):**P398

Identifying a safe, affordable, and well-tolerated intervention that prevents or delays cognitive decline in older adults is of critical importance. There is growing evidence from basic science and small randomized trials that cocoa flavanols may provide protection against this decline. COSMOS-Mind is an NIA-funded ancillary study to the Cocoa Supplement and Multivitamin Outcomes Study, a 2x2 factorial randomized controlled trial testing the effects of cocoa flavanols (600 mg/d) and a multivitamin with matching placebo on cardiovascular disease and cancer endpoints. The primary aim of COSMOS-MIND is to assess the effects of COSMOS supplements on cognitive trajectory over 3 years of follow-up using a composite measure of cognitive function. An important secondary outcome is incident Mild Cognitive Impairment or Alzheimer's disease and other dementias. A validated telephone-based protocol conducted at baseline and annually thereafter assesses attention, memory, executive function, language, and global cognitive functioning in 2,000 women and men aged 65 years and older without insulin-dependent diabetes. Additional measures administered by telephone assess subjective memory concerns, depression, and insomnia. For participants who score below a pre-specified threshold on a test of global cognition, a study partner will be interviewed to obtain additional information regarding cognitive and functional status. A web-based telephone call tracking system is used to prioritize COSMOS-Mind enrollment to ensure diverse representation. Real-time reports monitoring study calls provide information to detect issues that may impact recruitment goals, data collection, personnel resources, and costs. At the end of follow-up, cognitive status will be adjudicated by an expert panel to identify Mild Cognitive Impairment, and Alzheimer's and related dementias. Enrollment will be completed in the summer of 2017. COSMOS-Mind will establish whether daily use of a cocoa flavanol supplement, with or without a multivitamin, can protect cognitive function and reduce incidence of cognitive impairment, including Alzheimer's dementia.

### P399

#### Development of a safe haven analytical platform

Sharon Kean[1], Alan Stevenson[1], Allen Tervit[1], Marion Flood[2]
[1]University of Glasgow; [2]Greater Glasgow & Clyde Health Board
**Correspondence:** Sharon Kean
*Trials* 2017, **18(Suppl 1):**P399

**Background**
Using routinely collected data for clinical trials is on the increase and funders are recognising that this is an excellent method to get answers to many research questions. In order to utilise non-consented electronic records such as hospitalisation admission and discharge records, deaths and prescribing data requires appropriate governance and a secure IT environment which fulfils the requirements of the data providers.
**Method**
The Glasgow Safe Haven is a collaboration between University of Glasgow (UOG) and Greater Glasgow & Clyde Health Board. Approval is sought from a local privacy advisory committee for access to non-consented data on a per research project basis. Once approved, the data are extracted, anonymised and placed on an analytical platform hosted by UOG where researchers can then perform their analysis in a strictly monitored environment. In order to become a "safe haven" analytical platform, certain requirements need to be adhered to. Security is paramount and for very sensitive datasets the creation and use of a "safe room" is required.
**Conclusion**
The technical infrastructure to comply with data providers requirements will be presented. Governance procedures and oversight will be discussed. Exemplar project to demonstrate the value of using this method for research will be demonstrated.

### P400

#### The UKCRC information systems operational group: what is it we do?

Sharon Kean[1], Will Crocombe[2], Ian Kennedy[3], Danny Kirby[4], Duncan Appelbe[5], Carolyn McNamara[6], Tim Chater[7]
[1]Glasgow Clinical Trials Unit; [2]Leeds CTU, University of Leeds; [3]Diabetes Trials Unit Oxford, University of Oxford; [4]Leicester CTU, University of Leicester; [5]Liverpool CTU, University of Liverpool; [6]Institute of Cancer Research Clinical trials and Statistics unit; [7]Sheffield CTRU, University of Sheffield
**Correspondence:** Sharon Kean
*Trials* 2017, **18(Suppl 1):**P400

**Background**
The UK Clinical Research Collaboration (UKCRC) partner's goal is to establish the UK as a world leader in clinical research. The UKCRC provides a forum that enables all partners to work together to transform the clinical research environment within the UK.
**Methods**
The Information Systems Operational Group has a mandate to foster collaboration and improve quality through self-support (within the group) to help ensure robust secure and regulatory compliant provision of IS systems to registered CTUs. The objectives and support themes undertaken by the group evolve over time to take into account changes in the regulatory environment and technological methodology are directed by the UK group (consisting of representatives from all registered CTUs) as a whole following national meetings or feedback from working groups. The activities of the ISOG are overseen and directed by a steering group consisting of members proposed by the directors of the registered CTU's and mandated by the UKCRC executive.
**Conclusion**
The purpose, activities and outputs of the ISOG in collaboration with other UKCRC operational groups will be presented.

### P401

#### Increasing the availability of statistical tools through mobile development

Chris Cook[1], Taylor Phillips[1], Michael LeBlanc[2]
[1]Cancer Research And Biostatistics; [2]Fred Hutchinson Cancer Research Center
**Correspondence:** Chris Cook
*Trials* 2017, **18(Suppl 1):**P401

SWOG has a free set of online statistical tools that are used widely across the world at stattools.crab.org. We were interested in further expanding the reach of these tools by making them 'mobile friendly'. We wanted users to have a similar user experience on smartphones and tablets as they would on a normal desktop computer. This would give users an opportunity to use statistical tools wherever they went. SWOG wanted to also give training sessions where we can demonstrate statistical tools and then have attendees follow along on their mobile device to provide an active learning experience.
In July 2016, SWOG upgraded its statistical tools site to be 'mobile friendly' or responsive. The site is now able to dynamically change its interface to accommodate the different devices, screen sizes, and browsers of the different users that access it. The current site supports all mobile platforms including iphones, ipads, Android, blackberry, and Microsoft devices. This presented a technical challenge due to the sheer variety of devices and interfaces on the market.
To ensure a successful implementation of the site, SWOG added website analytics to obtain more metrics on what devices are using the site and how they're using it. There were several questions we wanted to answer: What devices are most commonly used on the website and were they able to use the site successfully? Did allowing mobile access give opportunities for new users? Are mobile devices utilized differently by users in different countries? We plan to gather this data to help answer these questions over the coming months and use them to improve the site.
Support: NIH/NCI/NCTN grants CA180819, CA180888.

### P402

#### Architecture design of an automated study drug distribution coordination module integrated in a web-based clinical trial management system

Wenle Zhao
Medical University of South Carolina
*Trials* 2017, **18(Suppl 1):**P402

High quality study drug distribution management is essential to the operation efficiency and quality of large multicenter clinical trials. First, each site must have a sufficient drug inventory, i.e. At least one drug kit for each treatment arm, in order to perform subject randomization. When response adaptive randomization is applied, the treatment allocation ratio may change multiple times during the study period, and corresponding site drug inventory adjustment may be needed. Second, the high cost and short shelf life of study drug and the slow subject recruitment speed may demand to minimize the site drug inventory. Finally, study drug distribution management must take into account the protection of treatment allocation concealment and treatment blinding, the time and financial cost of study drug shipping. To accomplish these critical tasks, traditional manual drug distribution coordination, often managed using spread sheets, is not reliable. As the national data management center for several NIH-funded clinical trial networks, we have developed a generic study drug tracking module integrated in the web-based clinical trial management system. It provides automated coordination of study drug distribution for multicenter clinical trials. Study drug requests are automatically generated when the site drug inventory is less than the pre-specified threshold and are triggered by a new subject randomization, confirmation of drug removal from site inventory for any reason. The entire study drug shipping process, from the central pharmacy to the clinical sites, including optional multiple regional drug depots, is tracked and information is shared among collaborators in real time. Central and local pharmacy staff are notified by automated emails when study drug distribution actions are requested. This module has been implemented in 8 multicenter trials, and received very positive feedback from investigators. This presentation will discuss the database design and implementation of the automated study drug distribution coordination module in the clinical trial management system.

### P403

#### Automated solution for importing lab test results from a laboratory information management system into an electronic data capture system

Elizabeth Hill, Nuria Porta, Miguel Miranda, Marie Hyslop, Penelope Flohr, Rebecca Lewis, Edward Heath, Claire Snowdon, Johann de Bono, Emma Hall
[1]The Institute of Cancer Research
**Correspondence:** Elizabeth Hill
*Trials* 2017, **18(Suppl 1):**P403

**Background**
CTC-STOP is a multicentre phase III trial for castration resistant prostate cancer patients with bone metastases (MCRPC). The trial is designed to determine if the use of serial circulating tumour cells (CTC) counts can direct early discontinuation of docetaxel chemotherapy in mcrpc patients without adversely impacting overall survival, when compared with standard approaches to guide treatment switch decisions. Treatment switch recommendations based on CTC progression are centrally managed by the co-ordinating clinical trials unit (ICR-CTSU) and require real time transfer of CTC counts from the central laboratory to the ICR-CTSU.
**Challenges**
CTC-STOP blood samples are received and processed by the central laboratory. CTC counts are recorded in the central laboratory's information management system (LIMS). The LIMS cannot be programmed to calculate or alert staff to CTC progressions and the ICR-CTSU cannot access LIMS. CTC counts are required by the ICR-CTSU trial team for CTC progression calculations within the Electronic Data Capture (EDC) system, so treatment switch recommendations can be issued promptly. Manual data re-entry of the CTC counts into an EDC database would be time consuming and could result in transcription errors; therefore a system was designed to automatically import the CTC counts from the LIMS into the EDC system.
**Solution**
An in-house C#.net application was developed by ICR-CTSU to verify and import CTC counts provided by the central laboratory LIMS. The application locates and verifies the LIMS export file before validating the subject identifiers and trial visit timepoint for each CTC count. Once validated, the data is imported into the EDC system using its Application Programming Interface. The application runs automatically every day via Windows Task Scheduler and generates a log file for each export file to detail any errors found during processing and the number of data rows successfully imported into the EDC system. When the application has completed processing the log file is emailed to the ICR-CTSU trial team for review. Details of each export file processed are also written to a database history table.
The CTC counts imported into the EDC system are used to perform computations to ascertain whether a treatment switch is required by calculating whether each subject has progressed or responded. The EDC system's event management tool automatically alerts the ICR-CTSU trial team if the imported results reveal an initial progression event requiring a second confirmatory CTC count, a confirmation of progression requiring a treatment switch recommendation or a continuation of therapy confirmation for any participant.
**Conclusion**
The application provides a robust, time critical, automatic solution to accurately import LIMS data into the EDC system, reducing workload for both the ICR-CTSU team and laboratory staff and removing error rates associated with manual data entry. The application also provides the ICR-CTSU trial team and site staff, including treating clinicians, with real-time access to the CTC counts.

### P404

#### Collating the evidence base to facilitate patient and public involvement in core outcome set development - A qualitative meta-synthesis

Lucy Brading[1], Kerry Woolfall[2], Paula Williamson[3], Bridget Young[2]
[1]University of Liverpool; [2]University of Liverpool, Institute of Psychology Health and Society/North West Hub for Trials Methodology Research; [3]University of Liverpool, Department of Biostatistics/North West Hub for Trials Methodology Research
**Correspondence:** Lucy Brading
*Trials* 2017, **18(Suppl 1):**P404

**Background**
Patient and public involvement (PPI) in health research has grown rapidly and is considered a key component of good research. Patient and public input into the development of core outcome sets (COS), which specify which outcomes should be measured and reported as a minimum in trials within specific health areas, is also increasing. It is vital to the credibility of a core outcome set that the chosen outcomes are relevant to both health professionals and patients and it is believed that this can be assisted through PPI in the development process.
Whilst research has investigated PPI in clinical trials and service improvement, PPI in the development of COS is yet to be explored; despite some distinct challenges and the need for guidance on how best to involve patients in COS development. As part of a wider project to inform methods to facilitate PPI in COS development, we are conducted a review and qualitative meta-synthesis of published COS studies that have reported on PPI. Meta-synthesis involves the integration of themes from numerous qualitative studies; the technique is interpretive and yields findings that are 'greater than the sum of the parts'.
As few published COS studies have reported on PPI, the scope of the review was widened to also include PPI in the development of patient-reported outcome measures (proms). It was anticipated that the challenges encountered in the development of both COS and proms would be analogous.
**Objective**
To identify studies which have involved patients and members of the public (as research partners, co-investigators, advisors or research team members), to develop COS or proms and describe the ways in which PPI has been conceptualised and reported in these studies, the methods of PPI used, and the contexts in which PPI has been employed.
**Method**
The review is currently underway; we are searching the COMET Initiative's database of all completed work in selecting COS to identify studies with PPI. We developed a search strategy to identify PROM development projects with PPI, via searches of MEDLINE, psycinfo, hapi and the Cochrane Methodology Register.
**Conclusion**
Previous research has highlighted the need for guidance on how best to seek the input of patients and the public in developing COS. As part of a wider project investigating PPI in COS development, the findings from this review will inform the development of guidelines for the COS development community on methods for involving patients as research partners, co-investigators, advisors or research team members. These guidelines will facilitate improved engagement with patients - one of the key stakeholders in COS development.

### P405

#### From molecule to medicine: a case study in how a statistician can provide strategic input to drug development

Nelson Kinnersley
Roche Products Ltd
*Trials* 2017, **18(Suppl 1):**P405

When evolving the design of a clinical trial, statisticians may spend a considerable amount of time to optimise certain characteristics of the proposed trial and there is considerable literature to support such work. However, the literature is more sparse on the strategic factors that a statistician should consider when involved with designing an entire drug development programme. The aim of this work is to use a suitably anonymised case study to inform the practising statistician about the considerations for strategic drug development. Through liberal use of scenarios covering topics such as Target Product Profile (TPP), Clinical Development Plans (CDP), gating criteria and probability of success we will offer suggestions for how a statistician can contribute to strategic drug development. Many concepts are applicable across a variety of therapeutic areas even if the technical implementations may differ. It is hoped that with a wider understanding of strategic drug development, more statisticians can be better equipped to contribute to the cross-functional teams who perform this type of work when the plans are being developed for how to turn a molecule into a medicine.

### P406

#### Challenges in teaching clinical trials: the experience of teaching biostatistics in online post-graduate academic courses, target to industrial biometrician

Laura Cavaliere[1], Egle Perissinotto[1], Ileana Baldi[1], Beatrice Barbetta[2], Dario Gregori[1]
[1]University of Padova; [2]Rottapharm Biotech
Correspondence: Laura Cavaliere
*Trials* 2017, **18(Suppl 1):**P406

**Background**

At today, new technologies and widespread web access are moving the traditional face-to-face teaching towards online and open digital teaching processes. New technologies provide widespread flexible and convenient learning tools overcoming any temporal and geographical constrains. The instances of innovation gradually enhanced higher education institutions towards new models of e-learning. The challenge is furtherly hard for teaching Biostatistics to health professionals and medical students, who are in general very little motivated to learn statistics in the traditional academic courses.

**Material and methods**

In the academic year 2015/2016, first in the teaching experience at the University of Padova, two fully online post-graduate courses of Biostatistics were established: the first one taught basic biostatistics and research methodology; the second one, advanced topics in biostatistics. The courses were organized into two phases: modules dealing with structured topics (25 weeks, each unit from 2 to 5 weeks) and a project-work (20 weeks). To perform statistical analysis, the R software was adopted. The student-centered model was used instead of the traditional teacher-centered model. Each student independently manages her/his access to the web pages contents, without limits in number of accesses, within the timetable. She/he attends the didactic activities individually, preforming self-test and participating in the discussion online. From the teaching point of view, the face-to-face lessons components of contents, interaction and assessment were translated into digital and online contents and tools; from the logistic point of view, the publication time of the work tools was planned, the quality and accessibility of platform was conveniently tested and the students' Access to the platform was monitored. The computerized portal for education was based on MOODLE platform. To realize the fully online courses, different tools were built: streaming videos (10–40 minutes) with in person teachers' explanation, slides highlighting central concepts, self-tests with multiple-choice questions and simulation-based tests with unlimited access, and homework with supervisor. Moodle platform allowed students for documents download and upload. At the end of each module a questionnaire regarding the assessment of teaching has been reported, the questions were proposed in a Likert scale from 1 to 10.

**Results**

Twelve students in the basic course and thirteen in the advanced course were able to access to Moodle platform. Most of the students were workers. Median number of individual accesses to Moodle platform was 458 for the basic course and 931 for the advanced one. They mostly appreciated: self-administration of hour, number and time of access and stimulating discussion board online. They reported as worst limits: inadequate preliminary knowledge to understand new biostatistics concepts (median score 6.3 for the basic course, 6.6 for the advanced one), too elevated study load in the advanced course (median score 6.7), time-spending searching concepts in videos during study and reviewing topics due to streaming modality.

**Conclusion**

Our experience supports feasibility and efficacy of online distance learning in teaching biostatistics. The experience suggests elaborating the following tools: videos length shorter than 20 min, lists of main concept and definition indicating the position (minute) in videos, widespread operative examples, timely matching of concepts and examples.

### P407

#### Clinical trials in neonatology: design and analyses issues

Abhik Das[1], Jon Tyson[2], Claudia Pedroza[2], Barbara Schmidt[3], Marie Gantz[1], Dennis Wallace[1], William Truog[4], Rosemary Higgins[5]
[1]RTI International; [2]University of Texas Health Sciences Center; [3]University of Pennsylvania; [4]Children's Mercy Hospital; [5]NICHD, NIH
**Correspondence:** Abhik Das
*Trials* 2017, **18(Suppl 1):**P407

Impressive advances in neonatology have occurred over the 30 years of life of The Eunice Kennedy Shriver National Institute of Child Health and Human Development Neonatal Research Network (NRN). However, substantial room for improvement remains in investigating and further developing the evidence base for improving outcomes among the extremely premature. We discuss some of the specific methodological challenges in the statistical design and analysis of randomized trials in this population. Challenges faced by the NRN, applicable to all neonatal trials, include designing trials for unusual or rare outcomes, accounting for and explaining center variations, identifying other subgroup differences, and balancing safety and efficacy concerns between short-term hospital outcomes and longer term neurodevelopmental outcomes. The constellation of unique patient characteristics in neonates calls for broad understanding and careful consideration of the issues identified in this presentation for conducting rigorous randomized trials in this population.

### P408

#### Independent adjudication of neonatal cranial ultrasound scans in a pilot randomised trial

Lucy Bradshaw[1], Jon Dorling[2], Lelia Duley[1], Lindsay Armstrong-Buisseret[1], Joe Fawke[3], Bernard Schoonakker[4], Eleanor Mitchell[1], Rob Dineen[5]
[1]Nottingham Clinical Trials Unit, University of Nottingham; [2]Early Life Research Group, University of Nottingham; [3]Leicester Neonatal Service, University Hosptials Leicester NHS Trust; [4]Nottingham Neonatal Service, Nottingham University Hospitals NHS Trust; [5]Division of Clinical Neuroscience, University of Nottingham
*Trials* 2017, **18(Suppl 1):**P408

This abstract is not included here as it has already been published.

### P409

#### Time well spent? A comparison of the work associated with collecting primary and secondary outcomes

David Pickles[1], Shaun Treweek[2]
[1]Leeds Teaching Hospitals NHS Trust; [2]University of Aberdeen, Health Services Research Unit
**Correspondence:** David Pickles
*Trials* 2017, **18(Suppl 1):**P409

**Background**

Trials are essential but often inefficient. Some of this inefficiency is due to designs that burden both trial participants and trial teams with measurements that are not essential to answer the trial's main research questions. Trialists are generally good at selecting their primary outcomes - the outcomes they consider most important. Trialists have less focus when it comes to secondary outcomes.

**Method**

A random selection of 115 protocols for publicly funded, randomised trials published 2010–2014 were selected (roughly 24 per year) for analysis. To date, twenty trials have been examined. The primary and secondary outcomes were extracted from protocols. Data on time to complete each outcome were sought from protocols; where timing was not available, these data were requested from the corresponding author, or from trial managers familiar with the outcome. To date, twenty trials have been examined.

**Results**

Trialists spend much more time on secondary outcomes than primaries. This is not surprising; there are more secondary outcomes. What is more surprising is how much more: some trials spend more than 20 times as much time collecting secondary outcome data as primary outcome data. As an example, one trial spent 66 hours collecting primary outcome data and 1466 hours on secondaries. Using UK costing data, this is approximately £2908 on primary data collection and £63990 on secondaries. Trials that spend less than 10% of data collection effort on primary outcomes seem common. The median ratio of time to obtain primary to secondary outcomes is 1:8.

**Conclusions**

Trialists routinely spend a far greater proportion of their time obtaining outcomes that they themselves deem of lesser importance than they do on primary outcomes. Given the significant expense of collecting data and the widely reported fact that much trial data goes unreported, we suggest that trialists should have an increased awareness of the burden and cost associated with each outcome when making their selections.

This work is part of the Trial Forge initiative to improve trial efficiency.

### P410

#### Maximising research impact: a systematic review of research impact frameworks

Samantha Cruz Rivera, Derek G. Kyte, Olalekan Aiyegbusi, Thomas J. Keeley, Melanie Calvert
Centre for Patient Reported Outcomes Research, University of Birmingham
**Correspondence:** Samantha Cruz Rivera
*Trials* 2017, **18(Suppl 1):**P410

**Background**

Increasingly, researchers are being asked to demonstrate the impact of their research to their sponsors, funders and fellow academics. However, the most appropriate way of measuring the impact of healthcare research is subject to debate. We aimed to identify the existing frameworks used to measure healthcare research impact and to summarise the common themes and metrics in an impact matrix.

**Methods**

Two independent investigators systematically searched, MEDLINE, EMBASE, CINAHL+, the Health Management Information Consortium and the Journal of Research Evaluation from inception until May 2016 for publications that presented an impact framework. We then summarised the common concepts and themes across frameworks and identified the metrics used to evaluate differing forms of impact.

**Results**

Twenty-four unique frameworks were identified, addressing five broad categories of impact: (1) 'Primary research-related impact', (2) 'Influence on policy-making', (3) 'Health and health systems impact', (4) 'Health-related and societal impact', and (5) 'Broader economic impact'. These categories were subdivided into 16 common impact subgroups. Authors of the included publications proposed 80 different metrics aimed at measuring impact in these areas.

**Conclusions**

The measurement of research impact is an essential exercise to help direct the allocation of limited research resources, to maximise benefit and help minimise research waste. This review provides a collective summary of existing impact frameworks, which funders may use to inform the measurement of research impact and researchers may use to inform study design decisions aimed at maximising the short, medium and long-term impact of their research.

Keywords: medical research impact, impact metrics, research impact framework

### P411

#### Methods for including patients in core outcome set development

Alice Biggane[1], Lucy Brading[1], Bridget Young[1], Philippe Ravaud[2], Paula R. Williamson[1]
[1]University of Liverpool; [2]Universite Paris Descartes
**Correspondence:** Alice Biggane
*Trials* 2017, **18(Suppl 1):**P411

**Background**

The usefulness and importance of a core outcome set (COS) is well recognised, as is the need for patient participation in its development. A COS needs patient input to ensure it is credible and that future studies using the COS can provide patients and clinicians with relevant knowledge regarding interventions, consequently reducing the amount of wasteful research. Researchers are increasingly aware of this and are progressively including patients and the public alongside other stakeholders in identifying what outcomes to measure in clinical trials. Whilst only 22% of 300 published COS reported that there was input from patients in their development, nearly 90% of 146 ongoing studies report including patients in some capacity. However, nobody knows the best methods for facilitating the participation of patients in COS development. There are numerous challenges in enabling patient

Participation in a COS study and these will depend on the patient group and the methods chosen. Therefore, this project aims to investigate and develop methods for including patients as participants in COS development in a meaningful and productive manner.

**Objectives**

To investigate which methods are being used by COS developers to facilitate patient participation in ongoing studies and the rationale behind using that method.

**Methods**

The COMET database currently has 146 ongoing registered studies, of which 124 aim to include patient and public representatives. We will identify the COS developer leads for these studies and invite them to participate in a short online survey. The survey will establish the capacity in which patients are being included (involvement vs. Participation), developers' methods for enabling patient inclusion, and their rationale for choosing a particular method(s). This survey will be conducted in English.

**Expected results**

The results will provide insights into the COS developers' Plans and rationale for facilitating patient participants in their studies. Details about methods used for recruitment (social media, NHS services etc.) And methods used for eliciting the outcomes will be obtained (qualitative interviews, Delphi survey etc.). We will also establish the reasoning for using these methods. We will then use this information to purposively sample COS developers and patients to participate in a subsequent qualitative interview phase of our study.

### P412

#### Designing trials that aim to evaluate therapies that target brain metastases in cancer patients: challenges and recommendations

Sujata Patil
Memorial Sloan Kettering Cancer Center
*Trials* 2017, **18(Suppl 1):**P412

**Objective**

To provide a comprehensive review of the methodological challenges in designing trials where progression in the brain is the primary endpoint and to provide concrete clinical design recommendations.

**Background**

The presence of brain metastases in cancer patients often indicates poor prognosis. Additionally, the presence of brain metastases can directly impact a patient's quality of life. Controlling brain disease is important and has been one current focus of clinical trials and retrospective reviews [Preusser et al., Eur J Cancer 2012; Lin, ecancer 2013]. However, there are challenges in conducting such studies and interpretations of results are not uniform. For instance, patients may progress extracrainially before progression in the brain can be assessed, thereby creating a competing risks analytic setting. Assessing true brain recurrence versus radionecrosis and the use of consistent criteria to assess brain recurrence have also been methodological issues.

**Methods**

Simulations modifying the following factors 1) sample size, 2) censoring, 3) effect size, 4) correlation between competing events, 5) degree of endpoint misclassification (pseudo-progression), and 6) method used for analysis (Kaplan-Meier, Cox regression, cumulative incidence, subdistribution regression) are conducted. The effect of these factors on power and type I error in Phase II clinical trials are reported.

**Results**

Simulations on the randomized phase II design show that per arm sample sizes of 75 to 100 have sufficient power to detect hazard ratios in the range of 1.7 and 2.0 where the endpoint is brain-specific. Higher correlation between competing risks (e.g. Brain vs systemic progression) and the method used for analysis (e.g. Cause-specific hazard or cumulative incidence subdistribution) have effects on sample size. Misclassification of the endpoint (eg pseudo- progression) also has a demonstrable effect on inference. These simulation findings will be described in detail. Results from ongoing simulations under other Phase II designs will also be described along with design recommendations.

### P413

#### A comparison of stroke diagnosis at trial entry by local clinicians versus independent adjudicators: secondary analysis and simulation

Peter Godolphin[1], Trish Hepburn[1], Liz Walker [1], Nikola Sprigg[1], Joanna M. Wardlaw[2], Philip M. Bath[1], Alan A. Montgomery[1]
[1]University of Nottingham; [2]University of Edinburgh
**Correspondence:** Peter Godolphin
*Trials* 2017, **18(Suppl 1):**P413

**Objectives**

The aim of this study was to investigate the benefit of adjudication of stroke type at trial entry in a large stroke trial. The three objectives were to: (1) compare stroke diagnoses made by site clinicians and independent adjudicators; (2) assess the impact of adjudication on the primary analysis and a subgroup analysis by stroke type; (3) using simulation, explore the effects of increasing levels of misclassification on analyses.

**Methods**

The Efficacy of Nitric Oxide in Stroke (ENOS) trial examined the safety and efficacy of glyceryl trinitrate (GTN) versus no GTN in patients with acute ischaemic or haemorrhagic stroke. Independent expert assessors, referred to as adjudicators, who were masked to treatment allocation, centrally assessed cranial scans to inform diagnosis of stroke type. For this study, diagnoses made by local site clinicians are referred to as Hospital diagnosis, whilst diagnoses with input from independent adjudicators are referred to as Trial diagnosis. The Trial diagnosis was the diagnosis used in all ENOS analyses. Agreement between Hospital and Trial diagnoses was determined using unweighted kappa. The trial primary analysis and subgroup analysis by stroke type were re-analysed using Hospital diagnosis as baseline covariate and interaction factor respectively. Statistical simulations were created to: (1) increase misclassification of Hospital compared with Trial diagnosis; (2) introduce an interaction (subgroup effect) between ENOS treatment arm and stroke type.

**Results**

Of 4011 participants randomised, 3857 (96%) had baseline scans that were assessed by adjudicators. There was excellent agreement between Hospital and Trial diagnoses (crude agreement 98%, unweighted kappa, $k = 0.92$). Adjudication of stroke type had no impact on the primary outcome ($p = 0.95$) or subgroup analysis by stroke type. These findings were robust to all except the most extreme simulated non-differential misclassification of stroke diagnosis and subgroup effect.

**Conclusion**

This study found that clinicians at ENOS trial sites largely were correct in their diagnosis of stroke, and adjudication did not impact on the trial results. Diagnostic adjudication may be important if diagnosis is complex and a treatment-diagnosis interaction is expected. Researchers should consider the value adjudication may bring to their study by using pilot or feasibility studies to estimate misclassification and potentially avoid substantial resource implications.

### P414

#### The impact of blinded endpoint review on the incidence of primary short-term outcomes in the SIFT trial

Christopher Partlett[1], Louise Linsell[1], Oliver Hewer[1], Ed Juszczak[1], Jon Dorling[2]
[1]NPEU, University of Oxford; [2]Division of Child Health, Obstetrics and Gynaecology, University of Nottingham
*Trials* 2017, **18(Suppl 1):**P414

**Background**

Blinded endpoint review committees (BERCs) comprise of a panel of clinical experts blinded to trial allocation. They are responsible for reviewing trial outcome data reported by participating centres, to ?A3B2 show $132#?>ensure they meet the protocol-specified criteria. This can be particularly useful for outcomes which are complex to assess, include subjective components, or when the original data collection could not be blinded.

SIFT (ISRCTN: 76463425) is an open-label multicentre randomised controlled trial of a feeding intervention in very preterm or very low birthweight infants in neonatal units in the United Kingdom and Ireland. Infants were randomised to receive either a faster rate of feeding (30 ml/kg/day) or a slower rate of feeding (18 ml/kg/day). BERCs were set up to assess the incidence of two primary short-term outcomes; late onset invasive infection (LOII) and necrotising enterocolitis (NEC).

**Objective**

To ascertain the impact of the BERC review on the reported incidence of LOII and NEC, compared to those derived from the original data collection forms (DCFs).

**Methods**

Pairs of BERC reviewers independently reviewed Gut Signs and Infection dcfs, feeding log data and any additional data requested (e.g. Discharge summaries) and completed a diagnostic classification form. These were cross-validated for discrepancies and referred to a third BERC reviewer if agreement could not be reached.

The incidence of LOII and NEC were calculated for each arm before and after BERC review; firstly, using data obtained from the Gut Signs and Infection dcfs, applying an algorithm detailed in the statistical analysis plan; and secondly, using the diagnostic classifications determined by the BERC.

For both outcomes we compared the risk ratio (fast/slow) and 95% confidence interval derived from the DCF and BERC classification. For each arm we also investigated the concordance between the classification of infants before and after BERC review, using the kappa statistic and mcnemar's test.

#### Results
There was little change in the risk of LOII for either arm, however there was a slight reduction in the risk ratio for NEC after BERC review; (RR 0.89, 95% CI 0.65 to 1.23) compared to (RR 1.00, 95% CI 0.73 to 1.36).
There was strong concordance between the classification of infants before and after BERC review, with over 95% agreement for both outcomes in both arms. Among the discordant cases the original DCFs were more likely to classify an infant as a case than the BERC, however this discordance was only marginally statistically significant for NEC in the fast feeding arm ($p = 0.04$).
### Conclusion
The two methods were highly concordant, however, there was marginal evidence that unblinded local investigators were more likely to assign a diagnosis of NEC in the fast feed arm, in infants deemed not to have NEC by the BERC. This may suggest a potential bias, reflecting concerns about rapid advancement of feeds and its possible effect on the gut. Thus, while the addition of BERC reviews did not alter the conclusions of the trial, this investigation highlights their importance in reinforcing confidence in the outcome results.

### P415
### How are surrogate outcomes defined in critical care trials? Preliminary results of a systematic review
Rejina Verghis[1], Bronagh Blackwood[2], Cliona McDowell[3], Daniel Hadfield[4], Philip Toner[5], Marianne. Fitzgerald[6], Daniel F. Mcauley[6], Mike Clarke[6]
[1]Queens University Belfast QUB; [2]Centre for Experimental Medicine, Queen's University Belfast; [3]Northern Ireland Clinical Trials Unit; [4]Kings College and Hospital; [5]Belfast Health and Social Care Trust; [6]Centre for Public Health, Queen's University Belfast
**Correspondence:** Rejina Verghis
*Trials* 2017, **18(Suppl 1):**P415

#### Background
The choice of outcome measure is a critical decision in the design of any clinical trial, but many phase III clinical trials in critical care fail to detect a difference between the interventions being compared. This may be because the surrogate outcomes used to show beneficial effects in early phase trials (which informed the design of the subsequent phase III trials) are not valid guides to the differences between the interventions for the main outcomes of the phase III trials. We did this review to determine the variability in reported surrogate outcomes in early phase, critical care trials.
### Methods
We undertook a systematic review to generate a list of outcome measures used in early phase critical care trials. We searched for trials published in the six top-ranked critical care journals between 2010 and 2015. The review was conducted according to the protocol published on the PROSPERO website (http://www.crd.york.ac.uk/PROSPERO/display_record.asp?ID=CRD42015017607). We searched MEDLINE and EMBASE using key words such as intensive care unit, critical care and randomised controlled trials. Two independent reviewers were involved in the search and article screening. All articles meeting inclusion criteria and published in 2010 were selected for data extraction and data saturation was achieved during this process. Therefore, we included only an additional 10% of the articles from 2014 and 2015 to boost the sample with some more recent papers. We extracted descriptive data including trial registration details, outcome measures reported in the methods, definition, and time-points. We classified outcomes into body organ systems, severity of disease and quality of life with sub-categories based on clinical judgement, and tabulated them to understand underlying patterns and variations.
### Results
A total of 5448 references were screened. The total number of included articles was 48, and based on the preliminary analysis, these mentioned over 300 outcomes in their methods sections. Focusing specifically on outcomes reported in the respiratory category, there were ten sub-categories and the number of different outcomes in the subcategories. The reported outcome measures were analysed and reported in a variety of ways. The definition of specific measurement (mechanical ventilation), participant level analysis metric (duration of mechanical ventilation or time to extubation), method of aggregation (mean & SD or median & IQR) and time points vary across trials.
### Conclusions
There is large variability in outcome reporting in early phase, critical care trials. This creates difficulties for synthesizing data in systematic reviews and planning definitive trials. This review highlights an urgent need for standardization and validation of surrogate outcomes reported in critical care trials. Future work will validate and develop a core outcome set for surrogate outcomes in critical care trials.

### P416
### Splintered adverse event reporting in multicenter clinical trials
Joy Black, Valerie LW. Stevenson, Robert Silbergleit
University of Michigan
**Correspondence:** Joy Black
*Trials* 2017, **18(Suppl 1):**P416

#### Objective
The use of controlled vocabularies like meddra are essential to practical useful adverse event (AE) reporting, but have limitations. The use of autocoding in meddra allows objective mapping of verbatim terms (VT) to preferred terms (PT) but can result in the listing of clinically identical events into a variety of effectively synonymous PT, an effect we call splintering. A potential solution involves a clinician grouping these splintered PT into a single collapsed PT relevant to the medical context of the trial. Both splintering and collapsing AE have the potential to obscure safety signals.
### Methods
We reviewed all AE reported in two clinical trials performed in our clinical trials network, protect (NCT00822900) and ATACH (NCT01176565) both of which were reported in the New England Journal of Medicine. For each trial, a splintered and collapsed list of PT were compared. Protect published the collapsed list and ATACH the splintered list. Splintered lists were generated primarily by autocoding, with manual coding by a data manager during the conduct of the trial when autocoding failed. Collapsed lists were generated from the splintered lists using clinical judgement by the trial investigators in protect at the end of the trial. For ATACH, the collapsed list was generated in part by investigators at the end of the trial and in part by the authors of this abstract. All splintered and collapsed lists used only meddra PT. Descriptive statistics were used to characterize and compare splintered and collapsed AE lists.
### Results
Substantial splintering was found in both trials. 3032 AE occurring in 810 patients in protect were coded under 399 unique PT in the splintered list, and under 235 unique PT in the collapsed list. Similarly, in 1000 subjects enrolled in ATACH, 3140 AE were coded under 344 unique PT in the splintered list and 193 unique PT in the collapsed list. There were 235 and 193 collapsed PT terms in protect and ATACH respectively and collapsed terms included a mean of 3.00 splintered terms with a range of 2 to 9 PT. Illustrative examples of splintered and collapsed terms in these two trials include: bronchopneumonia, lung infection, pneumonia aspiration, and 7 other PT that collapsed under the PT 'pneumonia'; and embolic stroke, cerebral artery embolism, cerebral infarction and 5 other PT clinically equivalent to the collapsed PT 'ischemic stroke'. An example of the potential effect of splintering was found in ATACH where splintered terms related to renal injury were similar between the two treatment groups as individually reported, but demonstrated a potentially significant difference when collapsed.
### Conclusions
We have demonstrated that autocoding AE VT to PT in meddra is objective but results in significant splintering as compared to clinically relevant collapsed terms, obscuring medically important safety effects. Use of clinical judgement to combine effectively synonymous PT is subjective, but is a practical solution.

### P417

#### What does qualitative case study methodology have to offer the interpretation of findings from trials of complex interventions? Reflections from a large complex intervention study

Caarol Bugge[1], Aileen Grant[1], Sarah Dean[2], Jean Hay Smith[3], Doreen McClurg[4], Suzanne Hagen[4]
[1]University of Stilring; [2]University of Exeter; [3]University of Otago; [4]NMAHP RU, Glasgow Caledonian University
**Correspondence:** Caarol Bugge
*Trials* 2017, **18(Suppl 1):**P417

The contribution qualitative research can make to improving intervention and trial design, evaluation and implementation is well recognised (O'Cathain et al., 2014; Moore et al. 2011). Qualitative methods are often used alongside quantitative methods within a process evaluation to explore trial processes, intervention components and mechanisms in relation to context (Grant et al. 2013). This paper describes the use, and presents the advantages, of a two-tailed qualitative case study methodological design linked to a trial of a complex intervention for women with urinary incontinence (UI) (OPAL ISRCTN 57746448).

OPAL (optimising pelvic floor exercises to achieve long-term benefits) has three elements: a large multi-centre trial, a mixed methods process evaluation, and a longitudinal qualitative case study. The trial investigates the effectiveness of biofeedback intensified pelvic floor muscle training (PFMT) versus PFMT alone in improving UI symptoms for women with stress or mixed UI. The case study is a two-tailed design, one tail is the control (PFMT) the other the intervention (intensified PFMT), exploring the views and experiences of trial women about UI and the interventions to identify barriers and facilitators to intervention delivery and adherence and to inform potential roll-out of the intervention.

Case study methodology is advocated for exploring real life phenomena within a contemporary context. The nature of the design lends itself to addressing questions that aim to understand, in detail, how or why events occur. The two-tailed design, offers a comparative focus; in the case of clinical trials the tails can be the control and intervention arms enabling comparison of relevant features of the control and intervention. OPAL uses a two-tailed longitudinal case study, where women are interviewed at four time points (baseline, post-treatment, 12 months and 24 months post-randomisation); mirroring the trial data collection. The nature of the analysis encourages a move beyond description to explanation; for example, identifying factors that may interact to influence participant outcomes, and the mechanisms of action.

In the OPAL qualitative longitudinal study the aim is to understand the links between context, delivery, and outcomes in each arm for women with UI. In a complex intervention such as that evaluated in OPAL, many factors could impact on the final outcome for a woman; only some of these factors may relate to intervention delivery. For example, women are asked to exercise at home in both trial arms and in the intensified arm women are asked to use biofeedback to support PFMT at home. There may be many psychological, social, or practical variables that influence a woman's ability to use biofeedback, or do this in the home environment. The case study design aims to support the identification of these factors and, importantly, how their influence may differ on the trial primary outcome (UI at two years) between the control and intervention arms.

In this paper we will explore: 1. The nature of case study methodology. 2. Why case study methodology might be useful for qualitative studies linked to complex intervention trials. 3. Lessons for researchers from our use of case study methodology within OPAL.

### P418

#### Physiotherapists' views of the acceptability and feasibility of the self-management of osteoarthritis and low back pain through activity and skills (SOLAS) complex intervention within a cluster randomised controlled feasibility trial [ISRCTN 49875385]

Deirdre Hurley, David Hayes, Danielle McArdle, James Matthews, Suzanne Guerin
University College Dublin
**Correspondence:** Deirdre Hurley
*Trials* 2017, **18(Suppl 1):**P418

##### Background

Self-management (SM) is endorsed by clinical guidelines for osteoarthritis (OA) and chronic low back pain (CLBP), but there is a current lack of multi-joint interventions to target both conditions in group settings. The 6 week group-based self-determination theory (SDT) driven education and exercise SOLAS intervention was developed in consultation with primary care physiotherapists through the intervention mapping process. Following Medical Research Council (MRC) guidelines for complex interventions, the SOLAS cluster randomised controlled feasibility trial aims to assess the (1) acceptability and feasibility of the SOLAS intervention to patients and physiotherapists compared to usual individual physiotherapy, (2) feasibility of trial procedures and sample size for a definitive trial and (3) effect on secondary outcomes. The aim of the present study was to explore physiotherapists' views of the SOLAS intervention's acceptability and feasibility.

##### Methods

Individual semi-structured telephone interviews were conducted by an independent researcher with 10 physiotherapists (PTs) within one week of their completion of delivery of the SOLAS intervention in the feasibility trial. The interviews were audio-recorded, transcribed verbatim and analysed using inductive thematic analysis, based on Braun and Clarke's method. Coding frames were developed, re-examined and refined. The reliability of the identified themes was established by a second researcher who independently coded a random sample of 25% of the data using the coding frame, with 70% agreement taken as the minimum cut-off rate of agreement.

##### Results

Twenty-six themes were identified that related to six topics; i.e. 1) overall views of the intervention; 2) experience of implementing the intervention; 3) changes made/suggested to the intervention; 4) views on participants' experience of the intervention; 5) perception of the intervention's feasibility for future delivery; and 6) views and experiences of training, and were mapped to the feasibility criteria: acceptability, demand, implementation, practicality, adaptation and integration.

PTs were positive about their experience of training and implementing the SOLAS intervention and its support materials to a mixed group of participants, reporting it acceptable and feasible to deliver. Key demands in delivering the intervention that impacted on implementation included the high volume of education content during class one, which required shortening the exercise session, and the challenge of using the needs supportive communication skills during goal setting and the group exercise component, highlighting the need for additional training. However, PTs felt that overall they implemented the intervention content and structure according to the protocol. Some PTs reported adapting the education component to include additional information based on their clinical expertise and participant needs. Practical considerations for future integration of the intervention into health services included attaining a minimum of six participants to run a successful group, the accessibility of the intervention in some primary care settings, and the need to address health literacy for some participants.

##### Conclusions

The SOLAS intervention content and support materials were considered acceptable and feasible to deliver within the trial and in future healthcare services provided sufficient numbers of clients could be enrolled and retained. Further training in the intervention SDT-based needs supportive communication skills is being developed through E-learning.

### P419

#### Image modification journaling for reproducibility and fraud prevention

Paul Thompson
Sanford Research
*Trials* 2017, **18(Suppl 1):**P419

##### Background

In scientific discussions, images are analyzed to make scientific points. In basic biology, in clinical trials, in clinical research, images are used to present information, show differences between conditions, and define phenomena. The presentation and manipulation of images is governed by rules which have been defined by editors of scientific publications. The images can be cropped (to select parts of a larger image). The contrast of the image can be increased so long as the changes are made over the entire image. The colors of the image can be adjusted over the entire image. Images can be places together, so long as it is clear what is done to the final image and that the component portions are not part of the original image (this can be done by lines of a black color). In recent years, the amount of fraudulent manipulation in images is becoming alarming. Fraud takes many forms, including re-using the same image for different purposes, adding new components to an existing image without clear markings and so forth. Image fraud is frequently discussed on the Retraction Watch blog.

**Results**

Several new approaches are presented here for partial solutions to these problems. When images are manipulated in an interactive tool, a record of the actions can be kept. This is called a "journal". This is a common part of a number of statistical analysis programs (JMP keeps a journal of analysis steps, which can then be used to analyze the data). GIMP has been modified to journal the process of manipulating the image to allow the process to be repeated and to allow inspection of the process. A shiny app (in R) has also been created to perform analysis and journal that analysis using imagemagick code. By making the process of image analysis transparent, fraud in the image manipulation will be reduced.

**Presentation**

Several examples of image manipulation will be presented to demonstrate this new tool and capability. Other image manipulation programs will be discussed to determine if the capability can be extended to them.

### P420

#### Perceptions and experiences of participant recruitment: a qualitative interview study with trial stakeholders

Heidi Gardner, Katie Gillies, Shaun Treweek
University of Aberdeen
**Correspondence:** Heidi Gardner
*Trials* 2017, **18(Suppl 1):**P420

**Background**

Recruitment to randomised controlled trials can be extremely difficult, and poor recruitment can lead to extensions to both time and budget, potentially resulting in an underpowered study which does not satisfactorily answer the original research question. In the worst cases, a trial may be abandoned, causing huge waste. Consequently, recruitment is considered the number one problem in trial methods research.

**Objectives**

To understand how the process of participant recruitment impacts the day-to-day lives of those charged with the task, we conducted a qualitative semi-structured interview study with a wide range of trial stakeholders. This study will help trial methodologists to understand the challenges that trial recruitment generates on the ground, which will enable them to better design future research work so that its results are more relevant and applicable to the challenges faced by those tasked with recruiting individuals to trials.

**Methods**

A mix of purposive and convenience sampling generated trial stakeholders that represented views of those that work within the National Health Service, academia and industry. Individuals categorised themselves as "designers"; those directly responsible for the design or recruitment methods, or "recruiters"; those who implement recruitment strategies to recruit participants to trials. Currently we have developed an interview topic guide that will allow us to investigate and explore the views of trial designers and recruiters. We will also explore how best to present research evidence about recruitment methods so that evidence-based recruitment strategies are effectively disseminated and implemented.

**Results**

Role type and recruitment experiences were varied, spanned various therapeutic indications, intervention types and trials units. Our sample was mainly UK based but does have some representation from further afield. Interviews are scheduled for fall 2016. We have approached 27 individuals in roles such as Research Nurse, Head of Patient Engagement, Clinical Trial Educator, Senior Research Manager and Professor of Health Services. Everyone we approached agreed to take part, giving our sample a split of 14 "recruiters" And 13 "designers", 23 of which are UK-based, 1 from Holland, 2 from South Africa and 1 from Italy. A Framework analysis approach will be used to analyse the data from one-to-one interviews. Anticipated and emergent themes will be identified, defined and linked through continual comparison of data both within and across stakeholder groups.

**Conclusions**

The results of this study will give a clear description of current recruitment practice. This is turn will make it easier for trial methodologists and others to design and present evidence-based recruitment strategies and highlight what sort of evidence future research should provide.

This work is part of the Trial Forge initiative to improve trial efficiency.

### P421

#### Implementation into practice of isotonic regression for simultaneous assessment of toxicity and pharmacodynamic endpoints in a dose escalation trial

Rani Jayswal, Stacey Slone, Peng Wang, Vivek Rangnekar, Heidi L. Weiss
Markey Cancer Center, University of Kentucky
**Correspondence:** Rani Jayswal
*Trials* 2017, **18(Suppl 1):**P421

Over the last several years, oncology drug development has focused on molecularly targeted agents necessitating development of early phase clinical studies driven by a need to better understand underlying molecular mechanisms, provide delivery of targeted interventions in enriched patient subgroups and evaluate biological outcomes. Given the relatively safe profile of these targeted therapies, dose-finding clinical trials aim to evaluate both toxicity and biological effectiveness. An isotonic regression model was utilized in the design and conduct of a dose escalation trial to determine an admissible set of drug doses based on toxicity outcomes and selects the lowest dose with the highest biological response rate within the admissible set of doses. Simulations under different scenarios of dose toxicity and biological effectiveness rates demonstrate optimal operating characteristics of this design based on high selection probabilities of the correct safe and biologic effective dose, increased number of patients allocated to the right dose and low toxicities on the correct dose. Implementation of the isotonic regression is underway to guide dose escalation decisions. We present the performance of the model based on observed toxicity and pharmacodynamic (PD) biological response at each dose level as well as varying scenarios of toxicity and PD rates. As expected, dose escalation was guided by doses closest to the target biologic response rate within doses with acceptable toxicity rates. We also present comparison of performance and dose escalation decisions of this model compared to algorithm-based method for assessing biological or tumor response rate for dose escalation recommendations. The application of this model has provided a flexible and efficient use of limited patient data to determine not only safety but incorporation of proof of concept of biological response in the very early phases drug development.

### P422

#### Enquiry into how qualitative methods influence trials and their yields (equity): exploring registered trials that include a reported qualitative component

Clare Clement[1], Suzanne Edwards[1], Hayley Hutchings[1], Frances Rapport[2]
[1]Swansea University; [2]Macquarie University
**Correspondence:** Clare Clement
*Trials* 2017, **18(Suppl 1):**P422

### Background

Support for the use of qualitative methods within trials is widely recognised; however, reports indicate their full potential is not being realised, and issues ensue with the visibility, recognition and reporting of the qualitative approach in trials. It is important to understand the global view of the historical and contemporary make-up of qualitative research linked to trials if we are to identify potential areas of improvement. As part of a larger project to explore patterns in, and status of, the use of qualitative methods in trials, a review of trial registries was conducted to determine the extent of qualitative methods' use in trials.

### Methods

A search of clinical trials registers (globally) was conducted and decisions were made as to the suitability of the registry. Included registries were searched using the term 'qualitative' and returns logged and analysed by; 1. Year Registered 2. Country of Sponsor 3. Type of Trial (Drug, medical device, surgical or other). Trials were only included if the researcher confirmed that they included qualitative methodology (i.e. Using qualitative data collection methods and analysis techniques). Trials reporting qualitative testing and statistical analysis were excluded (i.e. 'qualitative' was tagged to quality of life measures, reports about medical tests were included such as 'qualitative urine test' or statistical tests, such as 'qualitative Fishers Extract test' were in evidence). Initially, all registers were searched from first record available up to 31st October 2013. The activity was repeated to update records up to November 2016.

### Results

Three registries were included and searched; clinicaltrials.gov, International Standard Randomised Controlled Trial Number Register (ISRCTN) and World Health Organisation (WHO) International Clinical Trials Registry Platform (ICTRP). The initial search in 2013 found an extremely small proportion of the 382,944 trials being carried out worldwide were utilising qualitative methods. The overall percentage of registered trials confirmed as including qualitative methods was less than 0.2%. This number appears to have increased over time, but is limited to 'developed countries' such as the U.K. and U.S.A. Most trials using qualitative methods appeared to be non-clinical, and were mostly testing behavioural interventions (87%). Of the small percentage of those trials which appeared clinical in nature, drug trials appeared to utilise qualitative methods more than either medical device or surgical trials (7%, 4%, 2% respectively). This was consistent across the three trial registries. Early indications from the repeated 2016 activity show a continuation of the initial pattern of less than 0.2% of a total 428,175 trials recorded using qualitative methods. Full findings will be reported at the conference.

### Conclusions

This study has highlighted the increasing use of qualitative methods over time and the use of these methods worldwide. However, the use of qualitative methods is restricted to 'developed countries' and non-clinical trials. More needs to be done to increase the use and benefits of qualitative methods in 'under-developed', or 'developing' countries, and the reasons for their lack of inclusion in clinical trials needs further investigation and development.

## P423

### "To have and to hold, from this day forward": understanding current practice regarding retention of trial participants

Clare Clement[1], Anne Daykin[1], Carol Gamble[2], Anna Kearney[2], Jane Blazeby[1], Mike Clarke[3], Athene Lane[1], Ali Heawood[1]
[1]University of Bristol; [2]Universityof Liverpool; [3]Queen's University
**Correspondence:** Clare Clement
*Trials* 2017, **18(Suppl 1):**P423

### Background

A frequent problem in clinical trials is the failure to attain complete outcome data for all randomised participants. Loss to follow-up (attrition) is problematic as it can introduce bias and reduce the power of a trial. However, until recently the main focus has been on enhancing recruitment rather than retention. As part of a multi-method study, this qualitative study, sought to explore retention strategies used by trial teams within ongoing trials and factors which may influence the adoption of such strategies.

### Methods

A purposive sample of five trials was selected from the NIHR HTA portfolio of ongoing trials. Semi-structured interviews explored strategies utilised by trial teams when collecting outcome data and in retaining participants. With the aid of nvivo, the interview data were analysed thematically using techniques of constant comparison.

### Results

Nineteen semi-structured interviews were conducted with trial team members along with three supplementary interviews with experienced senior trial managers. Participants recognised the context of the wider focus on recruitment to the detriment of retention by limiting motivation and resources to maximise retention. In trial researchers' accounts, their retention practices were shaped by factors which were recognised and conscious, and unrecognised and unconscious.

Interviewees recognised that fostering positive relationships with participants was a key strategy for enhancing participant retention. Interpersonal connections were forged by social relational actions such as making cups of tea during trial appointments and offering flexible appointments to suit the participant's needs. However, these activities required additional time which the trial researchers felt was not always acknowledged by funders or valued by the wider trial team.

Interviewees were not aware of how their own 'moral compass' influenced retention of participants. However such unrecognised or unconscious strategies were present in their accounts. They expressed how they often utilised their own beliefs and values regarding how to interact with participants, reflecting for example on how they would want their own parents to be treated, or projecting their own feelings onto a situation which may conflict with obtaining data.

The influence of the level of experience of team members on retention practices also appeared unrecognised. Researchers lacking experience reported having less confidence to pursue participants for outcome measure data, especially when participants wished to withdraw from the trial, worrying about coercion. More experienced researchers were happy to negotiate with participants in order to at least collect primary outcome data. Novice researchers presumed the participants wanted to withdraw from all aspects of the trial and made no further contact with them.

Researchers recognised that incentives influenced retention but seemed unaware that incentives may affect their own behaviour. Trial staff felt more confident and comfortable maintaining contact with participants over a period of time and more motivated to pursue acquisition of data from participants.

### Conclusions

Strategies deployed by trial researchers to enhance retention include a combination of recognised and unrecognised influences. These are underpinned by relational factors as well as researchers beliefs about their responsibilities and professional values. However, the pursuit of retention is constrained by a systemic emphasis on recruitment.

## P424

### Ambulatory oxygen in fibrotic lung disease (AMBOX): study protocol for a randomised controlled trial

Vicky Tsipouri[1], Dina Visca[1], Letizia Mori[1], Toby M Maher[2], Paul Cullinan[1], Nick Hopkinson[1], Athol U. Wells[1], Winston Banya[1], Huzaifa Adamali[3], Lisa G. Spencer[4]
[1]Royal Brompton Hospital; [2]Imperial College London; [3]Southmead Hospital; [4]University Hospital Aintree
**Correspondence:** Vicky Tsipouri
*Trials* 2017, **18(Suppl 1):**P424

### Background

Fibrotic Interstitial Lung Diseases (ILD) are rare, chronic and often progressive conditions resulting in substantial morbidity and mortality. Shortness of breath, a symptom often linked to oxygen desaturation on exertion, is tightly linked to worsening quality of life in these patients. Although ambulatory oxygen is used empirically in these patients, there are no ILD specific guidelines on its use. To our knowledge, no studies are available on the effects of ambulatory oxygen on day to day life in patients with ILD.

**Methods**

Ambulatory oxygen in fibrotic lung disease (AMBOX) is a multicentre, randomized, cross-over controlled trial (RCT) funded by the Research for Patient Benefit Programme of the National Institute for Health Research. The RCT will evaluate the effects on health status (measured by the King's Brief ILD questionnaire: K-BILD) of ambulatory oxygen used at home, at an optimal flow rate determined by titration at screening visit, and administered for a two-week period, compared to two weeks off oxygen. Key secondary outcomes will include breathlessness on activity scores, as measured by the University of California San Diego Shortness of Breath questionnaire, global patient assessment of change scores, as well as quality of life scores (St George's Respiratory Questionnaire), anxiety and depression scores (Hospital Anxiety and Depression Scale), activity markers measured by sensewear Armbands, pulse oximetry measurements, patient reported daily activities, patient and oxygen company reported oxygen cylinder use. The study also includes a qualitative component and will explore in interviews patients' experiences of the use of a portable oxygen supply and trial participation in a subgroup of 20 patients.

**Results**

We have completed recruitment of 87 patients to the study which represents one of the largest such cohorts world-wide. We are presenting here the trial design and baseline characteristics.

**Discussion**

This is the first RCT of the effects of ambulatory oxygen during daily life on health status and breathlessness in fibrotic lung disease. The results generated should provide the basis for setting up ILD specific guidelines for the use of ambulatory oxygen.

### P426

#### Implementing training for recruiters based on a new simple six-step model to promote information sharing and recruitment to RCTs: challenges and opportunities

Alba Realpe[1], Edward Dickenson[1], Rachel Hobson[1], Damian Griffin[1], Marcus Jepson[2], Jenny L. Donovan[2]
[1]University of Warwick; [2]University of Bristol
**Correspondence:** Alba Realpe
*Trials* 2017, **18(Suppl 1):**P426

**Objective**

The way trial information is presented is a key determinant of recruitment to randomised controlled trials (RCTs), which can be modified in order to encourage patients to participate. Recruiters in a full-scale surgical RCT comparing a surgical procedure with physiotherapy were trained based on a simple six-step model to support recruitment (Realpe et al. 2016). This paper shows how the model was implemented. We compared communication practices in consultations where patients decided to take part versus those consultations in which patients declined participation in the trial in order to validate and expand the six-step model.

**Methods**

A sample of recruitment consultations with participants (n = 32) and decliners (n = 28) were recorded during a full-scale RCT. Recordings were analysed using techniques of thematic analysis and focused conversation analysis pioneered in previous studies.

**Results**

Recruitment to trial was successful, with 60% of patients approached across 20 centres agreeing to take part in the RCT. Recruiters used the six-step model to structure their consultations. However there were differences in the way recruiters addressed patient questions and concerns in participants' versus decliners' consultations. Differences were also observed in patients' view of the trial, those who declined to take part expressed more concerns and preferences and asked fewer questions than participants in the trial.

**Conclusions**

The six-step model provided a useful framework for recruitment to RCTs that was easy to implement. However further skill development to maintain patient equipoise is required when addressing patient questions and preferences. Patient views and their particular circumstances are important factors when deciding whether or not to participate in a surgical RCT with a less intensive comparison arm.

### P427

#### A case study method to support and promote recruitment at a multi-centre RCT comparing surgical versus non-surgical treatments

Alba Realpe[1], Edward Dickenson[1], Rachel Hobson[1], Damian Griffin[1], Marcus Jepson[2], Jenny L. Donovan[2]
[1]University of Warwick; [2]University of Bristol
**Correspondence:** Alba Realpe
*Trials* 2017, **18(Suppl 1):**P427

**Objective**

Multi-centre RCT designs provide robust evidence of therapeutic effect of health interventions. However participating centres often differ in how well they conduct the trial and the number of patients successfully recruited. This paper describes barriers different research teams encountered when conducting a complex RCT comparing a surgical procedure with physiotherapy, and the actions taken by the trial management group to overcome obstacles that were hindering recruitment.

**Methods**

We conducted 22 interviews with principal investigators and research associates at 14 sites involved in the delivery of a surgical RCT that compared hip arthroscopy and physiotherapy for hip pain. Interview transcripts were analysed thematically and case study approaches were utilised to present results to the trial management group.

**Results**

Research teams reported difficulties related to logistics (e.g. Room space); motivation (e.g. PI reluctant to approach patients); and skill (e.g. Lack of knowledge about the treatment arms). Similar Issues were shared by sites that recruited to target and those that did not, however there were differences in the team's response to challenges. Whilst on-target sites found local solutions to issues or support through their research infrastructure or the trial TMG, off-target sites usually did not show proactivity. Site profiles were created and action plans designed based on aspects that were particular to the individual sites. These plans were implemented in collaboration with site teams.

**Conclusions**

This qualitative study added to the growing evidence of how aspects of team functioning are important for recruitment to complex RCTs. Trial Management Groups can help research teams identify and address issues, and therefore contributing to a sense of ownership by the research team. Empowering research teams to find solutions at local level is essential to conduct multi-centre RCTs successfully.

### P428

#### When is enough, enough? Replication of behaviour change interventions to minimise attrition of follow up questionnaires

Anne Duncan[1], Beatriz Goulao[1], Patrick Fee[2], Fiona McLaren-Neil[2], Ruth Floate[2], Fiona Ord[2], Hazel Braid[2], Debbie Bonetti[2], Jan Clarkson[2], Craig Ramsay[1]
[1]University of Aberdeen; [2]University of Dundee
**Correspondence:** Anne Duncan
*Trials* 2017, **18(Suppl 1):**P428

**Background**

Low response rates to participant follow-up questionnaires in trials place the validity and generalisability of results in jeopardy. Evidence provided by the iquad Trial demonstrated that using the Theoretical Domains Framework (TDF) to identify theoretical targets for behaviour change interventions and incorporating these into a theory-based cover letter randomly issued to 1192 participants with their postal questionnaire at year 1 and year 2 of annual follow-up had a beneficial impact on response rates [1]. Lack of replication of research findings has been highlighted as a key limitation across health and related disciplines. To address this limitation, the strategy was replicated in the INTERVAL Dental Recalls trial to investigate if the intervention would improve participant questionnaire response rates in a similar patient population (primary dental care), with a similar level of non-response.

**Method**

1867 INTERVAL participants were sent annual questionnaires at year 2 and 3 of follow-up and randomly allocated to receive either the standard covering letter (control group) or theory-based cover letter (intervention cohort). The response rates between the groups to both the iquad and INTERVAL replicated SWAT were estimated with 95% confidence intervals. A fixed effect meta-analysis was calculated using the Mantel-Haenszel method.

**Results**

The participants in both the iquad and INTERVAL trials had similar baseline characteristics; the mean age of INTERVAL participants was 48.4 (14.9) years, 60% female and iquad participants 47.8 (15.7) years, 64% female. The response rate in INTERVAL was 67% for the intervention cohort and 66% in the control group. There was a +1% difference (95% CI −3 to 5%) between groups favouring the intervention. In iquad the response rate was 72% in the intervention cohort and 65% in the control group. There was a +7% difference (95% CI +2 to +12%) between groups favouring the intervention. On meta-analysis of results there is a risk difference of 3% (95% CI 0 to +7%) in favour of the intervention.

**Conclusions**

To our knowledge, this is the first true replication of a behaviour change intervention for improving response rates in a similar population. These results indicate that inclusion of a theory-based cover letter with postal questionnaires provides a cheap and effective method for improving participant response rates by 3% compared with a standard letter in a dental primary care population. We believe this study provides strong evidence of the effectiveness of the intervention in this population. However, the study has raised interesting methodological challenges around when should replication stop and the role of context (settings and population). As such further replication of this strategy is planned in different trial settings and populations through the Trial Forge initiative (http://trialforge.org) and through the Medical Research Council (MRC) Hubs for Trial Methodology Research (https://www.qub.ac.uk/sites/thenorthernirelandnetworkfortrialsmethodologyresearch/swatswarinformation/) to add to the evidence base.

### P429

#### Recruitment in GP practices: research assistant or research nurse?

Sarah Tearne
University of Oxford
*Trials* 2017, **18(Suppl 1):**P429

**Background**

It's important when designing clinical trials to select an appropriate method of recruitment. Traditionally research nurses recruit participants from GP practices. They are often familiar to the patients which could mean those patients are more likely to enter and complete clinical trials. A randomised controlled trial to test the effectiveness of a brief intervention for weight management in primary care compared practices using research nurses (N = 17) with practices using research assistants (RA) (N = 44) to opportunistically recruit participants.

**Aims**

Compare two different methods of recruitment, specifically the effect on participant enrolment and follow up.

**Methods**

Data was analysed as proportions. We reported the number of those enrolled and those being followed up in each group, divided by the total number eligible and the total number enrolled in each group respectively i.e. the risk ratio with 95% confidence intervals.

**Results**

93.8% in the RA group and 96.6% in the nurse group (RR 0.97 95% CI 0.94, 0.99) were enrolled of those that were eligible. 58.1% in the nurse group and 81.1% in the RA group (RR 0.71 95% CI 0.65, 0.79) were followed up at 3 months.

**Conclusions**

Research nurses were slightly more effective at successfully enrolling eligible participants into the trial than research assistants however the difference between the groups is barely significant. Once enrolled, participants were more likely to return for follow up in the RA group. This significant result suggests that using research assistants for recruitment is more likely to result in better follow up rates.

### P430

#### Development of the CORKA trial screening tool for identifying patients at increased risk of poor outcome following knee replacement

Michael Schlussel, Gary Collins, Susan Dutton, Karen Barker
University of Oxford
**Correspondence:** Michael Schlussel
*Trials* 2017, **18(Suppl 1):**P430

**Background**

Community based Rehabilitation after Knee Arthroplasty (CORKA) is a multicentre two-arm individually randomised controlled trial with blinded outcome assessment at 12 months. It aims to determine if a multi-component rehabilitation programme, provided to patients who had knee replacement (KR) and are deemed at risk of poor outcome, as measured by the Late Life Function and Disability Instrument (LLFDI) score, is better than usual care.

**Objective**

To describe the development of the trial's screening tool to recruit KR patients at greater risk of poor outcome and who therefore might benefit more from the intervention.

**Methods**

The screening tool was developed based on the principles of prognostic model development, using data from the KAT randomized clinical trial [1] which contains pre-operative and 12 months outcome data on more than 2,192 KR patients. As a proxy for poor outcome, since the KAT trial did not include the LLFDI score, poor outcome was defined as a score 26 in the Oxford Knee Score (OKS). Pre-operative characteristics considered as candidate predictors in the development of the screening tool included age, sex, height, body mass index (BMI), mobility, ASA grade, SF-12 (questions 6 and 11) and OKS (question 1) components. Multivariate imputation by chained equations (MICE) was used to handle missing data in the KAT dataset. Ten complete datasets were produced by MICE. One of these datasets was selected at random and multivariable logistic regression models were fitted to identify the statistically significant predictors of poor outcome after KR. Predictors were selected by using backwards elimination (stepwise) procedure. The final model was aimed to be simple and easy to implement in the clinical setting, considering both the clinical and statistical relevance of the predictors. Model simplification was done by rounding up the logistic regression coefficients (odds ratio) of the predictors in the final model to the nearest integer. Model performance was evaluated in terms of discrimination and calibration. Discrimination was quantified by the c-index (area under the receiver operating characteristics curve). Calibration was assessed by grouping individuals into tenths of predicted risk and graphically comparing the agreement between the mean predicted risk and the observed events in each tenth. The cut-offs to classify individuals at increased risk of poor outcome was determined with the aim of achieving a balance between model's specificity and recruitment feasibility.

**Results**

Subjects in the KAT dataset were aged 71(SD = 7.1) years on average, with a mean ASA grade of 2(SD = 0.6). From a total set of nine candidate predictors, four were selected for the screening tool: BMI, ASA grade, OKS question 1 and SF-12 question 6. Model discrimination, as measured by the c-index was 0.67. The screening tool score range is 0–10 and patients scoring 5 or more (29% of the KAT sample) are considered at increased risk of poor outcome following KR.

#### Conclusions
We developed a simple and objective screening tool to identify patients at increased risk of poor outcome for inclusion in to the CORKA randomized clinical trial, with a moderate discriminatory ability.

#### Reference
[1] J Bone Joint Surg Am. 2009;91:134–41.

### P431
### Informed consent and proxy decision making for research involving adults lacking capacity: a systematic review (framework synthesis)
Victoria Shepherd, Fiona Wood, Kerenza Hood
Cardiff University
**Correspondence:** Victoria Shepherd
*Trials* 2017, **18(Suppl 1):**P431

#### Introduction
Decisions about the participation of adults lacking mental capacity in medical research are complex, and raise considerable legal and ethical issues. There are differences between decisions relating to the medical treatment of adults lacking capacity, and those concerning their participation in medical research. Carers and relatives of adults lacking capacity are regularly called upon to make such decisions on their behalf, however little is known about the ethical basis on which these proxy decisions are made and there is a dearth of information or support available. The coming decades are expected to see a significant rise in health challenges resulting from ageing populations, with a proportionate rise in conditions characterised by cognitive disorders. Ambitious UK research agendas have been set out in order to address these challenges, however these will require considerable numbers of research participants.
### Background
There are specific legal provisions in England and Wales governing proxy decision making by another individual, such as a family member of friend, for those unable to provide consent for themselves to participate in research. Data regarding the ethical and regulatory factors influencing these decisions, and interventions to inform and support those involved, are urgently required in order to maximise the participation of adults lacking capacity in research. Research participants, their families and carers, clinicians and researchers require a clear, evidence-based ethical framework for research enrolment of adults lacking capacity. This systematic review forms part of an NIHR Doctoral Research Fellowship to investigate informed consent and proxy decision making in research involving adults lacking capacity, and the development of an intervention to support informed proxy decision making, set within ethical and legal frameworks.
### Methods
A mixed methods systematic review will be conducted to determine the ethical and legal issues encountered in proxy decision-making for research participation by adults lacking capacity, using a framework synthesis approach. The aim is to synthesise empirical evidence from qualitative, quantitative or observational studies which examine the relevant ethical issues. The review will be registered with PROSPERO database of systematic reviews.
### Results
The findings from the systematic review will be presented, which will include an examination of the ethical issues encountered, what factors are involved when proxy decisions are made, and factors that affect the quality of informed consent and proxy decision making in practice. The review will provide an overarching synthesis of proxy decision-making for research participation, and the development of a conceptual framework.
### Conclusions
This systematic review will examine a range of factors encountered in research involving adults lacking capacity, and what influence these and other factors have on informed consent and proxy decision making in practice. The findings will be used to develop a conceptual framework of proxy decision making which will form the basis of a subsequent qualitative study to explore how proxy decisions are made, and whether legal and ethical obligations are being met. The review and the qualitative study will then be used to determine the factors that must be included in a decision support intervention for research participation by proxy decision makers for adults lacking capacity.

### P432
### New methods for categorising recruitment research: a case study
Ceri Rowlands[1], Leila Rooshenhas[1], Jonathan Rees[2], Jane M. Blazeby[3]
[1]MRC conduct-II Hub for Trials Methodology Research, School of Social & Community Medicine, University of Bristol; [2]Division of Surgery, Head & Neck, University Hospitals Bristol NHS Foundation Trust; [3]MRC conduct-II Hub for Trials Methodology Research, School of Social & Community Medicine, University of Bristol and Division of Surgery, Head & Neck, University Hospitals Bristol NHS Foundation Trust
**Correspondence:** Ceri Rowlands
*Trials* 2017, **18(Suppl 1):**P432

#### Background
Research into optimising recruitment to RCTs is commonly undertaken, however there is no agreed method for organising and reporting studies. Adequately describing and classifying recruitment study types may enable researchers to evaluate and compare studies more reliably.
### Aim
This study developed and applied a categorisation system for different recruitment studies, encountered during a systematic review of recruitment to RCTs in unplanned hospital care (UHC), to inform future recruitment research.
### Methods
Search strategy
The ORRCA (Online Resource for Recruitment Research in Clinical Trials; www.orrca.org.uk) database was utilised in this systematic review. ORRCA includes studies of all designs, systematically extracted from the literature, reporting on recruitment into RCTs and non-randomised clinical studies. In this review, ORRCA was searched for primary research reports of studies that reported on recruitment to RCTs in adult patients receiving UHC.
Development of study categories Reading the articles led to initial categorisation of the recruitment studies into those with a randomised or non-randomised recruitment designs. Iterative refinement of the study structured categories through discussion between study authors (CR, JMB, LR, JR).
It was noted that papers reporting surveys in the community (community consultations) had been undertaken to establish the likelihood of recruitment success or acceptability of a trial. In recognition of this, a clear differentiation was made between studies that focused on recruitment to an actual clinical RCT (a 'host RCT') versus potential recruitment to a RCT that did not yet exist (a 'hypothetical RCT').
Latterly a further categorisation was introduced to classify whether the recruitment study evaluated an intervention to modify recruitment, or simply reported on recruitment experiences. The final classification for papers was formulated based on whether i) randomised or non-randomised study design was employed during the recruitment study ii) an intervention to optimise recruitment was evaluated, and iii) a host or hypothetical RCT was used.
Category A - Randomised controlled trials of interventions to optimise recruitment within one or more host RCTs Category B - Non-randomised studies of interventions to optimise recruitment within one or more host RCTs Category C - Non-randomised studies without interventions evaluating recruitment to one or more host RCTs Category D - Randomised studies to consider recruitment within proposed hypothetical RCTs (community consultations) Category E - Non-randomised studies to consider recruitment within proposed hypothetical RCTs (community consultations).
### Results
3114 papers were available in ORRCA and 39 met the inclusion criteria. The new categorisation was able to be applied to all papers with 1, 11, 16, 0 and 11 within categories A to E respectively.
### Conclusions
This case study illustrates new methods for categorising recruitment studies. It has potential utility to researchers by encompassing the different aspects of the recruitment study design and the use of real/hypothetical RCTs. This categorisation requires further evaluation in other recruitment settings to establish its validity and role.

### P433

#### Utilizing remote participant visits to boost retention in a long-term clinical trial

Ashley Hogan, Hanna Sherif, Nicole Butler, Tsedenia Bezabeh, Adrienne Gottlieb, Ella Temprosa
George Washington University
**Correspondence:** Ashley Hogan
*Trials* 2017, **18(Suppl 1):**P433

The success of long-term studies rely heavily on the ability to retain participants for the entire study duration which may span much of the participant's adult life. Researchers must accommodate participants' life changes including moving to locations that are no longer near a clinical center, personal circumstances that prevent in-person clinic visits, and most importantly aging. Prolonged illness and decreased mobility of aging participants create barriers to clinic access for data collection. In such cases, performing collection at convenient locations for participants including their home, work or nursing home may boost retention. In studies with event driven analysis approach, the data for every participant is valued. Their individual contribution may be small but their retention is essential to the success of the study; as a result, a critical concern is how we can expand our reach and continue to maximize data collection on all participants. Questionnaire data collected over the phone may not be enough and phenotypic data can offer a more complete picture. Thus to improve retention and minimize participant burden, cost-effective approaches to conduct remote visits can be implemented to collect anthropometric measurements and biospecimens with the use of external examination services.
However, many challenges abound in conducting sporadic remote visits by an external examination service technician. A clear and precise protocol is essential to ensure fidelity and consistency in data collection and equipment. Prioritizing data collection for non-clinic visits will help simplify the visit flow for external technicians to balance the capture of essential outcomes and participant burden. Training and communication are critical to facilitate interactions among the external examination service central office, the technician completing the visit, the clinical coordinator, the coordinating center staff, and the participant. In this presentation, we will describe the process of working closely with an external examination service for a long-term multi-center clinical trial with an aging cohort. We will present our experiences, both the successes and failures, over the first year of remote visit implementation within the framework of a national multi-center clinical trial. If long-term studies can overcome these obstacles, the use of external examination services to conduct remote visits may provide a cost-effective solution to boost participant retention and support study validity in otherwise hard to reach populations.

### P434

#### Site training to improve healthcare practitioners? Confidence in recruiting to a challenging critical care trial

Kerry Woolfall[1], Louise Roper[2], Amy Humphreys[3], Mark D. Lyttle[4], Shrouk Messahel[5], Elizabeth Lee[5], Joanne Noblet[5], Anand Iyer[6], Carrol Gamble[7], Helen Hickey[7]
[1]The University of Liverpool; [2]Department of Psychological Sciences, The University of Liverpool; [3]Clinical Trials Research Centre (CTRC); [4]Emergency Department, Bristol Royal Hospital for Children; Faculty of Health and Applied Sciences, University of the West of England; [5]Emergency Department, Alder Hey Children's NHS Foundation Trust; [6]Neurology Department Alder Hey Children's NHS Foundation Trust; [7]Clinical Trials Research Centre (CTRC), North West Hub for Trials Methodology Research
**Correspondence:** Kerry Woolfall
*Trials* 2017, **18(Suppl 1):**P434

##### Background
Many clinical trials experience recruitment difficulties, leading to underpowered studies, costly extensions or early closure. Trials in paediatric critical care encounter additional practical and ethical difficulties as there is no time to seek prior informed consent in an emergency situation. Eclipse is an unblinded pragmatic randomised controlled trial that explores the treatment (levetiracetam versus phenytoin) of status epilepticus in children. Challenges to the success of eclipse include: a vulnerable target population (children aged 6 months to <18 years); the need to administer the intervention without prior informed consent (deferred consent); and use of levetiracetam, an anti-epileptic medication which is not traditionally usually used in this clinical setting. We evaluated the effectiveness of site training on healthcare practitioners' (HCP) confidence in the recruitment of patients to eclipse. The interactive site training included: protocol presentations from the trial team; screening and randomisation simulation (video and real-time); a deferred consent scenario video informed by pre-trial feasibility work with parents, and a question and answer session.

##### Methods
Mixed method study including a 14 item questionnaire administered before and immediately after the site training as well as telephone interviews with eclipse HCPs in the first 12 months after site opening.

##### Results
In total, 156 HCPs from 25 UK hospital completed a before and after site training questionnaire. We interviewed eight HCPs involved in patient-recruitment and deferred consent in eclipse. Prior to site training, HCPs were concerned about recruitment because of a lack of knowledge about the trial protocol and apprehensions about parents' response to deferred consent. We found that site training improved HCP confidence in the trial, including being better able to discuss the study with parents ($p < 0.001$), explain randomisation ($p < 0.001$) explain deferred consent ($p < 0.001$) and address parents' objections to their child being randomised ($p < 0.001$). HCPs valued the clarity and content of site training and described how videos helped them to visualise recruitment and consent processes. HCPs offered suggestions about how the trial management team could provide ongoing recruitment and consent support through study updates, recruitment-training tips and advice from study team members as and when required.

##### Conclusions
Interactive site training can assist important HCP 'buy in' for challenging clinical trials. Our findings highlight how pre-trial feasibility work with parents can improve HCP confidence in recruitment and deferred consent seeking in a paediatric critical care trial.

### P435

#### Seeing the light at the end of the carpal tunnel: the challenges of recruiting sites to a clinical trial of an investigational medicinal product in primary care

Helen Myers[1], Claire Burton[2], Michelle Robinson[1], Graham Davenport[2], Krysia Dziedzic[2], Danielle van der Windt[2], Linda Chesterton[2], Edward Roddy[2], Elaine Hay[2]
[1]Keele Clinical Trials Unit; [2]Institute for Primary Care and Health Sciences
**Correspondence:** Helen Myers
*Trials* 2017, **18(Suppl 1):**P435

##### Background
Sub-optimal participant recruitment rates are common in trials. A multitude of factors have been suggested to explain this [1], including the task of opening sites to recruitment. Clinical trials of investigational medicinal products (CTIMPs) may pose additional challenges in primary care compared to other healthcare settings due to the need to engage busy general practitioners (GPs) in research processes. A recent report [2] highlighted the increasing pressures on GPs to meet the clinical needs of patients during routine consultations. Given this backdrop, facilitating GPs to recruit participants can prove challenging. This abstract presents the experiences of opening sites to recruitment for a nationwide clinical trial based in primary care: Injection versus Splinting in Carpal Tunnel Syndrome (instincts) [3].

#### Methods
Recruiting sites were GP practices, GPs using a 'hub-and-spoke' model, and primary-secondary care Interface Clinics. Practices were identified via the Primary Care Rheumatology Society and through the relevant Clinical Research Networks (CRN). Training was undertaken at regional workshops and individual sites. Some sites had access to Research Nurses, or support from the CRN for set-up. Support with completing site set-up paperwork was offered to sites by the Clinical Trials Unit (telephone or visit).
### Results
Of the 59 sites expressing interest, 27 opened, 12 were trained but did not open, and 20 were not trained and did not proceed further. Of the sites which opened to recruitment, 13 were GP practices, seven were GPs using a 'hub-and-spoke' model, and seven were Interface Clinics. Time from training to site opening ranged from two to 24 months. Delays between training and site opening were experienced for a variety of reasons related to overall trial set-up and site-specific set-up. The change from Primary Care Trusts to Clinical Commissioning Groups delayed sites engaging with the trial. Site-specific issues included delays completing site set-up paperwork and meeting the additional requirements for conducting CTIMPs (e.g. Good Clinical Practice training). Delays to site opening meant some sites needing re-training and others never opening. Reasons for trained sites never opening included changes in clinician availability and service re-structure. As a consequence, there was a need to recruit sites for longer than anticipated. Support from a Research Nurse or CRN had a positive effect on site set-up. GPs using a 'hub-and-spoke' model and Interface Clinics were eventually targeted as priority for site opening, as initial patterns of recruitment showed these sites gave a better return in terms of recruitment for the time invested in site set-up.
### Conclusions
Targeting GPs using a 'hub-and-spoke' model and Interface Clinics was generally most efficient for site opening and most productive for subsequent participant recruitment. Web-based initial training would have allowed sites to register interest after seeing what the trial involved, thereby saving time and resources. Ensuring sites were 'research ready' and had completed site set-up paperwork before training would have reduced the delay in site opening. GPs were required to offer each participant several appointment slots to screen, consent, randomise and treat, which may have dissuaded them from engaging: providing support with these activities may have encouraged more sites to participate.

### P436
## Retention in randomised trials: what matters to trial managers?
Katie Gillies, Shaun Treweek
University of Aberdeen
**Correspondence:** Katie Gillies
*Trials* 2017, **18(Suppl 1):**P436

#### Background
It is common for many trial participants (sometimes more than 20%) to drop out before the trial finishes. Drop out seriously affects the credibility of trial results and significantly affects a trials potential to influence clinical practice. Recent estimates have shown that in up to 53% of trials the results could have been overturned if the outcomes from those who had dropped out were known. Trial Managers are often faced with identifying ways to improve retention but the evidence base on effective methods in this area is lacking. Exploring and identifying Trial Managers experience and tacit knowledge regarding what they perceive as the biggest barriers and facilitators for retention in RCTs can contribute to this evidence base and identify areas for further research.
### Methods
A web-based survey was conducted as a scoping exercise to explore what Trial Managers perceive as the biggest barriers and facilitators for retention in randomised trials. Trial Managers were sent an invitation to complete the survey through the UK Trial Managers Network (UK TMN) listserv to all listed members (i.e. Those trial managers who are employed on a publicly funded trial and registered with the UK TMN). In addition to questions about demographics, there were 4 broad open-ended questions that asked Trial Managers about: experience of retention and what they perceive as main issues; thoughts on ways to improve retention; how trial design could be changed to improve retention; and to identify one thing in relation to retention that would make their lives easier. Demographic data was analysed using descriptive statistics and the free text responses were coded using a thematic analysis approach.
### Results
The email invite was sent to 501 list SERV members and we received 48 responses (9.6% response rate). The duration of trial management experience of respondents ranged from 0.8 to 34 years (median 5 (Q1:3; Q3: 12.25)). The types of trial managed by respondents showed wide variation and covered all phases of trial (Phase I-Phase IV) including pilot trials; trials set within primary, secondary and tertiary care; with children and adults; in a range of clinical areas; and interventions under investigation also varied (included CTIMPS, devices, surgery, educational, service level). The findings could be grouped in two main themes: considerations relating to trial design; and considerations relating to participant perspectives. The overwhelming issue raised in the 48 responses from across the UK was that many aspects of retention are context dependent: at the level of the clinical condition; its associated population; and the individual trial (i.e. Type of follow-up, differences across sites with relation to how retention is discussed and the rapport between recruiter and participant).
### Discussion
This survey identifies some of the main barriers and facilitators to trial retention as perceived by Trial Managers. Specifically, the results focus on trial design and participant perspectives, how poor protocols make retention harder, and identify potential priorities for future evaluation.

### P437
## Can trial managers predict whether sites will recruit to target or not - Results from the estimating site performance (ESP) study
Seonaidh Cotton, Kirsty Shearer, Anne Duncan, Hanne Bruhn, Shaun Treweek
University of Aberdeen
**Correspondence:** Seonaidh Cotton
*Trials* 2017, **18(Suppl 1):**P437

#### Background
It is estimated that around 50% of trials fail to recruit to target. Potential implications of this is that trials require extensions to their recruitment period, their sample size is revised, or the trial is closed, leaving the clinical question unanswered. In multi-centre trials, substantial time and effort is spent setting up clinical recruitment sites, and this has inherent cost. Experience suggests that some sites will recruit to target and others will not. The more sites that fail to recruit to target, the less likely the trial will meet recruitment targets.
### Aims
We explored whether trial managers (involved in recruitment site set-up) were able to predict whether a site would recruit to target or not before the site was opened to recruitment, the reasons for these predictions, and their subsequent reflections.
### Methods
Trial managers based in a registered CTU involved in setting-up recruitment sites during 2014/15 predicted the recruitment success of each site they opened: whether the site would recruit to target (ie recruit the number of participants documented in the site agreement) or not, and reasons for this. Predictions were placed in sealed envelopes and opened after a minimum of 8 months recruitment at each site. A focus group was held with the trial managers where predictions were revealed and compared with the actual recruitment achieved by the site; reasons for and accuracy of the prediction were discussed.
### Results
10 trial managers working across 7 randomised trials and one non-randomised diagnostic study participated; 56 predictions were made.

Of 39 predictions about sites involved in randomised trials, 31 (79%) were that the site would recruit to target; 17 of these (55%) were correct and the site met target. Of 17 predictions made about sites in the diagnostic study, 12 (71%) were that the site would recruit to target; all 12 did so. Reasons for positive predictions were similar across the types of studies and included engaged/enthusiastic PI/team, experienced research nurses, consideration of trial logistics prior to site initiation. Eight sites involved in randomised trials were predicted not to recruit to target; 6 of these predictions were correct. Five sites involved in the diagnostic study were predicted not to recruit to target; only 1 site did not recruit to target. Reasons given for these predictions included lack of interest/engagement, slow responses, involvement in multiple studies. Results from the focus group will be presented. We will also explore whether there is any difference between accuracy of prediction in sites where training was on-site or carried out remotely.

#### Conclusions
Trial managers were generally optimistic in their predictions. For the randomised studies, predictions were more likely to be correct when trial managers predicted that a site would not meet recruitment targets. We propose that the trial manager's tacit knowledge at site set up may help to target time and resources more effectively during site set-up: the implication of this is that time and resource may be directed away from sites predicted to not meet recruitment targets.

### P438

### Keys to successful participant response rates in a 30 year longitudinal study

Lisa Reeves
University of North Carolina
*Trials* 2017, **18(Suppl 1):**P438

#### Background
ARIC (Atherosclerosis Risk in Communities) is a prospective study funded by the National Heart, Lung and Blood Institute (NHLBI) that began over 30 years ago in 4 communities in the United States: Forsyth County, North Carolina; Jackson, Mississippi; Minneapolis Minnesota; and Washington County Maryland. At that time, 15,792 participants between the ages of 45 and 64 were enrolled and have been successfully followed over the years with an impressive response rate.

#### Methods
ARIC's key to successful retention include information that was gathered at visit 1, and consistent contact with the participant over the years. At visit 1, information on the participants' address, physicians, contacts, and other items were well documented. Spousal participation in the study was also encouraged in hopes of keeping both spouses involved. Annual and semi-annual phone interviews that include health related questions also verify contact information. This includes current participant physical and mailing address; multiple contact names, addresses and phone numbers; physician name, address and phone number; as well as a designated proxy who can represent the participant given any future health or cognitive problems (which is especially important with an aging cohort). Phone interviews are done at optimal times such as evenings or weekends. At the time of the call, interviewers have at their fingertips all the relevant information on the participant via reports produced on a web-based data management system called Carolina Data Acquisition and Reporting Tool, or CDART (developed by the ARIC coordinating center at the University of North Carolina at Chapel Hill). This report provides participant information to facilitate the call such as living arrangements, potential hearing loss, study history and other administrative notes. Long-term and dedicated interviewing staff provide familiarity for the participant, and go through annual interviewing recertification to ensure quality. Periodic clinic visits are done (currently conducting visit 6) where various interviews and medical tests are completed. The participants are offered a small stipend for the visit, as well as comprehensive test results that they can share with their physicians. If the participant is unable to come to the clinic, in-home visits are offered. To further engage the participant, periodic newsletters and birthday/greeting cards are mailed, and a participant focused public website (in both English and Spanish) is available.

#### Results
Currently, the ARIC study follow-up has over 80% response rate of those participants still alive, providing over 30 years of robust data sets for investigators worldwide, and contributed to over 1600 peer-reviewed articles. ARIC continues to follow these participants and has plans for its seventh clinic visit in 2017.

### P439

### Can behaviour change theory increase questionnaire response rates within trials?

Kirsteen Goodman[1], Suzanne Hagen[1], Doreen McClurg, Nicole Sergenson[1], Susan Stratton[1], Shaun Treweek[2]
[1]Nursing, Midwifery and Allied Health Professional Research Unit; [2]Health Services Research Unit, University of Aberdeen
**Correspondence:** Kirsteen Goodman
*Trials* 2017, **18(Suppl 1):**P439

#### Background
Maximising recruitment in trials is an important goal for trial teams; however a trial that has met recruitment targets can be underpowered with inconclusive results if retention rates are poor. Trialists anticipate around 10% of participants will withdraw from a trial or are "lost to follow up", however, there are few interventions with high quality evidence of benefit for increasing retention in trials [1–2].
The need for more rigorous evaluations of interventions to increase retention in trials has resulted in the Trial Forge Initiative (http://trialforge.org) coordinating SWAT studies ("study within a trial"): evaluations of trial methods innovations embedded within a host trial Research teams can use the same SWAT protocol, thus the results can be combined in a meta-analysis which may provide high-quality evidence to inform their trial design. The SWAT 24 protocol focuses on increasing the return of outcome data collected through participant-completed questionnaires. The completion and return of the questionnaires is a "behaviour" Which could be influenced by an intervention targeting participants' willingness "to do" this behaviour.
The iquad trial team [3] has developed a template for a theory-based cover letter (the intervention) using the Theoretical Domains Framework (TDF) [4] to identify theoretical targets for behaviour change interventions. This letter is issued to participants with follow-up questionnaires.
In the iquad trial the theory-based letter was associated with a 6% increase in questionnaire return rate when compared with the standard letter [5]. This is promising but needs replication to investigate if this effect can be repeated in other trials with different patient populations that may have different barriers to "behaviour change".

#### Objectives
To ascertain whether a theory-based cover letter accompanying patient follow-up questionnaires improves response rates in two trials with differing patient groups; the AMBER (neurogenic bowel) and the OPAL trial (urogynaecology) [6,7].

#### Methods
AMBER and OPAL trial participants are randomly assigned to receive a theory-based cover letter (group 1) or a standard letter (group 2) with follow-up postal questionnaires; 24 weeks in AMBER; 12 and 24 months in OPAL. Questionnaire response rates across time-points in the AMBER and OPAL trials will be presented.

#### Results
Sixty-four letters (group 1:32, group 2: 32) will be sent in the AMBER trial (41 sent to date). 384 letters (group 1: 205, group 2: 179) will be sent in the OPAL trial (174 sent to date). These data will be pooled with the iquad data to create a cumulative meta-analysis, which will also form part of future versions of the Cochrane review of interventions to improve retention.

#### Conclusions
We will provide evidence as to whether a theory-informed questionnaire cover letter can improve trial questionnaire response rates, and whether some patient populations are more receptive to these letters than others.

References
1. Treweek S, Mitchell E, Pitkethly M, Cook J, Kjeldstrøm M, Johansen M, Taskila TK, Sullivan F, Wilson S, Jackson C, Jones R, Lockhart P. Strategies to improve recruitment to randomised controlled trials. Cochrane Database of Systematic Reviews 2010, Issue 4. Art. No.: MR000013. doi:10.1002/14651858.MR000013.pub5.
2. Brueton VC, Tierney J, Stenning S, Harding S, Meredith S, Nazareth I, Rait G. Strategies to improve retention in randomised trials. Cochrane Database of Systematic Reviews 2013, Issue
3. Jan E Clarkson et al. IQuaD dental trial; improving the quality of dentistry: a multicentre randomised controlled trial comparing oral hygiene advice and periodontal instrumentation for the prevention and management of periodontal disease in dentate adults attending dental primary care.BMC Oral Health 10/2013; 13(1):58
4. Michie et al. Making psychological theory useful for implementing evidence based practice. Qual & Safety in Health Care 2005; 14:26–33
5. Duncan et al. Improving trial questionnaire response rates using behaviour change theory. Trials 2015, 16(Suppl 2):P92
6. The AMBER Study is A UK collaborative study funded by the National Institute for Health Research, Health Technology Assessment Programme (Project: 12/127/12) and sponsored by Glasgow Caledonian University (CI: Professor Doreen McClurg). Ethical approval was granted by the West of Scotland Research Ethics Committee.
7. The OPAL Study is a UK collaborative study funded by the National Institute for Health Research, Health Technology Assessment Programme (Project:11/71/03) and sponsored by Glasgow Caledonian University (CI: Professor Suzanne Hagen). Ethical approval was granted by the West of Scotland Research Ethics Committee.

### P440

#### The two-stage treatment selection (TSTS) design: a novel approach to treatment selection in clinical trials

Matthew Parkes[1], Mark Lunt[1], Philip S. Pallmann[2], David T. Felson[3]
[1]University of Manchester; [2]Lancaster University; [3]Boston University
**Correspondence:** Matthew Parkes
*Trials* 2017, **18(Suppl 1):**P440

Clinical trial designs which focus resources on treatments that show promise in early accrued data are efficient and desirable, particularly when resources are constrained. To help stop trials, or trial arms, which show a low likelihood of confirming a treatment effect at final analysis, typically one could include an interim analysis in a parallel-groups ?A3B2 show $132#?>confirmatory clinical trial, or adopt an adaptive multi-arm design, for example a seamless phase II/III design. These strategies all feature one or more interim analyses during trial recruitment to assess whether the trial is to continue to completion. In a typical interim analysis, the hypotheses tested at interim/transition are principally concerned with whether the trial is likely to observe a treatment effect which meets the chosen limit for statistical significance, at the final analysis. This analysis only indirectly tests whether the treatment is likely to produce a useful clinical effect at final analysis. Treatment selection designs may be better served by confidence intervals and estimation methods, than more traditional hypothesis testing approaches. Using confidence intervals at interim gives the researcher a better idea of an interim estimate's precision, and therefore provides more information about a treatment's potential efficacy than a p-value alone allows. A researcher interested in establishing the efficacy of an intervention may wish to continue a trial showing a large variance (imprecise estimate) at interim, in the hope that the later analyses (either interim or final), which feature more participants, may reveal a more precise estimate of the true treatment effect of the intervention. Interim analyses using p-values as the stopping criteria do not address this issue.Instead, imprecision in the estimate increases the likelihood of the trial stopping. To resolve this problem, the stopping criterion of a typical interim analysis for futility could be modified to instead stop a treatment arm when the interim treatment effect confidence interval is contained entirely within a region deemed clinically unimportant – an indication that the treatment is likely to not be of benefit. Interim estimates which are imprecise (have wide confidence intervals) are protected from stopping using this rule, rather than with a hypothesis test using a p-value as the criterion. Confidence intervals produced using interim data have limitations – most obviously the fact that they will be wider (less precise) than those expected at the final analysis of the trial, given the lower sample size, which, using the proposed stopping criteria. To avoid these intervals being so inadequately wide at a low sample size that all trials continue to completion, the use of normal-based confidence intervals at a lower nominal confidence level (e.g. 90%), 'predicted intervals', which replace elements of the confidence interval calculation with assumed values, Bayesian estimation, or novel, bootstrapping-based methods, could be used instead, all of which have varying implications on the analysis. A comparison of this method is discussed, using simulated data.

### P441

#### Methods to analyse partially nested randomised controlled trials

Jane Candlish, Dawn Teare, Judith Cohen, Munyaradzi Dimairo, Laura Flight, Laura Mandefield, Stephen Walters
University of Sheffield
**Correspondence:** Jane Candlish
*Trials* 2017, **18(Suppl 1):**P441

**Background**
Individually randomised trials often have the added complication of a comparison of interventions administered in different ways, where groups of outcomes are correlated in one trial arm and not the other, termed a partially nested design. The correlation of outcomes is defined by the nature of the intervention itself, for example, a comparison of group therapy intervention and drug therapy control. Small clusters, small intracluster correlations, and differential variance between the control and intervention arms are often present in partially nested trials. If not accounted for in the design or analysis this may result in biased effect size estimates with spurious precision.
**Objective**
To evaluate statistical methods to analyse partially nested trials and provide practical advice on the analysis of partially nested trials using a simulation study, with focus on the most appropriate method for imposing clustering in the unclustered control arm.
**Methods**
Simulation studies will be used to explore varying scenarios of cluster size, number of clusters, intra-cluster correlation, and differential variance between the two trial arms and their impact on bias, power, precision and ICC estimation. In theory the mixed effect models for partially nested trials do not model clustering in the control arm, however, when fitting these models in statistical software it is necessary to impose clustering in the unclustered control arm. We will explore the various methods for imposing clustering in the control arm: a unique singleton cluster of size one for every individual; one large single cluster; or pseudo random clusters.
**Results**
Results will be presented reporting the bias, power, precision and ICC estimation using the different analysis models. The effect of the choice of imposed clustering on the intracluster correlation estimate will be presented and the most appropriate method for imposing clustering in the unclustered control arm.
**Conclusions**
Partially nested trials are commonly used in complex intervention research. The design and analysis of these trials can take account of the hierarchical data structure and needs to consider the choice of imposing clustering in the unclustered control arm.

### P442

#### The effect of small or unbalanced clusters of patients on logistic regression models in surgical trials

Alison Pullan, Neil Corrigan, Julia Brown
University of Leeds
**Correspondence:** Alison Pullan
*Trials* 2017, **18(Suppl 1):**P442

The effect of small or unbalanced clusters of patients on logistic regression models in surgical trials In surgical trials it is necessary to adjust for the clustering effect of the operating surgeon, as outcomes will be more similar for patients with the same operating surgeon than for those with a different surgeon. Due to the incremental nature of surgeon recruitment into a large surgical trial, it is likely that within a trial there will be a large number of surgeons that operate on only a few patients each, causing small cluster sizes. It is also likely that there will be a few surgeons' recruited to the trial earlier - that operate on a lot more patients than the rest of the surgeons in the trial, creating unbalanced cluster sizes. Practically, the potential effects of these small or unbalanced cluster sizes on the bias and convergence of a multi-level model can be a concern when a trial is forced to recruit many more centres or surgeons than originally planned, for example to bolster recruitment. A simulation approach was used to explore and quantify the potential risk of recruiting many additional surgeons (while keeping the sample size fixed).
A binary endpoint (success/failure) was modelled using a multi-level logistic model, treating operating surgeon as the unit of clustering. The number of patients assigned to each surgeon was simulated using a gamma distribution, which allowed the small and/or unbalanced cluster sizes described above to be simulated.
A 'surgeon effect' was included in the simulation that would increase the probability of success based on the experience, skill etc. of the surgeon. Another 'surgeon treatment effect' was included that allowed the surgeon effect to be different depending on the treatment being performed.
Patients were allocated to one of two treatments using a minimisation algorithm, stratifying for the operating surgeon.
The patients were assigned a probability of success based on their treatment. The surgeon effects were incorporated to calculate a different probability for each surgeon and treatment combination. The outcome was then generated from a binomial distribution using the calculated probability as the probability of success. Both random intercept and random slope models were investigated. The effects of changing the number of surgeons, changing the variance of the ?A3B2 show $132#?>surgeon effect and changing the variance of the surgeon treatment effect on model bias and convergence were investigated, as well as their effects on the power of the trial.
Early results suggest that unbalanced and small cluster sizes do not appear to effect the convergence of the model or cause bias in the fixed effects of the model. The effects of different cluster size distributions on the power of the study will be investigated. The consequences of changing the variance of the 'surgeon effect' and the 'surgeon treatment effect' will also be investigated as these may vary depending on the difficulty of the operation and the difference in the skill required for each operation within a trial.

### P443

#### Overview of statistical methods to monitor harms during the conduct of a randomised controlled trial

Rachel Phillips[1], Victoria Cornelius[2]
[1]King's College London; [2]Imperial College London
**Correspondence:** Rachel Phillips
*Trials* 2017, **18(Suppl 1):**P443

**Background**
Data obtained from randomised controlled trials (RCTs) contribute important information to the harm profile of a drug as they provide unbiased estimates of harm effects and provide a controlled comparison allowing causality to be evaluated.
The most common approach for harm monitoring and analysis during a RCT is to tabulate event rates by treatment arm and sometimes the difference in event rates is estimated and p-values from hypothesis tests are presented. Data are examined by an independent data monitoring committee (DMCs) who will make a recommendation to proceed or halt a trial based on these presentations. More formal assessments and integration of existing or emerging knowledge for drug harm into the DMC report is rare and as a result there is an inefficiency present when monitoring and analysing harms in trials. In the last 15 years new methods for improving the monitoring and analysis of harms in trials have been proposed. We review these methods, outline when they are appropriate to implement, examine their use in published studies and discuss challenges of their implementation.
**Results**
The review identified 11 methods for use for harm monitoring and analysis in clinical trials. We have categorised these as: sequential methods, group sequential methods and surveillance methods.
The four sequential methods have been designed to be implemented after each observed harm event. They have been developed for use in a single treatment arm setting and require a pre-specified harm of interest and a pre-specified hypotheses to be tested. Since they are implemented after each observed event they are best suited to the evaluation of serious adverse events where immediate evaluation is necessary in order to determine whether the trial should continue.
Group sequential methods primarily proposed for use in monitoring efficacy outcomes have been extended by several authors for the purpose of harm monitoring. Analogous to the methods for efficacy each require a pre-specified harm of interest with a pre-specified hypothesis to be tested.
Four surveillance methods have been developed for multi-arm studies with the purpose of monitoring emerging harm events i.e. The harm is not pre-specified. The applications of these methods to date have been applied at body system level rather than reported adverse event level.
In a review we undertook to examine the methods and reporting of harms in rcts we found none of the 189 included trials used any of these methods.
**Conclusions**
Statistical methods have been proposed for use in a clinical trials setting to flag signals for adverse drug reactions for both pre-specified harm events and for emerging harm events. However the clinical trials community are not currently implementing these harm monitoring methods and tabulations of adverse events remains the most popular choice to evaluate disproportionalities between treatment arms. The reasons for this are unclear but could be due to: their relative infancy; sophisticated methodology; the computational intensity and increased resource level needed; and no formal requirement from regulatory bodies and the wider clinical community for more robust methods.

### P444

#### Quantifying effect sizes in clinical trials

Joanne Rothwell, Steven A Julious, Cindy Cooper
University of Sheffield
**Correspondence:** Joanne Rothwell
*Trials* 2017, **18(Suppl 1):**P444

**Background**
The target difference, or 'effect size', is an important component of a sample size calculation as the calculations are extremely sensitive to assumptions as to what the effect size will be. A review of 117 NIHR Health Technology Assessment funded randomised controlled trials indicated that over 50% of randomised controlled trials report that they have used a previous study or previous research to estimate their target effect size.
**Objective**
The objective of this presentation is to examine the issues that can arise when designing one trial based on the results of a previous trial, or previous research using a simulation study. When basing one study on the results of another, there is a bias which is introduced called regression to the mean. This bias means that there would be an over-estimation of the effect size, and the effect size observed in the second trial is likely to be considerably less than that which the study was powered on.
**Methods**
Simulations were performed to quantify the impact of using previously observed responses to design future studies. The inputs used in the simulations were based on the findings of the review. The simulations were completed under the context of having one trial based on the results of another. Various end-points were used to build from the simplest case to cases where biomarkers or surrogate end-points were used. The simulation results will be used to inform a mathematical solution using a truncated Normal distribution. This mathematical solution will provide an adjustment which can be used to better estimate sample sizes when using previous results. The results will be extended to different powers and significance levels.

**Results**

Using effect sizes previously observed to design a new study can lead to an over estimation of the treatment effect. This over estimation could be as much as 15% which, if not allowed for, could lead to studies with sample sizes that are too small and therefore are underpowered. Designing a trial dependent on the results of a first trial impacts on the distribution of plausible responses for this initial trial and leads to bias in effect size estimation. Methods will be presented that allow for this over estimation. The level of adjustment will depend on factors such as the statistical power of the first study or the p-value of a meta-analysis to combine previous studies.

**Conclusions**

When designing a clinical trial which is dependent on the results of a first trial, the effect size used will be overestimated and so as a result the sample size will be too small. The effect size should be adjusted to account for the sequential nature of the trials being investigated.

### P445

#### Bayesian prediction of the intra-cluster correlation for sample size calculation of cluster randomised trials

Chris Newby, Sandra Eldridge
Quenn Mary, University of London
**Correspondence:** Chris Newby
*Trials* 2017, **18(Suppl 1):**P445

**Background**

The intra-cluster correlation coefficient (ICC) is a statistic that is used to describe the variation between and within clusters. A trial ICC can be calculated from a pilot study but when calculated has a large confident interval. Alternatively we can select an ICC for a trial from previous trials ICC's that have similar cluster type and outcome.

**Aim**

We aim to collect data available on ICC's from previous trials to create a prior distribution of ICC's and then combine these with the data from pilot studies in a Bayesian analysis.

**Methods**

As an example we use simulated data of 10 clusters with 200 patients in each cluster with an ICC of 0.05 with four priors for the ICC. A non-informative prior and three informative priors, one based on 56 studies for continuous outcomes of GP surgeries from University of Aberdeen, one based on QMUL's cluster randomised course containing 150 ICC's and a prior based on 10 studies specific to asthma questionnaire data from GP surgeries.

**Results**

The ICC was calculated from Bayesian software winbugs and returned to R. The mean and credible interval for ICC were calculated from the posterior distribution. The different methods of ICC calculation along with their means and confidence/credible intervals are summarised and compared.

**Discussion and Conclusion**

Bayesian methods of calculating the ICC are similar to frequentist methods when a non-informative prior is used. If a more informative prior is used based on existing trials we can reduce the credible interval for the ICC in order to better inform sample size calculations and sensitivity analysis of sample size calculations. More disease specific trial ICC's need to be found to create more prior distributions for specific disease outcomes.

### P446

#### Current trends in data monitoring committees

Kent Koprowicz, David R. Kerr
Axio Research
**Correspondence:** Kent Koprowicz
*Trials* 2017, **18(Suppl 1):**P446

Axio Research has served as an independent statistical group serving Data Monitoring Committees (DMCs) for industry and government clinical trials in pharmaceuticals and devices for over two decades. Accepted, best, and most common practices have changed greatly over this period. The practice of DMCs continues to evolve and emerging trends will be discussed. Current trends include: program-wide DMCs, teleconference and web meetings, reduced sponsor and DMC interaction, focused DMC recommendation delivery, electronic reporting, DMCs for more studies (early phase, single-arm, open-label), expertise of DMC members. Pros and cons, implementation, and what to expect in the near future will be covered from the perspective of an independent statistician with guidance for both sponsors and DMC members.

### P447

#### Use of instrumental variables within randomised controlled trials

Katie Pike[1], Chris A. Rogers[1], Gavin J. Murphy[2], Massimo Caputo[3], Alasdair MacGowan[4], Barnaby C. Reeves[1]
[1]Clinical Trials and Evaluation Unit, University of Bristol; [2]University of Leicester; [3]Bristol Royal Hospital for Children; [4]Southmead Hospital
**Correspondence:** Katie Pike
*Trials* 2017, **18(Suppl 1):**P447

**Background**

Randomised controlled trials (RCTs) are widely considered the gold standard study design for quantifying the effect of an intervention, due to the minimal risk of bias from confounding. Some RCTs are designed whereby subjects are randomised to different strategies, for example differing criteria for red blood cell (RBC) transfusions to be given, rather than specifically to an intervention or control treatment. In such studies the groups should differ substantially overall in terms of the intervention received (e.g. The average number of RBC units transfused), but within each of the randomised groups there will be heterogeneity in the intervention received. Such situations give an opportunity to estimate across the RCT as a whole the effect of differing amounts of intervention (i.e. An observational analysis within the RCT). The latter can be estimated using instrumental variable (IV) techniques with randomised allocation as an instrument, avoiding the problem of confounding (measured or unmeasured) that is often a concern in observational analyses.

**Methods**

We have used this approach in three RCTs. In the titre2 trial a liberal RBC transfusion strategy after cardiac surgery was compared with a more restrictive strategy, creating two groups with different risks of transfusion and distributions of numbers of RBC units transfused. The Thermic trials compared paediatric cardiac surgery performed at warmer (normothermic) vs colder (hypothermic) temperatures, generating groups with different average surgery temperatures. Finally, the RAPIDO trial compared a rapid diagnostic pathway with the conventional method for patients with blood stream infections, with the resultant groups differing substantially in terms of the time until microbiological information is returned from the laboratory.

In addition to the primary intention-to-treat (ITT) analyses an IV analysis was performed for each trial, with randomised allocation as an instrument. Such models estimate different effects to the ITT analyses, namely: each RBC unit transfused on severe post-operative complication (titre2); each degree Celsius on intubation duration (Thermic); each hour in the time to provision of microbiological information on mortality (RAPIDO). Models were fitted in Stata. For titre2 and RAPIDO IV poisson models for binary outcomes were used. For Thermic an IV linear regression model was used.

**Results**

For titre2 the ITT estimate of the odds ratio for allocation (liberal vs restrictive) on post-operative severe complications was 0.87, 95% confidence interval (CI) (0.72-1.05). The IV estimate of the relative risk of each unit transfused on outcome was 0.89, 95% CI (0.75-1.06). In the Thermic trials the geometric mean ratio (GMR) from the ITT analysis of the effect of allocation (normothermic vs hypothermic) on intubation duration was 0.77, 95% CI (0.57-1.04). The IV estimate of the GMR of each degree Celsius was 0.95 (0.89, 1.02). Results from the RAPIDO trial are forthcoming.

**Discussion**

Although the ITT and IV models are estimating different effects we anticipated that the direction of effects would be consistent, which was the case in the examples we considered. The use of IV techniques to address secondary objectives in RCTs can be a useful tool in certain settings, although such models are generally low powered.

### P448

#### Methods for testing for and handling non-proportional hazards in a phase II rct in chronic lymphocytic leukaemia

Lucy McParland, Dena R. Howard
University of Leeds
**Correspondence:** Lucy McParland
*Trials* 2017, **18(Suppl 1):**P448

Cox's proportional hazards regression modelling is a common method for analysing time-to-event data in clinical trials, and provides an estimate of the hazard ratio (HR) as a measure of the overall treatment effect. This semi-parametric model relies on the assumption that the hazard ratio remains constant over time, such that the hazards between the treatment groups are proportional. If this assumption is violated, the Cox's proportional hazards model can lead to a reduction in power for the corresponding tests of significance and more crucially, imprecise and misleading estimates of the treatment effect.

ADMIRE is a two-arm Phase II randomised-controlled trial, in 215 patients with Chronic Lymphocytic Leukaemia (CLL). Progression-free survival (PFS) was one of the key secondary endpoints, the intention for analysis was via a multivariable Cox regression model and presentation of the Kaplan-Meier survival estimates. On analysis of PFS, there was strong evidence that the proportional hazards (PH) assumption did not hold, as indicated by the crossing of the survival curves, putting into question the reliability of the estimate of the HR in the Cox regression model.

I will present common methods for testing for non-proportional hazards in the analysis of survival data. I will then present an alternative method for estimating the treatment effect when the proportional hazards assumption is violated known as restricted mean survival time (RMST).

RMST provides a way of estimating the treatment effect when the PH assumption is in doubt or has clearly been violated as recommended by Royston and Parmar (2011). It is a measure of average survival from time 0 up to a restricted pre-specified time t, and can be estimated as the area under the survival curve using a pseudovalue approach. The difference in RMST between treatment groups can be calculated using standard regression methods and provide an appropriate estimate of the treatment effect, when non-proportional hazards exist.

The results of the RMST method when applied to analyse the PFS data in ADMIRE will be presented and compared to the results from the Cox proportional hazard model, which is inappropriately applied when the proportional hazards assumption fails to hold.

### P449

#### Use of longitudinal data in the analysis of biomarkers: lessons from simulation and reality

Francesca Fiorentino[1], Chris A. Rogers[2], Gianni Davide Angelini[2], Shahrul Mt-Isa[1], Barnaby C. Reeves[2]
[1]Imperial College London; [2]Bristol University
**Correspondence:** Francesca Fiorentino
*Trials* 2017, **18(Suppl 1):**P449

**Background**

Repeated post-randomization longitudinal measurements are often not used to maximum efficiency at the analysis stage, with baseline data being disregarded or used simply to derive a single 'change from baseline' measurement. We have previously presented a simulation model comparing different statistical methods of dealing with repeated biomarker measurements over time. This abstract extends that work to consider how the relative precision of the different methods are affected in different biomarker scenarios, both by simulation and using real data. Biomarkers can represent a physiological state (S) at any time (e.g. Reflecting a comorbidity such as chronic kidney dysfunction) or only reach measurable levels after an event (E; e.g. Organ-specific response to injury). Our previous work only considered the former scenario, using a simulation model. Here, we use data for creatinine and myocardial troponin from a trial to illustrate the two scenarios. Using the simulation model, or bootstrapped estimates for the trial data, we quantify how the relative precision of different methods for analysing repeated longitudinal measurements is affected, when: (a) adjusting for a baseline measurement or not, and (b) when varying amounts of data are missing-at-random.

**Methods**

We are using the simulation model to generate biomarker data of the S and E type. Four different analysis methods are being used to analyse the simulated biomarker data and estimate the relative precision of each model: t-test of the maximum change from baseline; area under the curve; multiple comparisons with Bonferroni correction; and a multilevel model. We are boot-strapping data on creatinine (type S biomarker) and troponin (type E biomarker) collected in a randomised controlled trial to mirror the simulated scenarios with real data. We are also investigating the impact on relative precision of removing varying amounts of data (5%, 10% and 20%) at random, since repeated biomarker measures are often incomplete.

**Results**

This work is ongoing. We know from our previous work that the multilevel model has the best precision compared to the other methods. What is unknown is whether the relative precision of the methods varies, and if so by how much, in these different scenarios. Results will be presented at the conference.

**Conclusions**

Awareness of the greater precision afforded by modern statistical methods of analysis is limited, leading to inefficiencies in translating discovery science into clinical settings. This research will highlight to researchers and funders the extent of the inefficiency and how practical constraints in doing the research, such as completeness of data, modify the penalty of using old-fashioned methods of analysis.

### P450

#### Robust methods for improving power in group sequential randomized trial designs, by leveraging prognostic baseline variables and short-term outcomes

Tianchen Qian, Michael Rosenblum, Huitong Qiu
Johns Hopkins University
**Correspondence:** Tianchen Qian
*Trials* 2017, **18(Suppl 1):**P450

In group sequential designs, adjusting for baseline variables and short-term outcomes can lead to increased power and reduced sample size. We derive simple formulas for the efficiency gain from such variable adjustment using semiparametric estimators. The formulas reveal the impact of the prognostic value in the variables and how the impact is modified by the proportion of pipeline participants, analysis timing, and enrollment rate. While strongly prognostic baseline variables are always valuable to adjust for, the added value from prognostic short-term outcomes is limited. For example, if at least 2/3 of the enrollees have primary outcome observed, the equivalent sample size reduction from prognostic short-term outcomes is at most half of the reduction from an equally prognostic baseline variable. The added value from prognostic short-term outcomes is generally smallest at later interim analyses which are the ones that tend to impact power the most. A practical implication is that in trial planning one should put priority on identifying prognostic baseline variables. Our results are corroborated by simulation studies based on data from a real trial, using the class of readily implemented semiparametric estimators.

### P452

#### Analysis of an ordinal endpoint for use in evaluating treatments for severe influenza requiring hospitalization

Ross Peterson[1], David M. Vock[1], John H. Powers III[2], Sean Emery[3], Eduardo Fernández-Cruz[4], Sally Hunsberger[5], Mamta K. Jain[6], Sarah Pett[7], James D. Neaton[1]
[1]University of Minnesota, School of Public Health, Division of Biostatistics; [2]George Washington University School of Medicine; [3]Kirby Institute, University of New South Wales; [4]Hospital General Universitario Gregorio Marañón, Instituto de Investigación Sanitaria Gregorio Marañón, Departamento de Microbiología I/Inmunología, Facultad de Medicina, Universidad Complutense de Madrid; [5]National Institute of Allergy and Infectious Disease, Biostatistics Research Branch; [6]UT Southwestern Medical Center, Department of Internal Medicine; [7]CRG, Infection and Population Health, UCL and MRC CTU at UCL, University College London
**Correspondence:** Ross Peterson
*Trials* 2017, **18(Suppl 1):**P452

**Background**
A single best endpoint for evaluating treatments of severe influenza requiring hospitalization has not been identified. A novel 6-category ordinal endpoint of patient status is being used in a randomized controlled trial (FLU-IVIG) of intravenous immunoglobulin (IVIG). We systematically examine four factors regarding the use of this ordinal endpoint that may affect power from fitting a proportional odds model: 1) deviations from the proportional odds assumption which result in the same overall treatment effect as specified in the FLU-IVIG trial protocol and which result in a diminished overall treatment effect; 2) deviations from the distribution of the placebo group that researchers assumed in the FLU-IVIG trial protocol; 3) the effect of patient misclassification among the 6 categories; and 4) the number of categories of the ordinal endpoint. We also consider interacting the treatment effect (i.e., Factor 1) with each other factor.
**Methods**
We conducted a Monte Carlo simulation study to assess the effect of each factor. To study factor 1, we developed an algorithm for deriving distributions of the IVIG group that deviated from proportional odds while maintaining the same overall treatment effect in the form of an average log odds ratio. To construct the algorithm, we know that for large samples the average log odds ratio of a misspecified model is the value for which the expected score function equals zero. Given information about the trial, our algorithm constrains the distribution of the IVIG group to maintain the average log odds ratio across deviations from proportional odds. Our algorithm can handle ordinal endpoints with any number of levels. For factor 2, we considered placebo group distributions which were more or less skewed than the one specified in the FLU-IVIG trial protocol by adding or subtracting a constant from the cumulative log odds ratios. To assess factor 3, we added misclassification between adjacent pairs of categories that depend on subjective patient/clinician assessments. For factor 4, we collapsed some categories into single categories.
**Results**
Deviations from proportional odds reduced power at most from 80% to 77% given the same overall treatment effect as specified in the FLU-IVIG trial protocol. Misclassification and collapsing categories can reduce power by over 40 and 10 percentage points, respectively, when they affect categories with many patients and a discernible treatment effect. But, collapsing categories that contain no treatment effect can raise power by over 20 percentage points. Differences in the distribution of the placebo group can raise power by over 20 percentage points or reduce power by over 40 percentage points depending on how patients are shifted to portions of the ordinal endpoint with a large treatment effect.
**Conclusions**
Provided that the overall treatment effect is maintained, deviations from proportional odds marginally reduce power. However, deviations from proportional odds can modify the effect of misclassification, the number of categories, and the distribution of the placebo group on power. In general, adjacent pairs of categories with many patients should be kept separate to help ensure that power is maintained at the pre-specified level.

### P453

#### The design and analysis of early phase ii trials with naturally bounded continuous fractional outcomes

Paul Silcocks, Richard Jackson
University of Liverpool
**Correspondence:** Paul Silcocks
*Trials* 2017, **18(Suppl 1):**P453

**Introduction**
We suggest that use of more appropriate statistical methods will improve interpretability and inferences for early phase II trials that use continuous fractional outcomes. Typically such trials are designed and analysed based on transformed data (e.g. Log transformation), with sample size calculations based on standardised effect sizes and results summarised using less familiar measures such as geometric means. We illustrate these issues in terms of Ki67, a common measure of tumour response in early breast cancer studies.
**Background**
Guidelines on assessment of Ki67 scores in breast cancer (Dowsett et al., 2011) give advice on the role of Ki67 in clinical management and methodological issues for its measurement, but neglect methods for statistical analysis. Ki67 scores are expressed as a percentage and hence restricted to the range 0–100. Despite the natural bounds of the data, recommendations propose Ki67 be analysed assuming a log-normal distribution.
**Methods**
We illustrate with both real and simulated datasets that the use of log transformed data when the data naturally bounded is not always appropriate. Particularly in randomised studies, it is often the case that a log transformation may be suitable for one, but not both arms of the study. Further, interpretations of the data are typically dependent on differences between means and may ignore changes in variation.
We show how beta regression and fractional logistic/probit modelling directly relate to the original (untransformed) scale can account for shifts in both location and spread. We also provide suggestions on sample size estimation.
**Conclusions**
Analysis of phase II trials that use continuous fractional outcomes should reflect the underlying nature of the data recorded. We hope to have increased researchers' awareness of better methodology that will enhance comparative analysis, and to provide suggestions for statistical colleagues who may be asked to perform such work.
We would also encourage researchers to provide summary data such as distributional shape, mean and variance along with their main results. Where a transformation is used, justification for choice and fit of chosen transformation should be provided.

### P454

#### An investigation of the factors which influence children with asthma having unscheduled medical contacts around the start of the new school year in England and Wales: a mixed methods study

Rebecca Simpson, Steven A. Julious, Wendy O. Baird
University of Sheffield, UK
**Correspondence:** Rebecca Simpson
*Trials* 2017, **18(Suppl 1):**P454

Evidence shows that there is an increase in the number of unscheduled medical contacts amongst school-aged children with asthma at the beginning of the school year (September). It has been suggested that this is caused by a viral challenge influenced by the return to school. It is hypothesised that this challenge is exacerbated as some children may stop taking their medication over the summer holiday. The aim of this research is to identify factors that can be used to predict which children are more likely to have an unscheduled medical contact in September.

A mixed methods approach is being used to investigate the factors that affect children having unscheduled medical contacts at the beginning of a new school year. The quantitative data comes from the PLEASANT (Preventing and Lessening Exacerbations of Asthma in School-age children Associated with a New Term) cluster intervention study. PLEASANT investigates whether a simple letter intervention reminding children to take their asthma medication during the summer holidays reduces unscheduled contacts. The quantitative component includes daily data over a two year period from approximately 12,000 children aged 5–16 with asthma. The qualitative data comes from a study which will be done in two stages, before and after the summer holidays. This qualitative research will explore why children may not take their medication and what factors the children think trigger their asthma symptoms. The first stage of the study will be used to inform the quantitative data analysis and the second stage will be used to validate the results. The two stages will also be used to investigate any differences from the children's responses before and after the summer holidays.

The first stage of the qualitative study was conducted in June/July 2016 and the second stage will have been conducted in Oct/Nov 2016. In the first stage there were 17 interviews with children aged 5–14, with a mixture of boys and girls.

The information collected from the qualitative studies will be used to identify any possible subgroups that could be incorporated into the quantitative analysis. Subsequently, a quantitative analysis will be performed to identify the subgroups for which the PLEASANT intervention could have been most effective.

This is one of the first studies using a mixed method design with children that have asthma. The findings can be used to propose a possible intervention that can be targeted at those who are most likely to have an unscheduled medical contact in September.

### P455

#### Using off-treatment data to estimate the de facto estimand in a randomised trial

Joseph Royes [1], Juan J. Abellan[2], Ian R. White[1], Dawn Edwards[2], Oliver Keene[2], Nicky Best[2]
[1]MRC Biostatistics Unit; [2]Glaxosmithkline
**Correspondence:** Joseph Royes
*Trials* 2017, **18(Suppl 1):**P455

##### Background
The FDA suggests that participants who discontinue or otherwise deviate from randomised treatment should continue to be followed up in order to facilitate the estimation of the de facto treatment effect in superiority trials.

##### Objective
We set out to explore how to perform the analysis where data collection is continued in some, but not all, patients after discontinuation of randomised treatment: we call this off-treatment data. The work was motivated by the problem of writing a statistical analysis plan for a pharmaceutical trial.

##### Methods
We consider several alternative multiple imputation methods that can be used. The methods vary in their use of earlier outcomes and treatment discontinuation time in the mean part and in their use of treatment discontinuation in the variance part. Different methods make different assumptions about the missing data, specifically about what observed data to condition on in order to justify a missing at random (MAR) assumption, and whether or not treatment discontinuation is considered to represent a treatment failure outcome; they also make different demands on the observed data. We explore the performance of the methods in a simulation study, aiming to quantify the impact of different MAR assumptions and different variance assumptions.

##### Results
The proposed imputation methods are shown to be valid when treatment discontinuation is not at random, provided that subsequent loss to follow-up after treatment discontinuation (i.e. Failure to provide off-treatment data) is at random. We show that the loss of performance due to making simpler assumptions when only the more complex assumptions are true must be balanced against the gain of performance due to making simpler assumptions when they are true. Optimal choice of model depends on likely assumptions and on the number of treatment discontinuations.

##### Conclusions
The proposed methods provide a framework for choosing a suitable imputation model in this setting, and the simulation results were used to support the choice of sensitivity analysis methods included in the statistical analysis plan for the pharmaceutical trial.

### P456

#### The SALVO study: a retrospective take on an interim analysis of futility in a randomised trial

Lee Beresford[1], Richard Hooper[2], Khalid S. Khan[3], Philip Moore[4], Matthew Wilson[5], Shubha Allard[6], Ian Wrench[7], Jane P. Daniels[8], Matthew Hogg[9], Doris Lanz[3]
[1]Queen Mary University of London; [2]Pragmatic Clinical Trials Unit, Queen Mary University of London; [3]Women's Health Research Unit, Barts and the London School of Medicine and Dentistry, Queen Mary University of London; [4]Birmingham Women's Hospital; [5]School of Health and Related Research (scharr), University of Sheffield; [6]NHS Blood and Transplant; [7]Sheffield Teaching Hospitals NHS Foundation Trust; [8]Birmingham Clinical Trials Unit, University of Birmingham; [9]Royal London Hospital, Barts Health NHS Trust
**Correspondence:** Lee Beresford
*Trials* 2017, **18(Suppl 1):**P456

Recruitment in clinical trials can often be problematic and marred by unforeseen circumstances. This often leads to requests from trial teams to the funders for extensions to their recruitment period, so that planned sample sizes can be reached. While numerous factors will play a role in deciding the future of an under-recruiting trial, futility analyses are a method sometimes used to assess whether there is hope for a significant result in a trial, should recruitment be allowed to continue. A funder may ask investigators to conduct such an analysis to determine whether an extension should be granted.

This was the case for the SALVO trial - an evaluation of the effect of intra-operative cell salvage during caesarean section on the need for donor blood transfusion. The funding body requested that the trial team conduct an analysis to assess the probability of obtaining a statistically significant result at the end of the study, given the data collected by that time. We proposed an approach to the futility analysis based on stochastic curtailment and predictive power, with the idea to evaluate the conditional power i.e. The probability of obtaining a statistically significant result at the end of the trial, given the data that had already been collected. There is no absolute cut-off for conditional power in deciding whether to continue a trial; instead it must be considered alongside other factors.

We also sought advice from the independent Data Monitoring Committee (DMC) for the trial, and sent them results from our futility analysis, generated by an independent statistician. In open correspondence the

DMC raised questions about the need for a futility analysis, and following a closed meeting they recommended that the funder extend the study recruitment period. The SALVO trial recruited to completion after a 13 month extension was granted.
We present the methods used and results of the futility analysis that was conducted, as well as final results of the primary analysis and other findings of the study for comparison. We discuss interpretations that could have been drawn from the futility analysis and provide a discussion of the pros and cons of conducting futility analysis with the help of hindsight and with particular reference to the events which occurred in the SALVO trial.

### P457

#### Single agent trastuzumab or lapatinib to treat her2-overexpressing breast cancer: combining past and current evidence in a Bayesian reanalysis

Giuseppe de Vito[1], Ileana Baldi[2], Annamaria Nuzzo[3], Filippo Montemurro[3], Paola Berchialla[4]
[1]European Laboratory for Non-Linear Spectroscopy; [2]Unit of Biostatistics, Epidemiology and Public Health, Department of Cardiac, Thoracic and Vascular Sciences, University of Padova); [3]Department of Investigative Clinical Oncology, Fondazione del Piemonte per l'Oncologia, Candiolo Cancer Institute; [4]Department of Clinical and Biological Sciences, University of Torino
**Correspondence:** Giuseppe de Vito
*Trials* 2017, **18(Suppl 1):**P457

**Introduction**
Recent studies investigated the possible role of Human Epidermal Growth Factor Receptor 2 (HER2)-targeting compounds as first-line, single-agent therapy for HER2-over-expressing Breast Cancer (BC) with promising results. In particular, for a subgroup of patients the observed disease control duration was similar to that reported for the commonly-used anti-HER2 and chemotherapy combination treatment. In order to gather further insights about the biomarkers that characterise the patients that can benefit from anti-HER2 single-agent therapy and to evaluate the efficacy of this therapy in patients not previously treated for HER2-positive metastatic BC, two clinical trials were initiated: HERLAP I and HERLAP II, both testing two anti-HER2 agents: trastuzumab and lapatinib. However HERLAP I was prematurely terminated, also due to the slow accrual of patients.
**Objective**
We devised to measure the Progression Free Survival (PFS) for patients in single-agent therapy from the HERLAP trial data, in order to compare it to the combination treatment. However, the small sample size makes it difficult to apply frequentist statistical approaches and calls for an integration of the information derived from the two trials. In this regard, the Guidance for the Use of Bayesian Statistics in Medical Device Clinical Trials, issued by the Food and Drug Administration, states the opportunity to use a Bayesian approach to combine prior information with new observations, suggesting to base this information on empirical evidences. Using this approach, we generated prior distributions from the data of the early-stopped HERLAP I trial, devising to use them in the analysis of the HERLAP II trial results.
**Methods**
We planned to employ a hierarchical Bayesian Weibull survival model to characterise both the 'Biological PFS' (i.e. Taking in consideration only the period of exclusive administration of anti-HER2 agents) and the 'Total PFS' (regardless of protocol failures). In particular, using non-informative prior distributions, we derived posterior distributions for the parameters of the Weibull model based on the HERLAP I data, and we have planned to use them in turns as prior distributions to derive the posterior distributions for the parameters based on the HERLAP II data, thus 'borrowing strength' from the first trial to the second.
**Results**
After describing the statistical method in details and presenting the data, in this contribution we shall discuss preliminary results that we obtained by deriving the posterior distributions from the HERLAP I data. In particular, we observed that the median survival times in days (and the extremes of their 95% credible intervals) for the biological PFS and the total PSF are 190 (96; 355) and 333 (172; 672), respectively. If we take into account only the trastuzumab-treated patients, then these values become 335 (139; 893) and 442 (162; 1304); whereas considering only the lapatinib-treated patients they become 99 (46; 232) and 250 (102; 805). It is interesting to note that these survival times are similar to those reported for the combination treatment.
**Conclusions**
These data, albeit very preliminary, represent an additional suggestion for the efficacy of the single-agent therapy for HER2-positive metastatic BC.

### P458

#### A validation and calibration process on self-reported tobacco with participants: Cotinine levels in building blocks

Chao Huang
Cardiff University
*Trials* 2017, **18(Suppl 1):**P458

**Background**
Building Blocks was a pragmatic randomised controlled trial assessing the effectiveness of giving the Family Nurse Partnership (FNP) home-visiting programme to teenage first-time mothers on infant and maternal outcomes up to 24 months after birth (Robling et al., 2016). One of the primary outcomes was to investigate the effectiveness of the intervention in reducing smoking during pregnancy. At baseline and late pregnancy, we collected a large amount of self-reported data on smoking habits from each participant during a face-to-face and telephone interview respectively. It is well known that self-reported smoking can be inaccurate and therefore some participants are likely to report smoking fewer or more cigarettes than they actually do. In the Building Blocks study, we collected urine samples at the same time as the baseline interview and at follow-up in late pregnancy. The cotinine levels within the urine sample were used to supplement the participants' self-reported behaviour and then further calibrate their number of cigarettes smoked per day (Dukic et al., 2007). However, this calibration approach requires complete and well-synchronized collection of self-reports and urine samples. The main challenge of our study lies in the collection of urine samples, particularly at follow-up stage. Some urine samples were collected at different time points from their interview and some were missing for a variety of reasons, which cause incompleteness in participant's data and potentially lead to bias in the results.
**Methods**
We tackled these issues using a validation and calibration process. Firstly, participants were divided into three categories according to their completeness of these outcomes. Secondly, time gaps between the urine sample and self-report dates were assessed over different thresholds. Thirdly, we examined the feasibility of inferring participants' reporting behaviours at follow-up stage by their baseline outcomes.
**Results**
870 participants with different levels of non-contemporary outcomes collection at follow-up stage were sub-grouped and investigated over their consistency in reporting behaviours. We further validated 222 participants with incomplete data at follow-up stage and calibrated their self-reported tobacco accordingly, which strengthened the power for the main analysis.
**Discussion**
It is not rare that difficulties arises when collecting data at follow-up stages, especially in populations that may be vulnerable and often mobile as in this study. Rather than losing those participants for key analyses, this proposed process could further validate and calibrate self-reported tobacco of participants for public health studies with similar settings. Because of the costings and challenges in urine sample collections, investigating the participants' reporting behaviours by some associated factors, such as social and demographic factors, has become one of our follow-up research topics.

### P459

#### Integrating continuous stratification variables into a dynamic adaptive randomisation algorithm

Nia Goulden, Zoe Hoare
Bangor University
**Correspondence:** Nia Goulden
*Trials* 2017, **18(Suppl 1):**P459

##### Background

Stratification variables are confounding variables which could potentially influence the outcomes being measured within a trial. The aim of this work is to extend a dynamic adaptive randomisation algorithm to be able to accept continuous stratification variables, such as age. Many randomisation algorithms categorise such variables, however specifying a measure of imbalance with an aim to minimise imbalance should improve the sensitivity of stratification schemes. From the literature two methods have been tested to integrate into the algorithm published previously by the North Wales Organisation for Randomised Trials in Health (NWORTH), Bangor University (Russell, Hoare, Whitaker, Whitaker, & Russell, 2011).

##### Method

Method Firstly we test a method that utilises the rank information of the covariates (Hu & Hu, 2012). Using a computationally efficient search the method finds the maximum possible difference resulting from assigning a new participant. Secondly we test a method that minimises the Kullback-Leibler divergence (Endo, Nagatani, Hamada, & Yoshimura, 2006). This method is based on probabilities of assigning a new participant to a group and therefore needed to be adapted in order to be integrated. A trial of 332 participants was simulated, using centre (6 centres recruiting 72, 66, 66, 62, 34, and 32 participants, respectively) and age (continuous 18–65 inclusive) as stratification variables. Comparisons of the methods were based on the resulting differences in means of the variable in two groups, results of t-tests and f-tests of the final allocations, sequence length and the imbalance.

##### Results

Results are displayed in for parameters total = 0.5, centre = 0.5, age = 0.5 and stratum = 0.5, which are set to control the amount of imbalance allowed for each variable. Reducing these parameters lessens the control on imbalance while increasing them will increase predictability. We have also tested different parameters to assess the effect of increasing and decreasing the stratum and strata. Increasing the weight for age decreases the difference between means overall, but increases the difference between means within centre for method 1, because the imbalance within the strata are not as well controlled. The 1st percentile for the t and f tests increase for both methods. Increasing the weight for the strata decreases the difference between means overall for both methods and within centre for method 1. Method 1 requires searching the randomised data so it does take longer to compute the result than for method 2. Despite this method 1 can still produce a randomisation result in a few seconds, even with 300 participants randomised.

##### Conclusions

Both methods produce similar acceptably balanced results however method 1 has been chosen as the best option to integrate into the current algorithm. Method 1 directly produces a measure of imbalance which is more easily integrated, whereas method 2 needed to be adapted to allow integration. In summary, inclusion of continuous stratification variables in randomisation schemes without the need to categorise allows more sensitivity to the variable and has indirect impact on the analysis. We advocate the use of stratification variables within models of analysis, if continuous then these should be utilised as such.

### P460

#### How accurately do trialists pre-specify sample sizes for test evaluation trials? The experience in NIHR funded trials in the HTA and EME programmes

Jonathan Deeks, Lucinda Archer, Kelly Handley, Catherine Hewitt, Natalie Marchevsky, Samir Mehta, Laura Quinn, Alice Sitch, Yongzhong Sun, Yemisi Takwoingi
University of Birmingham
**Correspondence:** Jonathan Deeks
*Trials* 2017, **18(Suppl 1):**P460

##### Background

Trials of tests may evaluate their role as screening, diagnostic, staging, monitoring or prognostic tests. The National Institute of Health Research (NIHR) programme has over 20 years experience in commissioning trials of tests for these clinical roles through the Health Technology Assessment (HTA) and Efficacy and Mechanism Evaluation (EME) Programmes. Trialists often struggle to identify appropriate methods for computing sample size for test evaluation trials, and there is often little data available to inform the assumptions made in sample size calculations.

##### Objective

To review the methods used for sample size calculation for trials of tests and assess the evidence base for the assumptions made in the original sample size calculation and assess their validity in comparison with the experience of the trial.

##### Method

Final study reports, published protocols, and (where available) grant applications from the NIHR HTA and NIHR EME programmes for trials evaluating tests were identified. The theoretical approach used for computation of sample size was identified and classified according to (i) the study outcome to which it related, (ii) whether it was based on consideration of statistical power to test a hypothesis or precision to estimate a parameter, and (iii) whether it was judged an appropriate method to compute sample size in comparison with the established literature. Estimates of key parameters describing the baseline scenario (such as disease prevalence and progression, the performance of comparative tests, the correlation between tests) were identified from the protocol and their sources identified. Assumed values for key parameters in each sample size calculation were compared with the estimates observed in the trials. Details of any sample size revisions undertaken during the study were identified and reported. All assessments were initially undertaken independently in duplicate and consensus reached through team discussion.

##### Results

62 reports of test evaluation studies were identified from the NIHR HTA and NIHR EME published monographs. Their evaluation is currently ongoing, and we will report on the aspects detailed above. We are considering whether it is possible to predict particular scenarios in which sample size estimates are most challenging and least likely to be valid.

##### Discussion

We will discuss the challenges that researchers across the NIHR programmes have faced in identifying methods and computing sample size calculations for test evaluation studies, and assess the importance of considering planning sample size revision processes in test evaluation studies.

### P461

#### S0819: lessons learned from conduct of a cooperative group phase III trial with a biomarker defined subset co-primary objective

James Moon, Mary Redman
SWOG Statistical Center
**Correspondence:** James Moon
*Trials* 2017, **18(Suppl 1):**P461

**Background**
SWOG S0819 is a phase III trial evaluating both the value of cetuximab in the treatment of advanced non-small cell lung cancer (NSCLC) and epidermal growth factor receptor (EGFR), as measured by FISH, as a predictive biomarker for cetuximab efficacy in NSCLC. The design of the study incorporated co-primary objectives to assess cetuximab in both the overall study population and among EGFR FISH-positive (FISH+) patients. Activated July 15, 2009, it was one of the first trials in SWOG requiring tissue to evaluate a primary objective in a biomarker-defined population. We will outline how methods for obtaining adequate tissue, and monitoring results from the FISH assay in comparison with design assumptions impacted the conduct of the study.
**Methods**
All patients were required to submit a paraffin-embedded tissue block or at least 10 unstained slides. In addition to the EGFR FISH assay, if additional tissue remained, secondary objectives included an investigation of the efficacy of cetuximab in patents whose tumor expressed EGFR by immunohistochemistry (IHC) and in patients whose tumor harbored a KRAS mutation, with priority given to IHC if tissue was limited. The FISH assay was performed at University of Colorado and results reported to the SWOG stat center on a monthly basis. IHC was performed at University of Colorado and KRAS performed at UC Davis.
**Results**
A total of 1333 patients were registered to S0819. Usable tissue specimens were obtained from 1208 patients, of which, 1046 were adequate for FISH. Of these, 406 were FISH+. Comparisons between the study design assumptions and the observed proportions were monitored on a monthly basis. The proportion of FISH+ patients was lower than anticipated as was the assay success rate. This monitoring resulted in the following interventions in the study conduct: efforts to improve these numbers included development of a form that required the local pathologist to review and confirm that the tissue contained at least 100 tumor cells prior to submission. An automated email notification system to prompt sites for additional tissue if their initial submission was deemed to be inadequate when the FISH assay was attempted at Colorado. The study design was modified in June 2015 as a result of the lower than expected number of EGFR FISH+ patients.
**Discussion**
Although activated in July 2009, planning for the study began two years earlier in July 2007. Around this time, data from multiple studies suggested that the efficacy of EGFR tyrosine kinase inhibitors was likely concentrated in patient with tumors harboring EGFR mutations. As EGFR mutation status and EGFR expression by FISH are correlated, this development could have affected accrual to this first line study and may have reduced the proportion of EGFR FISH+ observed.

### P462

#### A systematic review to inform a trial of comprehensive pain management for patients with chronic pain after total knee replacement: the star experience

Jane Dennis[1], Vikki Wylde[1], Andrew D. Beswick[1], Julie Bruce[2], Christopher Ecclesto[3], Nicholas Howells[4], Timothy J. Peters[1], Gooberman-Hill[1]
[1]University of Bristol; [2]University of Warwick; [3]University of Bath; [4]North Bristol NHS Trust
**Correspondence:** Jane Dennis
*Trials* 2017, **18(Suppl 1):**P462

**Background**
Total knee replacement is conducted to relieve pain and improve function, most commonly as a treatment for osteoarthritis. Over 90,000 operations take place annually in the NHS, and knee replacement provides pain relief for most people. However, at three months or more after surgery, around 20% of patients report moderate to severe pain. To inform the design of an intervention to improve management of chronic pain after knee replacement, we conducted a systematic review that identified only one small randomised controlled trial assessing an intervention to treat chronic pain following knee replacement. Given the fact that chronic post-surgical pain is multifactorial with surgical, biological and psychological contributions, we undertook a broader systematic review to evaluate the evidence for the management of chronic pain after any surgery type.
**Methods**
The protocol for the review was registered on PROSPERO in 2015. PICO criteria were: patients aged 18 years, with 90% of participants reporting chronic post-surgical pain; interventions for pain delivered a minimum of three months after surgery: control patients receiving placebo, usual care or alternative pain management intervention. Searches were conducted in MEDLINE, EMBASE, CINAHL, psycinfo, The Cochrane Library, and opensigle. Screening was performed by a single assessor with 10% of records double-screened. The primary effectiveness outcome was pain; that for harm was serious adverse events. Risk of bias was assessed using the Cochrane Risk of Bias tool.
**Results**
Searches run in March 2016 yielded 17,027 records. 66 trials with data from 3,149 participants were included. Most trials included patients with chronic pain after spinal surgery (23 trials) or phantom limb pain (21 trials). Interventions were predominantly pharmacological, including anti-epileptics, capsaicin, epidural steroid injections, local anaesthetic, neurotoxins, N-methyl-D-aspartate receptor antagonists and opioids. Other interventions included acupuncture, exercise, limb liner after amputation, spinal cord stimulation, further surgery, laser therapy, magnetic stimulation, mindfulness-based stress reduction, mirror therapy and sensory discrimination training. Opportunities for meta-analysis were limited by heterogeneity. For all interventions, there was insufficient evidence to draw conclusions on effectiveness.
**Conclusions**
The aim of our systematic review was to synthesise data on the management of chronic pain after surgery. Chronic pain is difficult to treat and combination treatments matched to patient characteristics are advocated. In this review, the majority of studies evaluated pharmacological interventions and we found no studies investigating multidisciplinary or individualised interventions for management of pain after surgery.
The results of our systematic review highlight the need for further evidence to inform recommendations about care provision for patients with chronic post-surgical pain. We are now addressing this gap through a multi-centre randomised controlled trial evaluating the clinical and cost-effectiveness of a care pathway for patients with chronic pain after knee replacement.

### P463

#### Decomposition of the treatment effect estimator in stepped wedge trials: understanding the horizontal and vertical contributions

Andrew Forbes[1], John N. S. Matthews[2]
[1]Monash University; [2]Newcastle University
**Correspondence:** Andrew Forbes
*Trials* 2017, **18(Suppl 1):**P463

**Background**
A linear mixed model incorporating a random cluster effect is the most commonly used model for analysis of complete stepped wedge designs with Gaussian outcomes and a repeated cross-sectional sampling structure. It is recognised that the maximum likelihood estimator of the treatment effect in this model is a combination of horizontal (within cluster) and vertical (between cluster) comparisons. However, the precise nature of this combination has not previously been clearly articulated for these designs.
**Methods**
We apply standard results using partitioned matrices to derive a simple expression for the weighted combination of the horizontal and vertical components of the treatment effect estimator, each presented as linear combinations of cluster-period means. We extend the mixed model to incorporate random effects appropriate for a closed cohort design and derive the analogous results under this design.

#### Results
The weights assigned to the horizontal and vertical comparisons involve a simple expression depending on the number of periods in the design, the cluster size and the intra-cluster correlation. We use this result to describe scenarios in which the treatment effect estimator is dominated heavily by the horizontal comparisons. We provide explicit expressions for the horizontal and vertical components of the treatment effect estimator in a number of example designs and ?A3B2 show $132#?>explain the intuition behind them. We also describe how the decomposition provides a basis for the construction of randomisation tests. The extension to the closed cohort design involves identical horizontal and vertical components as the cross-sectional sampling design, the only difference being in the construction of the weights.
### Conclusions
The decomposition into horizontal and vertical components enables a better understanding of the explicit linear combinations of cluster-period means underlying the treatment effect estimator. It also describes where the maximal information resides in these designs, leading to suggestions for optimal incomplete designs.

### P464
### Design of a practice-changing trial in the ultra-rare condition of Wolfram Syndrome
Kristian Brock[1], Lucinda Billingham[1], Zsuzsa Nagy[1], Tamara Hershey[2], Holly Smith[1], Darren Barton[1], Timothy Barrett[1]
[1]University of Birmingham; [2]Washington University
**Correspondence:** Kristian Brock
*Trials* 2017, **18(Suppl 1):**P464

#### Background
Wolfram Syndrome (OMIM 222300) is an ultra-rare, monogenic, neurodegenerative disorder of children and young adults. Prevalence is approximately 1:700,000. The prognosis is poor as premature death and severe neurological disabilities are not uncommon. The natural history of Wolfram Syndrome includes progressive optic and brainstem atrophy. Many children are registered blind by the age of 18 years. There is no effective treatment.
Sodium Valproate is classed as an anticonvulsant and is currently approved for use in the treatment of epilepsy and bipolar disorder. The cell cycle regulator p21cip1 has been identified as a therapeutic target for Wolfram Syndrome and one of the mechanisms through which sodium valproate is expected to mediate its effect is by increasing p21cip1 expression levels. We investigate the hypothesis that it slows the progression of symptoms.
### Methods
We present a randomised, double-masked, placebo-controlled, multicentre, international clinical trial to investigate whether sodium valproate halts the progression in clinical symptoms of Wolfram Syndrome. We propose the dual primary outcomes: (i) Visual acuity (VA), measured on the logmar scale using standard charts; and (ii) Ventral pons volume (VPV), measured in mm3 by MRI scan. These continuous outcomes are chosen because they are clinically meaningful and associated with disease progression. VA is very important to patients and their families, and any reduction in sight deterioration will be welcome.
Recruitment is severely constrained in this ultra-rare condition. We increase statistical power by conducting longitudinal analyses of the primary outcomes. This is feasible in Wolfram Syndrome because the symptoms under study tend to deteriorate linearly over time. Justification for this claim is given.
Mean outcome trajectories are modelled using linear mixed effects regression, allowing the average rates of change to be different in each arm, and each patient to have their own intercept. This method allows the study of serially-correlated outcomes. Treatment effect is tested by likelihood-ratio test using an alternative, nested model with no fixed effects for treatment arm. Treatment will be considered successful if it is associated with a significant, clinically-relevant reduction in the rate of degradation.
### Results
We assess VA every 6 months for 3 years. To investigate a 60% reduction in the annual rate of degradation from 0.075 to 0.03 logmar units requires 60 evaluable patients in total to provide 80% power and 10% significance.
We assess VPV every year for 3 years. To investigate a 70% reduction in the annual rate of degradation from 81.6 to 24.5 mm3 requires 60 evaluable patients to provide 81% power and 7% significance.
We infer operating characteristics by simulation. The equivalent non-longitudinal analyses would require approximately 120 patients in total.
### Conclusion
This efficient design, which uses a repeated-measures analysis of the primary outcome, will achieve conventional statistical error rates, thereby enabling a potentially practice-changing clinical trial in this ultra-rare condition.

### P467
### Stopping rules for long term clinical trials based on two consecutive rejections of the null hypothesis
Mohamed Mubasher[1], Howard Rockette[2]
[1]Morehouse School of Medicine; [2]University of Pittsburgh
**Correspondence:** Mohamed Mubasher
*Trials* 2017, **18(Suppl 1):**P467

This abstract is not included here as it has already been published.

### P468
### Handling poor accrual in adaptive trial setting: Bayesian interim analysis of rescue trial
Danila Azzolina, Ileana Baldi, Silvia Bressan, Paola Berchialla, Valentina di Leo, Liviana Da Dalt, Dario Gregori
[1]University of Padua
**Correspondence:** Danila Azzolina
*Trials* 2017, **18(Suppl 1):**P468

In several clinical trial settings, it is difficult to recruit the overall sample provided at the design stage, and different problems may occur in patient's enrolment. The amount of information conveyed by a trial terminated prematurely for poor accrual may be minimal. A Bayesian analysis of such a trial may salvage this information, by providing a framework in which to combine prior with current evidence. In this work we propose a Bayesian analysis of a trial candidate for termination due to poor accrual. RESCUE trial is a randomized controlled trial evaluating the effect of adjunctive oral steroids to prevent renal scarring in young children with febrile urinary tract infections. Primary outcome is the difference in scarring proportion between standard antibiotic therapy versus standard therapy + corticosteroids. By study protocol, a frequentist approach to sample size calculation require 92 randomized patients per arm, considering 20% lost follow-up. After 2 years, only 8 patients completed the follow up to determine the study outcome (3 in corticosteroids therapy group and 5 in control group). The sample size was recalculated with the Bayesian Worse Outcome Criterion for differences in proportions (length = 0.3 and coverage = 0.9) applying a 0.5% down-weight. An informative prior on scar proportions was derived from literature considering a scar probability of 0.33 and 0.66 respectively in treatment and control group (Huang YY, 2011). An interim Bayesian analysis on recruited patients has been performed; having a few data to estimate the likelihood, inference was expected to be seriously conditioned by the prior. To assess robustness of conclusion a sensitivity analysis on prior definition has been performed considering 1) informative Beta prior as in sample size estimation 2) informative Beta with 0.5% down-weight 2) uninformative Beta (1,1) prior. Results are compared in term of posterior probability. The estimated Bayesian sample size is 41 infants per arm, leading to a reduction of 51 patients compared with frequentist oneThe Bayesian inference is a flexible tool, compared to frequentist one, taking into account of a-priori knowledge about treatment effect. The informative inference, on small sample, may be weakly influenced by data. However, sensitivity analysis lead to consider the inference robustness. Nevertheless, we advocate to choose beforehand a Bayesian design and not to switch to a Bayesian analysis method that produces a more favourable outcome after observing the data.

### P470

#### Biomarker validation as a clinical trial endpoint: what works and what doesn't

David Raunig
ICON Clinical Research
*Trials* 2017, **18(Suppl 1):**P470

**Background**

As medical imaging technology advances, analysis methods mature and scanners become more globally available, there is an increasing interest to use advanced or novel imaging biomarkers as clinical trials endpoints. MRI, PET, high resolution CT and even ultrasound have demonstrated unique abilities to measure diseases closer to the mechanism of action. Many novel biomarkers are able to show both structural and functional changes and validation studies provide good evidence that imaging may provide both the sensitivity and specificity that have eluded the assessment of these diseases and their absence may actually be at least partly responsible for the failure to develop effective therapeutics. However, many of the published studies that declare biomarkers to be validated for use fall far short of demonstrating fitness for use. In 2015, the Quantitative Imaging Biomarker Alliance published the results of a two-year collaborative effort to standardize the statistical and technical methods and metrics to validate a biomarker for use as an endpoint in a clinical trial. Since then, these methods have been used to validate several imaging biomarkers for study-specific use as primary and secondary endpoints by providing statistically and clinically rigorous study designs to sufficiently demonstrate that these biomarkers are reliably acquired and analyzed and that there is reasonably good prediction of a clinically accepted outcome.

**Methods**

Standardized statistical methods that are globally recognized by metrology standards agencies, including the Bureau of International Weights and Measures (BIPM) and the National Institutes of Standards and Technology (NIST) are used to define reliability in terms of repeatability, reproducibility and linearity. Standard metrics include statistical estimation of the variance components that eventually define how reliable the imaging biomarker would be in a clinical trial setting, and a linear relationship to the truth. An additional component to validation is the ability of the imaging biomarker to predict clinical outcome. Results Two case studies, one with a quantitative and one with a semi-quantitative imaging biomarker evaluation of medical imaging will be examined for what would comprise a complete dossier for validation or qualification. From these case studies, we will summarize a standard protocol for a quantitative imaging biomarker validation study, risks to the successful completion of these trials and methods to incorporate biomarker validation into the drug development process.

### P471

#### When can hypothetical pilot data predict real-world trial results? A systematic concept review and framework

Jamie Brehaut[1], Tavis Hayes[1], Doug Coyle[2], Ian Graham[1]
[1]Ottawa Hospital Research Institute; [2]University of Ottawa
**Correspondence:** Jamie Brehaut
*Trials* 2017, **18(Suppl 1):**P471

**Background**

Trials of complex health services interventions often lack detailed preparatory work explicating the mechanisms by which the intervention is supposed to work. This lack of preparatory work contrasts sharply with drug trials, which can be the culmination of many years of preclinical work. The UK Medical Research Council provides guidance that underscores this issue, and highlights the need for better theory development and modeling to support, justify, and optimize trials of complex interventions. We propose that this requires an understanding of when 'hypothetical' Elements (e.g. Using healthy participants instead of patients; piloting interventions on physicians outside their clinical practice) can be used to predict 'real-world' Outcomes, analogous to our extensive understanding of which animal models are appropriate to different human biological mechanisms. At the moment, studies involving hypothetical elements are discounted in the literature (i.e. Considered not clinically relevant, excluded from systematic reviews), often because of implicit and unsupported objections that such data cannot predict real-world outcomes. While many literatures have explored predictors of the association between hypothetical and real-world decisions, none has summarized these in a manner that would help health care intervention developers know when hypothetical pilot data are likely to agree with the real world.

**Objective**

To conduct a systematic concept review of the factors affecting the association between hypothetical and real-world decision-making.

**Methods**

Our research question was: 'What are the factors that affect the association between hypothetical and real-world decisions?' A systematic, peer-reviewed search strategy was developed based on keywords, ?A3B2 show $132#?>titles, and MESH headings related to (i)decision making or behaviour (and related concepts e.g. Reasoning, risk taking); (ii) hypothetical situations (e.g. Uncertainty, proxy), and (iii) real world situations (e.g. Reality, everyday), and applied to psychinfo and Medline in December 2015. Two coders extracted study specifics, as well as quotations describing the relevant factor associating hypothetical and real outcomes. Factor wordings were standardized, collated, and organized into themes.

**Results**

A total of 1846 studies captured by our search strategy ultimately yielded 59 studies that contributed at least one factor. Contributing articles addressed issues of behavioural economics(80%), psychology of reasoning (31%), social psychology (17%), health behaviours (12%), and neuroscience(5%). A total of 42 factors were grouped into 5 categories, including Personal Characteristics (9 factors e.g. Age, cognitive ability, personal relevance); Presentation Characteristics (8 factors; e.g. Framing effect, time for reflection, issue salience); Cognitive Factors (17 factors; e.g. Discounting, normative beliefs, social desirability); and Participant Characteristics (1 factor; samples match target population).

**Discussion**

This work provides a summary of the factors known to affect when studies with hypothetical elements might be expected to agree with real world decisions. Based on a range of related literatures, our framework will aid investigators who are interested in understanding whether the design of their pilot study will allow them to draw conclusions about the real world. This initial work will help us to pilot our health services trials more effectively, making the ultimate interventions more efficient and effective.

### P472

#### Rituximab for the treatment of neuromyelitis optica: an application of individual patient data meta-analysis in a rare disease

Siobhan Bourke[1], Catrin Plumpton[1], Catrin Tudur Smith[2], Anu Jacob[3], Dyfrig Hughes[1]
[1]Center for Health Economics and Medicines Evaluation, Bangor University; [2]Institute of Translational Medicine, University of Liverpool; [3]The Walton Centre, Liverpool
**Correspondence:** Siobhan Bourke
*Trials* 2017, **18(Suppl 1):**P472

**Background**

Neuromyelitis optica (NMO) is a rare, autoimmune disease of the central nervous system that affects approximately 700 patients in the United Kingdom. It is characterised by relapses of the optic nerves and spinal cord. To reduce the severity and frequency of these attacks, patients are treated with immunosuppressants, including rituximab which is a second line therapy for NMO. Rare diseases pose unique challenges for clinical trials, including difficulties in recruiting sufficient numbers. Many studies are observational and prone to bias. In the absence of high quality randomised controlled trials, the use of individual patient data (IPD) meta-analysis to synthesise the results of existing studies whilst accounting for confounders provides an opportunity to summarise the available evidence to inform treatment decision making.

#### Objective
The aim of this paper is to review all available information to evaluate the effectiveness of rituximab in NMO.
### Methods
We included all experimental and observational study types that assessed rituximab for the treatment of NMO patients. We performed a literature search using MEDLINE, EMBASE, Web of Science, and Cochrane. Risk of bias was assessed for each study. The primary outcome was time until relapse; other outcomes of interest included ?A3B2 show $132#?>Expanded Disability Status Scale (EDSS), patient demographics, annualized relapse rate, NMO igg status, disease duration, number of relapses (before and after treatment), number and timing of rituximab doses. The authors of each study were contacted to obtain individual patient data. Where data were not forthcoming, data was extracted electronically by digitising figures presented in published papers.
### Results
Thirty-five studies involving 393 patients have been included. Of these, 30 were case studies and the remaining 5 were only available in abstract form, no RCT were identified. IPD for 186 patients were extracted from papers. Variable quality of the data has been noted with some papers not reporting key outcome information. All studies were poor quality with no study adjusting for confounders. Authors of selected studies have been contacted to share their data,responses have been positive, however no disclosure of data have been made at this time. There were 131 (70%) women, 13 (7%) men and 42 (23%) unknown participants, with a mean age of 37 years and disease duration of 41 months. The most-frequently used rituximab regimen was two 1 g doses separated by 14 days in 37 cases (28%).The average EDSS score before (after) treatment was 5.3 (4.3). The average number of relapses before (after) treatment was 5 (1). We will be using a Cox-(proportional hazards) regression model to predict the time to relapse rate whilst adjusting for important confounders.
### Conclusions
This study is ongoing and these preliminary results are susceptible to change. It is hoped that constructing a robust evidence based review can lead to more efficient RCTs. Bayesian design RCT have been suggested as a solution to small population trials, incorporating prior information on efficacy, can increase the possibilities for other RCT designs i.e. Non- inferiority or adaptive designs that previously were not feasible for a rare disease trial.

### P473
## The effect of the non-pharmacological extension of consort in quality of reporting of behavioural weight loss RCTs
Simon Bacon[1], Christina Kazazian[1], Ariane Jacob[2], Kim L. Lavoie[2]
[1]Concordia University & CIUSSS-NIM HSCMI; [2]UQAM & CIUSSS-NIM HSCM
**Correspondence:** Simon Bacon
*Trials* 2017, **18(Suppl 1):**P473

#### Background
Increased quality of reporting of randomized controlled trials (RCTs) has been associated with the publication of the main CONSORT Statement. Over time there have been a number of extensions to the CONSORT Statement, such as the Non-Pharmacological Trial [NPT] extension, yet we have little data on how these have changed the reporting practices of investigators.
### Objective
The aim of this paper was to assess the change in quality of reporting of RCTs for behavioural weight loss programs using CBT with the 2008 publication of the CONSORT NPT extension.
### Methods
A systematic review was conducted to identify randomised controlled trials that assessed the efficacy of cognitive behavioural therapy-based weight loss interventions on eating behaviour or psychological variables. The Downs and Black checklist was used to score the quality of reporting of 15 RCTs that were published before (3 trials) and after (12 trials) the publication of the 2008 CONSORT NPT.
### Results
There was a significant increase in the number of criteria that were fully met (M (SD) pre-2008 = 15.0 (1.0) vs. Post-2008 = 20.0 (2.7), F = 9.75, p = .008) and fully or partially met (M (SD) pre-2008 = 15.7 (1.2) vs. Post-2008 = 20.2 (2.6), F = 8.50, p = .012). There was also a significant reduction in the number of criteria that were not met or were ambiguous (M (SD) pre-2008 = 11.3 (1.2) vs. Post-2008 = 6.8 (2.6), F = 8.50, p = .012). However, it should be noted that even with the improved reporting many checklist items were still not being included (e.g., adverse event reporting, representativeness of the sample, blinding).
### Conclusions
This study showed that, although there seems to be some improvement with the publication of the CONSORT NPT Statement, its effects are still less than ideal. CONSORT should be more widely and strongly endorsed, and enforced, in order to have complete and understandable behavioural RCT reports.

### P474
## Improving the planning and monitoring of recruitment to clinical trials
Efstathia Gkioni[1], Roser Rius[2], Carrol Gamble[3]
[1]University of Liverpool, Polytechnic University of Catalonia, Paris Descartes University; [2]Polytechnic University of Catalonia; [3]University of Liverpool
**Correspondence:** Efstathia Gkioni
*Trials* 2017, **18(Suppl 1):**P474

#### Background
Successfully recruiting the pre-specified number of patients to time and target within clinical trials remains a difficult challenge that negatively impacts all stakeholders in a clinical trial. Current methods to monitor recruitment in practice appear limited to the usual comparison of the predicted and actual recruitment curves and the size of discrepancy. In 2010 a systematic review of methods to predict recruitment was conducted, which identified five classes of models and their limitations.
### Objectives
To update the systematic review by Barnard et al. (2010) to identify new methods to predict recruitment in clinical trials and determine whether these new methods address the limitations of methods previously identified. Identify perceived barriers to implementing these models in clinical trials.
### Methods
The project will update the systematic review of Barnard et al. (2010). This update will include methods identified and published from August 2008 until present. The Online Resource for Recruitment Research in Clinical Trials database (ORRCA) will be used to identify relevant literature. Newly identified methods will be assessed for eligibility. Methods will be assigned to existing proposed classifications of unconditional, conditional, Poisson, Bayesian and Monte Carlo Simulation Markov Model with new classifications as appropriate. The assumptions made by each method will be identified and compared between models. Levels of information required to implement the models, will be considered and applied to real examples of ongoing or recently completed clinical trials.
### Expected results
The results of this systematic review will explore the advances in methodology to predict recruitment in clinical trials. It will highlight limitations of existing methods and barriers to implementation highlighting direction for further developments. In this way we can provide more reliable predictions of recruitment based on each different trials recruitment needs. The benefits of more accurate predictions will be the reduction of the deviation between observed and expected recruitment curves.

### P475

#### Graphical display techniques for subgroup analysis

Yi-Da Chiu[1], Franz Koenig[2], Martin Posch[2], Thomas Jaki[1]
[1]Medical and pharmaceutical statistics research unit, department of Mathematics and Statistics, Lancaster University; [2]Center for Medical Statistics, Informatics, and Intelligent Systems, Medical University of Vienna
**Correspondence:** Yi-Da Chiu
*Trials* 2017, **18(Suppl 1):**P475

Subgroup analysis has received extensive attention in recent clinical research for the development of stratified medicine. This tendency reflects the advance of genetic testing and the potential exploitation of heterogeneity in subgroups. It also emphasises the identification of medical interventions to suitable subpopulations (as defined by biomarkers) for efficacy and against the others for safety. Graphical approaches are routinely employed in subgroup analysis, typically for describing effect sizes of subgroups. Such visualisation encapsulates subgroup information and greatly boosts the clinical decision-making process. However, existing approaches still have inherent drawbacks and their use may lead to misinterpretations to subgroup effect sizes. For instance, forest plots provide no insight on the overlap of different subgroups; additionally, whether or not a subgroup's confidence interval crosses the no-effect point does not necessarily imply a lack of effect or contribute an effect to the subgroup. It is therefore crucial to correctly depict the effect sizes and information, particularly in order to prevent overstating effects. To develop an optimal visualization approach, we assessed graphical approaches for subgroup analysis under a synthetic dataset. Several techniques (such as level plots, barcharts, Venn diagrams, tree plots, forest plots and matrix plots) were applied to exhibit certain subgroup information. Some have been further improved by mitigating their original demerits. In final, we summarise the general strengths and failings of the graphical approaches and outline potential visualisation techniques.

### P476

#### Venous thromboembolism and cancer trials evidence synthesis: dealing with both complex knowledge and unexplained heterogeneity

Martin Adamcik
Assumption University of Thailand
*Trials* 2017, **18(Suppl 1):**P476

##### Evidence Synthesis

Fixed-effect meta-analysis is a powerful instrument for combining related studies but such a combination is considered flawed if studies use different methods or investigate different populations. If differences are merely statistically detected then techniques of random-effects meta-analysis are employed to combine them. On the other hand, complex knowledge is difficult to interpret and although Bayesian methods are currently being developed they are unable to deal with complex knowledge when heterogeneity is statistically detected.

##### Venous Thromboembolism and Cancer

According to a large meta-analysis from 2008, around 10% of patients having acute unprovoked venous thromboembolism are expected to be diagnosed with cancer within a year. Nevertheless, several recent clinical studies indicate a lower incidence of diagnosis of cancer in such patients and also a lower sensitivity of extensive screening for cancer than the large meta-analysis suggests. Despite similarities, heterogeneity was statistically detected between those studies. The variability in screening designs requires a method that can deal with complex knowledge to combine them.

##### Method

A new method for meta-analysis that deals with both complex knowledge and unexplained heterogeneity was developed. The method is thus applicable to synthesise a wide range of related medical trials in different fields. The method uses propositional probabilistic logic to represent complex findings and merges them using an operator that was shown to be appropriate in the presented setting by an argument related to the maximum entropy principle.

##### Results

Our meta-analytical findings indicate the following: The incidence of diagnosis of cancer in patients with unprovoked venous thromboembolism is somewhere between 6.97% and 9.79%. Routine evaluation detects between 36.59% and 49.61% of those cancers while the combination of routine and extensive screening methods detects between 74.99% and 83.25% of those cancers. Therefore, the incidence of cancer diagnosis is still relatively high and the combined screening is superior to routine evaluation in detecting such an occult cancer.

### P477

#### Investigatin g the use of evidence synthesis in the design and analysis of clinical trials

Gemma Clayton[1], Isabelle Smith[2], Hayley E. Jones[3], Julian P. T. Higgins[3], Benjamin Thorpe[2], Duncan Wilson[2], Robert Cicero[2], Kusal Lokuge[4], Julia Forman[5], Borislava Mihaylova[4]
[1]University of Bristol; [2]Clinical Trials Research Unit, University of Leeds; [3]School of Social and Community Medicine, University of Bristol; [4]Health Economics Research Centre, Nuffield Department of Population Health, University of Oxford; [5]Cambridge Clinical Trials Unit, University of Cambridge
**Correspondence:** Gemma Clayton
*Trials* 2017, **18(Suppl 1):**P477

##### Background

When designing and analysing clinical trials, using previous relevant information, perhaps in the form of evidence syntheses, can reduce research waste. We conducted the INVEST (investigating the use of Evidence Synthesis in the design and analysis of clinical Trials) survey to summarise current evidence synthesis use in trial design and analysis, to capture the opinions of trialists and methodologists on such use, and to identify any barriers.

##### Methods

We distributed the INVEST survey during the two-day International Clinical Trials Methodology Conference in November 2015, and provided access to an online version for one month following the conference. All respondents were asked to indicate their views on use of evidence synthesis in trial design and analysis and to rank what they considered to be the three greatest barriers to such use. Respondents who indicated that they had been involved in trial design and/or analysis were asked additional questions about whether and how they have used evidence synthesis in practice. Among these respondents we contrasted their views on whether evidence synthesis methods should be used versus actual use.

##### Results

Of approximately 638 people attending the conference, 106 (17%) completed the survey, half of which were statisticians. Support was generally high for using a description of previous evidence, a systematic review or a meta-analysis when designing a trial. Fewer participants indicated support for use of network meta-analyses, decision models and value of information analyses. Only about 5% felt that external evidence should not be used in the analysis of a trial, with an additional 20% being unsure. Among respondents involved in trial design and/or analysis, fewer indicated that they had used evidence syntheses to inform design or analysis during the last 10 years than indicated that these methods should be used. For example, only 6% (5/81) had used a Value of Information analysis to inform sample size calculations, compared with 22% (18/81) feeling that this was desirable. The greatest perceived barrier to using evidence synthesis methods in trial design or analysis was time constraints, followed by a belief the new trial was the first in the area.

##### Conclusion

The INVEST survey indicates that, generally, trial teams are using evidence synthesis in trial design and analysis less than they think is desirable. Since evidence syntheses can be resource-intensive, we advocate additional research and training on ways to undertake them efficiently. Investment in adequate resources and training at this stage could lead to cost savings in the long term.

### P478

#### Performance bias in trials that cannot blind participants and healthcare providers to assigned interventions: implications for trial conduct

Roy Elbers, Jelena Savovic, Natalie Blencowe, Julian P. T. Higgins, Jonathan A. C. Sterne
University of Bristol
**Correspondence:** Roy Elbers
*Trials* 2017, **18(Suppl 1):**P478

**Background**
Successful blinding of participants, healthcare providers and trial personnel prevents knowledge of assignment from influencing adherence to intended interventions. However, blinding in nonpharmacological trials is difficult, and these trials are often considered to be at high risk of performance bias. The revised Cochrane risk of bias tool for randomized trials (rob 2.0) differentiates between the effect of assignment to intervention and the effect of starting and adhering to intervention. The former is the effect of interest in an intention-to-treat analysis and the latter is the effect of interest in a per-protocol analysis. Issues of blinding, implementation and adherence to intended interventions differ importantly between these two effects.
**Objective**
To provide guidelines for trialists to reduce bias due to deviations from intended interventions in nonpharmacological trials in the context of an intention-to-treat analysis.
**Methods**
Within the development of the rob 2.0 tool, one working group was tasked with the development of signalling questions, criteria for reaching a judgment and full guidance on the domain 'bias due to deviation from intended interventions'. The new tool provides a more nuanced judgement of performance bias in nonpharmacological trials. In trials that aim to assess the effect of assignment to intervention, deviations from intended interventions that reflect usual care do not lead to bias. In the current project we extended the insights acquired during development of the rob 2.0 tool to propose guidelines for clinical trialists. These guidelines aim to inform trial conduct from planning through to reporting, with the aim of minimizing performance bias in nonpharmacological trials.
**Results**
The guidance includes three components. First, interventions should be clearly articulated in the protocol, including any plans to stop or modify interventions in response to clinical events. In particular, trialists should define in advance any co-interventions that would be administered as part of usual care. Second, during the trial, all deviations from the protocol interventions that do not reflect usual care should be monitored and recorded. These deviations from the intended interventions might include co-interventions, contamination, switches to other interventions, non-adherence, or failure to implement some or all of the intervention. The important consideration is that these deviations occur because of the trial context rather than as a reflection of routine care. Third, the departures identified should be reported fully and clearly to facilitate risk of bias judgements by trialists themselves, peer reviewers and systematic review authors.
**Conclusion**
Development of the rob 2.0 tool has led to supplementary guidance aimed at clinical trialists. The work around performance bias presented here is part of the wider initiative to cover all biases that might arise in clinical trials. This initiative recognises that clinical trials and evidence synthesis are part of the same continuum of effectiveness research and aims to ensure that method development in one area is maximally integrated with applications in the other area to ensure optimal trial conduct and reporting.

### P479

#### What might a global health trials methodology research agenda look like?

Anna Rosala-Hallas[1], Paula R. Williamson[2], on behalf of The Global Health Trials Methodology Research Agenda Steering Committee
[1]Clinical Trials Research Centre, University of Liverpool; [2]North West Hub for Trials Research Methodology, Clinical Trials Research Centre, University of Liverpool
**Correspondence:** Anna Rosala-Hallas
*Trials* 2017, **18(Suppl 1):**P479

**Aim**
To identify priorities for methodological research to assist the design, conduct, analysis and reporting of clinical trials in low and middle income countries (LMICs).
**Background**
Research into methods used to design, conduct, analyse and report clinical trials is essential to ensure that clinical decisions made are derived from robust and reliable evidence. In a previous study [1] the key stakeholder group of Directors of UK Clinical Research Centre (UKCRC) registered clinical trials units (CTUS) identified the most important methodology research topics. However, it cannot be assumed that these research priorities reflect those in LMICs. There is a need for research to come from LMICs countries and it has been stated, in the 2013 World Health Report, that LMICs must become the generators and not the recipients of research data in order for improvements in public health outcomes in these most undeserved regions of the world. In order for any progress to be made in LMICs there is a critical need for this research. This is to ensure that particular methodological issues are identified and communicated to health care workers in these regions so that they might optimise future designs for trials.
**Methods**
An online survey will be conducted November 2016 to March 2017 with members of the Global Health Network, globalsurg, The Clinical Research Initiative, Cochrane, Evidence Aid and other clinical trials researchers with LMIC experience. The first round will be an online survey in relation to the design, conduct, analysis, reporting and interpretation of a trial. Participants will be asked to list up to three topics they feel are important priorities for trials methodology research. Topics identified will be independently reviewed and categorised by two members of the research team and split into two separate lists for the second round. The primary list will consist of topics identified by more than one respondent and the second list will consist of topics identified by a single individual. In the second round the participants will rank the topics in order to identify priorities within both the primary and secondary lists.
**Results**
A list of the top priorities for trials methodology research in LMICs countries will be presented. Common priorities to those in high income countries will also be noted.
**Conclusions**
By presenting these top priorities we will have the foundations of a global health trials methodological research agenda which we hope will instigate further methodology research in specific areas in order to increase and improve trials in LMICs.

**Reference**
[1] Tudur Smith C, Hickey H, Clarke M, Blazeby J, Williamson PR. The Trials Methodological Research Agenda: Results from a priority setting exercise. Trials 2014; 15:32 doi:10.1186/1745-6215-15-32.

### P480

#### Implementation in dental trials: an exploration of trial meta-processes

Paul Brocklehurst, Beth Hall
Bangor University
**Correspondence:** Paul Brocklehurst
*Trials* 2017, **18(Suppl 1):**P480

As highlighted by a recent report for the National Institute for Health Research's Health Services and Delivery Research funding stream, bridging the implementation gap is increasingly being recognised as an intransigent challenge for complex interventions in health services research. Patient and Public Involvement (PPI), process evaluation and the use of theoretical frameworks have all been highlighted as being important 'meta-processes' in trial conduct and design to reduce research waste and improve implementation of trial evidence. In addition, early consideration of an interventions pathway to impact has been advocated. The aim of this exploratory study was to examine the Cochrane Database of Trials over the last six years to determine the level of utilisation of PPI, process evaluation and theoretical frameworks alongside dental trials, whilst concurrently exploring whether the pathway to impact and implementation was being considered.

The Cochrane Database of Trials was searched for reports on Randomised Controlled trials (RCTs) and protocols of RCTs over a five year period (2010–2016). As the aim of this exploratory study was to get a 'snap-shot' of current activity, other subscription databases, open access databases and the grey literature were not searched. Any dental intervention that would have utilised psycho-social mechanisms explicitly or implicitly was included, whilst any intervention that acted through a pharmacological mechanism was excluded. Included studies were assessed to determine whether they reported on any of the 'meta-processes' detailed above. Titles and abstracts identified by the electronic search were downloaded to a reference management database and duplicates were removed.

582 of 932,577 records had the term 'dental', 'oral' and 'trials' in the Title, Abstract or Keyword. 56 studies related to psycho-social interventions or had psycho-social pathways to implementation. The proportion of trials that reported PPI, process evaluation, theoretical framework or mentioned implementation 0%, 21.7%, 43.5% and 4.3% respectively, whilst the proportion of protocols was higher (46.7%, 60.0%, 73.3% and 6.7%).

The use of 'meta-processes, in trial design and conduct has improved, although considerations about pathway to impact and the implementation of the research evidence, once generated, appears to remain poor.

### P481

#### Retrospective preparation of trial results for regulatory submission: challenges and lessons learned

Cathryn Rankin, Rachael Sexton, Evonne Lackey, Sarah Basse, Antje Hoering, Michael LeBlanc
SWOG Statistics and Data Management Center
**Correspondence:** Cathryn Rankin
*Trials* 2017, **18(Suppl 1):**P481

Upon release of primary results from a positive SWOG-coordinated Phase III myeloma trial, a pharmaceutical company supplying study drug plans to retrospectively use the results in a regulatory filing exactly two years after presentation of results. The trial accrued 525 patients from 2008 to 2012 with patients being followed for six years after randomization. After an initial meeting between the SWOG Statistical and Data Management Center (SDMC) and the company in May 2016, the company outlined an aggressive plan to reconsent all living patients in order to extend follow-up, capture additional data and initiate site monitoring including 100% source data verification (SDV) for all registered patients. All patient data as of December 2016 needs to be complete, query-free and verified by March 2017 in order meet the timeline for submission to the FDA in December 2017.

The protocol revision requiring reconsent and addition of new case report forms (CRFs) was distributed to the sites on 10/1/2016. Local institutional review board (IRB) review was required as this study was initially reviewed by such prior to formation of the CIRB. A CRO was hired and trained on the SWOG SDMC systems in September 2016 to facilitate onsite monitoring of nearly 100 physical sites beginning in November 2016. Data are to be complete and every data point verified by the CRO by February 2017 in preparation of a final data transfer in March 2017 to the pharmaceutical company.

An overview of all systems used during the conduct of this trial highlighted challenges including implementation of an online EDC system and subsequent capabilities for amending data online. Required updates in adverse event reporting (CTCAE 3.0 to 4.0) over the tenure of this trial also complicated the monitoring efforts. Drug company and CRO staff education of both legacy and current systems was necessary in order to evaluate and query all data for regulatory submission.

The membership structure and alignment of participating sites changed as the NCTN and NCORP networks replaced the cooperative group structure in 2014. With the configuration of the network membership shifting, it is vital to identify updated site contacts, as well as track and communicate with the cross-network membership. Sites have experienced staff turnover and some no longer participate in cooperative group research.

**Lessons learned**

Achievable goals, concise training, communications, and sufficient timelines are critical to prepare sites and monitors for extensive data verification. The SDMC staff has evaluated over 4500 inquiries generated by the company after initial review of clinical data, posting and resolving the relevant queries to sites (only 1350/3600 remain outstanding) while reviewing incoming data generated by additional CRFs and other site-initiated questions. Sufficient staffing and dynamic data management systems are vital.

The clinical data management system (CDMS), Medidata Rave®, provided by the NCI will benefit future endeavors similar in nature, both in communication and monitoring efforts. We will continue to identify additional challenges and lessons learned as well as strive to compare potential key outcomes based on standard data collection and review compared to the intense retrospective review.

### P483

#### Design and coordination of the DECAAF II randomized international trial

Richard Holubkov, Tom Greene, Leonie Morrison-de Boer, Russell Telford, Tyler Bardsley, Molly McFadden, Alicia Peterson, Christina Pacchia, Jeffrey Yearley, Ashley Snyder
University of Utah
**Correspondence:** Richard Holubkov
*Trials* 2017, **18(Suppl 1):**P483

The Efficacy of Delayed-Enhanced MRI-Guided Fibrosis Ablation vs. Conventional Catheter Ablation of Atrial Fibrillation (DECAAF) II trial is evaluating catheter-based ablation, guided by 3-dimensional MRI highlighting areas of fibrosis, as treatment for atrial arrhythmia. The observational DECAAF I cohort study found an association of atrial fibrosis burden with arrhythmia recurrence. Therefore, DECAAF II is comparing ablation specifically targeting visualized fibrosis to standard-of-care ablation performed without MRI guidance, among atrial arrhythmia patients undergoing first-time ablation.

Design and execution of DECAAF II are complex for several reasons. First, before a clinically eligible patient can be randomized, the baseline 3-dimensional MRI must be obtained, confirmed to be of adequate quality for study use, and processed by a central core laboratory, all with rapid turnaround to have these images available during intervention for patients assigned to the MRI-guided strategy. The core laboratory also determines extent of coronary fibrosis, a stratification factor for trial randomization. Second, the primary outcome of (time to) atrial fibrillation recurrence is mainly determined by a smartphone-based application that enrolled patients must implement daily. This technology is expected to increase statistical power to detect a treatment effect compared to previous trials, which employed conventional recurrence assessment methods such as standard ECGs that are administered much less frequently. However, adherence to this self-administered approach must be aggressively monitored. While the smartphone-based application generates regular reminders, the Coordinating Center must be aware of centers that have substandard patient compliance. The primary recurrence outcome will incorporate all available testing performed on the patient, including standard of care ECGs and Holter monitors, to eliminate dependence on full compliance with daily self-assessment.
Third, the trial design includes a 90-day post-intervention "blanking period" That allows atrial tissue targeted during the ablation procedure to respond to treatment and heal. Therefore, the success of the procedure is more appropriately assessed excluding any arrhythmias observed during this period. In the primary analysis, "time zero" for counting arrhythmia recurrence events therefore begins at 90 days after initial intervention (any repeat ablations after this time will also count as primary outcomes). This "blanking period" is potentially advantageous for training patients in the habitual daily use of the monitoring device. By the end of this period, participating physicians are encouraged to discontinue patients from treatment with anti-arrhythmic medications, whose use is considered a confounding factor for primary outcome assessment.
Assuming that MRI-guided ablation reduces relative risk of atrial arrhythmia recurrence by 25%, DECAAF II must observe 517 events in the two equally sized treatment arms to yield 90% power for a logrank test to detect a treatment effect with respect to recurrence time. With enrolled patients followed up to 18 months after index procedure, various realistic event rate scenarios indicate that from 750 to 1100 (best estimate: 900) patients will need to be recruited. The first patient was randomized in July 2016.
We will present details of the DECAAF II study design and implementation, and issues encountered in the initial rollout of the trial at vanguard study centers.

### P484

#### Implementation of CTMS functionality for remote monitoring of informed consents

Catherine Dillon
Medical University of South Carolina
*Trials* 2017, **18(Suppl 1):**P484

Informed consent is a high-risk activity that should be monitored. The FDA's 'Oversight of Clinical Investigations- A Risk-Based Approach to Monitoring' encourages alternative approaches to traditional monitoring procedures to improve sponsor oversight of human subject protection. It specifically permits the use of internet portals where sites can upload signed consent (IC) forms or other records for remote verification by designated monitors. Functionality was integrated into the Clinical Trial Management System (CTMS) for DEFUSE 3, a NINDS-funded Stroke Trial Network study, to facilitate remote monitoring of informed consents. This presentation will explore the implementation strategy utilized which links IC submissions directly to the subject ID and ecrf submission, while storing Protected Health Information (PHI) on a separate server. This strategy allows for IC submissions to be processed in a similar way as other clinical trial data, while allowing access to PHI only to designated remote monitors. While the potential benefits of this technique include reducing costs, increasing efficiency, and early detention of errors, serious privacy and confidentiality issues had to be addressed. Implementation strategy, advantages, challenges, lessons learned from the Defuse 3 Trial, and other applications of this monitoring strategy will be discussed.

### P485

#### The training documentation form – Going beyond the basics for the national institute on drug abuse (NIDA) national drug abuse treatment clinical trials network (CTN)

Tracee Williams, Radhika Kondapaka, Dikla Shmueli-Blumberg, Matthew Wright, Dagmar Salazar, Kayla Williams, Julia Collins, Eve Jelstrom, Robert Lindblad
National Drug Abuse Treatment Clinical Trials Network for the NIDA CCTN
**Correspondence:** Tracee Williams
*Trials* 2017, **18(Suppl 1):**P485

According to ICH GCP guidelines, investigators and research staff with delegated trial-related duties should be "Qualified by education, training, and experience" (ICH E6 GCP, 1996) to maintain integrity and quality in clinical trials. Training documentation is essential to demonstrate compliance of the investigator and research staff of these guidelines. Nonetheless, many researchers and sponsors, in particular in multicenter trials, find it difficult to adequately and accurately document the staff training requirements. When multicenter trials are conducted within a network, it is important to develop a sustainable level of standardization in training requirements across sites and studies that demonstrate the competency of the individuals being trained.
The Training Documentation Form (TDF) is a comprehensive document that tracks all training requirements for each study staff member correlated to their study role(s). The TDF clearly defines the training expectations and requirements from various stakeholders (e.g., the Sponsor, Institutional Review Board) as they relate to the responsibilities for each study role, and consistent with the Study Training Plan (STP) and Site Delegation of Responsibilities Log. When completed, the TDF demonstrates that staff members are qualified and fully trained for their study role(s) prior to performing delegated study activities. The TDF developed for the National Drug Abuse Treatment Clinical Trials Network (NIDA-CTN) by the Clinical Coordinating Center at The Emmes Corporation is a user-friendly modifiable electronic document that includes these basic elements critical to a TDF. It captures each staff member's name, research site, and delegated study role(s). The TDF lists all training outlined in the STP and maps the minimal required training and certification prescribed per study role in a grid, based on the staff's assigned role (e.g., study physician,) and assigned tasks (e.g., prescribe medication, data entry) for the study. The TDF efficiently organizes the training curriculum in accordance with the investigative team's predetermined decisions as to the various roles and associated training requirements. It includes both general training (e.g., Human Subjects Protection) and protocol-specific training requirements (e.g., administration of investigational product, conduct of study assessments). The TDF also captures the dates that staff completed each required training task and the final date of overall training completion, the latter of which is documented on the Site Delegation of Responsibilities Log to serve as the staff's starting date on the study. When all required training has been completed, the TDF is signed by the staff member and endorsed by both the site's Principal Investigator and the research center's training representative, who collectively confirm that staff members are ready to begin study responsibilities.
The NIDA CTN has implemented this standardized TDF on seven studies since 2013, aiding in setting up expectations for training documentation across studies while minimizing the difficulty of preparing and tracking the training completed by research staff. The TDF has been welcomed by the quality assurance monitors and the research management teams and has lead to more efficient study start up as well as provided a valuable tool for documentation of research staff competency in delegated study activities.
CTN Contract # HHSN271201500065C

### P486

#### Good order is the foundation of all things: a strong project management structure underpins successful research at Keele CTU

Sarah Lawton, Kris Clarkson, Ruth Beardmore, Irena Zweirska, Martyn Lewis, Nadine E. Foster
Keele Clinical Trials Unit
**Correspondence:** Sarah Lawton
*Trials* 2017, **18(Suppl 1):**P486

**Introduction**
Keele Clinical Trials Unit (CTU) is a UKCRC registered CTU based within the Faculty of Medicine and Health Sciences at Keele University. It specialises in the development and delivery of both feasibility and definitive multicentre clinical trials, an increasing portfolio of Clinical Trials of Investigational Medicinal Products (CTIMPs) and epidemiological studies, in primary care and at the primary-secondary care interface. An effective project management structure is essential for the delivery of high quality research.
**Background**
97% of Keele University's research was deemed to be world-leading, or of international importance in the REF 2014 and Keele CTU, ?A3B2 show $132#?>providing support for the design, delivery, analysis, reporting and dissemination of applied clinical research is contributing to this success. Specialist expertise in the areas of trial design, intervention development, biostatistic approaches, and regulatory ?A3B2 show $132#?>coordination have been developed and deployed. This expertise requires a strong project management structure to conduct and deliver clinical research to the highest quality standards.
**Methods**
A structural flow beginning at project conception through to successful dissemination of results for each CTU supported research project is employed. From conceptualisation, early research ideas are presented and discussed at a Clinical Studies Think Tank, attended by specialist and generalist clinicians as well as methodologists, resulting in research design improvements. Prior to grant application, projects seeking CTU collaboration are considered against CTU adoption criteria including; strategic fit, sponsorship, expertise, capacity and funding. Next follows project feasibility and visualisation of project operationalisation, then review by a team of research partners that includes the NIHR Clinical Research Network, before moving into the business of a CTU Operations Group. From here, projects are allocated to CTU Trials Managers and by employing the skills from a variety of integrated working groups, the research is delivered. Working groups, such as the Health Informatics and Standard Documents working groups, provide invaluable support for project delivery. Within each working group, innovative and effective methodologies are developed, that include technological advances and standardised resources. This process is underpinned by a Quality Management System (QMS) implemented from the Quality Assurance office, ensuring consistency and adherence to regulatory obligations.
**Results**
Employing this project management structure results in a transparent and auditable flow of information and processes within Keele CTU. Dedicated project management forges strong communication links within research teams and with collaborators and participants. Over 25 projects are presented at Clinical Studies Think Tank meetings per year and Keele CTU is currently supporting a portfolio of over 40 research projects, each managed efficiently and effectively within the resources available to secure the delivery of projects to time and target.
**Conclusions**
Keele CTU is increasing its portfolio of research projects whilst making strides with innovative and effective methodologies. This all needs to be carried out within a robust and supportive QMS to ensure successful project delivery. Good order is key to the foundations of any project. Our strong project management structure has allowed us to work collaboratively, integrating all specialties and expertise required, transparently, in order to achieve the successful delivery of research.

### P487

#### Going green whilst maximising questionnaire response rates - Does size matter?

Tracey Davidson, John Norrie, Alison McDonald, Gramem MacLennan, Mohamed Abdel-Fattah
University of Aberdeen
**Correspondence:** Tracey Davidson
*Trials* 2017, **18(Suppl 1):**P487

This abstract is not included here as it has already been published.

### P488

#### Evaluating the effectiveness of remote versus on-site initiation visits: an embedded randomised controlled feasibility cluster trial within the SWIFFT trial

Caroline Fairhurst[1], Laura Jefferson[2], Stephen Brealey[2], Liz Cook[2], Garry Tew[3], Catherine Hewitt[2], Ada Keding[2], Izzy Coleman[2], Matt Northgraves[2], Amar Rangan[4]
[1]University of York; [2]York Trials Unit, University of York; [3]Department of Sport, Exercise and Rehabilitation, Northumbria University; [4]South Tees Hospitals NHS Foundation Trust, The James Cook University Hospital
**Correspondence:** Caroline Fairhurst
*Trials* 2017, **18(Suppl 1):**P488

**Background**
Delays in site set-up are a common problem in multi-centre randomised controlled trials. The frequency and format of contact with potential sites could play a role in reducing delays. Preliminary contact, prior to sites submitting for R&D approval, may involve liaising with healthcare professionals at the site to discuss the trial rationale and design, responding to queries, finalising local arrangements, and obtaining agreement to participate in the study. Such contact can be conducted as face-to-face, on-site meetings, or remotely via email, web or telephone correspondence. We sought to compare the effectiveness of remote versus face-to-face initiation, followed by a final on-site set-up meeting, on recruitment to the SWIFFT trial, and to inform the feasibility of undertaking such a comparison across other trials.
**Methods**
This cluster randomised, feasibility trial was a study within a trial (SWAT) embedded within the SWIFFT surgical trial.
The primary outcome was the number of patients recruited per site. Secondary outcomes included: time to (i) submission of R&D application, (ii) receipt of R&D approval, (iii) final on-site set-up meeting prior to starting recruitment, and (iv) first randomised participant; number of patients screened; the proportion of hospital forms and participant questionnaires returned; and the time to return these forms.
No formal power calculation was conducted, as the sample size was restricted by the number of sites approach to take part in the host trial. All sites were randomised (except the site of the Chief Investigator), and blinded to their involvement. Allocation was 1:1 via minimisation balancing on (i) whether the Principal Investigator had previous experience of working on a multi-centre surgical RCT, (ii) whether the site had a research nurse in place, and (iii) the size of catchment area (< vs > 500,000).
The main analyses used intention to treat. Site-level, time to event outcomes were compared between the trial arms using Cox proportional hazards regression, and recruitment outcomes were analysed via Mann-Whitney U tests. Return of questionnaires and time to return were analysed at the participant-level using logistic and Cox regression as appropriate accounting for clustering by site using robust standard errors (logistic) or a shared frailty (Cox).
**Results**
Thirty-seven sites were included (20 face-to-face and 17 remote), of which 33 (89%) opened to recruitment. The median number of participants recruited from sites allocated to receive on-site initiation was higher than from those allocated to remote initiation (10 (interquartile range 1.5 – 17) vs 6 (5 – 23)), though this difference was not statistically significant ($p = 0.79$). No statistically significant differences were observed in any of the secondary outcomes. There were four crossovers: 3 on-site to remote, and 1 remote to on-site.

#### Conclusion

In this feasibility trial, we found no evidence that face-to-face preliminary initiation of sites recruited to take part in a multi-centre RCT is more effective than remote contact on reducing set-up time, or improving recruitment or data collection. The cost of the two approaches will be explored.

### P489

### Reducing attrition: the communication of retention and withdrawal within patient information sheets

Anna Kearney[1], Anna Rosala-Hallas[2], Naomi Bacon[2], Anne Daykin[3], Alison J. Heawood[3], Athene Lane[3], Jane Blazeby[3], Mike Clarke[4], Paula R. Williamson[1], Carrol Gamble[1]
[1]North West Hub for Trials Methodology Research/University of Liverpool; [2]Clinical Trials Research Centre/University of Liverpool; [3]conduct-II Hub for Trials Methodology Research/University of Bristol; [4]Centre for Public Health, Queen's University of Belfast
**Correspondence:** Anna Kearney
*Trials* 2017, **18(Suppl 1):**P489

#### Background

The recruitment and retention of patients are significant methodological challenges for trials. Whilst research has focused on recruitment, the failure to retain recruited patients and collect outcome data can lead to additional problems of biased interpretation of results. Research to identify effective retention strategies has focused on influencing patient behaviour through incentives, reminders and alleviating patient burden, but little attention has been giving to exploring how retention is explained to patients at consent.

#### Aim

To assess how withdrawal, retention and the value of outcome data collection is described within Patient Information Sheets (PIS).

#### Methods

50 adult or parent PIS from a cohort of 75 National Institute of Health Research Health Technology Assessment (NIHR HTA) programme funded trials that started between 2009–2012 were obtained from protocols, websites or by contacting trialists. A checklist of PIS content developed from UK Health Research Authority and ICH GCP Guidelines was supplemented with retention specific questions. Corresponding trial protocols were obtained and evaluated to cross reference trial specific procedures with information communicated to patients.

#### Results

PIS frequently reiterated the patient's right to withdraw at any time (n = 49, 98%), without having to give a reason and without penalty (n = 45, 90%). However, few informed patients they may be asked to give a withdrawal reason where willing (n = 6, 12%). Statements about the value of retention were infrequent (n = 8, 16%). Consent documents failed to include key content that might mitigate withdrawals, such as the need for treatment equipoise (n = 3, 6%). Nearly half the trials in the cohort (n = 23, 46%) wanted to continue to collect outcome data if patients stopped trial treatment. However, in 70% (n = 33) of the trials using prospective consent, withdrawal was described in generic terms leaving patients unaware of the difference between stopping treatment and all trial involvement. Nineteen (38%) trials offered withdrawing patients the option to delete previously collected data.

#### Conclusions

Withdrawal and retention is poorly described within PIS and addressing this might positively impact levels of patient attrition, reducing missing data. Consent information is unbalanced, focusing on patient's rights to withdraw without accompanying information that promotes robust consent and sustained participation. Future research is needed to explore the whether the lack of retention information given at consent is impacting on attrition and if so, how retention can be described to patients to avoid concerns of coercion.

### P490

### Challenges faced during implementation of a surgical clinical trial

Julie Crof, Neil Corrigan, Vicky Liversedge
University of Leeds
**Correspondence:** Julie Croft
*Trials* 2017, **18(Suppl 1):**P490

Safari is a UK multi-site, parallel-group, randomised controlled, unblinded surgical trial investigating the use of the FENIX MSA (magnetic sphincter augmentation) device, as compared to the current standard treatment of SNS (sacral nerve stimulation) for adult faecal incontinence. Whilst the trial team were aware of the challenges and complexities associated with the design and implementation of surgical trials, a number of unanticipated issues arose during the set-up of safari which severely impacted the trial timelines to the point where funding of the trial was at risk. The issues experienced were related to funding, associated training and supply of the new FENIX device within the trial.

The new intervention, FENIX, provided a cost saving compared to SNS. For non-commercial research within the UK, treatment costs should be met through the normal NHS commissioning process. SNS is funded at a national level through NHS England as a specialised treatment, therefore FENIX should have been funded in the same manner. At the time of safari set-up, NHS England was a relatively new entity. It transpired there was no formal route for approving funding of FENIX other than the lengthy NHS England formal adoption process which could take up to 2 years, with no guarantee of success. Although a quicker alternative route was eventually identified and funding for FENIX confirmed, this caused significant delays as sites were not willing to proceed with set-up until this confirmation had been received.

A baseline level of experience for surgeons was set for both procedures to minimise any potential learning curve effect or bias. This was not an issue for SNS as this is an established procedure, however most participating surgeons did not have the required FENIX procedure experience at the outset. The trial set-up period was extended to allow time for surgeons to gain this required experience, in addition to incorporating a registration phase within the protocol to facilitate local approvals for use of a new device. Identification of dates for FENIX 'training cases' took much longer than anticipated and last minute cancellations were experienced e.g. Due to lack of beds or patients being unfit on the day of surgery.

Finally, an alternative process had to be implemented for supply of the FENIX device. FENIX is available in 7 different sizes with the required size only confirmed at the time of surgery. A set of different sized FENIX devices are therefore required at the time of each operation. Participating sites were reluctant to purchase a full set devices given that only one would be used during each operation. We secured an agreement from the device manufacturer that a set of devices would be provided to each site and remain the property of the device company, with payment only required for a successfully implanted device.

Further details on the issues faced during set-up of the safari trial and how these were resolved will be presented, in addition to reflecting on lessons learnt.

### P491

### Using graphical displays to monitor start-up and recruitment in clinical trials

Saams Joy, Robert Henderson, Nancy Prusakowski, Elizabeth A. Sugar, Janet T. Holbrook
Johns Hopkins Bloomberg School of Public Health
**Correspondence:** Saams Joy
*Trials* 2017, **18(Suppl 1):**P491

#### Background

Multi-center clinical trials are often plagued with slow starts due to delays getting sites started and slow recruitment. Frequently we assume we know the cause of the problems, e.g., slow reviews by multiple

Institutional Review Boards (IRBs) or restrictive eligibility requirements. However, there are often other factors, which we can control, that contribute to the delays. Information on performance can be presented in tables, but graphical displays are often useful to show patterns, as it is with outcome and safety data, to help identify modifiable factors that contribute long start-up times and slow recruitment as well as to plan for drug packaging, monitoring meetings, and close of recruitment.

**Methods**

We present a range of graphical methods for monitoring clinical center start-up and recruitment in clinical trials that may be useful for recognizing patterns in performance. Examples are provided from displays we have developed in two clinical trials consortium, the American Lung Associations Airways Clinical Research Centers (ALA-ACRC) and the Multicenter Uveitis Research group (MUST), as well as displays developed by others.

**Results**

We have developed and utilized a number of graphical tools that have been effective for identifying key logjams in the conduct of trials, some of which can be addressed by investigators once they have been identified. For example, a display of the stages of trial start-up along with the duration for each individual center identified that training and certification of clinic personnel contributed as much or more to delay in opening trials as IRB reviews. By distributing and discussing this graphic with investigators, we were able decrease start-up times at many sites. We also use a display of recruitment information that integrates information on two characteristics, total number of participants per site and the time since the last participant was recruited at each site. This display differentiates among sites that have recruited well in the in the past but have either stopped recruitment efforts or have run into difficulties finding more eligible patients versus sites that have fewer patients but are actively recruiting. Typically the graphics reporting recruitment emphasize totals without examining recent recruitment activity. Displays make the information easily accessible to all investigators and can serve to motivate investigators.

**Conclusions**

Assuming that we know what the obstacles to clinical trials start-up and recruitment without examining the actual data on these metrics, can be counterproductive. We have found that graphical displays can facilitate identification of problems, many of which can be addressed once investigators are made aware of the barriers.

### P492

#### Biological sample collection: considerations and lessons learnt at ICR-CTSU

Sarah Kernaghan, Leona Batten, Lynsey Houlton, Christy Toms, Claire Snowdon, Emma Hall, Judith Bliss
The Institute of Cancer Research Clinical Trials & Statistics Unit (ICR-CTSU)
**Correspondence:** Sarah Kernaghan
*Trials* 2017, **18(Suppl 1):**P492

**Background**

With advancements towards precision medicine and development of novel targeted agents to actionable mutations, collection of biological samples is a key component of many oncology trials. The use of samples for biomarker characterisation, to determine eligibility or as primary/secondary endpoints emphasises their integral nature to the outcome of the trial. Ensuring the quality and integrity of these is vital and at ICR-CTSU the development of procedures to improve sample collection, processing, transfer and storage is ongoing.

**Key considerations**

Sample collection should be considered at an early stage and funding applications should include sufficient funds for consumables, shipment and storage, and trial management time for central coordination. The principle use of samples and mandatory/optional collection requirements should be considered when developing procedures for ensuring sample integrity and evaluability. Prospective use (e.g. For eligibility in biomarker-driven trials) and type of sample required (e.g. Tumour biopsy vs blood sample) can add additional complexities and time constraints.

Liaison with sites in advance through feasibility assessment is important to determine their capability and resources to perform sample collection. During site set-up, training on sample collection procedures should be provided to research teams. If biopsy collection is required, engagement with relevant staff, e.g. Surgeons/radiologists, is key.

Early engagement with the central laboratory is important to ensure robust procedures are in place at the laboratory to maintain sample and trial integrity, through training and SOP development. A laboratory manual for sites is developed in collaboration with the central laboratory to ensure that instructions for sample processing and handling at sites and shipment methods are adequate.

Patient information sheets should describe provision, storage and future use of samples to ensure that the informed consent provided by patients is sufficient to cover future translational work.

**Issues and resolutions**

To maximise participation in sample collection studies, it is crucial that patient advocates are involved in study design and in developing sample collection schedules. This will ensure that sample donation is acceptable to patients and as simple as possible, for example using generic consent to collect research samples at the same time as diagnostic samples. Where possible diagnostic samples should be used, with bespoke additional research biopsies or other samples taken as required.

Sample quality may be compromised by delays in processing and lack of resources at site and an adaptable approach is often required to resolve these issues with sites, e.g. Re-training on collection and processing procedures if necessary, provision of additional resources if feasible.

To optimise potential recruitment in multi-centre trials, ICR-CTSU acts as the key point of contact between the site and central laboratory when real-time testing of samples is required. Discrepancies between information provided by sites and the central laboratory regarding samples collected do occur, and sample reconciliation is conducted throughout the duration of a trial to identify issues at an early stage.

**Conclusion**

Sample collection in clinical trials is becoming increasingly important, but introduces logistical issues. ICR-CTSU constantly works to resolve these issues, and best practice is shared across the unit to minimise repeating problems.

### P493

#### Citation of articles published in clinical trials: design articles vs. others

Barbara Hawkins[1], Roberta W. Scherer[2]
[1]The Johns Hopkins University; [2]The Johns Hopkins Bloomberg School of Public Health
**Correspondence:** Barbara Hawkins
*Trials* 2017, **18(Suppl 1):**P493

**Background**

Policies regarding publication of articles that describe design features of an individual trial in journals of the Society for Clinical Trials have varied by editor. In 2004, the editor of Clinical Trials specified that such manuscripts must be "instructional" in order to be considered for publication in the Society's new journal. Subsequently, authors of design manuscripts submitted to the journal frequently received a letter that referred to the editorial, reviewed the requirements, and provided good examples. As review and publication of design articles typically require expenditure of as much or more resources as other types of articles, a question of interest is whether the frequency of citation of design articles has been similar to frequency of citation of articles of other types.

**Methods**

All issues of Clinical Trials published from 2004 through 2015 were searched to identify design and all other articles. We excluded papers published as part of proceedings of meetings, letters, editorials, invited commentaries, columns, etc. For our preliminary estimates of citation frequency, we randomly selected 20 articles of each type. We searched the Web of Science and Google Scholar databases to determine the number of citations per article as of October 31, 2016. We summarized the distribution of number of citations for each article type and each citation database by the median and interquartile range (IQR).

**Interim Results**

Of the 598 articles published in Clinical Trials in the 12-year period, 80 (13.4%) were design articles. The 20 design articles selected randomly had been cited from 0 to 58 times per Web of Science for a median of 11.0 citations per article (IQR: 7–20) and 5 to 91 times per Google Scholar for a median of 17.0 (IQR: 9–41). The 20 "other" articles selected randomly had been cited 9 to 49 times per Web of Science (median: 17.0; IQR: 4–18) and 2 to 93 times per Google Scholar. (median: 24.6; IQR: 7–30).

**Current Status**

We have begun to search for citations in the Scopus database for comparison with citation counts from the other two citation databases for all 598 articles. We also plan to compare the two types of articles for self-citation patterns, distributions of time from publication to citation, and other citation metrics.

**Interim Conclusion**

Based on findings for the random samples of 20 articles of each type, design articles published in Clinical Trials during 2004 to 2015 have been cited as frequently as other articles published in the journal during the same time period based on citations in the Google Scholar database but have been cited somewhat less frequently according to citations in the Web of Science database. Final conclusions await completion of data collection and analysis of all 598 articles.

### P494

#### An investigation of the methods used to design, analysis and quantify non-inferiority margins in four medical journals in a 12 month time period

Enass M. Duro
University of Sheffield
*Trials* 2017, **18(Suppl 1):**P494

**Background**

Studies with a non-inferiority (NI) objective have become more popular since the 1990s. A NI study is designed are used to demonstrate that the new treatment is not worse than the proven active comparator. There are methodological and regulatory challenges associated with the planning, conducting and interpretation of these studies and there are a number of regulatory guidelines. The main aim of this review was to investigate the design, analysis; interpretation and reporting of non-inferiority trials in the four top medical journals (Lancet, BMJ, JAMA, The New England Journal of Medicine) in accordance with CONSORT statement.

**Methods**

A search for Non-inferiority trials in Pub Med database that published between 1/1/2015 and 31/12/2015 was performed. The inclusion criteria were; Non-inferiority trials that were randomised clinical trials, done on adult humans, published in English and with the full text available. From this search, 387articles were retrieved. Only 45 articles published in the Lancet, BMJ, JAMA and NEJM, 37 of them were analysed.

**Results**

Of 37 articles included in analysis, 15 were published in The Lancet, 12 in the New England Journal of Medicine, 5 in BMJ and 5 in JAMA. According to the source of funding: 18(48.6%) of the trials were publicly funded, pharmaceutical companies funded 15(40.5%), and in 4(10.8%) trials, the funding was a combination of public and private sectors. All of the trials were multicentre trials. With respect to the blinding; 24 (64.9%) of the studies were open label studies (no blinding); in these open-label trials, 14 (58.3%) blinding was not possible, no specific reason was giving for non-blinding in the other 10(41.7%) trials. Only 8 (21.6%) of the trials were double blinded with 5(13.5%) single blinded. Phase III trials were the most common types of trials - 24 of the 37 (64.9%) while 3(8.1%) were phase IV trials, and 1(2.7%) was a phase II trial. The phase of the trial was not provided in 9 (24.3%) trials. All the trials reported their NI margin. The methods for determining NI margin were not clear in 5(13.55%) trials. In 13(35.1%) trials the margin calculated by the investigators based on previous studies, in 12(32.4%) trials the NI margins Based on both clinical judgment and historical trials, in 5(13.5%) the NI margins based on the regulatory guidelines, and in 2(5.4%) trials the NI margin based on clinical judgment only. Regarding the conclusion, non-inferiority established in 24(64.9%) trials, 8 (21.6%) trials failed to establish the non-inferiority, 5(13.5%) trials conclude the superiority of the tested drug compared with the active control.

**Conclusion**

Most of published NI trials in the four journals did not follow the regulatory guidelines regarding conduct and interpretation of NI trials. There is a need to improve the conduction, interpretation and inference of published NI trials.

### P495

#### Implementation of strategies to improve collection of a challenging laboratory outcome in the delivery room

Steven Weiner
George Washington University
*Trials* 2017, **18(Suppl 1):**P495

**Background**

In a multi-center obstetric randomized trial, a key outcome was a laboratory value not routinely collected for clinical reasons at most centers. We determined early in recruitment that a substantial proportion of these results were being missed. We describe the steps implemented to decrease these missing results, and some measures of their effectiveness.

This trial required collection of paired umbilical cord blood samples (artery and vein) for measurement of gases. Collection in the delivery room and testing by the hospital laboratory are time-sensitive, logistically challenging, and require the cooperation of clinical staff with competing priorities. Drawing blood from the correct vessel is challenging, testing must be completed promptly, and there is little chance for a second opportunity if there is any error. Meanwhile, clinical staff are focused on two patients – mother and baby. Therefore, our strategies focused on minimizing non-research staff participation where possible.

**Results**

After recruiting 3% of the participants, we realized that cord blood gases were missed for 20% of deliveries. Missed, incomplete, or erroneous results occurred even in centers where pre-trial collection was routine. Three successive strategies were implemented to improve this rate. The first was alerting the staff. With each center aware of its individual rate and receiving regular updates, unique center-initiated strategies were implemented. Research staff made collection a higher priority, often being present in the delivery room, hand-carrying specimens to the laboratory, troubleshooting with laboratory staff, and occasionally drawing the blood themselves. A video was distributed showing proper technique for drawing the sample. Next, centers were offered a point-of-care blood testing device, which allowed research staff to perform the test with a smaller volume and without involving the laboratory. It also facilitated repeat testing if needed. Lastly, additional funds were provided for after-hours staffing, making it more likely research staff could be present at delivery and facilitate sample collection. After each time point, improvements were observed. The missed rate decreased to 11%, then 9%, and 7.5%, respectively, after each of the three strategies were implemented.

Not all centers implemented these strategies, nor implemented them at the same time. However, we will describe the rates before and after, including a subset who did not use the point-of-care device nor extended their staffing coverage. Results varied; however in the final year when all strategies were implemented, centers that used both a point-of-care machine and extended hours achieved 3 percentage points fewer missed results than those who implemented neither. The improvement was also noted beyond the trial. Trial-collected quality control data were provided to the clinical departments, which encouraged them to improve collection procedures for all patients.

### Conclusion

When laboratory outcomes in a hospital setting are essential to a clinical trial, systems that rely less on clinical staff are advantageous. These strategies could apply to other settings in clinical research, such as emergency departments or remote-care locations. Furthermore, the systematic collection and review of data quality measures within a trial can provide useful information for clinical care outside the research setting.

## Oral Presentations

### O1

#### DSMB monitoring of the therapeutic hypothermia after pediatric cardiac arrest (THAPCA) trials

Richard Holubkov[1], Amy Clark[1], Andrew M. Atz[2], David Glidden[3], Beth S. Slomine[5], James R. Christensen[5], Angie Webster[1], Kent Page[1], J. Michael Dean[1]
[1]University of Utah School of Medicine; [2]Medical University of South Carolina; [3]University of California at Los Angeles; [4]University of California San Francisco; [5]Kennedy-Krieger Institute
**Correspondence:** Richard Holubkov
*Trials* 2017, **18(Suppl 1):**O1

The NIH-funded THAPCA randomized trials compared efficacy of therapeutic hypothermia (target temperature 33 degrees C) to normothermia (temperature 36.8 degrees) after cardiac arrest in children >48 hours to <18 years of age. One trial enrolled children who sustained out-of-hospital arrest (target n = 250 analysis-eligible), and a second trial enrolled children with unplanned in-hospital arrest (target *n* = 504 due to smaller expected hypothermia benefit). The primary efficacy outcome was survival with favorable neurological outcome 1 year post arrest. Due to key differences in arrest etiology and post-arrest outcomes, the parallel trials were monitored separately.
An NIH-appointed DSMB monitored safety and efficacy of both trials. A charter specified meetings approximately twice yearly. Symmetric O'Brien-Fleming boundaries were used for monitoring treatment superiority, with an informal <20% conditional power criterion specified for declaring futility.
Recruitment began in September 2009. The first safety-only DSMB review in 2010 combined adverse outcome data across trials as event numbers were small. Subsequent study-specific, safety-only DSMB reviews occurred until the first interim efficacy look for both trials in November 2012.
In the out-of-hospital trial, a treatment difference was noted among 141 children with 1-year outcomes available, but the treatment comparison (nominal p-value = 0.03) did not approach the conservative stopping boundaries. Enrollment continued, with n = 250 target surpassed at 2012 year-end. A second interim analysis in August 2013 (*n* = 213) found that the previously observed treatment difference diminished (nominal p = 0.12). Results of the 'negative' out-of-hospital trial were published in 2015.
The in-hospital trial encountered enrollment difficulties due to below-expected patient volumes. During the November 2012 interim analysis, for which 74 children had one-year outcomes available, no treatment difference was noted. Due to the slow accrual, the DSMB skipped efficacy monitoring during the 2013 meeting. The second efficacy analysis, in March 2014 (*n* = 149), again did not approach statistical significance. By late 2014, despite extended enrollment and addition of centers, only 254 eligible patients had been enrolled, indicating final enrollment would be substantially below target. Given these circumstances, the DSMB elected to consider stopping for futility during their January 2015 meeting. Conditional power for the primary efficacy outcome was found to be very limited under a range of assumed treatment effects and realistic final enrollment numbers ranging from 300–400. However, the DSMB decided to continue enrollment and reconvene after additional information about potential utility of the trial was available. In a supplemental February 2015 meeting, the DSMB reviewed conditional power estimates for exploratory outcomes, some ad hoc with postulated treatment effects based on out-of-hospital trial trends, and subsequently recommended stopping enrollment. In-hospital trial results are under journal review as of November 2016.
The THAPCA trials provide an interesting study of long-term DSMB review of two parallel studies with differing enrollment patterns. For example, the DSMB elected to remain masked to treatment arm identity in both trials throughout all reviews, recognized the marginal benefit of repeated efficacy monitoring under slow enrollment, and considered implications of futility stopping beyond the primary trial outcome. Our experience may help optimize strategies for successful DSMB involvement in randomized trials with long-term follow-up.

### O2

#### Review of pilot and feasibility studies from a registry website; an overview of recent practice

Nicola Totton, Andrew Brand, Rachel Evans, Zoë Hoare, Nia Goulden, Paul Brocklehurst
Bangor University
**Correspondence:** Nicola Totton
*Trials* 2017, **18(Suppl 1):**O2

##### Background

Pilot and feasibility studies are increasing in popularity, as demonstrated by the launch of the focussed Journal of Pilot and Feasibility Studies in January 2015. Pilot studies are defined as a mini replication of a proposed full study, used to identify any potential issues. A feasibility study is similar but considered as more exploratory and is used to judge the suitability of a proposed study by assessing the viability of each of the elements individually. In this review, we aim to assess the prevalence of pilot and feasibility studies in recent practice and determine whether the two differ on key characteristics. We envisage this work will then guide future research into the use of pilot and feasibility studies to better inform full studies.

##### Methods

Data on pilot and feasibility studies have been collected from the International Standard Randomised Controlled Trial Number (ISRCTN) registry website. As the website does not provide a downloadable database, a web scraping methodology was adopted and implemented using the R programme. Pilot and feasibility studies were identified using a keyword search on the main title. Unique trial records were extracted using regular expressions and collated in an Excel spreadsheet.

##### Results

Out of the 658 unique trials that were found within the ISRCTN database, 456 (69%) of these were labelled as pilot studies, 196 (30%) as feasibility studies and six (1%) considered both a pilot and feasibility study. The overall prevalence of all pilot or feasibility studies does not seem to be showing any particular trend over time. Initially, pilot studies were much more common; however, in recent years feasibility studies have increased in popularity and are now on a par with pilot studies. The median number of target participants for each trial was 50 (range: 5–130,000). When considered separately, pilot studies had a target of 44 (range: 5–130,000) with feasibility studies being slightly larger with a target of 60 (range: 6–1,600).
On average, the trials ran for a period of 18 months with pilot studies reporting a slightly shorter time (17 months) than feasibility studies (18.5 months), which aligns with the larger sample size target.
Mental and behavioural disorders accounted for the highest proportion of studies (20%), followed by cancer studies (13%).
The majority of the studies within the data were interventional (96%) and defined as randomised controlled trials (85%). A hospital environment is the most likely setting for the studies (45%) and in most cases, the recruitment was in a single country (97%). This is consistent between the pilot and feasibility studies within the database.

##### Conclusions

In recent years, the prevalence of feasibility studies has begun to match that of pilot studies but the characteristics found for the two are very similar. Due to their similar nature, there could be an argument that pilot and feasibility studies should considered as one. Alternatively, if these studies are to be separate more education is required to outline the differences and provide guidelines for each to help shape future research projects.

### O3

#### Using routinely recorded data in a clinical trial: the feasibility, agreement and additional benefits compared to standard prospective data collection methods

Graham Powell[1], Laura Bonnett[1], Catrin Tudur-Smith[1], Dyfrig Hughes[2], Tony Marson[1], Paula Williamson[1]
[1]University of Liverpool; [2]Bangor University
**Correspondence:** Graham Powell
*Trials* 2017, **18(Suppl 1):**O3

There are a number of administrative datasets that routinely record information on individuals in the UK. Such routine or 'Big Data' sources record data for a specified primary purpose and are regulated for security, confidentiality and disclosure by The Data Protection Act 1998 and Freedom of Information Act 2000. Access for secondary uses such as clinical research is permitted when health and social care benefit can be demonstrated. Routine sources include clinical datasets such as Hospital Episode Statistics (HES) and the Clinical Practice Research Datalink (CPRD) and non-clinical datasets such as data recorded by the Department of Work and Pensions (DWP), HM Revenue and Customs (HMRC) and The Driving and Vehicle Licensing Authority (DVLA).
Routinely recorded data used in clinical research is potentially cost and resource-use effective. Clinical sources of data such as HES and CPRD are commonly accessed to provide data for retrospective observational and record-linkage studies and provide a valid dataset for this purpose. Routinely recorded data may also present advantages to prospective clinical research such as randomised controlled trials (RCTs). For example, routinely recorded data have been used to inform judgements about the feasibility of sample size and recruitment targets and measure participant outcomes. Pragmatic cluster RCTs have even been conducted through routine data sources including patient recruitment, randomisation, administration of intervention and trial assessments, such as through The Clinical Practice Research Datalink (CPRD). The majority of RCTs incur health service costs as clinicians assess participants, record outcomes and complete Case Report Forms (CRFs) - hence using routinely recorded data may provide an efficient alternative method for data collection in addition to reducing the burden on participants. Furthermore, data from non-clinical routine sources may inform outcomes beyond the standard RCT assessments of clinical efficacy and effectiveness. For example, cost data (such as use of healthcare resources) and socio-economic data (such as employment and means-tested benefits data) may inform health economic analyses and the assessment of the broader societal impact of healthcare interventions.
However, limitations with accuracy of coding, confidentiality, ownership and access have previously been identified as significant barriers to accessing routinely recorded data for prospective research.
This study has assessed the feasibility of accessing both clinical and non-clinical routinely recorded data within the context of a RCT and will assess the agreement and additional benefits of routinely recorded data compared to data collected using standard prospective methods in a RCT.

### O4

#### Using re-randomisation in clinical trials to increase patient recruitment

Brennan Kahan
Queen Mary University of London
*Trials* 2017, **18(Suppl 1):**O4

##### Background
Patient recruitment is often a major challenge for randomised trials. Reviews of publicly funded UK trials have found that 45 to 69% fail to recruit to target. This increases costs, delays results, and adversely impacts on the feasibility of conducting trials for conditions where there is a limited patient pool.
For some conditions, patients may require treatment on multiple occasions. For example, patients with sickle cell disease require pain relief for each new pain crisis, and women using fertility treatment may undergo multiple treatment cycles until they become pregnant. The current norm for trials in these conditions is for patients to be enrolled for only one treatment episode.
An alternative approach is the re-randomisation design. This design allows patients to be re-enrolled and re-randomised for each new treatment episode. The number of times each patient is re-randomised is determined by the number of treatment episode each patient experiences, rather than by the trial design. Because each patient can contribute multiple treatment episodes, this design can facilitate an increased recruitment rate, potentially making trials easier and quicker to conduct.
#### Methods
We describe some properties of the re-randomisation design, such as the conditions required to obtain unbiased estimates of the treatment effect and control type I error rate. We also evaluate the likely impact of re-randomisation on the recruitment rate is several clinical areas.
#### Results
The re-randomisation design can provide unbiased estimates of treatment effect and control the type I error rate under some very simple conditions. Furthermore, in some instances this design will have the same or even higher power than a parallel group with an equivalent number of observations. Based on a modelling study across three different clinical areas, we estimated that re-randomisation could reduce the time to complete recruitment between 19-45%.
#### Conclusions
The re-randomisation design can increase the recruitment rate compared to parallel group designs, which could reduce costs and make trials more feasible to conduct. If used appropriately, it can provide unbiased estimates of treatment effect, control the type I error rate, and maintain or even increase power compared to a parallel group design.

### O5

#### Adaptive non-inferiority margins under observable non-constancy

Brett Hanscom[1], Deborah Donnell[1], Brian Williamson[2], James P Hughes[2]
[1]Fred Hutchinson Cancer Research Center; [2]University of Washington
**Correspondence:** Brett Hanscom
*Trials* 2017, **18(Suppl 1):**O5

##### Background
A central assumption in the design and conduct of non-inferiority (NI) trials is that the active-control therapy will have the same degree of effectiveness in the planned non-inferiority trial as it had in the prior placebo-controlled trials used to define the non-inferiority margin. This is referred to as the 'constancy' assumption. If the constancy assumption fails, the chosen non-inferiority margin is not valid and the study runs the risk of either approving an inferior product or failing to approve a beneficial product. The constancy assumption is unlikely ever to be met completely in practice, and it cannot be validated in a trial without a placebo arm. However, it is often the case that there exist strong, measurable predictors of constancy, such as dosing and adherence. Such predictors can be used to identify appropriate non-inferiority margins during the planning phase, as well as adjust the margin during the monitoring and analysis phases.
#### Methods
We propose using meta-analysis regression to model the association between population characteristics and the effectiveness of an active-control therapy, and assume that the model provides an unbiased estimate of effectiveness. Together with expected population characteristics, the fitted model parameters are used to specify a non-inferiority margin targeted to the planned study population. During interim and final analyses, observed population characteristics are used in combination with the initial meta-regression results to adjust the margin. Two methods of adjustment are proposed: one that maintains a pre-planned minimal clinically important difference (MCID) over an inferred placebo, and a second that maintains a fixed proportion of the estimated active-control benefit over placebo. In the second scenario, sample size adjustment may be necessary.
#### Results
We consider a hypothetical NI trial of an experimental HIV Pre-exposure Prophylaxis (PrEP) drug versus a standard PrEP drug (active control)

designed to provide one-sided alpha equal to 2.5%, and 90% power. If, due to lack of adherence to the standard drug, the constancy assumption fails and the active-control therapy is 10% less effective than planned, the probability of a false non-inferiority finding rises from 2.5% to 16%. If the active control therapy is 10% more effective than planned (for example, if adherence were higher than planned), power falls from 90% to 52%. By revising the NI margin according to the pre-specified meta-regression model, and maintaining the pre-specified MCID, both alpha and power can be corrected to planned levels without modification to the planned sample size. If the allowable effectiveness of the experimental therapy is permitted to vary depending on the estimated active-control effect, alpha and power can be partially corrected by updating the margin, and fully corrected by updating both the margin and the sample size.

**Conclusion**

If prior placebo-controlled trials provide evidence of an association between population characteristics and the effectiveness of an active-control therapy, non-inferiority margins can be adjusted based on observed population features, effectively maintaining pre-specified levels of Type-I error and power.

### O6

#### A revised tool for assessing risk of bias in randomized trials (RoB 2.0)

Jalena Savovic[1], Matthew Page[2], Roy Elbers[2], Asbjorn Hróbjartsson[3], Isabelle Boutron[4], Barney Reeves[2], Jonathan Sterne[2], Julian Higgins[2]
[1]University of Bristol; NIHR CLAHRC West University Hospitals Bristol NHS Foundation Trust; [2]University of Bristol; [3]University of Southern Denmark; [4]University Paris Descartes
**Correspondence:** Jalena Savovic
*Trials* 2017, **18(Suppl 1):**O6

**Background**

The Cochrane risk of bias tool for randomized trials seeks to determine whether the findings of a randomized trial can be believed. First released in 2008, and revised slightly in 2011, it is the most widely used risk of bias tool in both Cochrane and non-Cochrane reviews on the effects of interventions. However, evaluations of the tool have highlighted some problems. Objective: To introduce a revised tool to assess risk of bias in randomized trials (RoB 2.0), which builds on the established Cochrane risk-of-bias tool as well as the thinking behind the recently developed tool for non-randomized studies (ROBINS-I).

**Methods**

Over the last year, we assembled collaborators from across the world to develop RoB 2.0. We held an initial development meeting in August 2015 where the main structure of the tool was agreed. Working groups were formed and tasked with developing signalling questions, criteria for reaching a judgment and full guidance. Working groups' contributions were collated and the draft version of the new tool was extensively piloted by individuals with varying degrees of experience, at a three-day event held in Bristol in February 2016 and remotely. The piloting feedback was considered at a second development meeting in April 2016, where refinements to the tool and to the written guidance that accompanies it were made. The working groups were further tasked with developing algorithms for reaching a domain-level judgment and creating worked examples. Further pre-release piloting took place in September 2016.

**Results**

Key changes in RoB 2.0 compared with the 2011 version of the tool are: – simplification of issues into fewer (mandatory) bias domains; – clearer focus on risk of bias in a specific result from the randomized trial; – introduction of signalling questions - which are reasonably factual in nature - to facilitate risk-of-bias judgements; – algorithms to reach risk of bias judgements; – clarification of differences between the review team's interest on the effect of assignment to intervention (the intention-to-treat effect) versus the effect of starting and adhering to intervention: issues of blinding, implementation and adherence differ importantly between these; – clarification that selective reporting should be assessed only when a result is available (whereas selective non-reporting should be assessed at meta-level); – separate templates for parallel group trials, cluster-randomized trials and cross-over trials.

**Conclusions**

We believe the new tool will offer considerable advantages over the existing tool. Once programmed into software, we expect the tool will be easier to use than the first version. Some issues remain to be resolved, however, such as how many results should be assessed for each study, and how best to integrate the assessment into the data extraction process. This presentation will provide an introduction to the tool. Further details of RoB 2.0 will be available from riskofbias.info.

**Funding**

MRC Network of Hubs for Trials Methodology Research (MR/L004933/1- N61); MRC ConDuCT II (MR/K025643/1), NIHR CLAHRC West.

### O7

#### Simulation of various strategies for optimal selection of randomization methods in multicenter clinical trials

Zhibao Mi, Rebecca A. Horney, Eileen M. Stock, Kousick Biswas
VA Cooperative Studies Program Coordinating Center
**Correspondence:** Zhibao Mi
*Trials* 2017, **18(Suppl 1):**O7

The random allocation and masking of participants to treatment are procedures in a study design essential to minimizing bias and the success of a clinical trial. The essence of the randomization process is to ensure an equal probability for each participant to be assigned to active or control treatment groups, which naturally leads to three properties of the randomization procedure: balance in sample size and constitution between treatment and control groups, unpredictability in the allocation of a participant to a certain group, and simplicity for an investigator to implement without compromising the randomization principle. With recent advances in biomedical and statistical methodologies, the area of clinical trial design is evolving rapidly with varying opinions on an optimal randomization method. Randomization methods have now expanded into more advanced approaches beyond the classical assignment to treatment groups (e.g. covariate adjustment in the randomization procedure, changing probability of assignment as in adaptive designs). Thus, the traditional randomization method faces new challenges, both theoretically and pragmatically. With increasingly complex trial designs, it becomes more challenging to determining the most appropriate randomization method. To help select an optimal randomization method, we performed numerical simulations to assess various randomization strategies in a centralized randomization system for multicenter clinical trials, by considering varying values for several design parameters including trial sample size, covariate strata, clinical sites, treatment arms, and allocation schema. For each scenario of simple, permuted block, stratified permuted block, and adaptive randomization strategies, imbalance and predictability were estimated through numerical simulations (repeated 10,000 times). Simulation results are tabulated in a series of tables serving as a useful reference for choosing an appropriate randomization method given a particular trial design. The goal of this study is to provide data support for identifying an optimal randomization method, accounting for the trade-off between precision of randomization balance and simplicity of implementation since more complex methods may lead to a greater likelihood of randomization schedule or allocation algorithm errors during implementation and human errors during the trial.

### O8

#### Should consort do more to improve the quality of missing data reporting in trials?

Jamilla Hussain[1], Martin Bland[2], Dean Langan[3], Miriam J Johnson[4], David C Currow[5], Ian R White[6]
[1]Hull York Medical School, University of York; [2]Health Sciences Department, University of York; [3]University College London; [4]SEDA, University of Hull; [5]Palliative and Supportive Services, Flinders University; [6]MRC Biostatistics Unit, University of Cambridge
**Correspondence:** Jamilla Hussain
*Trials* 2017, **18(Suppl 1):**O8

**Background**
Transparent reporting of missing data is crucial to the critical appraisal of trial results. This is particularly important in palliative care trials where large amounts of missing data and truncated data due to death occur. Although the CONSORT 2010 statement recommends the impact of missing data on the validity of intention-to-treat analyses be reported, it does not provide specific guidance on how to report: methods to handle missing data, assumptions about the missing data mechanism and missing data sensitivity analyses. Several other groups have provided further missing data reporting recommendations that include such criteria. Whether trials report missing data according to the recommendations by CONSORT and other groups however is not known.
**Objectives**
Assess (i) the quality of reporting and handling missing data in palliative care trials against current guidance, (ii) any differences in the complete reporting of criteria specified by the CONSORT 2010 statement compared to those not specified by CONSORT, (iii) the association between the quality of missing data reporting and journal impact factor and CONSORT endorsement status, to explore how implementation of reporting guidance may be optimised.
**Methods**
Systematic review of palliative care randomised controlled trials. An information specialist searched CENTRAL, MEDLINE, and EMBASE (2009–2014) with no language restrictions. A random sample of identified trials were screened, selected and had data extracted by two independent reviewers.
**Results**
108 trials (15,560 participants) were included. In general missing data reporting was incomplete and not handled in accordance with current guidance. Reporting criteria specified by the CONSORT statement were better reported than those not specified by CONSORT (proportion of trials reporting CONSORT criteria: account for all participants who enter the study 69%, data completeness 94%, reason for missing data 71%). However item-level (15%) and secondary outcome (9%) missing data were poorly reported, so the proportion of missing data stated is likely to be an underestimate. Provided reasons for missing data were unclear for 54% of participants. 48% of trials clearly reported their method to handle missing data, of the trials with missing data (n = 93): 60% used complete case analysis alone and 16% reported a missing data sensitivity analysis. Only one trial used a recommended method to handle truncated data due to death. As the journal impact factor doubled the odds of reporting the flow of participants (odds ratio (OR) 1.54, 95% CI 1.20, 1.97), data completeness (OR 1.39, 95% CI 1.15, 1.69), comparison of baseline characteristics of those with and without missing data (OR 1.50, 95% CI 1.20, 1.87), and method of handling missing data (OR 1.40, 95% CI 1.13, 1.73) were statistically significantly increased. There was insufficient evidence that the criteria specified by CONSORT were more likely to be reported in journals that endorsed the CONSORT statement.
**Conclusion**
The rigorous methods, evolving nature, and wide recognition of the CONSORT statement make it ideally placed to facilitate better reporting of missing data. Further development and implementation of the CONSORT missing data reporting guidance is likely to improve the quality of reporting. Specific suggestions for CONSORT will be discussed.

### O9

#### Using outcomes to analyze patients rather than patients to analyze outcomes: partial credit, pragmatism, and benefit: risk evaluation

Scott Evans
Harvard University
*Trials* 2017, **18(Suppl 1):**O9

In the future, clinical trials will have an increased emphasis on pragmatism, providing a practical description of the effects of new treatments in realistic clinical settings. Accomplishing pragmatism requires better summaries of the totality of the evidence that allow for informed benefit:risk decision-making and in a way that clinical trials consumers, patients, physicians, insurers find transparent. The current approach to the analysis of clinical trials is to analyze efficacy and safety separately and then combine these analyses into a benefit:risk assessment. Many assume that this will effectively describe the impact on patients. But this approach is suboptimal for evaluating the totality of effects on patients. We describe a broad vision for the future of clinical trials consistent with increased pragmatism. Greater focus on using outcomes to analyze patients rather than patients to analyze outcomes particularly in late-phase/stage clinical trials is an important part of this vision. We discuss partial credit, a strategy for design and analysis of clinical trials based on benefit:risk assessment that has greater pragmatism than standard methods. The strategy involves utilizing composite benefit:risk endpoints with a goal of understanding how to analyze one patient before trying to figure out how to analyze many. With a desire to measure and weigh outcomes that are most important from the patient's perspective, we engage patients as a resource to inform analyses. We discuss partial credit within the context of antibiotic clinical trials.

### O10

#### Regression discontinuity for replication of randomized controlled trial results: an application to the mycotic ulcer treatment trials

Catherine Oldenburg[1], N. Venkatesh Prajna[2], Tiruvengada Krishnan[2], Revathi Rajaraman[2], Muthiah Srinivasan[2], Kathryn J. Ray[1], Kieran S. O'Brien[1], Travis C. Porco[1], Nisha R. Acharya[1], Jennifer Rose-Nussbaumer[1]
[1]University of California, San Francisco; [2]Aravind Eye Hospitals
**Correspondence:** Catherine Oldenburg
*Trials* 2017, **18(Suppl 1):**O10

**Background**
Regression discontinuity (RD) is a quasi-experimental method that utilizes threshold rules for determining treatment assignment to estimate causal effects when randomization is not available. Examples of clinical threshold rules include CD4 count for determining antiretroviral therapy eligibility in HIV-infected individuals or blood pressure determining eligibility for hypertension treatment. However, the validity of RD has not been established via direct comparison to effects estimated via the gold standard randomized controlled trial (RCT). Here, two concurrent RCTs allow us to directly compare an effect size from an RCT to that of RD. We utilize a continuous enrollment criterion in the RCTs to test if regression discontinuity achieves similar results to the intention-to-treat (ITT) effect from the trial itself.
**Methods**
The Mycotic Ulcer Treatment Trials (MUTT-I & MUTT-II) were two contemporaneous randomized controlled trials with identical outcome assessments designed to compare strategies for the treatment of fungal corneal ulcers. MUTT-I, which enrolled patients with better best spectacle-corrected visual acuity (BSCVA) (<20/400), compared topical voriconazole to topical natamycin. MUTT-II enrolled patients with worse BSCVA (20/400) and compared adding oral voriconazole versus placebo to topical voriconazole. We estimated the RD effect of natamycin versus voriconazole on 1) 3-month BSCVA and 2) odds of perforation and/or requiring a therapeutic penetrating keratoplasty (TPK), and compared these results to those estimated in the trial. We utilized enrollment visual acuity as a clinical decision rule to replicate the results of MUTT-I, using the natamycin arm from MUTT-I and the placebo arm of MUTT-II, and 20/400 as the threshold for receiving natamycin (<20/400) or voriconazole (20/400), representing an RD design. The RD model included terms for being above or below the threshold and a term for baseline visual acuity above and below the threshold.
**Results**
In the MUTT-I RCT, patients randomized to natamycin had a nearly 2-line improvement in BSCVA at 3 months (logMAR −0.18, 95%CI −0.30 to −0.05) and reduced odds of perforation and/or TPK (OR = 0.42, 95%CI 0.22 to 0.80) compared to voriconazole. In the RD model, natamycin was associated with a three-line improvement in BSCVA at 3 months (logMAR −0.29, 95%CI −0.50 to −0.08) and reduced odds of perforation/TPK (OR = 0.35, 95%CI 0.15 to 0.82). Results were robust to multiple sensitivity analyses, including flexible functional forms of visual acuity and restricting the analysis to narrower bandwidths around the cutoff (20/400).

**Conclusions**

While RD and RCT results were similar, the RD effect was larger, although not significantly so (P = 0.52 for BSCVA). These results suggest that the use of threshold rule in an RD design may be useful for estimation of causal effects under conditions where trials are not possible, or for replication of trial results.

### O11

#### Evaluation of failure time surrogate endpoints in individual patient data meta-analyses of randomized clinical trials. A poisson approach

Federico Rotolo[1], Xavier Paoletti[1], Toasz Burzykowski[2], Marc Buyse[2], Stefan Michiels[1]
[1]Gustave Roussy/INSERM; [2]I-Biostat U Hasselt/IDDI
**Correspondence:** Federico Rotolo
*Trials* 2017, **18(Suppl 1):**O11

Surrogate endpoints are often used in randomized clinical trials instead of well-established hard endpoints for practical convenience: they are usually cheaper, more rapid, or less invasive to measure. The meta-analytic approach relies on two measures of individual level surrogacy (R_indiv^2) and trial level surrogacy (R_trial^2) [1]. This approach was extended to the survival data case [2], with a first step using copulas to measure individual level surrogacy in terms of Kendall's tau and a ?A3B2 show $132#?>second step using weighted regression to compute R_trial^2. Despite being the reference method for survival data today, this approach can suffer from convergence problems in the second step, which is the one which computes R_trial^2. In the present work, we considered a bivariate survival model with (i) an individual random effect shared between the two endpoints to measure individual level surrogacy (Kendall's tau) and (ii) correlated treatment-by-trial interactions to measure R_trial^2. We used auxiliary mixed Poisson models to jointly estimate the parameters of such model with piecewise constant baseline hazards. To reduce the computational complexity, we also considered reduced Poisson models, accounting for only individual- or only trial-level surrogacy. We studied via simulations the operating characteristics of this mixed Poisson approach as compared to the two-step copula approach, with Clayton, Plackett and Hougaard copulas and with or without adjustment of the second-step regression for measurement error. The Clayton copula model was the most robust and reliable of the copula models compared; the Poisson model with both individual- and trial-level random effects outperformed its reduced equivalents. We also applied the methods to an individual patient data meta-analysis in advanced/recurrent gastric cancer (4069 patients from 20 randomized trials). As the convergence rate and the estimation results may vary substantially between models, we encourage the user to carefully evaluate the convergence of each alternative approach and to report the results of different models. We implemented the methods presented here in the R package surrosurv (https://cran.r-project.org/package=surrosurv).

### O12

#### Influence of peer review on the reporting of primary outcome(s) and statistical analyses of randomised trials

Sally Hopewell[1], Claudia M. Witt[2], Klaus Linde[3], Katja Icke[2], Olubusola Adedire[4], Shona Kirtley[4], Douglas G. Altman[4]
[1]Oxford Clinical Trials Research Unit; [2]Institute of General Practice, Technical University Munich; [3]Institute for Social Medicine, Epidemiology and Health Economics, Charité Universitätsmedizin Berlin; [4]Centre for Statistics in Medicine, University of Oxford
**Correspondence:** Sally Hopewell
*Trials* 2017, **18(Suppl 1):**O12

**Objective**

Selective reporting of outcomes in clinical trials is a serious problem. We aimed to investigate the influence of the peer review process within biomedical journals on the reporting of primary outcome(s) and statistical analyses of reports of randomised trials.

**Methods**

Each month, we searched PubMed (between May 2014 and April 2015) to identify primary reports of randomised trials published in six high impact general and 12 high impact specialty journals. The corresponding author of each trial publication was then contacted by email asking them to complete an online survey investigating changes made to their manuscript as part of the peer review process. Our main outcome was the nature and extent of changes made to manuscripts by authors as part of the peer review process, in relation to reporting of the primary outcome(s) and/or primary statistical analysis. We also assessed how often authors follow these requests and whether this was influenced by specific journal or trial characteristics.

**Results**

Nine hundred eighty-three corresponding authors were invited to take part in the online survey, of which 258 (29%) responded. The majority of trials were multicentre ($n = 191$; 74%), parallel group ($n = 225$; 86.5%); median sample size = 325 (IQR 138 to 1010). Half assessed drug interventions ($n = 127$; 49%), over half were non-industry funded ($n = 159$; 62%) and the primary outcome was clearly defined in 92% ($n = 238$), of which the direction of treatment effect was statistically significant in 48%.

The majority of authors responded (1–10 Likert scale) they were satisfied with the overall handling (mean 8.6, SD 1.5) and quality of peer review (mean 8.5, SD 1.5) of their manuscript by the journal. Only 3% ($n = 8$) said the editor or peer reviewers asked them to change or clarify the trial's primary outcome. However, 27% ($n = 69$) reported they were asked to change or clarify the statistical analysis of the primary outcome; most responded they fulfilled the request, the main motivation being to improve the statistical methods ($n = 38$; 55%) or avoid rejection ($n = 30$; 43.5%). Overall there was no difference between authors being asked to make this change and the type of journal, intervention, significance of the primary outcome or funding source. 36% ($n = 94$) responded that they were asked to include additional analyses that had not been included in the original manuscript; in 77% ($n = 72$) these were not pre-specified in the protocol. 23% ($n = 60$) were asked to modify their overall conclusion, in most cases ($n = 53$; 88%) to provide a more cautious interpretation.

**Conclusion**

Overall there was little evidence of a negative impact of the peer review process in terms of selective reporting of the primary outcome. Most changes requested resulted in improvements to the manuscript, improving clarity of statistical methods used, and providing more cautious conclusions. However, some changes requested by peer reviewers were deemed inappropriate and could have a negative impact on reporting of the final publication, such as the adding of unplanned additional analyses.

### O13

#### Agreeing outcomes that matter to patients? What are the challenges?

Heather Bagley, Bridget Young
University of Liverpool
**Correspondence:** Heather Bagley
*Trials* 2017, **18(Suppl 1):**O13

This abstract is not included here as it has already been published.

### O14

#### Opportunistic trial recruitment during routine primary care consultations for acute conditions: a mixed methods evaluation of recruitment performance and barriers

Jeremy Horwood, Niamh M. Redmond, Christie Cabra, Emer Brangan, Petra Manley, Sophie Turnbull, Jenny Ingram, Patricia Lucas, Alastair D. Hay, Peter S. Blair
University of Bristol
**Correspondence:** Jeremy Horwood
*Trials* 2017, **18(Suppl 1):**O14

**Background**
Evaluating the effectiveness of interventions for acute conditions in primary care often necessitates clinicians opportunistically recruiting patient during time-pressured consultations.
**Aim**
To describe the performance of, barriers to, and implications of clinicians recruiting trial participants during consultations within two primary care feasibility cluster randomised controlled trials, CHICO and IMPACT-PC.
**Methods**
For the CHICO trial GP practices were randomised to a within consultation web-based intervention to reduce antibiotic prescribing for children with acute cough and respiratory tract infection, or usual care. For the IMPACT-PC trial GP practices were randomised to a nurse-led telephone based management service for patients testing for Chlamydia trachomatis (CT) and Neisseria gonorrhoea (NG), or usual care. Performance data analyses were conducted and 44 clinicians and 26 trial participants (patients/parents) were interviewed post recruitment and analysed thematically to explore their experiences.
**Results**
For CHICO, 32 practices were randomised and 501 children were recruited one month ahead of schedule. More children were recruited to the intervention (292, 58%) than the control (209, 42%) arm. There was a difference in clinician type (higher proportion of nurses) and more unwell children in the intervention arm. Although just over a quarter of clinicians were nurses, they recruited more frequently, recruiting 220 (44%) of the children. Interviews revealed that many clinicians prioritised dealing with the cough first and only afterwards attempted to recruit children. This meant that clinicians, particularly in the control arm, reported they preferentially recruited less unwell children, because these were quicker and it was easier to ‘fit in’ the research on top of the normal consultation.
For IMPACT-PC, 11 practices were randomised, 1154 patients were recruited (60% of eligible patients) and 30 (2.6%) patients tested positive for CT, 9 (0.8%) tested positive for NG and 3 (0.3%) tested positive for both. CT positivity was higher (4.3%) amongst individuals’ eligible but not recruited to the study in intervention practices. Interviews revealed the main reason for failure to recruit eligible patients was insufficient time to undertake consent procedures. Despite patient consent being recorded, patients were sometimes unclear that they were participating in a research study. However, patients found both the intervention and the use of their medical records in evaluation acceptable, as long as their anonymity was maintained.
**Conclusions**
Recruitment to both trials was successful in terms of numbers recruited and timescales and the interventions were acceptable and feasible to clinicians and patients/parents. However, the requirement for individual patient/parent consent during the consultation was a barrier to recruitment and may have introduced bias. Given the nature of the interventions and the views expressed it is viable and valid that future trials of both interventions should not require individual consent providing the choice to opt out is provided and follow up procedures maintain patient anonymity. Trials evaluating the effectiveness of interventions for acute conditions in primary care should avoid recruitment processes that add burden to routine practice. The study highlights the value of conducting mixed method evaluations of recruitment performance and barriers during feasibility trials to inform future trial design.

### O15

#### Designing clinical trials with age-related multiple morbidity outcomes

Mark Espeland[1], Jill P. Crandall[2], Stephen Kritchevsky[1], Eileen M. Crimmins[3], Brandon R. Grossardt[4], Judy Bahnson[1], Michael E. Miller[1], Jamie Nicole Justice[1], Nir Barzilai[2]
[1]Wake Forest School of Medicine; [2]Albert Einstein College of Medicine; [3]University of Southern California; [4]Mayo Clinic
**Correspondence:** Mark Espeland
*Trials* 2017, **18(Suppl 1):**O15

**Background and Objectives**
The incidence of age-related chronic diseases rises exponentially with age. This parallels the exponential increases with age in rates of major disease-specific deaths tracked by the US National Center for Health Statistics, including those for heart disease, cancer, stroke, type 2 diabetes mellitus, and Alzheimer’s disease. It has repeatedly been shown that the major, and by far the most potent, risk factor cutting across age-related chronic diseases is age itself. There is growing evidence for a biologic construct underlying aging, leading to the potential that interventions may be developed to slow its progression. The primary goal is not to increase the number of years lived, but to increase the number of years lived with better health and function. The NIA Interventions Testing Program has been established to organize research towards this goal across model organisms. As interventions emerge from this program as candidates for human intervention, clinical trials will be mounted to assess their efficacy. We discuss design and analytical issues, including the choice of outcomes, eligibility criteria, monitoring rules, and analytical strategies. We present projections of rates at which outcomes occur, as benchmarks for estimating the statistical power for future trials.
**Methods**
Parallel analyses were conducted using data from three large cohorts of older individuals: the Rochester Epidemiology Project, the Health and Retirement Study, and the Women’s Health Initiative Observational Study. These allowed us to contrast outcomes, evaluate potential eligibility criteria, and project incidence rates.
**Results**
The incidence rate of composite multi-morbidity outcomes and the rate that they accumulate over time are attractive clinical trial outcomes. Rates increase with age and, for cohorts at suitably increased risk due to choice of eligibility criteria, are sufficiently great enough to support the development of tractable (4–6 years; $N =$ 3,000) multi-center clinical trials. To provide evidence that interventions target aging and health span, rather than individual components of the composite outcome measure, nuanced approaches to monitoring and analysis are required, which we describe. The benchmarks and methods that we present support the feasibility of designing efficient clinical trials for interventions targeting aging. As an example, we describe the design of the Targeting Aging with Metformin (TAME) multicenter clinical trial.
**Conclusions**
Clinical trials targeting aging are feasible, but require careful design consideration and monitoring rules.

### O16

#### Improving the testing of treatment effect in clinical trials with time to event outcomes

Song Yang[1], Ross Prentice
[1]National Heart, Lung, and Blood Institute, NIH; [2]Fred Hutchinson Cancer Research Center
**Correspondence:** Song Yang
*Trials* 2017, **18(Suppl 1):**O16

This abstract is not included here as it has already been published.

### O17

#### Value-added use of clinical study data: a biolincc perspective on creating well-annotated data packages for the wider scientific community

Leslie Carroll[1], John Adams[1], Corey Del Vecchio[1], Karen Mittu[1], Kevin Zhou[1], Jane Wang[1], Carol Giffen[1], Elizabeth Wagner[2], Sean Coady[3]
[1]Information Management Services, Inc.; [2]Translational Blood Science and Resources Branch, Division of Blood Diseases and Resources, National Heart, Lung, and Blood Institute; [3]Epidemiology Branch, Prevention and Population Sciences Program, Division of Cardiovascular Sciences, National Heart, Lung, and Blood Institute
**Correspondence:** Leslie Carroll
*Trials* 2017, **18(Suppl 1):**O17

**Introduction**
The National Heart, Lung, and Blood Institute (NHLBI) established the Biologic Specimen and Data Repositories Information Coordinating Center (BioLINCC) www.biolincc.nhlbi.nih.gov in 2008 to provide online access to NHLBI data and biospecimen resources. To assist non-study investigators' use of the datasets, each study's BioLINCC webpage provides information on the study design and results, including documents that provide insight into the study data. Given the recent interest by journal editors in the rapid release of publication data, the need for efficient curation methods is becoming more important. The procedures that have been developed by BioLINCC to review and prepare study datasets and documents for sharing with secondary users are one example of how this can be accomplished.
**Methods**
Data packages submitted to BioLINCC undergo review for secondary usability. Data dictionaries are examined for ease of use by researchers outside of the original study group. Reviews are performed to find any data elements that are considered personally identifiable information (PII) which are then redacted or recoded in order to de-identify the data for distribution. An informed consent questionnaire is completed to discern if there are any restrictions related to wide data sharing. A comparison of the data with a publication representative of the study as a whole, such as a primary outcome manuscript, is conducted. The population included in the analysis as well as key statistics are reproduced and deviations identified. Key variables used in the analysis (e.g. inclusion criteria, adjudicated variables, outcomes) are noted and the documentation is examined to ensure these variables are well annotated. If study biospecimens are being transferred to the NHLBI Biorepository, the link between clinical data and those specimens is verified. Additional documentation including the study protocol, informed consent templates, MOP/MOOs, annotated forms, codebooks, and a publications list are collected to provide a useful context for the data and biospecimens.
**Results**
Over the first seven years of BioLINCC, data from 139 completed studies were made available through BioLINCC and 666 requests for 1496 data packages were fulfilled. A total of 130 original data packages and updates were processed and shared with an average effort of 75 hours per data package. The level of effort varied, not according to the complexity of the study design, but due to the stage of curation of the submitted data and documentation. Additional effort at both BioLINCC and the parent study's coordinating center was required in nearly all reviews to prepare and obtain missing information such as algorithms for calculated analysis variables, explanatory data labels, code books, key variables used in analyses, annotated forms, and biospecimen linking files. To date, over 600 publications are known to have resulted from requestors using BioLINCC resources.
**Conclusion**
Efficient preparation of study data and documents is essential to maximizing the scientific utility of study resources. Preparing data for release to the general scientific community requires a significant commitment of time and effort to ensure investigators, not affiliated with the original study, have sufficient information to effectively conduct secondary analyses.

### O18

#### Patient preferences for outcomes in clinical trials: implications for medicines optimization

Emily Holmes[1], Anthony G. Marson[2], Dyfrig A. Hughes[1]
[1]Bangor University; [2]University of Liverpool
**Correspondence:** Emily Holmes
*Trials* 2017, **18(Suppl 1):**O18

**Background**
Drug choices for given therapeutic indications are often guided by clinical trial evidence, however, patients may consider outcomes beyond those measured as primary endpoints within trials in their decision to adhere to medication. Discrete choice experiments (DCEs) are a valid method that has been used to quantify patient preferences for drug outcomes. Data from DCEs may be combined with the results of clinical trials to provide a more patient-orientated perspective on drug choice.
**Objective**
To demonstrate the impact of incorporating patients' benefit-risk preferences into the results of clinical trials, using a case study of preferences for anti-epileptic drugs (AEDs).
**Methods**
Preference weights for outcomes of AEDs (12-month remission, fewer seizures, depression, memory problems, aggression, foetal abnormality) were derived from a web-based DCEs of 414 adult patients with epilepsy. Rates for each of these outcomes were extracted from a large randomised controlled trial comparing the effectiveness of new and standard AEDs (SANAD), and from a systematic review of treatments of epilepsy in pregnancy. The preference weights were combined with the clinical event rates to estimate of patient utility for each AED. The probability of patients preferring each AED was then calculated as the ratio of exponentiation of the utility of each individual AED to the sum of the exponentiation of the utilities of all AEDs. Results were compared to rankings of AEDs as indicated by clinical trials.
**Results**
The rank order of AEDs based on trial data for remission: lamotrigine, carbamazepine, topiramate, oxcarbazepine, then gabapentin, changed when patient benefit-risk preference was considered. The probability of patients with partial epilepsy preferring each AEDs was, in descending order: carbamazepine (0.29), lamotrigine (0.26), oxcarbazepine (0.24), gabapentin (0.15), topiramate (0.07). Women with the potential to become pregnant, had a preference probability of: lamotrigine (0.31), oxcarbazepine (0.21), gabapentin (0.20), carbamazepine (0.19), topiramate (0.09). Comparable results were found for patients with generalised or unclassified epilepsy. Changes to rank ordering are explained by patients' stronger preferences for reducing the risk of AEs than for improving treatment benefit. In return for a 1% improvement in 12-month remission, the maximum acceptable risk of adverse events was: depression 0.31%, memory problems 0.30%, aggression 0.25%. The maximum acceptable risk of adverse event in exchange for a 1% improvement in 12-remission was, for women with the potential to become pregnant was: depression 0.56%, memory problems 0.34%, and foetal abnormality 0.20%.
**Conclusions**
DCEs represent a robust method for quantifying benefit-risk preferences that can be analysed alongside clinical trial data, to provide a patient-orientated perspective on the optimal choice of treatment.

### O19

#### Patient and public involvement into the design of a paediatric surgical trial: the ninja study

Cushla Cooper[1], David Beard[1], Abhilash Jain[1], Aina Greig[2], Adam Sierakowski[3], Matthew Gardiner[1], Nicola Farrar[1], Jonathan Cook[1]
[1]University of Oxford; [2]Guys & St Thomas' Hospital; [3]Mid-Essex Hospital Services NHS Trust
**Correspondence:** Cushla Cooper
*Trials* 2017, **18(Suppl 1):**O19

##### Background

Patient and Public Involvement (PPI) is increasingly important in the design of research and surgical trials. Evidence on PPI for paediatric trials appears is limited. Using a case study, the NINJA study, the potential impact of PPI on the design of a new paediatric surgical trial is highlighted.

NINJA study: Nail bed injury is the commonest hand-related cause of emergency paediatric consultation. After nailbed repair, there is debate whether the nail should be replaced or not. There is controversy and uncertainty around whether replacing the nail is beneficial in causing or preventing infection. A 60 patient pilot study (NINJA-P) was conducted to demonstrate the viability of a large multicentre paediatric surgical trial comparing infection rates in patients with replaced and discarded fingernails. Patients were recruited in just over 4 months at 4 sites and followed-up for 4 months. The paediatric population created some unique aspects and challenges, especially with retention and completion of patient-reported assessments.

##### Methods

The issues raised by the pilot were put to a youth group - the Young Person's Executive (YiPpEe) group based at Oxford University Hospitals NHS Foundation Trust and also to a focus group of parents (and one toddler). Information from both groups was collated to inform the development of the definitive study. The issues discussed were: Choice of the primary outcome measure and how to administer this; Retention of this study population; Presentation of study information.

##### Results

Regarding the outcome, the appearance of the nail was overwhelmingly the most important variable. This was in contrast to the clinicians' choice of outcome; the incidence of infection. The NINJA study now has co-primary outcome measures of appearance and infection rates. The groups shared ideas for how children (or parents) could measure their satisfaction with their nail appearance. A simple 3 point scale showing facial expressions was developed. This was favoured over the 5 point scale used in the pilot. Both groups were clear about the method of collection for follow up data. This population includes busy working parents. They suggested moving away from postal questionnaires and clinical visits, if not necessary, and employing mobile technology i.e.: 'apps' to upload photographs and complete questionnaires. The parent group felt the option to complete follow up requirements in this manner would improve the retention rate. Both groups had specific ideas regarding patient information presentation. The use of technology, videos, and comic-strips showing real people was supported. Collaboration with YiPpEe will continue to help develop information portals for the study.

##### Conclusions

Due to PPI involvement, the full NINJA study objectives were modified and a follow-up regime and content designed to suit this very specific patient population was developed. Our experience shows that solutions offered by children and parents can be incorporated into trial design at an early stage. The PPI exercise helped address and titrate issues raised in a pilot study and generated design and procedural elements that had not previously been discussed.

### O20

#### Consent pathway options in trials of emergency interventions

Julia Sanders[1], Peter Collins[2], Julia Townson[3], Nadine Aawar[3]
[1]Cardiff University; [2]Cardiff University, School of Medicine;
[3]Cardiff University, Centre for Trials Research
**Correspondence:** Julia Sanders
*Trials* 2017, **18(Suppl 1):**O20

##### Background

In trials of emergency care interventions standardised pathways for obtaining participant consent can be inappropriate and alternative models are required. Unless specific approval is granted, obtaining participant consent is an essential prerequisite to research participation. OBS2 was a randomised trial evaluating the effectiveness of using early fibrinogen replacement in the management of complex postpartum haemorrhage. Reflecting the range of potential clinical scenarios in which recruitment could potentially occur, and to meet NHS ethics committee requirements, several consent pathways were identified, each requiring tailored patient information and consent forms.

##### Methods

All women booked to give birth at the six participating maternity units during the recruitment period, were provided with written information about the study during the antenatal period. Five consent pathways were developed: (1) for women at higher risk of postpartum haemorrhage, pre-event consent; (2) for women with controlled haemorrhage, written consent at the time of the bleed; (3) for women competent to provide assent during the bleed, but unable to provide written consent, verbal assent at the time of the bleed; and in the event of women lacking capacity to provide assent, pathways utilising a personal (4) or professional representatives (5). All women, regardless of the pathway followed, once well enough following their bleed were also required to provide written consent for use of their collected data.

##### Results

The study recruited 663 women who experienced a moderate to severe postpartum haemorrhage, with a minimum of 1,000 ml blood loss. Data relating to the mode of consent were captured on the site screening logs for 511 participants. No participants were recruited using the pathways developed for written consent provided at the time of the bleed, nor for the pathway utilising a professional legal representative. Antenatal (pre-bleed) consent was obtained from 15 (2.9%) recruited women; verbal assent was provided by 473 women (92.5%) during the haemorrhage, and for 23 women (4.5%) assent was provided by a personal representative, a relative or friend present. All women, once well enough following their bleed, provided written consent to the use of collected data.

##### Discussion

Appropriate recruitment and consent pathways are an essential component in the design of all trials. Trials of emergency intrapartum care bring particular challenges as they combine a known population, women booked to give birth at participating units, with unknown eligible potential participants, in the case of OBS2, women who went on to have a postpartum haemorrhage of >1,000 ml. The requirement to provide all potentially eligible women with antenatal information was intensive of professional time and resources. The pathways of consent available to staff in the recruitment of women were identified to have strengths and weaknesses, and these were reflected in the utilisation of each. Based on the experience of the OBS2 trial, the legal and logistic complexities of consent in emergency trial settings will be presented and discussed.

### O21

#### How can incentives be designed and used to improve recruitment and retention in ways that are effective, efficient and ethical?

Peter Bower[1], Beth Parkinson[2], Eleonora Fichera[2], Rachel Meacock[2], Matt Sutton[2], Shaun Treweek[3], Nicola Harman[4], Katie Gillies[3], Nicola Mills[5], Gillian Shorter[2]
[1]MRC North West Hub for Trials Methodology Research; [2]University of Manchester; [3]University of Aberdeen; [4]University of Liverpool; [5]University of Bristol; [6]Ulster University
**Correspondence:** Peter Bower
*Trials* 2017, **18(Suppl 1):**O21

##### Background

Recruitment and retention is critical for trials, yet both remain significant problems. Very little evidence exists on effective methods to boost recruitment and retention. There is increasing interest in exploring financial and non-financial incentives.

We asked the question: "How can incentives best be designed and used to improve recruitment and retention in ways that are effective, efficient and ethical?"

**Methods**

We conducted a structured scoping review to explore the current literature on the use of incentives in health care (inside and outside of trials).

The review was underpinned by a conceptual framework drawn from microeconomics, agency theory and behavioural economics to help us determine which elements of incentive design to consider. We also explored potential intended and unintended effects of incentives.

We also ran interactive sessions with experts in the field (trials and behavioural economics), principal investigators, regulatory representatives, and patients, to better understand stakeholder views.

Synthesising these two forms of data, we developed guidance for the design and delivery of incentives in trial recruitment and retention.

**Results**

We searched PubMed and Econlit, securing 963 eligible studies, of which 123 were included. Some of the core recommendations from the review are as follows:

1. When designing an incentive system it is vital to consider the current incentives already operating
2. The evidence is mixed about who incentives should be directed towards (patients, recruiters, clinicians or a combination)
3. Incentivising processes (such as invitations to a trial) is likely to induce more effort than incentivising outcomes (e.g. recruitment and retention). However, there is a danger that changes in process do not translate to increased recruitment and retention, or lower the overall suitability of recruits
4. Complex payment schemes can better direct incentives to increased activity, limiting costs. However, they will take more time and effort to implement, and may fail to induce increased effort
5. Monetary incentives are likely to have a larger direct price effect, but may have negative psychological effects (e.g. crowding out altruism)
6. Other unintended consequences of incentives may include effects on the types of patients recruited and research integrity

The impact of incentives will be influenced by many features, such as the setting of the trial, the risk inherent in trial procedures, and the social and demographic characteristics of patients. Patients discussed the importance of the language used in offering incentives, and the impact on the professional-patient relationship. Patients reported less concerns over the use of incentives for retention compared to recruitment.

We will present our findings in full, and explore incentives schemes which illustrate different features, advantages and disadvantages.

**Conclusions**

There is a need to consider the role of incentives in enhancing recruitment and retention, taking account of the ethical issues and thinking creatively about design to maximise benefit and minimise harm. Equally, there is a need to test their effectiveness and efficiency using appropriate randomised and non-randomised methods to ensure that any systems are a good use of public funds.

### O22

#### Scaling the drug supply management mountain: a case study of the add-aspirin trial

Kenneth Babigumira[1,2], Nancy Tappenden[1,2], Fay Cafferty[1,2], Marta Campos[1,2], Carlos Diaz-Montana[1,2], Keith Fairbrother[1,2], Samuel Rowley[1,2], Mary Rauchenberger[1,2], Ruth E. Langley[1,2]

[1]MRC Clinical Trials Unit at UCL, Institute of Clinical Trials and Methodology, UCL, London, UK; [2]MRC London Hub for Trials Methodology Research, London, UK

**Correspondence:** Kenneth Babigumira

*Trials* 2017, **18(Suppl 1):**O22

**Background**

Managing drug supply for large clinical trials presents significant logistical challenges. The MRC CTU at UCL has used either an external drug supply management system (DSMS) or in-house tools (spreadsheets or MS Access databases). However, there are challenges integrating these systems with internal study databases to work seamlessly.

Add-Aspirin is a phase III double-blind, randomised trial with over 180 sites in the UK, aiming to recruit 10,000 patients. The size of the trial and the use of double-blinded drug as part of the trial design led to a decision to develop an in-house DSMS.

**Methods**

We will describe the key considerations that influenced the design of the DSMS, whilst focussing on scalability, as the DSMS was intended to be a cost-effective solution that could be used by a number of trials within the unit.

The key activity was building a forecasting-and-site-refill algorithm to minimise drug wastage whilst optimising shipment quantities, therefore reducing shipping costs. To achieve the required scalability, the first consideration was introduction of flexibility into the algorithm. It was developed with many inputs/parameters to cater for variation across future trials and sites in terms of number of patients, recruitment rates, site capacity and location. Each shipment request is reviewed and approved by the trial team, which further allows the process to be fine-tuned for each study.

Integration with internal and external contract research organization (CRO) systems was fundamental to building a successful system. The DSMS is a web-based system (C#/MVC.NET/SQL Server database) and has been integrated with an existing randomisation system and study database. The system allows for pack/kit numbers to be used in blinded trials, therefore integration with CRO systems is vital to an efficient pack selection process for site shipments.

Another important consideration to facilitate scalability was ensuring ease of setting up new trials and studies, which has been enabled by how the DSMS integrates with internal systems which house both trial and site data. In addition to this, a metadata template was created to expedite the process of gathering new requirements from new trials. This has been successfully implemented for Add-Aspirin and its use has already been extended to the FOCUS4 biomarker-stratified platform trial.

Finally another important component was the ease of use, supported by the provision and implementation of system training. Training has been developed using different formats, webinars, user guide, slides and videos. The training materials are available from within the system and on the trial website; they can easily be adapted by new trials.

**Conclusion**

In the Add-Aspirin trial to date, over 1,500 participants from 128 UK sites have been dispensed 3,000 treatment packs using the DSMS. The number of shipments created so far is in line with what was projected using simulation before the start of the trial. The shipments have been optimised for low, medium and high recruiting sites. The DSMS is a platform that continues to evolve as new functionality is required for the Add-Aspirin trial and other trials within the MRC CTU at UCL portfolio.

### O23

#### Decision making in the face of biomarker uncertainty

Chris Harbron

Roche

*Trials* 2017, **18(Suppl 1):**O23

**Background**

Increasingly drugs are being developed with consideration to a biomarker defining a sub-population where the drug will demonstrate increased clinical benefit. However, frequently the prior evidence of the necessity of the marker isn't overwhelming or the exact definition of the biomarker cannot be specified in advance either in terms of a cut-off and/or the optimal assay or property of the biomarker that will be used to define the sub-population. In these cases a study will typically be run in an unselected population, and the analysis performed in both the whole study population as well as biomarker defined sub-populations. In these situations, although many separate biomarker hypotheses may be tested, it is desired to maintain an overall type-1 error rate control for testing the single hypothesis that the drug has an effect within a patient population.

#### Methods

Spiessens-Dubois (2010) provide an approach for controlling overall type-1 error rate when all biomarker hypotheses are nested by considering the correlation between different tests using an analogous approach to group sequential analysis. This describes the situation of a single biomarker being investigated at multiple cut-offs. In this presentation, this is extended to the more general case of non-nested tests representing the situation of multiple biomarkers which may be correlated but not ordered, still using the intrinsic correlation from overlapping populations to construct an efficient test. This solution generates sets of significance boundaries all maintaining an overall type-1 error rate which can be optimized according to a variety of different optimality criteria based upon different characteristics of the study including functions of power, effect size and significance levels.

#### Results

We present and compare the results of optimising the significance boundaries of a study using different optimality criteria and link this to the properties of the biomarkers being investigated. We give guidance as to how this approach may be implemented in practice and the beneficial discussions within clinical teams that adopting these approaches will facilitate.

### O24

### Using phone, SMS and email screening reminders to improve clinical trial recruitment: results from a sub-study of the t4dm diabetes prevention study

Karen Bracken[1], Wendy Hague[1], Gary Wittert[2], Anthony Keech[1], Kristy Robledo[1]
[1]NHMRC Clinical Trials Centre, University of Sydney; [2]University of Adelaide
**Correspondence:** Karen Bracken
*Trials* 2017, **18(Suppl 1):**O24

#### Background

Successful and timely participant recruitment is a key aspect of clinical trial conduct. Failure of potential participants to complete screening is often reported as an issue in prevention studies. The T4DM diabetes prevention study, being conducted at 6 sites around Australia, employs a step-wise screening process. Potential participants first complete an online study registration questionnaire and, if eligible, the online system generates consent and laboratory forms. Participants are then required to attend for lab screening before being allocated to the nearest study site. After the first 12 months, only 58% of men who registered online had attended the lab for screening. Given the cost and difficulty associated with attracting men to register, a quarterly email reminder and ad-hoc phone reminders were introduced to improve uptake of lab screening. After a further 24 months, we observed that the number of men attending for screening late (more than 12 weeks after registration) had grown but the overall lab screening rate remained largely unchanged at 60%.

#### Aim

In this sub-study we aimed to assess the impact of phone and SMS (text message) reminders on lab screening rates while also maximizing lab screening uptake in the lead up to recruitment close in December 2016.

#### Method

Between June and October 2016, 709 participants who did not attend lab screening within 4 weeks of online registration were randomized to receive either an SMS or a phone reminder to attend for lab screening. This was in addition to an automated email reminder that all registered participants receive at 4 weeks. Participants were followed to determine whether they attended lab screening by 8 and 12 week time points. The sub-study completed enrollment in October 2016 with all data collection to be complete by the end of 2016.

#### Preliminary results

Prior to the introduction of the reminder sub-study, only 12% of men who didn't attend lab screening within 4 weeks had done so by 8 weeks. To date, the introduction of reminders has increased this to 18% (a 6% increase) based on the 358 participants who had reached the 8 week time point by October 2016. Completion of the sub-study later in 2016 will reveal how effective phone reminders were compared to SMS reminders. The cost of an SMS reminder is approximately $0.18AUD with negligible staff time required per person reminded. The cost of a phone reminder is approximately $0.48AUD with an average of 4 minutes of staff time required per person reminded.

#### Conclusion

This sub-study will establish the extent to which phone and SMS screening reminder strategies increase participant follow-through with the screening process. It will also assess the relative costs of each approach in terms of cost per notification, cost per participant screened and cost per participant enrolled. These findings have the potential to inform the choice of screening reminder strategy in future prevention clinical trials.

### O25

### Adaptive enrichment design for randomised clinical trials with predictive biomarkers

Deepak Parashar, Iliana Peneva, Nigel Stallard
University of Warwick
**Correspondence:** Deepak Parashar
*Trials* 2017, **18(Suppl 1):**O25

There has been a surge in designing clinical trials based on the assumption that a biomarker is predictive of treatment response. Patients are stratified by their biomarker signature, and one tests the null hypothesis of no treatment effect in either the full population or the targeted subgroup. However, in order to directly verify the predictability of a biomarker, it is essential that hypothesis be tested in the non-targeted subgroup too and within a randomised controlled trial [1]. In a Phase IIB oncology trial with progression free survival (PFS) endpoint, the data obtained can inform the Phase III design aimed at establishing overall survival whether to restrict recruitment to just the targeted subgroup or not.
We propose a new two-stage adaptive randomised Phase II population enrichment trial design, with PFS as the primary endpoint and comparing an experimental drug with a control treatment. We adaptively test the null hypotheses of hazard ratios in both the targeted as well as the non-targeted subgroups, with strong control of the familywise error rate. It is assumed that the hazard ratio of the targeted subgroup is much less than that of non-targeted, since the drug is expected to be more beneficial for the biomarker-positive subpopulation.
Simulations for an example trial in non-small cell lung cancer show that the probability of recommending an enriched Phase III trial increases significantly with the hazard ratio in the non-targeted subgroup. We compare our decision rules with [1] and illustrate the efficiency achieved. Our adaptive design testing first in the non-targeted subgroup followed by testing in the targeted subgroup for a randomised controlled trial constitutes part of the proof of a biomarker's predictability.

### O26

### The quality of reporting of pilot and feasibility cluster randomised trials: a systematic review

Claire Chan[1], Leyrat Clémence[2], Eldridge M. Sandra[1]
[1]Queen Mary University of London; [2]London School of Hygiene and Tropical Medicine
**Correspondence:** Claire Chan
*Trials* 2017, **18(Suppl 1):**O26

**Background**
There are an increasing number of studies described as pilot and feasibility studies. A pilot or feasibility trial conducted in advance of a future definitive trial is a study where part or all of a future trial is carried out on a smaller scale to see whether it can be done and whether we should proceed with it. Reporting of pilot and feasibility studies is poor, and these studies are particularly important when designing cluster randomised trials (CRTs), which bring with them extra complications.
**Objectives**
To systematically review the quality of reporting of pilot and feasibility CRTs. In particular, to identify 1) The number of pilot CRTs conducted between 01/01/2011 and 31/12/2014, 2) Whether pilot CRTs have appropriate objectives and methods, and 3) The extent to which the quality of reporting of pilot CRTs is sufficient.
**Methods**
We searched PubMed (2011–2014) for CRTs with “pilot” or “feasibility” in the title/abstract, that were assessing some element of feasibility and showing evidence the study was in preparation for a main effectiveness trial. Quality assessment criteria were based on the CONSORT extension for CRTs, and the CONSORT extension for pilot trials which was in the final stages of development.
**Results**
Eighteen pilot CRTs were identified, with most (56%) published in the UK. 44% did not have feasibility as their primary objective, and many performed formal hypothesis testing for effectiveness/efficacy despite being underpowered (50%). Most pilot CRTs (83%) reported the term “pilot” or “feasibility” in the title, and discussed implications for progression from the pilot to the future definitive trial (89%), but less than half gave reasons for the randomised pilot trial (39%), reported a rationale for the sample size (44%), reported criteria used to judge whether or how to proceed with the future definitive trial (17%), or reported where the pilot trial protocol could be accessed (39%). Most pilot CRTs defined the cluster (100%), and reported the number of clusters randomised (94%) and assessed for the primary objective (82%). Items reported least well included how clusters were consented (11%), the cluster design during the description of the rationale for numbers in the pilot (17%), who enrolled clusters (17%), the number of exclusions for clusters after randomisation (18%), a table showing baseline characteristics for the cluster level (11%), and from whom consent was sought (11%).
**Conclusions**
The identification of just eighteen pilot CRTs highlights the need for increased awareness of the importance of carrying out and publishing pilot CRTs and good reporting. It is possible that some pilot CRTs were missed because they did not include “pilot” or “feasibility” in the title/abstract. Pilot CRTs should primarily be assessing feasibility, with methodology reflecting this focus. Improvement is needed in reporting reasons for the pilot, rationale for the sample size, progression criteria, and where the protocol can be accessed. Cluster level items also need better reporting, since these are important for assessing feasibility. We recommend adherence to the new CONSORT extension for pilot trials, in conjunction with continued adherence to the CONSORT extension for CRTs.

### O27

#### Best practices for study drug management and accountability throughout the study lifecycle in multi-site randomized controlled trials

Dikla Blumberg[1], Patricia Novo[2], Beth Jeffries[1], Lauren Yesko[1], Abigail G. Matthews[1], Julia Collins[1], Dagmar Salazar[1], Eve Jelstrom[1], Matthew Wright[1], Radhika Kondapaka[1]
[1]The Emmes Corporation; [2]NYU School of Medicine
**Correspondence:** Dikla Blumberg
*Trials* 2017, **18(Suppl 1):**O27

Managing study drug throughout a trial is a complex, vital task further compounded when there are multiple research sites participating. Adherence to good clinical practice (GCP) requirements and all applicable regulatory requirements is paramount. The National Drug Abuse Treatment Clinical Trials Network (CTN) Clinical Coordinating Center (CCC) and Data and Statistics Center (DSC), both at the Emmes Corporation, collaboratively developed a series of processes and tools, some of which are incorporated in the electronic data system, to ensure an efficient and controlled chain of custody and process beginning from initial supply distribution through dispensing procedures at the research sites and final reconciliation and destruction. The CCC and DSC consider several factors when determining the process for study drug management, including treatment blinding, drug type, quantity and packaging, frequency of distribution, expiration dating, and the number of sites. Based on these parameters, the CCC assists the study teams in development of clear and thorough drug management logs as well as defining drug storage and temperature monitoring requirements. To remedy last minute requests, supply hoarding, and waste at the sites, the coordinating centers have developed a centralized inventory tracking and reordering process to monitor drug supply and distribution. In this process, research staff report inventory weekly directly in the Electronic Data Capture (EDC) system, and the data is pulled into reports, which identify reorder needs based on thresholds and usage. Before shipping initial supplies to each site, all regulatory documents are collected and training is provided to research sites on the importance of drug accountability and consequences for participant safety if inaccurately reporting drug dosing and disposal. Site monitors review the drug logs, medication storage, and regulatory documentation throughout the trial (remotely or on-site) in order to identify and resolve any improper practices, discrepancies and errors. The Emmes Corporation has supported substance use treatment interventions implemented in the CTN for over 11 years, and throughout that time have developed best practices including using systematic, clear and precise processes for study drug procurement, distribution, and monitoring. Over 14 clinical trials across 105 clinical sites have involved study drug, including 4 double-blinded studies and 4 Investigational New Drugs and 6 studies using controlled substances. Effective communication between the CCC/DSC, central pharmacy, third-party vendors, research sites, and all other stakeholders allows for efficient planning and prompt resolution to problems that arise. Supporting this communication with real time data collection and reporting allows for the proper maintenance of a comprehensive and accurate study drug management system. This presentation will emphasize best practices for achieving an organized and controlled chain of custody throughout the life of a trial.

### O28

#### A look at the future of data standardization and sharing in clinical research

Derk Arts[1], River Wong[2], Nidal Amenchar[2]
[1]Department of medical informatics, Academic Medical Centre (AMC), Amsterdam; [2]Castor Electronic Data Capture
**Correspondence:** Derk Arts
*Trials* 2017, **18(Suppl 1):**O28

Sharing collected data from trials has the potential to exponentially increase the efficiency and accuracy of research and reduce research waste through repeated trials. Unfortunately, barriers to do so still exist. These include the difficulty to find, access and use previously collected research data sets because they are not centrally indexed or standardized.
The European Commission unveiled its plans in April earlier this year to create a new European Open Science Cloud that will offer Europe's 1.7 million researchers and 70 million science and technology professionals a virtual environment to store, share and reuse their data across disciplines and borders. The aim is to make all data derived from EU-funded research projects Findable, Accessible, Interoperable and Reusable (FAIR). The European Union has fully embraced the FAIR principles, which are created to ensure high data quality, shareability, and usability.
We will discuss the benefits of FAIR data, explain what is required for FAIR data, and give guiding principles on how to create FAIR data.

We will also go further into the challenges that we face as we move towards the worldwide implementation of FAIR.
These challenges include: Ensuring all research data is of high quality Standardization of research data at the source Provide everyone with the ability to make FAIR data (FAIRification)
We will discuss how to deal with these challenges and present our solution to make capturing FAIR data accessible for every researcher worldwide. By making these data available in environments like the European Open Science Cloud, the world will experience a major increase in the quality and efficiency of research. This in turn will help to improve healthcare in the long run, by ensuring better quality of evidence to base our medical guidelines on.

### O29

#### Participant involvement as a form of patient and public involvement in clinical trials: experience, reflections and recommendations

Claire Vale, William Cragg, Ben Cromarty, Bec Hanley, Annabelle South, Richard Stephens, Kate Sturgeon, Mitzy Gafos
MRC Clinical Trials Unit at UCL, Institute of Clinical Trials and Methodology, UCL, and MRC London Hub for Trials Methodology Research, London, UK
**Correspondence:** Claire Vale
*Trials* 2017, **18(Suppl 1):**O29

**Background**
Patient and public involvement (PPI) in clinical trials describes a variety of activities ensuring that research is carried out collaboratively with patients and/or members of the public. Traditionally, the patients and public involved have not been taking part in the study in question and in the UK, guidance from INVOLVE suggests that it is not appropriate to involve clinical trial participants in PPI activities. However, as part of a study exploring PPI in randomised controlled trials conducted by the MRC CTU at UCL, we identified 3 studies (2 trials and 1 cohort study) where participants had been involved. In the light of this we reviewed the concept of participant involvement, setting out to develop guidance based on our experience.
**Methods**
Two workshops were held at the MRC CTU at UCL to discuss: definitions; rationale; potential advantages and disadvantages; models; and appropriateness of participant involvement in clinical trials. We considered how participant involvement might overlap with, or differ to, involvement of other patients and the public. Workshops were attended by two patient representatives and seven staff members, each of whom has experience of PPI. Staff members from studies that had actively involved participants shared details of that work to inform discussions.
**Results**
Trial participants were defined as individuals taking part in the study in question, irrespective of whether or not they have completed their trial treatment and follow-up. Their direct experience of taking part in the trial may be especially useful in studies of new interventions or procedures, where they may be the only people who have experience of the interventions, or where it is hard to identify patient or community groups that include or speak for the study population, for example in prevention trials. Participant involvement is possible at all stages of a trial, except identifying the research question and trial design (when, there are no participants to involve). Participants can be involved in trials through a range of models, with managerial, oversight or responsive roles (as for PPI). The only specific role identified as being inappropriate for trial participants was involvement in data safety and monitoring committees, because of the likelihood of obtaining information about the arm of the trial they are in and the potential for unblinding. Involvement of participants can benefit trials by improving the trial experience for participants; optimising study procedures; and improving the communication of key messages and results. Specific challenges to involving participants included managing confidentiality; practicalities around payments; and ethical concerns around recruitment for involvement.
**Conclusions**
Our experiences of participant involvement have demonstrated that trial participants can add insight to the studies they are involved in. Participant involvement in clinical trials is feasible and seems to offer significant benefits in some circumstances. We recommend that current INVOLVE guidance on PPI should be updated to include participant involvement as a valid and potentially useful approach to PPI. Participant involvement can complement other forms of PPI in clinical trials in appropriate circumstances. We are developing plans and strategies to further explore its potential.

### O30

#### Administering patient-reported outcome questionnaires in Australian cancer trials: the roles, experiences, training received and needs of site coordinators

Rebecca Mercieca-Bebber[1], Derek Kyte[2], Melanie Calvert[2], Martin Stockler[1], Madeleine King[1]
[1]The University of Sydney; [2]University of Birmingham
**Correspondence:** Rebecca Mercieca-Bebber
*Trials* 2017, **18(Suppl 1):**O30

**Background**
In clinical trials, patient-reported outcome (PRO) questionnaires offer information about the impact of disease and treatment from the patients' perspective. The 'Clinical Research Coordinator (CRC)' is typically responsible for PRO data collection. Recent evidence suggests CRCs are not offered adequate PRO-specific trial guidance. As PROs are increasingly being valued in the interpretation of cancer trials, the need to scrutinise current practice has become ever more important. The present study explored the experiences of Australian CRCs responsible for PRO assessment in cancer trials.
**Methods**
Cancer trial CRCs at approved Australian sites with 12+ months PRO experience were eligible. Interested CRCs provided informed consent. Semi-structured interviews were audio-recorded and transcribed verbatim. Interviewees discussed their PRO-specific skills, responsibilities, challenges, procedures, PRO training received and training needs. Recruitment continued until data saturation. Transcripts underwent content analysis; codes were applied to organise interview content inductively and deductively by RMB and 20% were checked by DK. The study team agreed on the final code structure.
**Results**
Twenty participants (19 female) were interviewed (mean 9.3 years' experience) with professional training in nursing ($n = 12$), science/research ($n = 4$) or both ($n = 4$)). Participants worked in medical oncology ($n = 10$), haematology ($n = 5$), radiotherapy ($n = 4$), and endocrinology ($n = 1$) departments. Skills and responsibilities: All CRCs described organisational and communication skills, the ability to multi-task and work around patient needs. Differences included whether CRCs explained the purpose of PRO assessments to patients, which may result in bias if patients alter their responses if patients believe it will impact their care. There were also differences in assistance provided to patients; some CRCs read questions aloud and recorded patient responses, some paraphrased questions, others excluded patients who could not independently self-complete. This may lead to bias as a result of missing data from sicker patients, or differences in explanations of question meaning. Some CRCs pursued responses for accidentally missed questions; potentially leading to differences in data quality across sites. Some CRCs checked for concerning data or general outcome profile, whereas others felt questionnaires should be kept confidential and not checked, which may lead to bias if these CRCs adapted procedures of care in response to PRO data. Challenges: CRCs described challenges with electronic PRO assessment, non-English-speaking patients, dealing with patients' relatives who inappropriately attempted to complete questionnaires, and patient unwillingness to complete questionnaires. Inconsistencies in data collection and the nature of challenges experienced supports the need for increased PRO-specific training. Training: PRO-specific training received varied considerably; ranging from dedicated PRO training (study-specific or general); PROs being addressed in good clinical practice or nursing training; informal, on-the-job training from colleagues; and no PRO training. Many agreed that additional training was needed to improve current practices.

**Conclusion**

Differences between trials in PRO administration are expected, but the described differences between CRCs regarding communication, patient assistance and checking are concerning as they may lead to various forms of bias and poor data quality. PRO training received varied considerably between CRCs and may be a key reason for these differences. Our findings highlight the importance of providing clear, PRO-specific guidance to CRCs.

### O31

#### Why use cdisc for trials not submitted to regulators? Lessons from the experience of an academic clinical trial unit

Karl Wallendszus, William Stevens, Martin Landray
University of Oxford
**Correspondence:** Karl Wallendszus
*Trials* 2017, **18(Suppl 1):**O31

**Background**

The Clinical Data Interchange Standards Consortium (CDISC) data standards for clinical trials (http://www.cdisc.org/) are widely used in the pharmaceutical industry and are now mandatory for FDA submissions of studies started from December 2016. Relatively few trials run by academic groups are submitted to regulators for marketing authorisation, and the adoption of CDISC standards by them is substantially lower than in industry. We present the advantages and disadvantages of using CDISC standards in the light of the experience of the CTSU in the University of Oxford across multiple phase II to IV trials ranging from 400 to 30,000 participants.

**Experience**

CTSU first used CDISC when providing Study Data Tabulation Model (SDTM) data for an FDA submission after the main analyses had been developed without using CDISC. The resulting discrepancies between CDISC and non-CDISC analyses took considerable effort to resolve. In subsequent studies, whether or not regulatory submissions are planned, a more systematic approach has been used. Collected data is mapped to SDTM datasets, from which Analysis Data Model (ADaM) datasets are derived. All analyses and complex reports, both during and at the end of the trial, are performed on these ADaM datasets. This approach has been successfully employed for a number of large trials at different stages (completed, currently undergoing analysis and ongoing), which together have randomized over 70,000 participants, and more recently to partially convert some legacy studies to CDISC for particular analyses.

**Benefits**

(1) CDISC standards eliminate the need for an organisation to develop its own data standards, and, since they are developed by a wide range of stakeholders over many years, are more comprehensive and coherent than any single organisation is likely to achieve. (2) Analysis and validation tools are available which support CDISC standards. (3) CDISC-compliant datasets are well documented, so statisticians, data analysts and researchers can easily switch between studies without having to learn a new data schema. (4) The effort required to respond to queries about CDISC-compliant data is consistently less than that for non-compliant data. (5) CDISC standards provide a useful common framework for data sharing and long term data and metadata storage. (6) The large CDISC user community is a valuable source of support.

**Costs**

The most significant cost of using CDISC standards is staff training, both technical and on the value of using CDISC. When processing data, extra effort is required to ensure compliance with the standards. Where data are not collected using CDISC, a labour-intensive mapping stage is required, which is more onerous the later in the study life cycle it is done.

**Discussion**

We find the considerable investment required for CDISC at the start of a study, particularly when CDISC is first used, to be worthwhile in view of the benefits which are seen later. Because of the reuse of metadata and controlled terminology, costs are lower for subsequent studies. The benefits of CDISC for data analysis and reporting are maximised when their needs are built into the systems used to capture and process the data.

### O32

#### Prioritising recruitment in randomised trials: the priority study- an Ireland and UK priority setting partnership

Patricia Healy[1], Sandra Galvin[2], Shaun Treweek[3], Caroline Whiting[4], Beccy Maeso[4], Paula Williamson[5], Derek Stewart[6], Derick Mitchell[7], Joan Jordan[8], Mary Clarke-Moloney[9]
[1]14NIHR National Dental & Oral Health Speciality, Clinical Research Network/University of Leeds; [2]HRB Trials Methodology Research Network (Ireland) ; [3]TrialForge, University of Aberdeen; [4]James Lind Alliance; [5]University of Liverpool/MRC Trial Methodology; [6]5NIHR Clinical Research Network Associate Director for Patient and Public Involvement; [7]Irish Platform for Patients Organisations, Science and Industry (IPPOSI) ; [8]EUPATI trainee/IPPOSI; [9]Health Research Institute, University of Limerick
**Correspondence:** Patricia Healy
*Trials* 2017, **18(Suppl 1):**O32

**Objectives**

To identify unanswered questions around trial recruitment, and then prioritise those that stakeholder groups including members of the public, recruiting clinicians and researchers, agree are the most important.

**Background**

The PRioRiTy study - Priority Setting Partnership (PSP) included stakeholders involved in all aspects of clinical trial recruitment; members of the public approached to take part in a randomised trial or who have sat on randomised trial steering committees, health professionals and research staff with experience of recruiting to randomised trials, people who have designed, conducted, analysed or reported on randomised trials and people with experience of randomised trial methodology.

**Methods**

This partnership involved eight key stages: (i) formulating the PSP idea and identifying a unique, relevant prioritisation area within clinical trial methodology (ii) establishing an oversight Steering Group (iii) identifying and engaging with partners and stakeholders (iv) formulating an initial list of uncertainties from a stakeholder survey (v) collating the uncertainties into research question format (vi) checking existing research evidence to confirm that the questions are a current recruitment challenge (vii) shortlisting questions in an interim priority setting exercise through another survey of stakeholders and (viii) final prioritisation through a face to face workshop with stakeholders to agree a top 10 list of priorities of methodological uncertainties around trial recruitment. Both surveys were open to all stakeholders and were disseminated through national clinical trial research networks, patient groups, funding bodies and other relevant stakeholder channels including social media and direct emails.

**Results**

A total of 1,880 questions were extracted from 790 survey respondents, which after merging duplicate questions, was reduced to 496 questions. Merging appropriate questions together and excluding questions asked by fewer than 15 people and/or fewer than 6 of the 7 stakeholder groups resulted in 31 unique research questions. All questions were retained after confirming a lack of relevant, up to date research evidence addressing the question.

Currently (Nov 2016), the partnership is undergoing the interim prioritisation process in which stakeholders are shortlisting the top 10 questions they regard as important uncertainties. The top 10 priorities of methodological uncertainty around trial recruitment will be agreed at a final prioritisation stakeholder workshop scheduled for 1st December 2016. Full results will be available for presentation at ICTMC 2017.

**Conclusion**

Despite the global problem of inadequate recruitment to randomised trials, there is little evidence to guide researchers on decisions about how patients are recruited. A comprehensive, rigorous and inclusive process has been undertaken, with participation from key stakeholders, including members of the public. Priority areas of focus in trial recruitment methodology have been identified by those for whom it matters most. The Top 10 list should inform the scope and future activities of funders and researchers in the area of trial recruitment methodology.

**Sponsorship**

This project was funded by the Health Research Board (Ireland) Knowledge Exchange and Dissemination Scheme Award 2015 and was supported by the James Lind Alliance and NIHR.

### O33

#### Valuing the effect sizes hypothesized in phase 3 trials of targeted therapies in oncology

Nicola Lawrence[1], Felicia Roncolato[2], Andrew Martin[2], Martin Stockler[2]
[1]University of Sydney; [2]NHMRC Clinical Trials Centre, University of Sydney
**Correspondence:** Nicola Lawrence
*Trials* 2017, **18(Suppl 1):**O33

This abstract is not included here as it has already been published.

### O34

#### The changing world of clinical trials 2003–2017: a view from the aspect trial

Gavin Reilly[1], Adelyn Wise[2], Stephen Attwood[3], Claire Scudder[2], Sharon Love[2]
[1]The Centre for Statistic in Medicine, University of Oxford; [2]University of Oxford; [3]School of Medicine, Pharmacy and Health, Durham University
**Correspondence:** Gavin Reilly
*Trials* 2017, **18(Suppl 1):**O34

**Background**

The past two decades have seen dramatic changes in clinical trial conduct and methodology. From trial regulation to data analysis, the rapid rise of randomised control trials (RCTs) has introduced many new techniques. Some methods of designing and conducting RCTs have been widely adopted, whereas other ideas have been used sparingly, despite their promise. Large-scale phase III trials are no different. We present a case study to highlight the changes experienced in designing, setting up, and conducting a large-scale multicentre phase III trial over 14 years.

AspECT is a phase III RCT investigating the use of aspirin and Proton Pump Inhibitors to prevent oesophageal cancer and death in Barrett's Oesophagus patients. Trial follow-up will end in May 2017, 14 years since design and set-up began in 2003. Many challenges have been encountered and addressed by the trial team over this time. With the trial's close, we reflect on its set-up and management, to identify lessons learned and discuss these issues in relation to future trials.

**Methods**

We conducted semi-structured interviews with researchers involved with the set-up and running of the trial at any stage of its 14 years, such as trial coordinators, trial statisticians, and clinicians. The interview structure ensured each individual was asked to address the same key topics and also allowed them to provide their personal views of the changes in trial methodology and set-up. Thematic analysis identified the major challenges experienced by the respondents.

**Results**

Interviews conducted with the current trial coordinator, current trial statistician, and a clinician involved throughout the trial's history revealed the 14-year lifespan of the trial and regulation changes in this time to be the main challenge. AspECT was begun before the current clinical trials regulations were published as Statutory Instrument 2004/1031. The regulations are set to change again soon.

Themes reported included the difficulty in maintaining knowledge of the trial with changing PI's and study nurses in the hospital sites, maintaining and auditing a high quality database over the trial lifespan, handing over study roles, dealing with an ever-evolving and sceptical clinical world, and adapting to the changing processes for obtaining national regulatory and local R&D approval and multicentre trial set-up. Also, the trial management had to repeatedly react to poor quality epidemiology claims about drug reactions or side effects, or unproven benefits. We will also discuss issues around the evolving world of methodology, including placebo blinding costs today compared to during trial set-up and the potential for Studies Within A Trial, an emerging research movement to make better use of trial data.

**Conclusions**

Many changes have occurred since the set-up of AspECT in 2003. Some of these changes have made trials more transparent and safer for the patients involved, benefiting the medical research world. However, some changes may deter and slow good research, inhibiting the emergence of new treatments. Our experiences over a 14-year phase III trial highlight the issues experienced by the trial research community and are presented to inform the design and conduct of similar future trials.

### O35

#### An ethical analysis of the first trial: addressing ethical challenges in pragmatic cluster randomized trials of policy interventions targeting healthcare providers

Austin Horn[1], Cory E. Goldstein[2], Monica Taljaard[3], Charles Weijer[2]
[1]Western University; [2]Western University, Rotman Institute of Philosophy; [3]University of Ottawa, Ottawa Hospital Research Institute
**Correspondence:** Austin Horn
*Trials* 2017, **18(Suppl 1):**O35

**Background**

The Flexibility in Duty Hour Requirements for Surgical Trainees (FIRST) trial was a pragmatic cluster randomized trial (CRT) involving 117 surgery residency programs in the United States. It evaluated non-inferiority of flexible duty-hour policies compared to standard restricted duty-hour policies with respect to surgical resident well-being and patient safety. Investigators concluded that flexible duty-hour policies were non-inferior to standard duty-hour policies. The ethics of the FIRST trial have been vehemently debated. One commentator describes it as "among the most unethical research studies [he has] ever seen." Another argues that it was "not just ethical but laudable to comparatively evaluate duty-hour policies. The FIRST trial illustrates the complex ethical challenges posed by CRTs of policy interventions involving healthcare professionals.

**Objectives**

The Ottawa Statement, published in 2012, provides researchers and research ethics committees (RECs) with specific guidance for the ethical design and conduct of CRTs. Our objectives are to: (1) review critically the FIRST trial controversy; (2) apply the Ottawa Statement to the FIRST trial; and (3) identify issues not adequately addressed by the Ottawa Statement, thus requiring further analysis and guidance.

**Results**

Objective 1: Controversy erupted following publication of the FIRST trial in New England Journal of Medicine in 2016. Critics accused the investigators of "egregious ethical and regulatory violations," arguing that the flexible duty-hour intervention knowingly exposed residents and their patients to increased risks of serious harms. They decry the decision by Northwestern University's REC exempting the trial from human subjects research regulation, calling it a "colossal failure" of all participating RECs. Critics also denounce the resultant consent waiver as a violation of the ethical principle of respect for persons. Defenders of the FIRST trial argue that the flexible duty-hour intervention did not pose a greater risk to participants, and conditions for the waiver obtained. We critically review the FIRST trial controversy, finding that commentators fail to identify the relevant ethical issues systematically. Objective 2: We examine the utility of the Ottawa Statement for CRTs of policy interventions involving healthcare providers. We find that the Ottawa Statement provides much-needed clarity by identifying systematically the ethical issues common to all CRTs, including: justifying the cluster design, identifying research participants, consent, gatekeepers, benefit-harm analysis, and vulnerable participants. Objective 3: We show how the FIRST trial raises unique ethical issues not adequately addressed by the Ottawa Statement. For instance, does clinical equipoise obtain when a novel policy is compared to an existing policy that has little or no evidence-base? How should researchers and RECs conceptualize healthcare providers targeted by policy interventions? Are they obligated to participate in research? If so, what are the implications for consent? Alternatively, should healthcare providers be conceptualized as vulnerable participants? A power-differential often exists between healthcare providers and their superiors, particularly when providers are trainees or employees. Does this relationship undermine the validity of their consent? If so, what safeguards might be implemented to ensure protection of healthcare providers, while at the same time ensuring that important research proceeds both feasibly and expeditiously?

### O36

#### Mediation analysis to explore causal mechanisms in trials of complex interventions

Deborah DiLiberto, Charles Opondo, Diana Elbourne, Elizabeth Allen
London School of Hygiene and Tropical Medicine
**Correspondence:** Deborah DiLiberto
*Trials* 2017, **18(Suppl 1):**O36

**Background**

There is increasing enthusiasm for the use of mediation analysis in the secondary analysis of complex interventions with the aim of isolating the causal mechanisms through which an intervention produces the outcome of interest.

Recent guidance from the Medical Research Council (MRC) on evaluating complex interventions suggests that Randomised Controlled Trials (RCTs) should be complemented by process evaluations which might provide evidence about the possible causal mechanisms that produce intervention effects. Process evaluations often include the development of an intervention theory of change - a description of how the intervention inputs, change mechanisms and context are hypothesised to produce the intended outcomes. It is recommended that these intervention theories are represented and evaluated using 'logic models' which visually demonstrate the pathway of effect between intervention inputs and intended outcomes. Mediation frameworks are potentially useful here as they can generate tests of the logic model and hence the intervention theory of change.

The traditional framework for mediation analysis applies structural equation modelling (SEM). While SEM has been valuable because of its relatively simple approach to analysing mediators, recent advances in mediation theory have shown that the SEM approach has theoretical limitations which make it insufficient for more complex applications. An alternative nonparametric approach is based on the 'potential outcomes framework' and applies the logic of counterfactuals in an attempt to identify causal pathways.

**Materials and methods**

The PRIME intervention was designed to attract patients to seek care and to improve the quality of care, including for the diagnosis and treatment of malaria, delivered at public health centres. The complex, multi-component intervention focused on ensuring access to appropriate treatment and diagnostic tests at health centres through a range of components to improve provider behaviour and health centre operations. Following the MRC guidance, the impact of the PRIME intervention was comprehensively evaluated including a rigorous outcome evaluation; a cluster Randomised Controlled Trial (cRCT) with data from community cross-sectional surveys, and a parallel mixed-methods 'process' study. Here we explore the use of the 'potential outcomes framework' to undertake a mediation analysis of the PRIME intervention theory of change.

**Results, conclusions and future research**

We demonstrate the challenges and limitations of mediation analysis in this context and suggest a cautious approach for incorporating the ideas of mediation analysis into evaluations of complex interventions. Building on this experience, we discuss the utility of the suggested approach in the design process of the UPAVAN trial- a three-year, four arm cRCT to assess the impact and cost-effectiveness of three variants of an innovative intervention to improve agricultural and nutrition outcomes, with an integrated theory of change.

### O37

#### Standardised taxonomy for the classification of trial outcomes within core outcome sets and cochrane reviews

Susanna Dodd[1], Paula R. Williamson[1], Jane Blazeby[2], Mike Clarke[3]
[1]University of Liverpool; [2]University of Bristol; [3]Queen's University, Belfast
**Correspondence:** Susanna Dodd
*Trials* 2017, **18(Suppl 1):**O37

**Background**

The COMET (Core Outcome Measures in Effectiveness Trials, http://www.comet-initiative.org.uk) Initiative brings together people interested in the development and application of agreed standardised sets of outcomes, known as "core outcome sets" (COS). These sets represent the minimum that should be measured and reported in all clinical trials of a specific condition, and are also suitable for use in clinical audit or research other than randomised trials. One of the successes of COMET has been the development of a publicly available searchable database of completed and ongoing projects in COS development. This database is currently searchable by population, intervention and condition, but as yet has not been categorised according to outcome (the fourth of the essential elements that should be defined for a trial, according to the PICO model). Similarly, outcomes in trials registries (including the EU Clinical Trials Register, ClinicalTrials.gov and ISRCTN registry) can be entered as free text only, leading to inconsistencies. Ninety percent of queries related to requests to register a trial relate to outcomes (Alison Cuff, ISRCTN, personal communication). Standardised terminology to describe outcomes is starting to come into use in pre-clinical research (Robinson et al. "The Human Phenotype Ontology: A Tool for Annotating and Analyzing Human Hereditary Disease" (2008) The American Journal of Human Genetics 8: 610–615), but there is currently no consensus on how trial outcomes should be classified. The lack of a standard taxonomy relating to trial outcomes impedes the ability to efficiently and effectively search the literature. A standard classification system for trial outcomes would facilitate literature searches to identify the use of a particular COS, as well as being of use to reviewers when annotating Cochrane Reviews according to outcome, as part of the PICO review description (via the Cochrane Linked Data Project, http://linkeddata.cochrane.org/).

**Methods**

The COS outcome classification project involves the extraction of all core outcomes/domains from existing COS through the COMET database and reviewing the systematic reviews of outcomes in the COMET database to determine how outcomes were classified. Existing conceptual models (including The International Classification of Functioning, Disability and Health (ICF), Patient-Reported Outcome Measurement Information System (PROMIS) and the Wilson and Cleary framework) will be reviewed for suitability, with a view towards developing a standardised ontology for classification of research outcomes.

**Results**

Results on the progress of this project, in terms of the classification of COS outcomes within the COMET database and development of a standardised outcome taxonomy for the classification of COS outcomes, will be reported, along with any conclusions drawn during discussions which took place during the 'Outcome Classification' session at COMET VI (November 2016).

**Conclusions**

The ultimate aim of this project is to agree on standardising terminology and definitions through consensus among different stakeholders, including patients, clinicians and methodologists. Progress made to date on achieving this aim will be presented.

### O38

#### How might patient and public involvement (PPI) improve recruitment and retention in surgical trials? A qualitative study exploring the views of trial staff and PPI contributors

Joanna Crocker[1], Keira Pratt-Boyden[2], Jenny Hislop[2], Sian Rees[3], Louise Locock[1], Sophie Petit-Zeman[4], Alan Chant[5], Shaun Treweek[6], Jonathan A. Cook[7], Nicola Farrar[8]
[1]NIHR Biomedical Research Centre and Nuffield Department of Primary Care Health Sciences, University of Oxford; [2]Health Experiences Research Group, Nuffield Department of Primary Care Health Sciences, University of Oxford; [3]Health Experiences Institute, Nuffield Department of Primary Care Health Sciences, University of Oxford; [4]NIHR Oxford Biomedical Research Centre and Unit; [5]Patient Partner; [6]Health Services Research Unit, University of Aberdeen; [7]Surgical Intervention Trials Unit, Nuffield Department of Orthopaedics, Rheumatology and Musculoskeletal Sciences, University of Oxford and MRC ConDuCT-II Hub for Trials Methodology Research, School of Social and Community Medicine, University of Bristol; [8]Surgical Intervention Trials Unit, Nuffield Department of Orthopaedics, Rheumatology and Musculoskeletal Sciences, University of Oxford
**Correspondence:** Joanna Crocker
*Trials* 2017, **18(Suppl 1):**O38

##### Introduction
Clinical trials are commonly affected by slow recruitment, leading to prolonged study duration and increased cost, and also attrition, which weakens trials. It has been suggested that patient and public involvement (PPI) in designing and/or conducting trials could help to alleviate these problems, yet PPI is often implemented with little planning or thought as to the role of PPI contributors and how their input might benefit the trial. We are developing a PPI intervention aimed at improving recruitment and retention in surgical trials, which can be particularly difficult to recruit to. As part of this process we explored surgical trial staff and PPI contributors' views regarding how PPI might achieve such improvements.

##### Methods
Participants were recruited via surgical and PPI networks and organisations. 6 focus groups (4 with surgical trial staff and 2 with PPI contributors) were facilitated at 4 sites across the UK. PPI contributors unable to attend focus groups were offered a one-to-one interview in person or by telephone. All participants as well as those unable to attend focus groups were invited to submit additional comments in writing. Verbatim transcripts and textual data were analysed thematically by three researchers who identified emerging themes.

##### Results
Fifty-four people took part, of whom 31 were surgical trial staff (15 trial managers/coordinators, 7 investigators, 7 research nurses, 1 clinical trial administrator and 1 research associate), 21 were PPI contributors and 2 were PPI coordinators. Staff took part in focus groups at surgical research centres in Oxford (N = 7), Aberdeen (N = 8), Bristol (N = 9) and Birmingham (N = 7), while PPI contributors took part in one of two focus groups at the Library of Birmingham (N = 6 and N = 8) or a one-to-one interview (N = 7). Eleven people submitted written contributions.

Drawing on their experiences, participants proposed several ways in which PPI contributors could improve recruitment to trials: improving the relevance of the research question; informing trial design including the benefits and burdens for participants, recruitment process (where, when, who) and participant information sheets; assessing patients' willingness to take part; directly recruiting participants; and publically endorsing the trial

Suggested ways in which PPI contributors could improve retention in trials included: changing which outcomes are collected and how; assessing the burden or acceptability of follow-up methods to potential participants; suggesting appropriate incentives; communicating with participants during the trial (e.g. newsletter updates, explaining why it is important to stay in the trial); challenging regulatory barriers to adopting new data collection methods.

However, it was also suggested that PPI contributors could be unhelpful in some circumstances, for example if involved too late (e.g. only in developing informed consent documents), if their literacy level is too high, or if they are not from the trial's target population.

##### Conclusion
Participants proposed a variety of ways in which PPI contributors might improve recruitment and retention in surgical trials, also giving examples of when PPI might be unhelpful or even harmful. Trialists should carefully consider how to involve patients and members of the public most effectively.

### O39

#### Maximising information in pressure ulcer prevention trials using multi-state modeling

Linda Sharples, Isabelle Smith, Jane Nixon
University of Leeds
**Correspondence:** Linda Sharples
*Trials* 2017, **18(Suppl 1):**O39

##### Introduction
Long stay in hospital and poor mobility put people at risk of developing pressure ulcers (PU) at a number of areas of the body (buttocks, heels etc.). PUs result in admission to hospital, prolonged hospital stay, impaired quality of life, significant cost to the NHS and have been described as a key quality indicator for the Department of Health. Motivation PUs are classified on a 4 point ordinal scale from 1–4 with 4 the most severe category. In RCTs skin assessment for onset or progression of PUs is scheduled to take place at a number of fixed time points, resulting in serial measurements of PU categories at up to 14 skin sites. Thus each patient typically has 50–100 PU assessments during trial follow-up. However, due to administrative and patient-related events, scheduled measurements may be missed or only partially completed. This results in observation times that are different for different patients and different skin sites, and intervals between assessments may vary. Moreover, the reasons for missing data may not be independent of the PU category. Typically, the primary outcome for PU prevention trials is the time from randomisation to the first category 2 PU at any skin site, so that the 50–100 assessments per patient are reduced to a single outcome measurement. This outcome is inefficient in that it ignores the information from serial measurements and multiple skin sites; it may also be biased due to the interval censoring between observations and the missed assessments. Thus sample sizes for PU prevention trials may be larger than necessary, resulting in delays in getting effective treatments into practice, or in ruling out ineffective treatments. Aim The aim of this study was to investigate the use of multi-state models of PU onset and progression, in order to provide less biased and more efficient estimates of treatment effects.

##### Methods
In this study we show how to design a PU prevention trial and analyse resulting data. Specifically, multi-state models that incorporate both the sampling process (availability and completeness of follow-up) and the observed PU categories at all skin sites are developed. The assumptions that are required for different models, their implications and their validity in this context are presented. Methods for estimation of commonly used outcome measurements within this framework are presented. Through re-analysis of an existing serial measurement from a PU prevention study we demonstrate how fixed covariates (e.g. treatment group and stratification factors) can be incorporated into the analysis. Efficiency is explored using simulation studies based on the example trial to demonstrate potential influence on sample size estimates, of using more informative designs and analyses.

##### Conclusion
Given the current difficulties in recruiting patients to RCTs it is important to make best use of the rich data that accrue during trials. Important reductions in sample size for PU trials may be possible if all available observations are included in the analysis.

### O40

#### Quality control of SDTM domain mappings from electronic case report forms

Noga Lewin, Miebi Eradiri, Sheena Aris, Angela Soriano, Gaurav Sharma, Jill Barrett, Heather Hill, Marian Ewell, Noble Shore, Abigail G. Matthews
Emmes Corporation
**Correspondence:** Noga Lewin
*Trials* 2017, **18(Suppl 1):**O40

The Study Data Tabulation Model (SDTM) defines a standard structure for submission of electronic clinical trial data to a regulatory authority, such as the FDA. These electronic listings of individual observations comprise the essential data reported from a clinical trial and are submitted with the analysis datasets.
The Clinical Data Interchange Standards Consortium (CDISC) team at the Emmes Corporation, developed a novel process to map data collected in electronic case report forms (eCRFs) to the SDTM paradigm with these unique advantages: the mapping specifications are developed alongside the design of the CDASH conformant CRFs; Advantage eClinicalSM, Emmes' form building and data capture and management suite, provides an intuitive user interface that permits a non-programmer to specify the mapping to SDTM - this process is completed before the initiation of data collection; then the mapping is executed on the production data in an automated fashion at least daily while the trial is accumulating data, and the results are written to a tabulation database. This enables the use of SDTM data structures for oversight and safety reporting. The use of standardized data tables throughout the life cycle of the study yields efficiencies in statistical reporting and reduces the timeframe required for delivery of the final databases and code at the end of the study.
Quality control of the mapping process is partially automated. The proposed automated QC report algorithm reduces the amount of work involved in validating the mapping against a discrete set of rules (e.g. every variable is mapped, no cell in the mapping tool is left blank, each required variable in the domain is mapped, compare mapping to a gold standard - a protocol that was tested and can serve as a template, all fields in the mapping entries start with the form code, all subjects belong to the protocol).
The program creates a complete set of reports for the entire protocol, for each combination of domain and eCRF. Each report has a summary of the failed tests and hyperlinks are utilized so that the tester can easily navigate the report, see the description of the test, the mapping code used, and the relevant data, as well as the reason for failure. This program has been utilized and tested on Emmes platforms and has consistently helped to identify errors while saving testers time. It provides all the information for the tester to evaluate the results and relevant code if they want to execute it themselves. The more accurately this QC of the SDTM mapping is done, the more efficient subsequent testing will be.

### O41

#### Changing roles and relationships within trial oversight: an ethnographic study of eight clinical trials facing challenges

Anne Daykin[1], Lucy E. Selman[1], Helen Cramer[1], Sharon McCann[2], Gillian W. Shorter[3], Matthew R. Sydes[4], Carrol Gamble[5], Rhiannon Macefield[6], Alison Shaw[1], J. Athene Lane[6]
[1]University of Bristol; [2]Formerly: Health Services Research Unit, University of Aberdeen; [3]Ulster University; [4]1 MRC Clinical Trials Unit at UCL, Institute of Clinical Trials and Methodology and London Hub for Trials Methodology Research; [5]MRC North West Hub for Trials Methodology Research, Institute of Translational Medicine, University of Liverpool; [6]MRC ConDuCT Hub for Trials Methodology Research, School of Social and Community Medicine, University of Bristol
**Correspondence:** Anne Daykin
*Trials* 2017, **18(Suppl 1):**O41

**Background**
The Medical Research Council (MRC) 1998 Guidelines for Good Clinical Practice in Clinical Trials recommend that, in the UK, trial oversight is managed by three committees: a trial management group (TMG), trial steering committee (TSC) and data monitoring committee. This model is endorsed by several UK funders. According to these Guidelines, the Principal Investigator (PI) has the central role and overall responsibility for the co-ordination and day-to-day management of the trial. However, recent quantitative evidence suggests heterogeneity in trial oversight and some confusion regarding the diverse roles of stakeholders, indicating the MRC Guidelines may be outdated. Aim: To explore roles and relationships in trial oversight to ascertain current practice and suggest recommendations to support an update of the MRC guidelines.
**Methods**
Using an ethnographic study design, 8 TSC and 6 TMG meetings from eight trials were observed and audio-recorded and 65 semi-structured interviews conducted with 51 purposively sampled key informants (members of the trials' TSCs/TMGs and other relevant informants). Selected trials represented a range of clinical topics and were all dealing with challenging scenarios (e.g. recruitment issues, protocol deviation or amendments). Data were analysed thematically and findings triangulated and integrated to give a multi-perspective account of current oversight practices.
**Results**
The primary themes identified were the role of the CTU in trial oversight and power issues within trial oversight. The central role of the PI in the MRC Guidelines was not reflected in our data. Instead, the clinical trials units (CTUs) supporting the trials took on the responsibilities of the PI outlined in the Guidelines. We observed CTUs performing additional roles such as advising the PI on research methodology, being the main channel of communication for the trial and arbitrating between the PI and other trial oversight groups. The perceived power of individual oversight groups over trials was influenced by the behaviour of funding bodies. For example, by appointing their own TSC members, funders were viewed as reducing the power of TSCs and trial sponsors to make independent decisions. This could lead trial teams to fear their funder's power and be guarded in their communication with the funder. Trial oversight groups had differing views regarding who has the power to stop trials. The sponsors, independent TSC members, TSC chairs and funders all believed they had the power to terminate the trial and that the buck stopped with them.
**Conclusions**
The roles and relationships of trial oversight groups have changed since the publication of the MRC Guidelines in 1998. We found that CTUs, and not the PI or TMG, had responsibility for the day to day management of trials, and this should be acknowledged when the MRC Guidelines are revised. The TSC, funder and sponsor all have the power to stop trials, and acknowledging this may be useful to raise the awareness of all the parties concerned, in order to facilitate the constructive collaboration of trial oversight groups.

### O42

#### Outcome-adaptive randomization: some ethical issues

Julius Sim
Keele University
*Trials* 2017, **18(Suppl 1):**O42

In a conventional randomized controlled trial (RCT), randomization is in fixed, usually equal, proportions throughout. As judgments of relative treatment superiority are suspended until the end of the study, there is no reason to use accruing data to adjust allocation, other than in planned interim analyses. In trials using outcome-adaptive randomization (OAR), allocation to treatment arms is repeatedly adjusted, to weight allocation to the hitherto more effective treatment. This has the ethical merit of seeking to maximize the number of patients experiencing a treatment success. However, this apparent ethical advantage is offset by other issues concerning equipoise, informed consent and the methodology of the trial.
**Equipoise**
Equipoise indicates genuine uncertainty as to the relative merit of the treatments being tested. In a conventional RCT this is established at the outset and only revisited if interim analyses occur. Hence, no patient is knowingly disadvantaged by allocation to either treatment.

In OAR, equipoise is re-examined repeatedly, as it determines allocation. Accordingly, allocation is increased to the treatment showing superiority - but patients are nonetheless still knowingly allocated to the apparently inferior treatment, albeit in smaller numbers, and thereby disadvantaged. Whilst action is taken in response to changes in equipoise, equipoise is not thereby completely restored.
Additionally, at the end of the trial, OAR may have required a larger sample than a conventional RCT; whilst the proportion of participants disadvantaged by a poorer outcome may decrease, the number doing so may increase.

**Consent**

The moral force of consent depends on information about the trial being adequately understood. Empirical research suggests that this is hard to achieve, but it is likely to be even harder if one has to explain how randomization is continually readjusted in relation to outcomes. This is likely to increase the 'therapeutic misconception': participants' tendency to think that treatment allocation is based on their individual clinical need, rather than being (semi-)random. A further complication is that the information required by new participants will vary over the course of the trial, as it should reflect the accruing outcomes within the trial (rather than just external evidence that may become available). Conveying appropriate information is therefore challenging, and if not achieved, the value of consent will be reduced accordingly.
Crucially, simply telling participants that allocation reflects accumulating evidence without also indicating which specific treatment is currently favoured may be insufficient for consent to be informed.

**Methodological issues**

A study is only ethical if it generates methodologically robust findings. However, some features of OAR may have undesirable methodological implications. Thus, the fact that differing information should be given to patients entering the trial at different times may lead to contamination, or, coupled with the changing allocation ratio, may be a confounder. Additionally, the need to monitor outcomes repeatedly to determine allocation may limit the degree of blinding achievable.

**Conclusion**

Initially, OAR appears to have ethical merit in terms of maximizing the number of participants who receive the superior treatment within the trial, but this claim needs to be tempered by other ethical considerations.

### O43

#### Success of randomizing trial participants to disclosure of allocation early or late: a methodological study to investigate performance bias

Barnaby Reeves[1], Rosie A. Harris[1], Leila Rooshenas[1], Kate Ashton[1], David Hutton[1], Chris A. Rogers[1], Natalie S. Blencowe[1], Jane M. Blazeby[1], Bluebelle Study Group[2]
[1]University of Bristol; [2]Universities of Bristol & Birmingham
**Correspondence:** Barnaby Reeves
*Trials* 2017, **18(Suppl 1):**O43

**Background**

Performance bias arises in randomized controlled trials (RCTs) if care providers implement co-interventions differentially on the basis of their knowledge of participants' treatment allocation. It can especially affect surgical trials because it is rarely possible to blind surgeons and randomization within the operating theatre environment (i.e. as close to intervention delivery as possible) poses logistical challenges. This study aimed to measure and assess the influence of performance bias in a surgical RCT.

**Methods**

Participants having general abdominal surgery or caesarean section at five hospital sites are being recruited to a pilot RCT investigating the influence of wound dressings on surgical site infection. They are randomized twice: first, to the type of dressing to be applied (simple wound dressing, glue-as-a-dressing, or no dressing) and, second, to the time of disclosing the allocation (before or after the surgeon closes the wound at the end of operation). The protocol specifies that users should log into the randomization system at the beginning of surgery. The user is then either given the allocation or asked to log in again after wound closure to obtain the allocation. When logging in again, the user has to enter the time of wound closure. Acceptability of the double randomization is assessed from three sources of information: times for system log-on, knife-to-skin and wound closure from the trial database; in-depth interviews with health care professionals; feedback from participating centres about their ways of working.

**Results**

At present, 55 and 57 participants (before and after wound closure) have been allocated to no dressing; 52 and 54 to simple dressing; 54 and 54 to glue-as-a-dressing. Nine allocation disclosure deviations were identified. For 5/165 participants randomized to allocation disclosure AFTER wound closure, system log-on times for obtaining allocation were >50 minutes before the manually entered time of wound closure; another 2 participants had first and second log-on times <2 minutes apart. For 2/161 participants randomized to allocation disclosure BEFORE wound closure, the manually entered 'knife-to-skin' time preceded the first system log-on time by >90 minutes. Informants were not specifically aware of any attempts to work around the double-randomisation system; some were aware that such behaviours could be detected, and one questioned why one might try to 'cheat the system', acknowledging this as a protocol deviation. Practical issues, such as limited internet access in theatre or no one available to log into the database, were also reported. Feedback from two centres suggested that theatre staff are ringing a research nurse outside theatre to log-on when required. On at least one occasion, a surgeon first logged in after wound closure, to avoid having to log-on twice. Centres have also reported occasional difficulties in accessing the database from theatres. Generic usernames for randomization only, accessible using a mobile phone, were offered to improve access.

**Conclusions**

Timings collected during the trial demonstrate good adherence to the double randomization. Methods adopted by research personnel in order to adhere may not be practicable in a large trial. Generic access for randomization may facilitate theatre personnel doing this task.

### O44

#### Priority setting for core outcome set development

Sarah Gorst[1], Mike Clark[2], Paula R. Williamson[1]
[1]University of Liverpool; [2]Queen's University Belfast
**Correspondence:** Sarah Gorst
*Trials* 2017, **18(Suppl 1):**O44

**Background**

The Global Burden of Disease Study identified the leading causes of chronic disorders worldwide. If the findings from this study are to guide future health research, it is important to ensure that appropriate outcomes are measured in that research. Core outcome sets (COS) will help to achieve this. COS represent an agreed minimum set of outcomes that should be measured and reported, as a minimum, in all clinical trials for a specific health condition. The application of COS allows the results of clinical trials to be appropriately combined, minimising waste and ensuring that usable evidence is made available. If COS were available for the leading causes of chronic disorders, this should accelerate the impact of research and result in improvements in global health. No prioritisation for COS development has previously been undertaken, therefore this study aimed to identify COS that have been developed in relation to the most prevalent chronic conditions throughout the world, and to highlight areas for future COS development or improvement.

**Methods**

The COMET (Core Outcome Measures in Effectiveness Trials) Initiative promotes the development and application of COS, by including pertinent individual studies in a publically available online database. The COMET database is a unique inventory containing references of planned, ongoing and completed work relating to COS development. In total, there are more than 300 published and ongoing COS

registered in the COMET database. The COMET database was searched to identify published and ongoing COS that might be relevant to the 25 conditions with the highest global prevalence of chronic sequelae identified in the Global Burden of Disease Study.

**Results**

A search of the COMET database identified 33 published and ongoing COS that are relevant to 13 of the world's most prevalent conditions. The majority were developed only with the involvement of people from North America and Europe (n = 27/33). Thirty-one COS involved clinical experts in the development and 18 involved patients. No published or ongoing COS have been identified for the remaining 12 of the 25 most prevalent conditions.

**Conclusion**

This study describes the first approach to identifying gaps in existing COS, and to priority setting in this area. Important gaps have been identified for at least 12 of the 25 most prevalent conditions. The development and application of COS in these areas would provide the foundation for ensuring that appropriate outcomes are measured and reported in clinical trials for these most prevalent disorders worldwide. Without such international consensus on the key outcomes for research in these conditions, new studies might not make a full contribution to improving global health and opportunities to reduce waste in research will be lost. A wider range of perspectives, including those of patients, on existing COS are also needed when not otherwise included. Furthermore, it is evident that COS are failing to include a range of international stakeholders within the development process. Therefore, the inclusion of stakeholders from Asia, South America, Australia, and Africa is an additional gap that future research should aim to address.

### O45

#### Pragmatic integrated randomised controlled trials in screening: experience from a trial in 1.2million women attending breast screening

Sian Taylor-Phillips[1], David Jenkinson[1], Matthew Wallis[2], Janet Dunn[1], Aileen Clarke[1]
[1]University of Warwick; [2]Cambridge Universities NHS Foundation Trust
**Correspondence:** Sian Taylor-Phillips
*Trials* 2017, **18(Suppl 1):**O45

Randomised controlled trials (RCTs) are expensive, the pragmatic integrated randomised controlled trial has been proposed to deliver large scale RCTs at a much reduced cost. In these studies elements of the trial such as recruitment, randomization, intervention, data collection and/or long term follow up are integrated into standard practice to reduce costs and increase potential sample size. These designs are particularly appropriate for screening where practice is standardized and many centres use the same software systems. We present an example of a pragmatic integrated randomised controlled trial design in breast cancer screening, the Changing Case Order to Optimise Patterns of Performance in Screening (CO-OPS) ISRCTN46603370. The study was designed to examine whether breast screening radiologists experience a vigilance decrement of decreasing ability to detect cancer in x-rays with time on task, and whether an intervention to change case order could reduce such an effect. The trial was funded as part of an NIHR postdoctoral fellowship and cost less than £300 k. Of the 80 breast screening centres in England, 46 consented to take part in the trial for 1 year. This included research active centres and those with little experience of research. Consent was at the centre level rather than the individual woman screened, as both intervention and control groups were considered different versions of standard practice as both were implemented in different parts of the NHS. The trial was implemented through the National Breast Screening Service computer system, which is used at all English breast screening centres. The software was adapted to randomise women in batches to intervention or control, and display the cases in the desired order. A total of 1,194,147 women were randomised and analysed. A standard Crystal report was designed to extract trial outcomes from the NBSS computer system. Data extraction was delayed until after each centre completed their annual reports for routine quality assurance, as the datasets are cleaned in preparation for these. Further data cleaning was conducted in collaboration with each centre. As a result there was very little missing data, making up less than 0.1% of the final dataset. This is an example of implementing a successful pragmatic integrated trial in screening. Such trials are effective in situations where some of the following conditions are met: individual informed consent for the trial is not necessary, the intervention itself is inexpensive, trial outcomes are already routinely recorded in a standard way, routine data collection is accurate and audited, and management pathways are standardized and the intervention does not require major changes to these. The advantages of this design are the low cost and large sample size, and the opportunity to involve a greater number of hospitals to increase generalizability.

### O46

#### A value of information approach to optimal design of confirmatory clinical trials

Nigel Stallard[1], Michael Pearce[2], Siew Wan Hee[2], Jason Madan[2], Martin Posch[3], Simon Day[4], Frank Miller[5], Sarah Zohar[6]
[1]Warwick Medical School, University of Warwick; [2]University of Warwick; [3]Medical University of Vienna; [4]Clinical Trials Consulting and Training Limited; [5]Stockholm University; [6]INSERM
**Correspondence:** Nigel Stallard
*Trials* 2017, **18(Suppl 1):**O46

**Background**

Most confirmatory clinical trials are designed so as to achieve a specified power, usually 80% or 90%, for a hypothesis test conducted at a given significance level, which is almost invariably set to be 5% for a two-sided test. Licensing decisions by regulatory agencies are then based on the result of such a significance test informally combined with other information to balance the risk of adverse events against the value of the treatment to future patients. In the setting of a rare disease, recruitment of the number of patients required to achieve conventional error rates for clinically reasonable effect sizes may be infeasible or even impossible, suggesting that the decision-making process should reflect the size of the population for whom the treatment can be used in the future.

**Methods**

We have considered the use of the decision-theoretic value of information (VoI) method to obtain the optimal sample size and significance level for definitive randomised controlled clinical trials in a range of settings, focussing particularly on the impact of different population sizes. For simplicity we have assumed the primary endpoint to be continuous and normally distributed with unknown mean with some normal prior distribution, the latter representing information on the anticipated effectiveness of the therapy available from sources external to the trial itself. We explicitly specify the gain in terms of improvement in primary outcome for patients treated with the a new therapy and compared this with the costs, both financial and in terms of risk of potential harm, of treating patients, either in the trial or in the future if the therapy is approved.

**Results**

We have found that as the size of the population that can be treated in the future increases, the optimal sample size for the clinical trial also increases. If there is a non-zero cost, whether financial or in terms of potential harmful effects, of treating future patients, stronger evidence is required for approval as the population size increases, though this is not the case if the costs of treating future patients are ignored.

**Conclusions**

The results of clinical trials are often summarised by a frequentist hypothesis test conducted at a 5% significance level with the sample size chosen to give specified power of 80% or 90%. These values are arbitrary. We showed how decision-theoretic analysis suggests a more flexible approach with both type I error rate and power (or equivalently trial sample size) depending on the size of the future population for whom the treatment under investigation is intended.

### O47

#### Ethical issues in individual-cluster trials: beyond the Ottawa statement

Cory Goldstein[1], Austin R. Horn[1], Monica Taljaard[2], Charles Weijer[1]
[1]Western University, the Rotman Institute of Philosophy; [2]Ottawa Hospital Research Institute
**Correspondence:** Cory Goldstein
*Trials* 2017, **18(Suppl 1):**O47

The conduct of pragmatic randomized controlled trials is increasing due to their societal importance and their role within the Patient-Centered Outcomes Research Institute (PCORI) initiative "to improve the quality and relevance of evidence available to help patients, caregivers, clinicians, employers, insurers, and policy makers make informed health decisions." Cluster randomized trials (CRTs), in which groups rather than individuals are randomized to intervention and control conditions, naturally tend to be more pragmatic. CRTs may be categorized as "individual-cluster trials" where the intervention is delivered directly to individuals, or "cluster-cluster trials" where interventions are not divisible at the individual-level. The Ottawa Statement is the first comprehensive ethical guidance document specific to CRTs. Whereas the Ottawa Statement generally presumes that informed consent will be sought for individual-cluster trials, such trials' when used to evaluate usual care interventions' raise particular ethical issues that require further analysis and guidance. This paper has three objectives: to (1) describe current practices and reporting of ethical issues in published individual-cluster trials; (2) present an in-depth ethical analysis of an individual-cluster trial randomizing dialysis centres to two different usual care interventions; and (3) identify ethical issues that require further analysis and guidance.

Objective 1: Systematic review of individual-cluster trials Using an electronic search strategy, we identified a random sample of published individual-cluster trials in Canada, the USA, UK, France, Australia and Low and Middle Income Countries. Two reviewers independently extracted details about ethical issues and practices (e.g., justification for the cluster randomized design, prevalence of seeking informed consent, presence and roles of gatekeepers). Practices will be compared over time, between countries, types of clusters and interventions, and other descriptors.

Objective 2: An ethical analysis of the TiME trial the optimal duration for individual hemodialysis treatments in chronic renal failure is currently unknown. The Time to Reduce Mortality in End-Stage Renal Disease (TiME) trial is a PCORI funded individual-CRT in which dialysis treatment centres are randomized to one of two hemodialysis durations (usual care or extended) to evaluate their comparative effectiveness. The main outcome measures are mortality, hospitalization, and quality of life. The trial uses an IRB approved "opt out" approach to informed consent. Applying the Ottawa Statement highlights a range of issues, including justification for the study design, participant identification, informed consent, gatekeeper permission, benefit-harm analysis and protection of vulnerable participants.

Objective 3: Ethical issues that require further analysis and guidance While the Ottawa Statement provides a systematic approach to the ethical analysis of CRTs, we conclude that further analysis and guidance is required for individual-cluster trials of treatments adopted as policy at cluster-level. The TiME trial highlights a number of generalizable ethical issues, including (1) whether there is an appropriate justification for the cluster randomized design (e.g., what justifies adoption of cluster randomization if individual randomization is feasible in principle?), (2) the appropriateness of the consent procedure (e.g., can consent be waived due to pragmatic challenges?), and (3) how we should understand gatekeeper permission (e.g., is gatekeeper permission identical to obtaining proxy consent?).

### O48

#### An annotated guideline to the use of a health economics analysis plan (heap) alongside randomised controlled trial

Melina Dritsaki[1], Alastair Gray[2], Stavros Petrou[3], Susan Dutton[2], Sarah E. Lamb[2]
[1]Oxford Clinical Trials Research Unit, University of Oxford; [2]University of Oxford, Nuffield Department of Orthopaedic Rheumatology and Musculoskeletal Sciences, Oxford Clinical Trials Research Unit, Centre for Statistics and Medicine; [3]University of Warwick, Division of Health Sciences, Warwick Medical School
**Correspondence:** Melina Dritsaki
*Trials* 2017, **18(Suppl 1):**O48

##### Background
Health economists working on clinical trial based economic evaluations are often asked at a preliminary stages of studies, and sometimes before data are available, to propose a plan for the collection and analysis of information on resource use, costs and quality of life. Questions that frequently arise when designing a Health Economics Analysis Plan (HEAP) for a clinical trial include what information should be included as standard within the HEAP, whether and how a proposed plan can be changed, how health economists and statisticians should split responsibility for data preparation and analyses, how missing data should be dealt with, and whether there are circumstances when a HEAP is not needed (for example in a feasibility study).

##### Objective
The aim of this study is to develop agreed guidance for health economists who work on clinical trials on how to pre-plan their analysis in the absence of any data and how to present it in an unambiguous but flexible way.

##### Methods
Guidelines on how to perform economic evaluations based on clinical trials were searched from the literature. HEAPs were also obtained from a few clinical trial units, although there were certain confidentiality issues that had to be surpassed. Section headings (domains) and items were extracted on pre-specified schema we have designed for trial-based HEAPs.

##### Results
We have identified a lack of guidance or any standardised templates on how health economists should present HEAPs for clinical trials. In the current climate where clinical trials units increasingly rely on standard operating procedures (SOPs) that need to be followed, SOPs for economic evaluations should also be considered as good practice. We identified nine main sections that should be presented in a HEAP with a provisional total of 15 items included. We further recommend the analysis be carried out by an identified, appropriately qualified and experienced health economist, who should ensure the integrity of the data during processing. Any deviations from the health economics analysis plan should be described and justified in the final report of the trial.

##### Conclusions
Certain items should be included when designing a HEAP for a clinical trial, but others require debate and consensus. Senior health economists and principal investigators should be involved in writing, reading and authorising a HEAP. Deviations from or changes to the HEAP should be reported and justified when reporting.

### O49

#### Statistical considerations for analyzing multiplex biomarker data from prospective studies

Shun Fu Lee[1], Guillaume Paré[1], Matthew J. McQueen[1], Hyejung Jung[1], Sibylle Hess[2], Hertzel Gerstein[1]
[1]Population Health Research Institute; [2]Sanofi Aventis Deutschland GmbH R&D Division Diabetes
**Correspondence:** Shun Fu Lee
*Trials* 2017, **18(Suppl 1):**O49

##### Background
Biochemical markers of disease have the potential to both enhance our understanding of disease pathophysiology and identify as-yet unaffected people who are most likely to develop the disease. As the cost of measuring large sets of biomarkers has fallen, a growing number of prospective cohort studies and trials are including biomarker measurement in their design. However many of these studies are limited by the absence of a systematic, statistically conservative approach to integrating these measures into the reported findings.

#### Method

After preparing a prespecified analysis plan, the three main statistical considerations when using with multiplex biomarkers to predict outcomes based on survival data are, data preparation, model building and validation. Data with multiplex biomarkers require an extensive data cleaning process including handling the missing information, assessing the distribution for each biomarker, and normalizing and standardizing the biomarkers analyzed. During the model building phase, the approaches used to select the variables, to correct Type I error for multiple testing and to handle proportionality hazards assumptions in survival data are critical to identify the sets of biomarkers to predict the outcome. Lastly, both internal validation (sample splitting vs. bootstrap) and external validation from an independent sample can be used to assess the performance of the model. Biobanked serum samples from 8494 participants with dysglycemia in ORIGIN trial were assayed for 284 biomarkers. The objective of the study was to identify sets of biomarkers that could identify people with dysglycemia at risk for a CV outcome or death when added to well-established clinical predictors of the relevant outcomes. The results of this study will be used for illustration.

**Conclusion**

These statistical considerations provide a reproducible approach for developing clinically useful prognosis model with multiplex biomarkers based on survival data.

Funded by Sanofi; ORIGIN NCT 00069784

### O50

#### Sample size calculations for cluster randomised trials: reconciling variance components in generalised linear mixed models with marginal intracluster correlations

Andrew Forbes[1], James P. Hughes[2], Jessica Kasza[1], Richard Hooper[3]
[1]Monash University; [2]University of Washington; [3]Queen Mary University of London
**Correspondence:** Andrew Forbes
*Trials* 2017, **18(Suppl 1):**O50

**Background**

Sample size calculations for cluster randomised trials (e.g. parallel, stepped wedge, crossover) require specification of one or more intracluster correlations (ICC). With Gaussian outcomes, sample size formulae can be specified equivalently in terms of ICCs directly, or using variance components of a linear mixed model. However, with binary or count outcomes, recommended methods use one or the other approach due to lack of direct relationships between the values of ICCs on the 'raw' (i.e. binary or count) scales and the variance components from generalised linear mixed models (GLMMs). In this presentation we describe an approach for sample size calculations that reconciles the two by employing GLMMs with a logarithmic link function and normally distributed random effects.

**Methods**

Using the properties of the log-normal distribution we specify the relationship between ICCs on the raw data scale and the variance components from GLMMs. Results are exact when no covariates are present and we describe an approximate method in the presence of covariates. We implement these results in sample size calculations for a parallel cluster randomised trial with multiple baseline measurements and multiple followup measurements. We compare power for given sample sizes using specification of ICCs on the 'raw' data scale using the formulae in the approach of Hooper et al. [1] with that from numerical simulations of the corresponding GLMMs in which the variance components are computed from the relationships with ICCs assuming a log link and normally distributed random effects. We also describe a method for checking the compatibility of the specification of ICCs with random effects from GLMMs by solving a set of nonlinear equations.

**Results**

For our simulated examples the empirical power from the GLMM-based simulations using ICCs ranging in value up to 0.10 closely matched that of the formulae of Hooper et al. [1]. For binary outcomes, although large variance components can lead to simulated probabilities above unity, this caused only minimal bias in our examples. For count data one needs to be careful that specification of the marginal overdispersion is compatible with the specified ICCs.

**Conclusion**

GLMMs with log link and normal random effects can be useful for simulating binary and count data with specified marginal ICCs. Our results indicate that the approach of Hooper et al. [1] can be extended to binary and count outcomes under such models.

### O51

#### Explaining randomisation to potential clinical trial participants

Gillian Gyte[1], Sally Crowe[2], Dell Horey[3]
[1]Consumer Editor, Cochrane Pregnancy & Childbirth Group; [2]Crowe Associates; [3]Senior Lecturer, Department of Public Health, La Trobe University
**Correspondence:** Gillian Gyte
*Trials* 2017, **18(Suppl 1):**O51

**Background**

An important challenge in recruiting people to clinical trials is explaining randomised controlled trials, and this is exacerbated in the context of emergency situations. In the pilot phase of a feasibility randomised controlled trial (RCT) involving babies born at less than 32 weeks gestation that aimed to compare delayed and early cord clamping (CORD pilot trial), clinicians found it particularly difficult to explain why mothers in the study could not choose either option and why randomisation was important. We asked healthcare consumers, familiar with RCTs, to help us find ways to explain these issues in time-limited recruitment situations.

**Methods**

An interactive two-stage workshop was devised for consumers active in the Cochrane Collaboration at an annual Cochrane Colloquium. The aim of the workshop was to gain understanding in how to communicate randomisation to the public and to better inform potential participants of clinical trials. In stage 1, workshop members were given magazine adverts promoting purported clinical benefits and asked to design a study that would address the claims made. Subsequent discussion progressed to the concept of randomisation, potential biases that can arise and how these can be minimised with careful study design. With this background, in the second stage of the workshop, the consumers were asked to develop statements describing randomisation to potential study participants of the CORD pilot trial. The consumers, working in groups, were specifically asked for ways to explain to women why trial participants would not be able to choose which study group they could go into, and so would not have a say in when their baby's cord would be clamped. The final statements produced by the different groups were discussed by all workshop members and modifications suggested. This work was later presented at a CORD pilot trial collaborators' meeting.

**Results**

The process identified expressions that consumers disliked and expressions that consumers preferred when discussing clinical trials. The issues raised will be presented along with the statements the consumers produced explaining the CORD RCT. The feedback from collaborators in the CORD pilot trial will also be reported.

#### Discussion
Healthcare consumers can contribute to recruitment to RCTs by developing wording to help explain randomisation to potential trial participants. We used an interactive workshop to generate useful insight about consumer views about clinical trial participation. This approach could be extended to other areas to gather further ideas from healthcare consumers.

### O52
## Maximising trial efficiency in the airways-2 trial using a multi-functional database
Lauren Scott[1], Jodi Taylor[1], David Hutton[1], Barney C. Reeves[1], Jonathan R. Benger[2], Chris A. Rogers[1]
[1]Clinical Trials & Evaluation Unit, University of Bristol; [2]Faculty of Health and Applied Sciences, University of the West of England
**Correspondence:** Lauren Scott
*Trials* 2017, **18(Suppl 1):**O52

#### Background
Airways-2 is a cluster randomised trial comparing two airway management devices for the treatment of out-of-hospital cardiac arrest. Participating paramedics from four UK ambulance trusts are randomised to manage the patient's airway using either an i-gel device or intubation. We are aiming to recruit 1,500 paramedics, screen approximately 60,000 patients and enrol more than 9,000 patients over a two year period. As patient recruitment takes place in an emergency setting and patients who survive to hospital admission could be admitted to one of 100 hospitals, data collection needs to be as efficient and streamlined as possible.
### Methods
We designed a multi-functional database to track patients' progress through the trial, as well as capture the trial data. Data collection has been organised in sections reflecting the different stages of the patient journey. Access to the different sections is dependent on the outcome of the previous section, e.g. it is not possible to access the area of the database that captures hospital admission data if the patient died at the scene of the arrest. Access is also restricted according to role and location, e.g. hospital based researchers cannot access the sections relating to pre-hospital paramedic data collection. User access is via a secure NHS web server. Data quality is maximised by validation checks on data items; the database allows users to save invalid data or leave items blank but such instances generate queries. Users may correct data entry errors or provide responses to queries within the database, resulting in a complete audit trail of any changes. A "traffic light" system helps users to keep track of outstanding queries. Additionally, the database creates reports to facilitate the coordination team in identifying patients who are due 3 or 6 month post cardiac arrest follow-up. Documents for follow-up containing mail merged patient details can be downloaded from the database.
### Results
The Airways-2 database has approximately 300 users undertaking one of five different roles. So far, over 44,000 patients have been screened and nearly 6,000 patients have been enrolled. Data queries, along with regular central monitoring, have enabled successful patient tracking and over 99% data completeness for the primary outcome. User feedback has been positive. The system is intuitive and easy to use.
### Discussion
When running a large complex trial involving a diverse range of staff and researchers working across different health care settings, it is important to consider using IT solutions to optimise efficiency and data quality. Database features developed for the Airways-2 trial are now being implemented in other studies.

#### Funding
The Airways-2 trial is funded by the National Institute for Health Research (NIHR) Health Technology Assessment (project number 12/167/102). The views and opinions expressed are those of the authors and do not necessarily reflect those of the NIHR, NHS or the Department of Health.

### O53
## Priorities for methodological research on patient and public involvement in clinical trials: a modified Delphi process
Kerry Woolfall[1], Anna Kearney[2], Heather Bagley[2], Carrol Gamble[2], Jim T. Elliot[3], Helen Bulbeck[3], Stephen Thomas[3], Bridget Young[4], Simon Denegri[5], Delia Muir[6], Natalie A. Simon[7], Joanna C. Crocker[8], Claire Planner[9], Mike Clarke[10]
[1]The University of Liverpool; [2]Clinical Trials Research Centre (CTRC), North West Hub for Trials Methodology Research, University of Liverpool; [3]Patient and Public Involvement partner; [4]Department of Psychological Sciences, North West Hub for Trials Methodology Research, University of Liverpool; [5]University College London and National Institute for Health Research (NIHR), INVOLVE; [6]Leeds Institute of Clinical Trials Research (LICTR), University of Leeds; [7]Health Care Research Wales, Public Involvement and Engagement, Castlebridge 4; [8]NIHR Biomedical Research Centre and Nuffield Department of Primary Care Health Sciences, University of Oxford; [9]Centre for Primary Care, University of Manchester; [10]Centre for Public Health, Queen's University of Belfast
**Correspondence:** Kerry Woolfall
*Trials* 2017, **18(Suppl 1):**O53

#### Background
Despite increasing international interest, there is a lack of evidence about the most efficient, effective and acceptable ways to implement patient and public involvement (PPI) in clinical trials. This study aimed to identify the priorities of UK PPI stakeholders for methodological research to help resolve uncertainties about PPI in clinical trials.
### Methods
A modified Delphi process including a two round online survey and a stakeholder consensus meeting. We used snowball sampling to identify and invite PPI stakeholders to take part in the online Delphi. Stakeholders included PPI contributors, lay and non-lay reviewers from funding panels and Research Ethics Committees, PPI coordinators, PPI Planners (e.g. Chief Investigators and Trial managers), PPI advisors (e.g. Research Design Service members) and PPI researchers.
### Results
In total, 237 stakeholders registered of whom 219 (92%) completed the first round. 187 of 219 (85%) completed the second; 25 stakeholders attended the consensus meeting. Delphi findings were presented and considered at the consensus meeting. 16 of the 42 topics were rated of critical importance by over 70% of stakeholders at the meeting. 96% of stakeholders rated the top three topics as equally important. These were: research on ways to develop strong and productive working relationships between researchers and PPI contributors; exploring PPI practices in selecting trial outcomes of importance to patients; and a systematic review of PPI activity to improve the accessibility and usefulness of trial information materials for clinical trial participants.
### Conclusions
The prioritised methodological research topics identified by the Delphi process indicate important areas of uncertainty about PPI in trials. Addressing these uncertainties will be critical to enhancing PPI. Our findings should be used by those involved in the planning and funding of PPI in clinical trials to help focus research efforts and minimise waste.

### O54
## Audio-recording recruitment consultations - an exploratory study in two RCTs to investigate the impact on randomisation rates
Chris Rogers[1], Sangeetha Paramasivan[2], Daisy Elliott[2], Paul Whybrow[2], Sofia Kanavou[1], Rosie A. Harris[1], Graziella Mazza[1], Tim Brush[1], Jane M. Blazeby[2], Eric Lim[3]
[1]Clinical Trials and Evaluation Unit, University of Bristol; [2]School of Social and Community Medicine, University of Bristol; [3]The Royal Brompton Hospital
**Correspondence:** Chris Rogers
*Trials* 2017, **18(Suppl 1):**O54

#### Background
Recruitment to randomised controlled trials can be challenging, particularly when the burden of participation is high (e.g. long and/or intensive follow-up), the interventions under evaluation are very different (e.g. surgery versus best medical management) and there are strong preferences amongst clinicians and/or potential participants. To help overcome these challenges, a quintet recruitment intervention (QRI) was developed by Donovan and colleagues in the context of the ProtecT trial. The QRI is a complex intervention with several components. These include detailed assessment of screening logs, in-depth interviews, audio-recording of recruitment consultations, and expert review and analysis of the consultations to allow group and individual feedback to recruitment teams on how to optimise informed consent. The impact of the individual elements of the QRI have not been previously explored. The aim of this study is to examine the association between trial recruitment and audio-recording of consultations.

**Methods**

Data from two on-going "difficult-to-recruit to" multi-centre RCTs in surgery with an integrated QRI were used for this study; one comparing the effectiveness and cost-effectiveness of three types of bariatric surgery for severe and complex obesity (By-Band-Sleeve) and the other comparing open versus video-assisted thoracotomy (key hole) surgery for early stage lung cancer (Violet). In both trials, recruiters and eligible participants were invited to have their recruitment consultation audio-recorded. We examined the rate of consent amongst patients who did and did not agree to audio recording.

**Results**

Over the two trials, approximately two-thirds of eligible patients agreed to their consultation being audio-recorded (By-Band-Sleeve 1209/1806, 67%; Violet 149/185, 80%), with some variation in audio-consent rates across centres. In both studies the proportion of patients agreeing to randomisation is higher amongst the group who consented to audio-recording and the pattern is consistent across the two studies (By-Band-Sleeve 39% vs 9%, Violet 76% vs 16%). The probability of consenting to randomisation is four-times higher (risk ratio 4.45, 95% CI 3.2-6.1) when the consultation is recorded. The overall randomisation rate is 29% in By-Band-Sleeve and 50% in Violet. Withdrawal rates following consent are low in both trials (By-Band-Sleeve 4%, Violet 6%).

We will describe the variation in audio-consent rates by centre and recruiting surgeon and the impact of QRI feedback on trial recruitment rates.

**Discussion**

This is the first study to examine the association between consent for audio-recording and trial participation. Whilst we have demonstrated an association, the reasons for this are unclear and require further investigation. They may be patient-related, recruiter-related, or both. For example, a patient who consents to audio-recording may be naturally more predisposed to research, or they may feel they should agree to randomisation because the consultation could be listened to (although we have no evidence of coercion). Similarly, recruiters who consent to audio recording may be more comfortable with and better at conducting recruitment consultations. They may also "try harder" to recruit the patient when the consultation is being audio-recorded. It is important to explore and better understand the factors that might underlie this association to further support trial recruitment.

### O55

#### Assessing treatment effect moderation in trials of psychological interventions: a case for individual participant data meta-analysis of pooled trials

Sabine Landau[1], Victoria Harris[1], Patty Leijten[2], Joanna Mann[2], Eva-Maria Bonin[3], Jennifer Beecham[3], Judy Hutchings[4], Stephen Scott[1], Frances Gardner[2]
[1]King's College London; [2]University of Oxford; [3]London School of Economics and Political Science; [4]Bangor University
**Correspondence:** Sabine Landau
*Trials* 2017, **18(Suppl 1):**O55

**Background**

The investigation of treatment effect moderation is a common research objective in psychological studies. The benefit or harm of an intervention might vary within a study according to patient characteristics or between studies due to contextual factors such as the composition of the study population or the study setting. Statistically the investigation of treatment effect moderation amounts to an assessment of interactions between putative moderators and treatment groups. Interaction tests tend to be underpowered when based on individual participant data (IPD) from a single study. Pooling multiple trials aimed at evaluating the same intervention offers a way forward here.

Aggregate data meta-analytic techniques are routinely used to combine results from multiple studies. Specifically meta-regression obtains treatment effect estimates (and associated precision estimates) from different trials and then investigates the association between the trial-level treatment effect estimates and contextual factors using a weighted regression approach. Importantly, meta-regression is only able to assess trial-level variability in treatment effects. In addition, the number of replicate trials tends to be small and hence limits the ability for making adjustments in the analysis.

In order to investigate treatment-effect moderation by individual participant characteristics it is necessary to retrieve and analyse the IPD. Two strategies for IPD meta-analysis exist: A two-stage approach analyses the IPD for each trial separately, generates appropriate trial-level summaries and then combined these in a second step using aggregate data meta-analysis. In contrast multilevel modelling of the pooled IPD from all trials assesses moderation in a single analysis. Importantly, the latter approach can assess both, moderation by variables that vary between trials and by variables that vary within trials.

**Methods**

We pooled IPD from 13 European trials of the Incredible Years (IY) parenting intervention aimed at improving disruptive child behaviour (Eyberg Child Behavior Inventory) in children with conduct problems. We considered a number of pre-specified putative individual-level moderators of the IY intervention effect. We assessed moderation effects using both, meta-regression based on published effect sizes, as well multilevel modelling of the pooled IPD.

**Results**

IPD meta-analysis performed by multilevel modelling was able to account for trial design features such as variation in randomisation ratios, stratification and clustering; and enabled principled analyses to be carried out in the presence of missing values. It also allowed us to separate trial-level from individual-level moderation effects and to make adjustments. The comparison of the modelling results with the findings from a standard meta-regression showed that IPD meta-analysis can avoid aggregation bias (also known as the ecological fallacy), and can produce more powerful inferences when moderation effects do not differ between the trial level and the individual level.

**Conclusion**

We encourage the use of the one-stage multilevel modelling approach to IPD meta-analysis. A two-stage IPD meta-analysis does not require explicit assumptions regarding the variability of effects across trials and tools are increasingly becoming available. While there is a lot to be said for simplicity, we prefer the more complex one-stage approach as it offers the flexibility to address all the moderation questions with optimal efficiency.

### O56

#### Working with a vulnerable population on a sensitive topic to better understand neonatal intensive care trials: development of a suite of strategies to meet challenges for qualitative data collection in the bracelet study

Claire Snowden[1], Peiter Brocklehurst[2], Robert Tasker[3], Martin Ward-Platt[4], Diana Elbourne[5]
[1]London School of Hygiene and Tropical Medicine MRC London Hub for Trials Methodology Research, London, UK; [2]Birmingham Clinical Trials Unit, Institute of Applied Health Research, University of Birmingham; [3]Department of Neurology, and Anaesthesia (Pediatrics), Harvard Medical School; [4]Newcastle Neonatal Service, Royal Victoria Infirmary; [5]London School of Hygiene and Tropical Medicine
**Correspondence:** Claire Snowden
*Trials* 2017, **18(Suppl 1):**O56

**Background**
There is growing understanding of the value of qualitative research to elucidate the conduct and impact of randomised trials, and to guide future trial conduct. Some of the most challenging trials assess interventions aimed at improving care for seriously ill patients and so can take place in the context of major life events. For qualitative research to offer insights into such trial processes and settings, researchers must engage with potentially vulnerable populations to ask often difficult questions. As researchers, we must take responsibility for the proper care and treatment of those who take part in our research and engage with those questions, and so need appropriate research tools and support systems to work with challenging trials.
**Methods**
The BRACELET Study involved bereaved parents whose babies died subsequent to enrolment in five neonatal intensive care trials. A suite of strategies was developed to: maximise collection of good quality data, ensure data collection processes were sensitive and supportive to contributors, and to facilitate involvement of hard-to-reach bereaved parents. Data collection and support strategies were developed in response to the sensitivities involved. BRACELET involved face to face interviews with bereaved parents. Recognising that some parents would not wish to be interviewed, two alternative participation routes were developed, an online comments option and short questionnaires. A detailed Code of Practice was developed to guide the conduct of lone and joint interviews which took into account challenges of discussing bereavement and trials with potentially vulnerable individuals. The Code was organised around five key principles: the interviewees should be given control of the physical environment; the interviewee should be given control over the progress of the interview; the distinction between counselling and research should be clear; all interviewees will be treated with courtesy and respect; and the interviewee should have access to information and support after the interview. Each principle had multiple dimensions which were explored and operationalised in the Code. To meet the final principle, dedicated support systems were developed in collaboration with local and national support providers to ensure participants could access help and information post-interview should it be needed. A post-interview questionnaire was used to monitor reactions to the interviews.
**Results**
Fifty-one bereaved parents were interviewed, and the online comments option and short questionnaires yielded valuable extra data from 8 parents which offered new insights and confirmed themes arising from interviews. Thirty-nine post-interview questionnaires confirmed that, although the process was deeply emotional for many of the parents, it was perceived as valuable both for themselves and for parents in the future.
**Conclusions**
The BRACELET study enabled parents to elucidate their bereavement experiences in a trial context, and to offer guidance on good conduct for future trials where mortality is anticipated. The strategies developed for the BRACELET study were crucial factors in its success and ethical conduct, and may be adapted by others exploring sensitive issues for trials with vulnerable populations. While our strategies included elements tailored to our specific challenges, they are readily adaptable for other trial-related sensitive research areas.

### O57
### The patient journey planner: an integrated approach to trials of targeted therapies with application to cancer and haematological malignancies
Robert Hills[1], Nigel Russell[2], Kerry Hood[1], Girlish Patel[3], Richard Adams[1]
[1]Cardiff University; [2]University of Nottingham; [3]University Hospital of Wales
**Correspondence:** Robert Hills
*Trials* 2017, **18(Suppl 1):**O57

**Background**
Recent advances in the understanding of disease at a genetic level have demonstrated that many conditions are not a single homogeneous entity. However, while these insights provide valuable evidence on disease mechanisms, the fragmentation of conditions leads to challenges in trial design. Instead of a classical Phase 3 mega-trial, with wide eligibility, the evaluation of targeted therapies requires the identification of subgroups of patients, some of which might be very small. Agents may be at different stages of development; and further still, clonal evolution can mean that agents are not equally efficacious at different stages of disease.
**Aims**
To design trials to accelerate the evaluation of new targeted therapies which may be at different stages of development or relevant to different stages within the patient pathway.
**Results**
Patient eligibility for different treatments may change throughout their journey, mirroring the choices that are made in clinical care. At any one time, umbrella trials provide an infrastructure for screening patients and directing them to an appropriate treatment, and these can be combined with a platform trial which evaluates various different treatment options for each subgroup. However, numbers soon become vanishingly small and even large national collaborative groups can struggle to find sufficient patients. We present therefore an extension of these approaches called the Patient Journey Planner. The combined design allows different national collaborative groups to maintain their own trials infrastructure while contributing to multiple evaluations of targeted therapies. Evaluations will typically take place at different stages through a patient's "journey" with a condition; at each stage (e.g. induction, maintenance, relapse), a number of different options will be available based upon genetic characteristics or response to prior therapies. The design of the evaluation of each of these is appropriate to the stage of development of the therapy, and the aspirations for the treatment or the patient group. Such an approach is proposed and illustrated for the treatment of Acute Myeloid Leukaemia, and also in solid tumours such a skin cancer, where there is a similar degree of heterogeneity, and the requirement to evaluate novel targeted therapies at different disease stages. The approach also allows the integration of supportive care and prevention studies.
**Conclusion**
Relying on appropriate exchange of data the Patient Journey Planner allows the evaluation of therapy, using modern trial designs, across multiple collaborative groups, not only speeding up drug development but enabling the assessment of treatments in groups which otherwise would be too small for formal trials run by a single centre or collaborative group.

### O58
### The importance of randomisation in evaluation of treatments for acute myeloid leukaemia: lessons from the UK NCRI aml16 and li-1 trials
Ian Thomas[1], Robert K. Hills[1], Nigel Russell[2], Mike Dennis[3]
[1]Cardiff University; [2]University of Nottingham; [3]Christie Hospital NHS Trust
**Correspondence:** Ian Thomas
*Trials* 2017, **18(Suppl 1):**O58

**Background**
Initial evaluations of treatment activity are typically performed in Phase 2 clinical trials. Historically, such trials used either one or two-stage single-arm designs, such as the Gehan, A'Hern, or Simon approaches. Such designs rely on identifying the level of outcome (e.g. response rate) which merits further investigation, and the design of such a trial would specify a minimum number of responses to proceed to further evaluation. The choice of threshold depends on the condition being studied, which is often informed by historical data. By contrast, randomised Phase 2 trials do not specify an absolute threshold of response, but rather a level of improvement that is required to be seen.
**Methods**
We present here the results from the control arm of the UK National Cancer Research Institute Pick-A-Winner programme for older patients with acute Myeloid Leukaemia not suitable for intensive treatment. The programme uses a seamless Phase2/Phase 3 design to evaluate treatments, with an early assessment for futility. New treatment options can be brought in at any time to replace options which are closed, and evaluation only takes place between novel therapy and control among contemporaneously randomised patients. Therapies which pass the initial futility hurdles proceed seamlessly to Phase 3 evaluation within the programme. The programme has been running for over 10 years, and over 1500 patients have entered the randomised comparisons for 11 different novel therapies.

**Results**

Outcomes of the control arm of low-dose ara-C show a wide variation in complete remission rates from comparison to comparison, ranging from less than 10% in some cases to more than 30%. Response rates can fluctuate by comparator drug based upon the eligibility criteria for the treatments; but even when eligibility criteria remain constant, wide variation is seen in remission rates, which may be explained by differing referral patterns and the perceived attractiveness or toxicity of the novel therapies. The wide variation in results shows the issues associated with using a single arm phase 2 trial in a heterogeneous condition: setting an 'average' target of 20% response rate (similar to that seen in the original trial of low-dose ara-C), some treatments which showed a remission benefit would have failed to pass the hurdle, while others closed for futility would have proceeded to further evaluation.

**Conclusion**

Our results demonstrate the need for randomisation in phase 2 trials in this group of patients, and indeed in other heterogeneous conditions, to ensure interpretability of results.

### O59

#### If a tree falls in a forest: abstract view statistics as a measure of research impact

Andrew Embleton[1], Deborah Ashby[2], Ella Flemyng[3], Peter Langhorne[4], William J. Meurer[5], Annabelle South[1], Matthew Sydes[1]
[1]MRC Clinical Trials Unit at UCL, Institute of Clinical Trials and Methodology, UCL, London; MRC London Hub for Trials Methodology Research, London; [2]Imperial Clinical Trials Unit, Imperial College London; [3]BioMed Central; [4]University of Glasgow; [5]University of Michigan
**Correspondence:** Andrew Embleton
*Trials* 2017, **18(Suppl 1):**O59

**Background**

Co-publication of papers to coincide with conferences is not yet standard for clinical trials, although improving e.g. a selection of plenary abstracts at European Society of Medical Oncology 2016 were co-published in high-impact medical journals. For clinical trial methodology, publication usually lags presentation (if achieved); the conference abstract is the key written information available. The International Clinical Trials Methodology Conference (ICTMC) 2015 presented 3 invited talks with 94 oral and 238 poster presentations. We are interested in their online attention.

**Methods**

Abstracts were available online in a supplement to BioMed Central's Trials from conference Day 1. Initially motivated in tracking interest ICON6 (#P184), we used an algorithm applied to the journal website to collect basic article view metrics from Trials giving a snapshot of cumulative online views. The impact on abstract views of presentation type, submission category and title form can be explored.

**Results**

The results describing the views over time for every accepted abstract from ICTMC 2015, along with a summary of wider themes, are available at http://andyembleton.me/ICTMC/ The abstract addressing "Longitudinal mediation in the PACE trial" was consistently the most popular abstract from Day 4 onwards (i.e. post-conference). This abstract gained 527 views between Days 4 and 8, the largest uptick in views, perhaps due to its posting on a popular Twitter account and discussion on a patient forum. There was little overall difference between those oral and poster abstracts initially, but this manifested into an expected hierarchy over time. Though focusing specifically on the top-viewed 5% abstract (n = 16): at Day 3, 12 of these were poster presentations, including all the top 10; by the latest data capture at Day 55, the top four abstracts were oral presentations, although posters still occupied 11 out of the 16. The most viewed theme from oral abstracts was "Adapting Trials Based on Internal Data" (median 124 views by Day 55), with Trial and Project Management least popular (median 68 views by Day 55). The poster themes varied greatly in the number of comprising abstracts but the "Use of Innovative Methods in Active Trials" garnered the most average views (median 91), and "Involving Research Partners" least (median 60).

**Potential Results**

We propose presenting both short- and long-term findings from ICTMC 2015 plus initial, short-term results from ICTMC/SCT 2017 conference, possibly as a penultimate talk in the final session. We will provide an on-line searchable resource for this conference using at least daily views of abstract, during and beyond the conference.

**Potential Impact**

We can draw together conclusions from the previous conference, compare and contrast the abstract views from the two conferences and highlight any changes or consistent themes. Also, just as crucially, we will be tracking abstracts views over time beyond the end of the conference and ensuring access is available to both authors and external researchers, both on the website and in dataset format. This will ensure that interested parties from the 2017 conference can clearly gauge the impact of their own work and any promotional efforts they make.

### O60

#### A way to assess the impact of an omitted study on the overall meta-analysis estimate

Joana Vasconcelos[1], Rebecca M. Turner[2], Toby A. Prevost[1]
[1]Imperial College London; [2]Medical Research Council Biostatistics Unit
**Correspondence:** Joana Vasconcelos
*Trials* 2017, **18(Suppl 1):**O60

**Background**

Good reporting of research findings in primary papers is crucial to allow meta-analyses to include all available studies on the topic of interest. However, key information in trial results is still commonly missing (Altman, 2015), such as standard deviations (SD) of continuous outcomes. Specific recommendations in the Cochrane Handbook and PRISMA statement on handling missing data include retrieving all connected information and performing sensitivity analysis to challenge the missing data assumptions. These are not frequently followed, nor is there a simple method to evaluate the impact of omitting a study from a meta-analysis. We propose a statistic that would help reviewers to assess the influence that a study without an SD estimate may have on the pooled effect meta-analysis and calculate this statistic across multiple Cochrane meta-analyses. We also report on whether reviewers could have deduced or approximated the missing SD in primary papers or imputed the SDs by those reported in other studies in the same meta-analysis.

**Methods**

We used the whole Cochrane Database of Systematic Reviews issued in January 2008 (Davey et al., 2011) to identify studies omitted with a missing SD from 6672 continuous-outcome meta-analyses. A subsample of these, published since 2006, was selected and enabled hand-searching and extracting to assess whether SDs could have been deduced or approximated and to calculate the influence statistic. We report on fixed effect meta-analyses.

**Results**

Amongst the 6672 sampled meta-analyses, 396 (6%) were affected by omitted studies. The hand-searched sub-sample comprised 52 studies, from 28 meta-analyses within 18 Cochrane reviews, including 33 subgroup meta-analyses. In 5 (28%) reviews, reviewers did not attempt to contact original publication authors to retrieve missing information and only one meta-analysis reported sensitivity analysis to assess the impact of imputing SDs. For 12 (36%) subgroup meta-analyses, the SDs could have been externally imputed from other studies' SDs. In 19 (37%) of omitted studies, the SDs could have been internally retrieved through either deducing (8%) or approximating (29%) from available information. Developed from the inverse-variance weighting formula for fixed effect models, we proposed an 'influence statistic' that tells us whether, and by how much, a study that does not report the precision of an effect could influence the overall meta-analysis estimate, defined as the Z-statistic ratio of the distance between the omitted study effect from the pooled effect and the standard error of this distance. This statistic was calculated and its distribution displayed across 16 studies that were omitted from 12 subgroup meta-analyses. Nineteen percent of these had a high influence ($Z > 2$) on the subgroup meta-analysis pooled effect.

**Conclusions**

Incomplete reporting of variance estimates in continuous outcomes by study authors still remains a reality. We proposed a tool that would help authors to determine the influence of a missing trial in the pooled meta-analysis estimate. Current work includes development of the influence statistic to include random effects models and to the influence of multiple omissions per meta-analysis. References Altman, 2015. doi:10.1186/s13063-015-0575-7 Davey et al., 2011. doi:10.1186/1471-2288-11-160

### O61

#### Sensitivity analysis for missing data using the delta method: the impact of incorporating a prior distribution on delta

Suzie Cro[1], Michael Kenward[2], James Carpenter[3]
[1]Imperial College London; [2]The London School of Hygiene and Tropical Medicine; [3]MRC Clinical Trials Unit at UCL, Institute of Clinical Trials and Methodology, UCL
**Correspondence:** Suzie Cro
*Trials* 2017, **18(Suppl 1):**O61

Unfortunately, there will often be uncontrollable developments in clinical trials which result in the occurrence of missed patient visits or unrecoverable outcome measures. However primary analysis is approached, we must make an untestable assumption about the distribution of the unobserved data. It is then important to perform sensitivity analysis to explore the robustness of the conclusions to alternative plausible assumptions about the missing data.

In primary analysis it is most often reasonable to assume that conditional on any covariates included in the analysis, data is Missing at Random (MAR). Or equivalently, that the conditional distributions of the outcome data for observed and unobserved patients are equal. For sensitivity analysis it may then be of interest to explore the impact of unobserved patients having a poorer/better response than those observed. The so called `Delta- method' is an accessible pattern-mixture approach which enables such investigation using multiple imputation. Imputations are conducted under MAR, then edited by a postulated amount, Delta, to reflect a worse or better response. The primary analysis model is retained in the sensitivity analysis to asses purely the impact of alternative behaviour among the unobserved on the primary outcome.

In the sensitivity analysis we do not want to lose any of the valuable information collected in the trial. A natural principle, which we refer to as the information anchoring principle, is to preserve the information loss due to missing data in the primary analysis in the sensitivity analysis. However, it is also possible to have much less information in the sensitivity analysis than in the primary analysis. Moreover, a naïve sensitivity analysis may insert more information than we would have had if we actually observed the data. Conclusions from the primary analysis can then be overturned.

We show how an information anchored variance estimate is obtained for the treatment estimator when a fixed Delta adjustment is used. However, when uncertainty surrounds the value of Delta and we incorporate a prior distribution on Delta in imputation, there will be less information in sensitivity analysis than primary analysis. Results from a peer review trial demonstrate the consequences surrounding the choice of Delta in a real life setting and why careful elicitation is required.

### O62

#### A web-based system for the management and storage of metadata for multi-center clinical trials

Kevin Wilson, Michale Ham, Brenda Hair, Eugene Turner
RTI International
**Correspondence:** Kevin Wilson
*Trials* 2017, **18(Suppl 1):**O62

**Background**

The use of common data elements (CDEs) to standardize data collection in clinical trials is widely viewed as a mechanism to improve efficiency in trial design and conduct and to improve the quality of research. CDEs have the potential to enhance the comparability of data from different clinical trials, reduce development time for clinical trial protocols, and significantly reduce the time needed to develop electronic data capture (EDC) systems. There are several challenges to leveraging CDEs effectively on large clinical trial networks. Such networks typically comprise several collaborating institutions that conduct multiple trials, typically over a 5-year funding cycle. Each protocol is developed by a group of investigators in collaboration with staff from a data coordinating center. Due to the nature of this work flow with individual trials developed over a period, it is common for the definitions of individual data elements to evolve over different clinical trials.

**Methods**

The system design and methods encompassed several components, including: (1) an automated tool to import study metadata from previously implemented studies into a centralized repository; (2) a tool to standardize key study metadata elements to support curation and output to multiple EDC systems; (3) an efficient search engine to identify and group elements into new case report forms; and (4) the ability to output metadata to define a new study in the EDC system along with PDF versions of the case report forms. Study metadata for an EDC system is typically defined in a structured CSV or XML-based format and includes definitions of individual data elements, case report form (CRF) structures, and the study event structure. These metadata include the name of the variable, question text, data type, allowable values if categorical or range if numeric, and whether the variable is required. In addition, data elements are grouped into electronic case report forms, where related variables are collected together (e.g., demographics, medications, etc.). These elements were automatically extracted using a connector program and formatted for importing into a central repository.

**Results**

Data elements were imported from the EDC systems of two research networks. A total of 8,620 unique data elements, derived from 291 unique CRFs, were imported from 4 clinical research trials. Initial searches of the repository revealed interesting patterns in the data elements and clearly showed inconsistencies between similar variables collected across the trials. Examples included the same variable collected in multiple studies with different variable names, different response options for the same variable in different studies, and the same variable names used for different variables. After curation, the system was used to generate formatted CRFs and the associated EDC system.

**Conclusion**

Our previous efforts to standardize data collection in clinical trials focused on standardization at the CRF level but this approach was often unsuccessful. In contrast, standardization at the data element level, allows flexibility across studies while collecting variables in a standardized way. The collection of standardized metadata also enables the development of EDC systems for new trials extremely quickly.

### O63

#### A systematic review of measures of informed consent for randomised controlled trials

Katie Gillies, Alex Duthie, Marion Campbell
University of Aberdeen
**Correspondence:** Katie Gillies
*Trials* 2017, **18(Suppl 1):**O63

#### Background
The subjective assessment of informed consent for clinical trials, and the potential difficulties associated with it, has led several studies to develop objective measures of informed consent for clinical trials. These objective measures of informed consent are often specific to a particular population or clinical condition and largely focus on understanding of (some or all of) the key elements of informed consent, namely: capacity, disclosure, understanding, voluntariness and permission. Many of the developed tools are study-specific, but some validated measures exist. Whether these objective tools conceptualize and measure informed consent in the same way is not known. As such, it is not clear whether meta-analyzing data from studies reporting different tools is worthwhile. The aim of this systematic review was to critically appraise the evidence on the conceptualization and item content of validated questionnaire based measures of informed consent for randomised controlled trials.
### Methods
A systematic search of the literature was conducted to identify relevant articles that described the development, and/or validation, of measures of informed consent for RCTS. General data extraction categories were split into those relating to the context of the included study and those relating to items included in the instrument. Data was synthesized by coding of the items identified into domains and sub-domains which were determined by nomenclature defined in included studies. Both for descriptions of included studies and of the instruments reported in those studies, descriptive statistics were used to describe general information and instrument detail. A narrative synthesis of the instruments and their inter-related domains and sub domains was conducted to identify areas of both convergence and divergence.
### Results
The search identified 6669 citations. After screening titles and abstracts, 16 complied with our pre-specified inclusion criteria. The included studies report 16 separate instruments whose aim is to measure an aspect of informed consent for RCTs. Four of the included instruments report development of a tool to assess competence of research subjects to consent to participation in RCTs i.e. was set amongst participants who may have diminished decisional capacity (e.g. early stage Alzheimer's, schizophrenia, etc.). Of the 16 instruments, 3 explicitly reported a theoretical or conceptual framework underpinning their development, a further 3 implicitly refer to the "conceptual dimensions of informed consent" or "principles of research ethics" as informing their development and 10 reported no guiding theoretical framework. Linked to this, some instruments were explicit with regard to which constructs they were measuring while others were more vague. Finally, only 3 of the 16 studies reported patient or public involvement in the development of the tool. Findings from the narrative synthesis of individual constructs will also be presented.
### Discussion
This presentation will discuss the key issues relevant for this work specifically relating to the issues surrounding the heterogeneity of existing measures of informed consent to RCTs. The results from the narrative synthesis will be discussed with explicit considerations regarding the conceptualization of informed consent and inclusion of constructs and items that matter to potential trial participants.

### O64
### Economic evaluation alongside stepped wedge trials: a systematic review and a proposal of analysis
Gian Luca Di Tanna, Vladislav Berdunov, Richard Hooper
Pragmatic Clinical Trials Unit, Queen Mary University of London
**Correspondence:** Gian Luca Di Tanna
*Trials* 2017, **18(Suppl 1):**O64

#### Background
The stepped wedge trial (SWT) design has gained popularity in recent years and may have practical and methodological advantages over the classic parallel group design especially in the context of health services research. The use of statistical methods in economic evaluations conducted alongside SWTs have hitherto not been systematically explored. Objectives 1. To systematically review economic evaluations alongside SWTs to examine statistical methods used to adjust cost and outcome variables for clustering and time effects inherent to the stepped wedge design. 2. To explore theoretical models that combine existing approaches to the joint (bivariate) modelling of cost and effectiveness in cluster randomised trials with multi-level models that have been proposed for univariate outcomes in stepped wedge trials.
### Methods
An ad-hoc search strategy of Medline/PubMed, DARE, NHS-EED, HTA and the Cochrane Library was used to identify economic evaluations alongside SWTs or related methodological studies. Abstracts were independently screened by two investigators using the following inclusion criteria: (i) use of the SWT design and (ii) economic evaluation as part of the research question(s) in the study. Modelling the cost-effectiveness alongside SWTs We propose in which the multi-level model for effectiveness (on a continuous scale) mirrors the multi-level model for cost, with corresponding random effects and error terms in the two models assumed to follow bivariate normal distributions.
### Results
A total of 100 abstracts identified by the electronic search were screened. A total of 18 papers were included in our review: 15 were study protocols, 1 gave insights on the methods for economic evaluation alongside SWTs and 2 papers were trial results. Methods used (or intended to be used) in studies can be grouped into three main categories: mixed/multilevel models, generalized estimating equations and (generic) bootstrap, but there is an underlying vagueness in the models (to be) used given also the limited guidance available. We present the results of a set of simulations exploring the performance of the proposed method under different assumptions related to the number of participants per cluster, number of clusters in each trial arm and values of the ICC.
### Conclusions
SWT designs are gaining popularity, but statistical methods for economic evaluation conducted alongside SWTs have not been sufficiently explored and used. The use of appropriate methods that account for time, clustering, and correlation between costs and outcomes is an important part of SWT health economics analysis. Thus study aims to demonstrate how such models perform and can be implemented in the context of economic evaluation alongside SWTs.

### O65
### What are the methodological challenges in the design and conduct of orthopaedic randomised controlled trials comparing surgery and non-operative interventions? A systematic review
Loretta Davies, Jonathan Cook, Andrew Price, David Beard
University of Oxford
**Correspondence:** Loretta Davies
*Trials* 2017, **18(Suppl 1):**O65

#### Background
Surgical randomised controlled trials (RCTs) often present additional challenges in design, conduct and analysis to those evaluating pharmacological treatments. This is especially evident when the interventions being evaluated are quite different, for example, the comparison of surgery with a non-operative intervention such as physiotherapy. Specific methodological issues have been reported such as the impact of treatment preferences of both patients and participating clinicians on recruitment, difficulties with blinding and standardising interventions. The aim of this study was to systematically review the methodological challenges and limitations in musculoskeletal RCTs which compare a surgical and non-operative interventions (e.g. drug treatment or physiotherapy).
### Methods
A search of the MEDLINE and CENTRAL databases between 2010 and 2015 was conducted. Randomised orthopaedic clinical trials which had a surgical and non-operative comparison such as physiotherapy were included. Data were extracted and summarised on the study characteristics such as target and actual recruitment, adherence to randomisation allocation, and in addition reported challenges to successfully conducting the trial amongst other details. A quality assessment was also conducted using the Cochrane Risk of Bias tool.

**Results**

In total, 6484 records were identified and 54 trials were included in the review. The majority of included studies were of fracture management (28, 52%). Twenty three (43%) were multi centre studies, with a sample size of 85 (median, IQR [50,150]). The main intervention comparisons of the included studies were of surgery and physiotherapy interventions (44, 81%). Methodological problems identified included a high proportion of participant crossover from their randomised intervention and difficulties reaching target sample size. Challenges reported by the authors included the impact of both patient and clinician preference for particular treatment on the number of potentially eligible patients recruited. In addition, various practical challenges were reported related to the nature of the interventions evaluated and health care systems in which the research was being conducted. This included difficulties implementing blinding of patients, care providers, and outcome assessors and post-randomisation delays in receiving an intervention.

**Conclusion**

This review demonstrated that trials which compare a surgical and non-surgical interventions have been successfully conducted for a number of conditions, however, several methodological issues were identified that have the potential to impact on the design, conduct and analysis of these trials. If not adequately addressed, these issues may introduce significant bias and threaten the validity of the trial results. The planning and design of future studies should take into account and consider the specific challenges associated with the evaluation of surgical and non-surgical interventions.

### O66

#### Gatekeeping amongst allied health professionals: recruitment in stem cell trial of recovery enhancement-3 (STEMS-3) feasibility study

Claire Diver[1], Rebecca O'Connor[2], Nikola Sprigg[3], Louise Conell[4], Marion Walker[5], Philip Bath[3]
[1]Associate Professor; [2]Nottingham University Buisiness School, University of Nottingham; [3]Stroke Trials Unit, University of Nottingham; [4]Allied Health Research Unit, University of Central Lancashire; [5]Division of Rehabilitation and Ageing, University of Nottingham
**Correspondence:** Claire Diver
*Trials* 2017, **18(Suppl 1):**O66

**Background**

Randomised controlled trials (RCTs) of rehabilitation in the chronic stroke population are complex and challenging. Patient-therapist relationships within a rehabilitation setting can be strong and powerful. This is associated with treatment satisfaction in the traditional healthcare setting but it is unclear if this could impact on recruitment to clinical trials. The aims of clinical practice and clinical research can be different: clinical practice is focused on individual care and research on population benefit. Allied Health Professionals (AHPs) are known to be reluctant to recommend oncology and palliative care patients to clinical trials through well intentioned assumptions that it might not be in the best interests of the individual patient. This attitude has not been explored in chronic stroke rehabilitation trials. STEMS3 (ISRCTN16714730) was a feasibility, 2x2 factorial randomised controlled trial of granulocyte colony stimulating factor (G-CSF) vs placebo, and community stroke physiotherapy vs none, in stroke survivors discharged from rehabilitation services. ISRCTN1671470. The aim of this study was to explore recruitment by AHPs to STEMS-3.

**Method**

Qualitative interview study underpinned by theoretical perspectives of pragmatism and critical reflection. We used a purposive sampling strategy to identify AHP's working in a community stroke team (CST) involved in participant identification. Semi-structured interviews were conducted, recorded and transcribed verbatim. Thematic analysis using an iterative and emergent approach was adopted.

**Results**

Six NHS staff were recruited: 3 physiotherapists, 1 Occupational therapist, 1 speech and language therapist and 1 specialist mental health nurse. 3 main themes were identified: information, eligibility and intervention. AHPs felt informed about the trial and professional responsibility to recruit to clinical trials including STEMS-3; they considered the regular presence of a member of the trial team a valuable resource. Despite clear and broad inclusion criteria, AHPs were reluctant to approach eligible stroke survivors with communication, cognitive and low mood problems. A strong sense of duty of care to patients stemmed from perceptions that: the intensive physiotherapy intervention provided in the trial contradicted CST goals of self-management and independence; randomisation to the control group with no physiotherapy could not be blinded and might distress patients with expectations of further physiotherapy; patients might have misplaced expectations of G-CSF providing a 'miracle cure'; and wanting to identify patients that would benefit from further treatment.

**Discussion**

Gatekeeping was evident throughout the recruitment process and reflects possible tensions between research and clinical priorities. AHPs act to promote patient autonomy in their transition from acute stroke care and rehabilitation to self-management. This contrasts with protective behaviours evidenced in identifying potential patients for clinical trials and denying some eligible patients the opportunity for participation. The motivation for this was “acting” in the patient's best interests: this manifested itself in presenting the trial as the potential opportunity for further treatment when patients were discharged or not discussing the trial when they thought patient expectations of treatment were too high, or patients would not understand the implications of no active treatment allocation. Training of AHPs in study design issues should be considered especially when they have an active role in recruitment and delivery.

### O67

#### The use of regular text messaging over one year to collect primary outcome data in a randomised controlled trial

Reuben Ogollah, Martyn Lewis, Kika Konstantinou, Sarah Lawton, Jamie Garner, Nadine E. Foster
Keele University
**Correspondence:** Reuben Ogollah
*Trials* 2017, **18(Suppl 1):**O67

**Background**

Frequent data collection is desirable in randomised controlled trials (RCTs) where time-to-event is the primary outcome, in order to accurately capture the event of interest with reduced risk of recall bias. However, an inevitable problem with repeated data collection is missing data caused by participants' loss to follow-up prior to the occurrence of the event or the end of the planned follow-up period. This undermines the Intent-To-Treat (ITT) principle, leads to reduced power, and possible bias. Analytical strategies for drawing inferences from incomplete data, including the non-informative censoring for time-to-event data, rely on untestable assumptions about the missingness mechanism. Therefore, it is key to minimise the chance of dropouts at the design stage. Postal or electronically-sent questionnaires are not ideal for collecting frequent follow-up data as they often yield poor response rates even after reminder mailings. Short message service (SMS) using mobile telephones might offer a new way to enhance real-time outcome data collection. We describe the use of SMS to obtain weekly data on recovery of sciatica patients in the SCOPiC trial, describe the response patterns in the short and longer term and consider the implications for data analysis.

**Method**

We used data from 314 participants randomised to date (target recruitment of 470) to a trial that is testing the clinical and cost-effectiveness of stratified care versus usual care for patients with sciatica in primary care (SCOPiC Trial; HTA 12/201/09) in order to evaluate weekly response rates to SMS. The primary outcome measure is patient-reported time to resolution of sciatica symptoms (defined as completely recovered/

much better) measured on a six-point global perceived change scale, collected using regular SMS (with the alternative of brief phone calls for those where text messaging is not possible or missed) for the first 16 weeks for all participants, and thereafter monthly up to month 12, or until stable resolution of symptoms (defined as two consecutive SMS responses of patient-reported resolution).

**Results**

In total, 90% ($n = 283$) of participants opted for SMS follow-up and 10% ($n = 31$) for phone calls. There have been 4,299 valid responses via SMS out of the expected 4,787 (90% response rate), compared to 74% (417/567) telephone call responses. The median (IQR) weekly response rate over the first 16 weeks was 93% (90%, 95%) and 75% (71%, 77%) for texts and calls, respectively. There was no evidence of decrease in weekly response rate over time (i.e. pattern of missingness was intermittent). The median response rate for months 5 to 12 was 70% (62%, 80%) for SMS and 67% (63%, 82%) for telephone calls. 190/283(67%) and 3/31(10%) participants completed 100% of the expected texts and calls, respectively. 243/283 (86%) and 20/31 (65%) participants completed 80% texts and telephone calls, respectively.

**Conclusion**

Collecting frequent follow-up outcome data with SMS is feasible in an RCT and provides high response to both short and longer term follow-up. This could be an additional and/or alternative strategy to collecting data in large pragmatic trials, and is particularly useful for collecting regular primary outcome data, which is key to time-to-event and pragmatic ITT-evaluation.

### O68

#### Causal inference with randomised clinical trials of chemotherapy: the importance of well-documented treatment side-effects

Carlo Lancia[1], Cristian Spitoni[2], Jakob Anninga[3], Jeremy Whelan[4], Matthew R. Sydes[5], Gordana Jovic[5], Marta Fiocco[1]
[1]Leiden University Medical Center; [2]University Medical Center Utrecht; [3]Radboud University Medical Center; [4]University College London Hospital; [5]MRC Clinical Trials Unit at UCL, Institute of Clinical Trials and Methodology, UCL
**Correspondence:** Carlo Lancia
*Trials* 2017, **18(Suppl 1):**O68

**Introduction**

Randomised Controlled Trials (RCTs) are universally considered as the most reliable way to demonstrate and assess causal relationships between treatments and outcome; science-based medicine is rooted in them. Spurious relationships between the outcome and a time-fixed treatment-variable are eliminated by randomising patients over two or more arms of the trial. Hence, the randomisation procedure initiates the process by which treatment and outcomes of interest should be interpreted in a causal way. However, treatment is not always administered as intended, not least because of the occurrence of side effects and adverse events. In RCTs of chemotherapy, for example, the treatment administered may differ from the intended one because of the application of either cycle delays or dose reductions.

**Background**

Opposite to the *intention-to-treat approach*, a statistical analysis based on actual treatment data might be problematic due to the presence of the so-called *treatment-adjustment bias*. Exposure to chemotherapy agents may in fact be reduced and/or delayed as a consequence of previous-treatment side-effects. In particular, both reductions and delays contribute to lowering the value of the so-called Received Dose Intensity.

**Methods**

Inverse Probability-of-Treatment Weighting (IPTW) is a general methodology for removing treatment-adjustment bias. Working under the hypothesis of *No Unmeasured Confounding*, it creates a pseudo-population by weighting each patient with the inverse probability of observing a certain treatment administration given the past treatment and toxicity history. However, a review of data collected from RCTs on osteosarcoma suggests that treatment side-effects may not be sufficiently well-documented.

The pseudo-population created by IPTW has the following two properties:

1. Pseudo-patients' past toxicity-history no longer predicts exposure to chemotherapy in the next cycle; 2. The causal effect of treatment modifications on outcome is the same in both original and pseudo-populations.

**Results**

Using data from Medical Research Council trial BO06 (European Organisation for Research and Treatment of Cancer trial 80931) we will illustrate the use of IPTW and Marginal Structural Models (MSMs) for estimating the causal effect of dose reductions on Event-free Survival (EFS). The use of IPTW and MSMs allows to move beyond intention-to-treat and unbiasedly estimate the effect of treatment modifications on EFS.

**Conclusions**

We demonstrate that, even with complex and entangled data such as those collected in a RCT of chemotherapy, constructing and estimating a causal model is possible, provided that side-effects are well documented. When this is not the case, the removal of treatment-adjustment bias via IPTW might be problematic, if not prevented at all by the presence of unmeasured confounder. As such, good-quality toxicity data should be regarded as important enablers of causal modelling in RCTs of chemotherapy.

### O69

#### The compass study - dynamic generation of patient-specific ecare plans in a pragmatic trial

Scott Rushing, Pamela W. Duncan, Rica M. Abbott
Wake Forest Baptist Health
**Correspondence:** Scott Rushing
*Trials* 2017, **18(Suppl 1):**O69

The COMPASS Study is a pragmatic, cluster-randomized trial of 41 hospitals in North Carolina designed to determine the effectiveness of a comprehensive model of post-acute stroke care (i.e. the COMPASS intervention) compared with usual care (Control).

The COMPASS intervention includes activities such as: a follow-up telephone call two days after having been discharged; a 7–14 post-acute stroke clinic evaluation and take a patient-reported functional assessment; and generation of a individualized patient-specific care plan that is clearly discussed by the clinical provider with the patient and caregiver; and follow up phone calls 30, 60, and 90 days post discharge.

In order to efficiently implement COMPASS in participating hospitals, Wake Forest Baptist Health developed a web-based application that manages assessment collection, generates a series of helpful reports for the clinical provider and most importantly, uses a series of proprietary decision algorithms to dynamically in real time generate a patient specific care plan that identifies current health concerns for a patient, coaches the patient and family to access resources and to self manage health behaviors to improve recovery, independence, and health.

In this presentation, we will describe and demonstrate the COMPASS web-based application including the dynamic generation of patient specific care plans. Implementation of this care plan is a model of value based clinical care, meeting numerous CMS quality metrics.

### O70

#### Best practices for reporting safety data to data monitoring committees

Sonia Davis, Hengrui Sun, Kwanhye Jung
University of North Carolina
**Correspondence:** Sonia Davis
*Trials* 2017, **18(Suppl 1):**O70

**Background**

Data Monitoring Committees (DMCs) are charged with evaluation of accumulating patient safety data in ongoing clinical trials. Periodic review involves scrutinizing rates of adverse event (AE) coded terms, as well as trends and rates of abnormal values of clinical laboratory, ECG, vital sign and other assessments. DMC members of varying backgrounds complete their review in a brief time prior to and during the DMC meeting. Missing data from early drops-outs due to adverse event in the active treatment group or lack of efficacy in placebo group, plus missing at random (MAR) data caused by ongoing participants who have not yet had an assessment or experienced an event can impede data interpretation. Data reports that accurately and clearly display trends are essential for the identification of potential safety concerns. Both reviewability and statistical rigor are crucial, yet most common reporting formats used in manuscripts or study reports for completed studies lack both.

**Methods**

In this talk we present data reporting strategies that best help a DMC evaluate safety signals through (a) clear graphical displays, and (b) identification and adjustment for potentially differential rates of missing data caused both by early discontinuations and ongoing study participants. A simulation of typical DSMB data is used to demonstrate the impact of differential discontinuation on adverse event incidence rates compared to incidence densities.

**Results**

The reporting methods were implemented and refined through an unblinded independent statistical analysis center (ISAC) for a DMC of a program of phase II/III pharmaceutical clinical trials. Reviewability is substantially improved by maximizing the use of graphical displays at both the treatment group and patient level. Adverse events rates are accurately portrayed through incidence densities. We present best practices for graphical displays of disposition, AE rates, individual event profiles, assessment trends and abnormal values. All displays provide transparency of the ongoing or early discontinuation status of the participant and are producible by SAS. DMC members have assisted with further display refinement and have enthusiastically received these methods.

### O71

#### Lessons to learn from the reporting of adverse events (AEs) in randomised controlled trials published in high impact journals

Rachel Phillips[1], Victoria Cornelius[2]
[1]King's College London; [2]Imperial College London
**Correspondence:** Rachel Phillips
*Trials* 2017, **18(Suppl 1):**O71

**Introduction**

The harm profile of a drug is developed through a cumulative process incorporating information from preclinical through to post-marketing studies. An integral part of this process is the information elicited from randomised clinical trials (RCTs), as while RCTs are often not large enough to signal rare adverse drug reactions, they provide unbiased estimates of harm effects and provide a controlled comparison allowing causality to be evaluated. Many studies have demonstrated that the reporting of adverse events (AEs) in published trial reports is often inadequate, this is despite the gathering of high quality AE data required by regulators.

We undertook a systematic review to ascertain current approaches to the collection, selection, analysis and presentation of AEs in RCTs published in high impact journals. We identify examples of good practice and provide recommendations for future practice.

**Methods**

Original phase II-IV studies looking at the efficacy/effectiveness of an intervention published in the Lancet, British Medical Journal (BMJ), New England Journal of Medicine (NEJM) and Journal of the American Medical Association (JAMA) in the period September 2015 to September 2016 (inclusive) were included. RCTs where the intervention was non-medicinal and where harm/s was the primary outcome were excluded. We manually searched the archives of each journal for original reports of RCTs.

Using a standardised, pre-piloted, 60-point checklist we extracted data on trial characteristics, methods of collection, assessment of severity and causality, selection criteria for reporting, analysis methods and presentation of harm data. Descriptive analysis was performed.

**Results**

We identified 189 eligible trial reports (Lancet $n = 62$; BMJ $n = 4$; NEJM $n = 85$; and JAMA $n = 38$). AEs that cause patients to discontinue treatment or withdraw from the study are useful indicators of severity and impact to patients. We found that 56% reported the number of withdrawals/discontinuations due to AEs by treatment arm but only 14% included information on which AEs caused withdrawal. Only a quarter of reports included information on the severity of specific AEs, with several studies instead using severity to select events to report e.g. only reporting those of grade 3–5. Assessment of disproportionalities of AEs between arms was predominantly undertaken by means of tabulations, including frequencies (90%) and percentages (80%), allowing for informal comparisons. Despite a lack of power to undertake formal hypothesis testing of event counts, 39% of studies reported p-values and many studies included statements such as "no statistical differences found" to summarise the harm profile. Several studies only included AE data in online supplementary material.

We will also present exemplary examples of AE reporting, prespecified analysis methods for emerging AEs, criteria used to select which AEs to report and classification used, if any, to group AEs e.g. organ system.

**Conclusions**

Guidance is required to improve the consistency of selection, analysis and reporting multidimensional harm data in clinical trials. The use of online supplementary appendices for journals provide an opportunity to include more detailed information on AEs but this should not negate the inclusion of a useful harm profile summary in the main paper.

### O72

#### Continue or stop: the rationale for early closedown of a clinical trial as a result of stopping rules within the data monitoring committee charter

Lisa Woodhouse[1], Jason P. Appleton[1], Lelia Duley[1], Ian Ford[2], Didier Leys[3], Stuart Pocock[4], Nikola Sprigg[1], Catherine Sudlow[5], Matthew Walters[2], Philip M. Bath[1]
[1]University of Nottingham; [2]University of Glasgow; [3]University of Lille; [4]London School of Hygiene & Tropical Medicine; [5]University of Edinburgh
**Correspondence:** Lisa Woodhouse
*Trials* 2017, **18(Suppl 1):**O72

**Background**

The international Triple Antiplatelets for Reducing Dependency after Ischaemic Stroke (TARDIS) trial assessed the safety and efficacy of intensive (combined aspirin, dipyridamole and clopidogrel) versus guideline (aspirin/dipyridamole, or clopidogrel alone) antiplatelets given for one month in patients with acute stroke or transient ischaemic attack (TIA). The primary outcome was the incidence and severity of any recurrent stroke or TIA within 90 days. Recruitment commenced on 7 April 2009 but was halted on 18 March 2016, with a total enrolment of 3096 participants (of a planned target of 4100, 75.5%), on the advice of the independent Data Monitoring Committee (DMC).

**Methods**

The DMC for the TARDIS trial followed a pre-defined charter, which included stopping rules for safety and efficacy. The DMC charter did not include any stopping rules for futility. With respect to safety, the DMC charter listed the following reasons for recommending early stopping of the trial: the primary outcome favoured the control group, $P < 0.01$ (nominal, 2-sided); the combined outcome of fatal or non-fatal stroke or major bleeding favoured the control group, $P < 0.01$ (nominal, 2-sided); the overall rate of symptomatic intracranial haemorrhage exceeded 2%; that during the start-up phase, major bleeding favoured the control group, $P < 0.01$ (nominal, 2-sided). For efficacy, the DMC was to conduct formal interim analyses, after 40% and 70% of the target number of participants had had their 90 day outcome assessed, based on whether the combined outcome of fatal or non-fatal stroke or major bleeding event favoured the control group, $P < 0.001$ (2-sided). Before making any decision, the DMC was asked to consider the overall internal and external evidence, the multiplicity of testing and the possibility that trends in the data might be reversed with longer follow-up or increased recruitment.

**Results**

The DMC reviewed unblinded data in confidence every 6 months. Overall, the DMC met on 13 occasions and recommended trial continuation for all but the last data review. The recommendation to stop was based on a combination of three observations: (i) although intensive antiplatelet therapy was associated with a non-significant reduction in the primary outcome, a conditional power futility analysis suggested the trial was highly unlikely to ever demonstrate a significant difference in the primary outcome; (ii) the presence of a significant increase in major (including fatal) bleeding in participants randomised to intensive antiplatelet therapy (this increase was not present during the start-up phase as per the pre-defined stopping rules); and (iii) overall neutral findings with no difference in the net balance of death, stroke, myocardial infarction and major bleeding. The Trial Steering Committee (TSC) reviewed the same data (and subsequent additional analyses) and agreed that the trial should stop recruitment and complete follow-up on the basis of futility.

**Conclusions**

Even though the DMC charter for TARDIS did not include stopping rules for futility, it was agreed by both the DMC and TSC to stop the trial on this basis. DMCs may need to stop such trials in certain circumstances, as seen here.

### O73

#### This is a platform alteration: a trial management perspective on the operational aspects of adaptive platform trials

Francesca Schiavone, Riya Bathia, Krishna Letchemanan, Lindsey Masters, Louise Brown, Claire L. Amos, Anna Bara, Mahesh KB Parmar, Matthew R. Sydes
MRC Clinical Trials Unit at UCL, Institute of Clinical Trials and Methodology, UCL; MRC London Hub for Trials Methodology Research, London, UK
**Correspondence:** Francesca Schiavone
*Trials* 2017, **18(Suppl 1):**O73

**Background**

There is limited research and literature on the trial management challenges encountered in running adaptive platform trials. This trial design allows both (i) seamless addition of new research comparisons when compelling clinical and scientific research questions emerge, and (ii) early stopping of accrual to individual comparisons that do not show sufficient activity without affecting other active comparisons. FOCUS4 (colorectal cancer) and STAMPEDE (prostate cancer), run by the MRC CTU at UCL, are two leading UK examples of clinical trials implementing adaptive platform designs with biomarker stratified and non-stratified comparisons all within one protocol.

Adaptive platform design trials offer many potential benefits over traditional trials, from faster time to accrual to contemporaneously recruiting multiple research comparisons, added flexibility to focus on more promising research comparisons via pre-planned interim analyses and potentially shorter time to primary results. To date, STAMPEDE has added four new research comparisons, closed two following pre-planned interim analysis (lack-of-benefit) and completed recruitment to six research comparisons. FOCUS4 has closed one research comparison following pre-planned interim analysis (lack-of-benefit) and added one new research comparison, with a number of further comparisons in the pipeline.

We share here our experiences from a trial management perspective, highlighting the challenges and successes.

(Note: our lessons learnt from a central data management perspective are reported in our companion abstract: "Changing platforms without stopping the train: A Data Management Perspective on the operational aspects of adaptive platform trials").

**Methods**

We systematically evaluated the operational aspects of making changes to these adaptive platform trials and identified both common and trial-specific challenges. The operational steps and challenges linked to both the addition of new research comparisons and stopping recruitment following pre-planned interim analysis were considered in our evaluation.

**Discussion**

Specific operational challenges in these platform trials, additional to those in traditional two-arm trials, were identified that should be considered when setting up an adaptive platform trial.

During set-up of an adaptive platform trial, it is important to carefully consider the practicality of the protocol structure (modular versus single protocol), the longevity and continuity of trial oversight committees and having clear clinical and scientific criteria for the addition of new research comparisons. The common challenges in introducing a new research comparison were linked to the need for fast development timelines and a continuous training and communication programme with sites to ensure the protocol is clearly understood and followed.

Early stopping of a research comparison due to lack-of-activity also presents operational challenges, specifically when planning for stopping to recruitment is balanced with management of ongoing comparisons and sometimes the development of new research comparisons.

**Conclusions**

In our experience, the benefits arising from rapid acceleration of recruitment and shortened time to primary results outweighs the operational challenges. Careful planning is paramount when making all changes to ensure that day-to-day running is not affected and implementation of all changes is time-effective and efficient. Adaptive platform trials offer an efficient model to run randomised controlled trials and we are continuing to work to reduce further the effort required from an operational perspective.

### O74

#### Maintaining recruitment and informed consent in the advanced stages of a trial: the by-band-sleeve study

Paul Whybrow, Sangeetha Paramasivan, Jane M Blazeby, Chris Rogers, Jenny Donovan, on behalf of By-Band Sleeve
University of Bristol
**Correspondence:** Paul Whybrow
*Trials* 2017, **18(Suppl 1):**O74

**Background**

In 2012 National Institute of Health Research funded the By-Band-Sleeve study (BBS), a multi-centred, pragmatic trial comparing the effectiveness and cost effectiveness of three surgical interventions for weight-loss: the gastric bypass, the adjustable gastric band and the sleeve gastrectomy (Byrne et al. 2015). This was anticipated to be a "hard to recruit" study with the goal of recruiting 1,341 patients. The QuinteT Recruitment Intervention (QRI), a method of understanding recruitment and responding to trial recruitment difficulties (Donovan et al., 2016), was integrated with BBS. Over the last three years the QRI has informed recruitment by identifying issues with patient pathways, improving patient information leaflets and education sessions and providing regular feedback to surgeons and nurses. With 570 patients randomised, BBS is at an advanced stage of recruitment.

**Methods**

The QRI methods were employed by a) incorporating key findings from the internal pilot phase into the (pre-recruitment) training provided for the new centres in the main phase and b) concurrently initiating the QRIs in the new centres to identify challenges specific to each centre. This involved thematic analysis of a purposive sample of 198 audio-recorded consultations from 11 centres, observations of education sessions where details of the surgical procedures were explained to patients and analysis of screening log data. Findings were used to deliver group and individual feedback on recruitment across the BBS sites. Centre reviews, similar to those used in previous studies (ProtecT trial), were undertaken for the centres with particularly low recruitment.

**Findings**

The QRI identified new and unforeseen barriers to recruitment in each centre. Findings show how changes to staff, or changes to the organisation of patient care, present unexpected obstacles to recruitment.

Recruiters reported perceived changes in patient preferences or shifts in opinion within the broader clinical community. These issues undermined recruiters' confidence and ability to approach patients and obtain informed consent, bringing individual discomforts or conflicts about recruitment to the surface. Centre reviews were helpful in identifying where training and intervention could be most effective.

#### Conclusion

The By-Band-Sleeve study is a complex, multi-centred and pragmatic trial within a changeable clinical environment. The value of the QRI in the By-Band-Sleeve RCT has been in transforming recruitment in a difficult surgical RCT. More importantly, the flexible, iterative and responsive approach has helped to sustain recruitment and address emergent challenges.

### O75

### Use of data review software for data management

Leona Batten, Deborah Alawo, Lisa Jeffs, Charlotte Friend, Joanna Illambas, Sharon Ereira, Carolyn McNamara, Claire Snowdon, Judith Bliss, Emma Hall
The Institute of Cancer Research
**Correspondence:** Leona Batten
*Trials* 2017, **18(Suppl 1):**O75

#### Background

With the introduction of remote electronic data capture (EDC) at ICR-CTSU in 2012, a new process for data management was required to detect data discrepancies that would previously have been identified during central data entry. Use of a data review program was introduced in 2015. This software is now in use for several EDC trials, ?A3B2 show $132#?>necessitating a standard approach.

#### Implementation

Data are remotely entered into the EDC system by staff at participating sites and are immediately available for review at ICR-CTSU. However, only single forms can be opened in the EDC system at a time, making checks across several forms difficult. The data review software can be programmed to extract data from several forms for multiple trial visits across different participants. Reports are created, validated and saved to be run at required time points to extract live data, so irregularities can be identified and queried. This is now the principle method of data verification and validation for EDC trials at ICR-CTSU.

A data management plan (DMP) is developed for each trial and includes a comprehensive list of the data points which require verification. Data management checks can be grouped and programmed into reports produced using data review software. Higher priority checks, such as those related to eligibility criteria, safety data and endpoint data, are programmed first.

Filters can be built into reports to select specific patient subsets such as treatment cohorts. Reports can be programmed so that cells or lines are highlighted, to identify data that are outside of an expected range. Reports are saved within the system according to a standardised naming convention. Complex reports which filter or extract data from multiple forms are validated to ensure accuracy. Upon a change to the EDC system, reports may need to be altered and revalidated.

Report specification details are documented and reports are run according to priority. A log is completed to track when reports have been run. Report outputs can be exported to Microsoft Excel for further manipulation. Advanced filtering techniques can be used to distinguish new data from previously checked data.

Following the identification of data discrepancies, queries are raised in the EDC system and are available for immediate review by sites.

#### Conclusion

Data review software is now used at ICR-CTSU to facilitate data management in EDC trials. Implementing this new process can be time consuming, and manual checks are necessary while reports are being programmed and validated. Once in place the programmed checks are easy to run and streamline data management.

### O76

### Understanding methods for the assessment of patient adherence: a case study in removable devices for the improvement of trial outcome data

Zoe Tolkien[1], Shelley Potter[1], Dan Yeomans[2], Abhilash Jain[3], James Henderson[4], Jane M. Blazeby[1]
[1]University of Bristol; [2]John Radcliffe Hospital; [3]University of Oxford; [4]North Bristol NHS Trust
**Correspondence:** Zoe Tolkien
*Trials* 2017, **18(Suppl 1):**O76

#### Background and Aims

Patient adherence to treatment is a key determinant of outcome for healthcare interventions. Nonadherence likely influences therapeutic outcomes and, if unmeasured, introduces uncertainty into trial data. Whilst nonadherence has been well evidenced in settings such as drugs therapy, there is a paucity of information regarding patient adherence to rehabilitative or "removable" devices, such as splints and braces, whereby unscheduled removal may lead to irreversible deterioration of the injured structure. The aim of this study was to identify and summarise the methods reported in the literature for assessing patient adherence to removable devices to inform further research in this area and improve the quality of outcomes.

#### Methods

A systematic review was undertaken in seven electronic bibliographic databases including MEDLINE and CENTRAL, using terms relating to the device and assessment of adherence. Included were all types of trial, observational study, and case study/series prescribing a removable device after trauma or surgery. Devices were eligible for the review if they were removable by the patient, applied to a bone or joint of the appendicular skeleton, and provided full or partial immobilisation. Studies with children or animals published before 1990 were not included.

Screening and data extraction were conducted with standardised online forms developed by the researchers. Screening was conducted in full by two reviewers and discrepancies were resolved by two further reviewers. Data were summarised by study design for injury type, device, and methods for assessment of adherence. We scrutinised the papers to understand how authors valued adherence within the context of their study. The value of the methods to future research was considered.

#### Results

A total of 1655 de-duplicated records were screened for eligibility, resulting in 28 studies in the analysis (RCT = 7; Comparative study = 4; Non-comparative study = 17). The most frequently employed methods of adherence assessment were non-validated participant self-completion questionnaires and attendance at follow up appointments, with four studies using multiple methods (RCT = 3; Non-comparative = 1). More complex methods were used by one study only, i.e. sensors. The association between patient adherence and patient outcomes was evaluated by 13 studies (RCT = 2; Comparative = 1; Non-Comparative = 10) with the majority of studies reporting poorer outcomes with poorer adherence. Three studies inferring an association between outcome and adherence in the discussion presented neither methods nor data for the assessment of adherence. Reasons for non-adherence were reported by only nine studies (32%), most commonly citing discomfort as the cause.

#### Conclusions

This study provides novel information regarding methods for the assessment of patient adherence to a removable device. The majority of studies in this analysis employed low burden methods easily implemented into a trial protocol. A combination of methods would therefore represent a practical approach for future trials whilst providing both objective and subjective data for increased validity.

Given these findings, expensive and complex methods such as sensors may not be required. Developing a validated questionnaire would further increase validity and allow for comparison between studies. The paucity of data regarding reasons for non-adherence calls for a mixed methods approach including qualitative research to identify and explore patient factors.

### O77

#### Self-reported and electronic resource use for trial-based economic evaluations: the current state of play in England and considerations for the future

Matthew Franklin[1], Joanna Thorn[2]
[1]University of Sheffield; [2]University of Bristol
**Correspondence:** Matthew Franklin
*Trials* 2017, **18(Suppl 1):**O77

**Background**
Randomised controlled trials provide an opportunity to examine patient-level resource-use data to assess the costs associated with an intervention. Combined with outcome data in an economic evaluation, these measurements gauge the value for money that a particular intervention represents. Typically, researchers develop a bespoke questionnaire on a trial by trial basis to ask patients about their resource use. However, the advent of electronic data sources that are routinely collected within the health service has opened up new opportunities for collecting resource-use data without burdening patients.
**Methods**
We describe current electronic data sources that may be suitable for use in economic evaluations conducted alongside randomised controlled trials, including Hospital Episode Statistics (HES) and Secondary Users Service (SUS) datasets for secondary care in England; the Clinical Practice Research Datalink (CPRD), ResearchOne and in-practice software systems for primary care data; commissioning datasets and their potential use for research; as well as looking to future developments such as GP Connect and the need to link electronic datasets. We also describe the current status of instruments for measuring resource use via patient self-report, and discuss initiatives to improve the methodology. We assess the risks and benefits associated with each method, and compare the suitability of the two methodologies.
**Discussion**
Patient self-report forms a pragmatic and cheap method of data collection that is largely under the control of the researcher. However, there are known issues with the validity of the data collected, loss to follow-up may be high, and questionnaires suffer substantially from missing data. Routinely collected electronic data may be more accurate, although the validity of the datasets is not commonly assessed for the purposes of health economic evaluation. The use of routine datasets is likely to be more practical than patient self-report if large numbers of patients are involved. However, access to datasets often incurs a significant cost and researchers are bound by the time for data approval and extraction by the data holders.
**Conclusions**
Owing to information governance and issues with timeliness of access associated with electronic datasets, self-reported methods may currently be the preferred option. In particular, patient self-report is necessary if the study perspective encompasses costs borne by the patient themselves. However, hospital data are relatively easy to obtain electronically; in trials where secondary care constitutes a major cost driver, detailed electronic data may be considered superior to self-reported methods.

**Acknowledgments**
JT acknowledges funding from the ConDuCT-II Hub for Trials Methodology Research. MF acknowledges funding from the CLAHRC Yorkshire and Humber (CLAHRC-YH).

### O78

#### Using multiple qualitative methods to develop a complex intervention: the PACT study

Mirella Longo[1], Despina Anagnostou[2], Stephanie Sivell[2], Simon Noble[2], Jason Lester[3], Antony Byrne[2], David Jones[4], Lesley Radley[4], Catherine Sampson[2], Annmarie Nelson[2]
[1]Cardiff University, School of Medicine; [2]MCPCRC, Cardiff University, School of Medicine; [3]Velindre NHS Trust; [4]PPI
**Correspondence:** Mirella Longo
*Trials* 2017, **18(Suppl 1):**O78

**Background**
Qualitative research is often used to inform the development of complex interventions in health care. However, study designs rarely use a phased approach to fully understand the chain of events in any healthcare process, and tend to rely on a small and predictable set of data collection methods and qualitative analytical frameworks, which may not be best suited to either the research questions or data collected. We used a multimethod qualitative approach, tailored to each phase, research question, and data type to inform the development of a complex intervention to support patients and their clinicians when considering treatment options following a diagnosis of advanced lung cancer (The PACT study).
**Methods**
The PACT study is a five-stage prospective, qualitative and multicentre exploratory study. Data is collected from: I. Non-participant observation and audio recording of multi-disciplinary team (MDT) meetings to determine how patients were allocated to treatment pathways. II. Audio recording of patient-clinician consultations to measure patient's involvement in decision-making (stage II). III. From the same cohort, separate audio recorded and observed interviews with patients IV. And their clinicians to explore the perception of treatment options and involvement in decision-making. V. An expert consensus meeting to finalise the content and format of the intervention Qualitative analytical frameworks were used according to data type, research question and included: I. Mediated discourse analysis (MDA) II. Thematic Analysis and Option Grid scoring III. Interpretative Phenomenological Analysis (IPA) IV. Framework Analysis V. Consensus methodology and cognitive interviewing.
**Results**
Stages I to IV have been completed and stage V is currently underway. Data from stages I to IV provided an in-depth analysis of key turning points influencing information sharing, communication and decision-making between patients and clinicians. Based on the findings of stages I to IV, an expert consensus group to select the domains for decision-making, and mode of intervention followed by cognitive interviews using probing and "think-aloud" protocols with patients, will determine the face-validity of the intervention. The next step will test the intervention in a cluster randomised controlled trial to include QoL and economic outcomes in order to establish the intervention as effective and beneficial. This multimethod approach has enabled a reflection of the complexity of the clinical pathway, and has mapped what happens during the patient-clinician consultation. We hope that this example will encourage a broader approach to qualitative study design when developing complex interventions in health care.

### O79

#### Taking the redcap data management tool to the next step. Reducing complexity in study design by smoother integration of external randomisation services thus providing a more streamlined experience for users

Allan Walker, Garry Milne
University Of Edinburgh Clinical Trials Unit
**Correspondence:** Allan Walker
*Trials* 2017, **18(Suppl 1):**O79

The REDCap data management tool is gaining traction in the wider research community due to its generous licensing terms and its ease of use in setting up and managing research studies.
Many randomised clinical trials implement complex treatment allocation algorithms such as minimisation that are not supported by the REDCap tool. This forces study designers into building in additional complexity to the design to enable the study to proceed.
An outcome of this is that research nurses often need access to two systems' REDCap itself, plus an external randomisation system.
Additional complexity such as this should be avoided in a well-designed research study.
We propose to demonstrate a mechanism where we leverage the REDCap data trigger mechanism, the REDCap API and Edinburgh Clinical Trials Unit's own Randomisation Service to seamlessly integrate an external randomisation service with the REDCap data collection process.
The practical outcome of implementing the mechanism is that the end-users - our research nurses - are unaware they are in fact using two separate systems. Our stated aim of reducing complexity is thus achieved.

### O80

#### Adapting the quintet recruitment intervention (QRI) to optimise recruitment in an ongoing randomised controlled trial

Marcus Jepson[1], Jenny Donovan[1], Rafiyah Khan[2], Nikki Cotterill[2], Paul Abrams[2], The MASTER study group
[1]University of Bristol; [2]Bristol Urological Institute
**Correspondence:** Marcus Jepson
*Trials* 2017, **18(Suppl 1):**O80

**Introduction**
Randomised Controlled Trials (RCTs) are the most rigorous study design to evaluate healthcare interventions. However, their success relies on patient recruitment. The QuinteT Recruitment Intervention (QRI) aims to address recruitment difficulties and enhance information delivery in RCTs, using qualitative methods. It has been implemented in 26 current and completed RCTs, either as part of the initial study design, or brought in part-way through when recruitment is particularly challenging. The QRI comprises two-phases: Phase I - investigation of recruitment processes, using interviews, screening logs and recordings of trial consultations; Phase II - developing an action plan in agreement with the RCT's Chief Investigator. A QRI typically takes 12–18 months. Where trials are experiencing recruitment difficulties, and have to respond swiftly to funders it may not be possible to complete a full QRI. We report and reflect on a developmental abridged version of the QRI as applied to a UK-based RCT comparing urological surgical procedures.
**Methods**
An abridged QRI was applied at two time points, 20 and 30 months into the recruitment phase of the RCT, each culminating in a collaborators' workshop. Semi-structured interviews were undertaken with the CI, 16 recruiting urologists, and 2 research nurses (time point 1) and 5 urologists (time point 2). Interviews, screening and recruitment data were analysed to explore reasons for patient non-participation. Workshops were attended by the majority of recruiting centres and involved facilitation of an interactive discussion session based on emergent findings from the QRI. Attendees discussed the implications of these findings, and considered whether they would be able to amend their subsequent recruitment practices.
**Results**
Issues that may have hindered recruitment emerged from the analysis: different interpretations of eligibility criteria reduced the potential sample population in some centres, and different positions of equipoise emerged in relation to one very familiar and established procedure, and a newer, less well-established technique. Additionally, there was inconsistency between the reasons for patient preferences as documented in screening data and described in interviews. Prior to the first meeting, 113 patients had been randomised (average 4.7 per month). In the subsequent 10 months, 135 patients were randomised (average 13.5 per month). The second collaborators' meeting was held in November 2016. If current rates are maintained, the study is now expected to successfully achieve its recruitment target.
**Conclusions**
An abridged QRI in the form of interview data and good quality screening information, combined with the accumulated knowledge of the commonly-cited barriers to trial recruitment, appeared to lead to an increase in the average number of monthly randomisations in this RCT. If RCTs require a short-term fix to recruitment challenges, an abridged version of the QRI may be useful. However, without the benefit of a full Phase I of the QRI, there is a limited understanding of recruitment barriers and processes, reducing opportunities to offer tailored suggestions for improving communication which may be necessary in some RCTs.

### O81

#### Non-stochastic curtailment in phase ii oncology trials

Jessica Kandall[1], Sarah Brown[1], Cornelia Kunz[2]
[1]University of Leeds; [2]Lancaster University
**Correspondence:** Jessica Kandall
*Trials* 2017, **18(Suppl 1):**O81

**Background**
Two-stage, single-arm designs are commonly employed phase II oncology trials. Typically these designs aim to determine whether the experimental treatment has sufficient activity to warrant further development, without formally testing the safety of the treatment. Bryant and Day (1995) developed a two-stage design to formally evaluate both response and toxicity. This design allows early termination at the interim analysis due to inadequate response rate and/or excessive toxicities.
A disadvantage of these designs is early termination can only occur at the end of stage one, after a pre-specified number of patients are recruited and followed up. However it may be the case that during the trial, insufficient number of events occur such that early termination of the trial may occur at any point. Exposing more patients to a treatment, which has for example already observed too few responses and won't be recommended for further development, is unethical. Additionally, this is wasting precious time and resources, which could be used elsewhere. Implementing continuous monitoring throughout these designs, could help overcome this issue.
Here we consider the statistical implications of employing continuous monitoring in the Bryant and Day design, to allow stopping when the desired outcome becomes unachievable or already achieved.
**Methods**
Kunz and Kieser (2012) explained that type I error and power are preserved when non-stochastic curtailment (NSC) - a form of continuous monitoring - is implemented, as the trial is terminated once the desired result is impossible to achieve or already achieved. We therefore focus on the impact on probability of early termination and expected sample size. We consider the case where a treatment successfully makes it past stage one and continuous monitoring occurs in stage two.
Simulations were used to implement NSC in stage two of the Bryant and Day design considering a range of scenarios. Response and toxicity rates between 0.05-0.8 and 0.6-0.95 were investigated, respectively. The probability of early termination and expected sample size conditional on making it to the second stage of the trial were calculated, when allowing early stopping for futility or efficacy (simulated under the null or alternative hypotheses respectively).
**Results**
Under all scenarios investigated, on average 77% of trials were terminated early during the second stage for futility, compared to 89% for termination due to efficacy. Without curtailment, the mean total sample size was 29.9 patients, whereas when allowing early stopping for futility during stage two 26.4 patients were expected and for efficacy 26.7. This equates to savings of 12% and 11% respectively.
**Conclusion**
On average, a smaller sample size was required when implementing NSC in scenarios with larger toxicity rates. When allowing stopping for futility, greater savings in expected sample size were seen in the scenarios with larger toxicity rates. However greater savings were seen in the lower toxicity scenarios, when stopping for efficacy. The probability of early termination was significantly larger when allowing early stopping for efficacy compared with futility.
Where recruitment is slow or observations may be monitored in a short-time frame, NSC for futility and efficacy may be beneficial.

### O82
### Design issues of a non-inferiority trial: prompt panretinal photocoagulation versus intravitreous ranibizumab for proliferative diabetic retinopathy (a tale of two diseases)

Isoken Odia, Michele Melia
Jaeb Center for Health Research
**Correspondence:** Isoken Odia
*Trials* 2017, **18(Suppl 1):**O82

Non-inferiority trials are often used to compare a novel treatment with an active treatment in hopes of showing the novel treatment is not clinically worse than the proven standard therapy. Factors such as the choice of non-inferiority margins make these trials difficult to design, but these trials become significantly more complex when the condition being treated is associated with another disease that warrants additional treatment, making it difficult to tease out treatment effects. The Diabetic Retinopathy Research network designed a non-inferiority trial to determine whether intravitreous ranibizumab (IVR), an anti-VEGF agent, with deferred panretinal photocoagulation (PRP) resulted in outcomes that were non-inferior to prompt PRP alone for the treatment of proliferative diabetic retinopathy (PDR). Diabetic macula edema (DME) can occur concomitant with PDR and is usually treated with an anti-VEGF drug if visual acuity is decreased. In this trial, about 35% of the prompt PRP group received the study drug (IVR) in addition to prompt PRP for the treatment of DME at baseline, and an additional 18% received it during follow up, after developing DME. This presentation will highlight some of the challenges encountered in designing this trial such as choosing an appropriate non-inferiority margin, appropriate statistical methods and approach to treatment of DME in the standard treatment group. We discuss the advantages and disadvantages of the approaches taken to design this trial and discuss alternatives for handling these challenges. Our experience will help to inform investigators on possible solutions to similar situations in the future.

### O83
### Use of minimization in multi-center clinical trials - when to not add randomization

Elaine Pascoe, Darsy Darssan, Liza A. Vergara
The University of Queensland
**Correspondence:** Elaine Pascoe
*Trials* 2017, **18(Suppl 1):**O83

Minimization is increasingly recommended as the method of choice for allocating subjects to treatment groups in clinical trials. The method is designed to eliminate chronological bias by guaranteeing similar numbers of patients in treatment groups throughout the trial recruitment phase and to ensure treatment groups are balanced on prognostic and other baseline characteristics at the end of the trial. Primarily because of its superior capacity to balance treatments across large numbers of baseline variables, minimization has been referred to as the platinum standard for treatment allocation in clinical trials. In multi-center trials, study center is typically one of the minimization variables. In its original form, minimization deterministically ($p = 1.0$) allocates the next patient to the least common treatment for previously allocated patients with the same combination of baseline characteristics. Subsequent extensions to the minimization algorithm included the addition of a random element to the allocation process (p between 0.5 and 1.0) to mitigate the risk of a clinician predicting the next treatment to be allocated. We argue that the risk of selection bias is negligible in multi-center trials that use an interactive central allocation system (web-based or voice response). We present data showing that the addition of a random component to the minimization algorithm can be detrimental to the objectives of minimization and reduce the efficiency with which a trial can be conducted. Unpublished data from case studies of on-going and completed multi-center clinical trials that are using or used minimization with a random component show that imbalance in treatments at study centers often occurs throughout the recruitment phase and treatment balance at all study centers is not guaranteed at the end of the trial. Through simulation studies we show that these problems are less evident when minimization is used without a random component. We discuss the contexts (e.g., medication kits shipped in advance of need and stored at study centers) in which trial inefficiencies and cost overruns occur with the inclusion of a random element to the minimization algorithm. We conclude that consideration should be given to not adding a random component to minimization in multi-center clinical trials that use an interactive central allocation system and recommend that decisions about the statistical aspects of trial design, especially decisions about treatment allocation methods, be made in the context of planned procedural and operational aspects of a trial.

### O84
### Implementing a risk based monitoring approach in the early phase myeloma portfolio at Leeds CTRU

Lucy Bailey, Fiona K. Brudenell Straw, Suja E. George
University of Leeds
**Correspondence:** Lucy Bailey
*Trials* 2017, **18(Suppl 1):**O84

Employing efficient strategies for monitoring clinical trials is paramount to mitigating risks using limited resources. While effective monitoring is crucial in protecting the well-being of trial participants and maintaining the integrity of data, in recent years there has been a gradual shift towards a risk based monitoring (RBM) approach. In 2011, An MRC/DH/MHRA Joint Project produced a paper entitled “Risk-adapted Approaches to the Management of Clinical Trials of Investigational Medicinal Products”, which aimed to facilitate a risk-proportionate approach in applying the principles of GCP when running clinical trials in the UK. In the same year guidance was also released by the U.S Food and Drug Administration on the expanded use of RBM.
Like many clinical trials units, Leeds Institute of Clinical Trials Research (LICTR) previously assessed the risk for the trial as a whole and established a relatively inflexible monitoring plan whereby a designated number of visits was performed at each of the sites, with extra visits only being performed at sites that recruited large numbers of participants. Sites that had a good record of trial conduct and data compliance received the same number of visits as sites that had a poor record. The only monitoring above the standard would be when a triggered monitoring visit was required, such as if a site had breached the protocol and this was reportable to the MHRA. In order to prioritise finite resources, a formal, documented methodology was required for analysing trial-specific risks, and determining when more frequent, targeted monitoring was necessary.
With this goal in mind, a risk-based monitoring tool was developed for use in the early phase myeloma portfolio being run at the LICTR. Data regarding site performance is collated on a monthly basis. Each metric relating to the safety of participants, data management, regulatory compliance and adherence to protocol has parameters for low, medium or high risk scores. Sites are given an overall risk rating dependant on the number of areas in which they are considered a low, medium or high risk. The risk assessment is examined regularly throughout the course of the trial to ensure that the risk score and, subsequently, the monitoring approach is amended according to the changing quality of site performance. This tool involves collaboration between trial coordinators, data managers and monitors and enables simple and quantitative reporting at meetings and gives closer alignment of data management, trial management and site monitoring processes. Our monitoring plans still incorporate visits to every site but are now also adaptive, allowing the monitoring strategy for a particular site to evolve as the trial progresses.

This presentation will detail how we adapted the RBM approach and implemented a site monitoring tool for our phase two trials in the myeloma portfolio.

### O85

#### Preliminary analysis of a new measure of quality of patient decision making about research participation

Peter Knapp[1], Jonathan Graffy[2], Peter Bower[3], Joanna Rick[3], Caroline Fairhurst[1], Sarah Cockayne[1], S. Rogers[1], D. Torgerson[1]
[1]University of York; [2]University of Cambridge; [3]University of Manchester
**Correspondence:** Peter Knapp
*Trials* 2017, **18(Suppl 1):**O85

**Objectives**

To report the rationale for, and initial performance data of, a measure of quality of patient decision making about research participation.

**Background**

To understand patients' decisions about research participation, including trials, or to evaluate interventions intended to boost research recruitment rates, it is useful to assess quality of decision making about participation. It would be equally useful to do this when the patient has opted to participate or has declined. The QuIC measure [Joffe, 2001] has been used in several studies but there are concerns about its completion rate and the relevance of some items to non-drug trials. The ICQ measure [Guarino, 2006] has some useful items but also those that depend on post-study completion. There are established, well-performing measures of patient decision making about treatments [O'Connor, 1995; Parayre, 2014] but both these measures contain items that do not apply to decisions about research participation. Given these problems we devised a new measure and tested it with a small sample of people who had consented to take part in the REFORM study [Cockayne, 2016].

**Methods**

The measure was intended to be short (to limit patient data burden) with items referring to elements of participant information required under UK research governance. The measure has 5 items (covering: potential benefits of participation; potential disadvantages; likely experience of participation; possible changes to treatments or care; and an evaluation of the overall extent to which choice was informed), each using 5 Likert responses (from 'strongly agree' to 'strongly disagree', scored 5 to 1 respectively) This derives a total score with possible range 5–25, and higher scores indicating greater agreement. The measure was included in the baseline pack posted to participants in the REFORM study [Cockayne, 2016], which used a "trial within a cohort study" design to evaluate the effectiveness of a falls prevention intervention in older people living at home. The measure was posted to 301 people (aged 64–98; 50.8% were female) in 4 centres in northern England.

**Results**

280 (93.0%) participants completed at least some of the measure, of whom 98.9% completed all items. The mean total score was 20.03 (SD 2.44; range 12–25; mode 20; median 20). Individual item means ranged from 3.81 ("possible changes to treatment or care") to 4.24 ("overall informed choice"). Internal consistency was high (Cronbach alpha 0.83) and alpha was not increased by the deletion of any of the 5 items.

**Discussion & Conclusions**

The scale was acceptable to respondents and shows promise as a measure of the quality of decision making about research participation. Mean scores were relatively high, which may indicate the quality of information provision in this study but could also reflect the positive wording of the measure's items. Further evaluation is needed, particularly in studies with different age groups and lower participation rates. Also needed are consideration of a threshold approach to scoring, defined by minimally acceptable values to indicate that informed consent has been obtained; and evaluation of the measure's ability to discriminate and its sensitivity to change.

### O86

#### Assessing clinically relevant endpoints in a pragmatic trial: development of a measure to identify relevant clinical symptoms

Charlotte Murkin[1], Kerry Avery[1], Sian E. Cousins[1], Natalie S. Blencowe[1], Leila Rooshenas[1], Karen Coulman[1], Daisy Elliott[1], N. J. Smart[2], Jane M. Blazeby[1], On behalf of the CIPHER study group[2]
[1]University of Bristol, School of Social and Community Medicine; [2]Exeter Surgical Health Sciences Research Unit (HESRU), Royal Devon & Exeter Hospital
**Correspondence:** Charlotte Murkin
*Trials* 2017, **18(Suppl 1):**O86

**Background**

The importance of pragmatic study design is gaining increasing recognition in research. A distinctive feature of this is the selection of clinically relevant outcomes that are meaningful to patients. Studies, therefore may choose to focus on the symptomatic condition of interest rather than the asymptomatic presence of a condition alone. However, there are challenges associated with this, including the selection of symptoms and their assessment. Here we describe the methods used to design a questionnaire that aims to identify the important symptoms of the relevant condition. In a future study, we intend to set a threshold to define participants as symptomatic and use this as part of the definition of the primary outcome. This work highlights how built in feasibility work can be used to develop patient centred symptom-based outcomes to inform a pragmatic trial design.

**Methods**

The first phase of the project (Phase A) consists of built in feasibility work designed to inform the future study (Phase B). This methodological work, performed as part of Phase A, has three main components: i) Identification of known and unknown healthcare domains from the literature and qualitative interviews with patients living with and without the condition and interviews with clinicians and specialist nurses, ii) Development domains into questions within a questionnaire for completion by patients, iii) pre-testing the questionnaire in patients living with and without the condition to refine the instrument and develop thresholds (green, amber and red) to trigger further investigation for the condition of interest.

**Results**

169 relevant descriptions of known symptoms were identified from the literature, which were grouped into health domains independently by two researchers, resulting in 19 domains that included pain, body image and social functioning. These domains formed the basis of the topic guides for the semi-structured interviews. 10 healthcare professionals with clinical experience of the condition of interest and 17 patients (9 living with the condition and 8 without, on review of medical records) were interviewed. The topic guides were updated iteratively as new lines of enquiry were uncovered during the interviews. Interviews were analysed through constant comparison approaches. Transcripts were coded line by line and categorised into themes using NVivo software. The Interviews identified 16 additional symptom domains. Work is ongoing to develop items from the domains. Future work will pre-test the tool and establish thresholds to define participants as symptomatic.

**Conclusion**

This mixed methods feasibility work developed a patient centred outcome measure to inform a pragmatic study design. It focuses on endpoints relevant to patients (i.e. a symptomatic condition of interest). Further work is planned in the main study to validate the clinical findings with symptomatic and asymptomatic patients.

### O87

#### Record linkage of trial datasets to hospital admission records: a trial manager's experience

Suzanne Breeman, Jessica Wood, Graeme MacLennan, Jemma Hudson
University of Aberdeen
**Correspondence:** Suzanne Breeman
*Trials* 2017, **18(Suppl 1):**O87

#### Background
Collection of outcome data within randomised controlled trials (RCTs) can be challenging, especially in trials with a lengthy follow-up phase. If the outcomes of interest are participant-reported, for example, quality-of-life, then participant contact is unavoidable. However, if the outcomes are clinical, for example, re-operation or hospitalisation then record linkage of trial datasets to hospital admission records (or other routine data-sets) may be an option and could potentially overcome some of the issues associated with participant retention.
### Methods
Two surgical RCTs that utilised a dual model of data collection whereby clinical outcomes were reported by the participant and through record linkage were examined. In both trials the use of hospital admission data was appropriate as the key clinical outcome was further surgical intervention for reoccurrence of the condition. Consent for record linkage was obtained from all participants at the onset of the trials which allowed the collection of data from those participants who subsequently declined further questionnaire follow-up or who were lost to follow-up (unless their consent to do so was withdrawn). Both trials have reported their primary outcome and one has entered a longer follow-up phase.
### Results
The supplementation of participant-reported clinical outcomes with routine hospital admission data allowed us to better evaluate the key clinical question; approximately 20% of relevant hospital readmissions were obtained from routine admission data only. However the exercise also highlighted potential issues with the coding of routine admission data: for 5.8% of index operations, the coding in the routine data did not match that recorded within the trial dataset.
### Discussion
Inconsistencies between the participant-reported account and the record linkage report were detected. In an attempt to address this issue contact was made with the hospital site to extract information directly from the patients' notes. If no response was received, the participant's record of the event was included in the analyses in both trials. For an event reported by record linkage that could not be verified by the hospital (and was not reported by the participant) the event was excluded from the analyses.
Issues surrounding the collection of record linkage data were also identified. This was particularly problematic for data collected through the Health and Social Care Information Centre (now known as NHS Digital). Problems encountered included a lengthy application and approval process, mainly due to changes in the application process, the name of the service provider and to the legal gateway for consent.
We also experienced lengthy applications to extend retention periods beyond the initial agreement (to comply with sponsor and funders regulations) and extensive data security checks of the University's policies. In addition, there is still ongoing confusion regarding data classification, in particular, what constitutes derived reversible or irreversible source data.
### Conclusions
The collection of hospital readmission data to supplement participant-reported outcomes was a worthwhile exercise across both trials as it resulted in a more complete dataset. However it was a time consuming process and had implications in terms of staff time preparing the application, analysing the data and resolving any inconsistencies observed.

### O88
## Contamination in trials of complex interventions: measuring and accounting for it in the analysis of a trial
Nicholas Magill, Khalida Ismail, Paul McCrone, Sabine Landau
King's College London
**Correspondence:** Nicholas Magill
*Trials* 2017, **18(Suppl 1):**O88

#### Background
In mental health trials there is concern that the control treatment might be contaminated, meaning that a participant in the comparator arm receives the active intervention. This can often be avoided by design through the use of cluster randomisation, with clusters defined by the level at which contamination is thought to take place. One alternative to this problem is to account for contamination in the analysis by using complier average causal effect (CACE) estimation.
### Method
This methodological research was motivated by a trial of nurse-delivered cognitive behavioural therapy and motivational interviewing for people with poorly controlled type 2 diabetes in primary care. Participants were offered either this (in addition to standard care) or standard care with attention control. The primary outcome was glycated haemoglobin. Contamination was anticipated if a given nurse were asked to deliver both intervention and comparator treatments. Therefore cluster randomisation was used with treatment allocation at the level of the primary care centre. However, in practice both non-compliance and contamination did occur due to variation in nurses' skills and possibly as an effect of the attention control design.
Treatment fidelity was measured using two fidelity scales, which were then used to construct measures of treatment receipt and subsequently to account for non-compliance and contamination in the analysis of the trial. One measure was of binary treatment receipt and this was used to perform a CACE efficacy analysis using instrumental variable methods. Another was a continuous measure of dose of treatment. In addition, existing methodology was developed to allow the estimation of the effect of exposure on the causal treatment effect where exposure is dose of treatment and is measured in control and intervention participants.
### Results
The treatments that those in the intervention and control arms received were unexpectedly similar. This appeared to represent a mixture of treatment non-receipt in the active arm (non-compliance) and treatment receipt in the control arm (contamination).
The intention-to-treat analysis suggested little effectiveness of treatment. The efficacy analyses, which accounted for non-compliance and contamination, gave estimates that were a little larger and with wider confidence intervals. However, these did not show a statistically or clinically significant effect.
### Conclusions
The similarity between the treatment groups may in part explain the lack of a treatment effect in this trial. This research has demonstrated the importance of measuring treatment fidelity for all participants as a post randomisation process variable. Such a measure enables an assessment of fidelity and adjustment for treatment receipt in the results of a trial of a psychotherapy. The motivating trial also highlights the difficulty of training non-clinical psychologists to provide psychotherapy and possibly the drawback of the use of attention control.

### O89
## Successful recruitment to a large online randomised trial: the TIME study
Amy Rogers, Isla Mackenzie, David Rorie, Thomas M. MacDonald
University of Dundee
**Correspondence:** Amy Rogers
*Trials* 2017, **18(Suppl 1):**O89

#### Background
The TIME (Treatment in Morning versus Evening) Study is a randomised study of the effect of timing of antihypertensive medication administration on cardiovascular outcomes using an online methodology [1]. This presentation will describe and discuss the methods used to surpass the study recruitment target. Background TIME aims to determine if antihypertensive therapy taken in the evening results in improved cardiovascular outcomes compared with conventional morning dosing. Over 20,000 participants, followed-up over 4 years, will be required to generate the necessary number of cardiovascular events to answer this question. The TIME study is funded by the British Heart Foundation and we wanted to develop novel methods of recruitment to maximise cost efficiency.
### Methods
Prospective TIME participants are invited to visit a secure study website, https://www.timestudy.co.uk/. All study procedures are conducted online. Several different methods were used to identify potential participants including: letters sent from Primary and Secondary Care (using the NHS-approved Docmail service), emails sent to participants in biobank projects, pharmacy searches and more traditional advertising by poster and print media articles.

**Results**

21,415 participants throughout the UK were randomised between the start of the initial pilot study in 2012 and November 2016. The total cost of recruitment methods was £277,139. Primary care patient identification (PIC) sites sending letters of invitation generated the most participants (n = 16657). An arrangement with UK Biobank inviting potential participants by email resulted in 3210 randomisations. Recruitment strategies such as posters and media advertising had little impact on recruitment despite relatively high costs.

**Discussion**

Successful recruitment to the TIME study was mainly due to a combination of efficient study design with the use of IT to facilitate sending high numbers of study invitations at low cost. It is hoped that these methods can be used to streamline the recruitment process for future studies.

1. Rorie DA, Rogers A, Mackenzie IS, Ford I, Webb DJ, Willams B, et al. Methods of a large prospective, randomised, open-label, blinded endpoint study comparing morning versus evening dosing in hypertensive patients: the Treatment In Morning versus Evening (TIME) study.

### O90

#### Building comprehensive searches through a machine learning approach for systematic reviews

Ileana Baldi[1], Corrado Lanera[1], Clara Minto[1], Dario Gregori[1], Paola Berchialla[2]

[1]University of Padova; [2]University of Torino

**Correspondence:** Ileana Baldi

*Trials* 2017, **18(Suppl 1):**O90

**Background**

Systematic reviews of clinical trials are an important means of synthesizing medical evidence. The validity of systematic reviews depends on the ability to fully capture the complete body of evidence through searches of many data sources. Nevertheless, examination of clinical trials registry databases is not routinely included in systematic reviews (Jones et al., 2014). A possible explanation is that all databases have their own unique features and creating consistent search strategies across literature and registry databases may be challenging.

**Materials and Methods**

The main idea is to use the title and abstract text fields of relevant articles, identified by a literature search, for training purposes of a machine learning algorithm. Next, the trained algorithm may be used to identify documents with a comparable word characterization in a registry database. A hypothetical research question concerning nursing research is used to demonstrate the proof-of-concept. The PubMed database was searched for articles published from 2006/01/01 to 2016/11/01, tagged with Clinical trial Publication Type and appearing in journals allocated to the Nursing subject area according to the Journal Citation Reports®. To provide a training set with "positives" and "negatives", we selected also a set of clinical trials run in other research fields. The following text pre-processing procedures were applied to the title and abstract fields of the retrieved records in the following order: conversion to lowercase, removing numbers, removing punctuation, removing stop-words, stemming words, stripping white space, and building a sequence of two adjacent words from the text (bigrams). The whole collection was finally tokenized in a document-term matrix (DTM) which was used to train and test a logitBoost MLT classifier (Dettling and Buhlmann, 2002). The classifier was 10-fold cross-validated using 1,000 bootstrap iterations at each step. Next we accessed records of all clinical trials registered in the International Clinical Trials Registry Platform (ICTRP), established by World Health Organization in 2006. After eliminating duplicates, the same above mentioned text pre-processing strategy was applied to the title and condition fields and the DTM was built. A prediction task was then performed by applying the validated classifier to the ICTRP DTM.

**Results**

In the PubMed training set, the sensitivity of the classifier was good (81.2%) and the specificity was excellent (99.3%). When applied to the ICTRP database it allowed to identify a set of protocols likely pertaining to nursing research. We are currently organizing data and code in a R software package which will be freely available on GitHub.

**Conclusions**

Routine examination of registry databases deserves further consideration since it may allow a more accurate characterization of publication and outcome reporting biases and improve the validity of systematic reviews. The approach described here provides an automated solution that can be tailored to address a variety of clinical trial-related questions by building a comprehensive search on both literature and registry databases.

### O91

#### Response and progression endpoint review in a large phase III randomised control trial of multiple myeloma

Alina Striha[1], David A. Cairns[1], Corinne Collett[1], Charlotte Pawlyn[2], Phillip Best[1], Andrea Paterson[1], Inga Sakauskiene[1], John Jones[2], Mark T. Drayson[3], Graham H. Jackson[4]

[1]University of Leeds; [2]Institute of Cancer Research; [3]Clinical Immunology Service, University of Birmingham; [4]Northern Institute for Cancer Research, Newcastle University

**Correspondence:** Alina Striha

*Trials* 2017, **18(Suppl 1):**O91

**Background**

Response review is a necessary stage in the verification of efficacy endpoints in trials of multiple myeloma (MM) as it provides an indicator of quality assurance. UK NCRI Myeloma XI is probably the largest randomised clinical trial in newly diagnosed patients with MM conducted so far, with 4420 recruited patients. A large trial requires a robust method of assessment of response and progression throughout the patient pathway. As Myeloma XI requires response assessment after completion of key treatment periods, and progression monitoring throughout the protocol, a semi-automated verification process is considered essential to ensure robust results and manage resources efficiently.

**Methods**

Response and progression in MM were defined according to the International Myeloma Working Group (IMWG) Criteria. Myeloma XI collected local laboratory results alongside local response assessments, and for consenting patients - equivalent data from an experienced central laboratory. All data was stored on bespoke trial databases which could be easily imported into statistical software packages. We developed a suite of SAS programs which apply the IMWG criteria to reported laboratory values and verified local and central response and progression assessments, and validated local and central assessments against each other. If assessments were concordant, these were considered to be validated and required no further review. If results were discordant, the patient file was selected for review by a committee of clinicians.

Preparing the clinical review data involved collation of all local and central results available. This was achieved using a suite of R and Java programs to produce an MS Excel clinical review file for each patient. The clinical review file contained derived and reported assessments, as well as details of key assessment timepoints within the study. The review files were sent to clinicians for review via a Secure File Transfer System (SFTS). The reviewer considered available data to determine the response/progression and for difficult and discordant cases, a clinical committee meeting was convened to determine a consensus or classification of the case as unable to determine (UTD), or to request that more data be sought from site. When review was completed the review file was returned via SFTS.

**Results**

At final analysis in July 2016, 3894 patients had undergone central review. At this time 1848 subjects had demonstrated progression and 3795 had a response assessment. After computer-based verification and validation of progression, 443 patients (24.0%) required subsequent clinical review. A similar process for response resulted in 477 patients (12.6%) requiring clinical review. All remaining progressions and responses were considered verified without the need for review by clinician (83.7% of potential response and reviews). In clinician review of progression a decision was reached in most cases with only 25 patients (5.6%) classified as UTD.

**Conclusions**

This semi-automated process of central review will increase confidence in the robustness of reported results from Myeloma XI. This is an efficient process which has saved man-hours for data management and clinical reviewers. The software tools developed are being further validated for use in data management and central review of new trials of MM.

### O92

#### Exploring and adjusting for potential learning effects in ROLARR - a randomised controlled surgical trial

Neil Corrigan, Helen Marshall, Julia Brown
University of Leeds
**Correspondence:** Neil Corrigan
*Trials* 2017, **18(Suppl 1):**O92

ROLARR is an international, randomised controlled, confirmatory surgical trial designed to perform a rigorous comparison of robotic-assisted surgery against standard laparoscopic surgery for the curative treatment of rectal cancer. At the beginning of trial recruitment, the standard laparoscopic approach was already well-established, with many centres and expert surgeons proficient in delivering the intervention, whereas the robotic-assisted approach was relatively new, with only several centres having an established track record of delivering it. Proficiency in standard laparoscopic surgery coupled with enthusiasm for, but relatively little experience with, robotic-assisted surgery was a common trait of potential participating surgeons in ROLARR.

This situation is common in surgical trials and must be addressed during the design and analysis, since a naïve comparison of outcomes of patients who underwent a well-established technique in which the operating surgeons were already proficient vs. patients who underwent a new technique which operating surgeons were still learning would be inappropriate. Ideally, comparisons between the two interventions should be made when they are both being performed optimally i.e. when surgeons are proficient in both. Traditionally, this issue is addressed at the design stage by stipulating that any participating surgeon in the trial must have some minimum level of experience in each intervention before randomising any patients. This was done in ROLARR, with inclusion criteria stipulating that participating surgeons must have performed at least 30 rectal cancer operations (with at least 10 using each intervention).

However, as alluded to by Cook, Ramsay & Fayers [1], this is only a partial answer to the problem; in principle, statistical exploration of any potential learning effects within a trial is required to more fully address the issue. Following this guidance in ROLARR, data on the number of robotic-assisted and standard laparoscopic rectal cancer operations performed both within and outside of the trial was collected periodically throughout the trial. At analysis, for every ROLARR operation performed we had data about how many previous robotic-assisted and standard laparoscopic rectal cancer operations the operating surgeon had performed. This allowed us to perform sensitivity analyses exploring potential learning effects within the trial, in particular addressing the question of whether or not the estimated difference between the arms yielded by our primary analysis changed depending on operating surgeon experience.

Collecting this data and performing these analyses allowed us to quantify and adjust for the confounding effect that the difference in proficiencies between the arms had on the primary analysis, which was invaluable to our understanding and interpretation of the outcomes that we observed in ROLARR. Details about the approaches used in ROLARR, the resulting learning effects analyses that were performed and the impact that they had on our understanding of the outcomes that we saw in trial will be presented. Time permitting, the limitations of these particular analyses, including areas where further work is required and recommendations for future trials, will also be discussed.

### O93

#### A framework for identifying treatment-covariate interactions in individual participant data network meta-analysis

Suzanne Freeman, David Fisher, Jayne Tierney, James Carpenter
MRC Clinical Trials Unit at UCL; MRC London Hub for Trials Methodology Research
**Correspondence:** Suzanne Freeman
*Trials* 2017, **18(Suppl 1):**O93

**Background**

Stratified medicine aims to identify patients most likely to respond to treatment. However, individual trials are rarely powered to detect interactions between treatment effects and participant characteristics. Network meta-analysis (NMA) models potentially have greater power to identify such treatment-covariate interactions, particularly when individual participant data (IPD) are available. The Royston-Parmar model, fitted in a Bayesian framework using WinBUGS, provides a practical and flexible approach for IPD NMA of time-to-event data. Pairwise IPD meta-analysis of treatment-covariate interactions contains so-called “within-trial” and “across-trial” information, where across-trial information is particularly susceptible to confounding and ecological bias. The same is true of IPD NMA; therefore it is important that the within- and across-trial information are reviewed separately, before deciding whether to combine them. A further frequent complication is missing patient-level covariate data. We show that using a Bayesian framework for IPD NMA allows this to be handled relatively easily.

**Methods**

Our motivating data come from a network of IPD from 37 trials comparing combinations of radiotherapy, chemotherapy and surgery from 5922 patients with cervical cancer (2394 events), where age and disease stage are two potential interaction covariates. We show how the Royston-Parmar NMA model can be fitted using restricted cubic splines, and then extend it to include treatment-covariate interactions. We propose two models: (i) two effects separating out the within- and across-trial information and (ii) a single interaction effect (combining within- and across-trial information). We argue for a visual assessment of the consistency of the within- and across-trial information. We also discuss more detailed aspects of meta-analysis interaction modelling, such as common vs trial-specific main effects of the covariate, and suitable approaches for handling missing covariate data. This leads us to propose a practical framework for IPD NMA with treatment-covariate interactions.

**Results**

The cervical cancer network showed no evidence of a treatment-age interaction, but there was some evidence for a treatment-stage interaction. A visual assessment highlighted inconsistency between the within- and across-trial information. Following our proposed framework, the within- and across-trial information should not be combined. The remaining within-trial evidence for a treatment-stage interaction is weak; most evidence for an interaction is across-trial, with the NMA providing additional power for this over and above the pairwise evidence.

**Conclusion**

A Bayesian approach for NMA using the Royston-Parmar model with splines naturally handles missing covariate data and allows the separation of within- and across-trial treatment-covariate interaction information. Our proposed framework, incorporating this model, provides practical guidance for researchers, and reduces the risk of unduly optimistic interpretation of treatment-covariate interactions.

### O94

#### Formative research findings on the design of an early enrollment clinical trial on hospital-acquired bacterial pneumonia (HABP) and ventilator-associated bacterial pneumonia (VABP)

Amy Corneli[1], Brian Perry[2], Deborah Collyar[3], John H. Powers[4], Helen K. Donnelly[5], Carisa De Anda[6], Denise Hinton[7], Jonas Santiago[7], Sara B. Calvert[8], Vance G. Fowler Jr. [1]
[1]Duke University; [2]Duke Clinical Research Institute; [3]Patient Advocates In Research; [4]George Washington University School of Medicine; [5]Northwestern University; [6]Merck & Co., Inc.; [7]Center for Drug Evaluation and Research, U.S. Food and Drug Administration; [8]Clinical Trials Transformation Initiative
**Correspondence:** Amy Corneli
*Trials* 2017, **18(Suppl 1):**O94

**Background**
The Clinical Trials Transformation Initiative (CTTI), a public-private partnership established by the FDA and Duke University, is interested in assessing whether an Early Enrollment (EE) strategy can improve enrollment rates for HABP/VABP clinical trials. Using this proposed strategy, patients at high risk for developing HABP or VABP, or their legally-authorized representatives (LARs), would be asked to give their consent to enroll in a trial before the patient develops symptoms of pneumonia. Patients would agree to being monitored by study staff, and if pneumonia develops, to being randomized to receive the study antibiotics. We conducted formative research to assess the acceptability of and preferences for components (e.g., opt-out process) of an EE strategy for a HABP/VABP clinical trial among key stakeholders.
**Methods**
We conducted 52 qualitative, in-depth interviews with patients at risk for HABP/VABP (n = 18), caregivers/LARs (n = 12), members of Institutional Review Boards (IRBs) (n = 10), investigators (n = 7), and study coordinators (n = 5). We provided stakeholders with an overview of the proposed EE study prior to questions on acceptability and preferences. We also explored the acceptability of two treatment designs: randomization to 1) one of two FDA-approved antibiotics commonly used in hospitals to treat pneumonia (one approved for lower respiratory infections and the other for serious infections other than pneumonia), and 2) a new, investigational antibiotic (not FDA-approved) compared to a standard-of-care antibiotic. We used applied thematic analysis to analyze the data.
**Results**
None of the patients or caregivers/LARs expressed concern about study staff accessing patients' medical records to monitor for infection. Nearly all patients (88%) believed they could understand the study and give consent, even though they would not have pneumonia at the time of enrollment. While appreciated by many (56%), some patients (44%) said they may experience anxiety after being informed they are at risk for pneumonia. None of the IRB members raised concerns regarding the EE strategy, although suggestions were made about information to include in the study protocol. Most investigators and coordinators (83%) believed the EE strategy would improve recruitment. Half of IRB members believed an opt-out process was necessary and sufficient, while 40% believed it was unnecessary because initial consent was provided and participants can always withdraw from research at any time. Most patients (65%) favored an active opt-out process at the time of randomization. Stakeholders' views were mixed on the LAR's role in the opt-out process when patients provide initial consent but later become unable to opt out. While patients and caregivers/LARs did not perceive any physical risks from enrolling early, perceived risks were associated with treatment: using two FDA-approved antibiotics was viewed as low risk, whereas the "unknown" aspects of a new investigational treatment generally heightened risk perceptions. As a result, most patients (67%) and caregivers/LARs (67%) were willing to enroll in a trial with FDA-approved antibiotics but were far less willing to participate if an investigational antibiotic would be used (patients = 28%, caregivers/LARs = 25%).
**Conclusions**
The EE strategy was overwhelmingly accepted by stakeholders. Sponsors should evaluate whether this strategy improves enrollment rates in prospective HABP/VABP clinical trials.

### O95

#### Reducing bias in randomised controlled trials involving therapists: to randomise or not to randomise - What are the questions?

Alexandra Wright-Hughes, Rebecca Walwyn, Liz Graham, Sadie Reed, Amanda Farrin
University of Leeds
**Correspondence:** Alexandra Wright-Hughes
*Trials* 2017, **18(Suppl 1):**O95

Clinical trials of behavioural and psychological interventions often involve therapists or other clinical specialists. This adds further complexity to the trial design and leads to considerations relating to clustering effects (i.e. intra-cluster correlation, cluster size), randomisation, contamination, and the impact on analysis. In individually-randomised controlled trials (RCTs) the involvement of more than one therapist per site can introduce selection bias, whereby therapists can choose which participants they treat. If therapist selection is based on participants characteristics, for example with the "best" treating the "worst", the effects of therapists and participant characteristics may be confounded. This limits the inferences a trial is able to make, particularly if the therapist effect is of interest in its own right. It also reduces the ability to attribute treatment effects to treatment alone, thus impacting a trial's internal validity. To reduce the overall sample size required, and avoid problems fitting analysis models, trialists may also wish to ensure similar cluster sizes. To avoid this source of selection bias, allocation of therapists to participants can be concealed and random. However, doing so under constraints of therapist capacity, location, and a trial's relative priorities can be problematic. The success of therapist involvement and implications on randomisation can be further influenced by the context in which they work (e.g. involvement of 'research' therapists or those working within the NHS).
We summarise our experience of randomly allocating therapists in three clinical trials with different designs: (i) SHIFT, an NIHR HTA-funded phase III definitive RCT set in NHS Child and Adolescent Mental Health Services (CAMHS), had teams of Family Therapists (employed for the research) working together to deliver therapy across services. Where teams worked across a number of services, the lead therapist was randomised, otherwise participants were assigned to the single lead therapist for a particular service. (ii) TIGA-CUB, an NIHR RfPB-funded feasibility RCT, similarly set in CAMHS, has greater constraints on therapist capacity. Trial therapy is provided 'within' therapists NHS role, with variable numbers of therapists per service, and complications for therapist allocation to primary carer as well as child participants. (iii) OK-Diabetes, an NIHR HTA-funded feasibility RCT recruited community-based participants with diabetes and a learning disability. Diabetes nurse specialists were allocated to participants based on prior contact to ensure consistency in contact and nurse randomisation occurred where there had been no prior contact.
Challenges have included the practicality of randomising therapists within a restricted service, therapist capacity following variable recruitment rates, impact on recruitment, waiting lists, and deviations from the allocated therapist. Randomising therapists also has potential implications for the intention-to-treat principle, where participants are analysed as allocated, with consideration required as to how to analyse according to therapist.
The context in which therapists work, their capacity and the needs of the trial population all influence the feasibility and appropriateness of randomising therapists. We will discuss the methodological benefits of including therapist randomisation and the implementation challenges this introduces, providing guidance on when it is appropriate and how to ensure it 'works' for therapists, participants and trial delivery.

### O96

#### Developing a comprehensive site selection process for a cancer network in a resource-limited settings in sub-Saharan Africa

Megan Wirth, Dikla Blumberg, Kimberly Mosby-Griffin, Don Vena
Emmes Corporation
**Correspondence:** Megan Wirth
*Trials* 2017, **18(Suppl 1):**O96

**Background**
To successfully execute clinical trials the study operations team must have an appreciation of the capabilities of the clinical sites. This is particularly vital in developing countries where necessary infrastructure, availability of qualified staff and proper facilities are often scarcer than in wealthier nations. The AIDS Malignancy Consortium (AMC) is a National Cancer Institute supported multicenter clinical trials group founded in 1995 to support innovative trials for AIDS-related cancers. In 2010 the AMC was tasked with expanding operations internationally in sub-Saharan African countries with a high prevalence of HIV, with the goal of identifying and engaging a core group clinical sites that were capable of conducting contextually appropriate therapeutic and prevention trials in a variety of HIV-associated cancers and contributing to the AMC's scientific agenda.
**Methods**
In response to this new initiative, the AMC developed a two-tiered strategy to select international sites. This began by soliciting applications from 14 sites in which a member of the site staff had a pre-existing relationship with an AMC investigator, and systematically gathering written information about their resources, clinical research experience and active collaborations with other clinical trials organizations. After review of these data, the nine top-ranked sites participated in a structured data collection exercise that involved collection of disease-specific patient information about three AIDS-related cancers, auditing that data, and conducting site visits. Each site visit team included an oncologist and a representative from the AMC Operations and Data Management Center (ODMC) and evaluators filled out a site visit worksheet at the end of each visit.
**Results**
The information gathered from the sites provided insight into the identification, diagnosis, and treatment of patients at each of the sites. However, it was the on-site visits that proved most valuable in assessing suitability for participation in AMC trials. Based on the site visit evaluations the four top sites (in Zimbabwe, Uganda, Kenya, and South Africa) were selected for participation. Lessons learned from the implementation of the process allowed the ODMC to improve site selection methods by standardizing the site visit worksheets, and asking more targeted questions that better identified particular issues that were critical to study conduct. These changes streamlined the process and allowed for better comparison of the sites in the next round of site selection that occurred in 2014–5, during which three additional sites were selected.
**Conclusions**
The AMC has developed a detailed and methodical process for selecting international sites that have the clinical research infrastructure, data management operations and the human and material resource capacity required to successfully participate in clinical trials in HIV-associated malignancies. In our experience, the most essential and valuable part of that process is the on-site visits, which have been successful in vetting research sites that would be able to provide high quality data and contribute to AMC's mission of investigating new treatment and prevention interventions of malignancies in people living with HIV both in the USA and internationally. Strategies for maximizing the value of these visits and collection of material from the visits will be discussed.

### O97

#### Using causal diagrams to guide analyses of randomized controlled trials with missing outcomes

Elizabeth Turner[1], Melanie Prague[2]
[1]Duke University; [2]INRIA, Bordeaux University
**Correspondence**: Elizabeth Turner
*Trials* 2017, **18(Suppl 1):**O97

**Introduction**
It is common for randomized controlled trials (RCTs) to have some missing outcomes. Yet recent reviews indicate that some authors analyze using only available outcome data (sometimes called a complete records analysis) without explicitly addressing the nature of the missingness. In most cases, this will lead to bias due to selection of a non-representative population. This can throw in to doubt the evidence generated from the trial. However, when proper adjustments are made, a complete records approach can provide valid inference under certain conditions. Yet, there are misconceptions and difficulties in determining when such an approach is valid.
**Background**
There is a growing literature in the field of causal inference that focuses on the use of causal diagrams to determine when bias might arise in estimating a causal relationship of interest. Recent work by Daniel et al. (SMMR 21(3); 2011) used this approach to guide analyses with missing data. In the current talk, we adapt their approach to be specifically applicable to the RCT setting. Our goal is to provide a strategy to determine when a complete records analysis will be unbiased and therefore to determine when alternative methods (e.g. imputation or weighting) are required in order to avoid biased estimation of the intervention effect.
**Methods**
We consider three outcome-generating models where the outcome (Y) is dependent on: (1) the treatment arm (A) only, (2) on both treatment arm and a predictive baseline covariate (X) through an additive relationship, and, (3) additionally dependent on the interaction of A and X so that there is treatment heterogeneity by levels of the predictive covariate X. For each of these three outcome-generating models, we consider a range of common missing outcome mechanisms including: missing completely at random (MCAR), missing at random (MAR), including covariate dependent missingness (CDM), as well as specific examples of missing not at random (MNAR) mechanisms. Specifically, we consider the following missing data mechanisms, where the probability of an outcome being missing is dependent: (1) on neither A nor X (MCAR), (2) on treatment arm only, (3) on X only, (4) on A and X additively, (5) additionally on an interaction between A and X so that there is differential missingness by treatment arm (all examples of CDM), (6) on observed levels of the outcome (MAR), and, (7) on unobserved levels of the outcome (MNAR).
**Results and conclusion**
We show that in order to determine when a complete records analysis is valid it is as important to focus on the form of the outcome data-generating model as well as the missing data mechanism. By providing an easily implementable strategy with simple rules, we seek to aid a broad audience to understand when analysis of only available RCT data will be sufficient to address the causal research question of interest without the need for the sometimes complex imputation and weighting methods that are commonly used to address missing outcome data.

### O98

#### An adaptive design for the identification of the optimal dose using joint modelling of efficacy and toxicity in phase I/II clinical trials of molecularly targeted agents

Maria Altzerinakou[1], Xavier Paoletti[2]
[1]CESP OncoStat, INSERM, Université Paris-Saclay, Université Paris-Sud, UVSQ, Institut Gustave Roussy, Villejuif, France; [2]Institut Gustave Roussy, CESP OncoStat, Université Paris-Sud, UVSQ, INSERM, Université Paris-Saclay, Villejuif, France
**Correspondence:** Maria Altzerinakou
*Trials* 2017, **18(Suppl 1):**O98

**Background**
Conventional dose-finding approaches in oncology of phase I trials aim to identify the maximum tolerated dose (MTD), based on toxicity events observed during the first treatment cycle. Even though this is relevant for cytotoxic agents, this may not be the case for molecularly targeted agents, usually administered in chronic schedules. Lately, continuous biomarkers are used more and more to monitor efficacy. However, efficacy does not necessarily increase monotonically with dose and as corollary both toxicity and efficacy should be considered for the identification of the optimal dose. The optimal dose will be defined as the lowest dose that achieves high efficacy, while satisfying certain toxicity requirements.

**Methods**

We present an adaptive dose-finding method, using a joint modelling technique (Barrett J. et al., 2015) of a longitudinal outcome for continuous biomarker efficacy measurements and a probit time to first severe toxicity model, with shared random time slope. This method allows for exact likelihood inference, an important property, in the context of both missing at random due to low efficacy or increased toxicity and the case of small sample sizes. We combined this technique with the continual reassessment method, and used data collected over all treatment cycles. The selection of the dose at the entry of every new patient was based on the trade-off algorithm, proposed by Thall and Cook (2004), adapted to the situation of continuous efficacy measures. For the evaluation of the method we run a set of simulations for two scenarios. Dose and time were included in both models, with the addition of dose-time interaction for the longitudinal outcome. We considered up to 9 dose levels, 3 treatment cycles, a maximum of 12 visits for the efficacy measurement and 66 patients.

**Results**

In the first scenario the optimal dose was the first one and the MTD the second. The optimal dose was chosen at 85.6% of the cases, and another 12.8% was assigned to the MTD. For the second scenario, we assumed the fourth dose to be the optimal and the fifth to be the MTD. The optimal dose was suggested for 32.3% of the cases, while the MTD for 17.3% of them. At no point was the last dose level recommended. Our design performs very well when the optimal dose is at the edge of the dose range. This is because the desirability of certain doses around the optimal dose and MTD tends to be very close to one another. Even though this may be an issue when the optimal dose is located at the middle of the dose range, it is also the reason patients are very rarely allocated to doses far from the optimal dose. Finally, the method had interesting behavior regarding its ability to select the right dose in presence of missing data due to severe toxicity.

**Discussion**

In the future, we would like to test more scenarios and to evaluate more flexible models for dose-response relationship, providing the number of parameters to estimate remains in line with the sample size.

### O99

#### Detection of incomplete data in surveillance and impact of missing data in assessment of effectiveness of financial incentives of viral load suppression in HPTN 065

Deborah Donnell[1], Bernie Branson[2], Wafaa El-Sadr[3], Sahar Zangenah[1]
[1]Fred Hutchinson Cancer Research Center; [2]Scientific Affairs LLC; [3]Columbia University
**Correspondence:** Deborah Donnell
*Trials* 2017, **18(Suppl 1):**O99

**Background**

Pragmatic trials often rely on pre-existing data systems to evaluate trial outcomes. HPTN 065 used US HIV Surveillance data to evaluate the outcomes of a clinic-randomized strategy trial to test the use of financial incentives to improve viral load suppression outcomes in the Bronx NY and Washington DC.

**Methods**

In a collaborative effort between study and surveillance staff, aggregate clinic outcomes were defined using variables available in surveillance data and aligned with the financial incentive intervention. Outcomes were centrally programmed by surveillance experts for evaluation using local surveillance databases. Pre-trial data on trial outcomes were used to conduct a restricted randomization of the 38 trial clinics, with the goal of achieving balance in pre-trial viral load suppression and number of patients per clinic. During the trial, triangulation of data from the clinics, surveillance and the financial incentive delivery was used to assess data completeness. Sensitivity analysis and multiple imputation were subsequently used to evaluate 1) randomization balance based on incomplete pre-trial data in the restricted randomization; 2) the impact of incompleteness in baseline data on efficacy evaluation and 3) the sensitivity of the trial results to sites with missing data during the primary evaluation period.

**Results**

Data triangulation throughout the trial revealed missing data in surveillance for some clinics in Washington DC. Evaluation of data inconsistencies and investigation into the causes of incompleteness, together with extensive collaboration with HIV surveillance staff, were largely successful in remediating the missing data for the trial evaluation period. Some baseline data could not be corrected due to lost access to data. During initial trial evaluation of effectiveness, exploratory data analysis of time trends revealed a small number of clinics with more subtle data incompleteness issues, complicating the evaluation of the effectiveness of financial incentives. Increased imbalance on the restricted factors was observed using corrected data compared to the pre-trial data. Missing data in the baseline outcome assessment decreased the precision of efficacy estimates, with a 57% higher SE of the efficacy estimate in DC vs. NY. Trial efficacy results were sensitive to the effect of missing data, with the initial analysis of effectiveness of financial incentives showing an increase in viral load suppression of 3.9% (−3.4%, 11.1%; $p = 0.27$), changing to 3.7% (0.5, 6.9%) $p = 0.022$ after data completeness was addressed in Washington DC HIV surveillance.

**Conclusion**

Program assessments that utilize external data sources to evaluate outcomes need to conduct ongoing exploratory data analysis to understand and monitor data quality and completeness during the trial as trial results and study power will be affected by problems in the data source. Close collaboration with data source experts is critical to assure quality and completeness of outcome data.

### O100

#### Up-and-down designs enhanced with SPRT rules for phase I cancer trials

Assaf Oron[1], Nancy Flournoy[2]
[1]Seattle Children's Hospital Research Institute; [2]University of Missouri
**Correspondence:** Assaf Oron
*Trials* 2017, **18(Suppl 1):**O100

Up-and-Down (U&D) is an established dose-finding approach used in many fields, yet all but forgotten by the Phase I design community. With respect to finding Phase I's Maximum Tolerated Dose (MTD), when used with an appropriate estimation method U&D is on par with more complicated state-of-the-art designs, and its overall behavior is more tractable and stable (Oron and Hoff, 2013). However, unlike some novel methods U&D cannot "sharpen" its dose-allocation random walk over time.

We present an enhancement to U&D, one that gradually limits dose allocations to within a tolerance interval around the MTD, using Sequential Probability Ratio Test (SPRT) decision rules. The interval boundaries can be rendered "soft" or "hard", via SPRT decisions that are reversible or not, respectively. With some limitations, SPRT-enhanced U&D converges almost surely to a random walk confined inside the tolerance interval. An asymmetric "soft/hard" rule combination is possible, and is rather suitable for toxicity studies such as Phase I.

SPRT-enhanced U&D generates treatment distributions more sharply peaked around the MTD than ordinary U&D, and with fewer toxicities on average. Despite the added complication, it is still simpler, more robust, and more tractable than most leading novel designs, thus presenting a highly attractive design choice for Phase I trials. The development and use of SPRT-enhanced U&D for several ongoing Phase I immunotherapy trials at Seattle Children's Ben Towne Center is described.

### O101

#### Impact of results reporting in clinicaltrials.gov

Kevin Fain, Deborah A. Zarin, Rebecca J. Williams, Tony Tse, Thiyagu Rajakannan
ClinicalTrials.gov Program
**Correspondence:** Kevin Fain
*Trials* 2017, **18(Suppl 1):**O101

#### Background

Since 2008 U.S. federal law requires the submission of summary results to, and public posting on, ClinicalTrials.gov for certain clinical trials of FDA-regulated drugs, biologics and devices. We aimed to determine ways in which reporting to ClinicalTrials.gov has expanded the public availability and use of clinical trial information. Specifically, we assessed the extent to which ClinicalTrials.gov has improved the reporting of "full sets" of trials for specific drug products (aim 1). We also examined how researchers have used ClinicalTrials.gov data (aim 2).

#### Methods

For aim 1, we examined ClinicalTrials.gov records for phase 2–4 clinical trials of drugs or biologicals with at least one U.S. study location, completed or terminated between 2007–2009, and funded by industry. We grouped trials into a "drug trial family" when the drug, condition, and sponsor appeared to be the same across trials. We limited our analysis to a convenience sample of the first 96 drug trial families identified. For each trial, we assessed the presence of results posted on ClinicalTrials.gov and publications cited in PubMed as of June 2015. For aim 2, we searched PubMed to identify publications in English between 2010–2015 that conducted original research using data retrieved from the ClinicalTrials.gov registry and/or results database. Authors manually reviewed eligible publications to categorize how ClinicalTrials.gov data were used in the study.

#### Results

For aim 1, our sample of 96 drug trial families included 329 trials, studying 86 drugs for 78 conditions by 45 companies. Of the 329 trials, 109 (33%) had results posted on ClinicalTrials.gov only, 42 (13%) in PubMed only, 81 (25%) in both, and 97 (29%) in neither. Of the 96 drug trial families, 45 families had results disclosed in at least one source for all trials. Of these 45 families, 15 families had results disclosed only in ClinicalTrials.gov. For 18 drug trial families with a total of 48 trials, no results were available from any source.

For aim 2, we identified 404 research articles and 1,588 systematic reviews published between 2010–2015 that used data from the ClinicalTrials.gov registry, results database, or both. We categorized each research article into: characterization of clinical research for specific conditions (48%); research on ethics, adverse event reporting, and data mining (15%); quality of registered data and consistency with reporting policies (10%); characterization of the overall clinical research landscape (10%); evaluating publication bias or selective reporting (9%); and assessing specific research-related methods and issues (7%).

#### Conclusion

We found that results for 33% of sampled clinical trials were available only on ClinicalTrials.gov, suggesting the database may be a useful resource to identify clinical trial results that would not otherwise be publically available for specific drug products. Although there were still gaps in the public availability of trial results, the existence of the trials is publicly known because of the registry entries. ClinicalTrials.gov data is increasingly being used to assess a broad range of research questions, many of which would have been difficult or impossible to address using published literature alone.

### O102

#### A common data language for clinical research studies: five (5) new disease recommendations from the national institute of neurological disorders and stroke, national institutes of health common data elements project

Joy Esterlitz[1], Sherita Ala'i[1], Robin Feldman[1], Kristen Joseph[1], Muniza Sheikh[1], Claudia S. Moy[2], Sara S. Rue[2], NINDS CDE Working Groups[2]
[1]The Emmes Corporation; [2]The National Institute of Neurological Disorders and Stroke
**Correspondence:** Joy Esterlitz
*Trials* 2017, **18(Suppl 1):**O102

#### Objectives

In 2016–2017, the National Institute of Neurological Disorders and Stroke (NINDS), NIH, and their partners have put forth new data standards recommendations for the following diseases: (1) Cerebral Palsy; (2) Chiari I Malformation; (3) Headache, Version 2.0; (4) Sport Concussion; and (5) Subarachnoid Hemorrhage. The partners in development of these common data element (CDE) recommendations are the American Academy for Cerebral Palsy and Developmental Medicine, the Chiari & Syringomyelia Foundation, the Department of Defense, and the National Library of Medicine. Goals of the overall project include reducing study start-up time; increasing the efficiency and effectiveness of clinical research studies and treatment; and increasing data quality and data sharing across studies.

#### Background

CDEs were developed for 18 neurological disease areas prior to these five newly developed areas. There are over 11,000 CDEs in the NINDS library and over 600 validated instruments, Case Report Forms (CRFs), and study recommendations. The CDE initiatives strive to identify CDEs, template study forms, data dictionaries and guidelines to assist investigators who are initiating and conducting related clinical studies. Use of standardized CDEs is critical to ensure that data housed within a database are of better quality; improve data sharing and meta-analyses; and help educate new clinical investigators.

#### Methods

Each new disease CDE working group (WG), consisting of 40–60 worldwide experts with varied fields of related expertise, met regularly to select from existing CDEs or refine and add from field-tested data elements from national registries and funded research studies. For most diseases, these WG members are divided into subgroups applicable for each disease based on domains (e.g., Imaging, Demographics, Treatment/Interventions, Biomarkers). For Sport Concussion, subgroups are based on disease characteristics; Acute, Sub-Acute and Persistent/Chronic. All WGs began by reviewing CDEs previously developed for different diseases, conditions and symptoms to avoid unnecessary duplication. After the subgroup recommendations are completed, there is an internal review followed by public review in which time the recommendations are posted on the NINDS CDE Website for 1–2 months.

#### Results

All comments are collated and incorporated into the final CDE version release on the NINDS CDE website. The website provides uniform names and structures for each data element, as well as guidance documents and template CRFs using the CDEs. WGs are also given the charge to classify the recommended CDEs as "Core, Supplemental-Highly Recommended, Supplemental or Exploratory" according to set definitions from the NINDS CDE project.

#### Conclusion

Researchers who receive funding from NINDS are encouraged to use the CDEs in their CRFs and data management systems whenever possible. To date, the feedback has included the CDE project's impact on reducing study start-up time. Continued feedback is essential. The NINDS CDEs are a continually evolving resource, requiring updates as research advancements indicate. These newly developed CDEs serve to be a valuable starting point or update for many neurological disease researchers and facilitate harmonization, streamlining and sharing of data.
Support: This project was funded by HHSN271201200034C.

### O103

#### Using electronic health records in clinical trials: rising to the challenge of developing a data linkage pipeline - experience from the ISCOMAT programme

Suzanne Hartley[1], Gerry Armitage[2], Alison Blenkinsopp[2], Chris Gale[1], Beth Fylan Gwynn[2], Ivana Holloway[1], Claire Hulme[1], Hanif Ismail[2], Duncan Petty[2], Mohamed Amin Mohammed[2]
[1]University of Leeds; [2]University of Bradford
**Correspondence:** Suzanne Hartley
*Trials* 2017, **18(Suppl 1):**O103

**Background**
Electronic health records (EHRs), available for every patient who visits the UK NHS, are primarily used to inform the care of patients. They contain clinical information about primary and secondary care and treatments provided. Current literature suggests that EHRs can be used to inform the design and analysis of clinical trials, subject to appropriate data protection and governance, with potential to be more efficient in terms of costs and data return. Research funders are keen for trials to consider the use of routine data sources, where possible. Here we describe our progress with accessing EHRs and other electronic data sources to support our research that crosses primary and secondary care. Methods: The National Institute for Health Research (NIHR) ISCOMAT Programme (Improving the safety and continuity of medicines management at care transitions) aims to design and evaluate an intervention to make best use of medicines and reduce harm through effective medicines management for heart failure (HF) patients following hospital discharge and across the primary care transition. It is a series of interlinked projects, culminating in a definitive cluster randomised controlled trial of 2,100 HF patients from 42 NHS Trusts in 3 UK regions. The primary endpoint is all-cause mortality and HF related hospitalisations from hospital discharge. Key secondary endpoints are medications prescribed post discharge. The majority of these data are held in the EHR.
A data linkage project is in progress to determine the feasibility of the EHRs to obtain the data for the definitive trial. We will recruit 60 HF patients, who will provide written informed consent to access their EHR data from multiple data providers. The output will be (1) robust data linkage algorithm; (2) processes to access data for use in the definitive trial.
**Current progress**
We have recruited 43 patients and actively engaged with five data providers: National Institute for Cardiovascular Outcomes Research (NICOR) for national HF audit data; SystmOne & EMIS clinical systems for primary care data; NHS Digital for secondary care and mortality data and Community pharmacy/NHS Business Authority for dispensing and prescribing data.
**Discussion**
We will describe progress with accessing data and our approach to developing a data linkage pipeline. This includes: Mapping contact points where decisions are made about medicines management and identifying data providers at each contact; Mapping data fields from each provider with primary and key secondary endpoints; Identifying process to allow access to the data from the patient and from the data provider; Establishing robust processes to allow for the data to flow from the provider to the research team, including multiple data sharing agreements; Familiarisation with data sources through access to dummy data sets; Implementing governance requirements and submitting requests for data.
We will describe challenges, variations in processes across data sources; the impact on establishing the data linkage pipeline and how this knowledge will streamline processes for the definitive trial. The use of EHRs is becoming more established with trials, thus we provide recommendations for trialists seeking to access EHR across different health care providers.

### O104

#### Analysis of trial-specific training during the site initiation phase: the ATLAS study

Athanasia Gravani[1], Marcus Jepson[2], Caroline Wilson[2], Athene Lane[2], Chris Rogers[3]
[1]University of Bristol; [2]MRC ConDuCT-II Hub for Trials Methodology Research, School of Social and Community Medicine, University of Bristol, Bristol, UK; [3]Clinical Trials and Evaluation Unit, School of Clinical Sciences, University of Bristol, Bristol, UK
**Correspondence:** Athanasia Gravani
*Trials* 2017, **18(Suppl 1):**O104

**Background**
Trial-specific training provided during the start-up phase of studies has not been thoroughly investigated. In fact, little attention has been given to the role and structure of trial-specific training and how best to organise and implement it to ensure in-depth understanding of the trial protocol and trial-related procedures by trial staff.
**Aims**
To investigate the ways in which trial-specific training is provided during site initiation and explore trial managers; and site staff views on the importance of trial-specific training during study conduct. Moreover, ATLAS aimed to evaluate the process of trial-specific training and make recommendations for improving the educational support provided to site staff during the site start-up process.
**Methods**
Six studies were purposefully selected to serve as case studies for the ATLAS study. Within each case study, trial managers ($n = 6$) facilitating trial-specific training sessions as well as healthcare professionals (principal investigators and research nurses, $n = 13$) from trial sites receiving the training, were interviewed between June 2015 and April 2016. Semi-structured face-to-face and telephone interviews were conducted using a topic guide. Interviews were audio-taped, transcribed verbatim and analysed thematically using NVivo. Non-participant observations of trial-specific training sessions ($n = 13$) as well as questionnaires completed by trial managers and site staff ($n = 120$) were also used to gain an overview of site staff and facilitators' experience.
**Results**
A variety of training modes (face-to-face, teleconference, online) were used to deliver training across the six case studies. However, face-to-face interaction was considered most beneficial in facilitating learning and enhancing personal relationship building and networking. Despite acknowledging the significance of the decision-making process in selecting the appropriate level and mode of training required, this process was often overlooked at the early planning stages of trials and was usually poorly documented. Although evaluation of site initiation training to identify key areas where follow-up training might be required and improve future training sessions was considered best practice, it was not routinely conducted. The purpose of site initiations slightly differed between trial managers and site staff. Trial managers' focus was to check that everything was in place at the site and that staff were aware of their roles and responsibilities in the trial, whereas, site staff viewed site initiations as the best time to meet and connect with the trial team. Additional training and support provided to site staff over the course of the trial was considered of paramount importance in ensuring trial's successful conduct. However, this was mainly provided retroactively on an ad-hoc basis.
**Conclusions**
There is high variation and uncertainty on the ideal ways to provide trial-specific training to facilitate trial conduct. Based on the findings of the ATLAS study, a more standardised approach in the form of a training toolkit was developed. The ATLAS training toolkit aims to bring together information from the current literature, regulations and various stakeholders' views to advance the trial-specific training process.

### O105

#### Implementing and running an online trial - a case study using the react trial

Duncan Appelbe[1], Heather Robinson[2], Susie Dodd[3], Andrew Walker[2], Paula Williamson[3], Fiona Lobban[2]
[1]The University of Liverpool; [2]The Spectrum Centre for Mental Health Research, Lancaster University; [3]NWHTMR, Department of Biostatistics, The University of Liverpool
**Correspondence:** Duncan Appelbe
*Trials* 2017, **18(Suppl 1):**O105

**Background**
The REACT study (https://www.reacttoolkit.co.uk/) is an online study to compare the effectiveness of a Relatives Education And Coping Toolkit (REACT) with an online Resource Directory. Half the people in the study receive the REACT toolkit, and half will receive the Resource Directory. In this study the intervention and data collected from the participants is provided via the Internet, with little or no direct interaction with the study team. This study opened in April 2016 and recruitment will close in early 2018. The REACT study comprises two disparate systems, the first being a bespoke data collection system that manages the eligibility/consent/registration/data collection processes, whilst the second system is a customised version of WordPress that is used to deliver both arms of the intervention.
Participants within this study do not attend regular clinical appointments. As such retention at the two follow-up points and usage of the intervention requires automated reminders and more importantly the triggering of warnings/help to participants when they provide certain answers to specific questions.
The data required to determine the effectiveness of the intervention is taken from two sources: The response by the participants to outcome measures and data collected on the usage of the application (obtained from google analytics, server logs and custom reporting software).
**Objective**
This presentation will discuss the electronic solutions required by the design of the study and the processes by which these systems were designed and implemented. A comparison of the data collected via google analytics and the server logs will be compared and commented on, with a discussion about discrepancies being made.
**Conclusions**
Online interventions and data collection systems provide great promise for efficient trial design however, there are challenges to ensure that systems are user friendly and intuitive to use, yet still allow the collection of data to validate the outcome of the study.
The views expressed are those of the authors and not necessarily those of the NHS, the NIHR or the Department of Health.

### O106

#### New cochrane risk of bias tool for cluster randomised tools

Sandra Eldridge[1], Marion Campbell[2], Mike Campbell[3], Amy Drahota[4], Bruno Giraudeau[5], Julian Higgins[6], Barney Reeves[6], Nandi Seigfried[7]
[1]Queen Mary University of London; [2]Aberdeen University; [3]Sheffield University; [4]University of Porstmouth; [5]University of Tours; [6]Bristol University; [7]South African Medical Research Council
**Correspondence:** Sandra Eldridge
*Trials* 2017, **18(Suppl 1):**O106

**Background**
Bias has long been recognised as an issue that can seriously compromise the validity of study trial results. Assessing bias from trial reports is often not easy. The Cochrane risk of bias tool is well established tool for facilitating this. This tool has recently been updated and as part of the new tool there is a specific adaptation for cluster randomised trials which takes account of the design of these trials and specific issues for assessing bias in these trials.
**Aim**
The aim of the tool is to support reviewers of trial reports to assess bias in cluster randomised trials. In this talk we describe the aspects of bias which are particular to cluster randomised trials or operate differently in these trials from the way they operate in individually randomised trials.
**Methods**
A multi-disciplinary group of researchers including statisticians, other triallists, those leading the development of the new Cochrane risk of bias tool and experts in cluster randomised trials met over a period of a year to discuss the five different bias domains (bias arising from the randomization process, bias due to deviations from intended interventions, bias due to missing outcome data, bias in measurement of the outcome, bias in selection of the reported result) that are part of the new Cochrane risk of bias tool and how they relate to bias in cluster randomised trials.
**Results**
Given the extent of the differences between assessing risk of bias in individually randomised trials and in cluster randomised trials, the group developed an adapted Cochrane risk of bias tool for cluster randomised trials. Differences occur in relation to assessing allocation concealment; appropriate assessment of bias in relation to blinding of participants and assessors; and ensuring missing clusters are considered in addition to missing values from participants. We also added an additional domain (bias arising from the timing of identification and recruitment of individual participants in relation to timing of randomization) to cover the bias that may occur when individual participants in a cluster randomised trial are recruited after randomisation.
**Conclusions**
Assessing bias in cluster randomised trials is not the same as assessing bias in individually randomised trials. Authors and peer reviewers should be aware of key elements to include in trial reports to provide evidence that their trials are protected from bias. Systematic reviewers should use the Cochrane risk of bias tool adapted for cluster randomised trials to assess these trials for bias.

### O107

#### Continuous safety monitoring in large phase I cancer clinical trials with multiple expansion cohorts

Masha Kocherginsky[1], Theodore Karrison[2]
[1]Northwestern University; [2]University of Chicago
**Correspondence:** Masha Kocherginsky
*Trials* 2017, **18(Suppl 1):**O107

Traditional phase I oncology clinical trial designs have morphed into multiple, parallel phase I trials incorporating concurrent expansion cohorts across multiple diseases or disease subtypes. These cohorts are used to provide additional safety data, as well as preliminary efficacy data. As a result, some of them have become quite large. For example, a recent study [Cancer Letter, Oct 7, 2016] looked at PD-1 drug development, and identified 35 phase I trials with 200 patients. In such trials, dose escalation is typically done using a standard phase I design, e.g. "3 + 3", to determine the maximum tolerated dose (MTD), which is followed by the enrollment of multiple expansion cohorts in different disease types or biomarker-defined subgroups. Safety is typically monitored only within each expansion cohort, and the results are not jointly evaluated in real time across the multiple expansion cohorts.
Here we propose to use a continuous safety monitoring approach based on the sequential generalized likelihood ratio test (SGLR) and discuss its application to adverse event (AE) monitoring for such large phase I trials with multiple concurrent expansion cohorts. This approach is commonly used in vaccine studies [Shih et al.; Statistics in Medicine, 2009] to monitor rare events, and can be similarly used in phase I clinical trials to monitor the frequency of rarer high-grade or serious adverse events (SAEs), as well as to further evaluate whether the presumed MTD is near the targeted percentile. Trial designs based on this approach will consist of a continuous safety monitoring boundary with a rejection number for each additional AE, while preserving the overall Type I and Type II error rates for the assumed AE rates. The boundaries can be calculated using existing software.
We consider two different implementations of the SGLR procedure: the first employs an overall monitoring rule based on pooled data across the multiple cohorts; the second monitors each cohort separately, allowing for dose reduction in a given cohort if the boundary is exceeded. We simulate data using multiple expansion cohorts with different Poisson rates of AE's, and evaluate the operating characteristics of these SGLR procedures.

### O108

#### How frequently should a disease be monitored? The interval length between observations in interval-censored data

Fang-Shu Ou, Martin Heller
Mayo Clinic
**Correspondence:** Fang-Shu Ou
*Trials* 2017, **18(Suppl 1):**O108

**Background**
Clinical trials in oncology use disease-free survival (DFS) or progression-free survival (PFS) as study endpoints. DFS and PFS are composite endpoints of disease status and overall survival. The tumor status is typically measured by a computerized tomography (CT) scan which can be done every few months and the scan interval is typically determined by the treatment guideline. For example, DFS and PFS are common endpoints used in stage 3 colon cancer study and stage 4 colorectal cancer study, respectively. In stage 3 colon cancer, the CT scan interval used to be every 3 months and was extended to every 6 months. Recently, 12-month intervals between CT scans was proposed to reduce unnecessary radiation exposure. In stage 4 coloretal cancer, the typical CT scan interval is every 8–12 weeks due to the aggressive nature of the late stage disease. In clinical trials, if disease recurs or progresses, the event date is the date of the CT scan. As the interval length between each assessment gets longer, we lose more precision regarding the actual event time and increase the potential bias as well. In this study, we examine how much information is lost due to interval-censoring, in particular, the number of events required to recover the loss of power.
**Methods**
A numerical approximation of the exact distribution function for the hazard ratio (HR) sample statistic was used to assess the effect of interval censoring and determine the actual number of events needed to attain a given power. Monte-Carlo simulation was used to examine the extent of power loss using typical assumptions for stage 3 and 4 colorectal cancer trial design.
**Results**
For stage 3 colon cancer, assuming an exponential distribution with a 3-year DFS rate of 75% in the control arm and 84% in experimental arm (HR = 0.6), we found that 170 events are required to power the study with a one-sided alpha = 0.025 and beta = 0.1. If the patients received a scan every 3 months or 6 months, the study power is maintained. If scan frequency decreases to every 12 months, 2 additional events are required to maintain the study power. For stage 4 colorectal cancer, assuming the exponential distribution with median PFS equal to 2 months for the control arm and 4 months for the experimental arm (HR = 0.5), 90 events are required to provide 90% power with one-sided alpha = 0.25. For scan frequency of every 1 month, 6 additional events are required to maintain the study power. For scan frequency of every 2 and 3 months, which are standard of care, 14 and 26 additional events are required to maintain the study power.
**Conclusions**
A sparse CT scan frequency can affect the study power requiring additional events to maintain the study power. The loss of power can be quantified using a numerical approximation of the underlying distribution function. The effect of interval-censoring on accuracy of the time-to-event estimate is not the focus of this study but should be investigated to insure the point estimate is not horrendously biased when the scan interval lengthens.

### O109

#### Ensuring research quality in care home trials: learning from NHS governance procedures

Amanda Lilley-Kelly, Rachael Kelley, Liz Graham, Vicki McLellan, Ian Wheeler, Suzanne Hartley
Leeds Institute of Clinical Trials Research
**Correspondence:** Amanda Lilley-Kelly
*Trials* 2017, **18(Suppl 1):**O109

The National Health Service (NHS) has established procedures to support research activities covered by the Research Governance Framework (RGF). These include Health Research Authority (HRA) approval to ensure regulatory compliance, and site-specific Research and Development (R&D) agreement to support project delivery (capacity/capability). In addition, the importance of research has been embedded in the NHS constitution (including Frameworks and Pledges) endorsed by the UK Government. The National Institute for Health Research (NIHR) Research Networks have also been established to embed Research infrastructure - primarily in the NHS setting -supporting research activities (trained research practitioners) and funding via the Research Capability Funding (RCF). Research in non-NHS settings, such as Care Homes, is increasing however standardised procedures to support the delivery of high quality research have yet to be embedded in practice. Complexities arise in this setting as Care Homes are often independent businesses which vary in infrastructure with often a unique mix of funding streams including Local Authorities, NHS (Continuing Healthcare), and self-funding - therefore relevant permissions will vary dependent upon the sites selected. Work has been done within the social care setting to bridge the gap, with the development of the “Implementation plan for research governance in social care”, however the scope and applicability to Care Home research varies with no fixed format for formal research permissions. Existing literature highlights the barriers to implementing the principles of the RGF in the Care Home setting, often referencing experiences from individual projects. However these experiences have yet to be consolidated into a robust summary of complexities with strategies to manage them in practice whilst still ensuring the delivery of quality research. We will present our experience from two randomised controlled trials within the care home setting alongside findings from a review of existing literature regarding trials implementation in care homes. We will also outline an approach developed to support the delivery of high quality research projects in this setting, summarising the key considerations for successful delivery in the absence of research infrastructure, the impact on timelines, resource use, and trial implementation. We will also discuss in more detail the key considerations during trial set-up, such as: i) Development and completion of formal permissions (Care Home Letter of Agreement) to document requirements for trial delivery at site, ii) Using quality/performance markers to inform site selection and ongoing assessment of contextual factors impacting on trial delivery; iii) Selection of appropriate sites (eligibility/feasibility/recruitment) reflective of the setting, iv) Developing processes to deliver the project in line with the principles of GCP (i.e. documented informed consent, data storage and transfer in accordance with DPA), and v) Incentives which sustain Care Home engagement, vi) Establishing information available within care records and development of appropriate data collection tools to support trial analysis. We have identified a series of challenges and proposed solutions which may help with future research in the Care Home setting and provides a useful resource for researchers to deliver high-quality research in a research naïve setting.

### O110

#### Assessing implementation fidelity in clinical trials of behaviour change interventions

Elaine Toomey[1], James Matthews[2], Deirdre Hurley[2]
[1]National University of Ireland Galway; [2]University College Dublin
**Correspondence:** Elaine Toomey
*Trials* 2017, **18(Suppl 1):**O110

**Background**

Previous research published in the Lancet has estimated that as much as 85% of healthcare research might actually be wasted. A contributing factor to this may be interventions that are delivered with poor fidelity within clinical trials. Intervention fidelity is the extent to which an intervention is delivered as intended by its developers, and assessing it is crucial as it increases confidence that changes in study outcomes are due to the effect of the intervention itself and not due to variability in implementation. Complex interventions involve several aspects with the potential to be implemented variably, therefore it is important to ensure a comprehensive evaluation of the fidelity of intervention delivery in such interventions. However, fidelity continues to be poorly assessed in clinical trials across multiple disciplines. At present there is a lack of pragmatic guidance and an insufficient focus on developing feasible ways of incorporating fidelity assessment into clinical trials.

**Objectives**

This research aimed to provide guidance on assessing fidelity of intervention delivery within clinical trials, i.e. to inform appropriate choice of fidelity assessment methods and how to achieve a balance between comprehensiveness and feasibility. Specifically, the study aimed to do this through establishing the fidelity of delivery of a behaviour-change physiotherapy-led intervention within the context of a clinical feasibility randomised controlled trial (RCT) and to explore the potential factors that may have influenced these fidelity results.

**Methods**

This mixed methods study was a convergent triangulation design. 60 intervention sessions were delivered across seven sites by nine physiotherapists. Fidelity was assessed quantitatively using audio-recordings (n = 60), direct-observations (n = 24) and self-report checklists (n = 60) and qualitatively using individual semi-structured interviews with all physiotherapists (n = 9). Quantitative data were analysed using means and standard deviations. Qualitative data were analysed using thematic analysis. Integration of qualitative and quantitative data occurred at an interpretation level using a triangulation methodology.

**Results**

Quantitatively, the fidelity scores were high for all assessment methods; with self-report (92.7%) consistently higher than direct-observations (82.7%), or audio-recordings (81.7%). There was significant variation between physiotherapists' fidelity scores but all scored above 50%. These findings were corroborated by the physiotherapist interviews. In terms of factors influencing fidelity, both qualitative and quantitative data found that physiotherapists' knowledge and previous experience may have influenced their fidelity of the delivery. The physiotherapist qualitative data additionally showed that participant-level and programme-level factors also influenced their fidelity.

**Conclusions**

The research identified a number of key findings that can enable a feasible and comprehensive assessment of fidelity in clinical trials. Although conducted in the context of a feasibility RCT of a complex behavioural intervention, these findings can be generalised to other areas. Firstly, a 'spectrum' of quantitative assessment methods exists and choice may be guided by specific trial factors (e.g. size, resource availability). Secondly, a mixed methods approach provides a more comprehensive assessment of fidelity and can be feasibly done by utilising pre-existing trial qualitative data collection. Finally, assessing fidelity and its influencing factors can help understand how and why adaptations and deviations have occurred and can guide potential refinements.

### O111

#### An evaluation of constrained randomization for the design and analysis of group-randomized trials with binary outcomes

Fan Li[1], Elizabeth Turner[1], William Vollmer[2], David M. Murray[3], Patrick J. Heagerty[4], Elizabeth R. DeLong[1]
[1]Duke University; [2]Kaiser Permanente; [3]National Institutes of Health; [4]University of Washington
**Correspondence:** Fan Li
*Trials* 2017, **18(Suppl 1):**O111

Group-randomized trials (GRTs) are randomized studies that allocate intact groups of individuals to different comparison arms. A frequent practical limitation to adopting such research designs is that only a limited number of groups may be available, and therefore, simple randomization is unable to adequately balance multiple group-level covariates between arms. Therefore, covariate-based constrained randomization was proposed as an allocation technique to achieve this. Constrained randomization involves generating a very large number of possible allocation schemes (with a small number of groups to be randomized it is possible to generate all of them), calculating a pre-specified balance metric that assesses covariate imbalance, limiting the randomization space to a pre-specified percentage of those possible and randomly selecting one randomization to implement. However, related statistical issues on testing for intervention effect under such designs have not been thoroughly studied with binary outcomes. Motivated by two recent trials, we conduct a series of Monte Carlo simulations to evaluate the statistical properties of two model-based F-tests (linearization and likelihood F-test) and two randomization-based permutation tests (residual and likelihood P-test) under both simple and constrained randomization designs, with varying degrees of analysis-based covariate adjustment. Our results indicate that constrained randomization improves the power of linearization F-test and two permutation tests when the prognostic group-level variables are controlled for in the analysis and the size of randomization space is reasonably small. We also demonstrate that constrained randomization reduces power loss from redundant analysis-based adjustment for non-prognostic covariates. Design considerations such as the choice of the balance metric and the size of randomization space are emphasized throughout.

### O112

#### A predictive probability interim design for phase ii clinical trials with continuous endpoints

Meng Liu, Emily V. Dressler
Department of Biostatistics, College of Public Health, University of Kentucky
*Trials* 2017, **18(Suppl 1):**O112

Correspondence: Meng Liu
*Trials* 2017, **18(Suppl 1):**O112

**Background**

Phase II clinical trials aim to potentially screen out ineffective and identify effective therapies to move forward to the randomized phase III setting. In phase II trials, the most common way of assessing tumor shrinkage is to dichotomize the patients by the response rate according to RECIST. Besides loss of statistical efficiency, studies have shown that designs using response rate require much more patients than those using continuous tumor size shrinkage. Further, drugs can be still active even if they do not lead to high levels of tumor regression, as could be observed with molecular targeted therapies and immunotherapies. These treatment strategies come often with lower toxicity profiles than traditional cytotoxic treatments, and have shifted the drug development paradigm into establishing evidence of biological activity, target modulation and pharmacodynamics effects of these therapies in early phase trials. As such, these trials need to address simultaneous evaluation of safety as well as proof-of-concept biological marker activity or changes in continuous tumor size instead of binary response rates. However, there is a lack of interim strategies developed to monitor futility and/or efficacy for these types of continuous outcomes, especially in the single-arm setting.

**Objective**

We propose a two-stage single-arm design for continuous endpoints that allows for early futility stopping while maintaining desirable statistical properties.

**Methods**

Lee and Liu (2008) developed a predictive probability design for binary outcomes in a single-arm phase II cancer clinical trial. We extend their design into a two-stage setting for continuous endpoints assuming a normal distribution with known variance. We evaluate and present the design properties for both optimal and minimax designs.

**Results**

Both simulation results and presented case study have demonstrated that the proposed design can incorporate an interim stop for futility that controls both type I and type II error rates. As expected, using continuous tumor size resulted in lower expected and maximum sample sizes. A limited exploration of the choice of prior was performed and the numeric results were shown to be robust.

**Conclusions**

The proposed two-stage design based on predictive probability is efficient with similar sample size reduction for continuous outcomes and possess desirable operating characteristics. As research rapidly moves to incorporate more immunotherapies and targeted therapies, it will accommodate new types of outcomes while allowing for flexible stopping rules for futility and/or efficacy to continue optimizing trial resources and prioritize agents with compelling early phase data.

### O113

#### Control of type I error rates in Bayesian group sequential designs

Haolun Shi, Guosheng Yin
University of Hong Kong
**Correspondence:** Haolun Shi
*Trials* 2017, **18(Suppl 1):**O113

Bayesian approaches to phase II clinical trial designs usually base the inferences on the posterior distribution of the parameter of interest. If the posterior probability is computed and assessed in a sequential manner, the design may involve the problem of multiplicity, which, however, is often a neglected aspect in Bayesian trial designs. To effectively maintain the overall type I error rate, we propose solutions to the problem of multiplicity for Bayesian sequential designs and, in particular, the determination of the cutoff boundaries for the posterior probabilities. We present both theoretical and numerical methods for finding the optimal posterior probability boundaries with alpha-spending functions that mimic those of the frequentist group sequential designs. The theoretical approach is based on the asymptotic properties of the posterior probability, which establishes a connection between the Bayesian trial design and the frequentist group sequential method. The numerical approach uses a sandwich-type searching algorithm, which immensely reduces the computational burden. We apply least-square fitting to find the alpha-spending function closest to the target. We discuss the application of our method to single-arm and double-arm cases with binary and normal endpoints, respectively, and provide a real trial example for each case.

### O114

#### Efficiency of recruitment to stroke rehabilitation randomised controlled trials: secondary analysis of recruitment data

Kris McGill[1], Marian Brady[1], Jon Godwin[1], Catherine Sackley[2]
[1]Glasgow Caledonian University; [2]Kings College London
**Correspondence:** Kris McGill
*Trials* 2017, **18(Suppl 1):**O114

**Background**

Efficient recruitment to randomised controlled trials (RCTs) is vital in high-quality, cost effective, clinical research. Under-recruitment, recruitment extensions and supplementary funding requests are common. Under-recruitment leads to underpowered trials, inconclusive results and wasted research effort. Efficient recruitment to stroke rehabilitation RCTs is considered particularly problematic but has yet to be investigated.

**Aims and Objectives**

We aimed to examine stroke rehabilitation RCTs' (i) recruitment efficiency (ii) trial features associated with recruitment efficiency and (iii) reporting of recruitment information.

**Methods**

We included all trials identified by the Cochrane Stroke Group's trial register. This includes trials identified from 35 electronic databases (e.g. Medline, CINAHL, EMBASE); numerous clinical trial registers; and hand-searching other resources. We included publications between 2005–2015, grey literature, and had no language restrictions. Records that reported RCTs of stroke rehabilitation non-pharmacological interventions with patient populations were included. We extracted recruitment efficiency data (i) Rate: numbers randomised as a percentage of those screened for eligibility (ii) Speed: average monthly recruitment numbers across sites (iii) overall Dropout rates. Data on recruitment sites, recruiters, setting, funding support, ethical review, intervention type, targeted impairment, control comparison, and country of recruitment were extracted by two independent reviewers. Discrepancies were resolved by a third reviewer.

**Results**

Two independent reviewers screened 12,939 titles, 1,270 abstracts and 788 full texts for eligibility. 515 trials were included. Only 39% of stroke survivors screened were subsequently randomised. Subgroup analysis revealed that recruitment efficiency was significantly affected by the intervention type, control condition, targeted impairment, recruitment time point and setting.

**Conclusions**

Stroke rehabilitation trials experience notable recruitment inefficiencies.

### O115

#### A review of dose escalation methods in first-in-human small molecule oncology clinical trials

Daniel Greenwood[1], Ian Stratford[1], Steven Booth[2]
[1]University of Manchester; [2]University of Hertfordshire
**Correspondence:** Daniel Greenwood
*Trials* 2017, **18(Suppl 1):**O115

**Background**

First-in-human clinical trials are a crucial stage in the development of novel oncology drug candidates. Despite their importance, the design of these trials varies significantly. An improved understanding of these variations is needed in order to improve the impact of cancer research and make the process more economical. The aim of this research was to review, analyse and compare the dose escalation methods used in first-in-human small molecule oncology clinical trials to provide greater understanding of the variation of oncology clinical trial design.

**Method**:

The literature databases PubMED, Web of Science and Wiley Online Library were searched to identify published first-in-human trials of small molecule oncology candidates. Search terms used were: [1] "first-in-man" [title] OR [2] "first-in-human" [title] OR [3] "oncology OR cancer". Further searches were completed which replaced terms [1] and [2] with [4] "dose escalation". A total of 69 trial publications applicable to the aim of this review were identified. Each publication was reviewed and relevant data extracted which included the type of dose escalation method and individual dose levels used. To enable comparative analysis of 3 + 3 and "accelerated trial design" (ATD) escalation methods (the most popular methods with trials of suitable number for comparison), dose level data was translated to "Unique Dose Levels" (UDLs). The mean number of UDLs and dose increments of 3 + 3 and ATD trials were then compared. We hypothesised that, given its accelerated purpose, trials which employed the ATD escalation method would have fewer dose levels and larger increments between doses and thereby achieve the maximum tolerated dose (MTD) quicker.

**Results**

Of the 69 trials identified for review, 64 were parallel multiple dose design and five were parallel single dose design. Of the parallel multiple dose trials, 54/64 used a rule based dose escalation method with only 4/64 opting for a model based dose escalation method. The most popular rule based dose escalation methods were 3 + 3 (31/54) and ATD (16/54). The mean number of UDLs of 3 + 3 and ATD trials did not differ significantly (9.4 vs. 8.5; p-value = 0.31 for two-tailed t-test with 95% confidence intervals). Through use of a graphical heat map method of analysis, the dose increments of ATD trials do not appear to be greater than those of 3 + 3 trials. Given these findings, the research hypothesis was disproven.

**Conclusion**

The findings of this research suggest that the ATD dose escalation method may not achieve the MTD quicker than the traditional 3 + 3 dose escalation method. If ATD is in fact not accelerated, this finding has implications for drug development organisations where a prolonged clinical trial process has large cost implications, and also for patients who may have to wait longer for drugs to reach the clinic. Future research will seek to compare the dose escalation methods used in first-in-human clinical trials of the same drug candidate (e.g. in Caucasian and Asian populations) to control the effect of drug variation on dose escalation whilst acknowledging and accounting for population differences.

### O116

#### Missing data in trial-based cost-effectiveness analysis: a review of the current practice

Baptiste Leurent, Manuel Gomes, James Carpenter
London School of Hygiene & Tropical Medicine
**Correspondence:** Baptiste Leurent
*Trials* 2017, **18(Suppl 1):**O116

**Background**

Cost effectiveness analyses (CEA) of randomised trials are an important source of information for health commissioners to decide on how to best allocate limited resources. Missing data are a common issue in trials, and is particularly problematic in CEA which require complete information on both costs and effects. Recent guidelines addressing this issue encourage the use of suitable methods such as multiple imputation and recommend the conduct of sensitivity analyses under varied missing data assumptions. It is however unclear to what extent these recommendations have affected practice. Aim: To review the extent of, and methods used to address, missing data in trial-based cost-effectiveness analysis.

**Methods**

We conducted a review of trial-based CEA published in the Health Technology Assessment journal between January 2013 and December 2015. This journal is a key depository of full-scale economic evaluations funded by the UK National Institute for Health Research Health Technology Assessment Programme (NIHR HTA), where each report includes details of the analysis methods and results.

**Results**

Fifty-two eligible trials were identified. Nearly all of them had missing data, with a median proportion of participants with complete cost-effectiveness data of 63% (IQR 47% to 81%). Restricting analysis to complete-records remains the most common approach (43% of primary analysis), followed by multiple imputation (30%). Only half of the studies conducted sensitivity analyses for missing data, and it was typically using a limited range of assumptions such as data being either “missing completely at random” or “missing at random”.

**Conclusions**

Missing data remain a major concern for trial-based CEA. In spite of its limitations, restricting analysis to the subset of complete records is the most common approach. Analysts typically do not follow guidelines in evaluating the robustness of inferences to departure from the missing at random assumption. There may be lack of lack of awareness of the issue, or guidance on how to conduct such analyses.

## Chalmers Finalists

### O117

#### Adaptive prediction of event times in clinical trials

Yu Lan[1], Daniel F. Heitjan[2]
[1]Southern Methodist University; [2]Southern Methodist University & UT Southwestern Medical Center
**Correspondence:** Yu Lan
*Trials* 2017, **18(Suppl 1):**O117

In event-based clinical trials it is common to plan interim analyses to take place at planned event counts. Accurate prediction of these event times can support trial planning and the efficient allocation of resources. Available methods to create such predictions include parametric cure and non-cure models and a nonparametric approach based on the Bayesian bootstrap. The parametric methods work well when their underlying assumptions are met, and the nonparametric method gives calibrated but inefficient predictions across a wide range of models. However, in the early stages of a trial, when predictions have the highest marginal value, there is insufficient data to provide evidence about the form of underlying model, including whether a cure fraction exists. In this paper, we propose an adaptive method to address this deficiency. The method draws predictions from the model with the highest Bayesian posterior probability within a range of candidate models. To further capture the uncertainty in clinical trial prediction, we apply a simulation strategy using the Bayesian bootstrap. A simulation study demonstrates that the adaptive method produces prediction intervals that have good coverage and are slightly wider than non-adaptive intervals but narrower than nonparametric intervals. It leads to some improvements in making predictions with data from the International Chronic Granulomatous Disease Study.

### O118

#### Nonparametric overdose control for dose finding in drug-combination trials

Chi Kin Lam, Ruitao Lin, Guosheng Yin
The University of Hong Kong
**Correspondence:** Chi Kin Lam
*Trials* 2017, **18(Suppl 1):**O118

With the emergence of novel targeted anti-cancer agents, drug combinations have been recognized as cutting-edge development in oncology. However, limited attention has been paid to the overdose control in the existing drug-combination dose-finding trials. We develop the multi-agent nonparametric overdose control (MANOC) design for dose finding in phase I drug-combination trials. Based on a Bayesian decision-theoretic approach, we control the probability of overdosing in a local region at the current dose combination. Simulation studies are conducted to investigate the performance of the proposed design. While the MANOC can prevent patients from being allocated to overtoxic dose levels, its accuracy and efficiency are still competitive to the existing designs. As an illustration, the MANOC is applied to a phase I clinical trial for identifying the maximum tolerated dose combination of buparlisib and trametinib.

### O119

#### Biomarker stratified design enriched by auxiliary variables

Ting Wang[1], Xiaofei Wang[2], Haibo Zhou[1], Jianwen Cai[1], Stephen L. George[2]
[1]University of North Carolina at Chapel Hill; [2]Duke University
**Correspondence:** Ting Wang
*Trials* 2017, **18(Suppl 1):**O119

**Introduction**

In precision medicine, drugs are developed to target patients with certain genetic profiles. Targeted trials test treatment benefit only in the biomarker-positive patients. Trials with a biomarker-stratified design (BSD) allow a complete assessment of the effect of the new drug relative to the standard drug overall as well as in various biomarker-defined subgroups. However, a BSD trial often requires enrolling a large number of patients, especially when the proportion of the biomarker positives is small and thus the conduct of a BSD trial is expensive when the cost of ascertaining the true biomarker status is high.

**Methods**

We propose a special type of biomarker enrichment design, Biomarker Stratified Design Enriched by Auxiliary Variables (ABSD), in which a subgroup of patients, typically the biomarker-positive patients, are enriched based on the value of an inexpensive auxiliary variable that is positively correlated to the true biomarker. In such a design, all auxiliary-variable-positive patients and a proportion of the auxiliary-variable-negative patients are selected and included in the randomized trial. We compared the efficiency of ABSD with BSD in estimating various treatment parameters that are estimable in a BSD trial including the treatment effect in all patients and in specific biomarker subgroups and the interaction effect. We compared the efficiency of the two designs in term of the number of treated patients and the cost of the trial, assuming a range of prevalence of the true biomarker-positive patients in the overall population, the positive predictive value of the auxiliary variables for the true maker, and configurations of cost utilities of various items in conducting such trials.

**Results**

The proposed ABSD always reduces the total cost of the trial relative to a BSD when the prevalence rate is small and the PPV, the probability that a patient with positive auxiliary variable also has a positive true biomarker, is large enough.

When employing the proposed design in a practical study, Gefitinib or Carboplatin-Paclitaxel in Pulmonary Adenocarcinoma in North America, for testing the treatment effect among EGFR mutants and the interaction effect, ABSD requires 155 randomized patients compared to the 930 randomized patients required by a BSD. In addition, ABSD reduces the total cost cost by 64.6%.

Another advantage of ABSD is that in most cases we can immediately randomize patients selected in the screening process without waiting for the result of true biomarker test, which can substantially reduce reduce the waiting time.

Since PPV plays a very important role in the proposed design, a Bayesian adaptive ABSD is also proposed to deal with the misspecified PPV.

**Conclusion**

A biomarker stratified design enriched by an auxiliary variable can be more efficient than the standard BSD design. The efficiency gain can be particularly significant when the auxiliary variable has a high PPV, the prevalence rate of the biomarker-positive subgroup is small and the cost of ascertaining the true biomarker status is high relative to the auxiliary variable.

**Publisher's Note**

Springer Nature remains neutral with regard to jurisdictional claims in published maps and institutional affiliations.

Submit your next manuscript to BioMed Central and we will help you at every step:

- We accept pre-submission inquiries
- Our selector tool helps you to find the most relevant journal
- We provide round the clock customer support
- Convenient online submission
- Thorough peer review
- Inclusion in PubMed and all major indexing services
- Maximum visibility for your research

Submit your manuscript at www.biomedcentral.com/submit

BioMed Central

Download to read this full article text.

