# Correction to: Meeting abstracts from the 4th International Clinical Trials Methodology Conference (ICTMC) and the 38th Annual Meeting of the Society for Clinical Trials

**DOI:** 10.1186/s13063-017-2392-7

**Published:** 2018-02-09

**Authors:** Steven Penegar, Rebecca Lewis, James Catto, Joanne Cresswell, Leyshon Griffiths, Micki Hill, John Kelly, Allen Knight, John McGrath, Laura Wiley, Hugh Mostafid, Emma Hall

**Affiliations:** 1 The Institute of Cancer Research, Sutton, UK; 2 University of Sheffield, Sheffield, UK; 3 South Tees Hospitals NHS Foundation Trust, Middlesbrough, UK; 4 University Hospitals of Leicester NHS Trust, Leicester, UK; 5 University College London, London, UK; 6 Patient representative, London, UK; 7 Royal Devon & Exeter NHS Foundation Trust, Exeter, UK; 8 Royal Surrey County Hospital NHS Foundation Trust, Guildford, UK

## Correction

After publication of this supplement [1], it was brought to our attention that two authors were missing for abstract P66: *Recruitment aids for a phase II randomised trial in low risk bladder cancer*. The two missing authors can be found in this Erratum.

Hugh Mostafid (Royal Surrey County Hospital NHS Foundation Trust)

Emma Hall (The Institute of Cancer Research)

P66

Recruitment aids for a phase II randomised trial in low risk bladder cancer

Steven Penegar^1^, Rebecca Lewis^1^, James Catto^2^, Joanne Cresswell^3^, Leyshon Griffiths^4^, Micki Hill^1^, John Kelly^5^, Allen Knight^6^, John McGrath^7^, Laura Wiley^1^, Hugh Mostafid^8^, Emma Hall^1^

^1^The Institute of Cancer Research; ^2^University of Sheffield; ^3^South Tees Hospitals NHS Foundation Trust; ^4^University Hospitals of Leicester NHS Trust; ^5^University College London; ^6^Patient representative; ^7^Royal Devon & Exeter NHS Foundation Trust; ^8^Royal Surrey County Hospital NHS Foundation Trust Correspondence: Steven Penegar Trials 2017, 18(Suppl 1):P66

Background Non-muscle invasive bladder cancer (NMIBC) is a locally recurring disease for which patients undergo long term surveillance following initial diagnosis. CALIBER is a multicentre phase II feasibility study comparing intravesical chemotherapy (chemoresection) with surgery (standard of care) in patients with recurrent low risk NMIBC (2:1 chemoresection:surgery randomisation). The primary aim is to assess complete response to chemoresection and the trial is randomised to test feasibility of recruitment to a larger randomised phase III trial. It was anticipated that patient recruitment would be challenging due to the need to identify potential participants at the time of recurrence prior to treatment, complex risk stratification criteria and varied treatment pathways across participating sites. As such we developed recruitment aids with the aim of raising awareness amongst potential participants, ensuring site staff remain aware of the trial and promoting effective liaison between site staff when suitable patients are identified.

Methods From the outset of the trial, ethics approved short patient information leaflets and posters have been available to highlight the trial to patients attending surveillance visits. A staff poster was also provided to raise awareness amongst staff conducting surveillance. A CALIBER specific risk calculation tool was introduced in March 2016 as an aid to assess eligibility. We surveyed 34 participating centres about their use of these aids and their use of the tools was compared to their average recruitment.

Results Responses were received from 26/34 centres. 25/26 (96%) are using at least one of the short patient information leaflet, patient poster, clinician poster or eligibility. Average monthly recruitment does not appear to increase with increased use of the tools, with a median recruitment of 0.21 for the 8/26 (31%) sites using two tools and 0.03 for the 6/26 (23%) sites using all four. Since distributing the CALIBER risk calculator, the number of eligibility queries received by the coordinating clinical trials unit has substantially decreased. Initial feedback from centres suggests it is a useful tool for local pre-screening. Centres are advised to print the Trials 2017, 18(Suppl 1):200 Page 26 of 235 completed score calculation and retain in the patient notes to document this eligibility assessment.

Limitations The impact of introduction of different tools on recruitment could not be confirmed as most have been available since the trial commenced. The reduction in eligibility queries since introduction of the recurrence calculation tool may be a result of increased centre experience. In addition, the use of tools may be confounded with factors such as centre size and frequency of patient screening for the trial.

Conclusions With provision of targeted recruitment aids, centre staff training and ongoing support from the coordinating clinical trials unit, potential barriers to recruitment in a trial with challenging patient identification pathways and complex eligibility criteria can be managed effectively. However there is no obvious increase in recruitment with increased use of recruitment aids. In order to robustly evaluate the impact of recruitment aid interventions they should be introduced in a controlled manner to facilitate assessment of within and between centre pre- and post- intervention accrual rates.
